# The development of sulfonated terpyridine ligands for control of regioselectivity in palladium-catalysed fluorination of anilides†

**DOI:** 10.1039/d5sc05799j

**Published:** 2025-09-17

**Authors:** Jiri Dolezel, Robert J. Phipps

**Affiliations:** a Yusuf Hamied Department of Chemistry, University of Cambridge Lensfield Road Cambridge CB2 1EW UK rjp71@cam.ac.uk

## Abstract

Fluorinated arenes are ubiquitous in synthetically and medicinally valuable compounds. Direct fluorination of arene C–H bonds is an attractive disconnection to these targets, but methods are far more restricted than analogous bromination or chlorination and those that do exist often yield inseparable mixtures of regioisomers. We describe the superimposition of a non-covalent directing strategy to a recently reported method for palladium-catalysed arene fluorination which utilised terpyridine ligands on the key reactive complex. We have synthesised and evaluated a range of terpyridine ligands that bear a pendant sulfonate group at various different positions with the aim of engaging in hydrogen bonding with an anilide substrate to impact the otherwise non-selective *ortho*/*para* ratio that is typically obtained with standard terpyridine ligands. This has enabled the identification of a ligand that can enable *para*-selectivity in the fluorination of a range of trifluoroacetanilides. We envisage this will be of use in selective arene fluorination but also demonstrates the potential of targeted non-covalent strategies for control of regioselectivity in transition metal catalysis.

## Abstract 2

A sulfonated terpyridine ligand for palladium enables regioselective anilide fluorination by exploiting ligand-substrate hydrogen bonding interactions.

## Graphical abstract

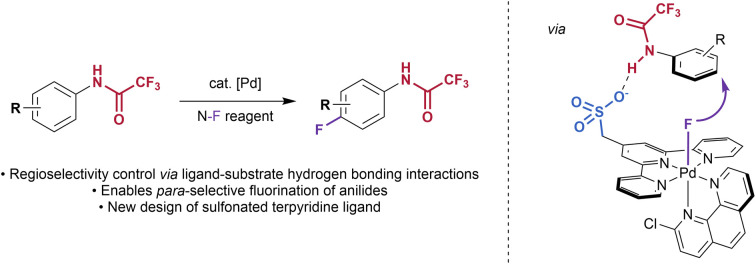

## Introduction

**Fig. 1:**
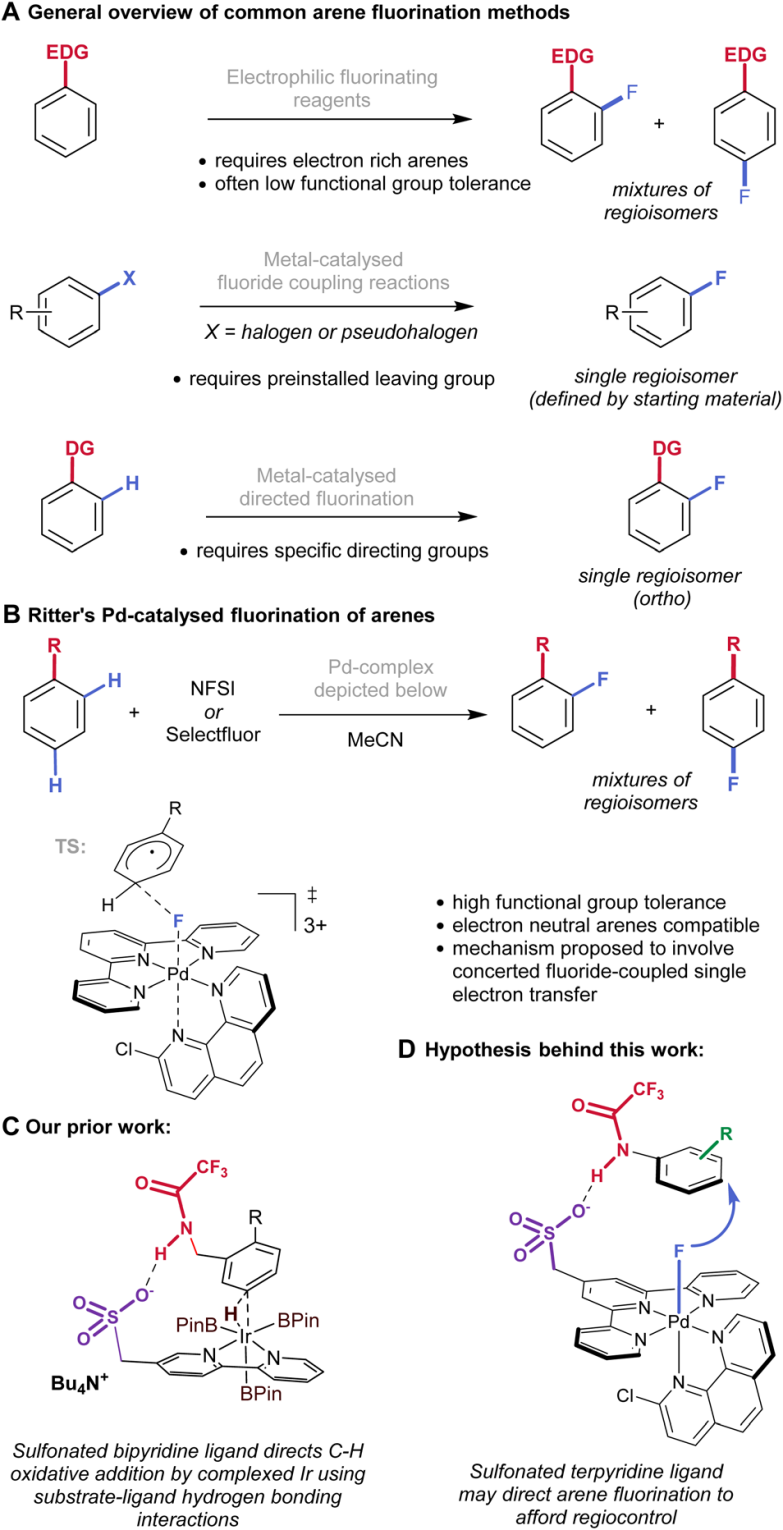
Background and hypothesis behind the present work. (A) Overview of arene fluorination methods. (B) Ritter's method using Pd catalysis. (C) Our prior work. (D) Present hypothesis.

Fluorinated arenes are ubiquitous in modern synthetic, biological and medicinal chemistry.^1^ Despite their importance, their preparations can be challenging because direct electrophilic fluorination is far more limited in scope than analogous halogenation methods such as bromination or chlorination.^2^ Whilst this can be achieved, it usually requires very electron-rich substrates and leads to non-selective regioisomeric mixtures (Fig. 1A, upper).^3^ An alternative strategy to access fluorinated arenes is to use Cu- and Pd-catalysed (or mediated) couplings where regioselectivity is not an issue, but a leaving group must necessarily be pre-installed in the desired position, requiring prior steps (Fig. 1A, middle).^4^ Extensive research into metal-catalysed C–H functionalisation has led to a number of directed C–H fluorination methods.^5^ These methods have the advantage of delivering the fluorinated products in a highly regioselective manner, but the nature of the directing process mean that these are usually restricted to the arene *ortho*-position.^6^ Additionally, the necessity for installation and removal of directing groups adds synthetic steps (Fig. 1A, lower).

In 2018 Ritter and co-workers reported a novel catalytic system for arene fluorination which used an *in situ* formed Pd(iv)–F complex and was proposed to operate in a manner akin to electrophilic aromatic substitution, described as fluoride-coupled SET, in which there is no proposed intermediate (Fig. 1B).^7^ In the reactive complex, palladium is complexed by a tridentate terpyridine ligand and a bidentate phenanthroline ligand. Their method was notable in its compatibility with electron neutral and even mildly electron-deficient substrates whilst simultaneously exhibiting a high functional group tolerance that would not be feasible with traditional fluorinating reagents. An outstanding challenge that remained unaddressed was regioselectivity, since in most cases mixtures of fluorinated arene isomers were formed.

We have previously experimented with regioselectivity control in Ir-catalyzed C–H borylation by designing ligand/substrate combinations that enable attractive non-covalent interactions to be realised at the selectivity-determining transition state, thereby exerting control through catalyst design.^8^ This is a strategy that was first applied to borylation by Kuninobu, Kanai and co-workers using hydrogen bonding.^9^ Our approach used sulfonated bipyridine ligands for iridium and showed that these could engage in either ion-pairing or hydrogen bonding interactions to realise *meta*-selective borylation (Fig. 1C, hydrogen bonding depicted). We speculated that a related approach may be applicable to Ritter's arene fluorination reaction with the aim of addressing the regioselectivity constraints.^10^ The selectivity determining-transition state is proposed to involve the palladium complex ligated by a terpyridine ligand and we hypothesised that modifying the ligand to incorporate a sulfonate group may permit intermolecular hydrogen bonding with a substrate bearing an appropriate donor to afford the products with enhanced regioselectivity for the *para* position over *ortho* (Fig. 1D).

## Results and discussion

### Evaluation of sulfonated terpyridine ligands

We set out to obtain a varied selection of sulfonated terpyridines, in which the location of the sulfonate group within the scaffold as well as the linker are systematically modulated (Table 1, L1–L9). The ligands were generally accessed either *via* Kröhnke condensation^11^ for symmetrical terpyridines or Negishi cross-coupling^12^ and all were finally isolated as their tetrabutylammonium salts to ensure solubility. L1, L2 and L3 each feature a methylene linker with the functionality located on the C4, C3 or C2 position of one external pyridine, respectively. L4–L9 all feature functionality extending from the C4 position of the internal pyridine. Whilst L4 and L5 feature one and two methylenes in the linker respectively, L6 contains a more conformationally-constrained *ortho*-phenyl linker. L7–L9 retain the phenyl linker but add an additional methylene unit between this and the sulfonate with the position of the methylenesulfonate being varied across all three possible positions of the phenyl.

**Table 1:** Ligand and substrate evaluation

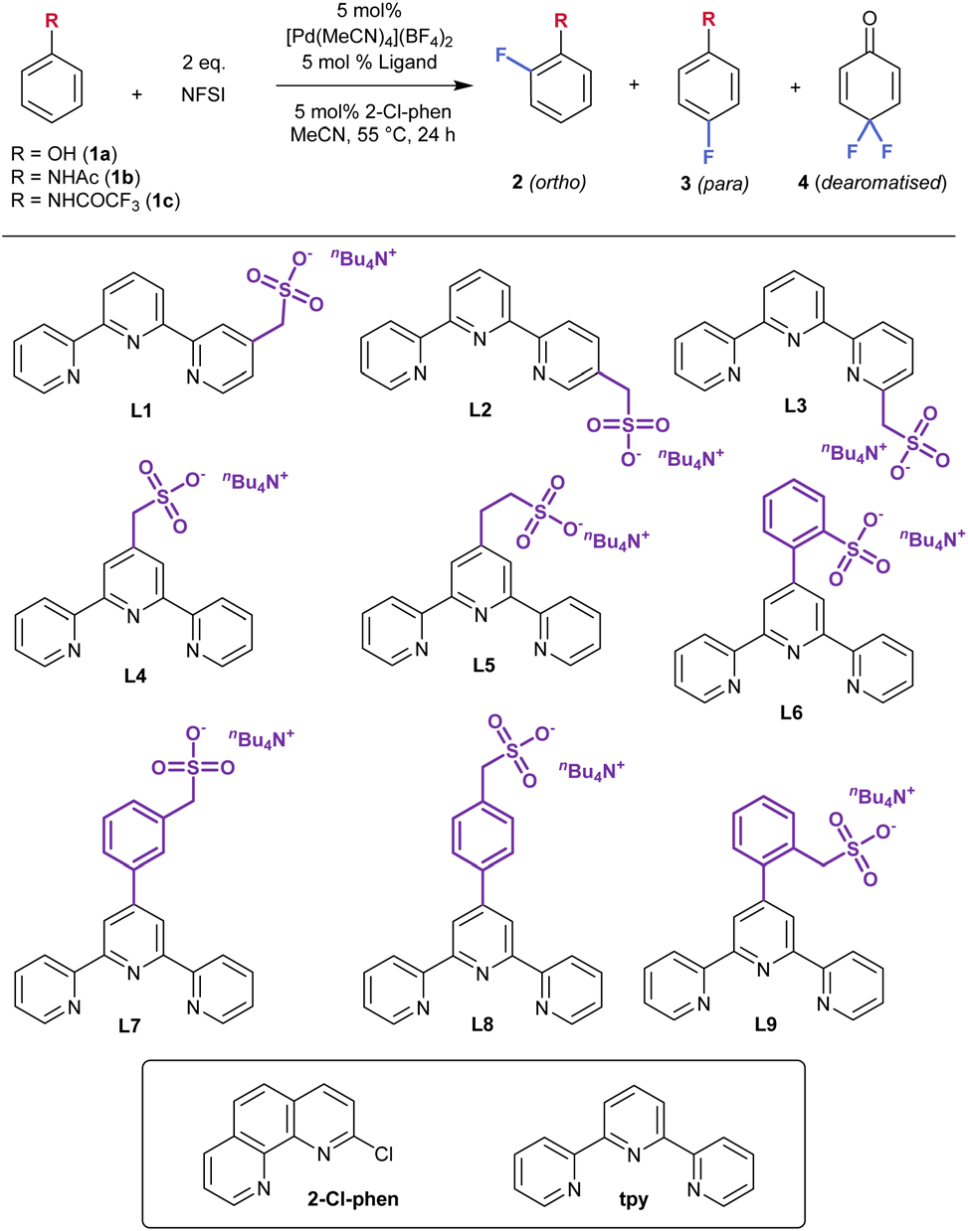					
Entry	Substrate	Ligand	Total yield^a^ [%]	Ratio 2 : 3 : 4^a^	*o*- : *p*-ratio^b^
1	PhOH 1a	tpy	27	8 : 11 : 8	1 : 1.4
2	PhOH 1a	L4	42	5 : 17 : 20	1 : 3.4
3	PhNHTFA 1c	tpy	13	3 : 8 : 2	1 : 2.7
4	PhNHTFA 1c	L1	39	6 : 27 : 6	1 : 4.5
5	PhNHTFA 1c	L2	18	4 : 13 : 1	1 : 3.3
6	PhNHTFA 1c	L3	6	2 : 4 : 0	1 : 2.0
7	PhNHTFA 1c	L4	30	4 : 25 : 1	**1 **:** 6.3**
8^c^	PhNHTFA 1c	L5	37	25 : 12 : 2	2.1 : 1
**9**	PhNHTFA 1c	L6	16	7 : 8 : 1	1 : 1.4
10	PhNHTFA 1c	L7	14	5 : 8 : 0	1 : 1.6
11	PhNHTFA 1c	L8	11	6 : 4 : 1	1.5 : 1
12	PhNHTFA 1c	L9	25	8 : 15 : 2	1 : 1.9

a The total yield (2 + 3 + 4) and 2 : 3 : 4 ratio were determined by ^19^F NMR coupled with ^1^H nuclei using 1,3-difluorobenzene or 4,4′-difluoroacetophenone as internal standard.

b Quoted *o*- : *p*-ratio is 2 : 3 and does not include dearomatised product 4.

c The reaction was carried out using Pd(acac)_2_ (5 mol%).

We first examined fluorination of phenol, constituting an electron-rich arene bearing an excellent hydrogen bond donor. Ritter had shown that the active catalyst could be assembled *in situ* by combining all three components – a Pd source, terpyridine ligand and 2-chlorophenanthroline.^7^ Before examining the sulfonated ligands, we assessed the outcome using simple terpyridine (tpy, Table 1, entry 1) and obtained an approximately equal mixture of 2a (*ortho* fluorination), 3a (*para* fluorination) and 4 (dearomatized product, presumably resulting from fluorination of 3a followed by hydrolysis). Upon evaluation of L1–L9 for phenol fluorination it was apparent that dearomatising difluorination to form 4 was competitive using many ligands, suggesting this would not be productive for obtaining the desired fluoroarene products (see SI, Table S1 for full details). Interestingly, one sulfonated ligand, L4, gave a selectivity outcome that deviated significantly from the tpy control (Table 1, entry 2). Disregarding 4, as expressed in the right hand column, showed *para* selectivity using L4 to be significantly higher than with tpy (*o*- : *p*- 1 : 3.4 *vs.* 1 : 1.4). If 4 were to be included with 3a as a consequence of *para*-directed fluorination by the catalyst, then these ratios would be 1 : 2.4 for tpy and 1 : 7.4 for L4, again suggesting that L4 may be promoting fluorination at the *para* position of the phenol. Acetanilide (1b) was found to be reactive but dearomatizing difluorination to give 4 was again competitive with desired monofluorination (see SI, Table S1 for details). Trifluoroacetanilide (1c) was less susceptible to over-fluorination and evaluation of the sulfonated ligand library on this substrate gave an accurate readout of *ortho*/*para* selectivity (Table 1, entries 3–12). It was notable that the control tpy ligand gave 1 : 2.7 ratio in favour of *para*, although the yield was low at only 13% (entry 3). Evaluation of ligands L1–L3 showed that L1, with the methylenesulfonate group extending from the C4 position of a peripheral pyridine, was the most effective of the three. The yield was significantly improved to 39%, with *para*-selectivity increased to 4.5 : 1 (entry 4) while both metrics were inferior with the C2 and C3 isomeric ligands (entries 5 and 6). Moving the methylene sulfonate group onto the central pyridine in L4 improved *para*-selectivity further, now reaching 6.3 : 1, with the yield comparable to that with L1 (entry 7). Extending the linker length in L5 interestingly favoured the *ortho* isomer, albeit only in a 2 : 1 ratio (entry 8). L6–L9 bearing phenyl linkers did not yield fruitful selectivity outcomes (entries 9–12). With L4 delivering significant *para*-selectivity enhancement compared with the other ligands, as well as the control, we next set out to fully optimise the reaction to increase yield.

### Reaction optimisation using L4

Having identified L4 as producing significantly increased *para*-selectivity compared with the control ligand tpy, we sought to improve the yield of the reaction. We evaluated a dozen solvents but none gave a superior outcome compared with MeCN (see SI, Table S2). A survey of 11 Pd sources revealed that Pd(acac)_2_ increased the reaction yield to 50% without impacting the regioselectivity (see SI, Tables S3 and 2, entry 1). Using L4 in combination with Pd(acac)_2_ we proceeded to evaluate nineteen different phenanthroline co-ligands (see SI, Table S4). This survey revealed that only 2-monosubstituted phenathrolines were effective and that alteration of this substituent away from chlorine failed to provide improvement. Alternative fluorinating reagents or modulation of equivalents of NFSI did not result in further improvement (Table S5). Variation of reaction concentration, time and temperature yielded the products in reduced yields and, in some cases, selectivity (Table S6). Ritter's study also used a preformed Pd-terpyridine complex, complex 1, and we prepared this as well as the analogous complex 2, incorporating L4 (Table 2). Complex 2 is partially zwitterionic due to the pendant sulfonate group and we found that it was necessary to generate this with a triflate counterion, rather than tetrafluoroborate, to permit purification of the complex by precipitation. Interestingly, tpy-containing complex 1 gave a 1.1 : 1 *o*- : *p*-ratio (Table 2, entry 2) which diverged from the 1 : 2.7 *o*- : *p*-ratio observed using the *in situ* formed complex (Table 1, entry 3). This could be attributable to complex 1 being more reactive, resulting it more 3a being depleted to form 4, impacting the final observed ratio of 2a : 3a. For complex 2, the outcome was almost identical to using the *in situ* formed protocol with L4, with only very small amounts of undesired 4 being formed (entry 4). Despite the moderate yield, which we were not able to improve further, the fluorination reaction using complex 2 delivers synthetically useful levels of regioselectivity for *para* mono-fluorination and minimal difluorination. It was noted in Ritter's report that separation of fluorinated arene isomers is extremely challenging and was typically accomplished on preparative HPLC. In the case of 3c we were able to isolate the *para*-fluorinated isomer on standard silica gel chromatography, albeit in a reduced yield (19%) due to close-running *ortho* isomer.

**Table 2:** Summary of further optimisation on 1c

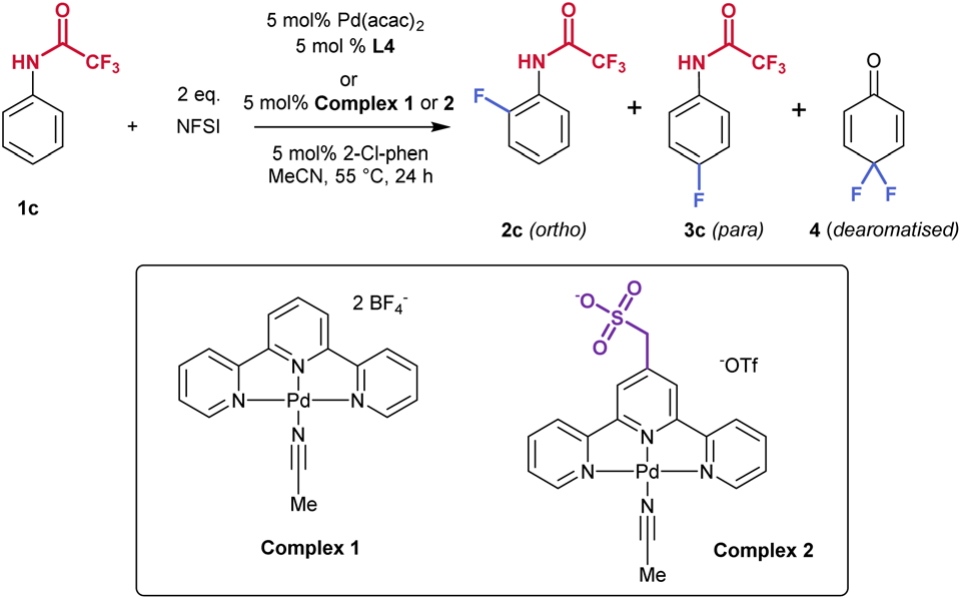					
Entry	Pd source	Ligand	Total yield^a^ [%]	Ratio 2 : 3 : 4^a^	*o*- : *p*-ratio^b^
1	Pd(acac)_2_	L4	50	6 : 38 : 6	1 : 6.3
2	Complex 1	—	33	17 : 16 : 14	1.1 : 1
**3**	**Complex**2	**—**	**50**	**6 : 41 : 3** c	**1 **:** 6.8**

a The yield and *o*- : *p*-dearomatised ratio were determined by ^19^F NMR.

b The *o*- : *p*-ratio does not include 4.

c 3c was able to be isolated cleanly in 19% yield following silica gel chromatography.

### Scope investigation

**Fig. 2:**
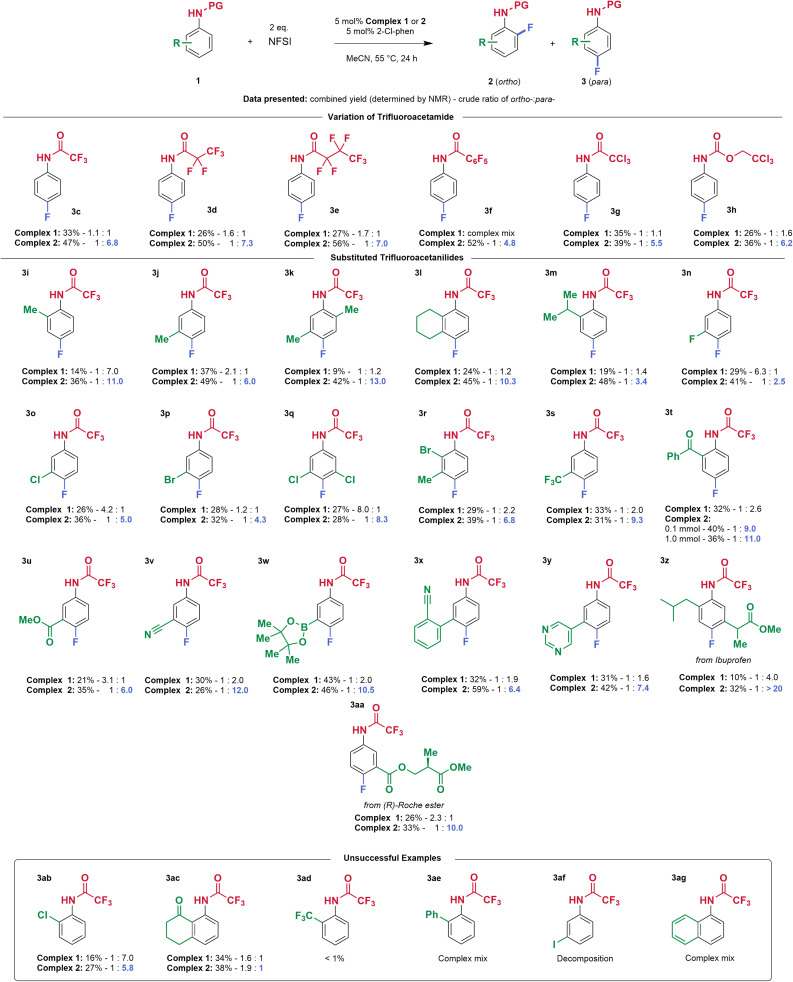
Reaction scope. The data is presented as follows: combined NMR yields of monofluorinated products – *ortho*- : *para*-isomer. The yields and *o*- : *p*-ratios were determined by ^19^F NMR coupled with ^1^H nuclei using 1,3-difluorobenzene or 4,4′-difluoroacetophenone as an internal standard. Only the major *para*-isomer is depicted.

We next explored the reaction scope of variously substituted trifluoroacetanilides and used both complex 1 and complex 2 to enable the direct comparison of sulfonated ligand L4 with tpy as a control. Yields quoted are determined by NMR analysis with internal standard. In some cases, pure *para* isomer could be isolated by conventional silica gel chromatography. In other cases, a mixture of isomers and/or with starting material was obtained and characterised as such, as preparative HPLC was not available to us (see SI for details). Firstly, variation of the trifluoroacetamide protecting group was investigated. Extending the perfluorinated chain yielded the products 3d and 3e with slightly increased yields and *o*- : *p*-ratios (1 : 7.3 and 1 : 7.0 respectively). Replacing the perfluoroalkyl chain with perfluorophenyl gave a slightly poorer outcome but still improvement over the control, which delivered a complex mixture (3f). Alternative chlorinated protecting groups trichloroacetamide (3g) and Troc (3h) gave good *para*-selectivity compared with the control, although the yields were lower. In all cases, complex 1 was essentially non-selective. Moving on to arene substitution, the reaction tolerates *ortho*- and *meta*-methyl substitution to afford the *para*-fluorinated products with good regioselectivity (1 : 11 for 3i and 1 : 6 for 3j). Dialkyl substituted substrates 3k and 3l afforded the *para*-product very selectively (1 : 13 and 1 : 10 *o*- : *p*-ratios respectively), particularly noting the outcome with complex 1 (1 : 1 in both cases). A bulky 2-isopropyl substituent was also tolerated, albeit with reduced regioselectivity (3m). 3-Halogenated substrates 3n–3p gave slightly lower yields but importantly delivered quite different regioselectivity outcomes with complex 1 and 2. As an example, 3-chloroacetanilide (3o) gave inversion, from 4 : 1 *o*- : *p*-with complex 1 to 1 : 5 *o*- : *p*-with complex 2 (see next paragraph for detailed discussion on origin of this large apparent shift). Other halogenated substrates also participated (3q–3r). A strongly electron-withdrawing trifluoromethyl group could be tolerated (3s) as could a 2-benzoyl substituent (3t). The synthesis of 3t was conducted on 1.0 mmol scale, without significantly diminishing the yield and regioselectivity (36% and 1 : 11 *o*- : *p*-), however, the *para*-isomer could be only isolated as a mixture with the starting material. This substrate could potentially provide access to fluorinated benzodiazepine derivates.^13^ Other electron withdrawing functional groups could be tolerated and again showed significantly improved regioselectivity compared with the control (3u, 3v). In line with the high tolerance of functionality, as emphasised by Ritter, substrate 3w, bearing a boronate ester, underwent fluorination without issue, proceeding with very good regioselectivity using complex 2 (*o*- : *p*- 1 : 11). Biphenyl substrate 3x performed well and no fluorination on the less-activated aromatic rings was observed. As emphasised in Ritter's work, the protocol also tolerates aromatic heterocycles: product 3y was obtained in 42% yield and *o*- : *p*- 1 : 7.4 (for comparison, complex 1 gave a 1 : 1 ratio). Substrates derived from ibuprofen (3z) and from (*R*)-Roche ester (3aa) yielded the *para*-fluorinated isomers with very good regioselectivity using complex 2 (*o*- : *p*- 1 : >20 and 1 : 10.0, respectively). With these substrates, complex 1 again showed poor selectivity and lower yields. These examples further emphasise the potential of the method for the late-stage functionalisation of complex molecules. Not all substrates were effective – 3ab and 3ac were obtained in a similar regioisomer distribution with both complexes (Fig. 2, inset box). Furthermore, the reaction does not tolerate a very electron withdrawing substituent at the *ortho*-position (3ad), or a phenyl, from which a complex mixture was obtained (3ae). An iodoarene or naphthalene, as in compounds 3af and 3ag, were not tolerated, leading to complex mixtures in both cases.

**Fig. 3:**
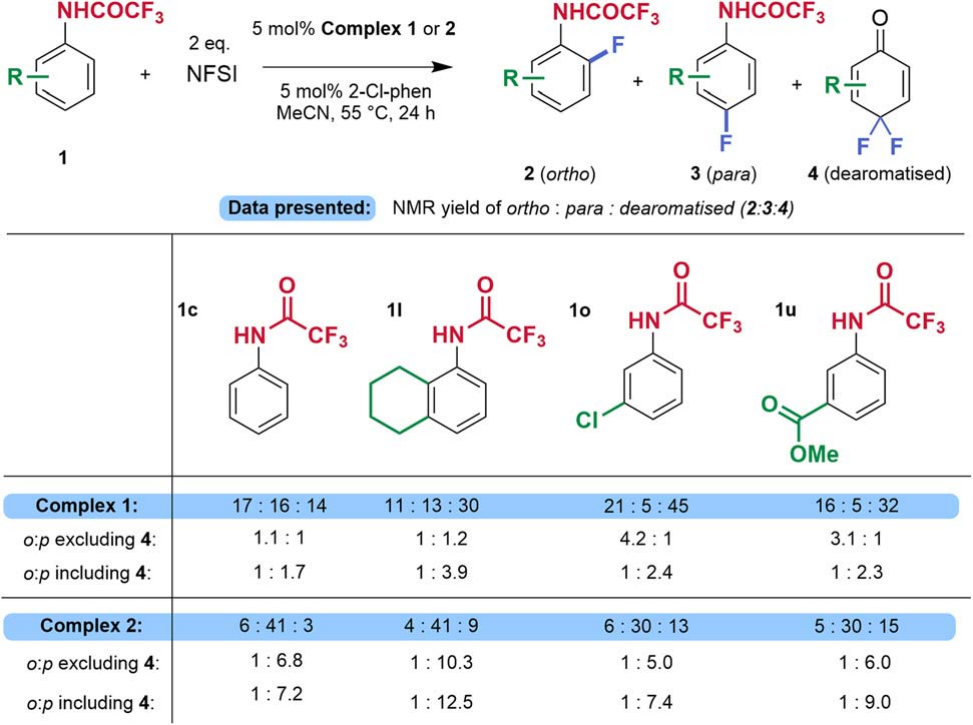
Detailed regioselectivity analysis of selected substrates that gave significant difluorination to form 4. Yields and ratios were determined by ^19^F NMR coupled with ^1^H nuclei using 1,3-difluorobenzene or 4,4′-difluoroacetophenone as the internal standard.

The selectivity ratios quoted in Fig. 2 reflect the *ortho* : *para* ratio of monofluorinated products (2 : 3) at the end of the reaction (24 h reaction time in all cases). Depending on the specific substitution pattern, dearomatising difluorination to form 4 was observed to different extents but was usually greater with non-sulfonated complex 1. Formation of 4, which arises from further fluorination of the *para*-fluorinated isomer 3 will inevitably impact the final ratio of 2 : 3 and we present further analysis of selected substrates in which formation of 4 was significant to show that, even taking this into account, complex 2 is still significantly impacting the overall *para*-selectivity (Fig. 3). In addition, complex 2 is also clearly impacting the chemoselectivity, favouring desired monofluorination and reducing undesired difluorination.

**Fig. 4:**
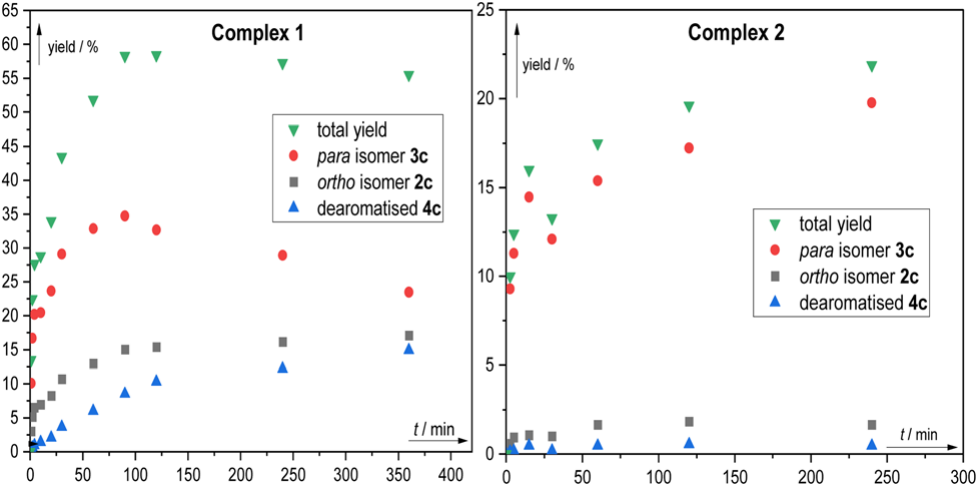
Reaction time course study in fluorination of 1c using (A) complex 1 and (B) complex 2. The yields were determined by ^19^F NMR coupled with ^1^H nuclei using 1,3-difluorobenzene. Shown data points are average of 2 measurements.

To visualise the evolution of the various fluorinated products as the reaction progresses, we carried out reaction time course studies on 1c with both complex 1 and complex 2 (Fig. 4). These revealed that for complex 1 the reaction is relatively fast and in the initial stages forms an approximately 1 : 2 ratio of 2 : 3 (*ortho* : *para*). The level of 3 quickly starts to deplete as 4 is formed and by the end of the analysis 2, 3 and 4 are evolving to similar concentrations. In contrast, with sulfonated complex 2 the reaction is highly regioselective from the outset, with very little of 2c or 4c forming although it is interesting that the rate of the reaction is noticeably slower than with complex 1.

### Probing ligand-substrate non-covalent interactions

**Fig. 5:**
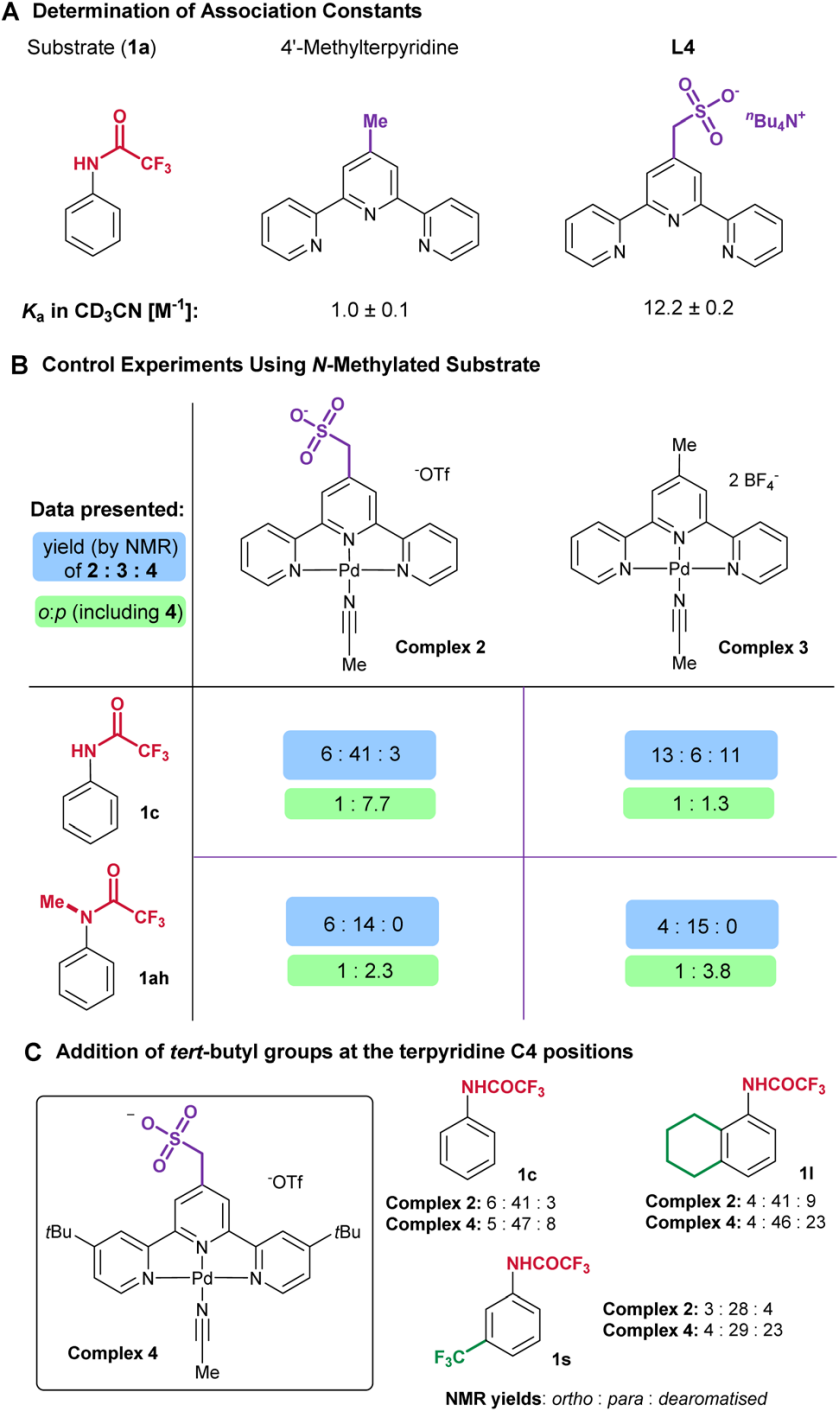
(A) Determination of association constants. (B) Control experiments using *N*-methylated substrate. (C) Complex 4 featuring *tert*-butyl groups and comparison with complex 2 across several substrates.

We sought to gain support for our hypothesis that the increased *para*-selectivity using the sulfonated complex 1 arises from intermolecular hydrogen bonding with the substrate. NMR titration in CD_3_CN showed there to be effectively no association between 1c and 4′-methylterpyridine (Fig. 5A, left). In comparison, the measured *K*_a_ with L4 was 12, demonstrating clearly the feasibility of hydrogen bonding between the two in the reaction solvent (Fig. 5A, right). We also carried out control experiments with *N*-methylated trifluoroacetanilide (1ah), which no longer possesses a hydrogen bond donor. Here we compared complex 2, containing the sulfonated terpyridine, and complex 3, containing 4′-methylterpyridine (Fig. 5B). Both complexes gave fairly similar selectivity outcomes on 1ah, slightly in favour of the *para* isomer. In comparison the difference between the two complexes on 1c is large and underscores that the best *para* selectivity is obtained by a proper match between substrate (1c) and ligand (complex 2).

### Evaluation of a di-*tert*-butyl-substituted version of complex 2

During the preparation of this manuscript, a detailed investigation into fluorination using complexes derived from complex 1 was reported by Sigman, Paton and co-workers.^14^ Through a variety of experimental and theoretical approaches they interrogated structural modulation of the heteroleptic Pd complexes. One outcome was that the introduction of *tert*-butyl groups at the C4 positions of each pyridine ring of the terpyridine ligand increased fluorination yields substantially. Furthermore, in certain substrates these groups could also increase *ortho*-selectivity, which was deduced to be a result of attractive dispersive interactions at the transition state for fluorination. Accordingly, we prepared complex 4 which differs from complex 2 by the addition of a *tert*-butyl group on each external pyridine (Fig. 5C). Substrates 1c, 1l and 1s were chosen as representative examples on which to evaluate complex 4, as they cover a range of electronics. It was immediately clear that complex 4 is more reactive than complex 2, as substantially more dearomatised 4 was formed in all cases. However, taking 4 into account when calculating the ratio of *ortho* : *para* fluorination, there was very little difference in terms of regioselectivity between the two complexes. These results are in line with Sigman and Paton's observations that the added *tert*-butyl groups increase reactivity. In our case it seems likely that substrate-ligand hydrogen bonding should override any weak dispersion interactions that might arise from the tertbutyl groups, so the *para* selectivity is not impacted.

## Conclusions

We have developed a strategy for *para*-selective fluorination of protected anilines by developing a new class of anionic terpyridine ligands, capable of hydrogen bonding with the substrate, and applying them to Ritter's Pd-catalysed fluorination method. The reaction tolerates a variety of functional groups to afford *para*-fluorinated products in synthetically useful yields and moderate to high regioselectivity. In all cases, control experiments using standard terpyridine emphasise the effectiveness of our strategy based on attractive non-covalent interactions. Initial investigations support the hypothesis of intermolecular hydrogen bonding being responsible for the observed regioselectivity and we corroborate the recent finding from Sigman, Paton and co-workers that addition of *tert*-butyl groups on the terpyridine increases reactivity in these complexes. Further applications of anionic terpyridine ligands for controlling selectivity *via* the secondary coordination sphere ongoing in our lab.

## Author contributions

The manuscript was written through the contributions of all authors and all authors have given approval to the final version.

## Conflicts of interest

There are no conflicts of interest.

## Supplementary Material

SC-016-D5SC05799J-s001

## Data Availability

The data supporting this article have been included as part of the SI. This includes additional optimisation data, synthesis and characterisation of compounds and experimental protocols. See DOI: https://doi.org/10.1039/d5sc05799j.
