# Supplementary material for: The development of sulfonated terpyridine ligands for control of regioselectivity in palladium-catalysed fluorination of anilides
Source: Chem Sci. 2025 Sep 17;16(42):19694–701. doi: 10.1039/d5sc05799j (PMC12461285; doi:10.1039/d5sc05799j)
Supplement: SC-016-D5SC05799J-s001 [file SC-016-D5SC05799J-s001.pdf]

Supplementary Information (SI) for Chemical Science.
This journal is © The Royal Society of Chemistry 2025

The Development of Sulfonated Terpyridine Ligands for Control of Regioselectivity in Palladium-Catalysed Fluorination of Anilides

Jiri Dolezel, Robert J. Phipps*

Yusuf Hamied Department of Chemistry, University of Cambridge, Lensfield Road, Cambridge, CB2 1EW, United Kingdom.

***Corresponding author:** rjp71@cam.ac.uk

Table of Contents

General Information 3
General Protocols 5
Additional Optimisation Data 9
Synthesis of Sulfonated Terpyridine Ligands 13
Synthesis of Pre-formed Palladium Terpyridine Complexes 35
Synthesis of 2-substituted 1,10-phenanthrolines 39
Synthesis of the Starting Materials 48
Characterisation Data for the Products 64
Synthesis of the Unsuccessful Starting Materials 96
Unsuccessful Substrate Scope Entries 100
Reaction on 1 mmol Scale 104
Synthesis and Fluorination of the *N*-Me Substrate 105
NMR Titrations 107
Kinetic Studies 110
NMR Spectra 113
References 457

General Information

Reaction Setup, Solvents and Reagents

Unless otherwise mentioned, all reactions were carried out under an inert atmosphere of nitrogen using the standard Schlenk line technique in oven-dried glassware. All starting materials and reagents were used as supplied from commercial sources without further purification unless stated otherwise. MeCN. $ZnCl_2$ was dried by melting under a vacuum using a heat gun prior to use. NBS was recrystallised from MeCN. Diisopropylamine was freshly distilled over finely ground NaOH. ^{n}BuLi was titrated against diphenylacetic acid, which was freshly recrystallised from hot toluene and dried under vacuum for one day.[1] Tetrabutylammonium hexafluorophosphate was recrystallised twice from absolute ethanol and dried under vacuum for 2 days. THF, Et_2O, MeCN and DCM were purified by distillation on site under an inert atmosphere via the following processes: THF and Et_2O were pre-dried over sodium wire and then distilled from CaH_2 and $LiAlH_4$. MeCN and DCM were distilled from CaH_2. Other anhydrous solvents were purchased from commercial sources and used as received. In the text below, "PE" refers to petroleum ether 40-60 °C.

Chromatography

Thin layer chromatography (TLC) was performed using 0.25 mm precoated Merck glass-backed silica gel plates (Silica gel 60 F254). The visualisation was by UV fluorescence (254 and 365 nm) and/or staining with iodine, potassium permanganate ($KMnO_4$), ninhydrin or 2,4-dinitrohydrazine. Flash column chromatography was performed using silica gel 60 (pore size: 60 Å, mesh: 40-63 μm) from Material Harvest or Fluorochem. In some cases, the silica gel was pre-treated (basified) with a solvent mixture including 2% of Et_3N.

NMR Spectroscopy

^{1}H NMR were recorded on 700 MHz TXO Cryoprobe, 600 MHz Bruker Avance DRX-600, 500 MHz Bruker DCH Cryoprobe, 400 MHz Bruker DPX-400 Dual, 400 MHz Avance III HD, 400 MHz Avance III HD Smart Probe or 400 MHz Neo Prodigy spectrometers. Chemical shifts (δ) are reported in parts per million (ppm), and the spectra are calibrated to the resonance resulting from incomplete deuteration of the solvent ($CDCl_3$: 7.26 ppm; CD_3CN: 1.94 ppm; $(CD_3)_2SO$: 2.50 ppm; $(CD_3)_2CO$: 2.05 ppm). ^{13}C NMR spectra were recorded on the same spectrometers with complete proton decoupling. Chemical shifts are reported in ppm with the solvent resonance as the internal standard ($^{13}CDCl_3$: 77.16 ppm, t; $^{13}CD_3CN$: 1.32 ppm; sept; $(^{13}CD_3)_2SO$: 39.52 ppm, sept; $(CD_3)_2CO$: 29.8 ppm, sept). ^{19}F NMR spectra with ^{1}H decoupling were recorded on 500 MHz Bruker DCH Cryoprobe, 400 MHz Avance III HD or 400 MHz Avance III HD Smart Probe. The ^{19}F chemicals shifts are in ppm and were indirectly referenced to the appropriate ^{1}H NMR spectra or with trifluorotoluene ($PhCF_3$), 1,3-difluorobenzene or 4,4'-difluorobenzophenone as internal standards ($PhCF_3$: −63.6 ppm; 1,3-difluorobenzene: −111.0

ppm; 4,4'-difluoroacetophenone: −108.6 ppm). Data are reported as follows: chemical shift δ, multiplicity (s = singlet, d = doublet, t = triplet, q = quartet, p = pentet, sext = sextet, sept = septet, br = broad, m = multiplet, app = apparent or combinations thereof (^{13}C signals are singlets unless otherwise stated), coupling constants J, number of nuclides (signals for all other nuclides except ^{1}H refer to one nuclide unless otherwise stated), assignment. ^{1}H NMR signals are reported in ppm to 2 decimal places, and all other nuclei are reported to 1 decimal place. Coupling constants (J) are quoted in Hertz (Hz) to one decimal place. ^{19}F NMR yields were obtained by recording ^{19}F spectra with ^{1}H coupling of the crude mixtures on 400 MHz Avance III HD using trifluorotoluene (3F, −63.6 ppm), 1,3-difluorobenzene (2F, −111.0 ppm) or 4,4'-difluorobenzophonenone (2F, −108.6 ppm). The NMR spectra of the fluorinated products isolated as mixtures have only the products integrated in the ^{1}H NMR. The isolated yields reported for the compounds obtained as mixtures are corrected from the data obtained by ^{19}F NMR of the mixture.

High Resolution Mass Spectrometry (HRMS)

Recorded on a Waters Vion IMS QTOF, Waters' Xevo G2-S bench top QTOF and AGILENT 6230 LC/TOF at the Department of Chemistry at the University of Cambridge. The ionisation method is noted as either positive or negative electrospray ionisation (+/−ESI). Measured values are reported to 4 decimal places and are within ± 5 ppm of the calculated value. The calculated values are based on the most abundant isotope unless otherwise stated in the chemical formula. For ions bearing more than a single unit of charge, the masses reported as "found" and "required" are the mass/charge ratios.

UV-vis Spectroscopy

The absorption spectra in anhydrous MeCN, DMSO and deionised water were obtained using a UV-vis spectrophotometer (Mettler Toledo UV5 spectrophotometer) with a matched 1.0-cm quartz cell. The concentration of the analyte was kept around 5×10^{-5} M.

Microanalysis

CHN combustion and ICP-OES data collection and analysis were performed by Dr Nigel Howard.

Optical Rotations

Measured in spectrophotometric grade methanol on a Perking Elmer 343 Polarimeter using a sodium lamp (λ = 589 nm, D-line). $[\alpha]_D$ values reported at stated temperature, with concentration in g/100 mL.

Naming and Numbering of the Compounds

Systematic names were generated by the commercially available software ChemDraw according to the guidelines specified by the IUPAC. Nevertheless, the numbering on the structure does not correspond to the systematic name.

General Protocols

General Procedure for Terpyridine Scaffold Assembly via Negishi Cross-coupling (GP1)

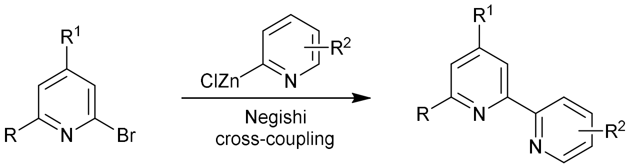

A variant of the protocol described by Hanan *et al.* was employed.[2] An oven dried flask was charged with substituted 2-bromopyridine (1.5-3.5 equiv.), THF (c = 0.5 M) and was cooled to −78 °C. The ^{n}BuLi (1.5-3.5 equiv., 1.6 M in hexanes) was added dropwise over a few minutes, and the mix was stirred for another 1 h at −78 °C. Then $ZnCl_2$ (1.5-3.5 equiv.) in THF (c = 0.5 M) was added dropwise at −78 °C. The mixture was allowed to warm to rt and stirred at rt for 2 h. A separate flask was charged with 6'-bromo-2,2'-bipyridine *or* substituted 2,6-dibromopyridine (1.0 equiv.), $Pd(PPh_3)_4$ (3.0-5.0 mol%) and THF (c ~ 0.5 M). The 2 mixtures were combined by slow addition of the *in-situ* formed organozinc species over 10 mins. Upon complete addition, the mixture was stirred at rt *OR* heated to reflux for 1-5 days. The mixture was allowed to cool to rt and was poured into the mixture of EDTA (approx. 8 g), aq. 10% NaOH (75 mL), water (75 mL) and trifluoroacetic acid (2.0 mL). The biphasic mixture was stirred vigorously until all solid dissolved. The aqueous phase was extracted with Et_2O (3×), and the combined organics were dried over $MgSO_4$ and concentrated *in vacuo*. The silica gel chromatography yielded the title compound.

General Procedure for Terpyridine Scaffold Assembly via Condensation (GP2)

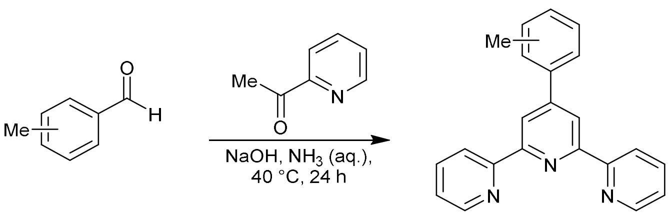

A variant of the protocol described by Peng *et al.* was employed.[3] The 2-acetylpyridine (1.21 g, 10.0 mmol, 2.0 equiv.), corresponding tolyladehyde (0.60 g, 5.0 mmol, 1.0 equiv.), sodium hydroxide (0.40 g, 10.0 mmol, 2.0 equiv.), aqueous ammonia (16.3 ml, 25-28 wt%) and ethanol (25 mL, 0.2 M) were added to the flask and heated to 40 °C for 24 h. The mixture was cooled to rt and the orange precipitate was collected by gravity filtration. To solid was dissolved in DCM and concentrated *in vacuo*. Purification of the crude mixture by silica gel chromatography yielded the title compound.

General Procedure for Nucleophilic Substitution towards Alkyl Bromides (GP3)

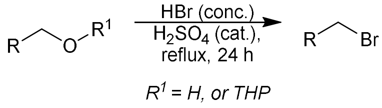

A variant of the protocol described by Phipps *et al.* was employed.[4] The (substituted) alcohol was dissolved in conc. HBr (c = 0.1 M) and conc. H_2SO_4 (0.4 mL/mmol of substrate). The mixture was refluxed for 24 h. Then, the mixture was diluted with water (20 mL) and basified to pH 8 with aq. sat. Na_2CO_3 solution. The aqueous phase was extracted with DCM (3×), and the combined organics were dried over $MgSO_4$ and concentrated *in vacuo*. The purification of the crude mixture by silica gel chromatography yielded the title compound.

General Procedure for the Preparation of Alkyl Sulfonates (GP4)

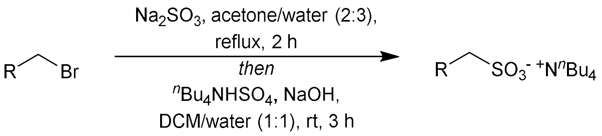

A variant of the protocol described by Phipps *et al.* was employed.[4] The alkyl bromide (1.0 equiv.) and Na_2SO_3 (1.5 equiv.) were dispersed in acetone/H_2O (2:3, v:v, c = 0.2 M) and heated to reflux for 2 h. The solvent was removed *in vacuo,* producing a white residue. The residue was fully redissolved in water (30 mL/mmol of substrate) and washed with DCM (3× volume of aqueous phase). The aqueous phase was diluted to double of the volume and washed with Et_2O (3× volume of aqueous phase). Then the aqueous layer was blown down and the resulting solid was redissolved in a biphasic mixture of $CHCl_3$/H_2O (1:1, v:v, c = 0.2 M). before the addition of tetrabutylammonium hydrogensulfate (0.8 equiv.) and NaOH (0.8 equiv.). The biphasic mixture was vigorously stirred for 3 h. The organic phase was reserved, and the aqueous phase was extracted with DCM (3× 30 mL/mmol of the substrate). The combined organics were washed with water (3× 40 mL/mmol of the substrate), dried over $MgSO_4$ and concentrated *in vacuo*.

Note: Bigger reaction scales (above 1.0 mmol) often require more aqueous washes of the final DCM solution.

General Procedure for Preparation of Terpyridine Palladium(II) Complexes (GP5)

A variant of the protocol described by Ritter *et al.* was employed.[5] The palladium acetate (1.1 equiv.) was fully dissolved in anhydrous MeCN (c = 0.08 M), and terpyridine (1.0 equiv.) was added at once, yielding a yellow suspension. The mixture was stirred for 30 mins at rt, which following the corresponding acid (2.2 equiv.) was added at once at rt. The mixture was stirred for a further 30 mins before the precipitate was collected by gravity filtration. The product was washed with ice-cold acetonitrile (approx. 2.0 mL). The crude product was either washed or sonicated with Et_2O (2× 5.0 mL).

General Protocol for the Suzuki-Miyaura Cross-coupling of 2-chloro-1,10-phenanthroline and Arylboronic Acids (GP6)

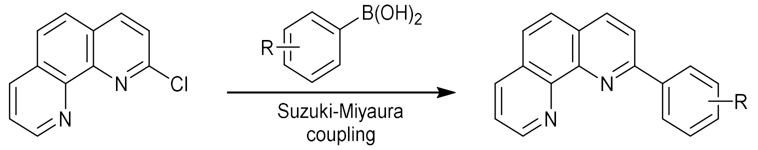

A variant of the protocol described by Stahl *et al.* was employed.[6] The flask was charged with 2-chloro-1,10-phenanthroline (1.0 equiv.), arylboronic acid (1.3 equiv.) and sodium carbonate (5.0 equiv.). The mixture was dispersed in 1,4-dioxane/water (4:1, v:v, c = 0.1 M) and sparged with nitrogen while stirring for 10 minutes. To this suspension was then added $Pd(PPh_3)_4$ (1.0 mol%), and the mixture was refluxed overnight. The mix was allowed to cool to rt, filtered through a plug of Celite and washed with EtOAc. The combined organics were dried over $MgSO_4$ and concentrated in vacuo. The crude product was purified by silica gel chromatography to yield the title compound.

General Procedure for Amines Protection with Acid Anhydrides (GP7)

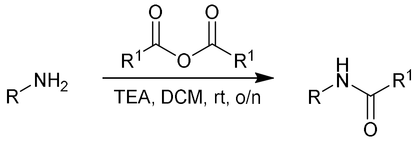

A variant of the protocol described by Phipps *et al.* was employed.[7] The amine (1.0-1.1 equiv.) was mixed with Et_3N (1.2 equiv.) and anhydrous DCM (c = 0.4 M) at rt. The acid anhydride (1.0-1.1 equiv.) was added dropwise at rt. The mix was stirred at rt overnight (16 h). The reaction was quenched with 1M HCl. The organic phase was separated, washed with 1M HCl (2×) and saturated aqueous $NaHCO_3$ (1×), dried over $MgSO_4$ and concentrated *in vacuo*. The title compound was obtained either by petrol washes, reprecipitation (DCM/petrol) or silica gel chromatography.

General Procedure for Pd-catalysed Fluorination of Arenes (GP8)

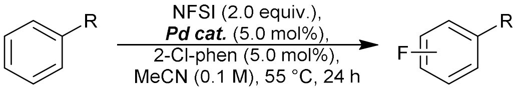

A variant of the protocol described by Ritter *et al.* was employed.[5] Under an inert atmosphere, the oven-dried 4.0 mL crimp-top vial was charged with the substrate (0.1 mmol, 1.0 equiv.), NFSI (63.1 mg, 0.2 mmol, 2.0 equiv.), 2-chloro-1,10-phenanthroline (1.1 mg, 0.005 mmol, 5 mol%) and either **Complex 1** $[Pd(tpy)(MeCN)](BF_4)_2$ (2.8 mg, 0.005 mmol, 5 mol%) *OR* **Complex 2** [Pd(s-tpy)(MeCN)](OTf) (3.1 mg, 0.005 mmol, 5 mol%) *OR* **Complex 4** $[Pd(s\text{-}tpy\text{-}^tBu)(MeCN)](BF_4)$ (3.4 mg, 0.005 mmol, 5 mol%). The capped vial was evacuated and backfilled with nitrogen (3×). Afterwards, acetonitrile (1.0 mL) was added at once and the mix was stirred at 55 °C for 24 h. The mixture was allowed to cool to rt. In the presence of solid particles, the crude mixture was filtered through a silica gel plug, washed with DCM (10 mL) and concentrated *in vacuo*. The internal standard was added to the crude mixture in MeCN-d^3, and NMR yield and reaction regioselectivity were determined. The products were purified by flash column chromatography or preparative TLC.

Note 1: The same procedure was applied to the reaction optimisation.

Note 2: The liquid starting materials were added as a stock solution in anhydrous MeCN.

Additional Optimisation Data

Table S1: Investigation of the Sulfonated Ligands and Directing Group

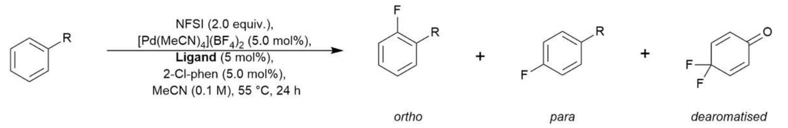

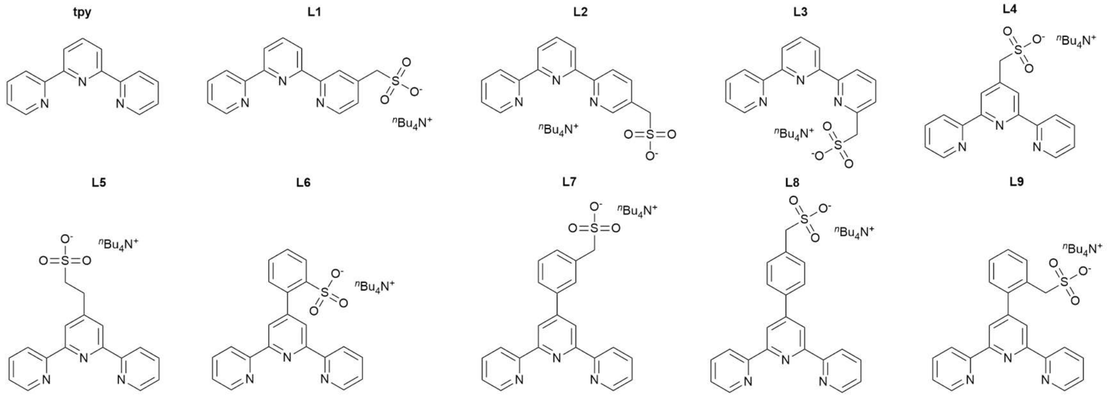

	Ph-OH				Ph-NHAc				Ph-TFA			
	Yield [%][1]	2a:3a:4a[1]	*o-:p-*[2]	*o-:p-*[3]	Yield [%][1]	2b:3b:4b[1]	*o-:p-*[2]	*o-:p-*[3]	Yield [%][1]	2c:3c:4c[1]	*o-:p-*[2]	*o-:p-*[3]
**tpy**	27	8:11:8	1:1.4	1:2.4	62	31:14:17	2.2:1	1:1.0	13	3:8:2	1:2.7	1:3.3
**L1**	*n.d.*	*n.d.*	*n.d.*	*n.d.*	*n.d.*	*n.d.*	*n.d.*	*n.d.*	39	6:27:6	1:4.5	1:5.5
**L2**	27	9:16:2	1:1.8	1:2.0	44	23:17:4	1.3:1	1.1:1	18	4:13:1	1:3.3	1:3.5
**L3**	3	1:2:0	1:2.0	1:2.0	3	1:2:0	1:2.0	1:2.0	6	2:4:0	1:2.0	1:2.0
**L4**	**42**	**5:17:20**	**1:3.4**	**1:7.4**	**59**	**20:27:12**	**1:1.4**	**1:2.0**	**30**	**4:25:1**	**1:6.3**	**1:6.5**
**L5**	*n.d.*	*n.d.*	*n.d.*	*n.d.*	*n.d.*	*n.d.*	*n.d.*	*n.d.*	37	25:12:2	2.1:1	1.8:1[3]
**L6**	21	7:11:3	1:1.6	1:2.0	42	18:8:16	2.3:1	1:1.3	16	7:8:1	1:1.1	1:1.3
**L7**	*n.d.*	*n.d.*	*n.d.*	*n.d.*	*n.d.*	*n.d.*	*n.d.*	*n.d.*	14	5:8:0	1:1.5	1:1.6
**L8**	10	4:5:1	1:1.3	1:1.5	44	18:17:9	1.1:1	1:1.4	11	6:4:1	1.5:1	1.2:1
**L9**	24	6:6:12	1:1.0	1:3.0	64	20:27:17	1:1.4	1:2.2	25	8:15:2	1:1.9	1:2.1

[1]The yield and *o-:p-*:dearomatised ratio were determined by ^{19}F NMR coupled with ^{1}H nuclei using 1,3-difluorobenzene or 4,4'-difluoroacetophenone as internal standard. [2]Quoted *o-:p-* ratio is **2**:**3** and does not include dearomatised product **4.** [3]The *o-:p-* ratio refers to the **2** vs (**3** + **4**). [3]The data were determined using $Pd(acac)_2$ (5 mol%).

Table S2: Solvent Screen

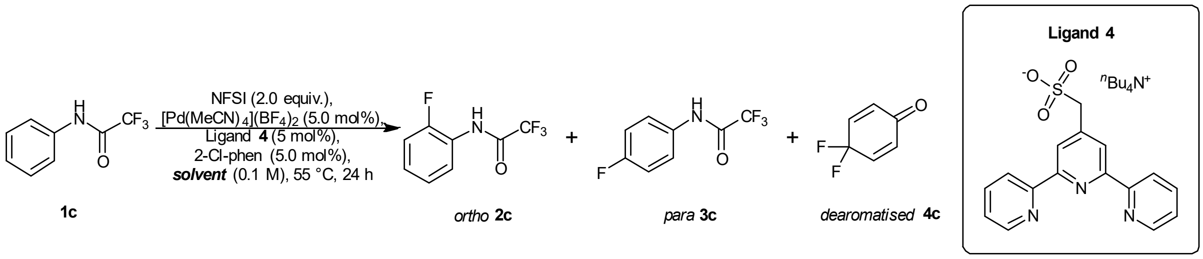

Entry	Solvent	Yield [%][1]	2c:3c:4c[1]	*o*-:*p*- ratio[2]
1	**MeCN**	**30**	**4:25:1**	**1:6.5**
2	DMF	2	*n.d.*	*n.d.*
3	DMA	<1	*n.d.*	*n.d.*
4	NMP	<1	*n.d.*	*n.d.*
5	DMSO	<1	*n.d.*	*n.d.*
6	EtOAc	14	7:7:0	1:1.0
7	1,4-dioxane	7	2:5:0	1:2.5
8	DCM	3	2:0:1	2.0:1
9	HFIP	3	1:1:1	1:2.0
10	$MeNO_2$	44	6:26:12	1:6.3
11	EtCN	14	4:9:1	1:2.5
12	butyrolactone	34	7:17:10	1:3.9

[1]The yield and *o*-:*p*-:dearomatised ratio were determined by ^{19}F NMR coupled with ^{1}H nuclei using 1,3-difluorobenzene or 4,4'-difluoroacetophenone as internal standard. [2]The *o*-:*p*- ratio refers to the ortho- isomer vs (para isomer + dearomatised).

Table S3: Investigation of the Palladium Source

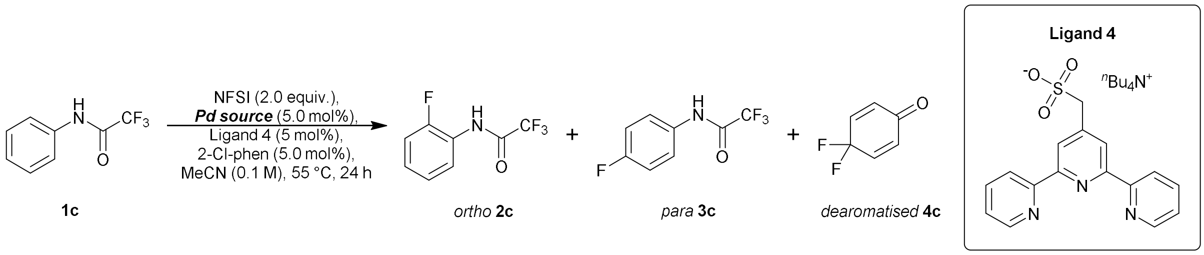

Entry	Pd Source	mmol %	Yield [%][1]	2c:3c:4c[1]	*o*-:*p*- ratio[2]
1	$Pd(MeCN)_4(BF_4)_2$	5	30	4:25:1	1:6.5
2	$Pd(PhCN)_2Cl_2$	5	<1	*n.d.*	*n.d.*
3	$Pd(OTFA)_2$	5	7	4:3:0	1.3:1
4	$Pd(OAc)_2$	5	39	6:30:3	1:5.5
5	$Pd(OPiv)_2$	5	46	5:36:5	1:8.2
6	**$Pd(acac)_2$**	**5**	**50**	**6:38:6**	**1:7.3**
7	$Pd(OAc)_2$	10	60	11:40:9	1:4.5
8	$Pd(OPiv)_2$	10	46	7:34:6	1:5.7
9	$Pd(acac)_2$	10	35	7:25:3	1:4.0
10	$Pd(cod)Cl_2$	5	40	6:32:2	1:5.7
11	$[Pd(allyl)Cl]_2$	5	25	7:17:1	1:2.6

[1]The yield and *o*-:*p*-:dearomatised ratio were determined by ^{19}F NMR coupled with ^{1}H nuclei using 1,3-difluorobenzene or 4,4'-difluoroacetophenone as internal standard. [2]The *o*-:*p*- ratio refers to the *ortho*- isomer vs (*para* isomer + dearomatised).

Table S4: Investigation of the Phenanthroline Co-ligand

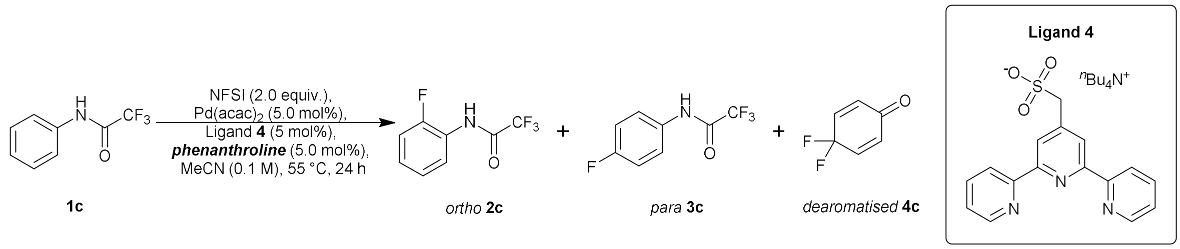

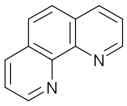

3% - 1:2:0 - 1:2.0

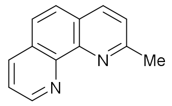

15% - 4:9:1 - 1:2.5

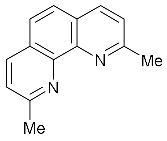

8% - 0.5:7.5:0 - 1:15.0

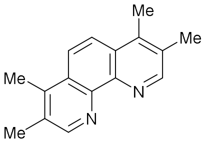

3% - 0.5:2.5:0 - 1:5.0

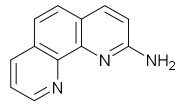

11% - 4:7:0 - 1:1.8

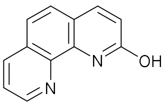

< 1% - n.d.

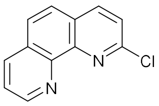

50% - 6:38:6 - 1:7.3

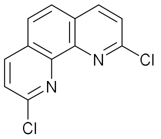

< 1% - n.d.

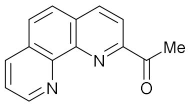

19% - 3:15:1 - 1:5.3

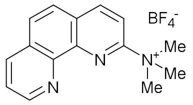

< 1% - n.d.

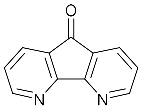

2% - 0.5:1.5:0 - 1:3.0

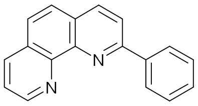

34% - 5:26:3 - 1:5.8

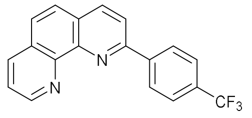

8% - 1:7:0 - 1:7.0

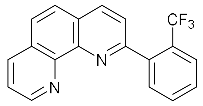

34% - 8:16:8 - 1:3.3

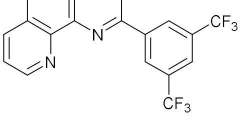

10% - 4:6:0 - 1:1.5

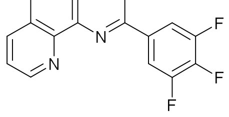

26% - 8:16:2 - 1:2.3

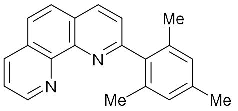

56% - 9:32:15 - 1:5.2

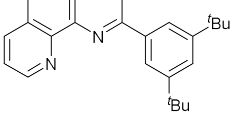

2% - 1:1:0 - 1:1.0

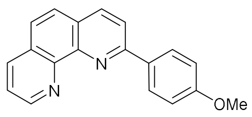

10% - 3:7:0 - 1:2.3

The data is presented as follows: NMR yield – *ortho*-:*para*-:dearomatised – *ortho* : (*para* + dearomatised). The yield and *o*-:*p*-dearomatised ratio were determined by ^{19}F NMR coupled with ^{1}H nuclei using 1,3-difluorobenzene or 4,4'-difluoroacetophenone as internal standard.

Table S5: Fluorinating Reagent Screen

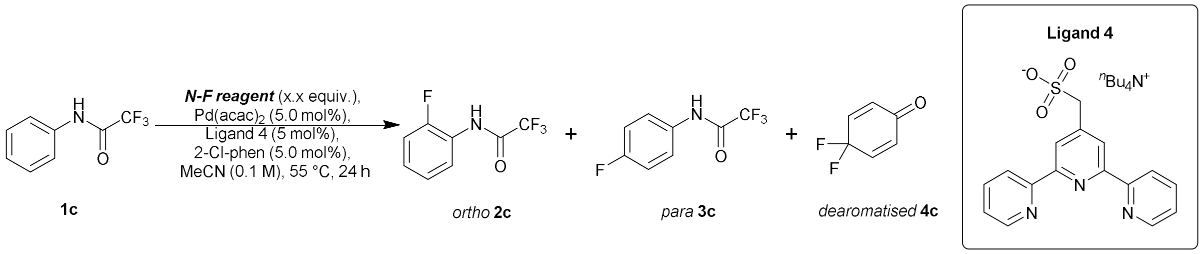

Entry	Reagent	Equiv.	Yield [%][1]	2c:3c:4c[1]	*o*-:*p*- ratio[2]
1	*N*-fluoropyridinium triflate	2.0	<1	*n.d.*	*n.d.*
2	Selectfluor	2.0	36	6:23:7	1:5.0
3	**NFSI**	**2.0**	**50**	**6:38:6**	**1:7.3**
4	NFSI	1.5	32	4:28:2	1:7.5
5	NFSI	3.0	48	7:32:9	1:5.9

[1]The yield and *o*-:*p*-:dearomatised ratio were determined by ^{19}F NMR coupled with ^{1}H nuclei using 1,3-difluorobenzene or 4,4'-difluoroacetophenone as internal standard. [2]The *o*-:*p*- ratio refers to the *ortho*- isomer vs (*para* isomer + dearomatised).

Table S6: Screening of Reaction Conditions

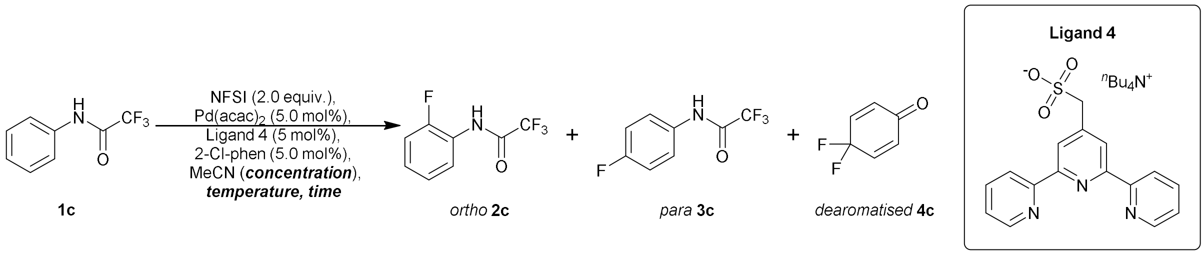

Entry	*T* [°C]	*t* [h]	*c* [M]	Yield [%][1]	2c:3c:4c[1]	*o*-:*p*- ratio[2]
1	40	24	0.10	21	3:18:0	1:6.0
2	**55**	**24**	**0.10**	**50**	**6:38:6**	**1:7.3**
3	55	48	0.10	38	7:30:1	1:4.4
4	70	16	0.10	19[3]	6:13:0	1:2.2
5	70	24	0.10	18[3]	6:12:0	1:2.0
6	55	24	0.05	11	5:6:0	1:1.1
7	55	24	0.20	20	6:13:1	1:2.3

[1]The yield and *o*-:*p*-:dearomatised ratio were determined by ^{19}F NMR coupled with ^{1}H nuclei using 1,3-difluorobenzene or 4,4'-difluoroacetophenone as internal standard. [2]The *o*-:*p*- ratio refers to the *ortho*- isomer vs (*para* isomer + dearomatised). [3]Catalyst decomposition was observed upon exposure to 70 °C within 1 h.

Synthesis of Sulfonated Terpyridine Ligands

6-bromo-2,2’-bipyridine

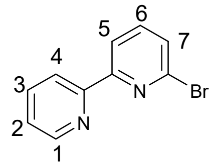

Prepared according to **GP1** using 2-bromopyridine (5.93 g, 37.5 mmol, 1.5 equiv.), ^{n}BuLi (23.4 mL, 37.5 mmol, 1.5 equiv., 1.6 M in hexanes), $ZnCl_2$ (5.11 g, 37.5 mmol, 1.5 equiv.), 2,6-dibromopyridine (5.92 g, 25.0 mmol, 1.0 equiv.) and $Pd(PPh_3)_4$ (1.16 g, 1.0 mmol, 4 mol%). The reaction was stirred at rt for 24 h. The silica gel chromatography (basified silica, Hex:EtOAc – 100:0 to 2:1) yielded the title compound as an off-white powder (2.54 g, 10.8 mmol, 43%).

Note: Around 25% of the product will react with second equivalent of organozinc to yield 2,2':6',2''-terpyridine.

^{1}H NMR (500 MHz, $CDCl_3$): δ (ppm) 8.67 (dd, J = 4.8, 1.6 Hz, 1H, H1), 8.41 (dd, J = 8.1, 1.2 Hz, 1H, H4), 8.39 (d, J = 7.8 Hz, 1H, H5), 7.82 (td, J = 7.8, 1.8 Hz, 1H, H3), 7.67 (app. t, J = 7.8 Hz, 1H, H6), 7.49 (d, J = 7.8 Hz, 1H, H7), 7.38-7.27 (m, 1H, H2).
^{13}C NMR (126 MHz, $CDCl_3$): δ (ppm) 157.4, 154.6, 149.3, 141.8, 139.4, 137.3, 128.2, 124.4, 121.7, 119.9

The NMR data are in agreement with that reported in the literature.[8]

4-methyl-2,2':6',2''-terpyridine

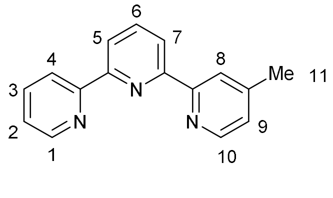

Prepared according to **GP1** using 2-bromo-4-methylpyridine (335 mg, 1.95 mmol, 1.5 equiv.), ^{n}BuLi (1.2 mL, 1.95 mmol, 1.5 equiv., 1.6 M in hexanes), $ZnCl_2$ (266 mg, 1.95 mmol, 1.5 equiv.), 6-bromo-2,2’-bipyridine (312 mg, 1.3 mmol, 1.0 equiv.) and $Pd(PPh_3)_4$ (75 mg, 0.065 mmol, 5 mol%). The reaction was refluxed for 3 days. Purification by silica gel chromatography (basified silica gel, PE:EtOAc – 15:1 to 3:1) afforded the title compound as a white powder (213 mg, 0.9 mmol, 66%).

^{1}**H NMR** (500 MHz, $CDCl_3$): δ (ppm) 8.70 (ddd, J = 4.8, 1.9, 0.9 Hz, 1H, H1), 8.62 (dt, J = 8.0, 1.1 Hz, 1H, H4), 8.56 (d, J = 4.9 Hz, 1H, H10), 8.45 (s, 1H, H8), 8.44 – 8.41 (m, 2H, H5, H7), 7.95 (app. t, J = 7.8 Hz, 1H, H6), 7.87 (td, J = 7.7, 1.8 Hz, 1H, H3), 7.33 (ddd, J = 7.5, 4.8, 1.2 Hz, 1H, H2), 7.16 (ddd, J = 5.1, 1.8, 0.9 Hz, 1H, H9), 2.49 (s, 3H, H11).

^{13}**C NMR** (126 MHz, $CDCl_3$): δ (ppm) 156.2, 155.9, 155.5, 155.3, 149.1, 148.9, 147.9, 137.8, 136.8, 124.7, 123.7, 121.9, 121.2, 121.1, 120.9, 21.3

The NMR data are in agreement with that reported in the literature.[9]

[2,2':6',2''-terpyridin]-4-ylmethanol

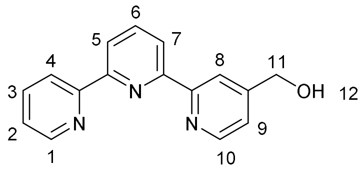

The 4-methyl-2,2':6',2''-terpyridine (124 mg, 0.5 mmol, 1.0 equiv.), selenium(IV) dioxide (139 mg, 1.25 mmol, 2.5 equiv.) and water (22.5 µL, 1.25 mmol, 2.5 equiv.) were refluxed for 24 h. After cooling to rt, the mixture was diluted with MeOH (1.2 mL) and aq. 2M NaOH (1.2 mL). The $NaBH_4$ (30.3 mg, 1.25 mmol, 2.5 equiv.) was added carefully at 0 °C. Afterwards, the mixture was stirred at rt for 30 mins. The precipitation was filtered, washed with MeOH and concentrated *in vacuo*, producing red residues. The residues were redissolved in $CHCl_3$ and filtered again to remove the selenium by-products. The crude yellow oil was purified by silica gel chromatography (basified silica gel, petrol:EtOAc – 7:3 to 1:3) afforded the title compound as a white powder (43 mg, 0.16 mmol, 33%).

^{1}**H NMR** (400 MHz, $CDCl_3$): δ (ppm) 8.64 (dd, J = 4.8, 1.5 Hz, 1H, H1), 8.56 (d, J = 5.0 Hz, 1H, H4), 8.49 (d, J = 7.9 Hz, 1H, H10), 8.46 (s, 1H, H8), 8.34 (d, J = 7.8 Hz, 2H, H5, H7), 7.86 (app. t, J = 7.8 Hz, 1H, H6), 7.78 (t, J = 7.6 Hz, 1H, H3), 7.31 – 7.21 (m, 2H, H2, H9), 4.78 (s, 2H, H11), 3.69 (s, 1H, H12).

^{13}**C NMR** (101 MHz, $CDCl_3$): δ (ppm) 156.3, 156.2, 155.3, 155.3, 151.3, 149.2, 149.1, 137.94, 137.1, 123.9, 121.5, 121.4, 121.2, 121.2, 118.7, 63.7

HRMS (ESI+): found $[M + H]^+$, m/z($C_{16}H_{14}N_3O^+$) calculated 264.1131, found 264.1132, δ = +0.2 ppm

4-(bromomethyl)-2,2':6',2''-terpyridine

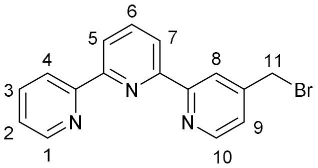

Prepared according to **GP3** using [2,2':6',2''-terpyridin]-4-ylmethanol (43 mg, 0.16 mmol, 1.0 equiv.) as the starting material. Purification by silica gel chromatography (basified silica gel, Hex:EtOAc – 9:1) afforded the title compound as a white powder (20 mg, 0.06 mmol, 38%, 98% purity).

Note: The product obtains 2% impurity; probably a product of the starting material oxidation (aldehyde).

^{1}H NMR (500 MHz, $CDCl_3$): δ (ppm) 8.71 (ddd, J = 4.8, 1.8, 0.9 Hz, 1H, H1), 8.68 (dd, J = 5.0, 0.8 Hz, 1H, H4), 8.63 – 8.59 (m, 2H, H8, H10), 8.46 (ddd, J = 7.9, 6.1, 1.1 Hz, 2H, H5, H7), 7.96 (app. t, J = 7.8 Hz, 1H, H6), 7.89 (td, J = 7.7, 1.8 Hz, 1H, H3), 7.39 – 7.31 (m, 2H, H2, H9), 4.54 (s, 2H, H11).

^{13}C NMR (126 MHz, $CDCl_3$): δ (ppm) 157.0, 156.2, 155.6, 154.9, 149.8, 149.3, 147.2, 138.1, 137.1, 124.0, 123.8, 121.5, 121.3, 121.3, 121.0, 31.1

HRMS (ESI+): found $[M + H]^+$, m/z($C_{16}H_{13}BrN_3^+$) calculated 326.0287, found 326.0287, $\delta = -0.1$ ppm

Tetrabutylammonium [2,2':6',2''-terpyridin]-4-ylmethanesulfonate (L1)

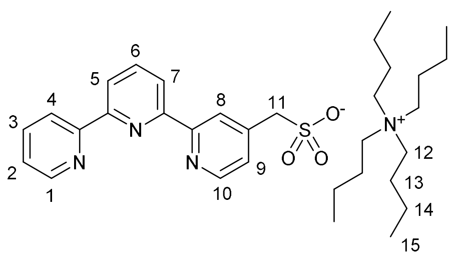

Prepared according to **GP4** using 4-(bromomethyl)-2,2':6',2''-terpyridine (20 mg, 0.06 mmol, 1.0 equiv.) as the starting material. Purification stated in **GP4** afforded the title compound as a white powder (20 mg, 0.04 mmol, 74%).

^{1}H NMR (500 MHz, $CDCl_3$): δ (ppm) 8.72 – 8.63 (m, 3H, H1, H4, H8), 8.58 (dd, J = 5.0, 0.8 Hz, 1H, H10), 8.42 (dd, J = 7.8, 1.4 Hz, 2H, H5, H7), 7.92 (app. t, J = 7.8 Hz, 1H, H6), 7.87 (td, J = 7.7, 1.8 Hz, 1H, H3), 7.52 (dd, J = 5.0, 1.7 Hz, 1H, H9), 7.32 (ddd, J = 7.5, 4.9, 1.1 Hz, 1H, H2), 4.22 (s, 2H, H11), 3.44 – 2.62 (m, 8H, H12), 1.53 – 1.33 (m, 8H, H13), 1.26 (h, J = 7.3 Hz, 8H, H14), 0.86 (t, J = 7.3 Hz, 12H, H15).

^{13}**C NMR** (126 MHz, $CDCl_3$): δ (ppm) 156.2, 155.6, 155.6, 155.5, 149.1, 148.7, 145.5, 137.9, 137.2, 126.3, 123.9, 123.4, 121.7, 121.0, 120.9, 58.6, 57.4, 24.0, 19.7, 13.7

HRMS (ESI−, anion): found $[M]^-$, m/z($C_{16}H_{12}N_3O_3S^-$) calculated 326.0599, found 326.0610, δ = +3.4 ppm

(6-bromopyridin-3-yl)methanol

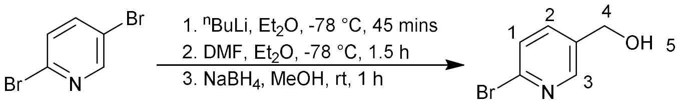

A variant of the procedure by Pritchard *et. al.* was employed.[10] To the suspension of 2,5-dibromopyridine (2.37 g, 10.0 mmol, 1.0 equiv.) in Et_2O (50.0 mL), the ^{n}BuLi (7.8 mL, 12.5 mmol, 1.6M in hexanes, 1.25 equiv.) was added dropwise over 15 mins at −78 °C. The mixture was stirred for a further 45 mins at −78 °C, which following the DMF (1.1 mL, 14.0 mmol, 1.4 equiv.) was added dropwise while keeping the temperature at −78 °C. The mixture was then stirred for 1.5 h at −78 °C before the reaction was quenched with water (5.0 mL) and sat. aq. NH_4Cl (5.0 mL). The aqueous phase was extracted with EtOAc (3×), and the combined organics were dried over $MgSO_4$ and concentrated *in vacuo*. The orange residue was redissolved in MeOH (25.0 mL), and $NaBH_4$ (114 mg, 3.0 mmol) was carefully added at 0 °C. The mixture was stirred for 1 h at rt, and following the 2M HCl (10.0 mL) was added. The solution was basified to pH 9 with sat. aq. Na_2CO_3 and the product was extracted with DCM (3×). Combined organics were dried over $MgSO_4$ and concentrated *in vacuo*. The compound was obtained as brown crystals (1.52 g, 8.1 mmol, 81%) and used in the following step without further purification.

^{1}**H NMR** (500 MHz, $CDCl_3$): δ (ppm) 8.34 (d, J = 2.5 Hz, 1H, H3), 7.59 (dd, J = 8.2, 2.5 Hz, 1H, H2), 7.48 (d, J = 8.2 Hz, 1H, H1), 4.71 (s, 2H, H4), 2.20 (br s, 1H, H5).

^{13}**C NMR** (126 MHz, $CDCl_3$): δ (ppm) 148.8, 141.3, 137.6, 135.6, 128.1, 62.0

The NMR data is in agreement with that reported in the literature.[10]

2-bromo-5-(((tetrahydro-2H-pyran-2-yl)oxy)methyl)pyridine

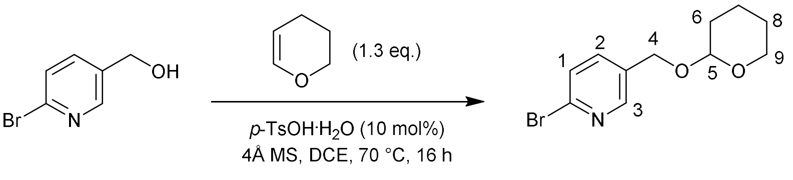

A variant of the procedure by Funeriu *et. al.* was employed.[11] The flask was charged with (6-bromopyridin-3-yl)methanol (2.26 g, 12 mmol, 1.0 equiv.), DHP (1.4 mL, 15.6 mmol, 1.3 equiv.), *p*-toluenesulfonic acid monohydrate (228 mg, 1.2 mmol, 0.1 equiv.), 4Å molecular sieves (200 mg) and DCE (14.0 mL). Then the mixture was heated to reflux for 16 h. The mixture was allowed to cool to rt and the solvent was removed *in vacuo*. Purification using silica gel chromatography (Hex:EtOAc – 7:3) afforded the product as a yellow oil (2.45 g, 9.0 mmol, 75%).

^{1}H NMR (500 MHz, $CDCl_3$): δ (ppm) 8.33 (dd, J = 2.4, 0.8 Hz, 1H, H3), 7.55 (dd, J = 8.2, 2.4 Hz, 1H, H2), 7.44 (dd, J = 8.2, 0.7 Hz, 1H, H1), 4.73 (d, J = 12.5 Hz, 1H, H4), 4.67 (t, J = 3.6 Hz, 1H, H5), 4.46 (d, J = 12.5 Hz, 1H, H4), 3.84 (ddd, J = 11.3, 8.5, 3.1 Hz, 1H, H9), 3.53 (dddd, J = 11.3, 5.1, 3.9, 1.3 Hz, 1H, H9), 1.86 – 1.77 (m, 1H, H6), 1.76 – 1.68 (m, 1H, H6), 1.66 – 1.48 (m, 4H, H7, H8).

^{13}C NMR (126 MHz, $CDCl_3$): δ (ppm) 149.6, 141.2, 138.2, 133.3, 127.9, 98.2, 65.7, 62.4, 30.5, 25.4, 19.3

The NMR data is in agreement with that reported in the literature.[11]

5-(((tetrahydro-2H-pyran-2-yl)oxy)methyl)-2,2':6',2''-terpyridine

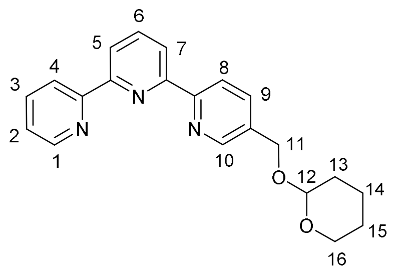

Prepared according to **GP1** using 2-bromo-5-(((tetrahydro-2H-pyran-2-yl)oxy)methyl)pyridine (2.38 g, 8.75 mmol, 1.75 equiv.), ^{n}BuLi (5.5 mL, 8.75 mmol, 1.75 equiv., 1.6 M in hexanes), $ZnCl_2$ (1.19 g, 8.75 mmol, 1.75 equiv.), 2-bromo-2,2'-bipyridine (1.18 g, 5.0 mmol, 1.0 equiv.) and $Pd(PPh_3)_4$ (288 mg, 0.25 mmol, 5 mol%). The mixture was heated to reflux for 5 days. The silica gel chromatography (basified silica, Hex:EtOAc - 20:1 to 4:1) yielded the title compound as a low melting colourless solid (1.41 g, 4.1 mmol, 81%).

^{1}H NMR (500 MHz, $CDCl_3$): δ (ppm) 8.72 – 8.65 (m, 2H, H1, H4), 8.64 – 8.53 (m, 2H, H8, H10), 8.44 (d, J = 7.8 Hz, 2H, H5, H7), 7.94 (app. t, J = 7.8 Hz, 1H, H6), 7.89 – 7.76 (m, 2H, H3, H9), 7.32 (ddd, J = 7.5, 4.8, 1.2 Hz, 1H, H2), 4.87 (d, J = 12.5 Hz, 1H, H11), 4.75 (t, J = 3.6 Hz, 1H, H12), 4.61 (d, J = 12.4 Hz, 1H, H11), 3.92 (ddd, J = 11.4, 8.4, 3.0 Hz, 1H, H13), 3.57 (ddd, J = 11.5, 4.9, 1.2 Hz, 1H, H13), 1.94 – 1.81 (m, 1H, H16), 1.81 – 1.73 (m, 1H, H16), 1.71 – 1.51 (m, 4H, H14, H15).

^{13}**C NMR** (126 MHz, $CDCl_3$): δ (ppm) 156.4, 155.7, 155.5, 155.3, 149.2, 148.8, 138.0, 136.9, 136.5, 134.0, 123.9, 121.3, 121.1, 121.0, 120.9, 98.1, 66.4, 62.4, 30.6, 25.5, 19.4

HRMS (ESI+): found $[M + Na]^+$, m/z($C_{21}H_{21}N_3O_2Na^+$) calculated 370.1526, found 370.1544, δ = +5.0 ppm

5-(bromomethyl)-2,2':6',2''-terpyridine

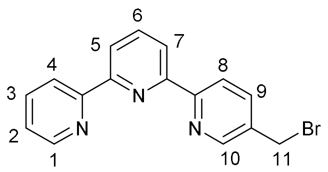

Prepared according to **GP3** using 5-(((tetrahydro-2H-pyran-2-yl)oxy)methyl)-2,2':6',2''-terpyridine (938 mg, 2.7 mmol, 1.0 equiv.) as the starting material. Purification by silica gel chromatography (basified silica gel, Hex:EtOAc – 9:1 to 5:1) afforded the title compound as a yellow powder (590 mg, 1.8 mmol, 67%).

^{1}**H NMR** (500 MHz, $CDCl_3$): δ (ppm) 8.74 – 8.66 (m, 2H, H1, H4), 8.63 – 8.56 (m, 2H, H8, H10), 8.45 (dd, J = 9.0, 7.8 Hz, 2H, H5, H7), 7.95 (app. t, J = 7.8 Hz, 1H, H6), 7.90 – 7.82 (m, 2H, H3, H9), 7.33 (ddd, J = 7.5, 4.8, 1.2 Hz, 1H, H2), 4.55 (s, 2H, H11).

^{13}**C NMR** (126 MHz, $CDCl_3$): δ (ppm) 156.3, 156.2, 155.6, 154.9, 149.4, 149.3, 138.1, 137.6, 137.0, 133.8, 123.9, 121.4, 121.3 (2C), 121.2, 29.9

The NMR data are in agreement with that reported in the literature.[12]

Tetrabutylammonium [2,2':6',2''-terpyridin]-5-ylmethanesulfonate (L2)

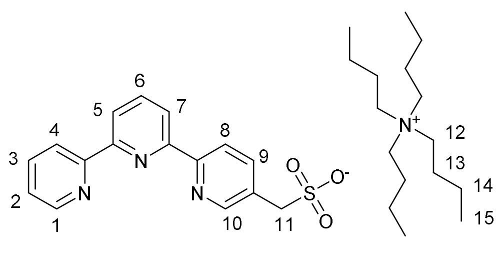

Prepared according to **GP4** using 5-(bromomethyl)-2,2':6',2''-terpyridine (326 mg, 1.0 mmol, 1.0 equiv.) as the starting material. Purification stated in **GP4** afforded the title compound as a white powder (340 mg, 0.60 mmol, 75%).

^{1}**H NMR** (400 MHz, $CDCl_3$): δ (ppm) 8.62 (dd, J = 2.2, 0.8 Hz, 1H, H4), 8.60 (ddd, J = 4.8, 1.9, 0.9 Hz, 1H, H1), 8.49 (dt, J = 7.9, 1.1 Hz, 1H, H4), 8.42 (dd, J = 8.1, 0.8 Hz, 1H, H8), 8.36 – 8.27 (m, 2H,

H5, H7), 7.91 (dd, J = 8.1, 2.2 Hz, 1H, H9), 7.83 (t, J = 7.8 Hz, 1H, H3), 7.77 (app. td, J = 7.7, 1.8 Hz, 1H, H6), 7.25 (ddd, J = 7.5, 4.8, 1.1 Hz, 1H, H2), 4.01 (s, 2H, H11), 3.24 – 2.60 (m, 8H, H12), 1.51 – 1.30 (m, 8H, H13), 1.22 (q, J = 7.3 Hz, 8H, H14), 0.81 (t, J = 7.2 Hz, 12H, H15).

^{13}C NMR (101 MHz, $CDCl_3$): δ (ppm) 156.0, 155.4, 155.2, 154.0, 150.8, 149.0, 138.7, 137.7, 136.8, 131.6, 123.7, 120.9, 120.6, 120.5, 120.3, 58.1, 54.8, 23.7, 19.5, 13.5.

HRMS (ESI−, anion): found $[M]^-$, m/z($C_{16}H_{12}N_3O_3S^-$) calculated 326.0599, found 326.0604, δ = +1.2 ppm

6-methyl-2,2':6',2''-terpyridine

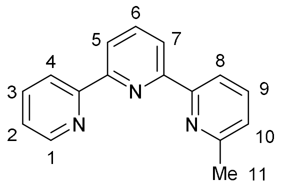

Prepared according to **GP1** using 2-bromo-4-methylpyridine (1.29 g, 7.5 mmol, 1.5 equiv.) ^{n}BuLi (4.7 mL, 7.5 mmol, 1.5 equiv., 1.6 M in hexanes), $ZnCl_2$ (1.02 g, 7.5 mmol, 1.5 equiv.), 6-bromo-2,2'-bipyridine (1.175 g, 5.0 mmol, 1.0 equiv.) and $Pd(PPh_3)_4$ (289 mg, 0.25 mmol, 5 mol%). The reaction was refluxed for 3 days. Purification by silica gel chromatography (basified silica gel, PE:EtOAc – 15:1 to 7:1) afforded the title compound as a white powder (900 mg, 3.6 mmol, 73%).

^{1}H NMR (500 MHz, $CDCl_3$): δ (ppm) 8.70 (ddd, J = 4.8, 1.8, 0.9 Hz, 1H, H1), 8.63 (dt, J = 8.0, 1.1 Hz, 1H, H4), 8.48 (dd, J = 7.8, 1.1 Hz, 1H, H8), 8.43 (dd, J = 7.8, 1.1 Hz, 1H, H5), 8.41 (d, J = 7.7 Hz, 1H, H7), 7.95 (app. t, J = 7.8 Hz, 1H, H6), 7.85 (d, J = 7.8, 1.8 Hz, 1H, H3), 7.74 (t, J = 7.7 Hz, 1H, H9), 7.33 (ddd, J = 7.5, 4.8, 1.2 Hz, 1H, H2), 7.19 (dd, J = 7.6, 0.5 Hz, 1H, H10), 2.66 (s, 3H, H11).

^{13}C NMR (126 MHz, $CDCl_3$): δ (ppm) 158.0, 156.4, 155.7, 155.3, 149.2, 138.0, 137.3, 137.1, 123.9, 123.5, 121.4, 121.3, 121.0, 118.4, 24.8

The NMR data is in agreement with that reported in the literature.[13]

[2,2':6',2''-terpyridin]-6-ylmethanol

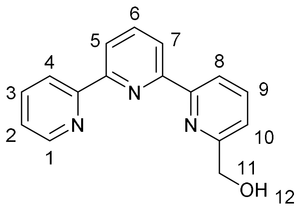

The 6-methyl-2,2':6',2"-terpyridine (594 mg, 2.4 mmol, 1.0 equiv.), selenium(IV) dioxide (667 mg, 6.0 mmol, 2.5 equiv.) and water (108 μL, 6.0 mmol, 2.5 equiv.) were refluxed for 48 h. After cooling to rt, the mixture was diluted with MeOH (3.0 mL) and aq. 2M NaOH (3.0 mL). The $NaBH_4$ (226 mg, 6.0 mmol, 2.5 equiv.) was added carefully at 0 °C. Afterwards, the mixture was stirred at rt for 30 mins. The precipitation was filtered, washed with MeOH and concentrated *in vacuo*, producing red residues. The residues were redissolved in $CHCl_3$ and filtered again to remove the selenium by-products. The crude yellow oil was purified by silica gel chromatography (basified silica gel, petrol:EtOAc – 7:3 to 4:6) afforded the title compound as white crystals (379 mg, 1.44 mmol, 60%, brsm 92%).

^{1}H NMR (500 MHz, $CDCl_3$): δ (ppm) 8.70 (ddd, J = 4.8, 1.9, 1.0 Hz, 1H, H1), 8.60 (dt, J = 7.9, 1.1 Hz, 1H, H4), 8.52 (d, J = 7.8 Hz, 1H, H8), 8.45 (d, J = 7.8 Hz, 2H, H5, H7), 7.94 (app. t, J = 7.8 Hz, 1H, H6), 7.89 – 7.76 (m, 2H, H3, H9), 7.32 (ddd, J = 7.5, 4.8, 1.2 Hz, 1H, H2), 7.26 (dd, J = 7.7, 0.9 Hz, 1H, H10), 4.84 (d, J = 4.0 Hz, 2H, H11), 4.12 (br t, J = 4.9 Hz, 1H, H12).

^{13}C NMR (126 MHz, $CDCl_3$): δ (ppm) 158.3, 156.3, 155.6, 155.1, 154.9, 149.3, 138.0, 137.7, 137.0, 123.9, 121.3, 121.3, 121.0, 120.6, 119.9, 64.1

The NMR data is in agreement with that reported in the literature.[13]

6-(bromomethyl)-2,2':6',2"-terpyridine

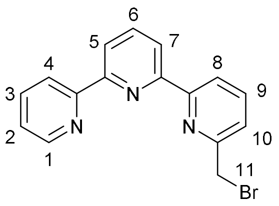

Prepared according to **GP3** using [2,2':6',2"-terpyridin]-6-ylmethanol (250 mg, 0.95 mmol, 1.0 equiv.) as the starting material. Purification by silica gel chromatography (basified silica gel, Hex:EtOAc – 9:1) afforded the title compound as a white powder (220 mg, 0.67 mmol, 71%).

^{1}H NMR (500 MHz, $CDCl_3$): δ (ppm) 8.70 (ddd, J = 4.8, 1.8, 0.9 Hz, 1H, H1), 8.60 (dt, J = 7.9, 1.1 Hz, 1H, H4), 8.53 (d, J = 7.9 Hz, 1H, H8), 8.51 (dd, J = 7.8, 1.1 Hz, 1H, H5), 8.46 (dd, J = 7.8, 1.1 Hz, 1H, H7), 7.95 (app. t, J = 7.8 Hz, 1H, H6), 7.85 (td, J = 7.7, 2.0 Hz, 2H, H3, H9), 7.48 (d, J = 7.6 Hz, 1H, H10), 7.33 (ddd, J = 7.5, 4.8, 1.2 Hz, 1H, H2), 4.65 (s, 2H, H11).

^{13}C NMR (126 MHz, $CDCl_3$): δ (ppm) 156.3, 156.3, 156.1, 155.5, 155.0, 149.3, 138.0, 137.9, 137.0, 123.9, 123.5, 121.4, 121.3, 121.3, 120.4, 34.3

The NMR data is in agreement with that reported in the literature.[13]

Tetrabutylammonium [2,2':6',2''-terpyridin]-6-ylmethanesulfonate (L3)

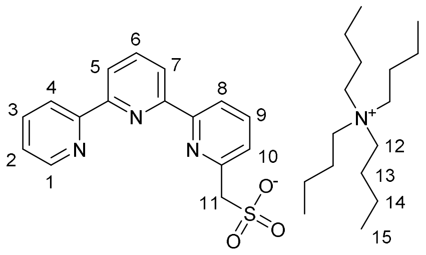

Prepared according to **GP4** using 4-(bromomethyl)-2,2':6',2"-terpyridine (147 mg, 0.45 mmol, 1.0 equiv.) as the starting material. Purification stated in **GP4** afforded the title compound as a white powder (150 mg, 0.26 mmol, 73%).

^{1}H NMR (500 MHz, $CDCl_3$): δ (ppm) 8.63 (ddd, J = 4.9, 1.8, 0.9 Hz, 1H, H1), 8.53 (dt, J = 8.0, 1.1 Hz, 1H, H4), 8.41 (dd, J = 7.9, 1.1 Hz, 1H, H8), 8.36 (dd, J = 7.7, 1.2 Hz, 1H, H5), 8.30 (dd, J = 7.8, 1.0 Hz, 1H, H7), 7.84 (app. td, J = 7.7, 1.8 Hz, 1H, H6), 7.79 (t, J = 7.8 Hz, 1H, H3), 7.76 – 7.73 (m, 1H, H9), 7.68 (t, J = 7.7 Hz, 1H, H10), 7.28 (ddd, J = 7.5, 4.8, 1.2 Hz, 1H, H2), 4.35 (s, 2H, H11), 3.44 – 2.67 (m, 8H, H12), 1.67 – 1.38 (m, 8H, H13), 1.28 (h, J = 7.4 Hz, 8H, H14), 0.87 (t, J = 7.3 Hz, 12H, H15).

^{13}C NMR (126 MHz, $CDCl_3$): δ (ppm) 156.0, 155.5, 155.0, 154.8, 154.7, 148.9, 137.6, 137.1, 136.8, 125.0, 123.8, 121.4, 121.2, 120.7, 118.9, 60.1, 58.5, 23.9, 19.6, 13.7

HRMS (ESI+, anion): found $[M]^{-}$, m/z($C_{16}H_{14}N_3O_3S^{-}$) calculated 328.0756, found 328.0762, δ = +1.8 ppm

2,6-dibromo-4-(((tetrahydro-2H-pyran-2-yl)oxy)methyl)pyridine

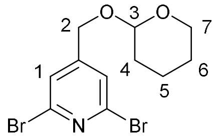

For the first step, a variant of the protocol by Chen was adapted.[14] The 2,6-dibromoisonicotinic acid (5.00 g, 17.8 mmol, 1.0 equiv.) was dissolved in anhydrous THF (89 mL, 0.2 M), and BH_3·THF (44.5 mL, 44.5 mmol, 1M solution, 2.5 equiv.) was added slowly at 0 °C. The mix was then heated to 50 °C for 24 h. To the cooled mixture, water was slowly added until gas evolution ceased, followed by concentration *in vacuo* to half of its original volume. To the concentrated mixture, aq. NaOH (50 mL, 10 wt%) was added, and the mix was refluxed for 1 h. The remaining THF was removed *in vacuo,* and the residual aqueous solution was extracted with DCM. Combined organic layers were dried over

$MgSO_4$ and concentrated *in vacuo*, yielding the crude product as brown powder (4.37 g, 16.4 mmol, 92%).

For the second step, a variant of a protocol by Chen *et. al.* was adapted.[14] The crude mixture, PPTS (1.03 g, 4.1 mmol, 25 mol%) and DHP (15.0 mL, 16.4 mmol, 1.0 equiv.) were dissolved in anhydrous DCM (41 mL, 0.4M) and stirred at rt for 20 h. The mix was diluted with DCM, washed with sat. aq. $NaHCO_3$ solution (1×) and water (1×). The organic phase was dried over $MgSO_4$ and concentrated *in vacuo*. Purification by silica gel chromatography (Hex:EtOAc – 9:1) yielded the compound as white crystals (5.08 g, 14.5 mmol, 88%).

^{1}H NMR (500 MHz, $CDCl_3$): δ (ppm) 7.43 (dd, J = 2.3, 1.1 Hz, 2H, H1), 4.73 (ddd, J = 14.5, 2.3, 1.1 Hz, 1H, H2), 4.70 – 4.67 (m, 1H, H3), 4.44 (ddd, J = 14.3, 1.9, 0.9 Hz, 1H, H2), 3.81 (ddd, J = 11.6, 8.8, 3.1 Hz, 1H, H7), 3.60 – 3.46 (m, 1H, H7), 1.93 – 1.81 (m, 1H, H5), 1.81 – 1.73 (m, 1H, H4), 1.73 – 1.65 (m, 1H, H4), 1.65 – 1.47 (m, 3H, H5, H6).

^{13}C NMR (126 MHz, $CDCl_3$): δ (ppm) 153.4, 153.4, 140.9, 140.9, 140.9, 125.0, 98.6, 65.9, 62.4, 30.39, 25.6, 25.4, 19.3

The NMR data is in agreement with that reported in the literature.[14]

Note, the compound undergoes a slow decomposition if stored at rt and under air for a few weeks.

4'-(((tetrahydro-2H-pyran-2-yl)oxy)methyl)-2,2':6',2''-terpyridine

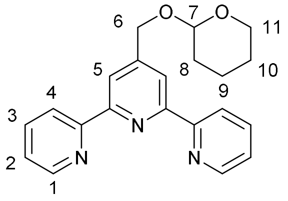

Prepared according to **GP1** using 2-bromopyridine (7.74 g, 49.0 mmol, 1.75 equiv.), ^{n}BuLi (30.6 mL, 49.0 mmol, 1.75 equiv., 1.6 M in hexanes), $ZnCl_2$ (6.68 g, 49.0 mmol, 1.75 equiv.), 2,6-dibromo-4-(((tetrahydro-2H-pyran-2-yl)oxy)methyl)pyridine (4.91 g, 14.0 mmol, 1.0 equiv.) and $Pd(PPh_3)_4$ (809 mg, 0.70 mmol, 5 mol%). The reaction was refluxed for 2 days. Purification by silica gel chromatography (basified silica gel, PE:EtOAc – 15:1 to 1:1) afforded the title compound as a yellow oil (2.83 g, 8.1 mmol, 58%).

^{1}H NMR (500 MHz, $CDCl_3$): δ (ppm) 8.70 (ddd, J = 4.5, 1.6, 1.4 Hz, 2H, H1), 8.61 (d, J = 7.9, 1.3 Hz, 2H, H4), 8.45 (s, 2H, H5), 7.84 (app. tt, J = 7.7, 1.7 Hz, 2H, H3), 7.32 (ddd, J = 7.6, 4.8, 1.4 Hz, 2H, H2), 4.95 (d, J = 13.8 Hz, 1H, H6), 4.80 (t, J = 3.3 Hz, 1H, H7), 4.68 (d, J = 13.5 Hz, 1H, H6), 4.08 –

3.83 (m, 1H, H11), 3.63 – 3.50 (m, 1H, H11), 2.00 – 1.85 (m, 1H, H9), 1.87 – 1.73 (m, 2H, H8), 1.69 – 1.49 (m, 3H, H9, H10).

^{13}C NMR (126 MHz, $CDCl_3$): δ (ppm) 156.4, 155.7, 149.8, 149.2, 136.9, 123.9, 121.5, 119.6, 98.5, 67.9, 62.2, 30.6, 25.6, 19.3

HRMS (ESI+): found $[M + H]^+$, m/z($C_{21}H_{22}N_3O_2^+$) calculated 348.1707, found 348.1704, $\delta = -0.9$ ppm

4'-(bromomethyl)-2,2':6',2''-terpyridine

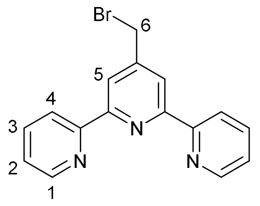

Prepared according to **GP3** using 4'-(((tetrahydro-2H-pyran-2-yl)oxy)methyl)-2,2':6',2''-terpyridine (2.43 g, 7.0 mmol, 1.0 equiv.) as the starting material. Purification by silica gel chromatography (basified silica gel, Hex:EtOAc – 9:1 to 5:1) afforded the title compound as a white powder (1.63 g, 5.0 mmol, 71%).

^{1}H NMR (500 MHz, $CDCl_3$): δ (ppm) 8.70 (ddd, J = 4.8, 1.8, 0.9 Hz, 2H, H1), 8.59 (app. dt, J = 8.0, 1.1 Hz, 2H, H4), 8.48 (s, 2H, H5), 7.84 (app. td, J = 7.7, 1.8 Hz, 2H, H3), 7.33 (ddd, J = 7.5, 4.8, 1.2 Hz, 2H, H2), 4.56 (s, 2H, H6).

^{13}C NMR (126 MHz, $CDCl_3$): δ (ppm) 156.2, 155.8, 149.3, 148.3, 137.0, 124.1, 121.4, 121.0, 31.1.

The NMR data is in agreement with that reported in the literature.[15]

Tetrabutylammonium [2,2':6',2''-terpyridin]-4'-ylmethanesulfonate (L4)

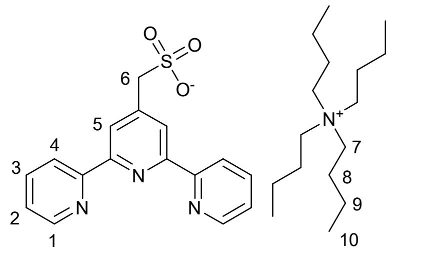

Prepared according to **GP4** using 4'-(bromomethyl)-2,2':6',2"-terpyridine (1.47 g, 4.5 mmol, 1.0 equiv.) as the starting material. Purification stated in **GP4** afforded the title compound as a white powder (1.64 g, 2.9 mmol, 80%).

^{1}H NMR (500 MHz, $CDCl_3$): δ (ppm) 8.61 (ddd, J = 4.7, 1.8, 0.9 Hz, 2H, H1), 8.52 (s, 2H, H5), 8.51 (dt, J = 8.0, 1.1 Hz, 2H, H4), 7.79 (app. td, J = 7.7, 1.8 Hz, 2H, H3), 7.26 (ddd, J = 7.3, 4.8, 1.2 Hz, 2H, H2), 4.19 (s, 2H, H6), 3.23 – 2.69 (m, 8H, H7), 1.44 – 1.24 (m, 8H, H8), 1.18 (h, J = 7.4 Hz, 8H, H9), 0.80 (t, J = 7.3 Hz, 12H, H10).

^{13}C NMR (126 MHz, $CDCl_3$): δ (ppm) 156.5, 155.0, 149.1, 146.6, 136.7, 123.6, 123.5, 121.2, 58.1, 57.5, 23.8, 19.6, 13.7

HRMS (ESI−, anion): found $[M]^-$, m/z($C_{16}H_{12}N_3O_3S^-$) calculated 326.0612, found 326.0612, δ = +4.0 ppm

4'-methyl-2,2':6',2"-terpyridine

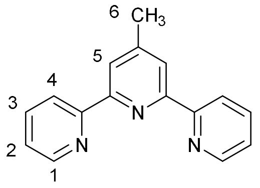

Prepared according to **GP1** using 2-bromopyridine (4.74 g, 30.0 mmol, 1.5 equiv.), ^{n}BuLi (18.8 mL, 30.0 mmol, 1.5 equiv., 1.6 M in hexanes), $ZnCl_2$ (4.09 g, 30.0 mmol, 1.5 equiv.), 2,6-dibromo-4-methylpyridine (2.50 g, 10.0 mmol, 1.0 equiv.), and $Pd(PPh_3)_4$ (578 mg, 0.5 mmol, 5 mol%). The reaction was refluxed for 24 h. Purification by silica gel chromatography (basified silica gel, PE:EtOAc – 100:0 to 3:1) afforded the title compound as a yellow powder (1.08 g, 4.4 mmol, 44%).

^{1}H NMR (400 MHz, $CDCl_3$): δ (ppm) 8.54 (ddd, J = 4.8, 1.9, 0.9 Hz, 2H, H1), 8.45 (dt, J = 8.0, 1.1 Hz, 2H, H4), 8.15 (s, 2H, H5), 7.63 (td, J = 7.7, 1.8 Hz, 2H, H3), 7.11 (ddd, J = 7.5, 4.8, 1.3 Hz, 2H, H2), 2.34 (s, 3H, H6).

^{13}C NMR (101 MHz, $CDCl_3$): δ (ppm) 156.5, 155.4, 149.2, 137.0, 123.8, 122.0, 121.5, 121.5, 21.5

The NMR data is in agreement with that reported in the literature.[9]

4'-(2-methoxyethyl)-2,2':6',2''-terpyridine

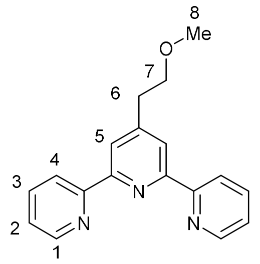

A variant of the protocol by Phipps *et. al.* adapted [4] Diisopropylamine (0.17 mL, 1.2 mmol, 1.2 equiv.) was dissolved in THF (2.0 mL) and cooled to −78 °C before the dropwise addition of ^{n}BuLi (0.71 mL, 1.2 mmol, 1.2 equiv.). The mix was stirred at −78 °C for 10 mins before the addition of 4'-methyl-2,2':6',2''-terpyridine (247 mg, 1.0 mmol, 1.0 equiv.) in THF (6.7 mL). The mix was kept at −78 °C and stirred for 2 h. Then, a solution of MOMCl (0.67 mL, 1.4 mmol, 1.4 equiv., 2M in PhMe) was added dropwise and the mixture was warmed to rt and stirred for 18 h. The reaction was quenched by the addition of sat. aq. $NaHCO_3$ solution. The crude mix was extracted with EtOAc (3×), and the combined organics were dried over $MgSO_4$ and concentrated *in vacuo*. Purification by silica gel chromatography (basified silica, Hex:EtOAc – 12:1 to 1:1) yielded the product as yellow crystals (109 mg, 0.37 mmol, 37%, brsm 97%).

^{1}H NMR (400 MHz, $CDCl_3$): δ (ppm) 8.70 (ddd, J = 4.8, 1.8, 0.9 Hz, 2H, H1), 8.61 (app. dt, J = 7.9, 1.1 Hz, 2H, H4), 8.33 (s, 2H, H5), 7.84 (app. td, J = 7.7, 1.8 Hz, 2H, H3), 7.32 (ddd, J = 7.5, 4.8, 1.2 Hz, 2H, H2), 3.76 (t, J = 7.0 Hz, 2H, H7), 3.38 (s, 3H, H8), 3.08 (t, J = 7.1 Hz, 2H, H6).

^{13}C NMR (101 MHz, $CDCl_3$): δ (ppm) 156.4, 155.5, 150.2, 149.2, 137.0, 123.8, 121.6, 121.5, 72.5, 58.9, 36.1

The NMR data is in agreement with that reported in the literature.[9]

4'-(2-bromoethyl)-2,2':6',2''-terpyridine

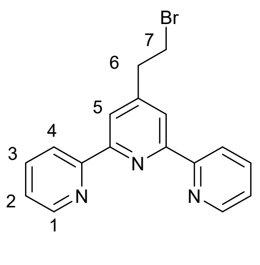

Prepared according to **GP3** using 4'-(2-methoxyethyl)-2,2':6',2''-terpyridine (73 mg, 0.25 mmol, 1.0 equiv.) as the starting material. Purification by silica gel chromatography (basified silica gel, Hex:EtOAc – 9:1 to 5:1) afforded the title compound as an off-white powder (40 mg, 0.12 mmol, 47%).

^{1}H NMR (500 MHz, $CDCl_3$): δ (ppm) 8.70 (ddd, J = 4.8, 1.8, 0.9 Hz, 2H, H1), 8.62 (dt, J = 8.0, 1.1 Hz, 2H, H4), 8.34 (s, 2H, H5), 7.86 (app. td, J = 7.7, 1.8 Hz, 2H, H3), 7.33 (ddd, J = 7.5, 4.8, 1.2 Hz, 2H, H2), 3.72 (t, J = 7.4 Hz, 2H, H7), 3.35 (t, J = 7.4 Hz, 2H, H6).

^{13}C NMR (126 MHz, $CDCl_3$): δ (ppm) 156.1, 155.8, 149.8, 149.2, 137.1, 124.0, 121.5, 121.2, 39.0, 31.4

HRMS (ESI+): found $[M + H]^+$, m/z($C_{17}H_{14}BrN_3^+$) calculated 340.0444, found 340.0447, δ = +0.9 ppm

Tetrabutylammonium 2-([2,2':6',2''-terpyridin]-4'-yl)ethane-1-sulfonate (L5)

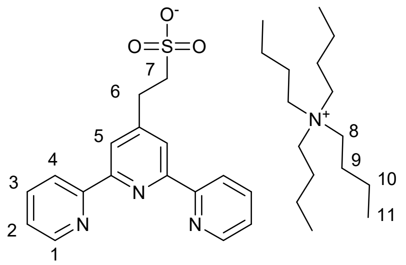

Prepared according to **GP4** using 4'-(2-bromoethyl)-2,2':6',2''-terpyridine (35 mg, 0.10 mmol, 1.0 equiv.) as the starting material. Purification stated in **GP4** afforded the title compound as a white powder (24 mg, 0.04 mmol, 52%).

^{1}H NMR (400 MHz, $CDCl_3$): δ (ppm) 8.66 (ddd, J = 4.8, 1.8, 0.9 Hz, 2H, H1), 8.56 (app. dt, J = 8.0, 1.1 Hz, 2H, H4), 8.35 (s, 2H, H5), 7.81 (app. td, J = 7.7, 1.8 Hz, 2H, H3), 7.29 (ddd, J = 7.5, 4.8, 1.2 Hz, 2H, H2), 3.43 – 3.33 (m, 2H, H7), 3.33 – 3.24 (m, 8H, H8), 3.24 – 3.18 (m, 2H, H6), 1.76 – 1.54 (m, 8H, H9), 1.42 (h, J = 7.3 Hz, 8H, H10), 0.98 (t, J = 7.3 Hz, 12H, H11).

^{13}C NMR (101 MHz, $CDCl_3$): δ (ppm) 156.5, 155.5, 152.7, 149.2, 136.8, 123.7, 121.4, 121.4, 58.9, 52.2, 31.9, 24.2, 19.9, 13.8

HRMS (ESI−, anion): found $[M]^-$, m/z($C_{17}H_{14}N_3O_3S^-$) calculated 340.0757, found 340.0752, δ = +0.3 ppm

Sodium 2-([2,2':6',2''-terpyridin]-4'-yl)benzenesulfonate

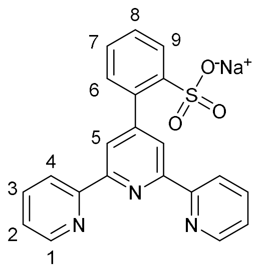

A variant of the protocol by Xue *et. al.* was adapted.[17] Sodium 2-formylbenzenesulfonate (2.08 g, 10.0 mmol, 1.0 equiv.) and 2-acetylpyridine (2.24 mL, 20.0 mmol, 1.0 equiv.) were dissolved in EtOH (33 mL) and ammonia (2.0 mL, 35% aq. solution). Then, NaOH (0.80 g, 20.0 mmol, 1.0 equiv.) was added at once. The mix was stirred at 0 °C for 3 h, followed by stirring at rt for 6 h and finally heating at 50 °C for 18 h. The mixture was allowed to cool to rt and concentrated in vacuo. The residue was dispersed into EtOH (15 mL) and filtered. Washing with MeOH (2× 7.5 mL) yielded the compound as a yellow powder (1.69 g, 4.1 mmol, 41%).

^{1}H NMR (400 MHz, DMSO-*d*^{6}): δ (ppm) 8.70 (ddd, J = 4.7, 1.8, 0.9 Hz, 2H, H1), 8.66 (app. dt, J = 8.1, 1.1 Hz, 2H, H4), 8.54 (s, 2H, H5), 8.07 – 7.94 (m, 3H, H3, H6), 7.52 – 7.41 (m, 4H, H2, H7, H9), 7.31 – 7.22 (m, 1H, H8).

^{13}C NMR (101 MHz, DMSO-*d*^{6}): δ (ppm) 155.7, 153.6, 152.3, 149.2, 145.9, 137.3, 137.1, 130.4, 128.8, 127.6, 127.5, 124.1, 122.0, 120.8

The NMR data is in agreement with that reported in the literature.[16]

Tetrabutylammonium 2-([2,2':6',2''-terpyridin]-4'-yl)benzenesulfonate (L6)

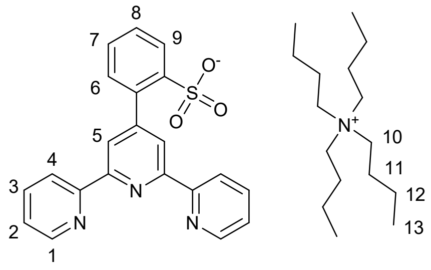

The cation exchange was identical to the procedure stated in **GP4** using sodium 2-([2,2':6',2''-terpyridin]-4'-yl)benzenesulfonate (411 mg, 1.0 mmol, 1.0 equiv.) as starting material and $TBAHSO_4$ (255 mg, 0.75 mmol, 0.75 equiv.) and NaOH (30 mg, 0.75 mmol, 0.75 equiv.) as reagents. Note, the DCM extract required additional aqueous washes (5× 10 mL). The target molecule was obtained as a yellow powder (404 mg, 0.64 mmol, 85%).

^{1}H NMR (400 MHz, $CDCl_3$): δ (ppm) 8.67 (s, 2H, H5), 8.64 (app. dt, J = 4.8, 1.3 Hz, 2H, H1), 8.60 (d, J = 8.0, 2H, H4), 8.28 – 8.21 (m, 1H, H6), 7.81 (app. td, J = 7.7, 1.8 Hz, 2H, H3), 7.36 (dtd, J = 12.4, 7.4, 1.8 Hz, 2H, H7, H9), 7.29 – 7.21 (m, 3H, H2, H8), 3.06 – 2.98 (m, 8H, H10), 1.44 – 1.33 (m, 8H, H11), 1.19 (h, J = 7.4 Hz, 8H, H12), 0.82 (t, J = 7.3 Hz, 12H, H13).

^{13}C NMR (101 MHz, $CDCl_3$): δ (ppm) 157.1, 154.1, 153.4, 149.2, 145.3, 137.8, 136.7, 131.0, 128.8, 128.5, 127.7, 123.4, 123.1, 121.5, 58.5, 24.0, 19.7, 13.8.

HRMS (ESI−, anion): found $[M]^-$, m/z($C_{21}H_{14}N_3O_3S^-$) calculated 388.0756, found 388.0744, δ = −1.6 ppm

4'-(*m*-tolyl)-2,2':6',2''-terpyridine

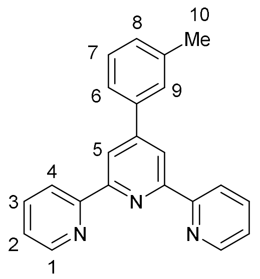

Prepared according to **GP2** using 3-methylbenzaldehyde as the starting material. Purification by silica gel chromatography (neutral silica, Hex:EtOAc – 10:1 to 7:1) afforded the target compound as a white powder (513 mg, 1.6 mmol, 32%).

^{1}H NMR (400 MHz, $CDCl_3$): δ (ppm) 8.79 – 8.71 (m, 4H, H4, H5), 8.67 (app. dt, J = 8.0, 1.1 Hz, 2H, H1), 7.87 (app. td, J = 7.7, 1.8 Hz, 2H, H3), 7.75 – 7.68 (m, 2H, H6, H8 *or* H9), 7.40 (app. t, J = 7.6 Hz, 1H, H7), 7.35 (ddd, J = 7.5, 4.8, 1.2 Hz, 2H, H2), 7.29 – 7.20 (m, 1H, H8 *or* H9), 2.47 (s, 3H, H10).

^{13}C NMR (101 MHz, $CDCl_3$): δ (ppm) 156.5, 156.0, 150.6, 149.3, 138.7, 138.6, 137.0, 129.9, 128.9, 128.1, 124.6, 123.9, 121.5, 119.1, 21.6

The NMR data is in agreement with that reported in the literature.[17]

Tetrabutylammonium (3-([2,2':6',2''-terpyridin]-4'-yl)phenyl)methanesulfonate (L7)

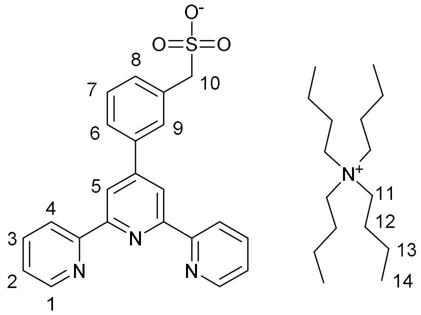

A variant of the procedure by Li *et. al.* was employed.[18] The 4'-(o-tolyl)-2,2':6',2''-terpyridine (388 mg, 1.2 mmol, 1.0 equiv.), NBS (285 mg, 1.6 mmol, 1.3 equiv.) and AIBN (11.8 mg, 0.072 mmol, 6 mol%) were dissolved in anhydrous degassed EtOAc (40 ml) and refluxed for 3 h. The formed precipitate was filtered away, and the filtrate was concentrated *in vacuo*. The sulfonate was prepared according to **GP4** using the crude bromination mixture. Purification stated in **GP4** with additional aqueous washes (2× 20 mL) of the DCM extract afforded the title compound as a colourless powder (31 mg, 0.05 mmol, 5% over 2 steps).

^{1}H NMR (500 MHz, $CDCl_3$): δ (ppm) 8.75 (s, 2H, H5), 8.71 (dd, J = 4.7, 0.8 Hz, 2H, H1), 8.64 (dd, J = 8.0, 1.4 Hz, 2H, H4), 8.03 (d, J = 2.0 Hz, 1H, H9), 7.86 (app. t, J = 7.8, 2H, H3), 7.78 (d, J = 7.7 Hz, 1H, H6), 7.62 (d, J = 7.6 Hz, 1H, H8), 7.41 (app. t, J = 7.7 Hz, 1H, H7), 7.33 (ddd, J = 7.4, 4.8, 1.1 Hz, 2H, H2), 4.19 (s, 2H, H10), 3.36 – 2.80 (m, 8H, H11), 1.55 – 1.38 (m, 8H, H12), 1.28 (h, J = 7.1 Hz, 8H, H13), 0.87 (t, J = 7.3 Hz, 12H, H14).

^{13}C NMR (126 MHz, $CDCl_3$): δ (ppm) 156.3, 156.0, 150.3, 149.2, 137.7, 136.9, 136.5, 131.9, 129.5, 128.6, 125.3, 123.9, 121.4, 118.9, 58.6, 57.8, 24.0, 19.7, 13.7

HRMS (ESI−, anion): found $[M + e^{-}]^{2-}$, m/z($C_{22}H_{16}N_3O_3S^{2-}$) calculated 402.0912, found 201.0457, δ = +3.2 ppm

4'-(*p*-tolyl)-2,2':6',2''-terpyridine

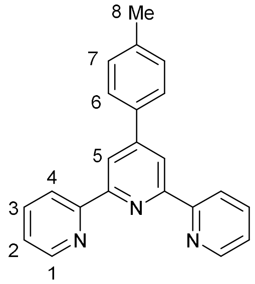

Prepared according to **GP2** using 4-methylbenzaldehyde as the starting material. Purification by silica gel chromatography (neutral silica, Hex:EtOAc – 10:1 to 7:1) afforded the target compound as a white powder (568 mg, 1.8 mmol, 35%).

^{1}H NMR (400 MHz, $CDCl_3$): δ (ppm) 8.76 – 8.70 (m, 4H, H4, H5), 8.67 (d, J = 8.0 Hz, 2H, H1), 7.93 – 7.76 (m, 4H, H3, H6), 7.39 – 7.28 (m, 4H, H2, H7), 2.42 (s, 3H, H8).

^{13}C NMR (101 MHz, $CDCl_3$): δ (ppm) 156.5, 156.0, 150.3, 149.2, 139.2, 137.0, 135.6, 129.8, 127.3, 123.9, 121.5, 118.7, 21.4.

The NMR data is in agreement with that reported in the literature.[17]

Tetrabutylammonium (3-([2,2':6',2''-terpyridin]-4'-yl)phenyl)methanesulfonate (L8)

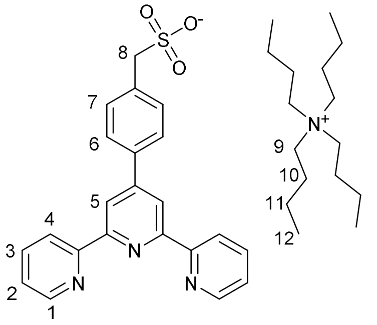

A variant of the procedure by Li *et. al.* was employed.[18] The 4'-(o-tolyl)-2,2':6',2''-terpyridine (388 mg, 1.2 mmol, 1.0 equiv.), NBS (285 mg, 1.6 mmol, 1.3 equiv.) and AIBN (11.8 mg, 0.072 mmol, 6 mol%) were dissolved in anhydrous degassed EtOAc (40 ml) and refluxed for 3 h. The formed precipitate was filtered away, and the filtrate was concentrated *in vacuo*. The sulfonate was prepared according to **GP4** using the crude bromination mixture. Purification stated in **GP4** with additional aqueous washes (2× 20 mL) of the DCM extract afforded the title compound as a white powder (195 mg, 0.30 mmol, 31% over 2 steps).

^{1}H NMR (400 MHz, $CDCl_3$): *δ* (ppm) 8.68 – 8.61 (m, 4H, H4, H5), 8.57 (dt, *J* = 8.0, 1.1 Hz, 2H, H1), 7.83 – 7.74 (m, 4H, H3, H6), 7.60 – 7.54 (m, 2H, H7), 7.33 – 7.21 (m, 2H, H2), 4.07 (s, 2H, H8), 3.06 – 2.91 (m, 8H, H9), 1.47 – 1.35 (m, 8H, H10), 1.26 (h, *J* = 7.3 Hz, 8H, H11), 0.85 (t, *J* = 7.3 Hz, 12H, H12).

^{13}C NMR (101 MHz, $CDCl_3$): *δ* (ppm) 156.0, 155.9, 149.8, 149.0, 136.8, 136.8, 136.1, 131.2, 126.5, 123.8, 121.2, 118.3, 58.2, 57.4, 23.7, 19.5, 13.5

HRMS (ESI−, anion): found $[M]^-$, m/z($C_{22}H_{17}N_3O_3S^-$) calculated 403.0991, found 403.0979, $\delta = -1.5$ ppm

4'-(*o*-tolyl)-2,2':6',2''-terpyridine

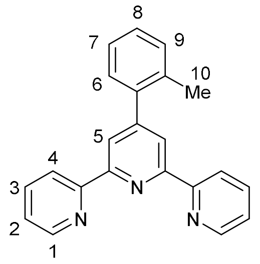

Prepared according to **GP2** using 2-methylbenzaldehyde as the starting material. Purification by silica gel chromatography (neutral silica, Hex:EtOAc – 10:1 to 7:1) afforded the target compound as off-white powder (586 mg, 1.8 mmol, 35%).

^{1}H NMR (500 MHz, $CDCl_3$): *δ* (ppm) 8.71 – 8.67 (m, 4H, H1, H4), 8.48 (s, 2H, H5), 7.90 – 7.84 (m, 2H, H3), 7.37 (dt, *J* = 7.2, 1.2 Hz, 1H, H6 *or* H8), 7.35 – 7.26 (m, 5H, H2, H6 *or* H8, H7, H9), 2.38 (s, 3H, H10).

^{13}C NMR (126 MHz, $CDCl_3$): *δ* (ppm) 156.4, 155.5, 152.1, 149.3, 139.8, 137.0, 135.3, 130.6, 129.5, 128.3, 126.0, 123.9, 121.7, 121.5, 20.3

The NMR data is in agreement with that reported in the literature.[19]

Tetrabutylammonium (2-([2,2':6',2''-terpyridin]-4'-yl)phenyl)methanesulfonate (L9)

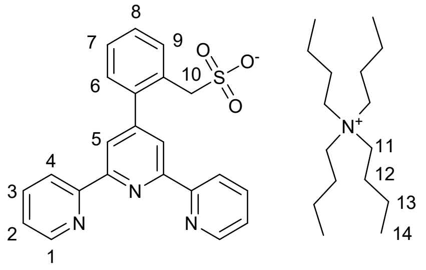

A variant of the procedure by Li *et. al.* was employed.[18] The 4'-(o-tolyl)-2,2':6',2''-terpyridine (388 mg, 1.2 mmol, 1.0 equiv.), NBS (285 mg, 1.6 mmol, 1.3 equiv.) and AIBN (11.8 mg, 0.072 mmol, 6 mol%) were dissolved in anhydrous degassed EtOAc (40 ml) and refluxed for 3 h. The formed precipitate was filtered away, and the filtrate was concentrated *in vacuo*. The sulfonate was prepared according to **GP4** using the crude bromination mixture. Purification stated in **GP4** afforded the title compound as a white powder (76 mg, 0.12 mmol, 10% over 2 steps).

^{1}H NMR (400 MHz, $CDCl_3$): δ (ppm) 8.69 – 8.59 (m, 4H, H1, H4), 8.52 (s, 2H, H5), 7.96 (dd, J = 7.8, 1.3 Hz, 1H, H9), 7.84 (app. td, J = 7.7, 1.8 Hz, 2H, H3), 7.44 – 7.27 (m, 5H, H2, H6, H7, H8), 4.21 (s, 2H, H10), 3.31 – 2.95 (m, 8H, H11), 1.61 – 1.42 (m, 8H, H12), 1.30 (h, J = 7.3 Hz, 8H, H13), 0.90 (t, J = 7.3 Hz, 12H, H14).

^{13}C NMR (101 MHz, $CDCl_3$): δ (ppm) 156.6, 155.2, 152.1, 149.1, 140.7, 137.0, 132.6, 131.7, 129.6, 128.0, 126.6, 123.7, 122.7, 121.8, 58.8, 54.2, 24.1, 19.8, 13.8

HRMS (ESI−, anion): found $[M]^-$, m/z($C_{22}H_{16}N_3O_3S^-$) calculated 402.0912, found 402.0908, $\delta = -1.0$ ppm

4,4''-di-*tert*-butyl-4'-(((tetrahydro-2H-pyran-2-yl)oxy)methyl)-2,2':6',2''-terpyridine

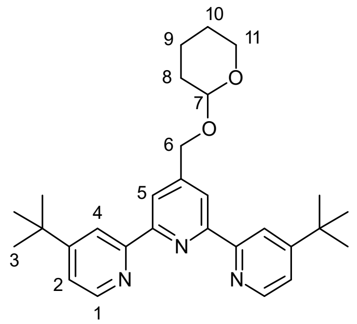

Prepared according to **GP1** using 2-bromo-4-*tert*-butylpyridine (3.00 g, 14.0 mmol, 1.75 equiv.), ^{n}BuLi (8.8 mL, 14.0 mmol, 1.75 equiv., 1.6 M in hexanes), $ZnCl_2$ (1.91 g, 14.0 mmol, 1.75 equiv.), 2,6-dibromo-4-(((tetrahydro-2H-pyran-2-yl)oxy)methyl)pyridine (1.40 g, 4.0 mmol, 1.0 equiv.) and $Pd(PPh_3)_4$ (231 mg, 0.2 mmol, 5 mol%). The reaction was refluxed for 48 h. Purification by silica gel chromatography (basified silica gel, PE:EtOAc – 15:1 to 3:1) afforded the title compound as a yellow powder (923 mg, 2.0 mmol, 50%).

^{1}H NMR (400 MHz, $CDCl_3$): δ (ppm) 8.73 (d, J = 2.0 Hz, 2H, H4), 8.60 (d, J = 5.2 Hz, 2H, H1), 8.44 (s, 2H, H5), 7.42-7.30 (m, 2H, H2), 4.95 (d, J = 13.3 Hz, 1H, H6), 4.79 (app. t, J = 3.4 Hz, 1H, H7), 4.67 (d, J = 13.3 Hz, 1H, H6), 3.92 (ddd, J = 11.7, 6.1, 1.9 Hz, 1H, H11), 3.64 – 3.49 (m, 1H, H11), 2.01 – 1.85 (m, 1H, H8), 1.77 (tt, J = 5.2, 2.8 Hz, 2H, H10), 1.66 – 1.49 (m, 3H, H8, H9), 1.42 (s, 18H, H3).

^{13}C NMR (101 MHz, $CDCl_3$): δ (ppm) 160.8, 156.2, 155.7, 149.8, 149.2, 121.1, 119.4, 118.3, 98.4, 67.9, 62.1, 35.0, 30.6, 30.6, 25.6, 19.3

HRMS (ESI+): found $[M + H]^+$, m/z($C_{29}H_{38}N_3O_2^+$) calculated 460.2959, found 460.2959, δ = +0.2 ppm

4'-(bromomethyl)-4,4''-di-*tert*-butyl-2,2':6',2''-terpyridine

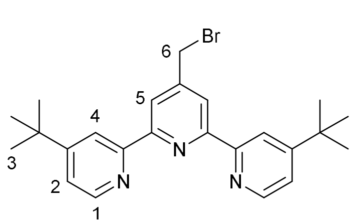

Prepared according to **GP3** using 4,4''-di-*tert*-butyl-4'-(((tetrahydro-2H-pyran-2-yl)oxy)methyl)-2,2':6',2''-terpyridine (460 mg, 1.0 mmol, 1.0 equiv.) as the starting material. Purification by silica gel chromatography (basified silica gel, Hex:EtOAc – 9:1 to 5:1) afforded the title compound as a white powder (300 mg, 0.68 mmol, 68%).

^{1}H NMR (400 MHz, $CDCl_3$): δ (ppm) 8.75 (dd, J = 2.0, 0.8 Hz, 2H, H4), 8.64 (dd, J = 5.3, 0.7 Hz, 2H, H1), 8.49 (s, 2H, H5), 7.38 (dd, J = 5.2, 2.0 Hz, 2H, H2), 4.59 (s, 2H, H6), 1.46 (s, 18H, H3).

^{13}C NMR (101 MHz, $CDCl_3$): δ (ppm) 161.0, 156.3, 155.7, 149.3, 148.4, 121.4, 120.8, 118.4, 35.1, 31.2, 30.7.

HRMS (ESI+): found $[M]^{2+}$, m/z($C_{24}H_{28}BrN_3^{2+}$) calculated 219.5719, found 219.5718, δ = −0.7 ppm

Sodium (4,4''-di-*tert*-butyl-[2,2':6',2''-terpyridin]-4'-yl)methanesulfonate (L10)

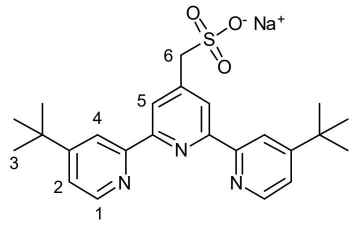

The 4'-(bromomethyl)-4,4''-di-*tert*-butyl-2,2':6',2''-terpyridine (175 mg, 0.4 mmol, 1.0 equiv.), sodium sulfite (73 mg, 0.6 mmol, 1.5 equiv.) were dissolved in acetone/water (2.0 mL, 2:3, *v*/*v*) and refluxed for 2 h. The cooled mix was concentrated in vacuo, and the residue was sonicated with DCM (3× 10 mL). Combined organics were filtered through a plug of Celite and concentrated in vacuo to yield the compound as yellow crystals (185 mg, 0.4 mmol, quantitative).

^{1}H NMR (400 MHz, DMSO-d^6): δ (ppm) 8.69 (dd, J = 2.0, 0.7 Hz, 2H, H4), 8.62 (dd, J = 5.2, 0.7 Hz, 2H, H1), 8.37 (s, 2H, H5), 7.51 (dd, J = 5.2, 2.0 Hz, 2H, H2), 3.94 (s, 2H, H6), 1.39 (s, 18H, H3).

^{13}C NMR (101 MHz, DMSO-d^6): δ (ppm) 160.3, 155.3, 154.1, 149.2, 146.6, 122.4, 121.3, 117.0, 57.4, 40.2, 39.9, 39.7, 39.5, 39.3, 39.1, 38.9, 34.7, 30.1.

HRMS (ESI−, anion): found $[M]^{-}$, m/z($C_{24}H_{28}N_3O_3S^{-}$) calculated 438.1857, found 438.1865, δ = +1.8 ppm

Note: The sodium salt is partially soluble in all organic solvents; therefore, the ion exchange to tetrabutylammonium salt was not conducted.

Synthesis of Pre-formed Palladium Terpyridine Complexes

[(2,2':6',2''-terpyridine)(acetonitrile)palladium](2+) tetrafluoroborate (Complex 1)

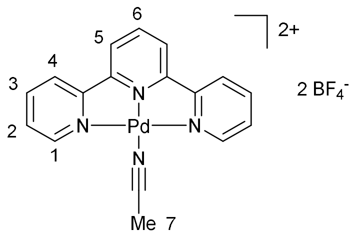

Prepared according to **GP5** using 2,2':6',2''-terpyridine (466 mg, 2.0 mmol, 1.0 equiv.) as the starting material and $Pd(OAc)_2$ (471 mg, 2.2 mmol, 1.1 equiv.) and tetrafluoroboric acid diethyl etherate (0.6 mL, 4.4 mmol, 2.2 equiv.) as reagents. Purification stated in **GP5** afforded the title compound as a tan powder (937 mg, 1.7 mmol, 84%).

^{1}H NMR (500 MHz, CD_3CN): δ (ppm) 8.57 (dd, J = 5.6, 0.9 Hz, 2H, H1), 8.51 (app. t, J = 8.2 Hz, 1H, H6), 8.42 (app. td, J = 7.9, 1.5 Hz, 2H, H3), 8.34 (ddd, J = 8.1, 1.5, 0.7 Hz, 2H, H4), 8.28 (d, J = 8.1 Hz, 2H, H5), 7.84 (ddd, J = 7.8, 5.6, 1.5 Hz, 2H, H2), 1.96 (s, 3H, H7).

^{13}C NMR (126 MHz, DMSO-d^6): δ (ppm) 157.1, 155.2, 150.6, 143.5, 143.2, 129.0, 125.5, 124.7, 118.1, 1.2

^{19}F NMR (471 MHz, DMSO-d^6): δ (ppm) −152.5 (s), −152.6 (s)

^{11}B NMR (160 MHz, DMSO-d^6): δ (ppm) −1.84

HRMS (ESI+, cation): found $[M + HCOO^-]^+$, m/z($C_{16}H_{12}N_3O_2Pd^+$) calculated 383.9964, found 383.9968, δ = −1.0 ppm

Elemental analysis: calculated for $C_{17}H_{14}B_2F_8N_4Pd$: C 36.83, H 2.55, N 10.11, Pd 19.20; found C 36.88, H 2.45, N 9.97, Pd 19.10.

ε^1_{MeCN} (345 nm) = 8 760 $dm^3 \cdot mol^{-1} \cdot cm^{-1}$, ε^2_{MeCN} (362 nm) = 7 850 $dm^3 \cdot mol^{-1} \cdot cm^{-1}$

ε^1_{water} (344 nm) = 4 680 $dm^3 \cdot mol^{-1} \cdot cm^{-1}$, ε^2_{water} (362 nm) = 4 540 $dm^3 \cdot mol^{-1} \cdot cm^{-1}$

The spectroscopic data is in agreement with that reported in the literature.[20]

[((2,2':6',2''-terpyridin)-4'-ylmethanesulfonate)(acetonitrile)palladium](+) tetrafluoroborate (Complex 2)

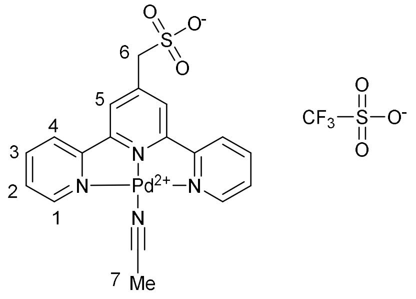

Prepared according to **GP5** using tetrabutylammonium [2,2':6',2"-terpyridin]-4'-ylmethanesulfonate (228 mg, 0.40 mmol, 1.0 equiv.) as the starting material and $Pd(OAc)_2$ (99 mg, 0.44 mmol, 1.1 equiv.) and trifluoromethanesulfonate (78 µL, 0.88 mmol, 2.2 equiv., 1:12.7 v/v solution in MeCN) as reagents. Purification stated in **GP5** afforded the title compound as a tan powder (160 mg, 0.26 mmol, 64%)

^{1}H NMR (400 MHz, DMSO-d^6): δ (ppm) 8.58 (s, 2H, H5), 8.55 (d, J = 7.8 Hz, 2H, H4), 8.47 (app. td, J = 7.8, 1.5 Hz, 2H, H3), 8.21 (d, J = 5.5 Hz, 2H, H1), 7.86 (dd, J = 7.7, 5.7 Hz, 2H, H2), 4.14 (s, 2H, H6), 2.07 (s, 3H, H7).

^{13}C NMR (176 MHz, DMSO-d^6): δ (ppm) 157.0, 154.0, 153.4, 150.5, 143.4, 129.0, 126.3, 125.1, 120.7 (q, J = 322.8 Hz), 118.1, 57.6, 1.2

^{19}F NMR (471 MHz, DMSO-d^6): δ (ppm) −78.3 (s)

HRMS (ESI+, cation): found $[M]^+$, $[C_{18}H_{15}N_4O_3PdS]^+$, m/z($C_{18}H_{15}N_4O_3PdS^+$) calculated 472.9894 found 472.9894, δ = −1.6 ppm

Elemental analysis: calculated for $C_{19}H_{15}F_3N_4O_6PdS_2$: C 36.64, H 2.43, N 8.99, Pd 17.08; found C 35.75, H 2.45, N 8.02, Pd 17.40

ε^1_{water} (345 nm) = 4 170 $dm^3 \cdot mol^{-1} \cdot cm^{-1}$, ε^2_{water} (362 nm) = 4 080 $dm^3 \cdot mol^{-1} \cdot cm^{-1}$

[(4’-methyl-2,2':6',2''-terpyridine)(acetonitrile)palladium](2+) tetrafluoroborate (Complex 3)

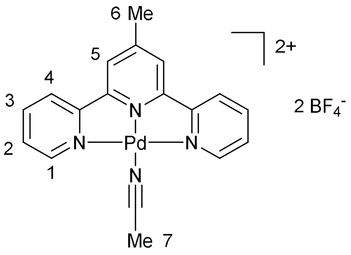

Prepared according to **GP5** using 4’-methyl-2,2':6',2''-terpyridine (99 mg, 0.40 mmol, 1.0 equiv.) as the starting material and $Pd(OAc)_2$ (99 mg, 0.44 mmol, 1.1 equiv.) and tetrafluoroboric acid diethyl etherate (0.12 mL, 0.88 mmol, 2.2 equiv.) as reagents. Purification stated in **GP5** afforded the title compound as a tan powder (179 mg, 0.31 mmol, 79%).

^{1}H NMR (500 MHz, CD_3CN): δ (ppm) 8.56 (ddd, J = 5.7, 1.5, 0.6 Hz, 2H, H1), 8.42 (app. td, J = 7.9, 1.5 Hz, 2H, H3), 8.28 (ddd, J = 8.0, 1.4, 0.6 Hz, 2H, H4), 8.17 (d, J = 0.8 Hz, 2H, H5), 7.82 (ddd, J = 7.8, 5.6, 1.4 Hz, 2H, H2), 2.70 (d, J = 0.7 Hz, 3H, H6), 1.96 (s, 3H, H7).

^{13}C NMR (126 MHz, DMSO-d^6): δ (ppm) 157.1, 156.3, 154.4, 150.6, 143.2, 128.8, 125.2, 125.1, 118.1, 21.9, 1.2

^{19}F NMR (471 MHz, DMSO-d^6): δ (ppm) −148.7 (s), −148.8 (m)

^{11}B NMR (160 MHz, DMSO-d^6): δ (ppm) −1.83

HRMS (ESI+, cation): found $[M]^{2+}$, $[C_{18}H_{16}N_4Pd]^{2+}$, m/z($C_{18}H_{16}N_4Pd^{2+}$) calculated 197.0199 found 197.0200, δ = +0.5 ppm

Elemental analysis: calculated for $C_{18}H_{16}B_2F_8N_4Pd$: C 38.04, H 2.84, N 9.86, Pd 18.72; found C 38.37, H 2.92, N 10.28, Pd 20.09

ε^1_{MeCN} (342 nm) = 8 320 $dm^3 \cdot mol^{-1} \cdot cm^{-1}$, ε^2_{MeCN} (359 nm) = 7 600 $dm^3 \cdot mol^{-1} \cdot cm^{-1}$

The spectroscopic data is in agreement with that reported in the literature.[21]

[((4,4''-di-*tert*-butyl-[2,2':6',2''-terpyridin]-4'-yl)methanesulfonate)(acetonitrile)palladium](2+) tetrafluoroborate (Complex 4)

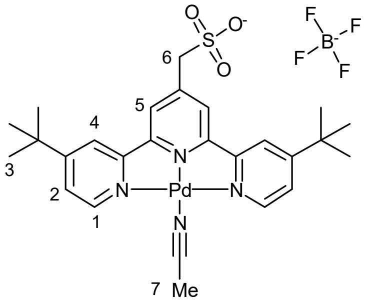

Prepared according to **GP5** using sodium (4,4''-di-*tert*-butyl-[2,2':6',2''-terpyridin]-4'-yl)methanesulfonate (69 mg, 0.15 mmol, 1.0 equiv.) as the starting material and $Pd(OAc)_2$ (37 mg, 0.17 mmol, 1.1 equiv.) and tetrafluoroboric acid diethyl etherate (45 µL, 0.33 mmol, 2.2 equiv.) as reagents. Purification stated in **GP5** afforded the title compound as a tan powder (48 mg, 71 µmol, 48%).

^{1}H NMR (400 MHz, DMSO-d^6): δ (ppm) 8.84 (s, 2H, H5), 8.66 (s, 2H, H4), 8.32 (app. s, 2H, H1), 7.82 (dd, J = 6.0, 2.1 Hz, 2H, H2), 4.10 (s, 2H, H6), 2.07 (s, 3H, H7), 1.42 (s, 18H, H3).

^{13}C NMR (176 MHz, DMSO-d^6): δ (ppm) 167.5, 157.1, 154.3, 153.1, 150.2, 126.4, 125.1, 122.8, 118.1, 57.7, 36.2, 29.7, 1.2.

^{19}F NMR (376 MHz, DMSO-d^6): δ (ppm) −148.78 (m), −148.83 (q, J = 1.1 Hz).

^{11}B NMR (128 MHz, DMSO-d^6): δ (ppm) −1.85

HRMS (ESI+, cation): found [M + HCOOH]$^+$, [$C_{25}H_{30}N_3O_5PdS$]$^+$, m/z($C_{25}H_{30}N_3O_5PdS^+$) calculated 590.0941 found 590.0930, δ = −1.9 ppm

Elemental analysis: calculated for $C_{26}H_{31}BF_4N_4O_3PdS$: C 46.41, H 4.64, N 8.33; found C 43.81, H 4.38, N 7.68

ε^1_{DMSO} (348 nm) = 11 590 dm^3·mol^{-1}·cm^{-1}, ε^2_{DMSO} (366 nm) = 10 690 dm^3·mol^{-1}·cm^{-1}

Note: A drop of D_2O had to be added to fully solubilise the complex in DMSO-d^6.

Synthesis of 2-substituted 1,10-phenanthrolines

1,10-phenanthrolin-2-amine

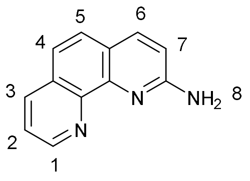

A variant of the protocol by Nakatani *et. al.* was adapted.[22] The 2-chloro-1,10-phenanthroline (1.07 g, 5.0 mmol, 1.0 equiv.), acetamide (5.79 g, 115 mmol, 23 equiv.) and potassium carbonate (4.49 g, 32.5 mmol, 6.5 equiv.) were all mixed and heated to 250 °C for 5 h. The mix was allowed to cool to rt, followed by dilution with water (30 mL). The aqueous phase was extracted with $CHCl_3$ (3×), and the combined organics were dried over $MgSO_4$ and concentrated *in vacuo*. Purification by silica gel chromatography (EtOAc:MeOH – 100:0 to 94:6) and sonication with water (15 mL) yielded the title compound as a yellow fine powder (321 mg, 1.6 mmol, 33%).

^{1}H NMR (400 MHz, $CDCl_3$): δ (ppm) 9.07 (dd, J = 4.3, 1.7 Hz, 1H, H1), 8.13 (dd, J = 8.1, 1.7 Hz, 1H, H3), 7.91 (d, J = 8.6 Hz, 1H, H6), 7.59 (d, J = 8.6 Hz, 1H, H5), 7.54 – 7.40 (m, 2H, H2, H4), 6.87 (d, J = 8.6 Hz, 1H, H7), 5.27 (br s, 2H, H8).

^{13}C NMR (101 MHz, $CDCl_3$): δ (ppm) 157.8, 149.6, 145.7, 145.0, 138.2, 136.0, 129.3, 126.5, 122.8, 122.5, 121.9, 112.0

The NMR data is in agreement with that reported in the literature.[22]

1,10-phenanthrolin-2-ol

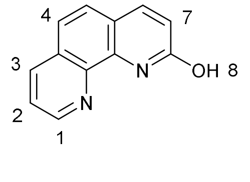

The title compound was isolated as a byproduct of 1,10-phenanthroline-2-amine preparation. The compound was obtained as a yellow powder (31 mg, 0.16 mmol, 3%).

^{1}H NMR (400 MHz, $CDCl_3$): δ (ppm) 10.69 (s, 1H, H8), 8.91 (dd, J = 4.3, 1.7 Hz, 1H, H1), 8.21 (dd, J = 8.3, 1.7 Hz, 1H, H3), 7.88 (d, J = 9.4 Hz, 1H, H6), 7.62 (d, J = 8.6 Hz, 1H, H5), 7.59 – 7.54 (m, 1H, H2), 7.54 (d, J = 8.8 Hz, 1H, H4), 6.85 (d, J = 9.4 Hz, 1H, H7).

^{13}**C NMR** (101 MHz, $CDCl_3$): δ (ppm) 162.1, 149.5, 140.3, 136.8, 136.2, 135.5, 128.5, 125.5, 123.9, 123.3, 121.1, 117.5

The NMR data is in agreement with that reported in the literature.[23]

1-(1,10-phenanthrolin-2-yl)ethan-1-one

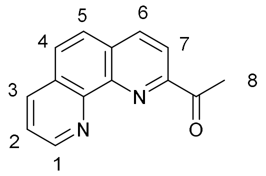

A variant of the protocol by Zhu was adapted.[24] The 2-chloro-1,10-phenanthroline (429 mg, 2.0 mmol, 1.0 equiv.) and $Pd(PPh_3)_4$ (231 mg, 0.2 mmol, 10 mol%) were introduced to the flash and flushed with nitrogen/vacuum cycle (3×). Then, the 1-ethoxyvinyltri-n-butylstannane (0.81 mL, 2.4 mmol, 1.2 equiv.) and anhydrous DMF (25 mL) were added, and the mixture was refluxed for 24 h. The mixture was allowed to cool to rt followed by quenching with sat. aq. KF solution. The aqueous phase was extracted with Et_2O (3×) and the combined organics were dried over $MgSO_4$ and concentrated *in vacuo*. The crude product was dissolved in acetone (30 mL) and stirred with conc. aq. HCl (10 mL) overnight. Then, the mixture was neutralised with sat. aq. $NaHCO_3$ solution. Neutralised mixture was concentrated in vacuo and the aqueous phase was extracted with DCM (3×) and the combined organic were dried over $MgSO_4$ and concentrated *in vacuo*. Purification by silica gel chromatography (Hex:EtOAc – 10:1 to 1:1) afforded the title compound as a white powder (124 mg, 0.6 mmol, 28%).

^{1}**H NMR** (400 MHz, $CDCl_3$): δ (ppm) 9.19 (dd, J = 4.4, 1.8 Hz, 1H, H1), 8.28 (app. br s, 2H, H6, H7), 8.22 (dd, J = 8.1, 1.8 Hz, 1H, H3), 7.82 (d, J = 8.8 Hz, 1H, H4), 7.75 (d, J = 8.8 Hz, 1H, H5), 7.62 (dd, J = 8.1, 4.4 Hz, 1H, H2), 3.03 (s, 3H, H8).

^{13}**C NMR** (126 MHz, $CDCl_3$): δ (ppm) 201.0, 153.4, 151.0, 146.4, 145.3, 137.2, 136.5, 130.8, 129.2, 128.9, 126.4, 123.5, 120.6, 26.2

The ^{1}H NMR data is in agreement with that reported in the literature.[25]

1,10-phenanthroline-1-oxide

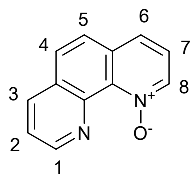

A variant of the protocol reported by van der Plas *et al.* was reported.[26] To the 1,10-phenanthroline (1.44 g, 8.0 mmol) solution in glacial acetic acid (10.0 mL), the H_2O_2 (1.0 mL, 30% w/v) was slowly added. The solution was heated to reflux for 3 h, which following more H_2O_2 (1.0 mL, 30% w/v) was added and the solution was refluxed for a further 3 h. The mixture was allowed to cool to rt and the final portion of H_2O_2 (0.7 mL, 30% w/v) was slowly added, and the mixture was stirred at rt for 16 h. The reaction mixture was concentrated *in vacuo* to approximately a quarter of its volume. Then, water (5.0 mL) was slowly added, and the solution was again concentrated to a quarter of its volume *in vacuo*. The solid Na_2CO_3 (15 g) was added portionwise, affording a green slurry which was extracted with Soxhlet apparatus using $CHCl_3$ (150 mL) for 9 h. The combined organics were stirred with $MgSO_4$ and decolourising charcoal for 1 h. The filtration through Celite® and concentration *in vacuo* yielded the title compound as a yellow powder (539 mg, 2.7 mmol, 34%).

^{1}H NMR (500 MHz, $CDCl_3$): δ (ppm) 9.30 (dd, J = 4.4, 1.9 Hz, 1H, H8), 8.72 (dd, J = 6.3, 1.2 Hz, 1H, H1), 8.22 (dd, J = 8.1, 1.9 Hz, 1H, H6), 7.78 (d, J = 8.8 Hz, 1H, H5), 7.73 (d, J = 5.9 Hz, 1H, H4), 7.71 (d, J = 4.7 Hz, 1H, H3), 7.65 (dd, J = 8.0, 4.3 Hz, 1H, H7), 7.44 (dd, J = 8.1, 6.3 Hz, 1H, H2).

^{13}C NMR (126 MHz, $CDCl_3$): δ (ppm) 150.1, 142.8, 140.9, 138.5, 136.0, 133.4, 129.2, 129.0, 126.6, 124.5, 123.3, 122.9

The NMR data is in agreement with that reported in the literature.[27]

***N,N,N*-trimethyl-1,10-phenanthrolin-2-aminium tetrafluoroborate**

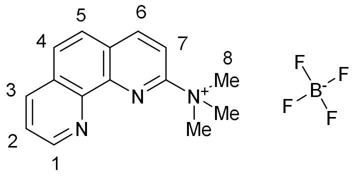

An adapted version of the protocol reported by Yang was used.[28] The flask was charged with 1,10-phenanthroline-1-oxide (157 mg, 0.8 mmol, 1.0 equiv.) followed by the addition of NMe_3 (2.0 mL, 4.0 mmol, 5.0 equiv., 2M in THF) and anhydrous DCM (6.0 mL). The mix was cooled to 0 °C, and

TFAA (0.33 mL, 2.4 mmol, 3.0 equiv.) was added dropwise. After complete addition, the reaction mixture was warmed to rt and stirred overnight. The mix was diluted with DCM (1.0 mL), and the slurry was poured into a flask with Et_2O (50 mL). The precipitate was collected by gravity filtration and washed with Et_2O (20 mL). The crude product was redissolved in water (20 mL) and charged with $NaBF_4$ solution (2 M in water). Gravity filtration followed by washings with water and EtOAc and drying overnight yielded the product as a yellowish powder (120 mg, 0.37 mmol, 46%).

^{1}H NMR (500 MHz, CD_3CN): δ (ppm) 9.20 (dd, J = 4.6, 1.7 Hz, 1H, H1), 8.83 (d, J = 8.9 Hz, 1H, H4 or H5), 8.65 (d, J = 8.2 Hz, 1H, H3), 8.28-8.08 (m, 3H, H4 or H5, H6, H7), 7.94 (dd, J = 8.1, 4.6 Hz, 1H, H2), 3.75 (s, 9H, H8).

^{13}C NMR (176 MHz, CD_3CN): δ (ppm) 156.2, 150.6, 144.1, 143.6, 143.1, 139.8, 130.8, 131.1, 130.1, 127.2, 125.8, 115.2, 56.3

^{19}F NMR (471 MHz, CD_3CN): δ (ppm) −152.7

^{11}B NMR (160 MHz, CD_3CN): δ (ppm) −2.02

The NMR data is in agreement with that reported in the literature.[28]

2-phenyl-1,10-phenanthroline

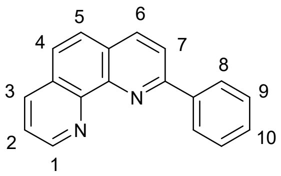

An adapted version of the protocol reported by Advincula was employed.[29] The 1,10-phenanthroline (541 mg, 3.0 mmol, 1.0 equiv.) was dispersed in anhydrous toluene (7.5 mL), and the PhLi (1.7 mmol, 3.3 mmol, 1.1 equiv., 1.9M in ^{n}Bu$_2$O) was added dropwise at rt. The mix was left to stir at rt for 3 h before the addition of water (5 mL). The aqueous phase was extracted with DCM (2×), and the combined organics were re-oxidised by the addition of MnO_2 (3× 1.0 g over 15 mins). The suspension was filtered through Celite, and the filtrate was concentrated *in vacuo*. Purification by silica gel chromatography yielded the title compound as a yellow powder (270 mg, 1.1 mmol, 35%).

^{1}H NMR (500 MHz, $CDCl_3$): δ (ppm) 9.24 (dd, J = 4.3, 1.8 Hz, 1H, H1), 8.36 – 8.31 (m, 2H, H8), 8.28 (d, J = 8.4 Hz, 1H, H6), 8.24 (dd, J = 8.0, 1.8 Hz, 1H, H3), 8.09 (d, J = 8.4 Hz, 1H, H7), 7.79 (d, J = 8.7 Hz, 1H, H, H5), 7.75 (d, J = 8.8 Hz, 1H, H, H4), 7.62 (dd, J = 8.0, 4.3 Hz, 1H, H2), 7.54 (t, J = 7.5 Hz, 2H, H9), 7.47 (tt, J = 7.3, 1.2 Hz, 1H, H10).

^{13}**C NMR** (126 MHz, $CDCl_3$): δ (ppm) 157.7, 150.5, 146.6, 146.2, 139.8, 136.9, 136.2, 129.4, 129.2, 128.9, 128.1, 127.6, 126.5, 126.4, 123.0, 120.7

The NMR data is in agreement with that reported in the literature.[30]

2-(4-(trifluoromethyl)phenyl)-1,10-phenanthroline

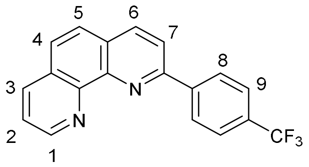

Prepared according to the **GP6** using 2-chloro-1,10-phenanthroline (322 mg, 1.5 mmol, 1.0 equiv.) and 4-trifluoromethylphenyl boronic acid (370 mg, 2.0 mmol, 1.3 equiv.) as starting materials. Purification by silica gel chromatography (Hex:EtOAc – 4:1 to 1:1) yielded the target compound as a white powder (480 mg, 1.5 mmol, quantitative).

^{1}**H NMR** (700 MHz, $CDCl_3$): 9.25 (dd, J = 4.3, 1.7 Hz, 1H, H1), 8.44 (d, J = 7.9 Hz, 2H, H9), 8.34 (d, J = 8.4 Hz, 1H, H6), 8.27 (dd, J = 8.0, 1.8 Hz, 1H, H3), 8.10 (d, J = 8.3 Hz, 1H, H7), 7.83 (d, J = 8.7 Hz, 1H, H5), 7.82 – 7.78 (m, 3H, H4, H8), 7.66 (dd, J = 8.0, 4.3 Hz, 1H, H2).

^{13}**C NMR** (176 MHz, $CDCl_3$): δ (ppm) 156.1, 150.7, 146.4, 146.3, 143.0, 137.3, 136.4, 131.1 (q, J = 32.5 Hz), 129.3, 128.4, 128.1, 127.0, 126.4, 125.8 (q, J = 3.8 Hz), 124.4 (q, J = 272.4 Hz), 123.3, 120.8

^{19}**F NMR** (471 MHz, $CDCl_3$): δ (ppm) −62.5

HRMS (ESI+): found $[M + H]^+$, m/z($C_{19}H_{12}F_3N_2^+$) calculated 325.0947, found 325.0955, δ = +2.4 ppm

2-(2-(trifluoromethyl)phenyl)-1,10-phenanthroline

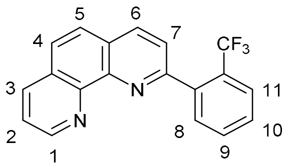

Prepared according to the **GP6** using 2-chloro-1,10-phenanthroline (322 mg, 1.5 mmol, 1.0 equiv.) and 2-trifluoromethylphenyl boronic acid (370 mg, 2.0 mmol, 1.3 equiv.) as starting materials. Purification by silica gel chromatography (Hex:EtOAc – 4:1 to 2:3) yielded the target compound as a white powder (269 mg, 0.8 mmol, 43%).

^{1}**H NMR** (700 MHz, $CDCl_3$): *δ* (ppm) 9.20 (dd, *J* = 4.2, 1.8 Hz, 1H, H1), 8.27 (d, *J* = 8.2 Hz, 1H, H6), 8.23 (dd, *J* = 8.0, 1.8 Hz, 1H, H3), 7.82 (d, *J* = 8.8 Hz, 1H, H4), 7.79 (d, *J* = 8.8 Hz, 1H, H5), 7.78 – 7.75 (m, 2H, H7, H11), 7.73 (d, *J* = 7.6 Hz, 1H, H8), 7.64 (app. t, *J* = 7.6 Hz, 1H, H10), 7.60 (dd, *J* = 8.0, 4.3 Hz, 1H, H2), 7.54 (app. t, *J* = 8.0 Hz, 1H, H9).

^{13}**C NMR** (176 MHz, $CDCl_3$): *δ* (ppm) 158.2, 150.6, 146.4, 145.9, 140.6, 136.2, 135.9, 132.5, 131.8, 129.1, 128.8 (q, *J* = 31.0 Hz), 128.5, 127.7, 126.9, 126.4, 126.2 (q, *J* = 5.1 Hz), 124.6 (q, *J* = 274.1 Hz), 124.0, 123.1.

^{19}**F NMR** (376 MHz, $CDCl_3$): *δ* (ppm) −56.2

HRMS (ESI+): found $[M + H]^+$, m/z($C_{19}H_{11}F_3N_2^+$) calculated 325.0947, found 325.0952, *δ* = +1.4 ppm

2-(3,5-bis(trifluoromethyl)phenyl)-1,10-phenanthroline

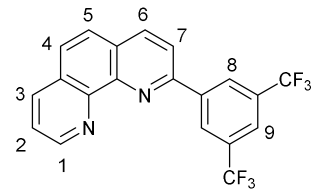

Prepared according to the **GP6** using 2-chloro-1,10-phenanthroline (322 mg, 1.5 mmol, 1.0 equiv.) and 3,5-bis(trifluoromethyl)phenyl boronic acid (503 mg, 2.0 mmol, 1.3 equiv.) as starting materials. Purification by silica gel chromatography (Hex:EtOAc – 4:1 to 1:1) yielded the target compound as an off-white powder (340 mg, 0.9 mmol, 58%).

^{1}**H NMR** (400 MHz, $CDCl_3$): *δ* (ppm) 9.19 (dd, *J* = 4.3, 1.7 Hz, 1H, H1), 8.70 (s, 2H, H8), 8.29 (d, *J* = 8.4 Hz, 1H, H6), 8.20 (dd, *J* = 8.1, 1.7 Hz, 1H, H3), 8.02 (d, *J* = 8.3 Hz, 1H, H7), 7.96 (s, 1H, H9), 7.75 (d, *J* = 9.0 Hz, 1H, H4), 7.74 (d, *J* = 8.9 Hz, 1H, H5), 7.61 (dd, *J* = 8.0, 4.3 Hz, 1H, H2).

^{13}**C NMR** (101 MHz, $CDCl_3$): *δ* (ppm) 154.2, 150.5, 146.2, 146.0, 141.8, 137.5, 136.1, 132.1 (q, *J* = 33.4 Hz), 129.2, 128.1, 128.1 – 127.8 (m), 127.4, 126.1, 123.4 (q, *J* = 273.9 Hz), 123.3, 122.7 (app. p, *J* = 3.8 Hz), 120.4

^{19}**F NMR** (471 MHz, $CDCl_3$): *δ* (ppm) −62.6

HRMS (ESI+): found $[M + H]^+$, m/z($C_{20}H_{10}F_6N_2^+$) calculated 393.0821, found 393.0822, *δ* = +0.2 ppm

2-(3,4,5-trifluorophenyl)-1,10-phenanthroline

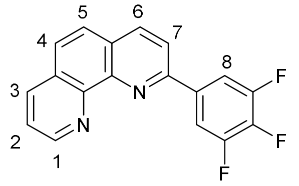

Prepared according to the **GP6** using 2-chloro-1,10-phenanthroline (322 mg, 1.5 mmol, 1.0 equiv.) and 3,4,5-trifluorophenyl boronic acid (343 mg, 2.0 mmol, 1.3 equiv.) as starting materials. Purification by silica gel chromatography (Hex:EtOAc – 4:1 to 1:1) yielded the target compound as an off-white powder (66 mg, 0.2 mmol, 14%).

^{1}H NMR (500 MHz, $CDCl_3$): δ (ppm) 9.22 (dd, J = 4.3, 1.8 Hz, 1H, H1), 8.31 (d, J = 8.4 Hz, 1H, H6), 8.25 (dd, J = 8.1, 1.8 Hz, 1H, H3), 8.01 – 7.90 (m, 3H, H4, H5, H7), 7.79 (s, 2H, H8), 7.65 (dd, J = 8.0, 4.3 Hz, 1H, H2).

^{13}C NMR (126 MHz, $CDCl_3$): δ (ppm) 154.1 (app. q, J = 2.4 Hz), 151.7 (ddd, J = 249.8, 10.2, 3.9 Hz), 150.7, 146.3, 146.2, 140.8 (dt, J = 254.2, 15.5 Hz), 137.4, 136.3, 136.0 – 135.6 (m), 129.3, 128.1, 127.2, 126.3, 123.3, 112.0, 112.1 (dd, J = 16.9, 5.4 Hz).

^{19}F NMR (471 MHz, $CDCl_3$): δ (ppm) −133.90 (d, J = 20.5 Hz, 2F), −159.58 (t, J = 20.5 Hz, 1F).

HRMS (ESI+): found $[M + H]^+$, m/z($C_{18}H_9F_3N_2^+$) calculated 311.0791, found 311.0795, δ = +1.5 ppm

2-mesityl-1,10-phenanthroline

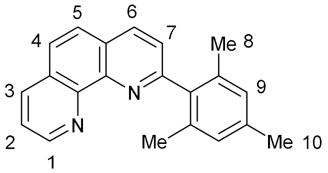

Prepared according to the **GP6** using 2-chloro-1,10-phenanthroline (322 mg, 1.5 mmol, 1.0 equiv.) and mesityl boronic acid (320 mg, 2.0 mmol, 1.3 equiv.) as starting materials. Purification by silica gel chromatography (Hex:EtOAc – 4:1 to 2:3) yielded the target compound as a yellow powder (198 mg, 0.7 mmol, 44%, 46% brsm).

^{1}H NMR (500 MHz, $CDCl_3$): δ (ppm) 9.23 – 8.99 (m, 1H, H1), 8.24 (dd, J = 8.1, 1.6 Hz, 1H, H6), 8.18 (app. dt, J = 8.1, 1.7 Hz, 1H, H3), 7.78 (dd, J = 8.9, 1.3 Hz, 1H, H5), 7.73 (dd, J = 8.8, 1.3 Hz, 1H, H4), 7.61 – 7.48 (m, 2H, H2, H7), 6.93 (s, 2H, H9), 2.33 (s, 3H, H10), 2.07 (s, 6H, H8).

^{13}C NMR (126 MHz, $CDCl_3$): δ (ppm) 161.0, 150.5, 138.6, 137.5, 136.2, 136.2, 136.1, 129.0, 128.3, 127.2, 126.6, 126.4, 125.0, 122.98, 21.3, 20.6

The ^{1}H NMR data is in agreement with that reported in the literature.[30]

2-(3,5-di-*tert*-butylphenyl)-1,10-phenanthroline

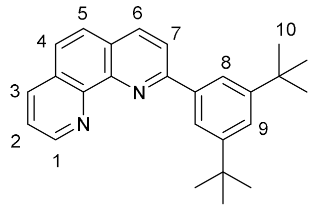

Prepared according to the **GP6** using 2-chloro-1,10-phenanthroline (322 mg, 1.5 mmol, 1.0 equiv.) and 3,5-di-*tert*-butylphenyl boronic acid (457 mg, 2.0 mmol, 1.3 equiv.) as starting materials. Purification by silica gel chromatography (Hex:EtOAc – 4:1 to 2:3) yielded the target compound as a yellow powder (93 mg, 0.3 mmol, 17%).

^{1}H NMR (400 MHz, $CDCl_3$): δ (ppm) 9.21 (dd, J = 4.3, 1.8 Hz, 1H, H1), 8.30 (d, J = 8.4 Hz, 1H, H6), 8.25 (dd, J = 8.1, 1.8 Hz, 1H, H3), 8.06 (d, J = 8.4 Hz, 1H, H7), 8.02 (d, J = 1.8 Hz, 2H, H8), 7.84 (d, J = 8.7 Hz, 1H, H5), 7.77 (d, J = 8.7 Hz, 1H, H4), 7.63 (dd, J = 8.1, 4.4 Hz, 1H, H2), 7.56 (t, J = 1.8 Hz, 1H, H9), 1.44 (s, 18H, H10).

^{13}C NMR (101 MHz, $CDCl_3$): δ (ppm) 159.7, 151.2, 150.4, 146.6, 146.2, 139.9, 136.7, 136.0, 129.1, 127.5, 126.5, 126.2, 123.6, 123.0, 122.7, 121.8, 35.2, 31.7

HRMS (ESI+): found $[M + H]^+$, m/z($C_{26}H_{28}N_2^+$) calculated 369.2325, found 369.2325, δ = 0.0 ppm**2-(4-methoxyphenyl)-1,10-phenanthroline**

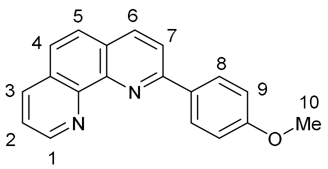

Prepared according to the **GP6** using 2-chloro-1,10-phenanthroline (322 mg, 1.5 mmol, 1.0 equiv.) and 4-methoxyphenyl boronic acid (296 mg, 2.0 mmol, 1.3 equiv.) as starting materials. Purification by silica gel chromatography (Hex:EtOAc – 3:2 to 1:4) yielded the target compound as a yellow powder 385 mg, 1.3 mmol, 90%).

[1]**H NMR** (500 MHz, $CDCl_3$): δ (ppm) 9.23 (dd, J = 4.3, 1.8 Hz, 1H, H1), 8.38 – 8.29 (m, 2H, H8), 8.28 – 8.20 (m, 2H, H3, H6), 8.06 (d, J = 8.4 Hz, 1H, H7), 7.80 (d, J = 8.7 Hz, 1H, H5), 7.74 (d, J = 8.7 Hz, 1H, H4), 7.62 (dd, J = 8.0, 4.3 Hz, 1H, H2), 7.07 (d, J = 8.8 Hz, 2H, H9), 3.90 (s, 3H, H10).

[13]**C NMR** (126 MHz, $CDCl_3$): δ (ppm) 161.0, 157.3, 150.5, 146.6, 146.2, 136.8, 136.2, 132.4, 129.4, 129.2, 127.3, 126.5, 125.9, 122.9, 120.2, 114.3, 55.5.

The NMR data is in agreement with that reported in the literature.[31]

Synthesis of the Starting Materials

2,2,2-trifluoro-*N*-phenylacetamide (1c)

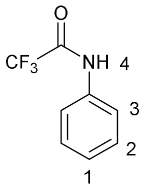

Prepared according to the **GP7** using aniline (5.0 mL, 55.0 mmol, 1.1 equiv.), TFAA (7.0 mL, 50.0 mmol, 1.0 equiv.), and TEA (8.4 mL, 60.0 mmol, 1.2 equiv.). Washes with PE yielded the compound as white crystals (6.48 g, 34.3 mmol, 69%).

^{1}H NMR (400 MHz, $CDCl_3$) δ (ppm) 8.13 (br s, 1H, H4), 7.58 (d, J = 8.0 Hz, 2H, H3), 7.41 (t, J = 8.0 Hz, 2H, H2), 7.31 – 7.21 (m, 1H, H1).

^{13}C NMR (176 MHz, $CDCl_3$) δ (ppm) 155.1 (q, J = 37.3 Hz), 135.2, 129.5, 126.6, 120.8, 115.9 (q, J = 288.6 Hz).

^{19}F NMR (376 MHz, $CDCl_3$) δ (ppm) −75.8

The NMR data is in agreement with that reported in the literature.[32]

2,2,3,3,3-pentafluoro-*N*-phenylpropanamide (1d)

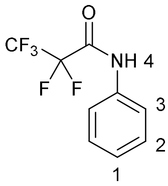

Prepared according to the **GP7** using aniline (0.91 mL, 10.0 mmol, 1.1 equiv.), pentafluoropropionic anhydride (1.8 mL, 9.0 mmol, 1.0 equiv.), and TEA (1.6 mL, 11 mmol, 1.2 equiv.). Washes with PE yielded the compound as white needles (730 mg, 3.1 mmol, 31%).

^{1}H NMR (400 MHz, $CDCl_3$) δ (ppm) 7.93 (br s, 1H, H4), 7.61 – 7.52 (m, 2H, H3), 7.46 – 7.36 (m, 2H, H2), 7.30 – 7.21 (m, 1H, H1).

^{13}C NMR (176 MHz, $CDCl_3$) δ (ppm) 155.5 (t, J = 25.6 Hz), 135.2, 12954, 126.7, 120.7, 118.0 (qt, J = 286.7, 34.6 Hz), 106.9 (tq, J = 267.1, 38.9 Hz).

^{19}F NMR (376 MHz, $CDCl_3$) δ (ppm) −82.6 (t, J = 1.5 Hz, 3F), −122.5 (q, J = 1.7 Hz, 2F)

The NMR data is in agreement with that reported in the literature.[33]

2,2,3,3,4,4,4-heptafluoro-*N*-phenylbutanamide (1e)

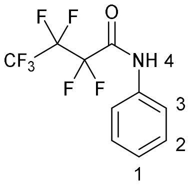

Prepared according to the **GP7** using aniline (0.20 mL, 2.2 mmol, 1.1 equiv.), heptafluorobutyric anhydride (0.49 mL, 2.0 mmol, 1.0 equiv.), and TEA (0.34 mL, 2.4 mmol, 1.2 equiv.). Washes with PE yielded the compound as white crystals (513 mg, 1.8 mmol, 89%).

^{1}H NMR (400 MHz, $CDCl_3$) δ (ppm) 7.88 (br s, 1H, H4), 7.57 (d, J = 8.4 Hz, 2H, H3), 7.41 (t, J = 7.7 Hz, 2H, H2), 7.29 – 7.21 (m, 1H, H1).

^{13}C NMR (176 MHz, $CDCl_3$) δ (ppm) 155.3 (t, J = 25.9 Hz), 135.2, 129.5, 126.7, 120.7, 117.6 (qt, J = 287.6, 33.8 Hz), 110.46 – 105.95 (m, 2C).

^{19}F NMR (376 MHz, $CDCl_3$) δ (ppm) −80.51 (t, J = 8.8 Hz, 3F, CF_3), −120.31 (app. qd, J = 8.8, 2.1 Hz, 2F, CF_2), −126.75 (app. s, 2F, CF_2)

The NMR data is in agreement with that reported in the literature.[34]

2,3,4,5,6-pentafluoro-*N*-phenylbenzamide (1f)

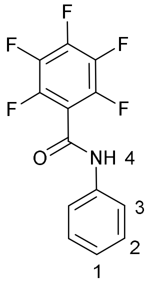

Prepared according to the **GP7** using aniline (0.20 mL, 2.2 mmol, 1.3 equiv.), pentafluorobenzoyl chloride (0.25 mL, 1.7 mmol, 1.0 equiv.), and TEA (0.34 mL, 2.4 mmol, 1.4 equiv.). Washes with PE yielded the compound as white crystals (404, 1.4 mmol, 82%).

^{1}H NMR (400 MHz, $CDCl_3$) δ (ppm) 7.66 (br s, 1H, H4), 7.58 (d, J = 7.9 Hz, 2H, H3), 7.43 – 7.35 (t, 2H, H2), 7.22 (t, J = 7.9 Hz, 1H, H1).

^{13}C NMR (176 MHz, CD_3CN) δ (ppm) 156.3, 144.9 (dddt, J = 249.3, 12.3, 8.3, 4.2 Hz), 144.1 – 142.2 (m), 139.4 – 138.0 (m), 138.6, 130.1, 126.2, 120.9, 113.7 – 112.8 (m).

^{19}F NMR (376 MHz, $CDCl_3$) δ (ppm) −139.87 – −140.24 (m, 2F), −149.78 (tt, J = 20.7, 3.3 Hz, 1F), −159.56 (tt, J = 20.7, 5.9 Hz, 2F).

The NMR data is in agreement with that reported in the literature.[35]

2,2,2-trichloro-*N*-phenylacetamide (1g)

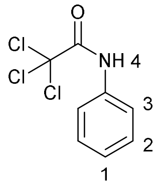

Prepared according to the **GP7** using aniline (0.50 mL, 5.5 mmol, 1.1 equiv.), trichloroacetyl chloride (0.56 mL, 5.0 mmol, 1.0 equiv.), and TEA (0.85 mL, 6.1 mmol, 1.2 equiv.). Washes with PE yielded the compound as white crystals (718 mg, 3.0 mmol, 60%).

^{1}H NMR (500 MHz, $CDCl_3$) δ (ppm) 8.31 (br s, 1H, H4), 7.65 – 7.51 (m,2H, H3), 7.41 (t, J = 8.2 Hz, 2H, H1), 7.29 – 7.19 (m, 1H, H1).

^{13}C NMR (126 MHz, $CDCl_3$) δ (ppm) 159.3, 136.1, 129.5, 126.2, 120.5, 93.0

The NMR data is in agreement with that reported in the literature.[36]

2,2,2-trichloroethyl phenylcarbamate (1h)

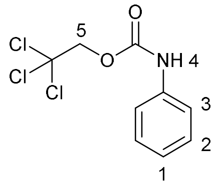

Prepared according to the **GP7** using aniline (0.50 mL, 5.5 mmol, 1.1 equiv.), 3,3,3-trichloropropanoic chloride (0.69 mL, 5.0 mmol, 1.0 equiv.), and TEA (0.85 mL, 6.1 mmol, 1.2 equiv.). Washes with PE yielded the compound as a white powder (808 mg, 3.0 mmol, 60%).

^{1}H NMR (500 MHz, $CDCl_3$) δ (ppm) 7.43 (d, J = 8.0 Hz, 2H, H3), 7.34 (dd, J = 8.6, 7.3 Hz, 2H, H2), 7.12 (t, J = 7.3 Hz, 1H, H1), 6.94 (br s, 1H, H4), 4.83 (s, 2H, H5).

^{13}C NMR (126 MHz, $CDCl_3$) δ (ppm) 151.6, 140.4, 137.1, 129.3, 124.3, 119.0, 95.4, 74.6

The NMR data is in agreement with that reported in the literature.[37]

2,2,2-trifluoro-*N*-(*o*-tolyl)acetamide (1i)

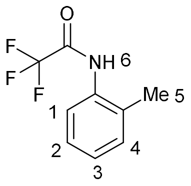

Prepared according to the **GP7** using 2-methylaniline (0.32 mL, 3.0 mmol, 1.1 equiv.), TFAA (0.38 mL, 2.7 mmol, 1.0 equiv.), and TEA (0.46 mL, 3.3 mmol, 1.2 equiv.). Washes with hexane yielded the compound as white crystals (233 mg, 1.1 mmol, 43%).

^{1}H NMR (400 MHz, $CDCl_3$) δ (ppm) 7.77 (br d, J = 7.7 Hz, 2H, H4, H6), 7.37 – 7.13 (m, 3H, H1, H2, H3), 2.31 (s, 3H, H5).

^{13}C NMR (126 MHz, $CDCl_3$) δ (ppm) 155.0 (d, J = 36.9 Hz), 132.8, 130.9, 130.0, 127.1 (d, J = 2.2 Hz), 123.4, 115.9 (d, J = 288.8 Hz), 17.4

^{19}F NMR (376 MHz, $CDCl_3$) δ (ppm) −75.7

The ^{1}H NMR data is in agreement with that reported in the literature.[38]

2,2,2-trifluoro-*N*-(*m*-tolyl)acetamide (1j)

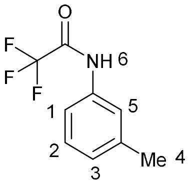

Prepared according to the **GP7** using 3-methylaniline (0.32 mL, 3.0 mmol, 1.1 equiv.), TFAA (0.38 mL, 2.7 mmol, 1.0 equiv.), and TEA (0.46 mL, 3.3 mmol, 1.2 equiv.). Reprecipitation from DCM using hexane yielded the compound as white crystals (307 mg, 1.5 mmol, 51%).

^{1}H NMR (400 MHz, $CDCl_3$) δ (ppm) 7.89 (br s, 1H, H6), 7.39 (s, 1H, H5), 7.34 (d J = 8.0 Hz, 1H, H1), 7.29 – 7.22 (m, 1H, H2), 7.05 (d J = 7.6 Hz, 1H, H3), 2.35 (s, 3H, H4).

^{13}C NMR (126 MHz, $CDCl_3$) δ (ppm) 154.9 (q, J = 37.0 Hz), 139.6, 135.1, 129.3, 127.3, 121.2, 117.7, 115.9 (q, J = 288.8 Hz), 21.6

^{19}F NMR (376 MHz, $CDCl_3$) δ (ppm) −75.8

The NMR data is in agreement with that reported in the literature.[39]

***N*-(2,5-dimethylphenyl)-2,2,2-trifluoroacetamide (1k)**

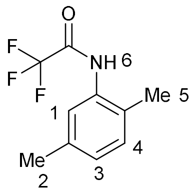

Prepared according to the **GP7** using 2,5-dimethylaniline (1.40 mL, 11.0 mmol, 1.1 equiv.), TFAA (1.40 mL, 10.0 mmol, 1.0 equiv.), and TEA (1.70 mL, 12.0 mmol, 1.2 equiv.). Purification by silica gel chromatography (Hex:EtOAc – 95:5) yielded the target compound as pink crystals (1.78 g, 8.2 mmol, 82%).

^{1}H NMR (500 MHz, $CDCl_3$) δ (ppm) 7.68 (br s, 1H, H6), 7.63 (s, 1H, H1), 7.15 (d, J = 7.7 Hz, 1H, H4), 7.03 (d, J = 8.0 Hz, 1H, H3), 2.37 (s, 3H, H2), 2.27 (s, 3H, H5).

^{13}C NMR (126 MHz, $CDCl_3$) δ (ppm) 155.1 (q, J = 36.6 Hz), 137.2, 132.7, 130.8, 128.0, 126.8, 116.1 (q, J = 288.9 Hz), 21.2, 17.1

^{19}F NMR (376 MHz, $CDCl_3$) δ (ppm) −75.7

2,2,2-trifluoro-*N*-(5,6,7,8-tetrahydronaphthalen-1-yl)acetamide (1l)

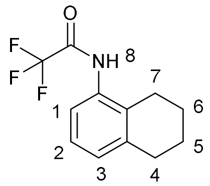

Prepared according to the **GP7** using 5,6,7,8-tetrahydronaphthalen-1-amine (736 mg, 5.0 mmol, 1.1 equiv.), TFAA (0.63 mL, 4.5 mmol, 1.0 equiv.), and TEA (0.77 mL, 5.5 mmol, 1.2 equiv.). Washes with PE yielded the target compound as a white powder (754 mg, 3.1 mmol, 69%).

^{1}H NMR (400 MHz, $CDCl_3$) δ (ppm) 7.80 – 7.55 (br m, 2H, H1, H8), 7.17 (t, J = 7.8 Hz, 1H, H2), 7.03 (dd, J = 7.7, 1.2 Hz, 1H, H3), 2.80 (t, J = 6.2 Hz, 2H, H4), 2.60 (t, J = 6.3 Hz, 2H, H7), 1.94 – 1.83 (m, 2H, H5), 1.83 – 1.74 (m, 2H, H6).

^{13}C NMR (126 MHz, $CDCl_3$) δ (ppm) 155.26 (q, J = 36.8 Hz), 138.84, 132.58, 129.17, 128.23, 126.20, 120.77, 116.09 (q, J = 288.9 Hz), 29.78, 24.29, 22.72, 22.47.

^{19}F NMR (376 MHz, $CDCl_3$) δ (ppm) −75.7

HRMS (ESI+): found $[M + H]^+$, m/z($C_{12}H_{12}F_3NO^+$) calculated 244.0944, found 294.0942, δ = −0.7 ppm

2,2,2-trifluoro-*N*-(2-isopropylphenyl)acetamide (1m)

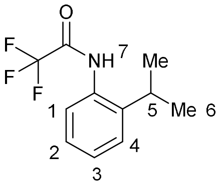

Prepared according to the **GP7** using 2-*iso*-propylaniline (1.49 g, 11.0 mmol, 1.1 equiv.), TFAA (1.40 mL, 10.0 mmol, 1.0 equiv.), and TEA (1.70 mL, 12.0 mmol, 1.2 equiv.). Reprecipitation from DCM using hexane yielded the compound as white crystals (796 mg, 3.4 mmol, 34%).

^{1}H NMR (400 MHz, $CDCl_3$) δ (ppm) 7.77 (br s, 1H, H7), 7.69 (dd, J = 7.3, 2.0 Hz, 1H, H4), 7.41 – 7.17 (m, 3H, H1, H2, H3), 2.98 (sept, J = 6.8 Hz, 1H, H5), 1.28 (d, J = 6.8 Hz, 6H, H6).

^{13}C NMR (126 MHz, $CDCl_3$) δ (ppm) 155.7 (q, J = 36.7 Hz), 141.3, 131.3, 128.0, 126.9, 126.3, 124.7, 116.1 (q, J = 288.9 Hz), 28.4, 23.1

^{19}F NMR (376 MHz, $CDCl_3$) δ (ppm) −75.7

The NMR data is in agreement with that reported in the literature.[40]

2,2,2-trifluoro-*N*-(3-fluorophenyl)acetamide (1n)

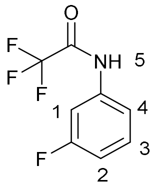

Prepared according to the **GP7** using 3-fluoroaniline (1.10 mL, 11.0 mmol, 1.1 equiv.), TFAA (1.40 mL, 10.0 mmol, 1.0 equiv.), and TEA (1.70 mL, 12.0 mmol, 1.2 equiv.). Washes with PE yielded the target compound as a white powder (1.25 g, 6.0 mmol, 60%).

^{1}H NMR (400 MHz, $CDCl_3$) δ (ppm) 7.92 (br s, 1H, H5), 7.51 (app. dt, J = 10.2, 2.3 Hz, 1H, H1), 7.36 (td, J = 8.2, 6.2 Hz, 1H, H3), 7.29 – 7.20 (m, 1H, H4), 6.96 (tdd, J = 8.3, 2.4, 1.0 Hz, 1H, H2).

^{13}C NMR (126, MHz, $CDCl_3$) δ (ppm) 163.0 (d, J = 246.7 Hz), 155.4 (q, J = 37.7 Hz), 136.6 (d, J = 10.6 Hz), 130.7 (d, J = 9.2 Hz), 117.5 – 115.2 (m), 115.7 (q, J = 288.9 Hz), 113.5 (d, J = 21.3 Hz), 108.5 (d, J = 26.6 Hz).

^{19}F NMR (376 MHz, $CDCl_3$) δ (ppm) −75.8 (s, 3F), −110.1 (s, 1F)

The NMR data is in agreement with that reported in the literature.[41]

***N*-(3-chlorophenyl)-2,2,2-trifluoroacetamide (1o)**

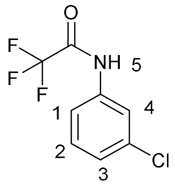

Prepared according to the **GP7** using 3-chloroaniline (0.32 mL, 3.0 mmol, 1.1 equiv.), TFAA (0.38 mL, 2.7 mmol, 1.0 equiv.), and TEA (0.46 mL, 3.3 mmol, 1.2 equiv.). Reprecipitation from DCM using hexane yielded the compound as white crystals (178 mg, 0.8 mmol, 32%).

^{1}H NMR (500 MHz, $CDCl_3$) δ (ppm) 7.95 (br s, 1H, H5), 7.70 (t, J = 2.1 Hz, 1H, H4), 7.45 (d, J = 8.3 Hz, 1H, H1), 7.35 (app. t, J = 8.1 Hz, 1H, H2), 7.25 (d, J = 8.1, 1.3 Hz, 1H, H3).

^{13}C NMR (126 MHz, $CDCl_3$) δ (ppm) 155.0 (q, J = 38.0 Hz), 136.3, 135.3, 130.5, 126.7, 120.9, 118.67, 115.7 (q, J = 288.9 Hz).

^{19}F NMR (376 MHz, $CDCl_3$) δ (ppm) −76.31 (s, 3F), −120.30 (ddd, J = 8.9, 6.6, 4.2 Hz, 1F).

The NMR data is in agreement with that reported in the literature.[41]

***N*-(3-bromophenyl)-2,2,2-trifluoroacetamide (1p)**

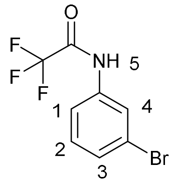

Prepared according to the **GP7** using 3-bromoaniline (1.10 mL, 11.0 mmol, 1.1 equiv.), TFAA (1.40 mL, 10.0 mmol, 1.0 equiv.), and TEA (1.70 mL, 12.0 mmol, 1.2 equiv.). Washes with PE yielded the target compound as off-white crystals (1.92 g, 5.2 mmol, 52%).

^{1}H NMR (500 MHz, $CDCl_3$) δ (ppm) 8.19 (br s, 1H, H5), 7.81 (app. t, J = 2.0 Hz, 1H, H4), 7.50 (ddd, J = 8.2, 2.2, 1.0 Hz, 1H, H1), 7.38 (ddd, J = 8.0, 1.9, 1.0 Hz, 1H, H3), 7.26 (app. t, J = 8.1 Hz, 1H, H2).

^{13}C NMR (126 MHz, $CDCl_3$) δ (ppm) 155.22 (q, J = 37.8 Hz), 136.31, 130.75, 129.67, 123.81, 123.01, 119.31, 115.67 (q, J = 288.5 Hz).

^{19}F NMR (376 MHz, $CDCl_3$) δ (ppm) −75.7

The NMR data is in agreement with that reported in the literature.[42]

***N*-(3,5-dichlorophenyl)-2,2,2-trifluoroacetamide (1q)**

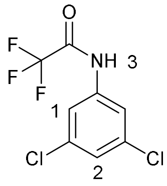

Prepared according to the **GP7** using 3,5-dichloroaniline (1.78 g, 11.0 mmol, 1.1 equiv.), TFAA (1.40 mL, 10.0 mmol, 1.0 equiv.), and TEA (1.70 mL, 12.0 mmol, 1.2 equiv.). Purification by silica gel chromatography (Hex:EtOAc – 95:5) yielded the target compound as white crystals (2.07 g, 8.0 mmol, 80%).

^{1}H NMR (400 MHz, $CDCl_3$) δ (ppm) 7.90 (br s, 1H, H3), 7.55 (d, J = 1.8 Hz, 2H, H1), 7.30 – 7.18 (m, 1H, H2).

^{13}C NMR (101 MHz, $CDCl_3$) δ (ppm) 155.2 (q, J = 38.2 Hz), 136.8, 135.9, 126.7, 119.1, 115.5 (q, J = 288.6 Hz).

^{19}F NMR (376 MHz, $CDCl_3$) δ (ppm) −75.7

The NMR data is in agreement with that reported in the literature.[43]

***N*-(2-bromo-3-methylphenyl)-2,2,2-trifluoroacetamide (1r)**

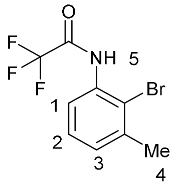

Prepared according to the **GP7** using 2-bromo-3-methylaniline (1.4 mL, 11.0 mmol, 1.1 equiv.), TFAA (1.40 mL, 10.0 mmol, 1.0 equiv.), and TEA (1.70 mL, 12.0 mmol, 1.2 equiv.). Purification by silica gel chromatography (PE:EtOAc – 90:10) yielded the target compound as a yellow powder (1.26 g, 8.9 mmol, 89%).

^{1}H NMR (400 MHz, $CDCl_3$) δ (ppm) 8.58 (br s, 1H, H5), 8.13 (dd, J = 8.1, 1.5 Hz, 1H, H1), 7.27 (app. t, J = 7.9 Hz, 1H, H2), 7.13 (app. ddd, J = 7.6, 1.6, 0.8 Hz, 1H, H3), 2.45 (s, 3H, H4).

^{13}C NMR (101 MHz, $CDCl_3$) δ (ppm) 154.8 (q, J = 37.6 Hz), 139.2, 133.3, 128.3, 128.0, 119.6, 117.0, 112.9 (d, J = 288.8 Hz), 23.8

^{19}F NMR (376 MHz, $CDCl_3$) δ (ppm) −75.9

HRMS (ESI+): found $[M + H]^+$, m/z($C_9H_8BrF_3NO^+$) calculated 281.9736, found 281.9740, $\delta = +1.5$ ppm

2,2,2-trifluoro-*N*-(3-(trifluoromethyl)phenyl)acetamide (1s)

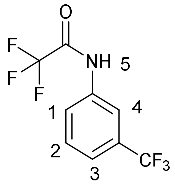

Prepared according to the **GP7** using 3-trifluoromethylaniline (1.77 g, 11.0 mmol, 1.1 equiv.), TFAA (1.40 mL, 10.0 mmol, 1.0 equiv.), and TEA (1.70 mL, 12.0 mmol, 1.2 equiv.). Washes with PE yielded the target compound as off-white crystals (1.62 g, 6.3 mmol, 63%).

^{1}H NMR (500 MHz, $CDCl_3$) δ (ppm) 8.50 (br s, 1H, H5), 7.87 (s, 1H, H4), 7.81 – 7.70 (m, 1H, H1), 7.55 – 7.45 (m, 2H, H2, H3).

^{13}C NMR (126 MHz, $CDCl_3$) δ (ppm) 155.7 (q, J = 38.0 Hz), 135.7, 132.0 (q, J = 33.0 Hz), 130.1, 124.1 (q, J = 1.2 Hz), 123.6 (q, J = 271.8 Hz), 123.3 (q, J = 3.8 Hz), 117.8 (q, J = 4.0 Hz), 115.7 (q, J = 288.2 Hz).

^{19}F NMR (376 MHz, $CDCl_3$) δ (ppm) −63.0 (s, 3F), −75.8 (s, 3F)

The NMR data is in agreement with that reported in the literature.[44]

***N*-(2-benzoylphenyl)-2,2,2-trifluoroacetamide (1t)**

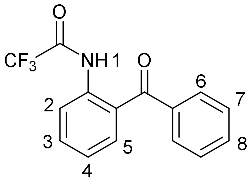

Prepared according to the **GP7** using (2-aminophenyl)(phenyl)methanone (986 mg, 5.5 mmol, 1.1 equiv.), TFAA (0.63 mL, 4.5 mmol, 1.0 equiv.), and TEA (0.77 mL, 5.5 mmol, 1.2 equiv.). Purification by silica gel chromatography (Hex:EtOAc – 95:5 to 90:10) yielded the target compound as white crystals (770 mg, 2.6 mmol, 58%).

^{1}H NMR (400 MHz, $CDCl_3$) δ (ppm) 12.09 (br s, 1H, H1), 8.65 (dd, J = 8.4, 1.1 Hz, 1H, H2), 7.76 – 7.66 (m, 4H, H3, H5, H6), 7.64 (tt, J = 7.3, 1.6 Hz, 1H, H8), 7.52 (td, J = 7.6, 1.5 Hz, 2H, H7), 7.26 (app. td, J = 7.6, 1.2 Hz, 1H, H4).

^{13}**C NMR** (126 MHz, $CDCl_3$) δ (ppm) 199.9, 155.7 (q, J = 37.7 Hz), 138.3, 138.1, 134.8, 134.2, 133.0, 130.1, 128.6, 124.4, 123.9, 121.8, 115.8 (q, J = 288.7 Hz).

^{19}**F NMR** (376 MHz, $CDCl_3$) δ (ppm) −76.2

HRMS (ESI+): found $[M + H]^+$, m/z($C_{15}H_{11}F_3NO_2^+$) calculated 294.0736, found 294.0738, δ = +0.6 ppm

Methyl 3-(2,2,2-trifluoroacetamido)benzoate (1u)

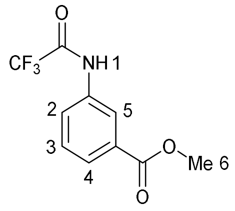

Prepared according to the **GP7** using methyl 3-aminobenzoate (1.4 mL, 11.0 mmol, 1.1 equiv.), TFAA (1.4 mL, 10.0 mmol, 1.0 equiv.), and TEA (1.7 mL, 12.0 mmol, 1.2 equiv.). Purification by silica gel chromatography (Hex:EtOAc – 95:5) yielded the target compound as white crystals (1.95 g, 7.9 mmol, 79%).

^{1}**H NMR** (700 MHz, $CDCl_3$) δ (ppm) 8.53 (br s, 1H, H1), 8.16 (app. t, J = 1.9 Hz, 1H, H5), 7.98 (ddd, J = 8.2, 2.4, 1.1 Hz, 1H, H4), 7.90 (ddd, J = 7.8, 1.6, 1.1 Hz, 1H, H2), 7.47 (app. t, J = 8.0 Hz, 1H, H3), 3.92 (s, 3H, H6).

^{13}**C NMR** (126 MHz, $CDCl_3$) δ (ppm) 166.6, 155.3 (q, J = 37.7 Hz), 135.7, 131.3, 129.7, 127.4, 125.2, 121.8, 115.8 (q, J = 288.6 Hz), 52.6

^{19}**F NMR** (376 MHz, $CDCl_3$) δ (ppm) −75.7

HRMS (ESI+): found $[M + e^-]^-$, m/z($C_{10}H_8F_3NO_3^+$) calculated 247.0456, found 247.0449, δ = −0.8 ppm

***N*-(3-cyanophenyl)-2,2,2-trifluoroacetamide (1v)**

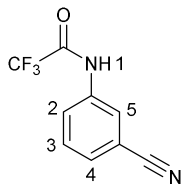

Prepared according to the **GP7** using 3-aminobenzonitrile (1.30 g, 11.0 mmol, 1.1 equiv.), TFAA (1.4 mL, 10.0 mmol, 1.0 equiv.), and TEA (1.7 mL, 12.0 mmol, 1.2 equiv.). Purification by silica gel chromatography (Hex:EtOAc – 90:10 to 80:20) yielded the target compound as yellow crystals (621 mg, 2.9 mmol, 29%).

^{1}H NMR (400 MHz, CD_3CN) *δ* (ppm) 9.41 (br s, 1H, H1), 8.00 (ddd, *J* = 2.1, 1.4, 0.6 Hz, 1H, H5), 7.85 (dt, *J* = 7.5, 2.0 Hz, 1H, H2), 7.64 – 7.49 (m, 2H, H2, H4).

^{13}C NMR (126 MHz, CD_3CN) *δ* (ppm) 156.2 (q, *J* = 37.8 Hz), 137.9, 131.3, 130.5, 126.4, 125.0, 119.0, 116.7 (q, *J* = 287.6 Hz), 113.7

^{19}F NMR (376 MHz, CD_3CN) *δ* (ppm) −77.1

The NMR data is in agreement with that reported in the literature.[45]

2,2,2-trifluoro-*N*-(3-(4,4,5,5-tetramethyl-1,3,2-dioxaborolan-2-yl)phenyl)acetamide (1w)

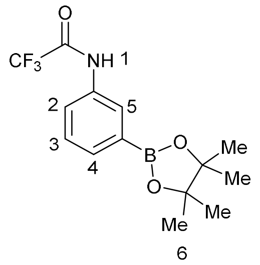

Prepared according to the GP7 using 3-(4,4,5,5-tetramethyl-1,3,2-dioxaborolan-2-yl)aniline (1.095 g, 5.0 mmol, 1.0 equiv.), TFAA (0.76 mL, 5.0 mmol, 1.0 equiv.), and TEA (0.86 mL, 5.5 mmol, 1.1 equiv.). Purification by silica gel chromatography (PE:EtOAc – 90:10) yielded the target compound as an off-white powder (937 mg, 3.0 mmol, 59%).

^{1}H NMR (400 MHz, $CDCl_3$) *δ* (ppm) 8.17 (br s, 1H, H1), 7.84 (ddd, *J* = 8.2, 2.4, 1.1 Hz, 1H, H2), 7.78 (d, *J* = 2.4 Hz, 1H, H5), 7.66 (d, *J* = 7.4 Hz, 1H, H4), 7.38 (app. t, *J* = 7.7 Hz, 1H, H3), 1.33 (s, 12H, H6).

^{13}C NMR (101 MHz, $CDCl_3$) δ (ppm) 147.9 (q, J = 37.3 Hz), 127.6, 125.6, 121.8, 119.6, 116.5, 108.7 (q, J = 288.7 Hz), 77.2, 17.8.

^{19}F NMR (376 MHz, $CDCl_3$) δ (ppm) −75.81

^{11}B NMR (128 MHz, $CDCl_3$) δ (ppm) 30.66

The NMR data is in agreement with that reported in the literature.[46]

3'-amino-[1,1'-biphenyl]-2-carbonitrile

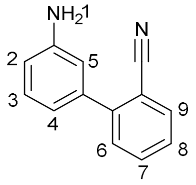

A variant of the protocol by Zhou was adapted.[47] The flask was charged with 3-bromoaniline (344 mg, 2.0 mmol, 1.0 equiv.), 2-cyanophenylboronic acid (294 mg, 2.0 mmol, 1.0 equiv.), $Pd(PPh_3)_2Cl_2$ (70.2 mg, 0.1 mmol, 5 mol%) and flushed with nitrogen/vacuum cycle (3×). Then, the degassed PhMe/EtOH (11.4 mL, 2.8:1 v/v) and sat. aq. Na_2CO_3 (1.0 mL) were added. The mixture was refluxed for 24 h, followed by concentration *in vacuo*. Purification by silica gel chromatography (PE:EtOAc – 8:2 to 6:4) yielded the target compound as brown powder (134 mg, 0.7 mmol, 35%).

^{1}H NMR (400 MHz, CD_3CN) δ (ppm) 7.80 (ddd, J = 7.7, 1.4, 0.6 Hz, 1H, H9), 7.69 (app. td, J = 7.7, 1.4 Hz, 1H, H7), 7.55 – 7.51 (m, 1H, H6), 7.49 (app. td, J = 7.6, 1.3 Hz, 1H, H, H8), 7.22 (app. t, J = 8.0 Hz, 1H, H3), 6.88 – 6.79 (m, 2H, H2, H5), 6.75 (ddd, J = 8.0, 2.3, 1.0 Hz, 1H, H4), 4.22 (br s, 2H, H1).

^{13}C NMR (101 MHz, CD_3CN) δ (ppm) 149.0, 146.7, 140.4, 134.6, 134.0, 130.9, 130.4, 128.7, 119.6, 118.7, 115.7, 115.6, 111.8

The NMR data is in agreement with that reported in the literature.[48]

***N*-(2'-cyano-[1,1'-biphenyl]-3-yl)-2,2,2-trifluoroacetamide (1x)**

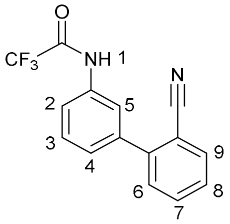

Prepared according to the **GP7** using methyl 3'-amino-[1,1'-biphenyl]-2-carbonitrile (117 mg, 0.6 mmol, 1.0 equiv.), TFAA (0.08 mL, 0.6 mmol, 1.0 equiv.), and TEA (0.09 mL, 0.7 mmol, 1.1 equiv.).

Purification by silica gel chromatography (PE:EtOAc – 9:1 to 7:3) yielded the target compound as a yellow powder (133 mg, 0.46 mmol, 76%).

^{1}H NMR (500 MHz, CD_3CN) δ (ppm) 9.36 (br s, 1H, H1), 7.88 – 7.79 (m, 2H, H7, H9), 7.74 (app. td, J = 7.7, 1.4 Hz, 2H, H6, H8), 7.60 – 7.51 (m, 3H, H3, H4, H5), 7.45 (d, J = 7.7 Hz, 1H, H2).

^{13}C NMR (126 MHz, CD_3CN) δ (ppm) 156.1 (q, J = 37.4 Hz), 145.3, 140.4, 137.4, 134.8, 134.3, 131.1, 130.5, 129.3, 127.4, 122.3, 122.2, 119.3, 116.9 (q, J = 287.8 Hz), 112.0

^{19}F NMR (376 MHz, CD_3CN) δ (ppm) −77.1

HRMS (ESI+, cation): found $[M - H]^-$, m/z($C_{15}H_8F_3N_2O^-$) calculated 289.0594, 289.0594, δ = −0.1 ppm

2,2,2-trifluoro-*N*-(3-(pyrimidin-5-yl)phenyl)acetamide (1y)

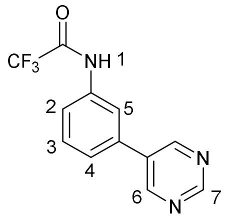

A variant of the protocol by TES Pharma was adapted.[49] The flask was charged with 3-bromoaniline (1.03 g, 6.0 mmol, 1.0 equiv.), pyrimidine-5-boronic acid (966 mg, 7.8 mmol, 1.3 equiv.), K_2CO_3 (3.32 g, 24.0 mmol, 4.0 equiv.), Pd(dppf)Cl_2 (220 mg, 0.3 mmol, 5 mol%) and flushed with nitrogen/vacuum cycle (3×). Then, the degassed dioxane/water (30.0 mL, 3:1 v/v) and the mixture was heated to 80 °C for 4 h. The mixture was allowed to cool to rt and the aqueous phase was extracted with DCM (3×). The combined organics were dried over $MgSO_4$ and concentrated *in vacuo*. The crude mixture was used for the following step.

Following the **GP7**, the crude mixture of methyl 3-(pyrimidin-5-yl)aniline was mixed with TFAA (1.0 mL, 6.6 mmol, 1.1 equiv.), and TEA (1.1 mL, 7.2 mmol, 1.2 equiv.). Purification by silica gel chromatography (PE:EtOAc – 9:1 to 4:6) yielded the target compound as orange powder (1.04 g, 3.9 mmol, 65% over 2 steps).

^{1}H NMR (500 MHz, DMSO-d^6) δ (ppm) 11.42 (br s, 1H, H1), 9.21 (s, 1H, H7), 9.09 (s, 2H, H6), 8.02 (app. t, J = 1.9 Hz, 1H, H5), 7.77 (ddd, J = 8.1, 2.1, 1.0 Hz, 1H, H2), 7.66 (dt, J = 7.8, 1.3 Hz, 1H, H4), 7.58 (app. t, J = 7.9 Hz, 1H, H3).

^{13}C NMR (126 MHz, DMSO-d^6) δ (ppm) 157.4, 154.6, 154.5 (q, J = 37.0 Hz), 137.0, 134.4, 132.7, 129.9, 124.2, 121.4, 119.4, 115.6 (q, J = 288.7 Hz).

^{19}F NMR (376 MHz, DMSO-d^6) δ (ppm) −74.5

HRMS (ESI+, cation): found $[M + H]^+$, m/z($C_{12}H_9F_3N_3O^+$) calculated 268.0692, found 268.0696, δ = +1.4 ppm

Methyl 2-(4-isobutylphenyl)propanoate

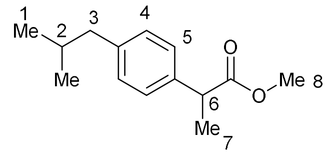

A variant of the protocol by Dzuba was adapted.[50] The 2-(4-isobutylphenyl)propanoic acid (1.03 g, 5.0 mmol, 1.0 equiv.) was dissolved in MeOH (8.5 mL) and conc. H_2SO_4 (0.1 mL) and refluxed for 24 h. The mixture was allowed to cool to rt and was neutralised with sat. aq. $NaHCO_3$. The aqueous phase was extracted with hexane (3×), and the combined organics were dried over $MgSO_4$ and concentrated *in vacuo*. The product was obtained as a colourless oil (1.01 g, 4.6 mmol, 92%).

^{1}H NMR (400 MHz, $CDCl_3$) δ (ppm) 7.20 (d, J = 8.1 Hz, 2H, H5), 7.10 (d, J = 8.1 Hz, 2H, H4), 3.70 (q, J = 7.3 Hz, 1H, H6), 3.66 (s, 3H, H8), 2.44 (d, J = 7.2 Hz, 2H, H3), 1.84 (nonet, J = 6.7 Hz, 1H, H2), 1.49 (d, J = 7.2 Hz, 3H, H6), 0.90 (d, J = 6.6 Hz, 6H, H1).

^{13}C NMR (101 MHz, $CDCl_3$) δ (ppm) 175.4, 140.7, 137.9, 129.5, 127.3, 52.1, 45.2, 45.2, 30.3, 22.6, 18.8

The NMR data is in agreement with that reported in the literature.[50]

Methyl 2-(4-isobutyl-3-(2,2,2-trifluoroacetamido)phenyl)propanoate (1z)

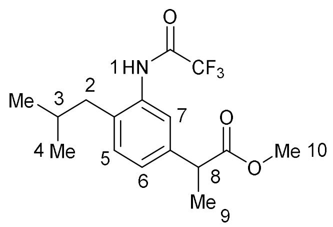

A variant of the protocol by Dzuba was adapted.[50] The methyl 2-(4-isobutylphenyl)propanoate (880 mg, 4.0 mmol, 1.0 equiv.) was dissolved in conc. H_2SO_4 (14.0 mL), followed by addition of the nitrating mixture (conc. HNO_3 – 0.33 mL and conc. H_2SO_4 – 2.6 mL) at 0 °C over 10 mins. The mixture was stirred at 0 °C for 20 mins before being poured onto ice. The aqueous phase was extracted with EtOAc

(3×) and the combined organic were dried over $MgSO_4$ and concentrated *in vacuo*. The crude mixture was used without further purification.

The flask was charged with the crude mixture, Fe powder (894 mg, 16.0 mmol, 4.0 equiv.), NH_4Cl (642 mg, 12.0 mmol, 3.0 equiv.) and dissolved in MeOH/H_2O (11 mL, 10:1, v/v). The suspension was then refluxed for 24 h. The cooled mixture was extracted with EtOAc (2×). Combined organics were washed with H_2O (1×), dried over $MgSO_4$ and concentrated in vacuo. The crude product was obtained as yellow oil (409 mg, 1.7 mmol, 43%).

Following the **GP7,** the crude methyl 2-(3-amino-4-isobutylphenyl)propanoate (409 mg, 1.7 mmol, 1.0 equiv.) was mixed with TFAA (0.27 mL, 1.9 mmol, 1.1 equiv.) and TEA (0.32 mL, 1.9 mmol, 1.1 equiv.). Purification by silica gel chromatography (PE:EtOAc – 9:1) yielded the target compound as orange oil (373 mg, 0.66 mmol, 66%, 17% over 3 steps).

^{1}H NMR (700 MHz, $CDCl_3$) δ (ppm) 7.82 (br s, 1H, H1), 7.74 (d, J = 1.2 Hz, 1H, H7), 7.16 (d, J = 1.2 Hz, 2H, H8, H9), 3.72 (q, J = 7.2 Hz, 1H, H8), 3.66 (s, 3H, H10), 2.43 (d, J = 7.2 Hz, 2H, H2), 1.81 (app. nonet , J = 6.7 Hz, 1H, H3), 1.50 (d, J = 7.2 Hz, 3H, H9), 0.94 (d, J = 6.7 Hz, 6H, H4).

^{13}C NMR (176 MHz, $CDCl_3$) δ (ppm) 174.8, 155.2 (q, J = 36.8 Hz), 139.8, 132.8, 132.4, 131.4, 126.0, 123.1, 116.1 (q, J = 289.9 Hz), 52.3, 45.1, 40.5, 29.5, 22.6, 18.6

^{19}F NMR (376 MHz, $CDCl_3$) δ (ppm) −75.9 (d, J = 1.4 Hz)

HRMS (ESI+): found $[M + Na]^+$, m/z($C_{16}H_{20}F_3NNaO_3^+$) calculated 354.1287, found 354.1291, δ = +0.9 ppm

3-(2,2,2-trifluoroacetamido)benzoic acid

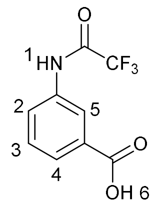

A variant of the protocol by Jiang *et. al.* was adapted.[51] To the stirred solution of 3-aminobenzoic acid (2.74 g, 20.0 mmol, 1.0 equiv.) in EtOA (30 mL, 0.7 M) was added TFAA (3.1 mL, 22.0 mmol, 1.1 equiv.). The mix was heated to 65 °C for 1 h. Then, the suspension was cooled to rt, filtered, and the solids were washed with EtOAc (3×). Drying in vacuo yielded the target compounds as a white powder (2.77 g, 11.9 mmol, 59%).

^{1}H NMR (400 MHz, acetone-*d*^{6}) *δ* (ppm) 11.42 (br s, 1H, H6), 10.38 (s, 1H, H1), 8.43 (app. t, *J* = 1.9 Hz, 1H, H5), 8.00 (ddd, *J* = 8.2, 2.3, 1.1 Hz, 1H, H2), 7.91 (app. dt, *J* = 7.8, 1.4 Hz, 1H, H4), 7.57 (t, *J* = 7.9 Hz, 1H, H3).

^{13}C NMR (176 MHz, acetone-*d*^{6}) *δ* (ppm) 167.0, 155.9 (q, *J* = 37.4 Hz), 137.7, 132.4, 130.2, 127.6, 125.9, 122.8, 116.9 (q, *J* = 287.9 Hz).

^{19}F NMR (376 MHz, acetone-*d*^{6}) *δ* (ppm) −77.3

(*R*)-3-methoxy-2-methyl-3-oxopropyl 3-(2,2,2-trifluoroacetamido)benzoate (1aa)

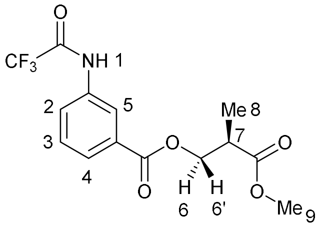

A variant of the protocol by Pal was adapted.[52] The 3-(2,2,2-trifluoroacetamido)benzoic acid (350 mg, 1.5 mmol, 1.0 equiv.), DCC (371 mg, 1.8 mmol, 1.2 equiv.), DMAP (18.3 mg, 0.15 mmol, 10 mol%) were dissolved in DCM (8.0 ml, 0.2 M) and stirred for 10 mins at rt. Then, (*S*)-Rocher ester (500 mg, 1.5 mmol, 1.0 equiv.) was added at once, and the mix was stirred at rt overnight. The suspension was filtered through Celite, rinsed with DCM (2× 10 mL), and the solution was concentrated *in vacuo*. Purification by silica gel chromatography (PE:EtOAc – 9:1 to 3:1) yielded the target compound as colourless oil (364 mg, 1.1 mmol, 73%).

^{1}H NMR (700 MHz, CD_3CN) *δ* (ppm) 9.35 (br s, 1H, H1), 8.25 (app. t, *J* = 1.9 Hz, 1H, H5), 7.86 (ddd, *J* = 8.1, 2.3, 1.1 Hz, 1H, H4), 7.84 (ddd, *J* = 7.8, 1.6, 1.1 Hz, 1H, H2), 7.53 (app. t, *J* = 7.9 Hz, 1H, H3), 4.43 (dd, *J* = 10.8, 7.0 Hz, 1H, H6), 4.40 (dd, *J* = 10.8, 5.6 Hz, 1H, H6'), 3.67 (s, 3H, H9), 2.95 (sextd, *J* = 7.1, 5.6 Hz, 1H, H7), 1.25 (d, *J* = 7.1 Hz, 3H, H8).

^{13}C NMR (176 MHz, CD_3CN) *δ* (ppm) 175.1, 166.2, 156.1 (q, *J* = 37.5 Hz), 137.4, 132.0, 130.5, 127.6, 126.4, 122.6, 116.9 (d, *J* = 287.6 Hz), 67.2, 52.5, 39.8, 14.0

^{19}F NMR (376 MHz, CD_3CN) *δ* (ppm) −75.69 (d, *J* = 1.3 Hz)

HRMS (ESI+): found $[M - H]^-$, m/z($C_{14}H_{13}F_3NO_5^-$) calculated 332.0751, found 332.0750, *δ* = −0.4 ppm

$[\alpha_D]^{25}$ = 14.7 (*c* = 0.75, MeOH)

Characterisation Data for the Products

Fluorination of 2,2,2-trifluoro-*N*-phenylacetamide

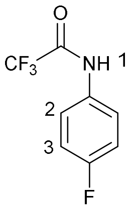

Reaction with Complex 2

Reaction conducted according to the **GP8** using 2,2,2-trifluoro-*N*-phenylacetamide (18.1 mg, 0.1 mmol, 1.0 equiv.) and [Pd(s-tpy)(MeCN)](OTf) (3.1 mg, 0.005 mmol, 5 mol%). The mixture was allowed to cool to rt and filtered through a silica gel plug. The $^{19}F\{^1H\}$ NMR showed 50% NMR yield and *ortho*-:*para*-:dearomatized ratio of 6:41:3. Purification by silica gel chromatography (PE:EtOAc – 100:0 to 95:5) yielded the *para*- isomer as white solid (3.6 mg, 17.4 µmol, 19%).

Reaction with Complex 1

Reaction conducted according to the **GP8** using 2,2,2-trifluoro-*N*-phenylacetamide (18.1 mg, 0.1 mmol, 1.0 equiv.) and [Pd(tpy)(MeCN)](BF_4)$_2$ (2.8 mg, 0.005 mmol, 5 mol%). The $^{19}F\{^1H\}$ NMR showed 47% NMR yield and *ortho*-:*para*-:dearomatized ratio of 17:16:14. Purification by pTLC (PE:EtOAc – 95:5) yielded the *ortho*- isomer as a white solid (0.2 mg, 1.0 µmol, 1%) and the *para*- isomer as a white solid (1.3 mg, 6.3 µmol, 6%).

Assignment of the products: The *para*- isomer was isolated. The positional isomer of the isolated product was assigned by the observed triplet of triplets at −118.4 ppm in ^{19}F NMR. This is further supported by observing only 4 doublets arising from coupling to fluorine in ^{13}C NMR. The *ortho*-isomer was prepared according to the **GP7** using 2-fluoroaniline. The dearomatised product was assigned based on the triplet at −98.9 ppm in the crude ^{19}F NMR, which is in agreement with the previous reports.[53]

***Para*- isomer (3c)**

^{1}H NMR (700 MHz, CD_3CN) δ (ppm) 9.22 (br s, 1H, H1), 7.66 – 7.57 (m, 2H, H2), 7.22 – 7.11 (m, 2H, H3).

^{13}C NMR (176 MHz, CD_3CN) δ (ppm) 160.2 (d, J = 243.3 Hz), 155.0 (q, J = 37.2 Hz), 132.2 (d, J = 2.4 Hz), 123.2 (d, J = 8.2 Hz), 116.3 (q, J = 287.3 Hz), 115.7 (d, J = 23.0 Hz).

$^{19}F\{^1H\}$ NMR (376 MHz, CD_3CN) δ (ppm) −77.1 (s, 3F), −118.4 (tt, J = 8.6, 4.9 Hz, 1F)

HRMS (ESI−): found $[M-H]^-$, m/z($C_8H_4F_4NO^-$) calculated 206.0235, found 206.0238, $\delta = +1.7$ ppm

***Ortho*- isomer (2c)**

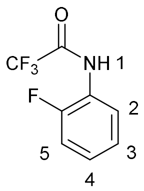

Prepared according to the **GP7** using 2-fluoroaniline (556 mg, 5.0 mmol, 1.1 equiv.), TEA (0.76 mL, 5.5 mmol, 1.2 equiv.), and TFAA (0.63 mL, 4.5 mmol, 1.0 equiv.). The residue was washed with PE to yield the title compound as white crystals (732 mg, 3.5 mmol, 79%).

^{1}H NMR (700 MHz, CD_3CN) δ (ppm) 9.05 (br s, 1H, H1), 7.80 – 7.61 (m, 1H, H2), 7.34 (app. tdd, J = 7.0, 5.1, 1.7 Hz, 1H, H4), 7.29 – 7.20 (m, 2H, H3, H5).

^{13}C NMR (176 MHz, CD_3CN) δ (ppm) 156.4 (q, J = 37.5 Hz), 156.3 (d, J = 248.2 Hz), 129.5 (d, J = 8.3 Hz), 126.8, 125.7 (d, J = 3.7 Hz), 123.7 (d, J = 12.3 Hz), 117.0 (d, J = 19.5 Hz), 116.9 (q, J = 287.4 Hz).

^{19}F NMR{^{1}H} (376 MHz, CD_3CN) δ (ppm) −77.04 (s, 3F), −125.45 (ddd, J = 11.5, 7.8, 5.3 Hz, 1F).

HRMS (ESI−): found $[M-H]^-$, m/z($C_8H_4F_4NO^-$) calculated 206.0235, found 206.0238, $\delta = +1.6$ ppm

Fluorination of 2,2,3,3,3-pentafluoro-*N*-phenylpropanamide

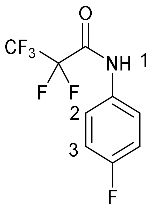

Reaction with Complex 2

Reaction conducted according to the **GP8** using 2,2,3,3,3-pentafluoro-*N*-phenylpropanamide (23.9 mg, 0.1 mmol, 1.0 equiv.) and [Pd(s-tpy)(MeCN)](OTf) (3.1 mg, 0.005 mmol, 5 mol%). The mixture was allowed to cool to rt and filtered through a silica gel plug. The ^{19}F{^{1}H} NMR showed 56% NMR yield and *ortho*-:*para*-:dearomatized ratio of 6:44:6. Purification by pTLC (PE:EtOAc – 95:5) yielded a white crystalline mixture of *para*- isomer (6.0 mg, 23.3 μmol, 23%) and the starting material (3.7 mg, 15% recovery).

Reaction with Complex 1

Reaction conducted according to the **GP8** using 2,2,3,3,3-pentafluoro-*N*-phenylpropanamide (23.9 mg, 0.1 mmol, 1.0 equiv.) and [Pd(tpy)(MeCN)]$(BF_4)_2$ (2.8 mg, 0.005 mmol, 5 mol%). The $^{19}F\{^1H\}$ NMR showed 30% NMR yield and *ortho*-:*para*-:dearomatized ratio of 16:10:4. Purification by pTLC (PE:EtOAc – 95:5) yielded the *para*- isomer in a mixture with the starting material as white solid (3.5 mg, 13.6 μmol, 14%).

Assignment of the products: The *para*- isomer was isolated as a mixture with the starting material. The positional isomer of the isolated product was assigned as described for **3c**. The *ortho*- isomer and the dearomatised compound were assigned by analogy with **3c**.

***Para*- isomer (3d)**

^{1}H NMR (700 MHz, CD_3CN) δ (ppm) 9.30 (br s, 1H, H1), 7.70 – 7.55 (m, 2H, H2), 7.21 – 7.10 (m, 2H, H3).

^{13}C NMR (176 MHz, CD_3CN) δ (ppm) 161.3 (d, J = 243.3 Hz), 156.7 (td, J = 25.9, 6.9 Hz), 133.1 (d, J = 2.6 Hz), 124.5 (d, J = 8.5 Hz), 120.3 – 119.5 (m), 116.7 (d, J = 22.2 Hz), 111.0 – 105.4 (m).

$^{19}F\{^1H\}$ NMR (376 MHz, CD_3CN) δ (ppm) −84.5 (t, J = 1.4 Hz, 3F), −118.1 (tt, J = 8.6, 4.9 Hz, 1F), −123.9 (q, J = 1.4 Hz, 2F).

HRMS: the compound did not ionise

Fluorination of 2,2,3,3,4,4,4-heptafluoro-*N*-phenylbutanamide

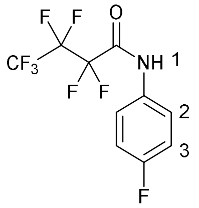

Reaction with Complex 2

Reaction conducted according to the **GP8** using 2,2,3,3,4,4,4-heptafluoro-*N*-phenylbutanamide (28.7 mg, 0.1 mmol, 1.0 equiv.) and [Pd(s-tpy)(MeCN)](OTf) (3.1 mg, 0.005 mmol, 5 mol%). The mixture was allowed to cool to rt and filtered through a silica gel plug. The $^{19}F\{^1H\}$ NMR showed 66% NMR yield and *ortho*-:*para*-:dearomatized ratio of 7:49:10. Purification by pTLC (PE:EtOAc – 9:1) yielded a white crystalline mixture of the *para*- isomer (11.5 mg, 37.4 μmol, 37%) and the starting material (6.8 mg, 24% recovery).

Reaction with Complex 1

Reaction conducted according to the **GP8** using 2,2,3,3,4,4,4-heptafluoro-*N*-phenylbutanamide (28.7 mg, 0.1 mmol, 1.0 equiv.) and [Pd(tpy)(MeCN)](BF_4)$_2$ (2.8 mg, 0.005 mmol, 5 mol%). The ^{19}F{^{1}H} NMR showed 27% NMR yield and *ortho*-:*para*-:dearomatized ratio of 17:10:0.

Assignment of the products: The *para*- isomer was isolated as a mixture with the starting material. The positional isomer of the isolated product was assigned as described for **3c**. The *ortho*- isomer and the dearomatised compound were assigned by analogy with **3c**.

***Para*- isomer (3e)**

^{1}H NMR (500 MHz, CD_3CN) δ (ppm) 9.32 (br s, 1H, H1)*, 7.66 – 7.57 (m, 2H, H2)*, 7.20 – 7.11 (m, 1H, H3).

^{13}C NMR (126 MHz, CD_3CN) δ (ppm) 161.35 (d, J = 243.7 Hz), 156.56 (t, J = 26.1 Hz), 133.05 (d, J = 3.0 Hz), 124.54 (d, J = 8.4 Hz), 118.5(qtm, J = 291.1, 33.4 Hz), 116.74 (d, J = 23.0 Hz), 112.83 – 105.81 (m, 2C)*.

^{19}F{^{1}H} NMR (376 MHz, CD_3CN) δ (ppm) −82.36 (t, J = 8.8 Hz, 3F, CF_3)*, −118.03 (tt, J = 8.5, 4.8 Hz, 1F, ArF), −121.66 (q, J = 8.8 Hz, 2F, CF_2), −128.46 (app. s, 2F, CF_2)*.

HRMS (ESI−): found [M − H]$^-$, m/z($C_{10}H_4F_8NO^-$) calculated 306.0171, found 306.0176, δ = +1.6 ppm

*Note: The peaks labelled with * overlap with the peaks of the starting material.*

Fluorination of 2,3,4,5,6-pentafluoro-*N*-phenylbenzamide

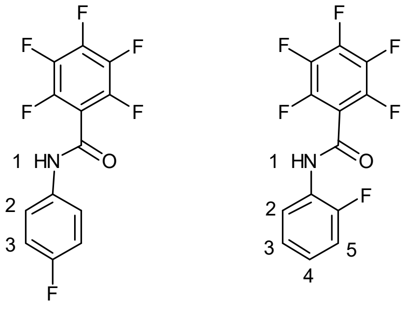

Reaction with Complex 2

Reaction conducted according to the **GP8** using 2,3,4,5,6-pentafluoro-*N*-phenylbenzamide (28.5 mg, 0.1 mmol, 1.0 equiv.) and [Pd(s-tpy)(MeCN)](OTf) (3.1 mg, 0.005 mmol, 5 mol%). The mixture was allowed to cool to rt and filtered through a silica gel plug. The ^{19}F{^{1}H} NMR showed 58% NMR yield and *ortho*-:*para*-:dearomatized ratio of 9:43:6. Purification by pTLC (PE:EtOAc – 9:1) yielded the white crystalline mixture of the *ortho*- isomer (1.6 mg, 4.4 µmol, 5%), *para*- isomer (6.0 mg, 19.7 µmol, 20%) and starting material (11.7 mg, .41% recovery).

Reaction with Complex 1

Reaction conducted according to the **GP8** using 2,3,4,5,6-pentafluoro-*N*-phenylbenzamide (28.5 mg, 0.1 mmol, 1.0 equiv.) and [Pd(tpy)(MeCN)]$(BF_4)_2$ (2.8 mg, 0.005 mmol, 5 mol%). The $^{19}F\{^1H\}$ NMR showed a complex mixture that was unsuitable for further analysis.

Assignment of the products: The *ortho*- and *para*- isomers were isolated as a mixture with the starting material. The positional isomer of the isolated product was assigned as described for **3c**. The dearomatised compound was assigned by analogy with **3c**.

***Para*- isomer (3f)**

^{1}H NMR (500 MHz, CD_3CN) δ (ppm) 9.02 (br s, 1H, H1)*, 7.71 – 7.56 (m, 2H, H2)*, 7.18 – 7.09 (m, 2H, H3).

^{13}C NMR (126 MHz, CD_3CN) δ (ppm) 160.7 (d, J = 242.2 Hz), 146.09 – 143.61 (m)*, 144.1 – 142.2 (m)*, 139.4 – 138.0 (m)*, 134.9 (d, J = 2.9 Hz), 123.0 (d, J = 8.1 Hz), 116.6 (d, J = 22.8 Hz)*, 113.7 – 112.8 (m)*.

^{19}F{^{1}H} NMR (376 MHz, CD_3CN) δ (ppm) −118.61 (tt, J = 8.6, 4.9 Hz, 1F), −142.77 – −143.26 (m, 2F)*, −153.69 (tt, J = 20.0, 2.7 Hz, F)*, −162.64 – −162.99 (m, 2F)*.

HRMS (ESI−): found $[M-H]^-$, m/z($C_{13}H_4F_6NO^-$) calculated 304.0203, found 304.0199, δ = −1.3 ppm

***Ortho*- isomer (2f)**

^{1}H NMR (500 MHz, CD_3CN) δ (ppm) 8.87 (br s, 1H, H1), 8.06 (app. td, J = 8.0, 2.6 Hz, 1H, H2), 7.28 – 7.18 (m, 3H, H3, H4, H5)*.

^{13}C NMR (126 MHz, CD_3CN) δ (ppm) 155.0 (d, J = 245.8 Hz), 146.09 – 143.61 (m)*, 144.1 – 142.2 (m)*, 139.4 – 138.0 (m)*, 127.8 (d, J = 7.7 Hz), 125.9 (d, J = 11.6 Hz), 125.6 (d, J = 3.7 Hz), 124.8 (d, J = 1.2 Hz), 116.6 (d, J = 20.2 Hz)*, 113.7 – 112.8 (m)*.

^{19}F{^{1}H} NMR (376 MHz, CD_3CN) δ (ppm) −127.06 (app. dddt, J = 10.3, 7.5, 5.5, 2.5 Hz, 1F), −142.59 – −143.93 (m, 2F)*, −153.58 (tt, J = 19.9, 2.9 Hz, 1F)*, −162.13* – −164.02* (m, 2F)*.

*Note: The peaks labelled with * overlap with the peaks of the starting material or the other regioisomer.*

Fluorination of 2,2,2-trichloro-*N*-phenylacetamide

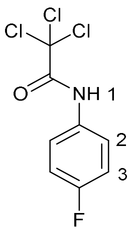

Reaction with Complex 2

Reaction conducted according to the **GP8** using 2,2,2-trichloro-*N*-phenylacetamide (23.8 mg, 0.1 mmol, 1.0 equiv.) and [Pd(s-tpy)(MeCN)](OTf) (3.1 mg, 0.005 mmol, 5 mol%). The mixture was allowed to cool to rt and filtered through a silica gel plug. The $^{19}F\{^1H\}$ NMR showed 42% NMR yield and *ortho*-:*para*-:dearomatized ratio of 6:33:3. Purification by pTLC (PE:EtOAc – 100:0 to 95:5) yielded the *para*- isomer (8.5 mg, 33.1 μmol, 33%) as white crystals.

Reaction with Complex 1

Reaction conducted according to the **GP8** using 2,2,2-trichloro-*N*-phenylacetamide (23.8 mg, 0.1 mmol, 1.0 equiv.) and [Pd(tpy)(MeCN)]$(BF_4)_2$ (2.8 mg, 0.005 mmol, 5 mol%). The $^{19}F\{^1H\}$ NMR showed 35% NMR yield and *ortho*-:*para*-:dearomatized ratio of 17:18:0.

Assignment of the products: The *para*- isomer was isolated. The positional isomer of the isolated product was assigned as described for **3c**. The *ortho*- isomer and the dearomatised compound were assigned by analogy with **3c**.

***Para*- isomer (3g)**

^{1}H NMR (500 MHz, CD_3CN) δ (ppm) 9.12 (br s, 1H, H1), 7.67 – 7.41 (m, 2H, H2), 7.16 (dd, J = 9.1, 8.6 Hz, 2H, H3).

^{13}C NMR (126 MHz, CD_3CN) δ (ppm) 160.2 (d, J = 243.2 Hz), 160.1, 132.8 (d, J = 2.8 Hz), 129.0, 123.8 (d, J = 8.3 Hz), 115.6 (d, J = 22.9 Hz), 92.7

^{19}F NMR (376 MHz, CD_3CN) δ (ppm) −118.7

HRMS: *the compound did not ionise*

Fluorination of 2,2,2-trichloroethyl phenylcarbamate

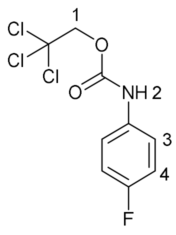

Reaction with Complex 2

Reaction conducted according to the **GP8** using 2,2,2-trichloroethyl phenylcarbamate (23.8 mg, 0.1 mmol, 1.0 equiv.) and [Pd(s-tpy)(MeCN)](OTf) (3.1 mg, 0.005 mmol, 5 mol%). The mixture was allowed to cool to rt and filtered through a silica gel plug. The $^{19}F\{^1H\}$ NMR showed 36% NMR yield

and *ortho*-:*para*-:dearomatized ratio of 5:28:3. Purification by silica gel chromatography (PE:EtOAc – 94:6) yielded the *para*- isomer (3.1 mg, 10.8 µmol, 11%) as white crystals.

Reaction with Complex 1

Reaction conducted according to the **GP8** using 2,2,2-trichloroethyl phenylcarbamate (23.8 mg, 0.1 mmol, 1.0 equiv.) and [Pd(tpy)(MeCN)]$(BF_4)_2$ (2.8 mg, 0.005 mmol, 5 mol%). The $^{19}F\{^1H\}$ NMR showed 28% NMR yield and *ortho*-:*para*-:dearomatized ratio of 10:16:2.

Assignment of the products: The *para*- isomer was isolated. The positional isomer of the isolated product was assigned as described for **3c**. The *ortho*- isomer and the dearomatised compound were assigned by analogy with **3c**.

***Para*- isomer (3h)**

^{1}H NMR (500 MHz, CD_3CN) δ (ppm) 8.14 (br s, 1H, H2), 7.45 (dd, J = 8.8, 4.8 Hz, 2H, H3), 7.20 – 6.92 (m, 2H, H4), 4.86 (s, 2H, H1).

^{13}C NMR (176 MHz, CD_3CN) δ (ppm) 159.9 (d, J = 241.2 Hz), 153.1, 135.3, 121.7 (br app. s), 116.5 (d, J = 23.1 Hz), 96.6, 74.9.

^{19}F{^{1}H} NMR (376 MHz, CD_3CN) δ (ppm) −121.99 (br app. s).

HRMS (ESI−): found $[M-H]^-$, m/z($C_{10}H_8F_4NO^-$) calculated 234.0548, found 234.0545, δ = −1.1 ppm

Fluorination of 2,2,2-trifluoro-*N*-(*o*-tolyl)acetamide

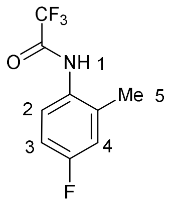

Reaction with Complex 2

Reaction conducted according to the **GP8** using 2,2,2-trifluoro-*N*-(*o*-tolyl)acetamide (20.3 mg, 0.1 mmol, 1.0 equiv.) and [Pd(s-tpy)(MeCN)](OTf) (3.1 mg, 0.005 mmol, 5 mol%). The mixture was allowed to cool to rt and filtered through a silica gel plug. The $^{19}F\{^1H\}$ NMR showed 47% NMR yield and *ortho*-:*para*-:dearomatized ratio of 3:33:11. Purification by pTLC (PE:EtOAc – 95:5) yielded the *para*- isomer (4.2 mg, 19.0 µmol, 19%) as white crystals.

Reaction with Complex 1

Reaction conducted according to the **GP8** using 2,2,2-trifluoro-*N*-(*o*-tolyl)acetamide (20.3 mg, 0.1 mmol, 1.0 equiv.) and [Pd(tpy)(MeCN)]$(BF_4)_2$ (2.8 mg, 0.005 mmol, 5 mol%). The $^{19}F\{^1H\}$ NMR showed 16% NMR yield and *ortho*-:*para*-:dearomatized ratio of 2:12:2.

Assignment of the products: The *para*- isomer was isolated. The positional isomer of the isolated product was assigned on the basis of observing H4 coupling with only one other H atom in COSY, suggesting the large coupling constant coming from $^3J_{H\text{-}F}$ coupling. The *ortho*- isomer was prepared according to the **GP7** using 2-fluoro-6-methylaniline. The dearomatised compound were assigned by analogy with 2,2,2-trifluoro-*N*-phenylacetamide **4c**.

***Para*- isomer (3i)**

^{1}H NMR (400 MHz, CD_3CN) δ (ppm) 8.90 (br s, 1H, H1), 7.34 (dd, J = 8.8, 5.5 Hz, 1H, H2), 7.09 (dd, J = 9.6, 3.0 Hz, 1H, H4), 7.01 (app. td, J = 8.5, 3.0 Hz, 1H, H3), 2.23 (s, 3H, H5).

^{13}C NMR (176 MHz, CD_3CN) δ (ppm) 161.5 (q, J = 244.5 Hz), 155.8 (d, J = 36.4 Hz), 137.1 (d, J = 8.5 Hz), 129.1 (d, J = 3.3 Hz), 128.2 (d, J = 9.1 Hz), 117.1*, 116.2 (d, J = 288.3 Hz), 113.2 (d, J = 23.1 Hz), 16.9.

^{19}F{^{1}H} NMR (376 MHz, CD_3CN) δ (ppm) −77.10 (3F), −117.07 (ddd, J = 9.4, 8.3, 5.4 Hz, 1F).

*Note: The peak labelled with * overlaps with the solvent peak.*

HRMS (ESI−): found $[M - H]^-$, m/z($C_9H_6F_4NO^-$) calculated 220.0391, found 220.0398, δ = +3.1 ppm

***Ortho*- isomer (2i)**

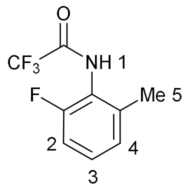

Prepared according to the **GP7** using 2-fluoro-6-methylaniline (375 mg, 3.0 mmol, 1.1 equiv.), TEA (0.56 mL, 3.3 mmol, 1.2 equiv.), and TFAA (0.38 mL, 2.7 mmol, 1.0 equiv.). The residue was washed with PE to yield the title compound as white crystals (507 mg, 2.3 mmol, 85%).

^{1}H NMR (700 MHz, CD_3CN) δ (ppm) 8.93 (br s, 1H, H1), 7.32 (td, J = 8.0, 5.6 Hz, 1H, H3), 7.15 (dq, J = 7.8, 1.0 Hz, 1H, H4), 7.08 (ddd, J = 9.8, 8.1, 1.3 Hz, 1H, H2), 2.26 (s, 3H, H5).

^{13}C NMR (176 MHz, CD_3CN) δ (ppm) 158.8 (d, J = 248.0 Hz), 156.8 (q, J = 37.2 Hz), 139.2, 130.6 (d, J = 8.8 Hz), 127.2 (d, J = 3.3 Hz), 121.6 (d, J = 13.3 Hz), 117.1 (q, J = 287.3 Hz), 114.5 (d, J = 20.4 Hz), 17.7 (d, J = 2.5 Hz).

^{19}F{^{1}H} NMR (376 MHz, CD_3CN) δ (ppm) −76.92 (s, 3F), −123.35 – −123.51 (m, 1F).

HRMS (ESI−): found $[M - H]^-$, m/z($C_9H_6F_4NO^-$) calculated 220.0391, found 220.0386, $\delta = -2.2$ ppm

Fluorination of 2,2,2-trifluoro-*N*-(*m*-tolyl)acetamide

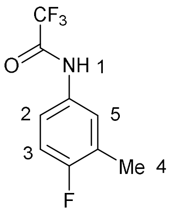

Reaction with Complex 2

Reaction conducted according to the **GP8** using 2,2,2-trifluoro-*N*-(*m*-tolyl)acetamide (20.3 mg, 0.1 mmol, 1.0 equiv.) and [Pd(s-tpy)(MeCN)](OTf) (3.1 mg, 0.005 mmol, 5 mol%). The mixture was allowed to cool to rt and filtered through a silica gel plug. The $^{19}F\{^1H\}$ NMR showed 54% NMR yield and *ortho*-:*para*-:dearomatized ratio of 7:42:5. Purification by pTLC (PE:EtOAc – 95:5) yielded the *para*- isomer (2.3 mg, 10.4 µmol, 10%) as white crystals.

Reaction with Complex 1

Reaction conducted according to the **GP8** using 2,2,2-trifluoro-*N*-(*m*-tolyl)acetamide (20.3 mg, 0.1 mmol, 1.0 equiv.) and $[Pd(tpy)(MeCN)](BF_4)_2$ (2.8 mg, 0.005 mmol, 5 mol%). The $^{19}F\{^1H\}$ NMR showed 46% NMR yield and *ortho*-:*para*-:dearomatized ratio of 25:12:9.

Note: The ortho- isomer reported above is a sum of both possible ortho- isomers (fluorination at H2 and H5).

Assignment of the products: The *para*- isomer was isolated. The positional isomer of the isolated product was assigned by the doublet in ^1H corresponding to the *meta*- methyl group. This is further supported by the H4 and ^{13}C-F coupling in HMBC. The *ortho*- isomers were prepared according to the **GP7** using 2-fluoro-3-methylaniline and 2-fluoro-5-methylaniline. The dearomatised compound were assigned by analogy with **3c**.

***Para*- isomer (3j)**

^1H NMR (700 MHz, CD_3CN) δ (ppm) 9.15 (br s, 1H, H1), 7.49 (dd, J = 7.0, 2.8 Hz, 1H), 7.42 (app. dt, J = 7.8, 3.5 Hz, 1H), 7.08 (app. t, J = 9.1 Hz, 1H), 2.27 (d, J = 2.1 Hz, 3H, H4).

^{13}C NMR (176 MHz, CD_3CN) δ (ppm) 159.8 (d, J = 241.8 Hz), 155.9 (q, J = 37.1 Hz), 132.8 (d, J = 3.7 Hz), 126.7 (d, J = 18.5 Hz), 125.4 (d, J = 6.1 Hz), 121.6 (d, J = 8.2 Hz), 117.8 (q, J = 288.5 Hz), 116.3, 14.7 (d, J = 3.8 Hz).

$^{19}F\{^1H\}$ NMR (376 MHz, CD_3CN) δ (ppm) −76.29 (s, 3F), −121.62 (app. ddt, J = 9.2, 4.5, 2.3 Hz, 1F).

***Ortho*- isomer 1 (2j)**

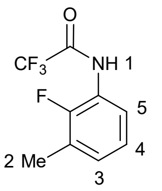

Prepared according to the **GP7** using 2-fluoro-3-methylaniline (375 mg, 3.0 mmol, 1.1 equiv.), TEA (0.56 mL, 3.3 mmol, 1.2 equiv.), and TFAA (0.38 mL, 2.7 mmol, 1.0 equiv.). The residue was washed with PE to yield the title compound as white crystals (467 mg, 2.1 mmol, 78%).

^{1}H NMR (700 MHz, CD_3CN) δ (ppm) 8.99 (br s, 1H, H1), 7.52 (app. t, J = 7.6 Hz, 1H, H5), 7.20 (app. t, J = 7.5 Hz, 1H, H3), 7.12 (app. t, J = 7.8 Hz, 1H, H4), 2.30 (d, J = 2.1 Hz, 3H, H2).

^{13}C NMR (176 MHz, CD_3CN) δ (ppm) 156.4 (q, J = 37.5 Hz), 154.8 (d, J = 246.5 Hz), 130.8 (d, J = 4.7 Hz), 126.8 (d, J = 15.7 Hz), 125.0 (d, J = 4.5 Hz), 124.0 (d, J = 3.3 Hz), 123.5 (d, J = 12.9 Hz), 116.9 (d, J = 287.5 Hz), 14.5 (d, J = 4.3 Hz).

^{19}F{^{1}H} NMR (376 MHz, CD_3CN) δ (ppm) −77.04 (s, 3F), −130.03 – −130.15 (m, 1F).

HRMS (ESI−): found $[M-H]^-$, m/z($C_9H_6F_4NO^-$) calculated 220.0391, found 220.0384, δ = −3.2 ppm

***Ortho*- isomer 2 (2j')**

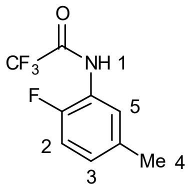

Prepared according to the **GP7** using 2-fluoro-5-methylaniline (375 mg, 3.0 mmol, 1.1 equiv.), TEA (0.56 mL, 3.3 mmol, 1.2 equiv.), and TFAA (0.38 mL, 2.7 mmol, 1.0 equiv.). The residue was washed with PE to yield the title compound as white crystals (295 mg, 1.3 mmol, 49%).

^{1}H NMR (700 MHz, CD_3CN) δ (ppm) 8.98 (br s, 1H, H1), 7.50 (dd, J = 7.5, 2.1 Hz, 1H, H5), 7.20 – 7.01 (m, 2H, H2, H3), 2.33 (d, J = 1.1 Hz, 3H, H4).

^{13}C NMR (176 MHz, CD_3CN) δ (ppm) 156.4 (q, J = 37.5 Hz), 154.5 (d, J = 245.3 Hz), 135.7 (d, J = 3.7 Hz), 129.8 (d, J = 7.5 Hz), 127.0, 123.1 (d, J = 12.5 Hz), 117.2 (q, J = 286.7 Hz), 116.6 (d, J = 19.4 Hz), 20.8.

^{19}F{^{1}H} NMR (376 MHz, CD_3CN) δ (ppm) −77.05, −130.43 (app. dt, J = 8.8, 6.8 Hz).

HRMS (ESI−): found $[M-H]^-$, m/z($C_9H_6F_4NO^-$) calculated 220.0391, found 220.0390, δ = −0.6 ppm

Fluorination of *N*-(2,5-dimethylphenyl)-2,2,2-trifluoroacetamide

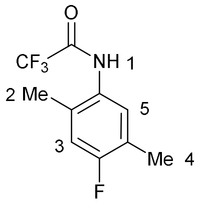

Reaction with Complex 2

Reaction conducted according to the **GP8** using *N*-(2,5-dimethylphenyl)-2,2,2-trifluoroacetamide (21.7 mg, 0.1 mmol, 1.0 equiv.) and [Pd(s-tpy)(MeCN)](OTf) (3.1 mg, 0.005 mmol, 5 mol%). The mixture was allowed to cool to rt and filtered through a silica gel plug. The $^{19}F\{^{1}H\}$ NMR showed 51% NMR yield and *ortho-*:*para-*:dearomatized ratio of 3:39:9. Purification by pTLC (PE:EtOAc – 95:5) yielded the *para-* isomer (5.5 mg, 23.4 μmol, 23%) as white crystals.

Reaction with Complex 1

Reaction conducted according to the **GP8** using *N*-(2,5-dimethylphenyl)-2,2,2-trifluoroacetamide (21.7 mg, 0.1 mmol, 1.0 equiv.) and $[Pd(tpy)(MeCN)](BF_4)_2$ (2.8 mg, 0.005 mmol, 5 mol%). The $^{19}F\{^{1}H\}$ NMR showed 37% NMR yield and *ortho-*:*para-*:dearomatized ratio of 4:5:28.

Assignment of the products: The *para-* isomer was isolated. The positional isomer of the isolated product was assigned by the splitting in ^{1}H corresponding to the *meta-* methyl group. This is further supported by the H4 and ^{13}C-F coupling in HMBC. The *ortho-* isomer was assigned by analogy with the previous examples. The dearomatised compound showed a peak at –98.28 (d, J = 5.4 Hz) ppm, which suggests a formation of *para-* dearomatised compound.

***Para-* isomer (3k)**

^{1}H NMR (700 MHz, CD_3CN) δ (ppm) 8.85 (br s, 1H, H1), 7.19 (d, J = 7.4 Hz, 1H, H5), 7.01 (d, J = 10.3 Hz, 1H, H3), 2.24 – 2.22 (m, 3H, H4), 2.18 (s, 3H, H2).

^{13}C NMR (176 MHz, CD_3CN) δ (ppm) 160.9 (d, J = 243.3 Hz), 156.8 (q, J = 36.9 Hz), 135.0 (d, J = 8.3 Hz), 130.3 (d, J = 5.6 Hz), 126.4 (d, J = 3.4 Hz), 124.0 (d, J = 18.4 Hz), 117.7 (d, J = 23.7 Hz), 117.2 (q, J = 286.5 Hz), 17.4, 14.1 (d, J = 2.7 Hz).

^{19}F{^{1}H} NMR (376 MHz, CD_3CN) δ (ppm) −77.10 (3F), −121.22 (dddq, J = 9.8, 7.5, 1.9 Hz, 1F).

HRMS (ESI−): found $[M-H]^-$, m/z($C_{10}H_8F_4NO^-$) calculated 234.0548, found 234.0545, δ = −1.1 ppm

Fluorination of 2,2,2-trifluoro-*N*-(5,6,7,8-tetrahydronaphthalen-1-yl)acetamide

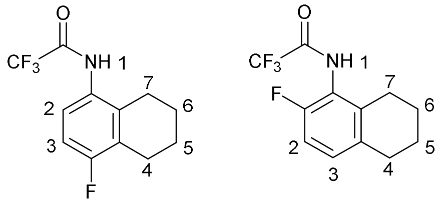

Reaction with Complex 2

Reaction conducted according to the **GP8** using 2,2,2-trifluoro-*N*-(5,6,7,8-tetrahydronaphthalen-1-yl)acetamide (24.3 mg, 0.1 mmol, 1.0 equiv.) and [Pd(s-tpy)(MeCN)](OTf) (3.1 mg, 0.005 mmol, 5 mol%). The mixture was allowed to cool to rt and filtered through silica gel plug. The $^{19}F\{^{1}H\}$ NMR showed 54% NMR yield and *ortho*-:*para*-:dearomatized ratio of 4:41:9. Purification by pTLC (PE:EtOAc – 95:5) yielded a white crystalline mixture of the *para*- isomer (8.6 mg, 32.9 µmol, 33%), *ortho*- isomer (1.5 mg, 5.7 µmol, 6%).

Reaction with Complex 1

Reaction conducted according to the **GP8** using 2,2,2-trifluoro-*N*-(2-isopropylphenyl)acetamide (23.1 mg, 0.1 mmol, 1.0 equiv.) and $[Pd(tpy)(MeCN)](BF_4)_2$ (2.8 mg, 0.005 mmol, 5 mol%). The $^{19}F\{^{1}H\}$ NMR showed 24% NMR yield and *ortho*-:*para*-:dearomatized ratio of 8:11:5.

Assignment of the products: The *para*- isomer and *ortho*- isomer were isolated as a mixture. The positional isomer of the *para*- product was assigned by a doublet in 13C corresponding to the *meta*-benzylic position. This is further supported by the H4/H7 and ^{13}C-F coupling in HMBC. The dearomatised product was assigned by analogy with previous results.

***Para*- isomer (3l)**

^{1}H NMR (700 MHz, CD_3CN) δ (ppm) 8.78 (br s, 1H, H1)*, 7.16 (dd, J = 8.7, 5.2 Hz, 1H, H2)*, 6.95 (app. t, J = 8.9 Hz, 1H, H3), 2.73 (t, J = 5.7 Hz, 2H, H4), 2.64 – 2.56 (m, 2H, H7)*, 1.83 – 1.69 (m, 4H, H5, H6)*.

^{13}C NMR (176 MHz, CD_3CN) δ (ppm) 160.9 (d, J = 243.4 Hz), 156.8 (q, J = 36.6 Hz), 137.1 (d, J = 5.6 Hz), 129.5 (d, J = 2.7 Hz), 126.8 (d, J = 19.1 Hz), 125.9 (d, J = 9.1 Hz), 117.2 (d, J = 287.5 Hz), 113.0 (d, J = 23.5 Hz), 25.5 (d, J = 2.0 Hz), 22.8 (d, J = 3.8 Hz), 22.6, 22.2.

$^{19}F\{^{1}H\}$ NMR (376 MHz, CD_3CN) δ (ppm) −77.07 (s, 3F), −119.75 – −121.14 (m, 1F).

HRMS (ESI−): found $[M - H]^-$, m/z($C_{12}H_{10}F_4NO^-$) calculated 260.0704, found 260.0691, δ = −4.8 ppm

***Ortho*- isomer (3l)**

^{1}H NMR (700 MHz, CD_3CN) δ (ppm) 8.78 (br s, 1H, H1)*, 7.18-7.12 (m, 1H, H3),* 6.99 (dd, J = 9.6, 8.7 Hz, 1H, H2), 2.81 – 2.74 (m, 2H, H4), 2.64 – 2.56 (m, 2H, H7)*, 1.83 – 1.69 (m, 4H, H5, H6)*.

^{13}C NMR (176 MHz, CD_3CN) δ (ppm) 157.5 (d, J = 244.6 Hz), 137.5, 135.2 (d, J = 3.6 Hz), 131.3 (d, J = 8.5 Hz), 120.7 (d, J = 13.2 Hz), 113.8 (d, J = 20.5 Hz), 29.5, 23.3 (d, J = 2.9 Hz), 23.2, 22.9.

^{19}F{^{1}H} NMR (376 MHz, CD_3CN) δ (ppm) –76.89 (s, 3F), –126.90 (dd, J = 9.7, 5.7 Hz, 1F).

*Note: The peaks labelled with * overlap with the peaks of the starting material or the other regioisomer.*

Fluorination of 2,2,2-trifluoro-*N*-(2-isopropylphenyl)acetamide

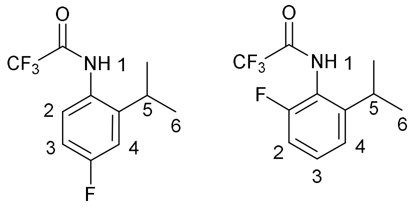

Reaction with Complex 2

Reaction conducted according to the **GP8** using 2,2,2-trifluoro-*N*-(2-isopropylphenyl)acetamide (23.1 mg, 0.1 mmol, 1.0 equiv.) and [Pd(s-tpy)(MeCN)](OTf) (3.1 mg, 0.005 mmol, 5 mol%). The mixture was allowed to cool to rt and filtered through a silica gel plug. The ^{19}F{^{1}H} NMR showed 56% NMR yield and *ortho*-:*para*-:dearomatized ratio of 11:37:8. Purification by pTLC (PE:EtOAc – 95:5) yielded a white crystalline mixture of the *para*- isomer (5.4 mg, 21.7 µmol, 22%), *ortho*- isomer (0.4 mg, 1.6 µmol, 2%) and the starting material (0.6 mg, 2% recovery).

Reaction with Complex 1

Reaction conducted according to the **GP8** using 2,2,2-trifluoro-*N*-(2-isopropylphenyl)acetamide (23.1 mg, 0.1 mmol, 1.0 equiv.) and $[Pd(tpy)(MeCN)](BF_4)_2$ (2.8 mg, 0.005 mmol, 5 mol%). The ^{19}F{^{1}H} NMR showed 24% NMR yield and *ortho*-:*para*-:dearomatized ratio of 8:11:5.

Assignment of the products: The *para*- isomer was isolated as a mixture with the starting material. The positional isomer of the *para*- product was assigned by the peak at .17 (dd, J = 10.3, 2.9 Hz, 1H, H4) in ^{1}H NMR, as its ^{1}H-^{1}H COSY shows coupling only to 1 other H atom, hence, the large coupling constants come from 3 bond coupling with fluorine. The *ortho*- isomer was isolated in low yield; the fluorine position was assigned by analogy and ^{19}F spectrum. The dearomatised compounds were assigned by analogy with 2,2,2-trifluoro-*N*-phenylacetamide.

***Para-* isomer (3m)**

^{1}H NMR (700 MHz, CD_3CN) δ (ppm) 8.91 (br s, 1H, H1), 7.28 (dd, J = 8.8, 5.5 Hz, 1H, H2), 7.17 (dd, J = 10.3, 2.9 Hz, 1H, H4)*, 7.01 (ddd, J = 8.7, 8.0, 2.9 Hz, 1H, H3), 3.04 (septd, J = 6.8, 1.8 Hz, 1H, H5)*, 1.18 (d, J = 6.9 Hz, 6H, H6)*.

^{13}C NMR (176 MHz, CD_3CN) δ (ppm) 163.4 (d, J = 244.5 Hz), 157.6 (q, J = 37.0 Hz), 149.2 (d, J = 7.5 Hz), 131.2 (d, J = 8.9 Hz), 130.5 (d, J = 8.8 Hz), 128.4 (d, J = 2.7 Hz), 117.2 (q, J = 287.9 Hz), 114.3 (d, J = 23.3 Hz), 114.1 (d, J = 23.5 Hz), 29.3, 23.3.

^{19}F{^{1}H} NMR (376 MHz, CD_3CN) δ (ppm) −77.09 (3F, s), −115.31 – −115.49 (1F, m).

HRMS (ESI−): found $[M - H]^-$, m/z($C_{11}H_{10}F_4NO^-$) calculated 248.0704, found 248.0701, δ = −1.4 ppm

***Ortho-* isomer (2m)**

^{19}F{^{1}H} NMR (376 MHz, CD_3CN) δ (ppm) −76.94 (1F, s), −123.01 (3F, ddd, J = 9.8, 5.7, 0.7 Hz).

*Note: The peaks labelled with * overlap with the peaks of the starting material or the other regioisomer.*

Fluorination of 2,2,2-trifluoro-*N*-(3-fluorophenyl)acetamide

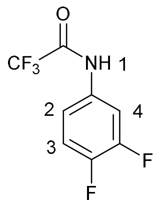

Reaction with Complex 2

Reaction conducted according to the **GP8** using 2,2,2-trifluoro-*N*-(3-fluorophenyl)acetamide (20.7 mg, 0.1 mmol, 1.0 equiv.) and [Pd(s-tpy)(MeCN)](OTf) (3.1 mg, 0.005 mmol, 5 mol%). The mixture was allowed to cool to rt and filtered through a silica gel plug. The ^{19}F{^{1}H} NMR showed 49% NMR yield and *ortho-*:*para-*:dearomatized ratio of 13:28:8. Purification by pTLC (PE:EtOAc – 99:1 and 98:2) yielded a white crystalline mixture of the *para-* isomer (3.0 mg, 13.3 µmol, 13%) and starting material (1.9 mg, 10% recovery).

Reaction with Complex 1

Reaction conducted according to the **GP8** using 2,2,2-trifluoro-*N*-(3-fluorophenyl)acetamide (20.7 mg, 0.1 mmol, 1.0 equiv.) and [Pd(tpy)(MeCN)](BF_4)$_2$ (2.8 mg, 0.005 mmol, 5 mol%). The ^{19}F{^{1}H} NMR showed 58% NMR yield and *ortho-*:*para-*:dearomatized ratio of 25:4:29.

Note: The ortho- isomer reported above is a sum of both possible ortho- isomers (fluorination at H2 and H4).

Assignment of the products: The *para*- isomer was isolated as a mixture with the starting material. The positional isomer of the *para*- product was assigned by the coupling of the ^{13}C(arene)-N peak - 133.7 (d, J = 8.8 Hz), which shows only coupling with the *meta*- fluorine. The *ortho*- isomers were prepared according to **GP7** using 2,3-difluoroaniline and 2,5-difluoroaniline.

***Para*- isomer (3n)**

^{1}H NMR (700 MHz, CD_3CN) δ (ppm) 9.32 (br s, 1H, H1), 7.67 (ddd, J = 12.3, 7.2, 2.6 Hz, 1H, H4), 7.46 – 7.35 (m, 1H, H2)*, 7.30 (app. dt, J = 10.4, 8.9 Hz, 1H, H3).

^{13}C NMR (176 MHz, CD_3CN) δ (ppm) 156.0 (q, J = 38.1 Hz), 150.7 (dd, J = 245.1, 13.4 Hz), 148.8 (dd, J = 245.0, 12.6 Hz), 133.7 (d, J = 8.8 Hz), 118.7 – 118.5 (m, 2C), 117.6 (q, J = 286.6 Hz), 111.6 (d, J = 22.0 Hz).

^{19}F{^{1}H} NMR (376 MHz, $CDCl_3$) δ (ppm) −75.69 (d, J = 1.3 Hz, 3F), −133.65 – −134.27 (m, 1F), −138.55 – −139.19 (m, 1F).

HRMS (ESI−): found $[M-H]^-$, m/z($C_8H_3F_5NO^-$) calculated 234.0548, found 224.0134, δ = −2.8 ppm

*Note: The peaks labelled with * overlap with the peaks of the starting material or the other regioisomer.*

***Ortho*- isomer 1 (2n)**

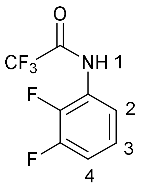

Prepared according to the **GP7** using 2,3-difluoroaniline (646 mg, 5.0 mmol, 1.1 equiv.), TEA (0.93 mL, 5.5 mmol, 1.2 equiv.), and TFAA (0.64 mL, 4.5 mmol, 1.0 equiv.). The residue was washed with PE to yield the title compound as white crystals (500 mg, 2.2 mmol, 49%).

^{1}H NMR (700 MHz, CD_3CN) δ (ppm) 9.17 (br s, 1H, H1), 7.57 – 7.43 (m, 1H, H2), 7.31 – 7.16 (m, 1H, H3, H4).

^{13}C NMR (176 MHz, CD_3CN) δ (ppm) 156.4 (q, J = 38.0 Hz), 151.6 (dd, J = 246.1, 11.1 Hz), 145.0 (dd, J = 250.2, 14.7 Hz), 125.6 (d, J = 7.5 Hz), 125.4 (dd, J = 7.8, 4.9 Hz), 123.0 – 121.3 (m), 117.1 (q, J = 288.8 Hz), 116.6 (d, J = 17.2 Hz).

^{19}F NMR (376 MHz, CD_3CN) δ (ppm) −76.13 (s, 3F), −138.54 – −139.55 (m, 1F), −147.66 – −148.34 (m, 1F).

HRMS (ESI−): found $[M - H]^-$, m/z($C_8H_3F_5NO^-$) calculated 224.0140, found 224.0137, δ = −1.4 ppm

***Ortho-* isomer 2 (2n')**

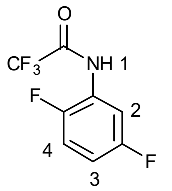

Prepared according to the **GP7** using 2,5-difluoroaniline (646 mg, 5.0 mmol, 1.1 equiv.), TEA (0.93 mL, 5.5 mmol, 1.2 equiv.), and TFAA (0.64 mL, 4.5 mmol, 1.0 equiv.). The residue was washed with PE to yield the title compound as orange crystals (542 mg, 2.4 mmol, 54%).

^{1}H NMR (700 MHz, CD_3CN) δ (ppm) 9.10 (br s, 1H, H1), 7.62 (ddd, J = 9.3, 5.9, 3.1 Hz, 1H, H2), 7.32 – 7.16 (m, 1H, H3), 7.13 – 6.95 (m, 1H, H4).

^{13}C NMR (176 MHz, CD_3CN) δ (ppm) 159.1 (dd, J = 240.8, 2.5 Hz), 156.3 (q, J = 38.2 Hz), 153.3 – 150.8 (m), 124.9 (dd, J = 14.3, 11.6 Hz), 117.9 (ddd, J = 22.2, 9.7, 1.6 Hz), 116.7 (d, J = 287.3 Hz), 115.2 (ddd, J = 24.5, 8.0, 2.4 Hz), 112.9 (dd, J = 28.6, 2.8 Hz).

^{19}F{^{1}H} NMR (376 MHz, CD_3CN) δ (ppm) −76.99 (s, 3F), −139.15 – −140.30 (m, 1F), −148.07 – −149.40 (m, 1F).

HRMS (ESI−): found $[M - H]^-$, m/z($C_8H_3F_5NO^-$) calculated 224.0140, found 224.0138, δ = −1.2 ppm

Fluorination of *N*-(3-chlorophenyl)-2,2,2-trifluoroacetamide

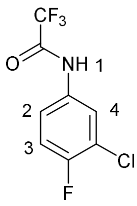

Reaction with Complex 2

Reaction conducted according to the **GP8** using 2,2,2-trifluoro-*N*-(3-chlorophenyl)acetamide (22.4 mg, 0.1 mmol, 1.0 equiv.) and [Pd(s-tpy)(MeCN)](OTf) (3.1 mg, 0.005 mmol, 5 mol%). The mixture was allowed to cool to rt and filtered through a silica gel plug. The ^{19}F{^{1}H} NMR showed 49% NMR yield

and *ortho*-:*para*-:dearomatized ratio of 6:30.13. Purification by pTLC (PE:EtOAc – 97:3) yielded the *para*- isomer (5.1 mg, 21.1 μmol, 21%) as white crystals.

Reaction with Complex 1

Reaction conducted according to the **GP8** using 2,2,2-trifluoro-*N*-(3-chlorophenyl)acetamide (22.4 mg, 0.1 mmol, 1.0 equiv.) and $[Pd(tpy)(MeCN)](BF_4)_2$ (2.8 mg, 0.005 mmol, 5 mol%). The $^{19}F\{^1H\}$ NMR showed 71% NMR yield and *ortho*-:*para*-:dearomatized ratio of 21:5:45.

Note: The ortho- isomer reported above is a sum of both possible ortho- isomers (fluorination at H2 and H4).

Assignment of the products: The *para*- isomer was isolated. The positional isomer of the isolated product was assigned as follows: HMBC coupling between H2 and ^{13}C(arene)-N peak - 133.9 (d, *J* = 3.5 Hz), but no HMBC coupling between H2 and ^{13}C-Cl - 121.5 (d, *J* = 18.7 Hz). Combining this with the observed coupling constant for the ^{13}C(arene)-N peak, this suggests a *para*- fluorinated product. The *ortho*- isomers were prepared according to the **GP7** using 2-fluoro-3-chloroaniline and 2-fluoro-5-chloroaniline. The dearomatised compounds were assigned by analogy with 2,2,2-trifluoro-*N*-phenylacetamide.

***Para*- isomer (3o)**

^{1}H NMR (700 MHz, CD_3CN) δ (ppm) 9.29 (br s, 1H, H1), 7.81 (dd, *J* = 6.6, 2.6 Hz, 1H, H4), 7.53 (ddd, *J* = 9.0, 4.2, 2.6 Hz, 1H, H2), 7.28 (app. t, *J* = 9.0 Hz, 1H, H3).

^{13}C NMR (176 MHz, CD_3CN) δ (ppm) 156.5 (d, *J* = 245.7 Hz), 156.1 (q, *J* = 37.9 Hz), 133.9 (d, *J* = 3.5 Hz), 124.2, 122.6 (d, *J* = 7.2 Hz), 121.5 (d, *J* = 18.7 Hz), 118.0 (d, *J* = 22.6 Hz), 116.8 (q, *J* = 287.5 Hz).

$^{19}F\{^1H\}$ NMR (376 MHz, CD_3CN) δ (ppm) −76.31 (s, 3F), −120.30 (ddd, *J* = 8.9, 6.6, 4.2 Hz, 1F).

HRMS (ESI−): found $[M - H]^-$, m/z($C_8H_3ClF_4NO^-$) calculated 239.9845, found 239.9837, δ = −3.2 ppm

***Ortho*- isomer 1 (2o)**

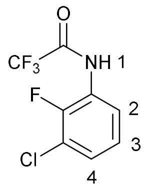

Prepared according to the **GP7** using 3-chloro-2-fluoroaniline (437 mg, 3.0 mmol, 1.1 equiv.), TEA (0.56 mL, 3.3 mmol, 1.2 equiv.), and TFAA (0.38 mL, 2.7 mmol, 1.0 equiv.). The residue was washed with PE to yield the title compound as white crystals (439 mg, 1.8 mmol, 67%).

^{1}H NMR (400 MHz, CD_3CN) δ (ppm) 9.14 (s, 1H, H1), 7.66 (ddd, J = 8.3, 6.7, 1.6 Hz, 1H, H2), 7.42 (ddd, J = 8.4, 6.8, 1.6 Hz, 1H, H4), 7.22 (app. td, J = 8.2, 1.6 Hz, 1H, H3).

^{13}C NMR (101 MHz, CD_3CN) δ (ppm) 156.4 (q, J = 38.9 Hz), 152.1 (d, J = 250.1 Hz), 129.7, 126.0 (d, J = 4.9 Hz), 125.4, 125.2 (d, J = 12.0 Hz), 122.1 (d, J = 16.0 Hz), 116.8 (q, J = 287.0 Hz).

^{19}F{^{1}H} NMR (376 MHz, CD_3CN) δ (ppm) −76.98 (s, 3F), −126.35 (td, J = 6.8, 1.6 Hz, 1F).

HRMS (ESI−): found $[M - H]^-$, m/z($C_7H_3F_4NO^-$) calculated 239.9845, found 239.9841, δ = −1.4 ppm

***Ortho*- isomer 2 (2o')**

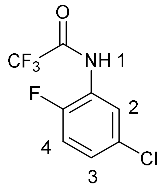

Prepared according to the **GP7** using 5-chloro-2-fluoroaniline (437 mg, 3.0 mmol, 1.1 equiv.), TEA (0.56 mL, 3.3 mmol, 1.2 equiv.), and TFAA (0.38 mL, 2.7 mmol, 1.0 equiv.). The residue was washed with PE to yield the title compound as white crystals (435 mg, 1.8 mmol, 67%).

^{1}H NMR (700 MHz, CD_3CN) δ (ppm) 9.10 (br s, 1H, H1), 7.81 (dd, J = 6.5, 2.6 Hz, 1H, H2), 7.39 – 7.29 (m, 1H, H3), 7.28 – 7.19 (m, 1H, H4).

^{13}C NMR (176 MHz, CD_3CN) δ (ppm) 156.4 (q, J = 38.2 Hz), 154.7 (d, J = 248.3 Hz), 129.9 (d, J = 3.6 Hz), 128.9 (d, J = 8.3 Hz), 126.0, 125.1 (d, J = 13.6 Hz), 116.7 (q, J = 287.3 Hz).

^{19}F{^{1}H} NMR (376 MHz, CD_3CN) δ (ppm) −77.00 (s, 3F), − 127.27 – −127.54 (m, 1F)

HRMS (ESI−): found $[M - H]^-$, m/z($C_7H_3F_4NO^-$) calculated 239.9845, found 239.9841, δ = −1.5 ppm

Fluorination of *N*-(3-bromophenyl)-2,2,2-trifluoroacetamide

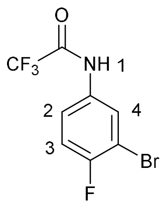

Reaction with Complex 2

Reaction conducted according to the **GP8** using 2,2,2-trifluoro-*N*-(3-bromophenyl)acetamide (26.8 mg, 0.1 mmol, 1.0 equiv.) and [Pd(s-tpy)(MeCN)](OTf) (3.1 mg, 0.005 mmol, 5 mol%). The mixture was allowed to cool to rt and filtered through a silica gel plug. The $^{19}F\{^1H\}$ NMR showed 51% NMR yield and *ortho*-:*para*-:dearomatized ratio of 6:26:29. Purification by pTLC (PE:EtOAc – 97:3) yielded the *para*- isomer (7.3 mg, 25.5 µmol, 26%) as yellow crystals.

Reaction with Complex 1

Reaction conducted according to the **GP8** using 2,2,2-trifluoro-*N*-(3-bromophenyl)acetamide (26.8 mg, 0.1 mmol, 1.0 equiv.) and $[Pd(tpy)(MeCN)](BF_4)_2$ (2.8 mg, 0.005 mmol, 5 mol%). The $^{19}F\{^1H\}$ NMR showed 85% NMR yield and *ortho*-:*para*-:dearomatized ratio 15:13:47.

Note: The ortho- isomer reported above is a sum of both possible ortho- isomers (fluorination at H2 and H4).

Assignment of the products: The *para*- isomer was isolated. The positional isomer of the isolated product was assigned as follows: HMBC coupling between H2 and ^{13}C(arene)-N peak - 134.1 (d, J = 3.3 Hz), but no HMBC coupling between H2 and ^{13}C-Br – 109.3 (d, J = 22.2 Hz). Combining this with the observed coupling constant for the ^{13}C(arene)-N peak, this suggests a *para*- fluorinated product. The *ortho*- isomers and the dearomatised compound were assigned by analogy with the 2,2,2-trifluoro-*N*-(3-chlorophenyl)acetamide results.

***Para*- isomer (3p)**

^{1}H NMR (700 MHz, CD_3CN) δ (ppm) 9.29 (br s, 1H, H1), 7.94 (dd, J = 6.2, 2.6 Hz, 1H, H4), 7.58 (ddd, J = 8.9, 4.2, 2.6 Hz, 1H, H2), 7.26 (app. t, J = 8.6 Hz, 1H, H3).

^{13}C NMR (176 MHz, CD_3CN) δ (ppm) 157.6 (d, J = 244.5 Hz), 156.1 (q, J = 37.7 Hz), 134.1 (d, J = 3.3 Hz), 127.1, 123.3 (d, J = 7.5 Hz), 117.8 (d, J = 23.6 Hz), 116.8 (q, J = 287.7 Hz), 109.3 (d, J = 22.2 Hz).

$^{19}F\{^1H\}$ NMR (376 MHz, CD_3CN) δ (ppm) −77.17 (s, 3F), −113.13 (ddd, J = 8.6, 6.2, 4.3 Hz, 1F).

HRMS (ESI−): found $[M - H]^-$, m/z($C_8H_3BrF_4NO^-$) calculated 283.9340, found 283.9341, δ = +0.6 ppm

Fluorination of *N*-(3,5-dichlorophenyl)-2,2,2-trifluoroacetamide

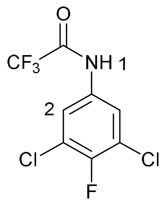

Reaction with Complex 2

Reaction conducted according to the **GP8** using *N*-(3,5-dichlorophenyl)-2,2,2-trifluoroacetamide (25.8 mg, 0.1 mmol, 1.0 equiv.) and [Pd(s-tpy)(MeCN)](OTf) (3.1 mg, 0.005 mmol, 5 mol%). The mixture was allowed to cool to rt and filtered through a silica gel plug. The $^{19}F\{^{1}H\}$ NMR showed 38% NMR yield and *ortho*-:*para*-:dearomatized ratio of 4:15:19. Purification by pTLC (PE:EtOAc – 98:2) yielded the *para*- isomer (4.3 mg, 15.6 μmol, 16%) as while crystals.

Reaction with Complex 1

Reaction conducted according to the **GP8** using *N*-(3,5-dichlorophenyl)-2,2,2-trifluoroacetamide (25.8 mg, 0.1 mmol, 1.0 equiv.) and $[Pd(tpy)(MeCN)](BF_4)_2$ (2.8 mg, 0.005 mmol, 5 mol%). The $^{19}F\{^{1}H\}$ NMR showed 78% NMR yield and *ortho*-:*para*-:dearomatized ratio 24:3:51.

Assignment of the products: The *para*- isomer was isolated. The positional isomer of the isolated product was assigned on the basis of the splitting patterns in ^{1}H and ^{19}F NMR. The *ortho*- isomers and the dearomatised compound were assigned by analogy with the previous results.

***Para*- isomer (3q)**

^{1}H NMR (700 MHz, CD_3CN) δ (ppm) 9.35 (br s, 1H, H1), 7.71 (d, J = 6.0 Hz, 2H, H2).

^{13}C NMR (176 MHz, CD_3CN) δ (ppm) 156.2 (q, J = 38.0 Hz), 152.7 (d, J = 246.9 Hz), 133.9 (d, J = 4.3 Hz), 123.0 (d, J = 18.7 Hz), 122.8, 118.3 (q, J = 287.5 Hz).

$^{19}F\{^{1}H\}$ NMR (376 MHz, CD_3CN) δ (ppm) −76.33 (s, 3F), −121.13 (t, J = 6.0 Hz, 1F).

HRMS (ESI−): found $[M - H]^-$, m/z($C_8H_2Cl_2F_4NO^-$) calculated 273.9455, found 273.9452, δ = −1.1 ppm

Fluorination of *N*-(2-bromo-3-methylphenyl)-2,2,2-trifluoroacetamide

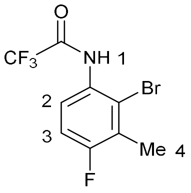

Reaction with Complex 2

Reaction conducted according to the **GP8** using *N*-(2-bromo-3-methylphenyl)-2,2,2-trifluoroacetamide (28.2 mg, 0.1 mmol, 1.0 equiv.) and [Pd(s-tpy)(MeCN)](OTf) (3.1 mg, 0.005 mmol, 5 mol%). The mixture was allowed to cool to rt and filtered through a silica gel plug. The $^{19}F\{^1H\}$ NMR showed 67% NMR yield and *ortho*-:*para*-:dearomatized ratio of 7:42:18. Purification by pTLC (PE:EtOAc – 98:2) yielded a white crystalline mixture of the *para*- isomer (2.6 mg, 8.7 μmol, 9%) and starting material (1.6 mg, 6% recovery).

Reaction with Complex 1

Reaction conducted according to the **GP8** using *N*-(2-bromo-3-methylphenyl)-2,2,2-trifluoroacetamide (28.2 mg, 0.1 mmol, 1.0 equiv.) and $[Pd(tpy)(MeCN)](BF_4)_2$ (2.8 mg, 0.005 mmol, 5 mol%). The $^{19}F\{^1H\}$ NMR showed 53% NMR yield and *ortho*-:*para*-:dearomatized ratio 9:20:24.

Assignment of the products: The *para*- isomer was isolated as a mixture with the starting material. The positional isomer of the isolated product was assigned by the doublet in ^1H corresponding to the *meta*- methyl group. This is further supported by the HMBC coupling between H4 and ^{13}C-F. The *ortho*-isomers and the dearomatised compound were assigned by analogy with previous results.

***Para*- isomer (3r)**

^{1}H NMR (700 MHz, CD_3CN) δ (ppm) 9.00 (br s, 1H, H1)*, 7.48 (dd, J = 8.9, 5.4 Hz, 1H, H2), 7.19 (app. t, J = 9.0 Hz, 1H, H3), 2.38 (d, J = 2.4 Hz, 3H, H4).

^{13}C NMR (176 MHz, CD_3CN) δ (ppm) 160.5 (d, J = 247.3 Hz), 156.6 (q, J = 37.6 Hz)*, 130.6 (d, J = 3.2 Hz), 128.2 (d, J = 20.7 Hz), 126.7 (d, J = 9.2 Hz), 124.2 (d, J = 5.3 Hz), 117.1 (q, J = 287.7 Hz)*, 115.6 (d, J = 24.8 Hz), 15.7 (d, J = 3.9 Hz).

$^{19}F\{^1H\}$ NMR (376 MHz, CD_3CN) δ (ppm) −76.45 (s, 3F), −112.85 (ddd, J = 8.6, 5.4, 2.6 Hz, 1F).

*Note: The peaks labelled with * overlap with the peaks of the starting material.*

HRMS (ESI−): found $[M - H]^-$, m/z($C_9H_5BrF_4NO^-$) calculated 297.9496, found 297.9487, δ = −2.9 ppm

Fluorination of 2,2,2-trifluoro-*N*-(3-(trifluoromethyl)phenyl)acetamide

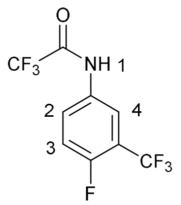

Reaction with Complex 2

Reaction conducted according to the **GP8** using 2,2,2-trifluoro-*N*-(3-(trifluoromethyl)phenyl)acetamide (25.7 mg, 0.1 mmol, 1.0 equiv.) and [Pd(s-tpy)(MeCN)](OTf) (3.1 mg, 0.005 mmol, 5 mol%). The mixture was allowed to cool to rt and filtered through a silica gel plug. The $^{19}F\{^1H\}$ NMR showed 35% NMR yield and *ortho*-:*para*-:dearomatized ratio of 3:28:4. Purification by pTLC (PE:EtOAc – 95:5) yielded the *para*- isomer (5.8 mg, 21.1 μmol, 21%) as white crystals.

Reaction with Complex 1

Reaction conducted according to the **GP8** using 2,2,2-trifluoro-*N*-(3-bromophenyl)acetamide (25.7 mg, 0.1 mmol, 1.0 equiv.) and $[Pd(tpy)(MeCN)](BF_4)_2$ (2.8 mg, 0.005 mmol, 5 mol%). The $^{19}F\{^1H\}$ NMR showed 47% NMR yield and *ortho*-:*para*-:dearomatized ratio 11:22:14.

Note: The ortho- isomer reported above is a sum of both possible ortho- isomers (fluorination at H2 and H4).

Assignment of the products: The *para*- isomer was isolated. The positional isomer of the isolated product was assigned by the doublet in ^{19}F corresponding to the *meta*- CF_3 group. The *ortho*- isomers and the dearomatised compound were assigned by analogy with the 2,2,2-trifluoro-*N*-(3-chlorophenyl)acetamide results.

***Para*- isomer (3s)**

^{1}H NMR (700 MHz, CD_3CN) δ (ppm) 9.41 (br s, 1H, H1), 7.99 (dd, J = 6.4, 2.8 Hz, 1H, H4), 7.89 – 7.86 (m, 1H, H2), 7.37 (app. t, J = 9.7 Hz, 1H, H3).

^{13}C NMR (176 MHz, CD_3CN) δ (ppm) 157.8 (d, J = 253.2 Hz), 156.2 (q, J = 37.9 Hz), 133.6 (d, J = 3.9 Hz), 128.3 (d, J = 9.0 Hz), 123.4 (q, J = 270.6 Hz), 120.8 (q, J = 4.9 Hz), 118.9 (d, J = 22.2 Hz), 118.6 (d, J = 13.7 Hz)*, 116.8 (q, J = 287.6 Hz).

$^{19}F\{^1H\}$ NMR (376 MHz, CD_3CN) δ (ppm) −62.27 (d, J = 12.9 Hz, 3F), −76.36 (s, 3F), −119.64 - −119.719 (m, 1F)

HRMS (ESI−): found $[M-H]^-$, m/z($C_9H_3F_7NO^-$) calculated 273.9452, found 239.9837, δ = −1.1 ppm

**Note, this peak overlaps with the CD_3CN peak.*

Fluorination of *N*-(2-benzoylphenyl)-2,2,2-trifluoroacetamide

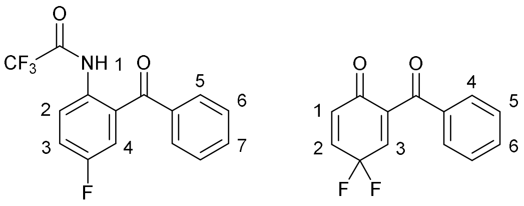

Reaction with Complex 2

Reaction conducted according to the **GP8** using *N*-(2-benzoylphenyl)-2,2,2-trifluoroacetamide (29.3 mg, 0.1 mmol, 1.0 equiv.) and [Pd(s-tpy)(MeCN)](OTf) (3.1 mg, 0.005 mmol, 5 mol%). The mixture was allowed to cool to rt and filtered through a silica gel plug. The $^{19}F\{^1H\}$ NMR showed 49% NMR yield and *ortho*-:*para*-:dearomatized ratio of 4:36:9. Purification by pTLC (PE:EtOAc – 90:10) yielded a yellow crystalline mixture of the *para*- isomer (10.3 mg, 33.1 μmol, 33%).

Reaction with Complex 1

Reaction conducted according to the **GP8** using *N*-(2-benzoylphenyl)-2,2,2-trifluoroacetamide (29.3 mg, 0.1 mmol, 1.0 equiv.) and $[Pd(tpy)(MeCN)](BF_4)_2$ (2.8 mg, 0.005 mmol, 5 mol%). The $^{19}F\{^1H\}$ NMR showed 41% NMR yield and *ortho*-:*para*-:dearomatized ratio 9:23:9.

Assignment of the products: The *para*- isomer was isolated. The positional isomer of the isolated product was assigned by the peak at 7.38 (dd, J = 8.8, 3.0 Hz, 1H, H4) in ^1H NMR, as its ^1H-^1H COSY shows coupling only to 1 other H atom, hence, the large coupling constants come from 3 bond coupling with fluorine. The *ortho*- isomers and the dearomatised compound were assigned by analogy with the 2,2,2-trifluoro-*N*-(3-chlorophenyl)acetamide results.

***Para*- isomer (3t)**

^{1}H NMR (700 MHz, CD_3CN) δ (ppm) 10.76 (br s, 1H, H1), 8.15 (dd, J = 9.1, 4.9 Hz, 1H, H2), 7.76 (dd, J = 8.3, 1.3 Hz, 2H, H5), 7.73 – 7.64 (m, 1H, H7), 7.60 – 7.51 (m, 2H, H6), 7.46 (ddd, J = 9.0, 8.0, 3.0 Hz, 1H, H3), 7.38 (dd, J = 8.8, 3.0 Hz, 1H, H4).

^{13}C NMR (176 MHz, CD_3CN) δ (ppm) 197.6, 160.1 (d, J = 245.8 Hz), 156.1 (q, J = 37.4 Hz), 138.0, 134.4, 133.0 (d, J = 2.5 Hz), 130.9, 129.5, 126.4 (d, J = 7.9 Hz), 121.2, 120.1 (d, J = 24.7 Hz), 116.8 (q, J = 287.6 Hz).

^{19}F{^{1}H} NMR (376 MHz, CD_3CN) δ (ppm) −76.58 (s, 3F), −120.54 – −120.67 (m, 1F).

HRMS (ESI−): found $[M - H]^-$, m/z($C_{15}H_8F_4NO_2^-$) calculated 310.0497, found 310.0486, δ = −3.6 ppm

Dearomatised Product (4t)

^{1}H NMR (700 MHz, CD_3CN) δ (ppm) 7.86 (dd, J = 8.3, 1.3 Hz, 2H, H4), 7.70 (tt, J = 7.4, 1.3 Hz, 1H, H6), 7.54 (app. t, J = 7.8 Hz, 2H, H5), 7.27 – 7.05 (m, 2H, H2, H3), 6.46 (d, J = 10.3 Hz, 1H, H1).

^{13}C NMR (176 MHz, CD_3CN) δ (ppm) 192.5, 183.0, 142.4 (t, J = 8.6 Hz), 138.2 (t, J = 29.1 Hz), 136.5, 136.4 (t, J = 30.3 Hz), 135.5, 132.6 (t, J = 9.1 Hz), 130.4, 129.9, 111.4 (t, J = 227.3 Hz).

^{19}F{^{1}H} NMR (376 MHz, CD_3CN) δ (ppm) –99.66 (t, J = 5.2 Hz)

HRMS: compound did not ionise

Fluorination of methyl 3-(2,2,2-trifluoroacetamido)benzoate

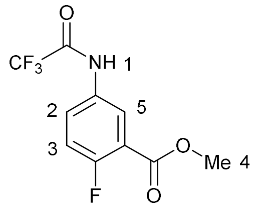

Reaction with Complex 2

Reaction conducted according to the **GP8** using methyl 3-(2,2,2-trifluoroacetamido)benzoate (24.7 mg, 0.1 mmol, 1.0 equiv.) and [Pd(s-tpy)(MeCN)](OTf) (3.1 mg, 0.005 mmol, 5 mol%). The mixture was allowed to cool to rt and filtered through a silica gel plug. The ^{19}F{^{1}H} NMR showed 50% NMR yield and *ortho*-:*para*-:dearomatized ratio of 5:30:15. Purification by pTLC (PE:EtOAc – 95:5 and 90:10) yielded a white crystalline mixture of the *para*- isomer (4.5 mg, 17.0 μmol, 17%).

Reaction with Complex 1

Reaction conducted according to the **GP8** using methyl 3-(2,2,2-trifluoroacetamido)benzoate (24.7 mg, 0.1 mmol, 1.0 equiv.) and [Pd(tpy)(MeCN)]$(BF_4)_2$ (2.8 mg, 0.005 mmol, 5 mol%). The ^{19}F{^{1}H} NMR showed 53% NMR yield and *ortho*-:*para*-:dearomatized ratio 16:5:32.

Note: The ortho- isomer reported above is a sum of both possible ortho- isomers (fluorination at H2 and H5).

Assignment of the products: The *para*- isomer was isolated. The positional isomer of the isolated product was assigned by the doublet in ^{13}C corresponding to the *meta*- carbonyl group. The *ortho*-isomers and the dearomatised compound were assigned by analogy with previous results.

***Para*- isomer (3u)**

^{1}H NMR (700 MHz, CD_3CN) δ (ppm) 9.33 (br s, 1H, H1), 8.18 (dd, J = 6.3, 2.8 Hz, 1H, H5), 7.82 (ddd, J = 9.0, 4.2, 2.9 Hz, 1H, H2), 7.26 (dd, J = 10.4, 9.0 Hz, 1H, H3), 3.89 (s, 3H, H4).

^{13}C NMR (176 MHz, CD_3CN) δ (ppm) 164.8 (d, J = 3.8 Hz), 160.0 (d, J = 257.4 Hz), 156.1 (q, J = 37.2, 36.5 Hz), 133.2 (d, J = 3.8 Hz), 128.4 (d, J = 9.2 Hz), 125.3, 120.0 (d, J = 11.6 Hz), 118.7 (d, J = 24.3 Hz), 116.9 (d, J = 287.7 Hz), 53.2.

^{19}F{^{1}H} NMR (376 MHz, CD_3CN) δ (ppm) −76.32 (s, 3F), −114.29 (ddd, J = 10.5, 6.4, 4.1 Hz, 1F).

HRMS (ESI−): found $[M - H]^-$, m/z($C_{10}H_6F_4NO_3^-$) calculated 264.0289, found 264.0282, δ = −2.9 ppm

Fluorination of *N*-(3-cyanophenyl)-2,2,2-trifluoroacetamide

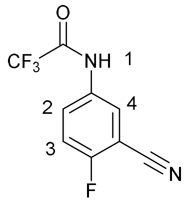

Reaction with Complex 2

Reaction conducted according to the **GP8** using *N*-(3-cyanophenyl)-2,2,2-trifluoroacetamide (21.4 mg, 0.1 mmol, 1.0 equiv.) and [Pd(s-tpy)(MeCN)](OTf) (3.1 mg, 0.005 mmol, 5 mol%). The mixture was allowed to cool to rt and filtered through a silica gel plug. The ^{19}F{^{1}H} NMR showed 31% NMR yield and *ortho*-:*para*-:dearomatized ratio of 2:24:5. Purification by pTLC (PE:acetone – 95:5) yielded a mixture of the *para*- isomer (4.5 mg, 19.2 µmol, 19%), *ortho*- isomer (0.2 mg, 0.9 µmol, 1%) and starting material (4.5 mg, 21% recovery) as an orange powder.

Reaction with Complex 1

Reaction conducted according to the **GP8** using *N*-(3-cyanophenyl)-2,2,2-trifluoroacetamide (21.4 mg, 0.1 mmol, 1.0 equiv.) and $[Pd(tpy)(MeCN)](BF_4)_2$ (2.8 mg, 0.005 mmol, 5 mol%). The ^{19}F{^{1}H} NMR showed 44% NMR yield and *ortho*-:*para*-:dearomatized ratio 10:20:14.

Note: The ortho- isomer reported above is a sum of both possible ortho- isomers (fluorination at H2 and H4).

Assignment of the products: The *para*- isomer was isolated as a mixture with the starting material. The positional isomer of the isolated product was assigned as follows: HMBC coupling between H2 and ^{13}C(arene)-N peak - 134.0, but no HMBC coupling between H2 and ^{13}C-CN – 102.4 (d, J = 16.8

Hz). Combining this with no observable coupling for the ^{13}C(arene)-N peak, this suggests a *para*-fluorinated product. The *ortho*- isomers and the dearomatised compound were assigned by analogy with previous results.

***Para*- isomer (3v)**

^{1}H NMR (700 MHz, CD_3CN) δ (ppm) 9.50 (br s, 1H, H1), 8.10 – 7.97 (m, 1H, H4)*, 7.93 – 7.84 (m, 1H, H2)*, 7.37 (app. t, J = 9.0 Hz, 1H, H3).

^{13}C NMR (176 MHz, CD_3CN) δ (ppm) 161.4 (d, J = 255.2 Hz), 156.3 (q, J = 37.7 Hz), 134.0, 129.4 (d, J = 9.2 Hz), 126.7, 118.2 (d, J = 21.3 Hz),†, 116.7 (q, J = 288.1 Hz), 114.3, 102.4 (d, J = 16.8 Hz).

^{19}F{^{1}H} NMR (376 MHz, CD_3CN) δ (ppm) −77.16 (s, 3F), −113.21 (ddd, J = 8.7, 5.6, 4.7 Hz, 1F).

HRMS (ESI−): found $[M - H]^-$, m/z($C_9H_3F_4N_2O^-$) calculated 231.0187, found 231.0182, δ = −2.2 ppm

*Note: The peaks labelled with * overlap with the peaks of the starting material or the other regioisomer.*
†Peaks overlap with the solvent peaks.

Fluorination of 2,2,2-trifluoro-*N*-(3-(4,4,5,5-tetramethyl-1,3,2-dioxaborolan-2-yl)phenyl)acetamide

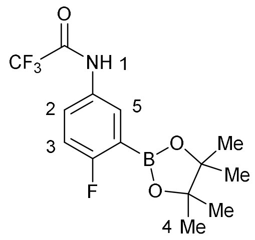

Reaction with Complex 2

Reaction conducted according to the **GP8** using 2,2,2-trifluoro-*N*-(3-(4,4,5,5-tetramethyl-1,3,2-dioxaborolan-2-yl)phenyl)acetamide (31.5 mg, 0.1 mmol, 1.0 equiv.) and [Pd(s-tpy)(MeCN)](OTf) (3.1 mg, 0.005 mmol, 5 mol%). The mixture was allowed to cool to rt and filtered through a silica gel plug. The ^{19}F{^{1}H} NMR showed 54% NMR yield and *ortho*-:*para*-:dearomatized ratio of 4:42:8. Purification by pTLC (PE:EtOAc – 90:10) yielded a white crystalline mixture of the *para*- isomer (7.7 mg, 23.1 μmol, 23%) and starting material (1.5 mg, 5% recovery).

Reaction with Complex 1

Reaction conducted according to the **GP8** using 2,2,2-trifluoro-*N*-(3-(4,4,5,5-tetramethyl-1,3,2-dioxaborolan-2-yl)phenyl)acetamide (31.5 mg, 0.1 mmol, 1.0 equiv.) and [Pd(tpy)(MeCN)]$(BF_4)_2$ (2.8

mg, 0.005 mmol, 5 mol%). The $^{19}F\{^1H\}$ NMR showed 48% NMR yield and *ortho*-:*para*-:dearomatized ratio 14:29:5.

Note: The ortho- isomer reported above is a sum of both possible ortho- isomers (fluorination at H2 and H5).

Assignment of the products: The *para*- isomer was isolated as a mixture with the starting material. The positional isomer of the isolated product was assigned as follows: HMBC coupling between H2 and ^{13}C(arene)-N peak – 132.8 ppm. Combining this with no observable coupling for the ^{13}C(arene)-N peak, this suggests a *para*- fluorinated product, which is analogous to previous examples. The *ortho*-isomers and the dearomatised compound were assigned by analogy with previous results.

***Para*- isomer (3w)**

^{1}H NMR (700 MHz, CD_3CN) δ (ppm) 9.21 (br s, 1H, H1)*, 7.91 (dd, J = 5.1, 2.9 Hz, 1H, H5), 7.71 (ddd, J = 8.9, 4.7, 2.9 Hz, 1H, H2)*, 7.11 (app. t, J = 8.9 Hz, 1H, H3), 1.34 (s, 12H, H4)*.

^{13}C NMR† (176 MHz, CD_3CN) δ (ppm) 165.3 (d, J = 249.5 Hz), 156.0 (q, J = 37.3 Hz), 132.8, 130.1 (d, J = 8.8 Hz), 127.6 (d, J = 9.1 Hz), 117.0 (d, J = 26.0 Hz), 116.9 (q, J = 286.9 Hz), 85.2, 25.1.

$^{19}F\{^1H\}$ NMR (376 MHz, CD_3CN) δ (ppm) −76.31 (s, 3F), −103.89 – −109.47 (m, 1F).

^{11}B NMR (160 MHz, CD_3CN) δ (ppm) 29.03.

HRMS (ESI−): found $[M-H]^-$, m/z($C_{14}H_{15}BF_4NO_3^-$) calculated 332.1087, found 332.1081, δ = −1.7 ppm

*Note: The peaks labelled with * overlap with the peaks of the starting material.*

†The quaternary centre connected to the boronate ester is not visible due to its fast quadrupole relaxation.

Fluorination of *N*-(2'-cyano-[1,1'-biphenyl]-3-yl)-2,2,2-trifluoroacetamide

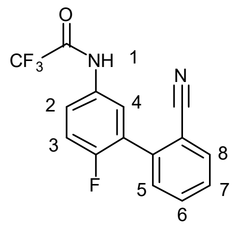

Reaction with Complex 2

Reaction conducted according to the **GP8** using *N*-(2'-cyano-[1,1'-biphenyl]-3-yl)-2,2,2-trifluoroacetamide (29.0 mg, 0.1 mmol, 1.0 equiv.) and [Pd(s-tpy)(MeCN)](OTf) (3.1 mg, 0.005 mmol,

5 mol%). The mixture was allowed to cool to rt and filtered through a silica gel plug. The $^{19}F\{^1H\}$ NMR showed 73% NMR yield and *ortho*-:*para*-:dearomatized ratio of 8:51:14. Purification by pTLC using neutral silica (PE:acetone – 85:15) yielded a white crystalline mixture of the *para*- isomer (4.4 mg, 14.3 µmol, 14%), starting material (3.7 mg, 13% recovery) and unknown impurity (5%).

Reaction with Complex 1

Reaction conducted according to the **GP8** using *N*-(2'-cyano-[1,1'-biphenyl]-3-yl)-2,2,2-trifluoroacetamide (29.0 mg, 0.1 mmol, 1.0 equiv.) and [Pd(tpy)(MeCN)]$(BF_4)_2$ (2.8 mg, 0.005 mmol, 5 mol%). The $^{19}F\{^1H\}$ NMR showed 56% NMR yield and *ortho*-:*para*-:dearomatized ratio 11:21:24.

Note: The ortho- isomer reported above is a sum of both possible ortho- isomers (fluorination at H2 and H4).

Assignment of the products: The *para*- isomer was isolated as a mixture. The positional isomer of the isolated product was assigned as follows: HMBC coupling between H2 and ^{13}C(arene)-N peak – 133.4 (d, J = 2.6 Hz). Combining this with the observed coupling constant for the ^{13}C(arene)-N peak, this suggests a *para*- fluorinated product, which is analogous to previous examples. The *ortho*- isomers and the dearomatised compound were assigned by analogy with previous results.

***Para*- isomer (3x)**

^1H NMR (700 MHz, CD_3CN) δ (ppm) 9.38 (br s, 1H, H1)*, 7.87 (ddd, J = 7.8, 1.4, 0.6 Hz, 1H, H8), 7.79 – 7.73 (m, 2H, H2, H6)*, 7.72 (dd, J = 6.5, 2.7 Hz, 1H, H4), 7.61 (app. td, J = 7.7, 1.2 Hz, 1H, H7), 7.58 – 7.54 (m, 1H, H5)*, 7.33 (app. t, J = 9.2 Hz, 1H, H3).

^{13}C NMR (176 MHz, CD_3CN) δ (ppm) 157.8 (d, J = 245.9 Hz), 156.1 (q, J = 37.4 Hz), 139.4, 134.2 (d, J = 3.7 Hz), 133.4 (d, J = 2.6 Hz), 131.8, 130.0, 129.4, 127.5 (d, J = 16.9 Hz), 125.1, 124.9 (d, J = 8.6 Hz), 118.7, 117.5 (d, J = 23.7 Hz), 117.3 – 115.4 (m), 113.5.

$^{19}F\{^1H\}$ NMR (376 MHz, CD_3CN) δ (ppm) −77.11 (s, 3F), −120.33 (ddd, J = 9.7, 6.6, 4.5 Hz, 1F).

HRMS (ESI−): found $[M - H]^-$, m/z($C_{15}H_7F_4N_2O^-$) calculated 307.0500, found 307.0494, δ = −1.9 ppm

Fluorination of 2,2,2-trifluoro-*N*-(3-(pyrimidin-5-yl)phenyl)acetamide

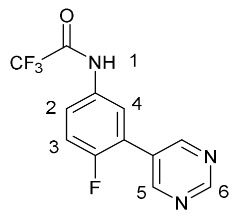

Reaction with Complex 2

Reaction conducted according to the **GP8** using 2,2,2-trifluoro-*N*-(3-(pyrimidin-5-yl)phenyl)acetamide (26.7 mg, 0.1 mmol, 1.0 equiv.) and [Pd(s-tpy)(MeCN)](OTf) (3.1 mg, 0.005 mmol, 5 mol%). The mixture was allowed to cool to rt and filtered through a silica gel plug. The $^{19}F\{^{1}H\}$ NMR showed 42% NMR yield and *ortho*-:*para*-:dearomatized ratio of 5:37:1. Purification by pTLC (PE:acetone – 80.20) yielded the *para*- isomer (4.8 mg, 16.8 μmol, 17%) as brown powder.

Reaction with Complex 1

Reaction conducted according to the **GP8** using 2,2,2-trifluoro-*N*-(3-(pyrimidin-5-yl)phenyl)acetamide (26.7 mg, 0.1 mmol, 1.0 equiv.) and $[Pd(tpy)(MeCN)](BF_4)_2$ (2.8 mg, 0.005 mmol, 5 mol%). The $^{19}F\{^{1}H\}$ NMR showed 31% NMR yield and *ortho*-:*para*-:dearomatized ratio 12:19:0.

Note: The ortho- isomer reported above is a sum of both possible ortho- isomers (fluorination at H2 and H4).

Assignment of the products: The *para*- isomer was isolated. The positional isomer of the isolated product was assigned by the peak 157.3 (d, *J* = 3.6 Hz) in ^{13}C NMR, corresponding to the C-H5. The *ortho*- isomers and the dearomatised compound were assigned by analogy with previous results.

***Para*- isomer (3y)**

^{1}H NMR (700 MHz, CD_3CN) δ (ppm) 9.39 (br s, 1H, H1), 9.18 (s, 1H, H6), 8.94 (d, *J* = 1.4 Hz, 2H, H5), 7.81 (dd, *J* = 6.7, 2.7 Hz, 1H, H4), 7.72 (ddd, *J* = 8.9, 4.3, 2.7 Hz, 1H, H2), 7.34 (dd, *J* = 10.1, 9.0 Hz, 1H, H3).

^{13}C NMR (176 MHz, CD_3CN) δ (ppm) 158.9, 157.9 (d, *J* = 272.8 Hz), 157.3 (d, *J* = 3.6 Hz), 156.1 (q, *J* = 37.8 Hz), 133.9, 130.0, 125.0 (d, *J* = 8.2 Hz), 124.2, 123.8 (d, *J* = 15.6 Hz), 117.9 (d, *J* = 23.8 Hz), 116.9 (q, *J* = 286.7 Hz).

$^{19}F\{^{1}H\}$ NMR (376 MHz, CD_3CN) δ (ppm) −77.15 (s, 3F), −122.87 (app. dt, *J* = 11.0, 5.8 Hz, 1F).

HRMS (ESI−): found $[M - H]^-$, m/z($C_{12}H_6F_4N_3O^-$) calculated 284.0452, found 284.0453, δ = + 0.1 ppm

Fluorination of methyl 2-(4-isobutyl-3-(2,2,2-trifluoroacetamido)phenyl)propanoate

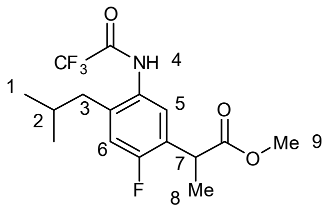

Reaction with Complex 2

Reaction conducted according to the **GP8** using methyl 2-(4-isobutyl-3-(2,2,2-trifluoroacetamido)phenyl)propanoate (33.1 mg, 0.1 mmol, 1.0 equiv.) and [Pd(s-tpy)(MeCN)](OTf) (3.1 mg, 0.005 mmol, 5 mol%). The mixture was allowed to cool to rt and filtered through a silica gel plug. The $^{19}F\{^{1}H\}$ NMR showed 37% NMR yield and *ortho*-:*para*-:dearomatized ratio of 1:31:5. Purification by pTLC (PE:EtOAc – 95:5) yielded a yellow oily mixture of the *para*- isomer (9.8 mg, 28.1 μmol, 28%) and starting material (7.0 mg, 21% recovery).

Reaction with Complex 1

Reaction conducted according to the **GP8** using methyl 2-(4-isobutyl-3-(2,2,2-trifluoroacetamido)phenyl)propanoate (33.1 mg, 0.1 mmol, 1.0 equiv.) and [Pd(tpy)(MeCN)]$(BF_4)_2$ (2.8 mg, 0.005 mmol, 5 mol%). The $^{19}F\{^{1}H\}$ NMR showed 22% NMR yield and *ortho*-:*para*-:dearomatized ratio 2:8:12.

Assignment of the products: The *para*- isomer was isolated as a mixture with the starting material. The positional isomer of the isolated product was assigned by the HMBC between H7 and ^{13}C-F. The *ortho*- isomers and the dearomatised compound were assigned by analogy with previous results.

***Para*- isomer (3z)**

^{1}H NMR (700 MHz, CD_3CN) δ (ppm) 8.88 (br s, 1H, H4), 7.28 – 7.20 (m, 1H, H5)*, 7.05 (d, J = 11.0 Hz, 1H, H6), 3.97 (q, J = 7.2 Hz, 1H, H7), 3.64 (s, 3H, H9), 2.43 (app. t, J = 7.7 Hz, 2H, H3)*, 1.86 – 1.73 (m, 1H, H2)*, 1.45 (d, J = 7.3 Hz, 3H, H8), 0.87 (dd, J = 6.6, 5.6 Hz, 6H, H1)*.

^{13}C NMR (176 MHz, CD_3CN) δ (ppm) 174.7, 160.2 (d, J = 245.8 Hz), 157.0 (q, J = 36.6 Hz)*, 129.8 (d, J = 3.5 Hz), 129.4 (d, J = 4.9 Hz), 128.9 (d, J = 5.2 Hz), 127.4 (d, J = 16.4 Hz), 118.2, 117.2 (q, J = 286.9 Hz)*, 52.8, 40.8, 39.3, 29.9, 22.5, 17.6.

$^{19}F\{^{1}H\}$ NMR (376 MHz, CD_3CN) δ (ppm) −76.30 (s, 3F), −120.19 (dd, J = 11.0, 7.2 Hz, 1F).

*Note: The peaks labelled with * overlap with the peaks of the starting material.*

HRMS (ESI−): found $[M-H]^-$, m/z($C_9H_3F_4N_2O^-$) calculated 348.1228, found 348.1218, δ = −2.9 ppm

Fluorination of *(R)*-3-methoxy-2-methyl-3-oxopropyl 3-(2,2,2-trifluoroacetamido)benzoate

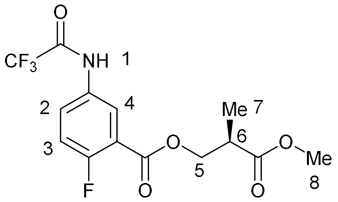

Reaction with Complex 2

Reaction conducted according to the **GP8** using *(R)*-3-methoxy-2-methyl-3-oxopropyl 3-(2,2,2-trifluoroacetamido)benzoate (33.3 mg, 0.1 mmol, 1.0 equiv.) and [Pd(s-tpy)(MeCN)](OTf) (3.1 mg, 0.005 mmol, 5 mol%). The mixture was allowed to cool to rt and filtered through a silica gel plug. The $^{19}F\{^1H\}$ NMR showed 36% NMR yield and *ortho*-:*para*-:dearomatized ratio of 3:30:3 Purification by pTLC (PE:acetone – 85:15) yielded a white crystalline mixture of the *para*- isomer (7.2 mg, 20.5 µmol, 21%), *ortho*- isomer (0.9 mg, 2.6 µmol, 3%), starting material (16.8 mg, 50% recovery) and NFSI (0.9 mg).

Reaction with Complex 1

Reaction conducted according to the **GP8** using methyl 2-(4-isobutyl-3-(2,2,2-trifluoroacetamido)phenyl)propanoate (33.3 mg, 0.1 mmol, 1.0 equiv.) and [Pd(tpy)(MeCN)]$(BF_4)_2$ (2.8 mg, 0.005 mmol, 5 mol%). The $^{19}F\{^1H\}$ NMR showed 68% NMR yield and *ortho*-:*para*-:dearomatized ratio 18:8:42.

Note: The ortho- isomer reported above is a sum of both possible ortho- isomers (fluorination at H2 and H4).

Assignment of the products: The *ortho*- and *para*- isomers were isolated as a mixture with the starting material. The positional isomer of the *para*- product was assigned by the doublet in ^{13}C corresponding to the *meta*- carbonyl group. The dearomatised compound was assigned by analogy with previous results.

***Para*- isomer (3aa)**

^{1}H NMR (700 MHz, CD_3CN) δ (ppm) 9.37 (br s, 1H, H1), 8.15 (dd, J = 6.3, 2.8 Hz, 1H, H4), 7.85 – 7.81 (m, 1H, H2), 7.25 (dd, J = 10.4, 9.0 Hz, 1H, H3), 4.48 – 4.36 (m, 2H, H5), 3.66 (s, 3H, H8), 3.09 – 2.83 (m, 1H, H6), 1.24 (d, J = 7.1 Hz, 3H, H7).

^{13}C NMR (176 MHz, CD_3CN) δ (ppm) 175.0, 164.0 (d, J = 4.1 Hz), 160.0 (q, J = 257.6 Hz)*, 156.1 (q, J = 37.7, 37.2 Hz), 133.2 (d, J = 3.7 Hz), 128.5 (d, J = 9.1 Hz), 125.3 (d, J = 4.9 Hz), 119.8 (d, J = 10.9 Hz), 118.8 (d, J = 23.6 Hz), 116.9 (q, J = 288.5 Hz)*, 67.4, 52.5, 39.7, 14.0.

^{19}F{^{1}H} NMR (376 MHz, CD_3CN) δ (ppm) −76.30 (s, 3F), −114.19 (ddd, J = 10.5, 6.3, 4.1 Hz, 1F).

HRMS (ESI−): found $[M - H]^-$, m/z($C_{14}H_{12}F_4NO_5^-$) calculated 350.0657, found 350.0657, δ = + 0.1 ppm

*Note: The peaks labelled with * overlap with the peaks of the other isomer or the starting material.*

Synthesis of the Unsuccessful Starting Materials

4-methyl-*N*-phenylbenzenesulfonamide

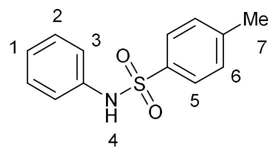

Prepared according to **GP7** using aniline (0.46 mL, 5.0 mmol, 1.0 equiv.), Et_3N (1.04 mL, 5.0 mmol, 1.0 equiv.) and *p*-tolylsulfonyl chloride (953 mg, 7.5 mmol, 1.5 equiv.). The mix was stirred at 25 °C for 1h. The reaction was quenched with water, and the aqueous phase was extracted with DCM (2×). Combined organics were washed with brine (1×), dried over $MgSO_4$ and concentrated *in vacuo*. Purification by silica gel chromatography (Hex:EtOAc – 7:1) yielded the product as a white powder (517 mg, 2.1 mmol, 42%).

^{1}H NMR (500 MHz, $CDCl_3$): δ (ppm) 7.77 – 7.68 (m, 3H, H4, H5), 7.22 – 7.18 (m, 4H, H2, H6), 7.17 – 7.11 (m, 2H, H3), 7.10 – 7.03 (m, 1H, H1), 2.33 (s, 3H, H7).

^{13}C NMR (126 MHz, $CDCl_3$): δ (ppm) 143.9, 136.8, 136.0, 129.7, 129.3, 127.3, 125.1, 121.3, 21.5

The spectroscopic data is in agreement with that reported in the literature.[54]

1,1,1-trifluoro-*N*-phenylmethanesulfonamide

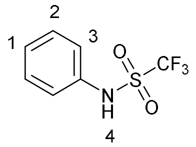

Prepared according to **GP7** using aniline (0.92 mL, 10.0 mmol, 1.0 equiv.), Et_3N (1.53 mL, 11.0 mmol, 1.1 equiv.) and triflic anhydride (1.68 mL, 10.0 mmol, 1.0 equiv.). Purification by silica gel chromatography (Hex:acetone – 97:3 to 90:10) yielded the product as white crystals (1.35 g, 6.0 mmol, 60%).

^{1}H NMR (400 MHz, $CDCl_3$): δ (ppm) 7.43 – 7.37 (m, 2H, H2), 7.32 (tt, J = 7.4, 1.2 Hz, 1H, H1), 7.30 – 7.27 (m, 2H, H3), 6.93 (br s, 1H, H4).

^{13}C NMR (126 MHz, $CDCl_3$): δ (ppm) 133.7, 129.9, 127.8, 123.8, 119.9 (q, J = 322.6 Hz).

^{19}F NMR (376 MHz, $CDCl_3$): δ (ppm) −75.32

The spectroscopic data is in agreement with that reported in the literature.[55]

***N*-(2-chlorophenyl)-2,2,2-trifluoroacetamide (1ab)**

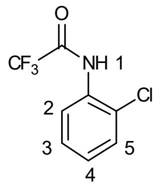

Prepared according to the **GP7** using 2-chloroaniline (0.32 mL, 3.0 mmol, 1.1 equiv.), TFAA (0.38 mL, 2.7 mmol, 1.0 equiv.), and TEA (0.46 mL, 3.3 mmol, 1.2 equiv.). Washes with hexane yielded the compound as white crystals (540 mg, 2.4 mmol, 90%).

^{1}H NMR (500 MHz, $CDCl_3$) δ (ppm) 8.43 (br s, 1H, H1), 8.32 (dd, J = 8.2, 1.4 Hz, 1H, H5), 7.44 (dd, J = 8.0, 1.4 Hz, 1H, H2), 7.35 (app. td, J = 7.9, 1.4 Hz, 1H, H3), 7.19 (app. td, J = 7.8, 1.5 Hz, 1H, H4).

^{13}C NMR (126 MHz, $CDCl_3$) δ (ppm) 154.8 (q, J = 37.8 Hz), 132.2, 129.5, 128.2, 126.9, 123.9, 122.0, 115.7 (q, J = 289.2 Hz).

^{19}F NMR (376 MHz, $CDCl_3$) δ (ppm) −75.9

The NMR data is in agreement with that reported in the literature.[56]

2,2,2-trifluoro-*N*-(8-oxo-5,6,7,8-tetrahydronaphthalen-1-yl)acetamide (1ac)

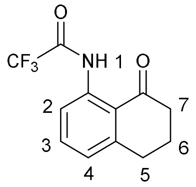

A variant of the protocol reported by Garst was adapted. The 2,2,2-trifluoro-*N*-(5,6,7,8-tetrahydronaphthalen-1-yl)acetamide (243 mg, 1.0 mmol, 1.0 equiv.) was dissolved in acetone (6.3 mL), and $KMnO_4$ (379 mg, 2.4 mmol, 2.4 equiv.) was added together with aq. $MgSO_4$ (0.9 mL, 15 wt%). The mix was stirred at rt for 18 h. Then, the mixture was filtered through Celite, and the solids were washed with $CHCl_3$ and H_2O. The aqueous phase was extracted with $CHCl_3$, and the combined organics were dried over $MgSO_4$ and concentrated in vacuo. Purification by silica gel chromatography (Hex:EtOAc – 19:1) yielded the title compound as white crystals (126 mg, 0.5 mmol, 49%).

^{1}H NMR (400 MHz, $CDCl_3$) δ (ppm) 13.36 (br s, 1H, H1), 8.52 (d, J = 8.4 Hz, 1H, H2), 7.52 (app. t, J = 8.0 Hz, 1H, H3), 7.09 (dd, J = 7.8, 1.2 Hz, 1H, H4), 3.02 (t, J = 6.1 Hz, 2H, H7), 2.74 (t, J = 6.9, 6.4 Hz, 2H, H5), 2.13 (app. p, J = 6.5 Hz, 3H, H6).

^{13}C NMR (101 MHz, $CDCl_3$) δ (ppm) 204.0, 155.8 (d, J = 37.8 Hz), 146.7, 139.4, 135.4, 125.5, 119.3, 118.7, 115.9 (q, J = 288.7 Hz), 40.5, 30.8, 22.7

^{19}F NMR (376 MHz, $CDCl_3$) δ (ppm) −76.3

The ^{1}H NMR data is in agreement with that reported in the literature.[57]

2,2,2-trifluoro-*N*-(2-(trifluoromethyl)phenyl)acetamide (1ad)

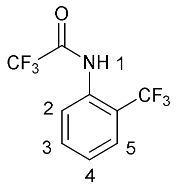

Prepared according to the **GP7** using 2-trifluoromethylaniline (0.38 mL, 3.0 mmol, 1.1 equiv.), TFAA (0.38 mL, 2.7 mmol, 1.0 equiv.), and TEA (0.46 mL, 3.3 mmol, 1.2 equiv.). Washes with hexane yielded the compound as white crystals (524 mg, 2.0 mmol, 76%).

^{1}H NMR (400 MHz, $CDCl_3$) δ (ppm) 8.20 (br s, 1H, H1), 8.13 (d, J = 8.3 Hz, 1H, H5), 7.70 (d, J = 7.9 Hz, 1H, H2), 7.64 (t, J = 7.9 Hz, 1H, H3), 7.39 (t, J = 7.7 Hz, 1H, H4).

^{13}C NMR (101 MHz, $CDCl_3$) δ (ppm) 155.4 (q, J = 38.3 Hz), 133.4, 132.4, 126.9, 126.7 (q, J = 5.2 Hz), 124.9, 123.7 (q, J = 30.5 Hz), 121.8 (q, J = 30.2 Hz), 115.7 (d, J = 288.7 Hz).

^{19}F NMR (376 MHz, $CDCl_3$) δ (ppm) −60.7 (s, 3F), −76.2 (s, 3F)

The NMR data is in agreement with that reported in the literature.[58]

***N*-([1,1'-biphenyl]-2-yl)-2,2,2-trifluoroacetamide (1ae)**

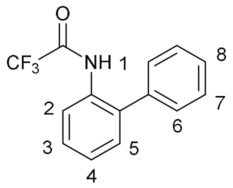

Prepared according to the **GP7** using 2-aminobiphenyl (846 mg, 5.0 mmol, 1.1 equiv.), TFAA (0.63 mL, 4.5 mmol, 1.0 equiv.), and TEA (0.77 mL, 5.5 mmol, 1.2 equiv.). Purification by silica gel chromatography (Hex:EtOAc – 95:5 to 90:10) yielded the title compound as white crystals (650 mg, 2.5 mmol, 54%).

^{1}H NMR (700 MHz, $CDCl_3$) δ (ppm) 8.30 (dd, J = 8.2, 1.1 Hz, 1H, H5), 7.99 (br s, 1H, H1), 7.56 – 7.50 (m, 2H, H7), 7.49 – 7.42 (m, 2H, H3, H8), 7.39 – 7.35 (m, 2H, H6), 7.34 (dd, J = 7.6, 1.8 Hz, 1H, H2), 7.31 (app. td, J = 7.4, 1.2 Hz, 1H, H4).

^{13}C NMR (176 MHz, $CDCl_3$) δ (ppm) 154.7 (q, J = 37.2 Hz), 136.9, 133.3, 132.3, 130.5, 129.6, 129.2, 128.9, 128.8, 126.4, 121.4, 115.8 (q, J = 288.9 Hz).

^{19}F NMR (376 MHz, $CDCl_3$) δ (ppm) −76.11 (d, J = 1.4 Hz).

The NMR data is in agreement with that reported in the literature.[59]

2,2,2-trifluoro-*N*-(3-iodophenyl)acetamide (1af)

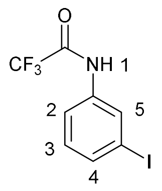

Prepared according to the **GP7** using 2-iodoaniline (1.095 g, 5.0 mmol, 1.0 equiv.), TFAA (0.76 mL, 5.0 mmol, 1.0 equiv.), and TEA (0.84 mL, 5.5 mmol, 1.1 equiv.). Reprecipitation from DCM using PE yielded the compound as pink crystals (904 mg, 2.9 mmol, 57%).

^{1}H NMR (400 MHz, $CDCl_3$) δ (ppm) 7.96 (t, J = 1.9 Hz, 1H, H5), 7.84 (br s, 1H, H1), 7.59 (ddd, J = 7.9, 1.6, 0.9 Hz, 1H, H2), 7.56 (ddd, J = 8.2, 2.2, 0.9 Hz, 1H, H4), 7.12 (t, J = 8.0 Hz, 1H, H3).

^{13}C NMR (101 MHz, $CDCl_3$) δ (ppm) 154.9 (q, J = 37.7 Hz), 136.2, 135.6, 130.9, 129.3, 119.8, 115.7 (q, J = 288.6 Hz), 94.3.

^{19}F NMR (376 MHz, $CDCl_3$) δ (ppm) −75.7

HRMS (ESI−): found $[M-H]^-$, m/z($C_8H_4F_3INO^-$) calculated 313.9295, found 313.9300, δ = +1.4 ppm

2,2,2-trifluoro-*N*-(naphthalen-1-yl)acetamide (1ag)

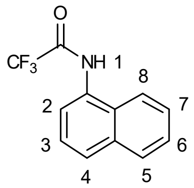

Prepared according to the **GP7** using 1-naphthylamine (1.58 g, 11.0 mmol, 1.1 equiv.), TFAA (1.4 mL, 10.0 mmol, 1.0 equiv.), and TEA (1.7 mL, 12.1 mmol, 1.2 equiv.). Reprecipitation from DCM using hexane yielded the title compound as purple crystals (1.28 g, 5.3 mmol, 53%).

^{1}H NMR (400 MHz, $CDCl_3$) δ (ppm) 8.27 (br s, 1H, H1), 7.91 (dd, J = 7.8, 1.7 Hz, 1H, H8), 7.87 (dd, J = 7.5, 1.1 Hz, 1H, H2), 7.84 – 7.78 (m, 1H, H4), 7.79 – 7.72 (m, 1H, H5), 7.62 – 7.53 (m, 2H, H6, H7), 7.49 (dd, J = 8.3, 7.5 Hz, 1H, H3).

^{13}C NMR (101 MHz, $CDCl_3$) δ (ppm) 155.8 (q, J = 37.4 Hz), 134.2, 129.3, 129.1, 128.0, 127.3, 127.1, 126.7, 125.6, 121.8, 120.1, 116.2 (q, J = 288.8 Hz).

^{19}F NMR (376 MHz, $CDCl_3$) δ (ppm) −75.4

The NMR data is in agreement with that reported in the literature.[60]

Unsuccessful Substrate Scope Entries

Summary of the unsecured substrate entries

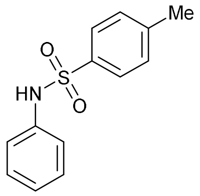

Cat 1: 19% - 1 : 1.7
Cat 2: 38% - 1 : 1.9

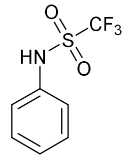

substrate decomposition

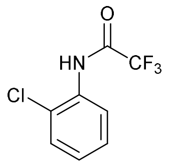

Cat 1: 16% - 1 : 7.0
Cat 2: 27% - 1 : 5.8

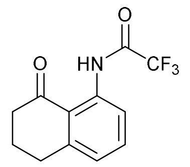

Cat 1: 34% - 1.6 : 1
Cat 2: 38% - 1.9 : 1

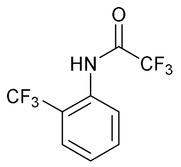

yield < 1%

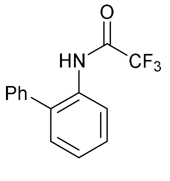

complex mix

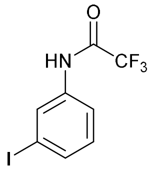

substrate decomposition

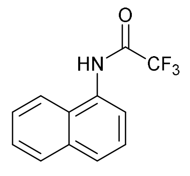

complex mix

Fluorination of 4-methyl-*N*-phenylbenzenesulfonamide

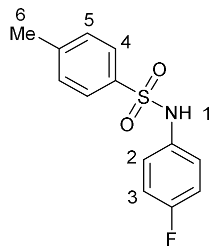

Reaction with Complex 2

Reaction conducted according to the **GP8** using 4-methyl-*N*-phenylbenzenesulfonamide (24.7 mg, 0.1 mmol, 1.0 equiv.) and [Pd(s-tpy)(MeCN)](OTf) (3.1 mg, 0.005 mmol, 5 mol%). The mixture was allowed to cool to rt and filtered through a silica gel plug. The $^{19}F\{^1H\}$ NMR showed 45% NMR yield and *ortho*-:*para*-:dearomatized ratio of 13:25:7. Purification by silica gel chromatography (PE:EtOAc – 100:0 to 87:13) yielded a white crystalline mixture of the *para*- isomer (4.0 mg, 15.1 μmol, 15%) and starting material (2.7 mg, 11% recovery).

Reaction with Complex 1

Reaction conducted according to the **GP8** using 4-methyl-*N*-phenylbenzenesulfonamide (24.7 mg, 0.1 mmol, 1.0 equiv.) and [Pd(tpy)(MeCN)](BF_4)$_2$ (2.8 mg, 0.005 mmol, 5 mol%). The $^{19}F\{^1H\}$ NMR showed 19% NMR yield and *ortho*-:*para*-:dearomatized ratio 7:12:0.

Assignment of the products: The *para*- isomer was isolated as a mixture with the starting material. The positional isomer of the compound was assigned by the triplet of triplets observed in ^{19}F NMR. The

ortho- isomer and dearomatised product were assigned by analogy with the results from fluorination of 2,2,2-trifluoro-*N*-phenylacetamide.

***Para*- isomer**

^{1}H NMR (700 MHz, CD_3CN) δ (ppm) 7.82 (br s, 1H, H1)*, 7.59 (d, J = 8.3 Hz, 2H, H4), 7.33 – 7.27 (m, 2H, H6)*, 7.12 – 7.05 (m, 2H, H2)*, 6.98 (app. t, J = 8.8 Hz, 2H, H3), 2.37 (s, 3H, H6).

^{13}C NMR (176 MHz, CD_3CN) δ (ppm) 161.1 (d, J = 242.2 Hz), 145.2, 137.1, 134.5 (d, J = 1.8 Hz), 130.6*, 128.1*, 124.7 (d, J = 8.0 Hz), 116.8 (d, J = 23.2 Hz), 21.5*.

^{19}F{^{1}H} NMR (376 MHz, CD_3CN) δ (ppm) −120.04 (tt, J = 8.8, 4.8 Hz).

*Note: The peaks labelled with * overlap with the peaks of the starting material.*

Fluorination of *N*-(2-chlorophenyl)-2,2,2-trifluoroacetamide (3ab)

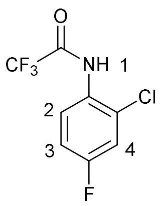

Reaction with Complex 2

Reaction conducted according to the **GP8** using *N*-(2-chlorophenyl)-2,2,2-trifluoroacetamide (22.4 mg, 0.1 mmol, 1.0 equiv.) and [Pd(s-tpy)(MeCN)](OTf) (3.1 mg, 0.005 mmol, 5 mol%). The mixture was allowed to cool to rt and filtered through a silica gel plug. The ^{19}F{^{1}H} NMR showed 31% NMR yield and *ortho*-:*para*-:dearomatized ratio of 5:23:4.

Reaction with Complex 1

Reaction conducted according to the **GP8** using *N*-(2-chlorophenyl)-2,2,2-trifluoroacetamide (22.4 mg, 0.1 mmol, 1.0 equiv.) and $[Pd(tpy)(MeCN)](BF_4)_2$ (2.8 mg, 0.005 mmol, 5 mol%). The ^{19}F{^{1}H} NMR showed 18% NMR yield and *ortho*-:*para*-:dearomatized ratio 3:13:2.

Assignment of the products: An attempt at the product isolation was not successful. However, the ^{19}F spectra of the crude reactions, both with Complex 1 and 2, showed the same ratios of products, although in different yields. The products were assigned as *para*- and *ortho*- isomers based on analogy with the previous results, but the nature of the products remains speculative.

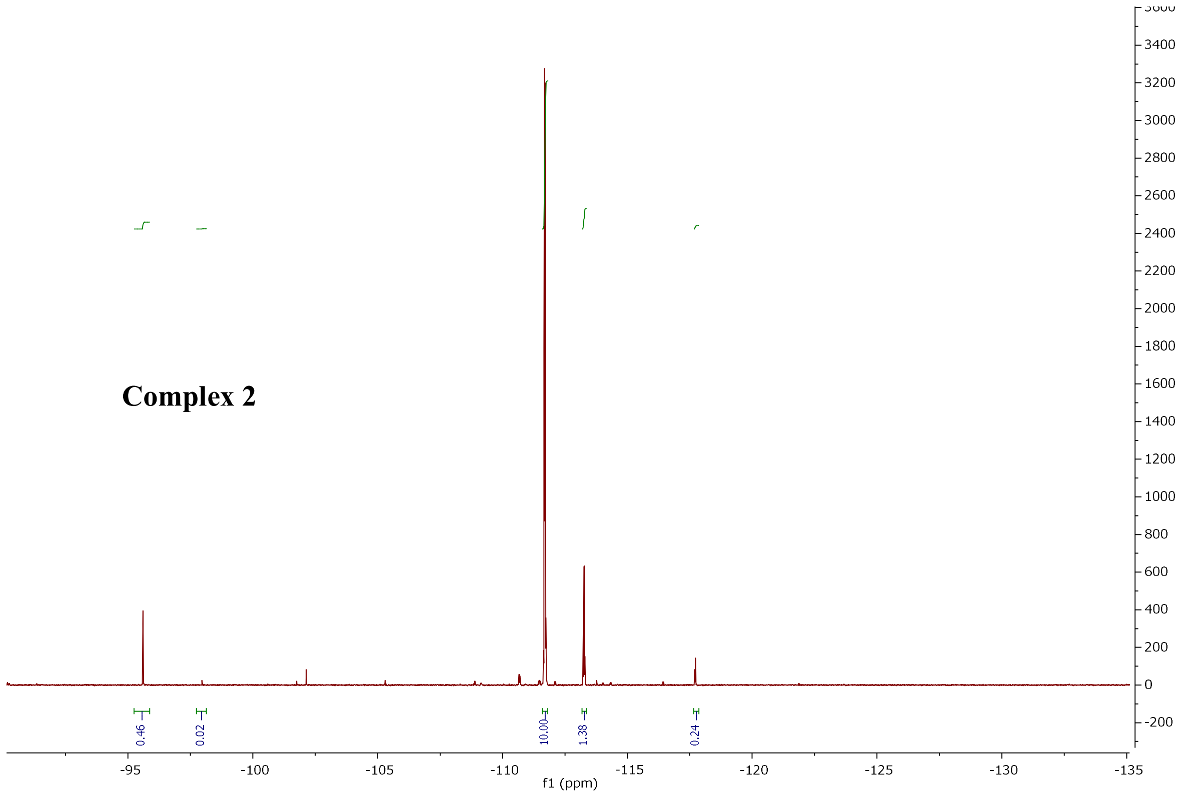
Complex 2
0.46
0.02
10.00
1.38
0.24
f1 (ppm)

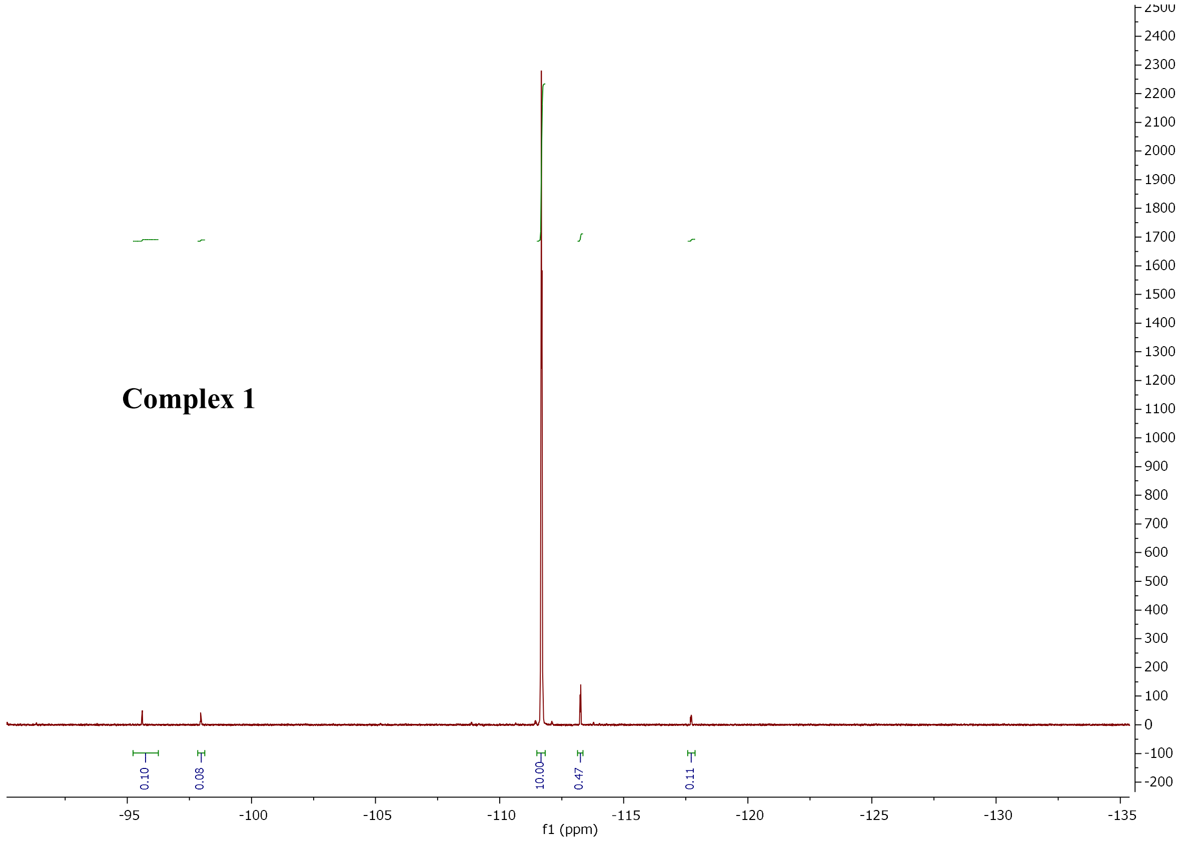
Complex 1
0.10
0.08
10.00
0.47
0.11
f1 (ppm)

Fluorination of 2,2,2-trifluoro-*N*-(8-oxo-5,6,7,8-tetrahydronaphthalen-1-yl)acetamide

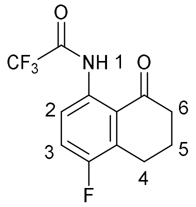

Reaction with Complex 2

Reaction conducted according to the **GP8** using 2,2,2-trifluoro-*N*-(8-oxo-5,6,7,8-tetrahydronaphthalen-1-yl)acetamide (25.7 mg, 0.1 mmol, 1.0 equiv.) and [Pd(s-tpy)(MeCN)](OTf) (3.1 mg, 0.005 mmol, 5 mol%). The mixture was allowed to cool to rt and filtered through a silica gel plug. The $^{19}F\{^1H\}$ NMR showed 38% NMR yield and *ortho*-:*para*-:dearomatized ratio of 25:13:0. Purification by pTLC (PE:EtOAc – 90:10 × 2) yielded the *para*- isomer (2.5 mg, 9.1 μmol, 9%) as white crystals.

Reaction with Complex 1

Reaction conducted according to the **GP8** using 2,2,2-trifluoro-*N*-(8-oxo-5,6,7,8-tetrahydronaphthalen-1-yl)acetamide (25.7 mg, 0.1 mmol, 1.0 equiv.) and $[Pd(tpy)(MeCN)](BF_4)_2$ (2.8 mg, 0.005 mmol, 5 mol%). The $^{19}F\{^1H\}$ NMR showed 34% NMR yield and *ortho*-:*para*-:dearomatized ratio 21:13:0.

Assignment of the products: The *para*- isomer was isolated. The positional isomer of the compound was assigned on the basis of the HMBC coupling between H4 and ^{13}C-F. The *ortho*- isomer was assigned by analogy with the previous results.

***Para*- isomer (3ac)**

^{1}H NMR (700 MHz, CD_3CN) δ (ppm) 13.19 (br s, 1H, H1), 8.46 (dd, J = 9.2, 4.9 Hz, 1H), 7.41 (t, J = 9.0 Hz, 1H), 2.98 (t, J = 6.1 Hz, 3H, H4), 2.81 – 2.64 (m, 2H, H6), 2.13 – 2.07 (m, 2H, H5)

^{13}C NMR (176 MHz, CD_3CN) δ (ppm) 204.7, 157.2 (d, J = 242.1 Hz), 156.0 (q, J = 36.9 Hz), 136.1, 134.6 (d, J = 18.3 Hz), 122.4 (d, J = 23.7 Hz), 121.6 (d, J = 3.0 Hz), 120.1 (d, J = 7.9 Hz), 116.9 (d, J = 288.2 Hz), 40.6, 23.0 (d, J = 4.7 Hz), 22.3.

^{19}F{^{1}H} NMR (376 MHz, CD_3CN) δ (ppm) −77.45 (s, 3F), −119.23 (dd, J = 10.2, 5.0 Hz, 1F)).

HRMS (ESI−): found $[M - H]^-$, m/z($C_{12}H_8F_4NO_2^-$) calculated 274.0497, found 274.0495, δ = −0.7 ppm

Reaction on 1 mmol Scale

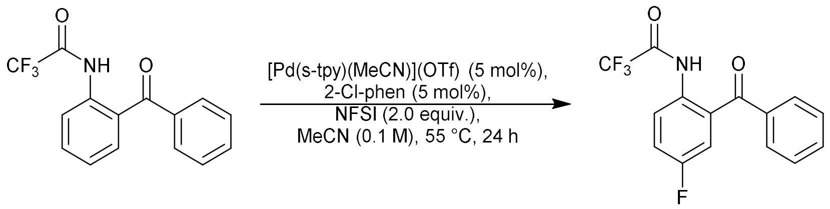

Under an inert atmosphere, the oven-dried 10.0 mL crimp-top vial was charged with the substrate (1.0 mmol, 293.2 mg, 1.0 equiv.), NFSI (631 mg, 2.0 mmol, 2.0 equiv.), 2-chloro-1,10-phenanthroline (11.0 mg, 0.05 mmol, 5 mol%) and [Pd(s-tpy)(MeCN)](OTf) (31.0 mg, 0.05 mmol, 5 mol%). The capped vial was evacuated and backfilled with nitrogen (3×). Afterwards, acetonitrile (10 mL) was added at once and the mix was stirred at 55 °C for 24 h. The mixture was allowed to cool to rt. The crude mixture was filtered through filtration paper and washed with MeCN (10 mL) and concentrated *in vacuo*. The $^{19}F\{^{1}H\}$ NMR showed 45% NMR yield and *ortho*-:*para*-:dearomatized ratio of 3:33:9. Purification by column chromatography (PE:EtOAc – 95:5) yielded the while crystalline mixture of the *para*- isomer (78.1 mg, 0.25 mmol, 25%) and starting material (107.9 mg, 0.37 mmol, 37% recovery). The dearomatised compound (1.9 mg, 8.1 µmol, 0.8%) was obtained as a yellow powder.

Synthesis and Fluorination of the *N*-Me Substrate

2,2,2-trifluoro-*N*-methyl-*N*-phenylacetamide (1ah)

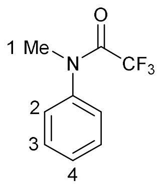

Prepared according to the **GP7** using *N*-methylaniline (1.2 mL, 10.0 mmol, 1.0 equiv.), TFAA (1.4 mL, 10.0 mmol, 1.0 equiv.), and TEA (1.7 mL, 12.1 mmol, 1.2 equiv.). Purification by silica gel chromatography (PE:EtOAc – 95:5) yielded the title compound as a yellow oil (1.04 g, 5.1 mmol, 51%).

^{1}H NMR (400 MHz, CD_3OD) δ (ppm) 7.57 – 7.40 (m, 3H, H3, H4), 7.39 – 7.28 (m, 2H, H2), 3.34 (s, 3H, H1).

^{13}C NMR (126 MHz, CD_3OD) δ (ppm) 157.9 (q, J = 35.6 Hz), 142.1, 130.6, 130.1, 128.6 (d, J = 1.2 Hz), 117.8 (q, J = 287.4 Hz), 40.0

^{19}F NMR (376 MHz, CD_3OD) δ (ppm) −69.2

Note, the compound exists in equilibrium with its rotamer, which is the most visible in additional N-Me peaks in ^{1}H and ^{13}C NMR (3.50 ppm and 49.8 ppm, respectively).

The NMR data is in agreement with that reported in the literature.[61]

Fluorination of 2,2,2-trifluoro-*N*-methyl-*N*-phenylacetamide (3ah)

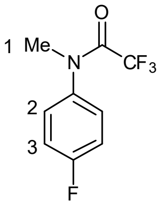

Reaction with Complex 2

Reaction conducted according to the **GP8** using 2,2,2-trifluoro-*N*-methyl-*N*-phenylacetamide (20.3 mg, 0.1 mmol, 1.0 equiv.) and [Pd(s-tpy)(MeCN)](OTf) (3.1 mg, 0.005 mmol, 5 mol%). The mixture was allowed to cool to rt and filtered through a silica gel plug. The ^{19}F{^{1}H} NMR showed 20% NMR yield and *ortho*-:*para*-:dearomatized ratio of 6:14:0.

Reaction with Complex 3

Reaction conducted according to the **GP8** using 2,2,2-trifluoro-*N*-methyl-*N*-phenylacetamide (20.3 mg, 0.1 mmol, 1.0 equiv.) and [Pd(Me-tpy)(MeCN)]$(BF_4)_2$ (2.8 mg, 0.005 mmol, 5 mol%). The ^{19}F{^{1}H} NMR showed 19% NMR yield and *ortho*-:*para*-:dearomatized ratio of 4:15:0.

Assignment of the products: Several attempts at purification of the products were made but only a small amount of the product was isolated (0.6 mg), together with unknown impurities. Hence, only ^{1}H and ^{19}F spectra were recorded. The yields and ratios were determined from analogy with the N-H substrate **1c**, as the products have relatively similar shifts in ^{19}F NMR and splitting patterns (*para*: −114.86 (tt, J = 8.9, 4.5 Hz) and *ortho*: −124.67 (dddd, *J* = 11.0, 8.3, 6.0, 2.9 Hz).

***Para*- isomer**

^{1}H NMR (400 MHz, $CDCl_3$) δ (ppm) 7.23 (dd, *J* = 8.6, 4.9 Hz, 2H, H2), 7.12 (app. t, *J* = 8.5 Hz, 2H, H3), 3.34 (s, 3H, H1).

^{19}F NMR (376 MHz, $CDCl_3$) δ (ppm) −67.12 (s, 3F), −111.53 (s, 1F).

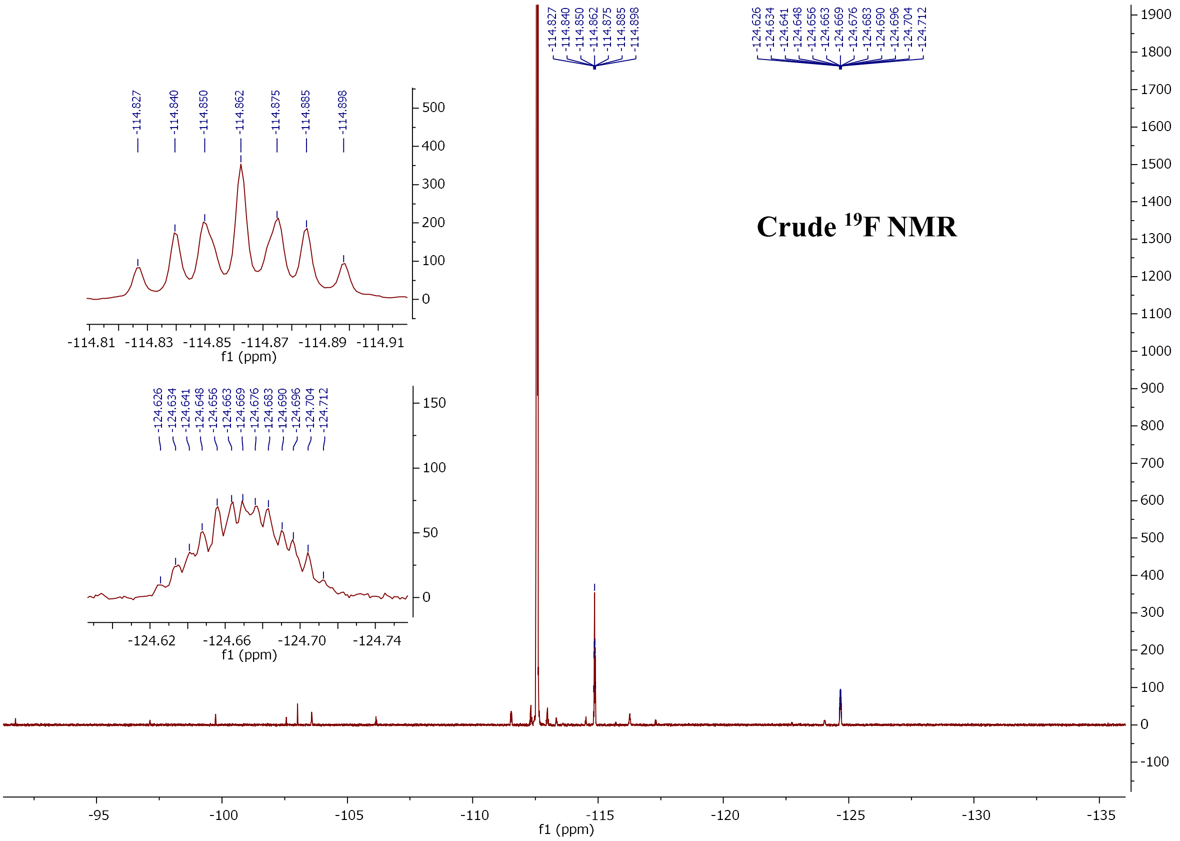

NMR Titrations

NMR titrations were carried out on a Bruker 400 MHz spectrometer using ^{1}H measurement. A 2 mL sample of the 2,2,2-trifluoro-*N*-phenylacetamide (5.1-8.7×10^{-3} M in CD_3CN). The NMR spectrum of the host solution (0.6 mL) was recorded. The ligand solution (0.34-1.0 ×10^{-3} M) was prepared using the host solution as solvent. Aliquots of the ligand solution were successively added to the NMR sample of the 2,2,2-trifluoro-*N*-phenylacetamide solution, the ^{1}H NMR spectrum was recorded after each addition, and the chemical shift of the N-H proton was observed. The resulting points were fitted (http://app.supramolecular.org/bindfit/) using the assumption of 1:1 binding, using a Nelder-Mead algorithm.

The 2,2,2-trifluoro-*N*-phenylacetamide was titrated with ligand 8 (**L8**) and 2,2':6',2"-terpyridine as a control, in order to rule out possible interaction of the pyridine nitrogens with the amide N-H bond on the substrate.

Table S7: Ligand 4 and 2,2,2-trifluoro-*N*-phenylacetamide 1c

c_1(N-H) [M]	c_2(L8) [M]	c_1:c_2	δ(N-H) [ppm]
0.0087237	0	0	9.1872
0.0087237	0.00688371	0.8	9.2951
0.0087237	0.01158852	1.3	9.3562
0.0087237	0.0143941	1.7	9.3963
0.0087237	0.01729511	2.0	9.4291
0.0087237	0.02308959	2.6	9.5013
0.0087237	0.02067516	2.4	9.4631
0.0087237	0.03284472	3.8	9.5929
0.0087237	0.05058409	5.8	9.7284
0.0087237	0.05945199	6.8	9.8061
0.0087237	0.0712726	8.2	9.8565

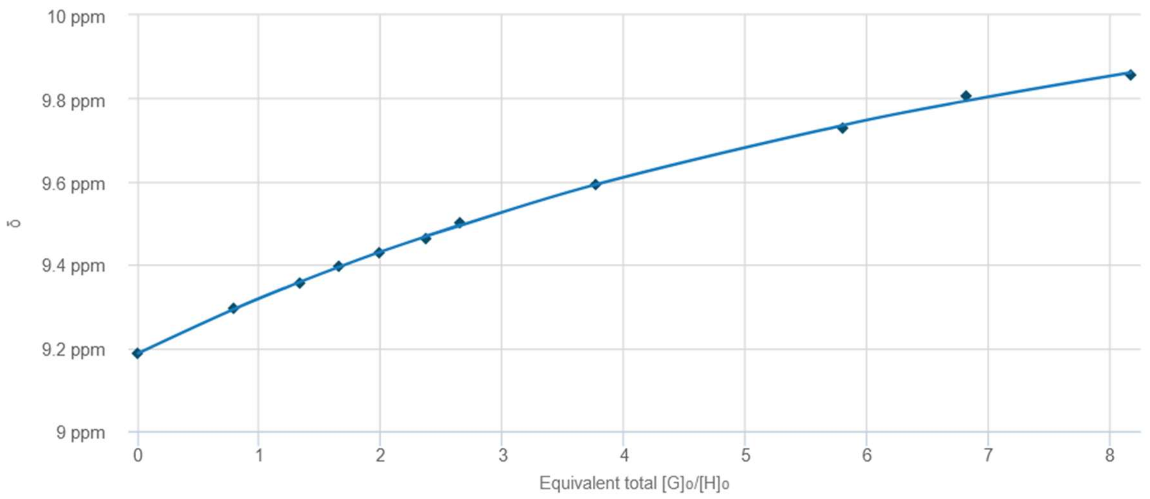

Calculated *Ka*: 12.2 ± 0.2 M^{-1}

Table S8: 4’-methyl-2,2':6',2''-terpyridine and 2,2,2-trifluoro-*N*-phenylacetamide 1c

c_1(N-H) [M]	c_2(tpy) [M]	c_1:c_2	δ(N-H) [ppm]
0.00845934	0	0	9.1898
0.00840647	0.00592656	0.7	9.1936
0.00837122	0.01012918	1.2	9.1959
0.00851221	0.01853534	2.2	9.1963
0.00854746	0.03157327	3.7	9.2004
0.00859152	0.04458999	5.2	9.2048
0.0086	0.057137	6.6	9.2104
0.00863558	0.06010363	7.0	9.2117

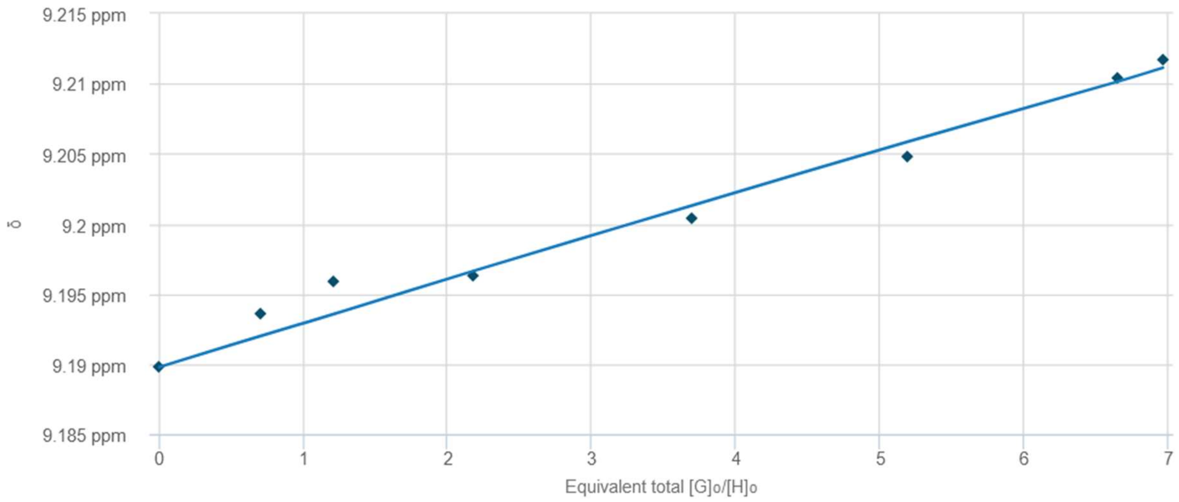

Calculated *K*a: 1.0 ± 0.1 M^{-1}

Table S9: Ligand 4 and 2,2,2-trifluoro-*N*-(4-fluorophenyl)acetamide 1c

c_1(N-H) [M]	c_2(L8) [M]	c_1:c_2	δ(N-H) [ppm]
0.00893159	0	0	9.224
0.00893159	0.00714527	0.8	9.3893
0.00893159	0.01159947	1.3	9.4783
0.00893159	0.01513829	1.7	9.5415
0.00893159	0.01751292	2.0	9.5871
0.00893159	0.02232897	2.5	9.6465
0.00893159	0.03572636	4.0	9.8183
0.00893159	0.05253876	5.9	10.0021
0.00893159	0.06379706	7.1	10.0868

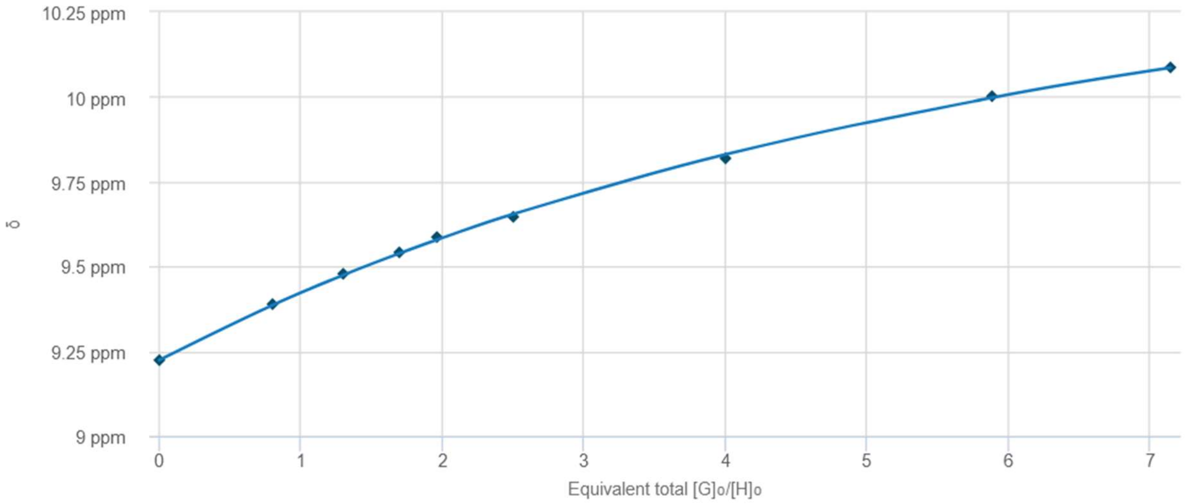

Calculated *K*a: 15.9 ± 0.3 M^{-1}

Kinetic Studies

All the time points correspond to individual reactions.

Reaction setup

Under an inert atmosphere, the oven-dried 4.0 mL crimp-top vial was charged with the 2,2,2-trifluoro-*N*-phenylacetamide (18.9 mg, 0.1 mmol, 1.0 equiv.), NFSI (63.1 mg, 0.2 mmol, 2.0 equiv.), 2-chloro-1,10-phenanthroline (1.1 mg, 0.005 mmol, 5 mol%) and either [Pd(tpy)(MeCN)](BF_4)$_2$ (2.8 mg, 0.005 mmol, 5 mol%) *OR* [Pd(s-tpy)(MeCN)](OTf) (3.1 mg, 0.005 mmol, 5 mol%). The capped vial was evacuated and backfilled with nitrogen (3×). Afterwards, acetonitrile (1.0 mL) was added at once and the mix was stirred at 55 °C and 1000 rpm for the specified time. Upon completion, the mixture was quickly cooled to rt and opened to air. Then, the mixture was concentrated *in vacuo*. The solid residue was redissolved in MeCN-d^3 and analysed by ^{19}F NMR using 1,3-difluorobenzene as internal standard.

Note, for reactions using [Pd(s-tpy)(MeCN)](OTf) catalyst, the crude mixture was filtered through a silica gel plug, washed with DCM (10 mL) prior to concentration in vacuo.

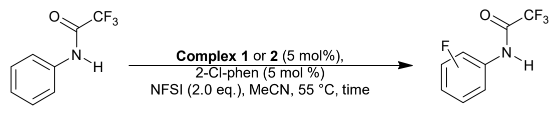

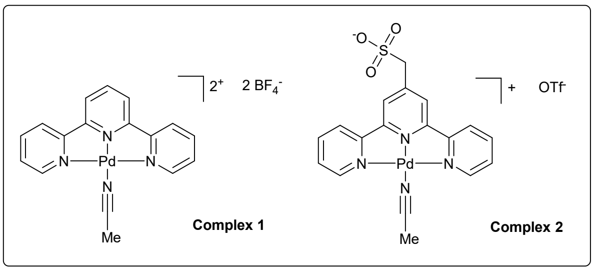

Reaction with Complex 1

t [min]	yield(*ortho*) [%]	yield(*para*) [%]	yield(dearo) [%]	Combined yield [%]
0	0.0	0.0	0.0	0.0
1	3.0	10.1	0.4	13.5
2	5.1	16.7	0.6	22.5
4	6.5	20.2	0.9	27.7
10	6.9	20.5	1.4	28.8
20	8.3	23.7	2.1	34.0
30	10.7	29.1	3.7	43.5
60	13.0	32.9	6.0	51.8
90	15.0	34.7	8.5	58.3
120	15.4	32.7	10.3	58.4
240	16.1	28.9	12.2	57.3
360	17.1	23.5	14.9	55.5
1440	16.9	15.8	14.0	46.7

Table S10: *The time course of the reaction with [Pd(tpy)(MeCN)](BF_4)$_2$ as a catalyst. The yields were determined by ^{19}F NMR using 1,3-difluorobenzene as an internal standard.*

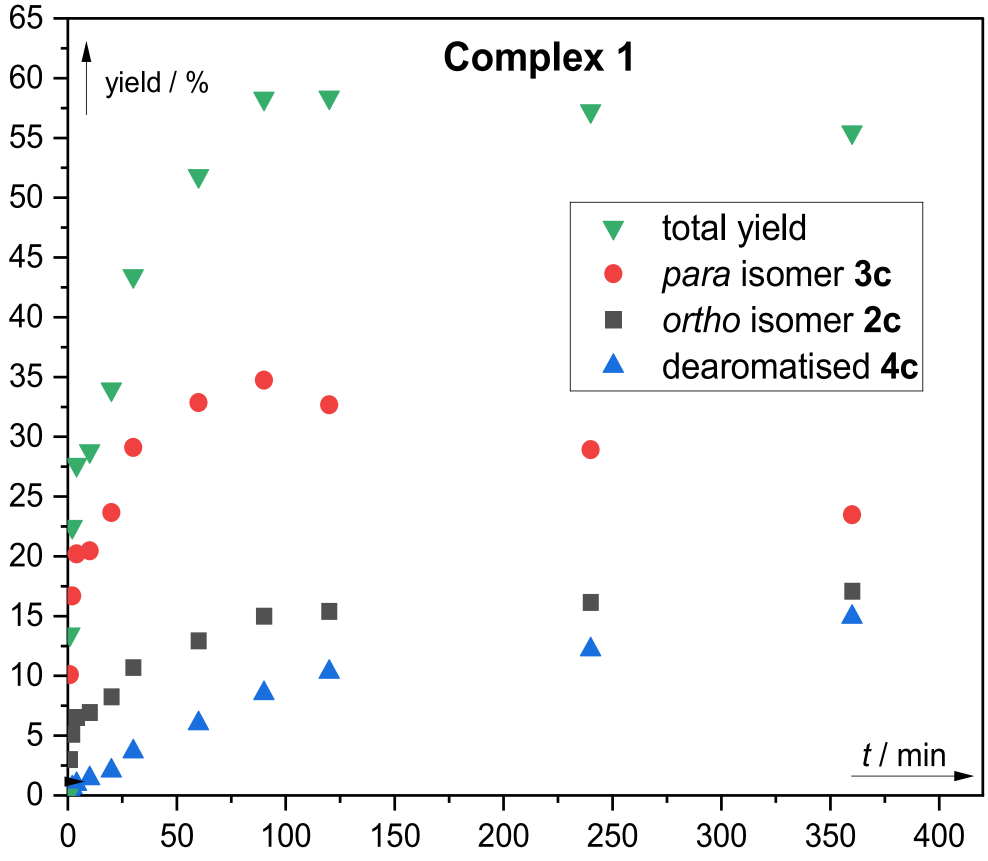

Figure S1: *The plot of products formation using Complex 1.*

Reaction with Complex 2

t *[min]*	yield(*ortho*) [%]	yield(*para*) [%]	yield(dearo) [%]	Combined yield [%]
0	0	0	0	0
2.5	0.6	9.3	0.1	10.0
5	0.9	11.4	0.2	12.6
15	1.3	14.6	0.5	16.4
30	1.0	12.1	0.2	13.3
60	1.6	15.4	0.5	17.5
120	1.8	17.2	0.5	19.6
240	1.6	19.8	0.5	21.9
1440	6.0	40.5	2.6	49.1

Table S11: *The time course of the reaction with [Pd(s-tpy)(MeCN)](OTf) as a catalyst. The yields were determined by ^{19}F NMR using 1,3-difluorobenzene as an internal standard.*

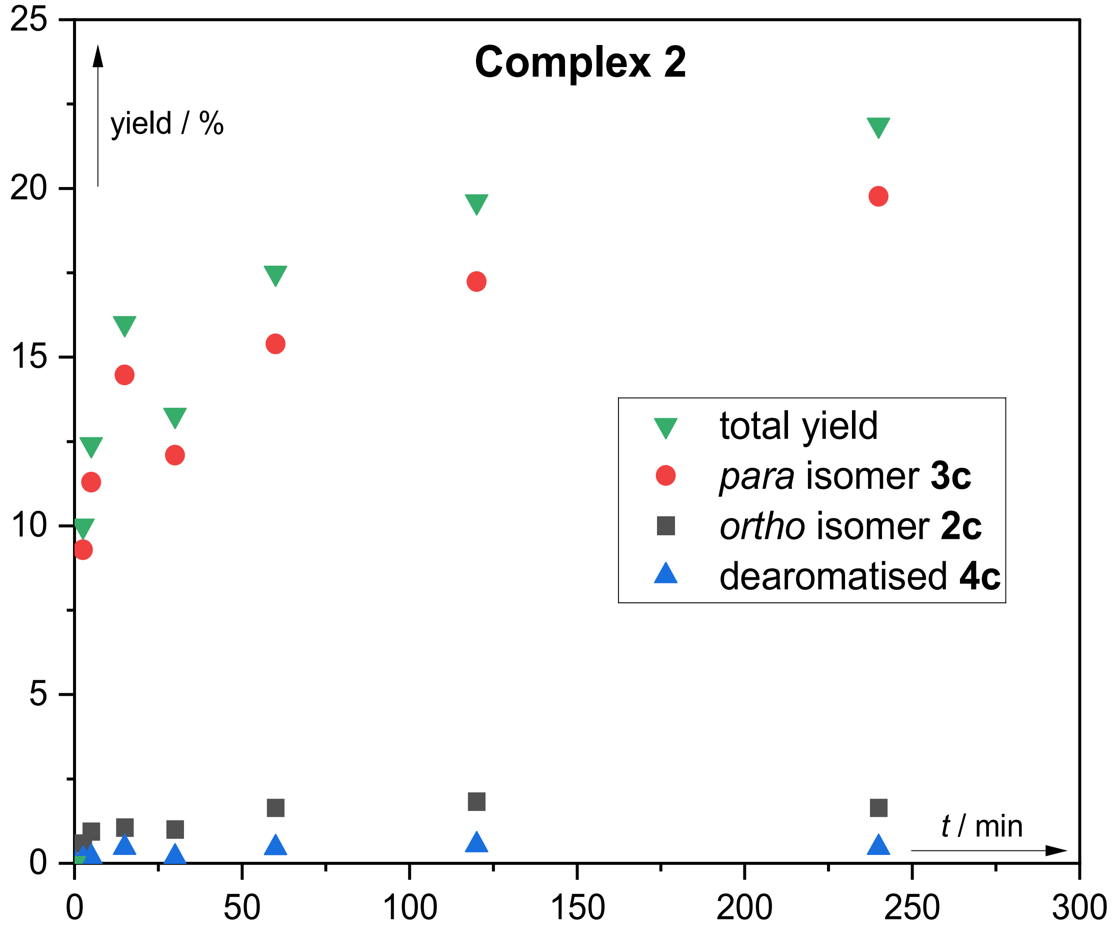

Figure S2: *The plot of products formation using Complex 2.*

NMR Spectra

¹H NMR (500 MHz, $CDCl_3$) for 6-bromo-2,2'-bipyridine

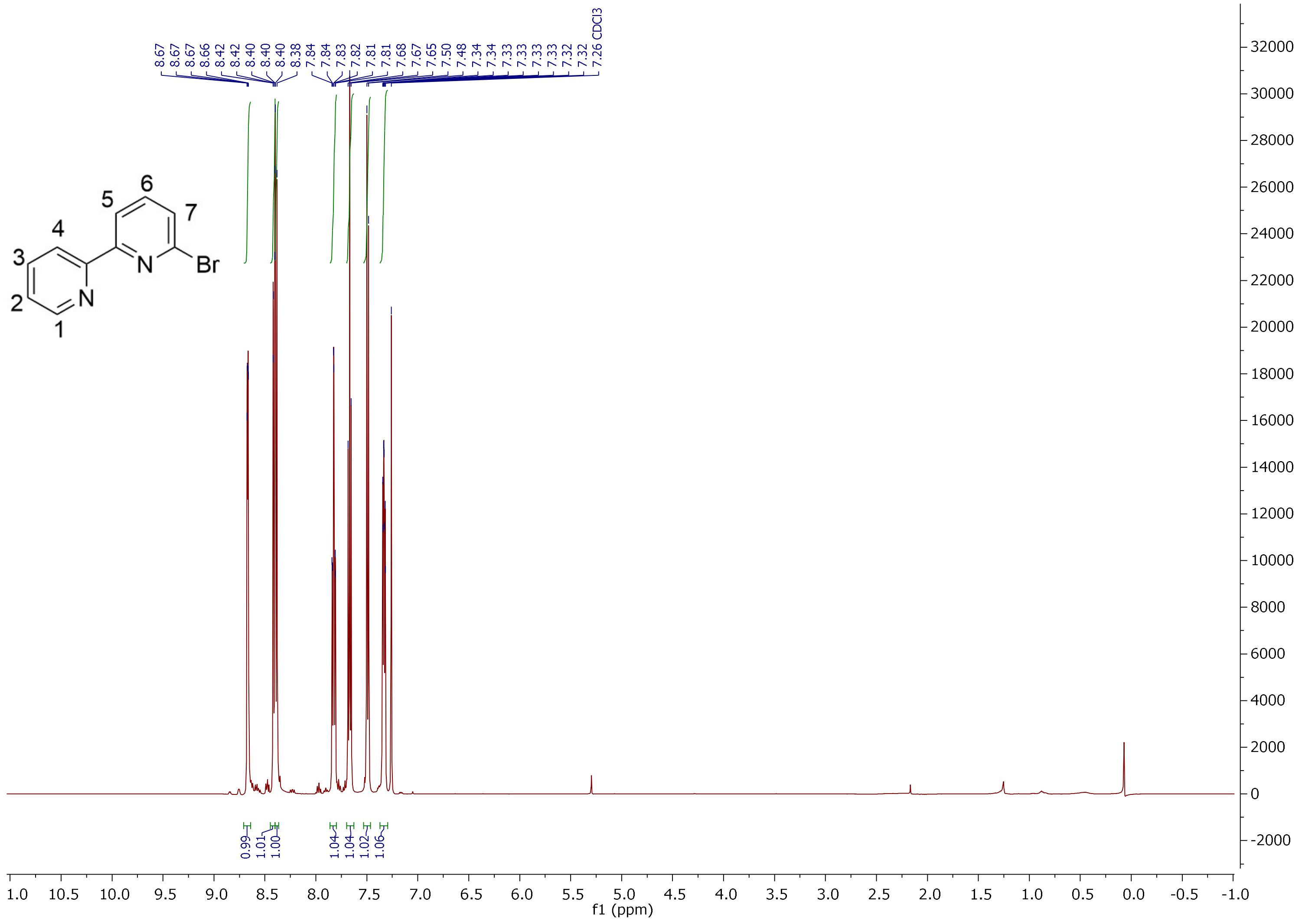

^{13}C NMR (126 MHz, $CDCl_3$) for 6-bromo-2,2'-bipyridine

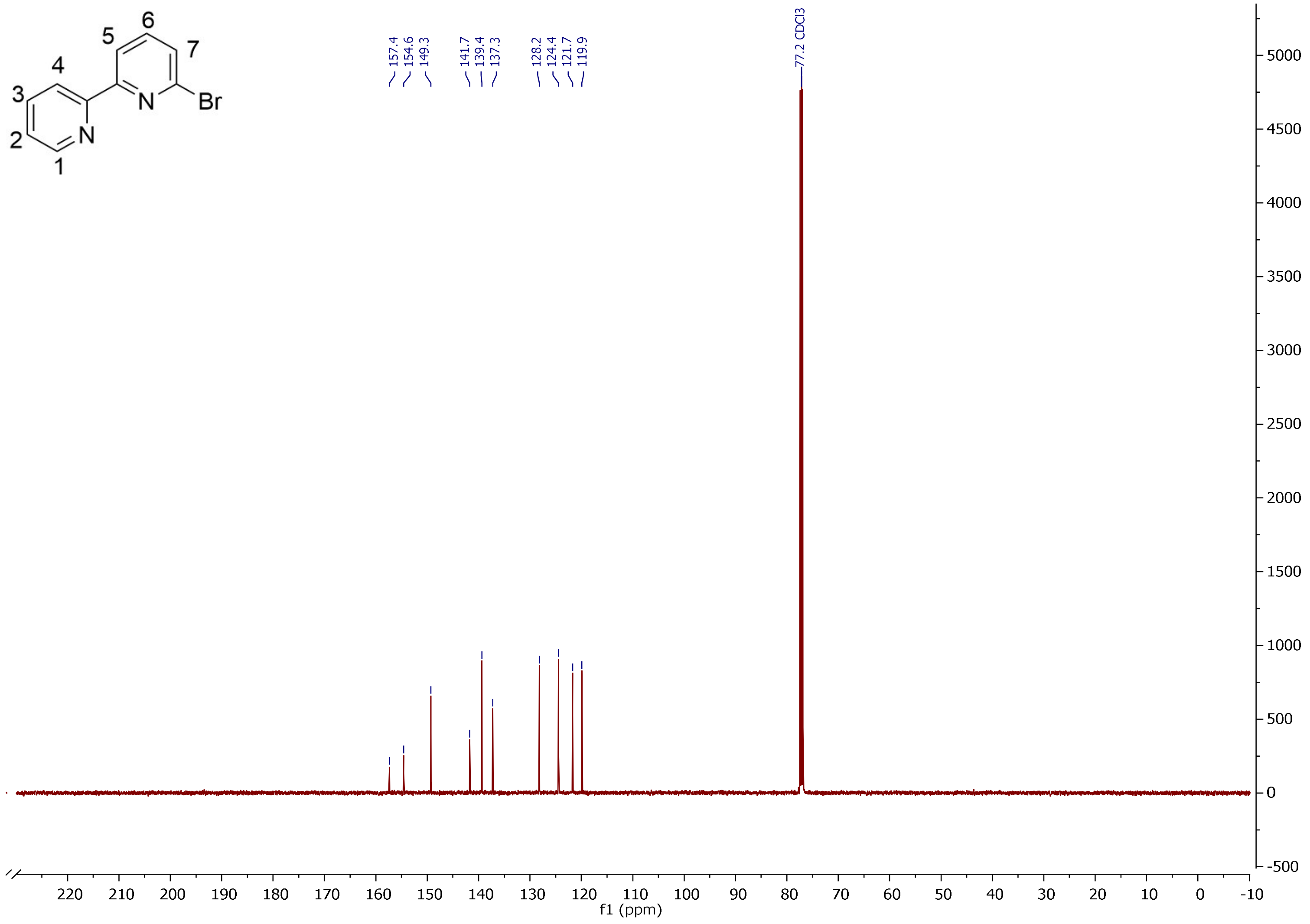

^{1}H NMR (500 MHz, $CDCl_3$) for 4-methyl-2,2':6',2"-terpyridine

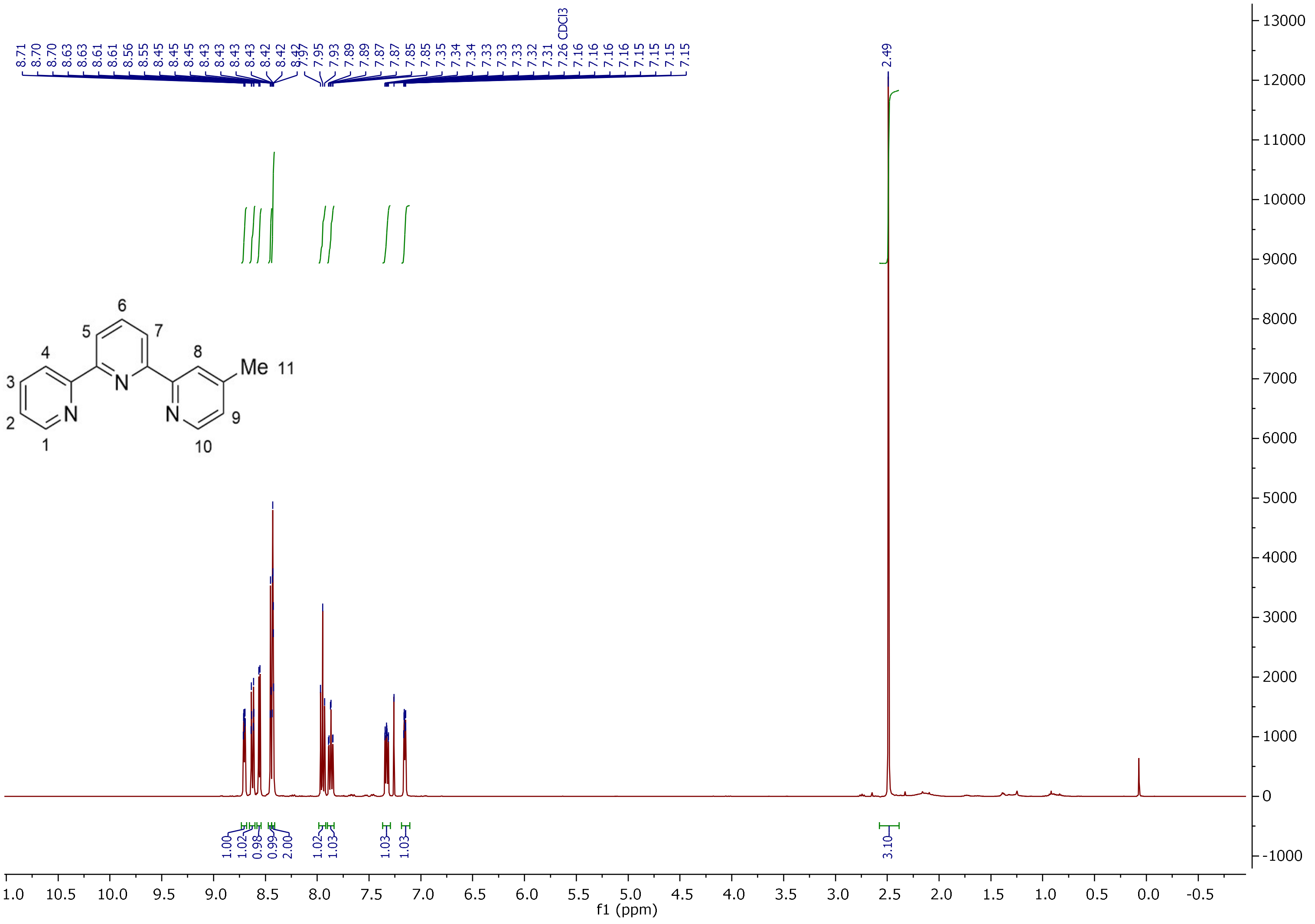

^{13}C NMR (126 MHz, $CDCl_3$) for 4-methyl-2,2':6',2"-terpyridine

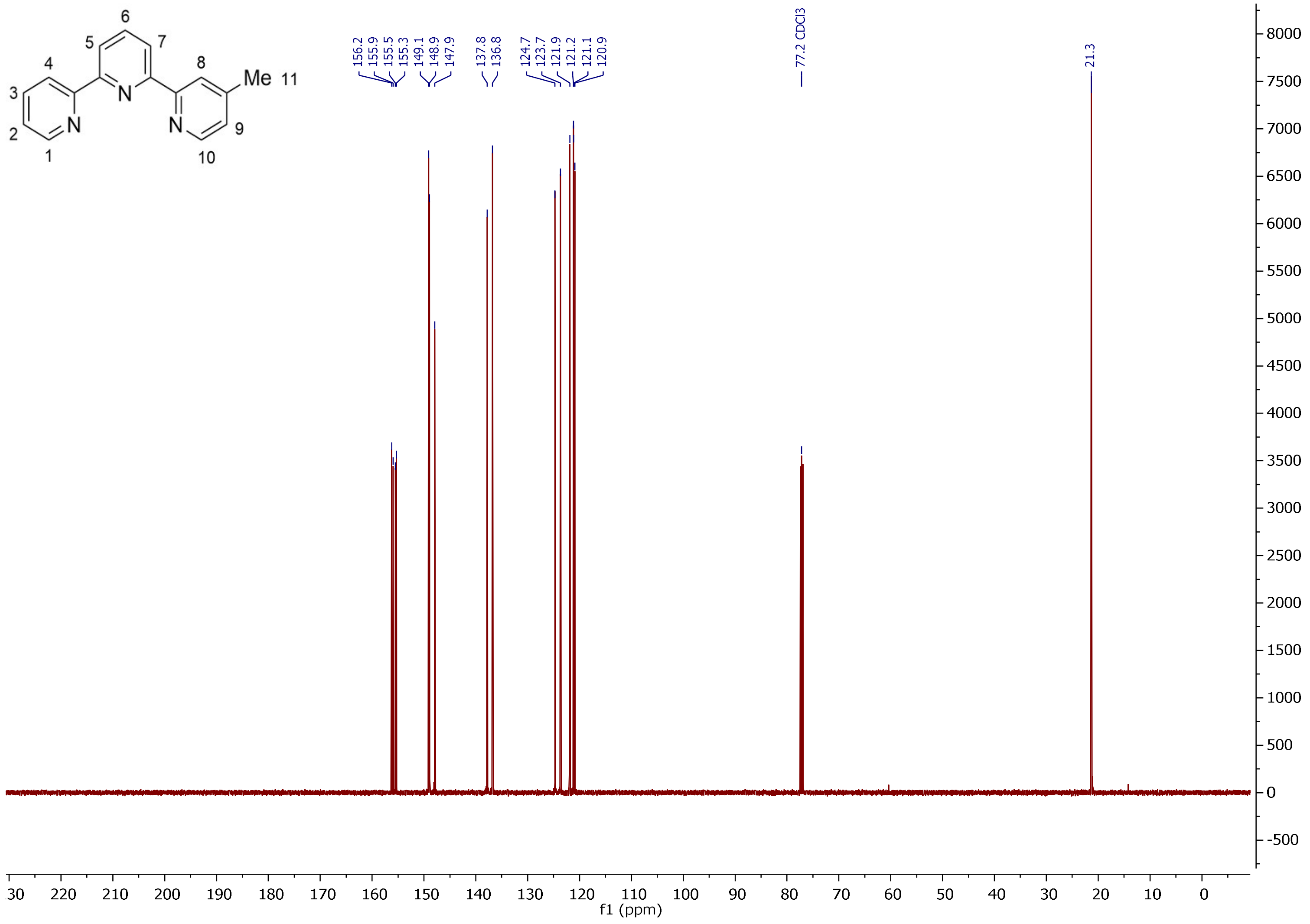

^{1}H NMR (400 MHz, $CDCl_3$) for [2,2':6',2''-terpyridin]-4-ylmethanol

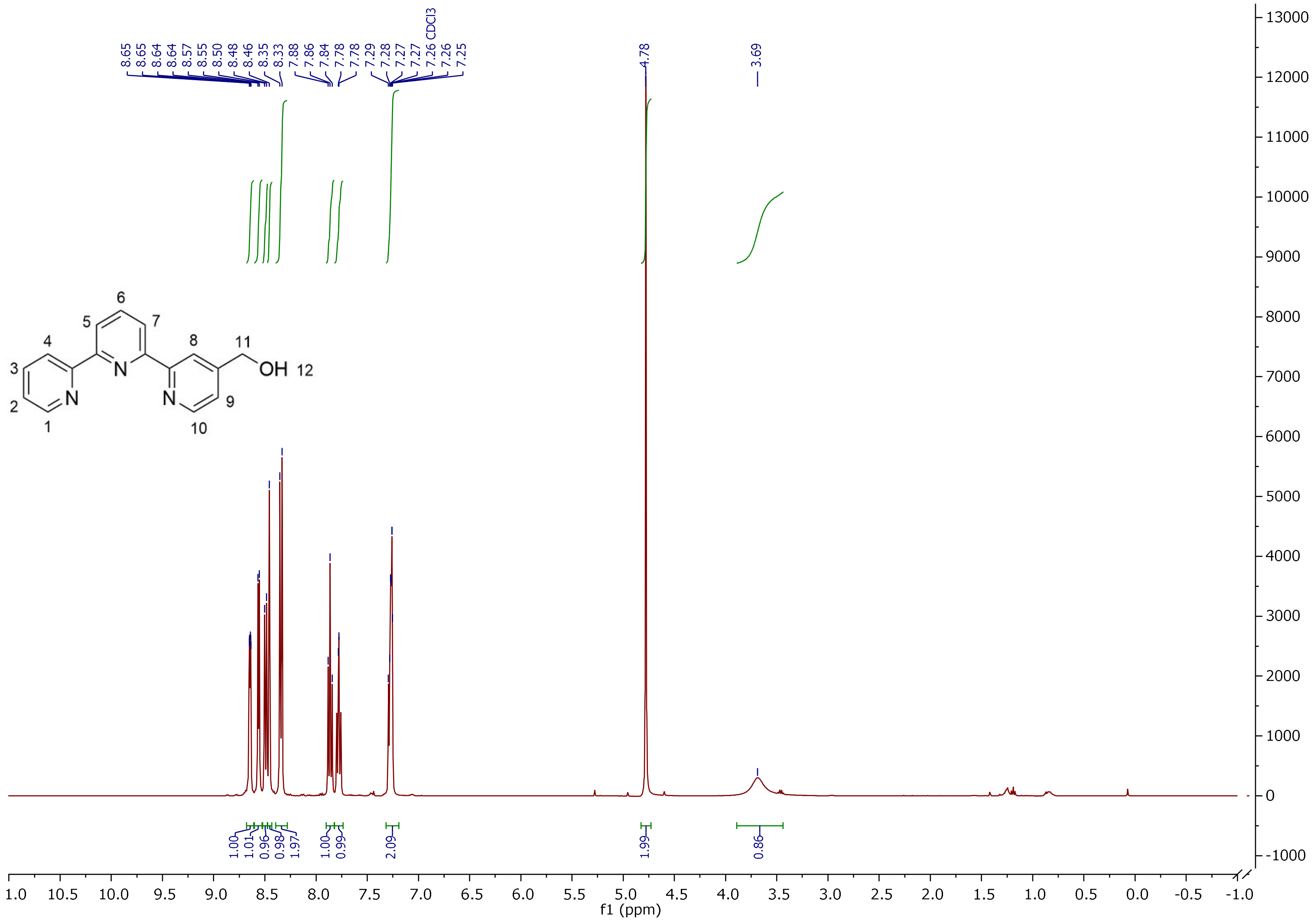

^{13}C NMR (101 MHz, $CDCl_3$) for [2,2':6',2"-terpyridin]-4-ylmethanol

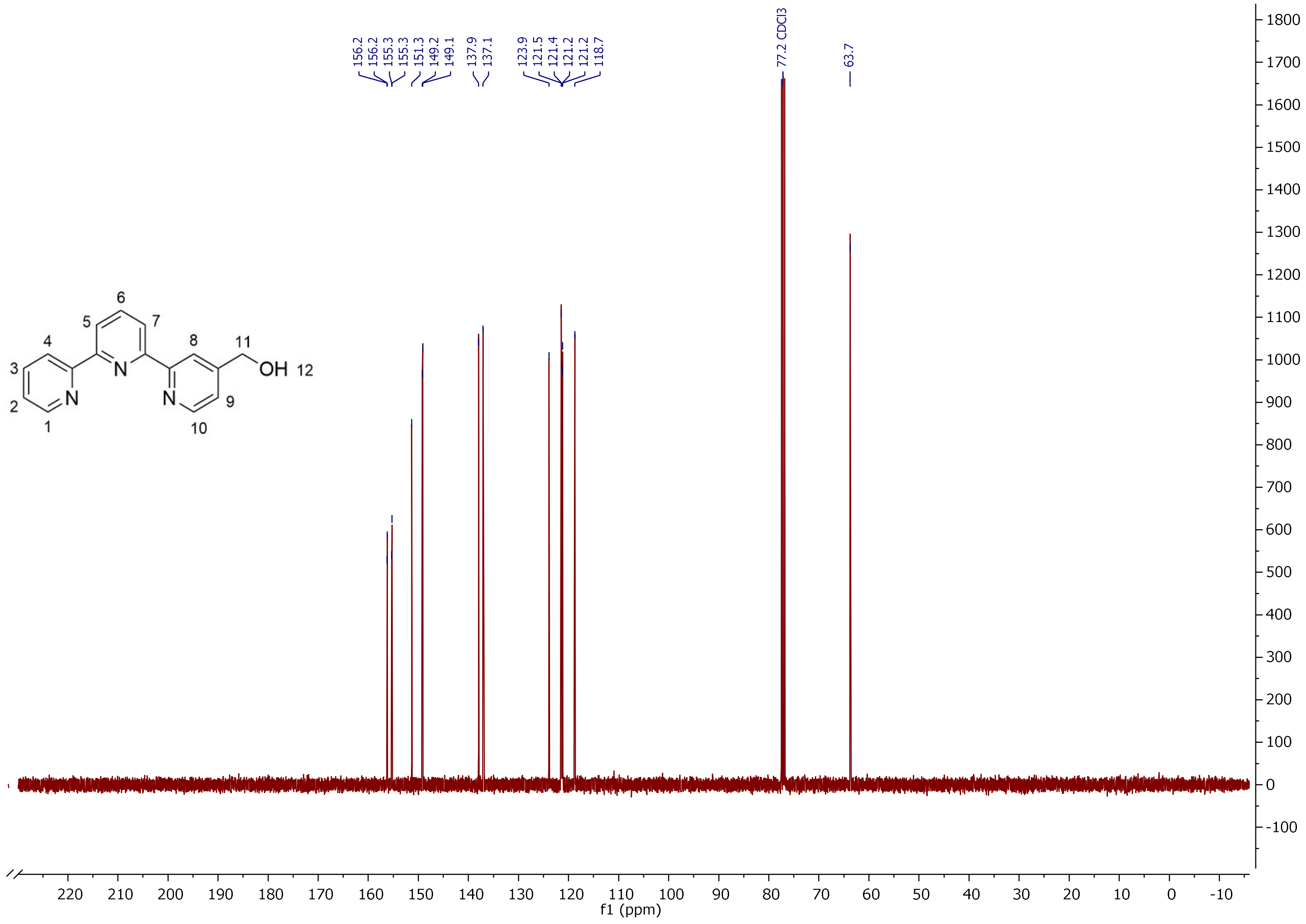

¹H NMR (500 MHz, $CDCl_3$) for 4-(bromomethyl)-2,2':6',2''-terpyridine

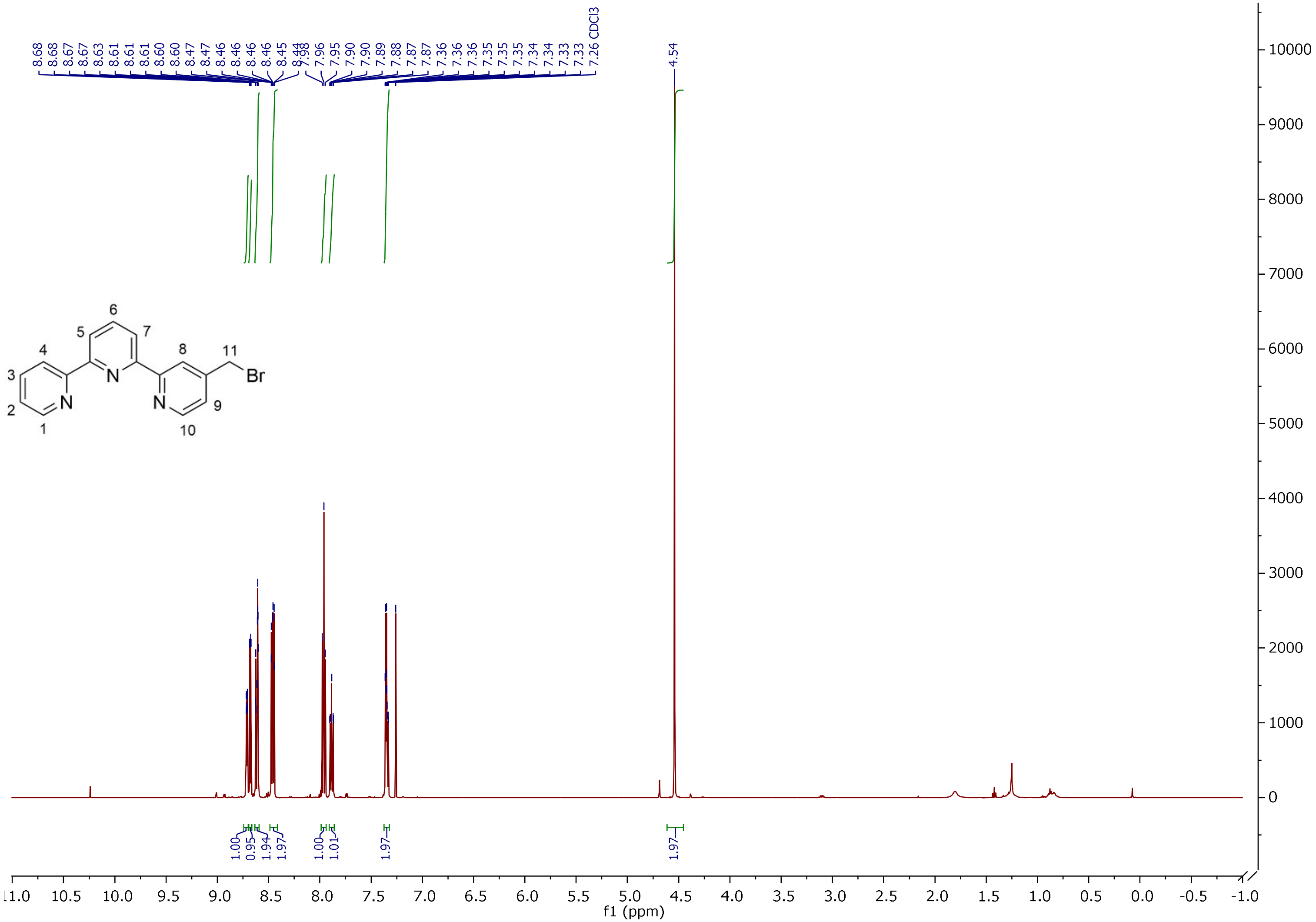

^{13}C NMR (126 MHz, $CDCl_3$) for 4-(bromomethyl)-2,2':6',2"-terpyridine

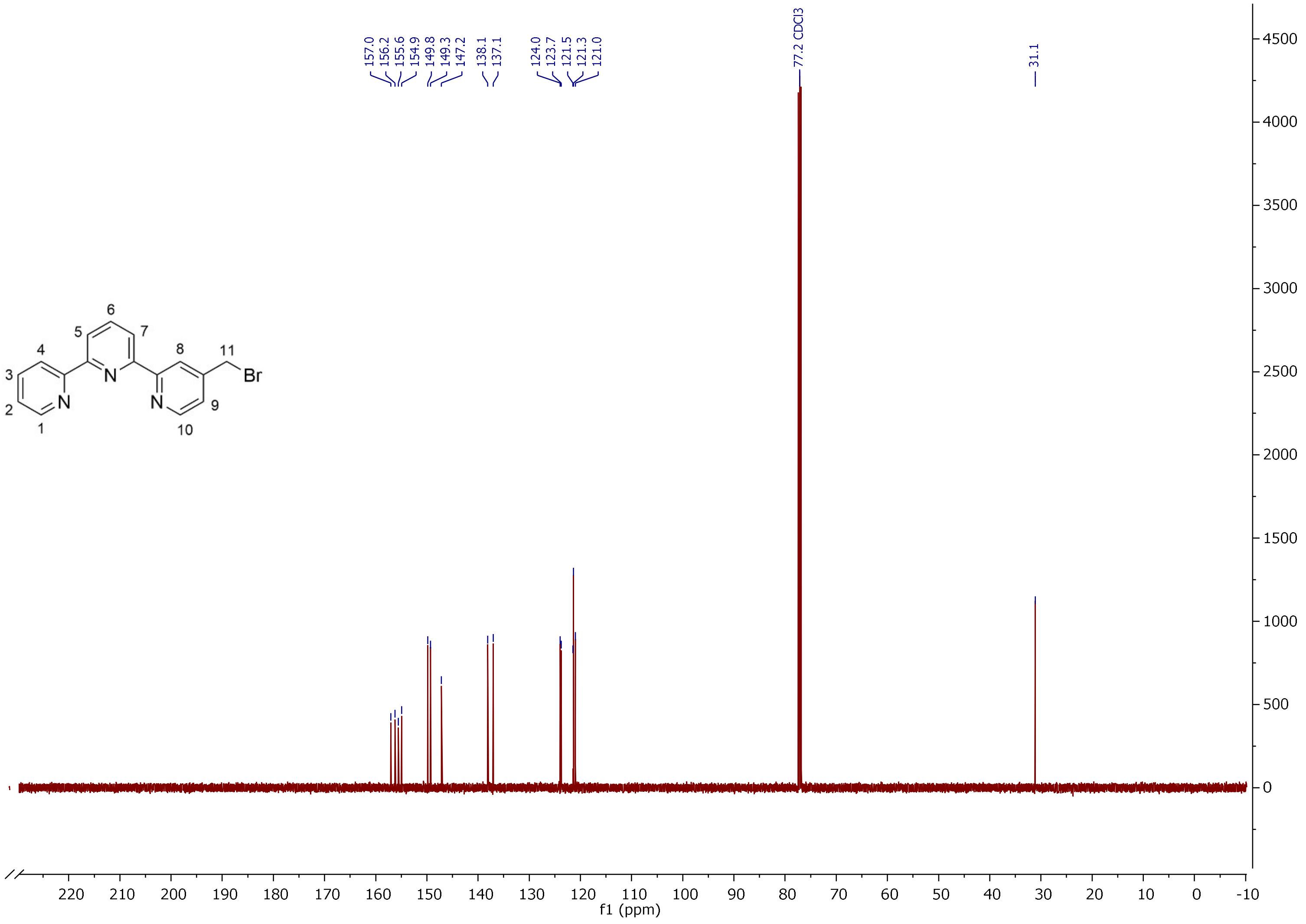

^{1}H NMR (500 MHz, $CDCl_3$) for tetrabutylammonium [2,2':6',2"-terpyridin]-4-ylmethanesulfonate **(L1)**

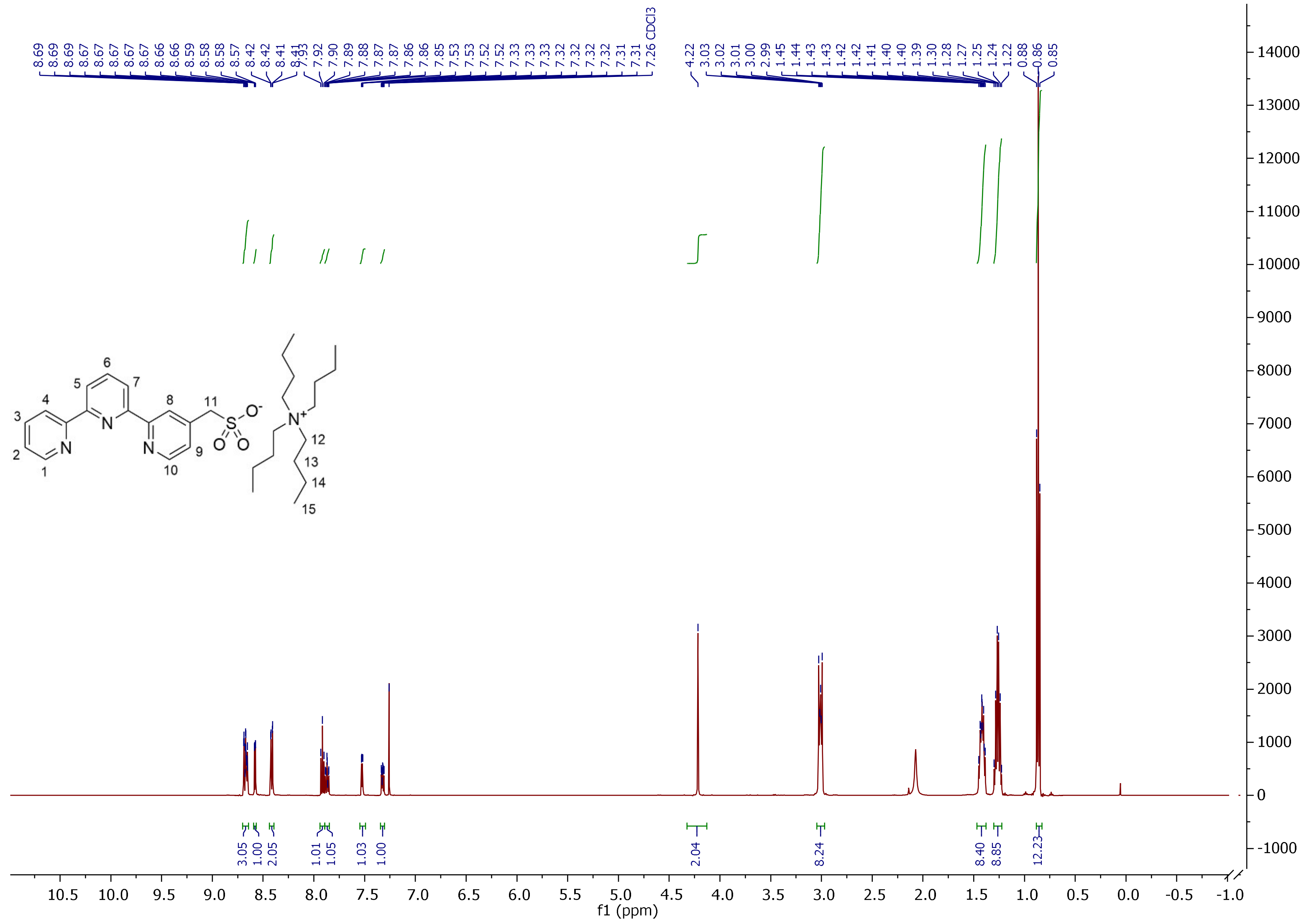

^{13}C NMR (126 MHz, $CDCl_3$) for tetrabutylammonium [2,2':6',2"-terpyridin]-4-ylmethanesulfonate **(L1)**

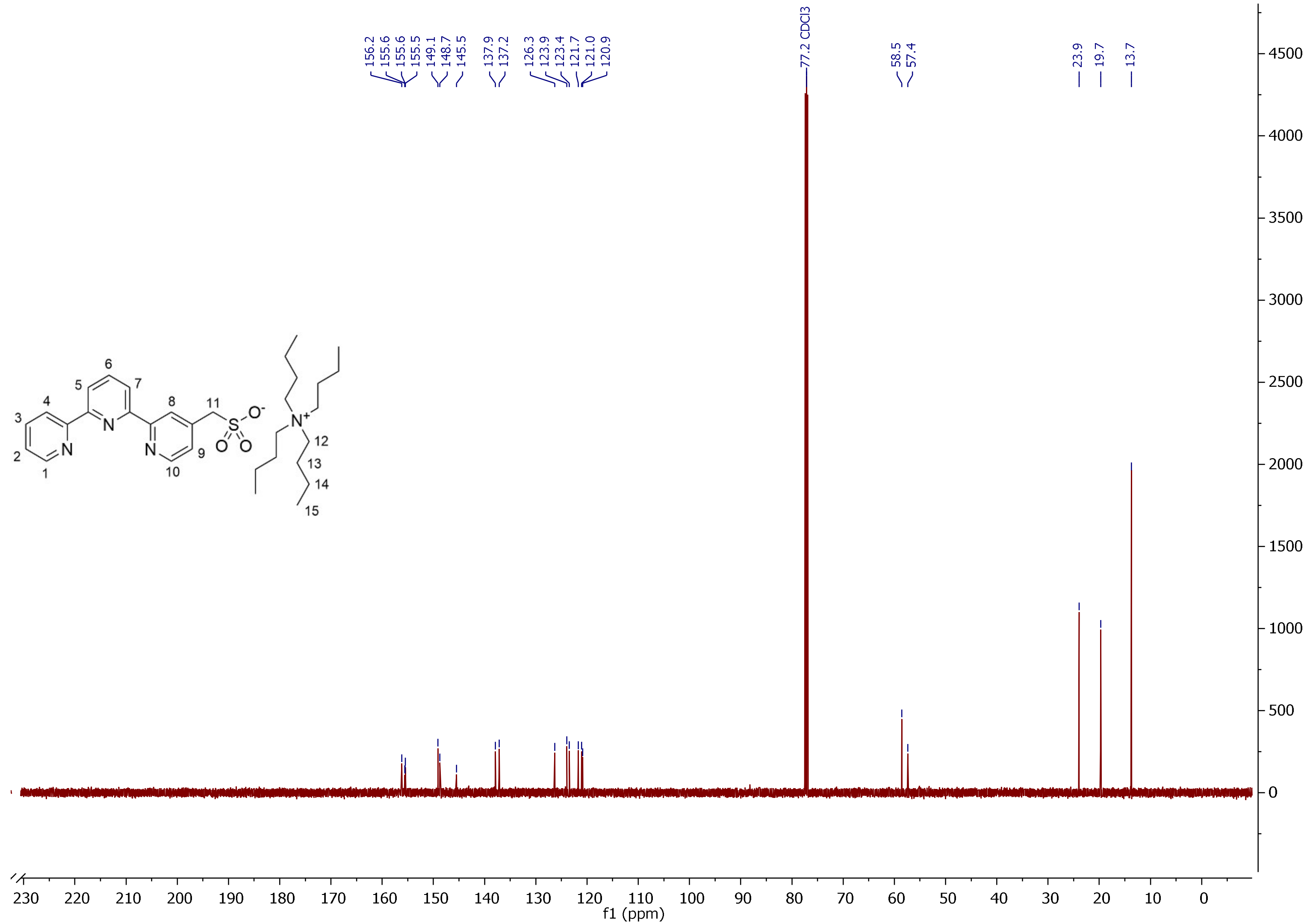

¹H NMR (500 MHz, $CDCl_3$) for (6-bromopyridin-3-yl)methanol

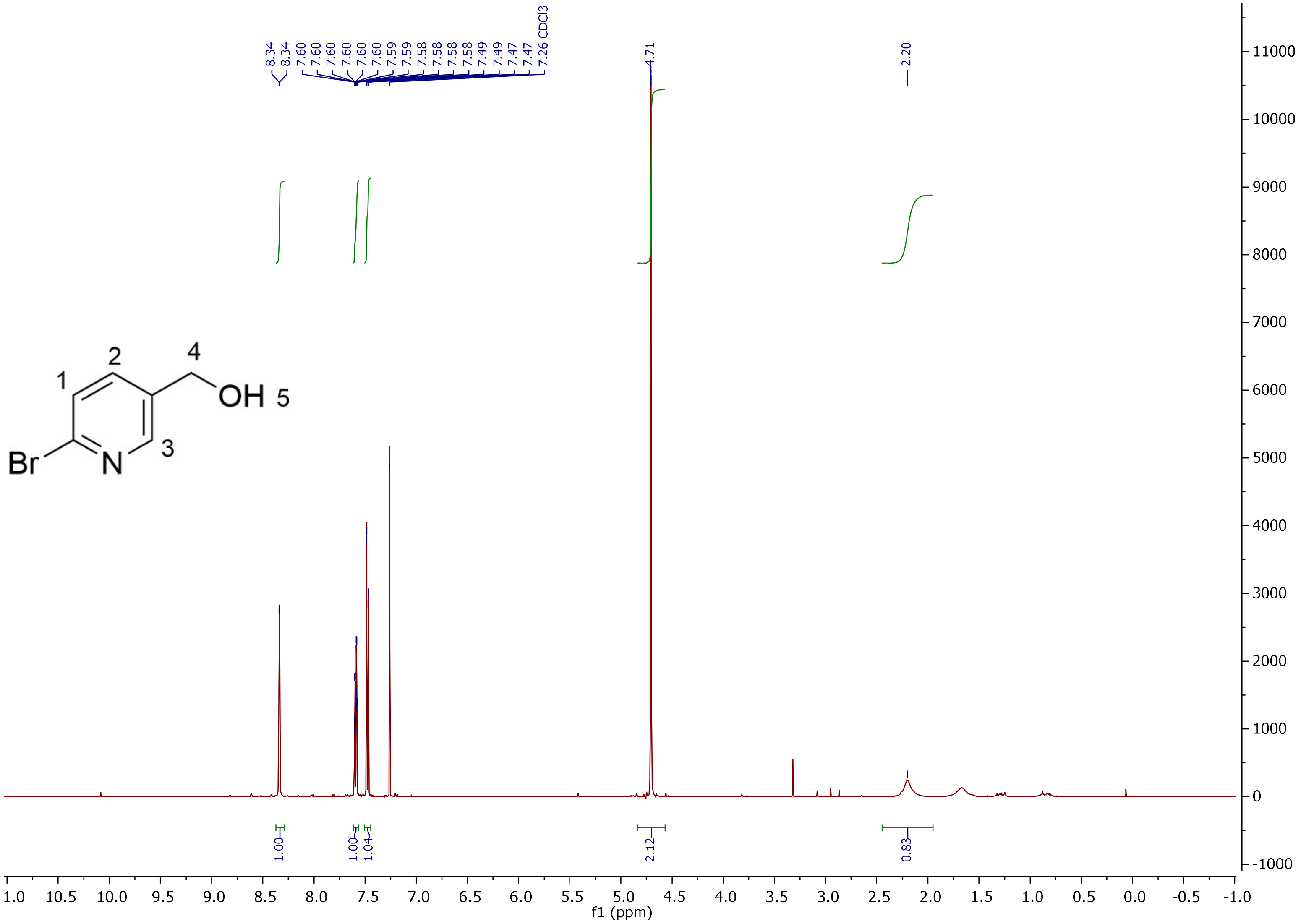

^{13}C NMR (126 MHz, $CDCl_3$) for (6-bromopyridin-3-yl)methanol

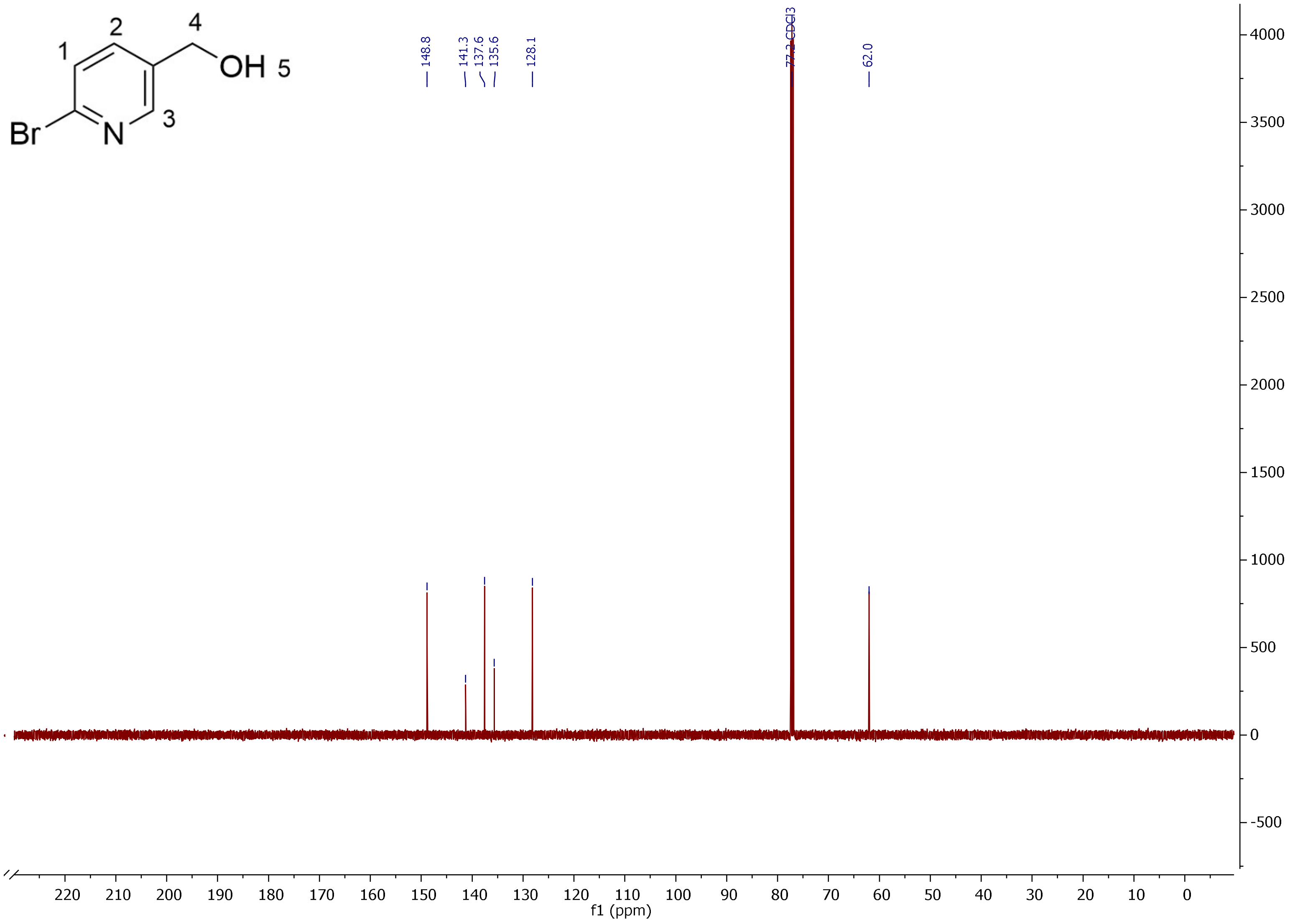

^{1}H NMR (500 MHz, $CDCl_3$) for 2-bromo-5-(((tetrahydro-2H-pyran-2-yl)oxy)methyl)pyridine

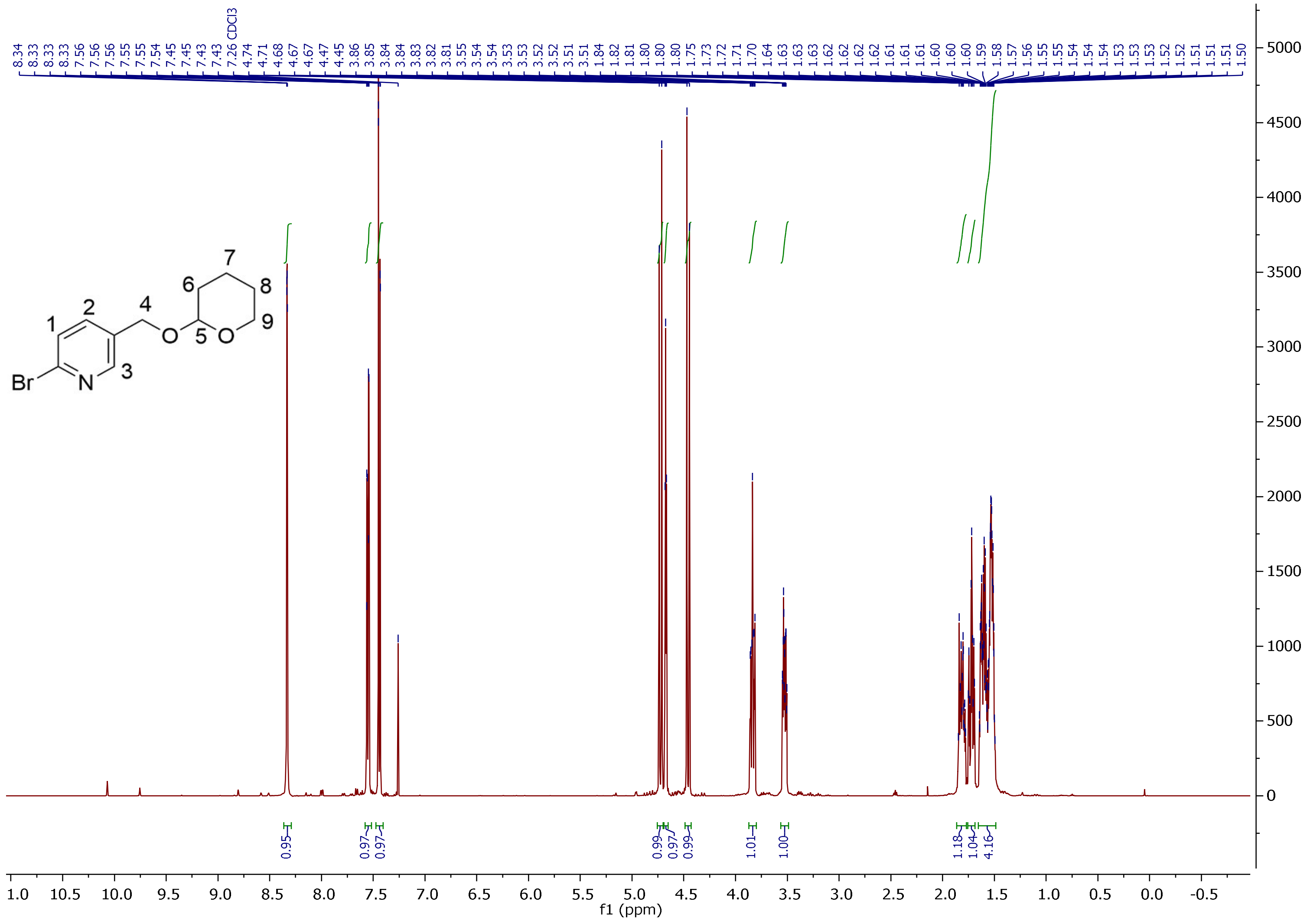

^{13}C NMR (126 MHz, $CDCl_3$) for 2-bromo-5-(((tetrahydro-2H-pyran-2-yl)oxy)methyl)pyridine

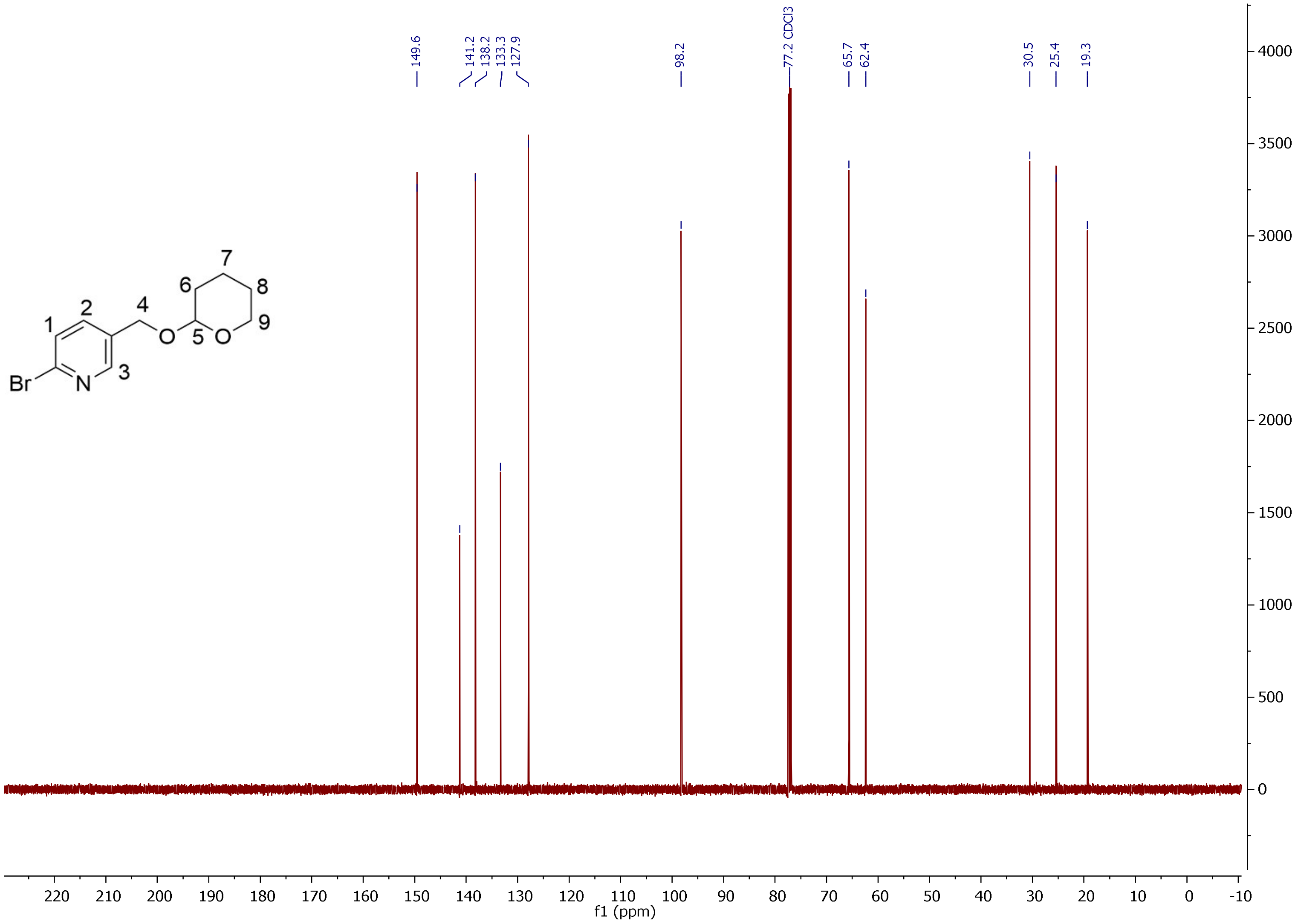

[1]**H NMR** (500 MHz, $CDCl_3$) for 5-(((tetrahydro-2H-pyran-2-yl)oxy)methyl)-2,2':6',2"-terpyridine

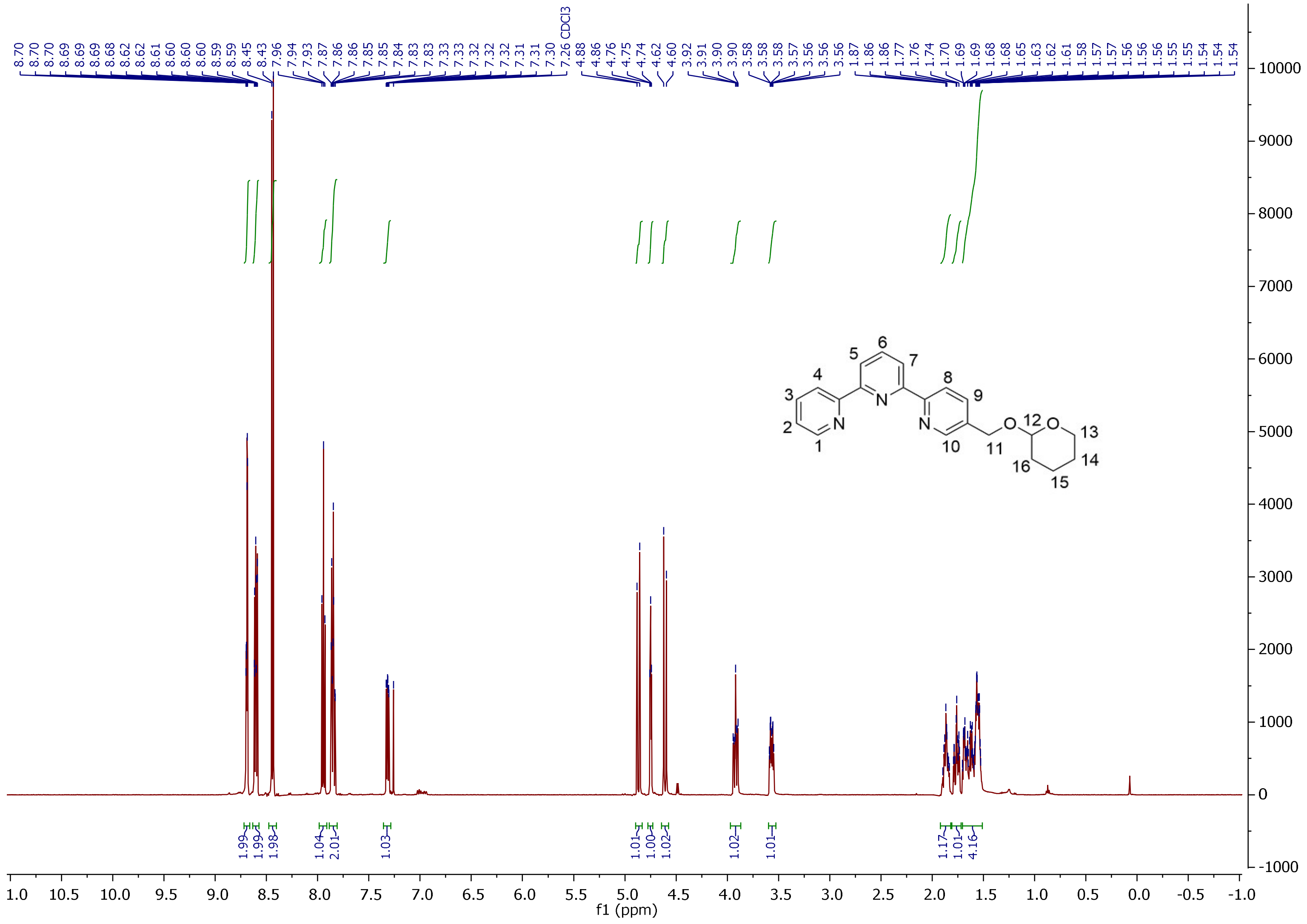

^{13}**C NMR** (126 MHz, $CDCl_3$) for 5-(((tetrahydro-2H-pyran-2-yl)oxy)methyl)-2,2':6',2"-terpyridine

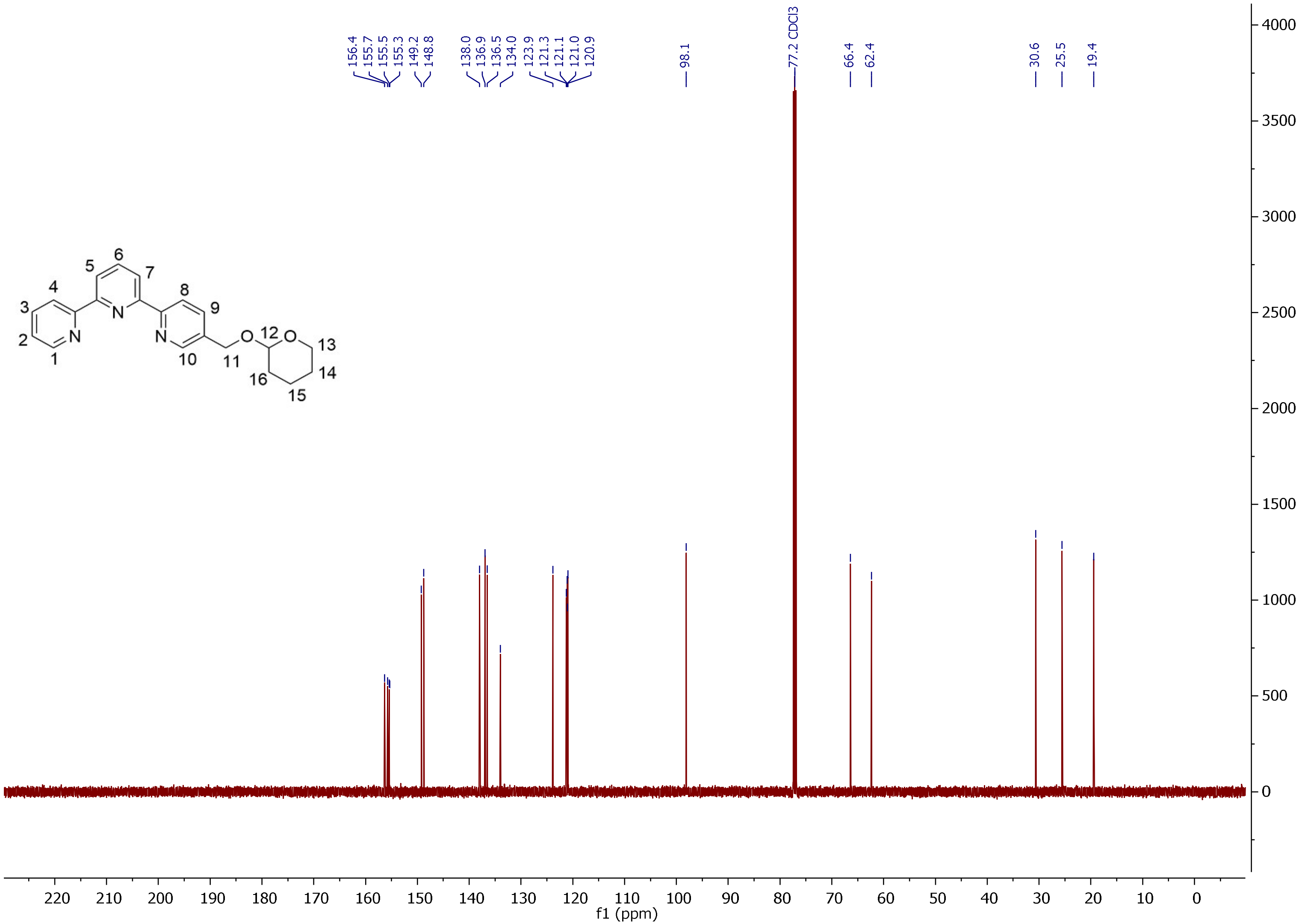

^{1}H NMR (500 MHz, $CDCl_3$) for 5-(bromomethyl)-2,2':6',2"-terpyridine

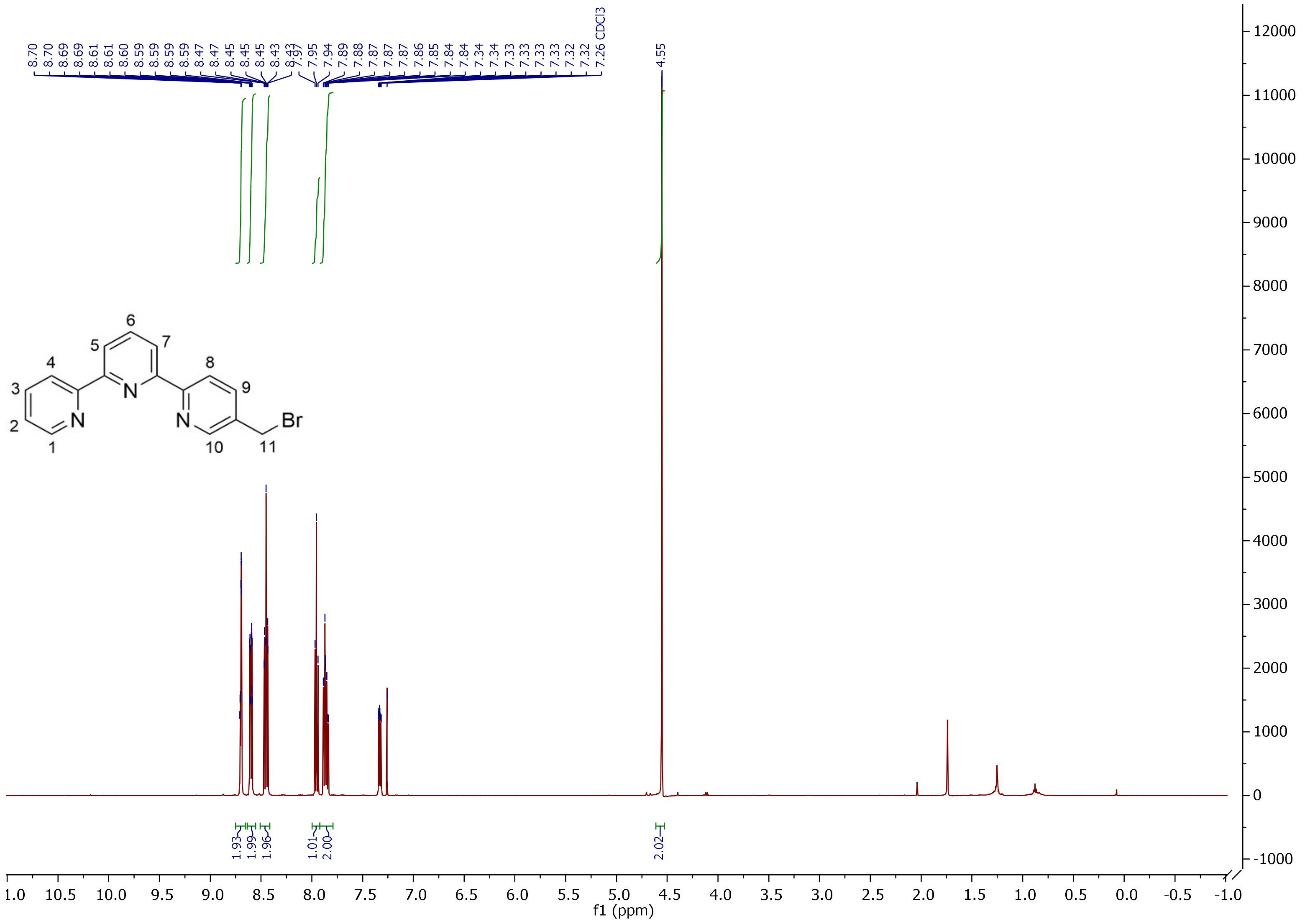

^{13}C NMR (126 MHz, $CDCl_3$) for 5-(bromomethyl)-2,2':6',2"-terpyridine

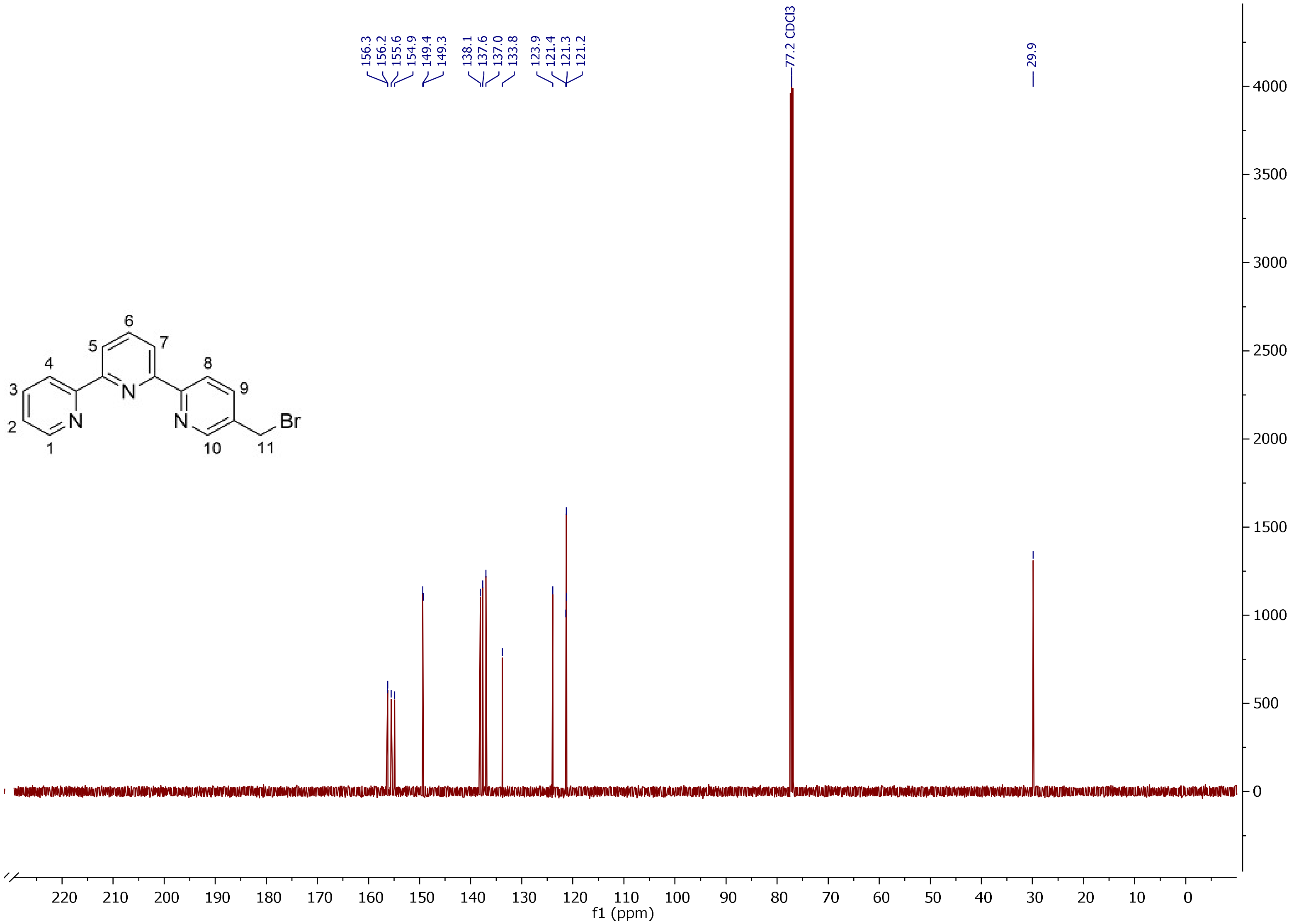

^{1}H NMR (400 MHz, $CDCl_3$) for tetrabutylammonium [2,2':6',2"-terpyridin]-5-ylmethanesulfonate **(L2)**

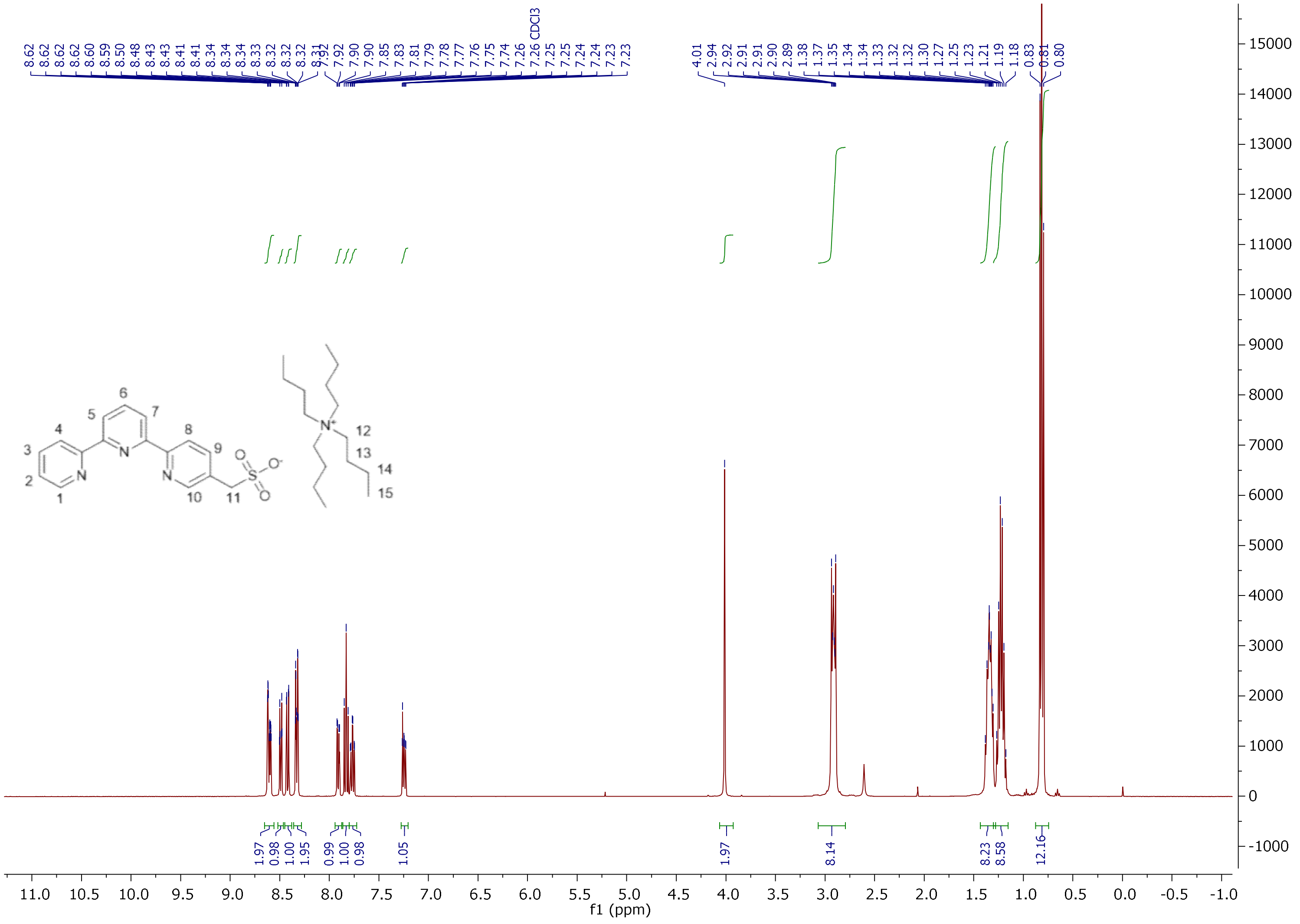

^{13}C NMR (101 MHz, $CDCl_3$) for tetrabutylammonium [2,2':6',2"-terpyridin]-5-ylmethanesulfonate **(L2)**

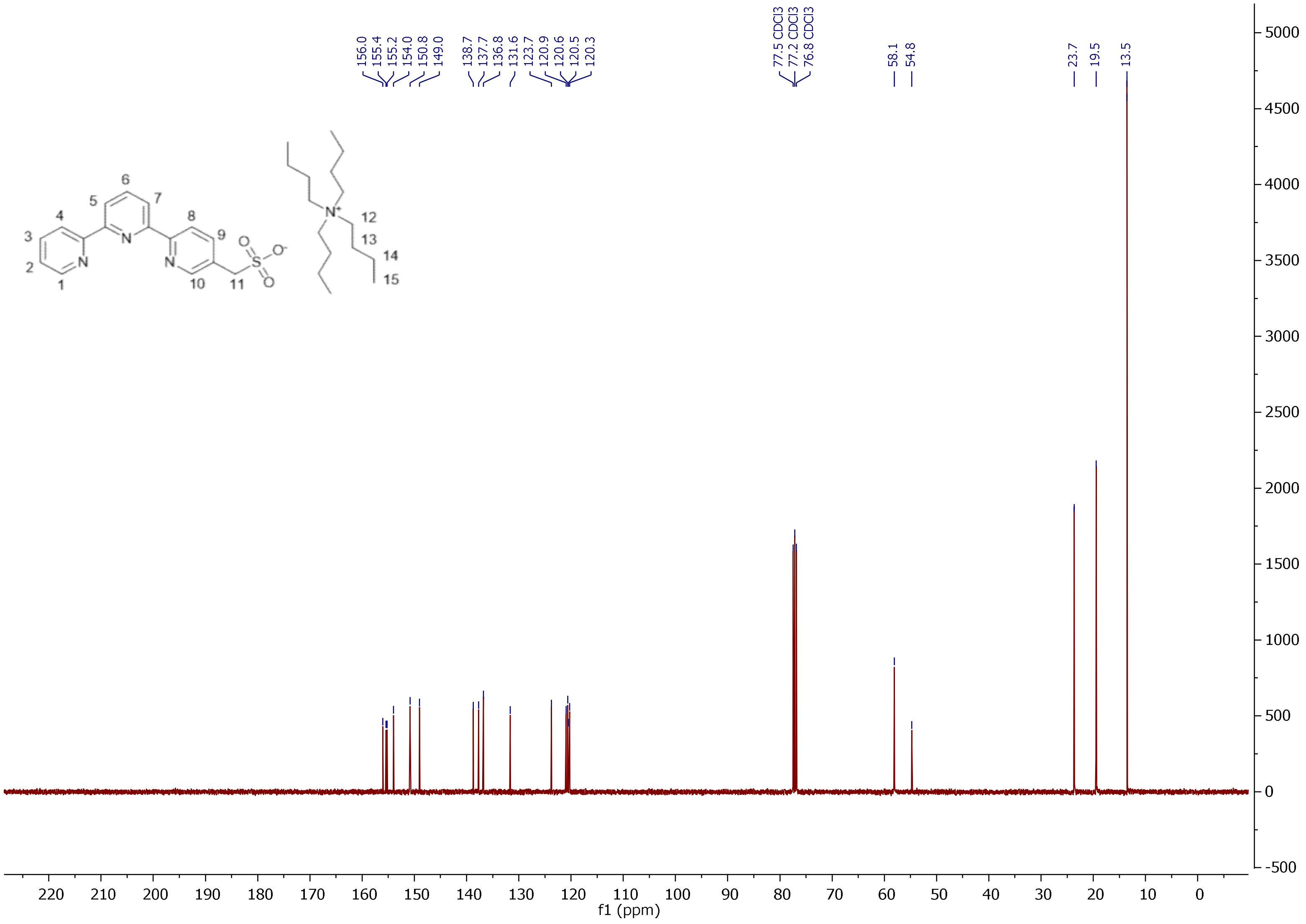

¹H NMR (500 MHz, $CDCl_3$) for 6-methyl-2,2':6',2"-terpyridine

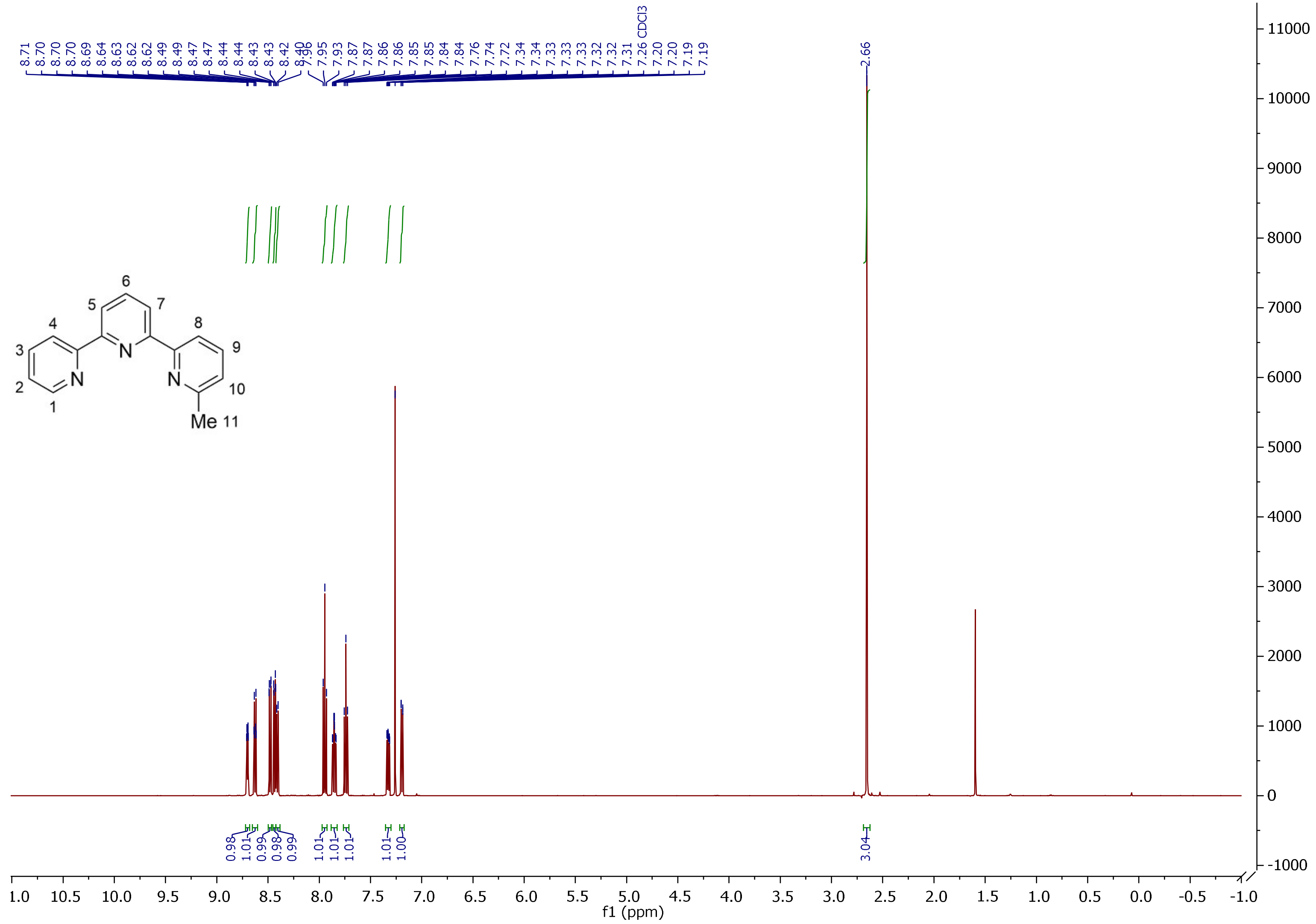

^{13}C NMR (126 MHz, $CDCl_3$) for 6-methyl-2,2':6',2"-terpyridine

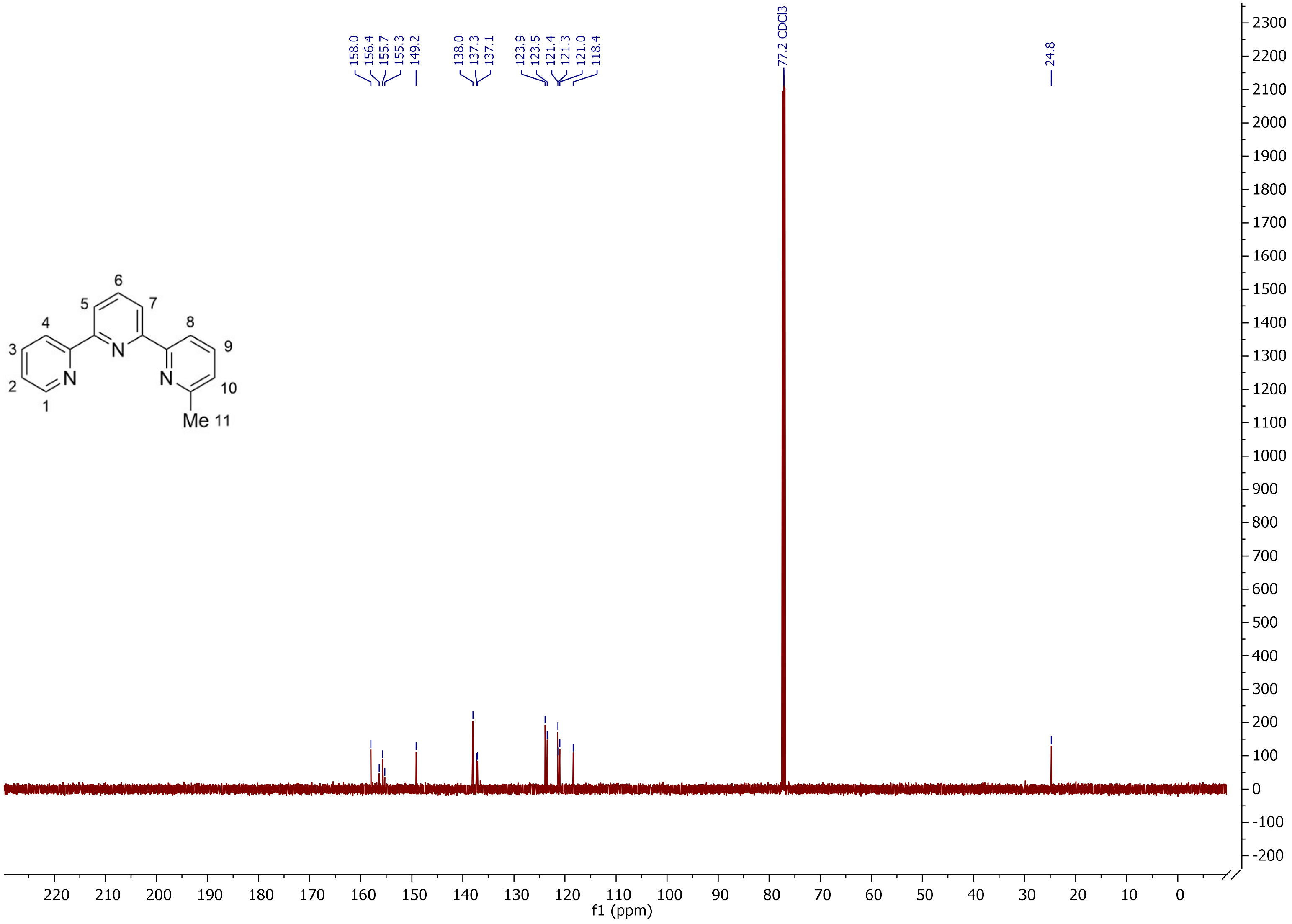

¹H NMR (500 MHz, $CDCl_3$) for [2,2':6',2"-terpyridin]-6-ylmethanol

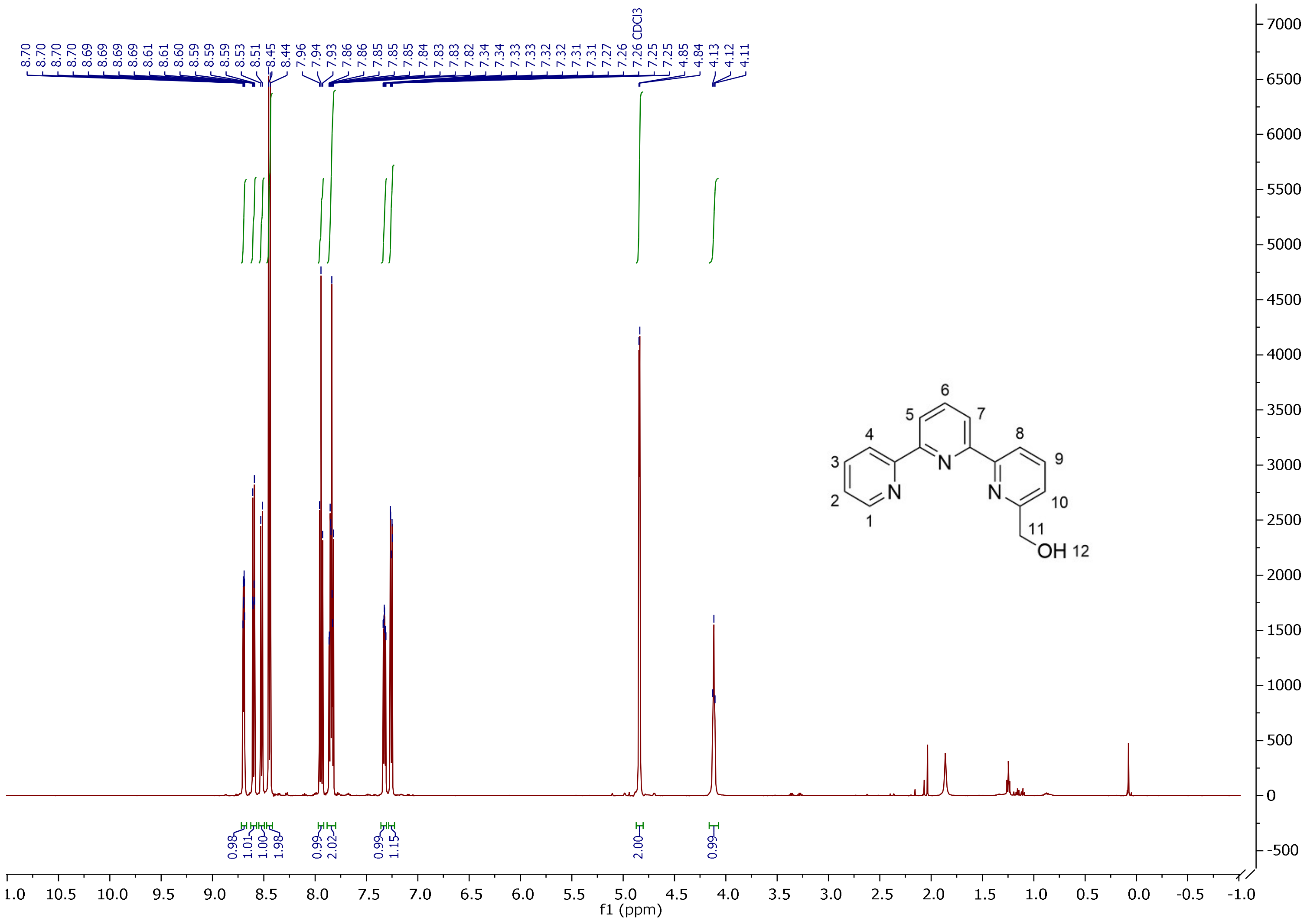

^{13}C NMR (126 MHz, $CDCl_3$) for [2,2':6',2''-terpyridin]-6-ylmethanol

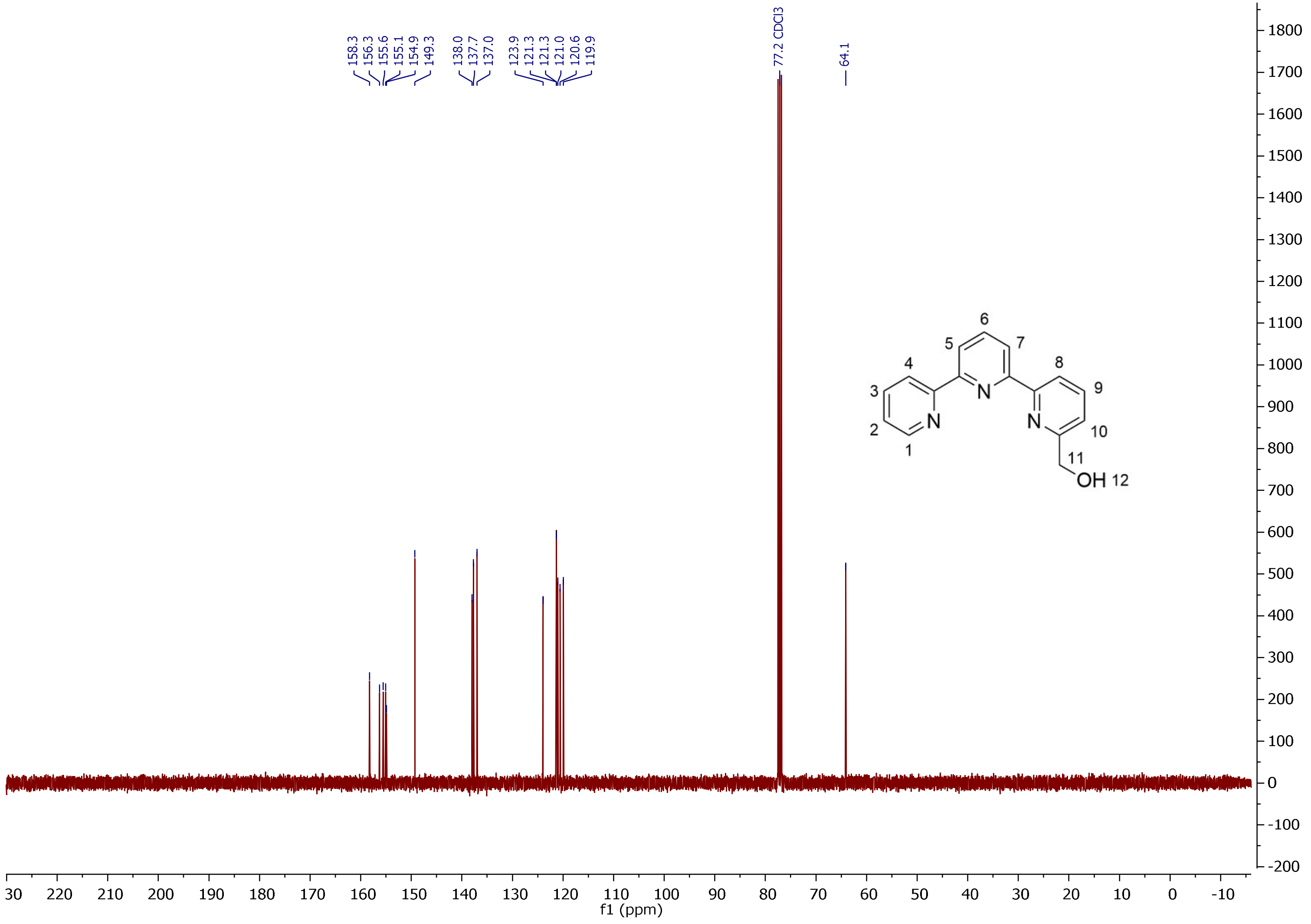

^{1}H NMR (500 MHz, $CDCl_3$) for 4-(bromomethyl)-2,2':6',2"-terpyridine

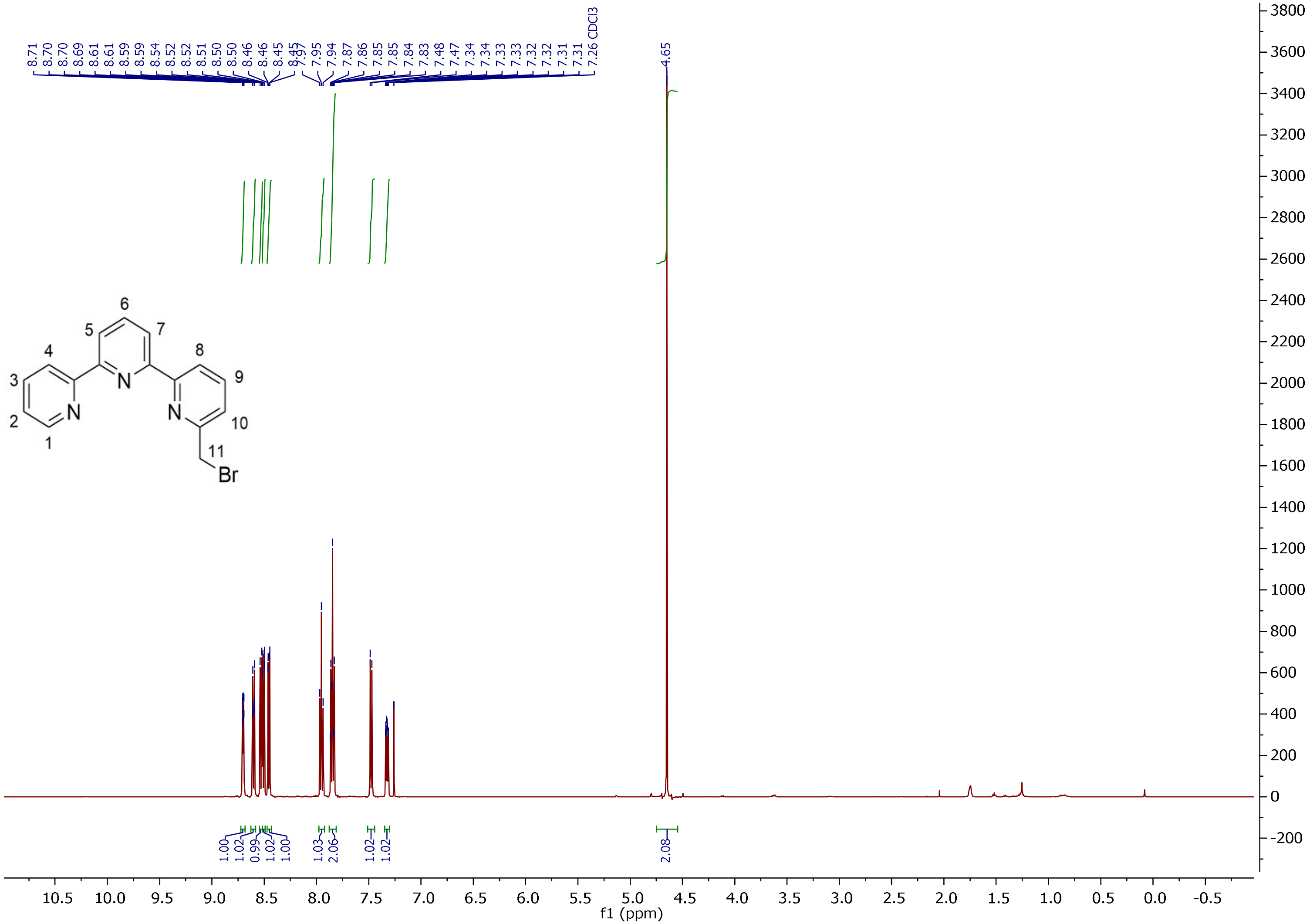

^{13}C NMR (126 MHz, $CDCl_3$) for 4-(bromomethyl)-2,2':6',2"-terpyridine

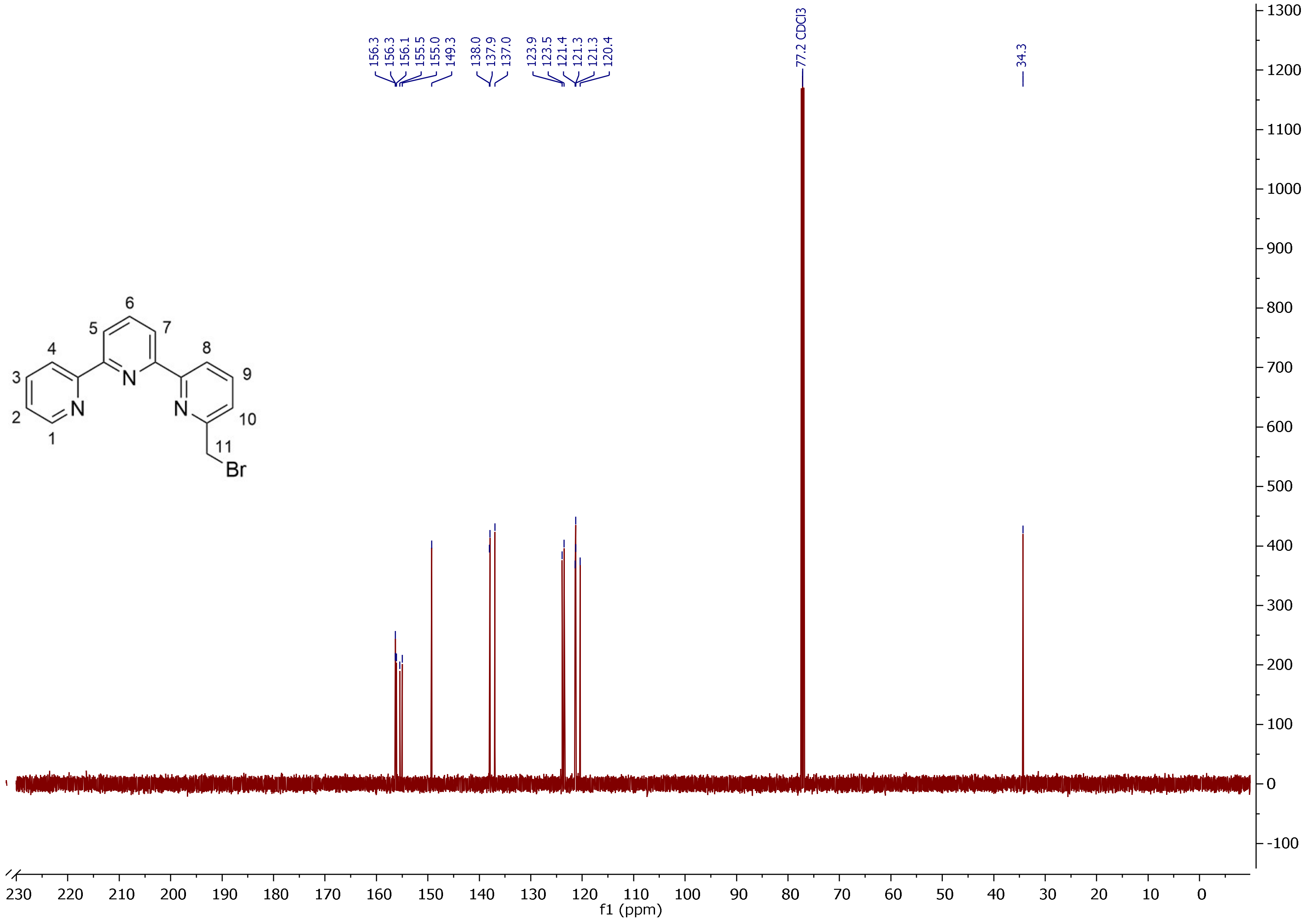

^{1}H NMR (500 MHz, $CDCl_3$) for tetrabutylammonium [2,2':6',2"-terpyridin]-6-ylmethanesulfonate **(L3)**

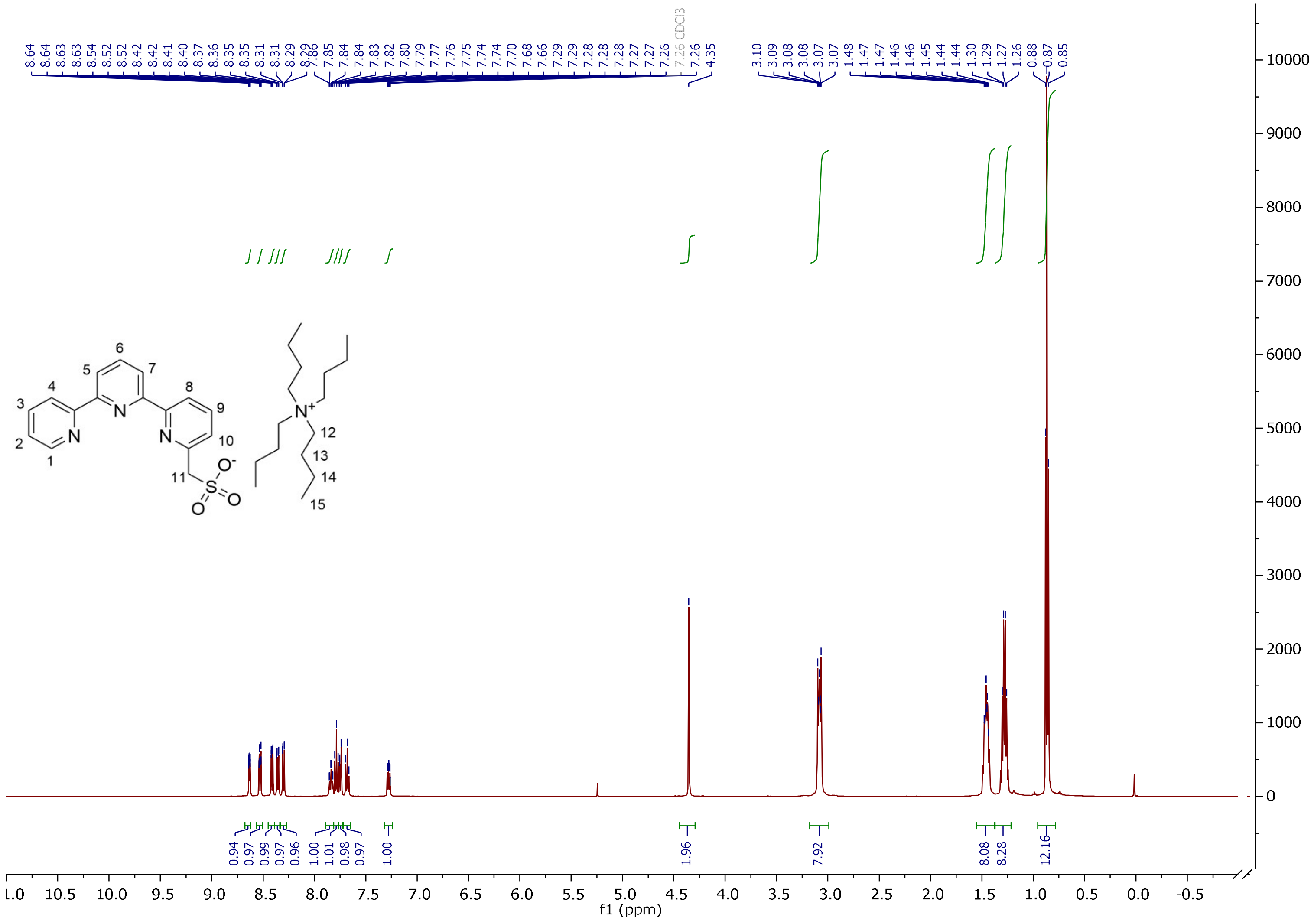

^{13}C NMR (126 MHz, $CDCl_3$) for tetrabutylammonium [2,2':6',2"-terpyridin]-6-ylmethanesulfonate **(L3)**

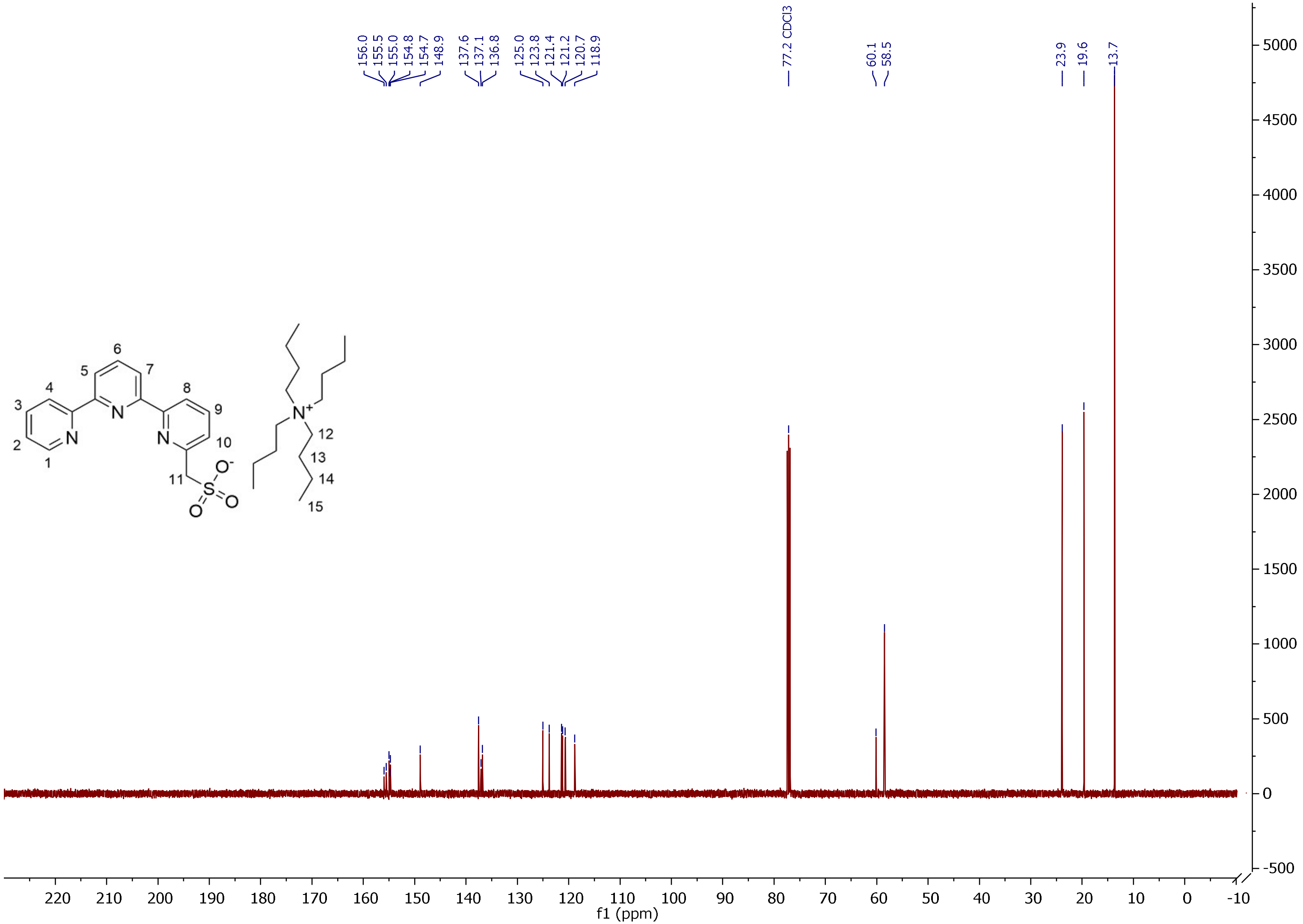

^{1}H NMR (500 MHz, $CDCl_3$) 2,6-dibromo-4-(((tetrahydro-2H-pyran-2-yl)oxy)methyl)pyridine

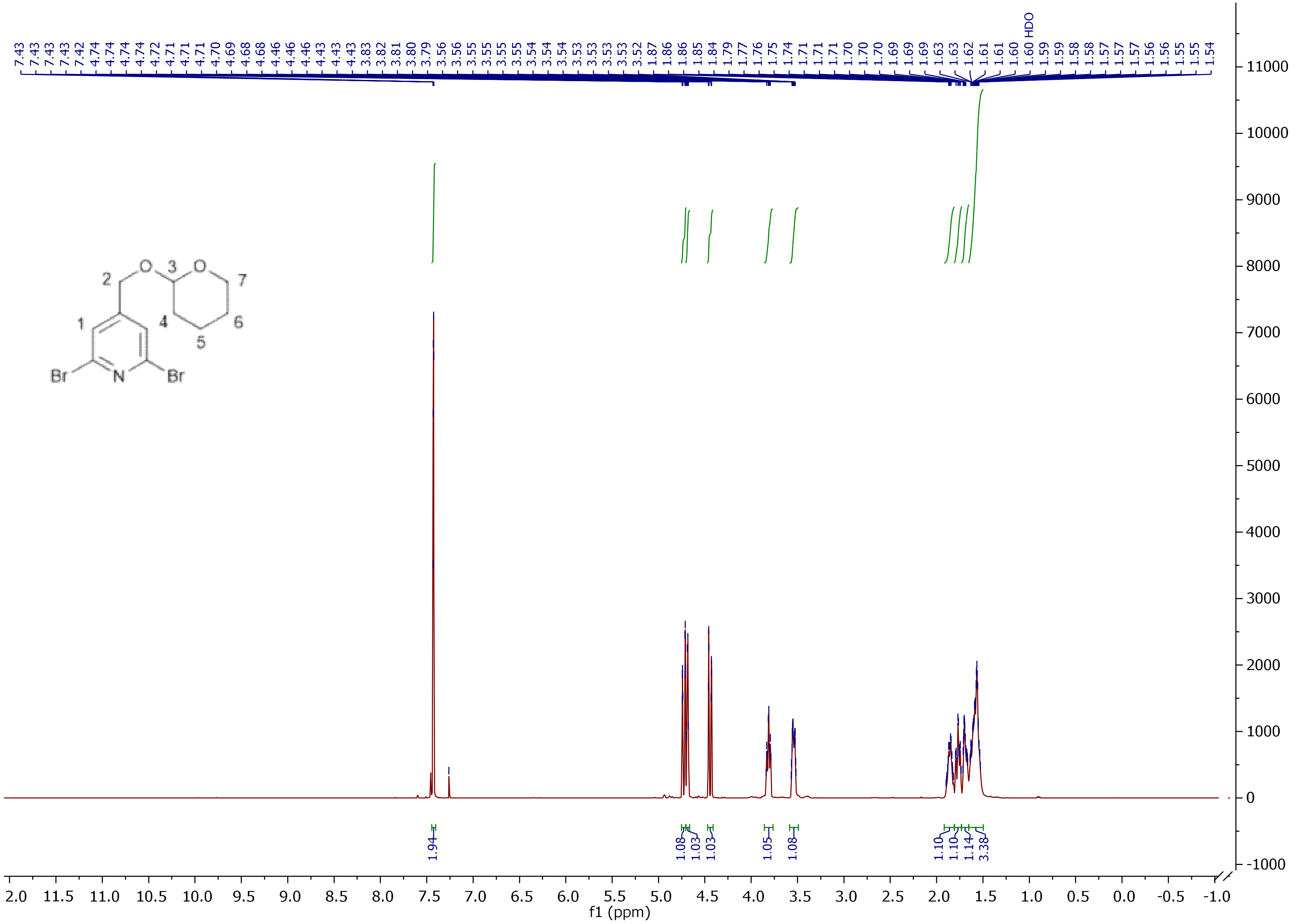

^{13}C NMR (126 MHz, $CDCl_3$) 2,6-dibromo-4-(((tetrahydro-2H-pyran-2-yl)oxy)methyl)pyridine

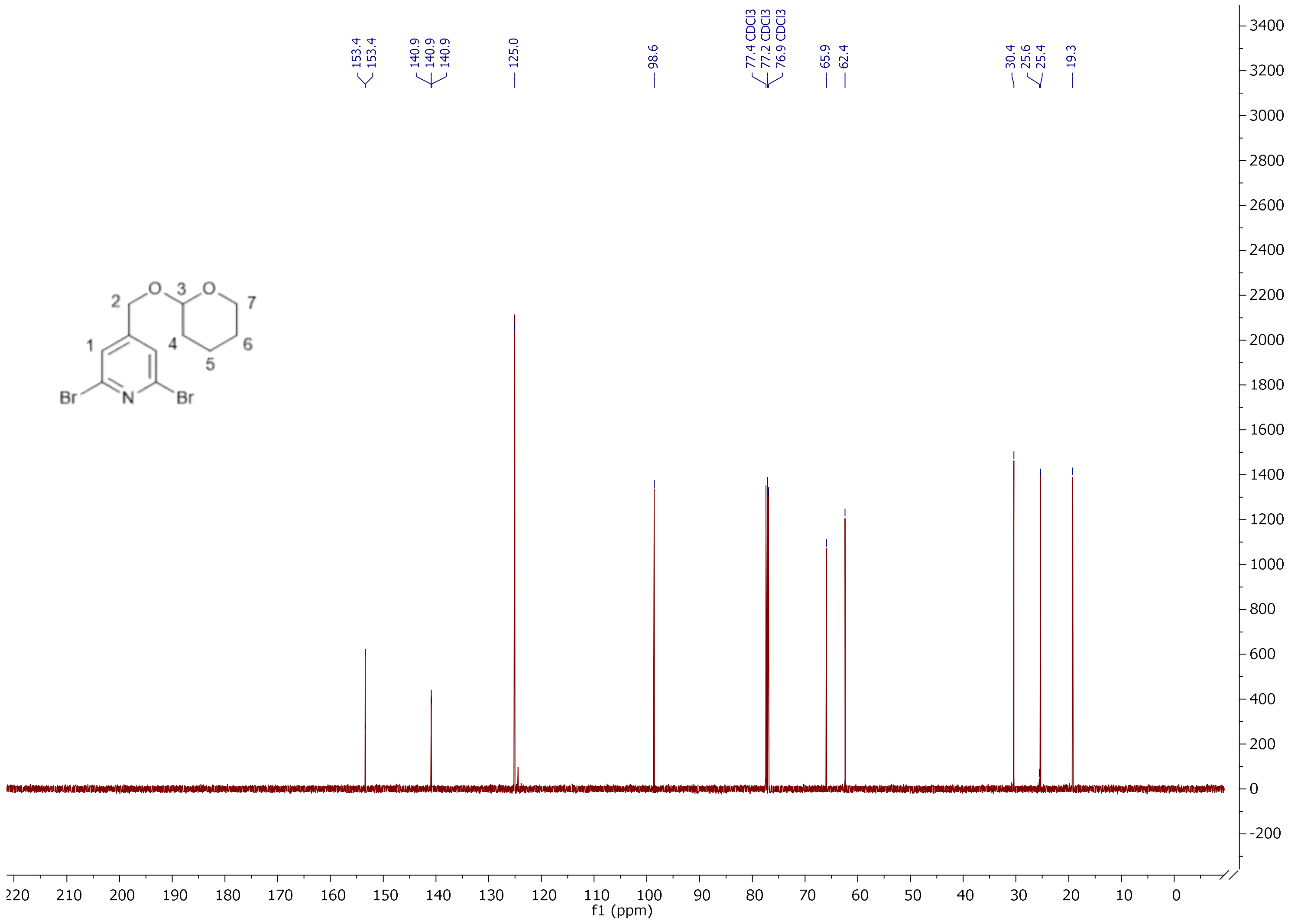

^{1}H NMR (400 MHz, $CDCl_3$) for 4'-(((tetrahydro-2H-pyran-2-yl)oxy)methyl)-2,2':6',2"-terpyridine

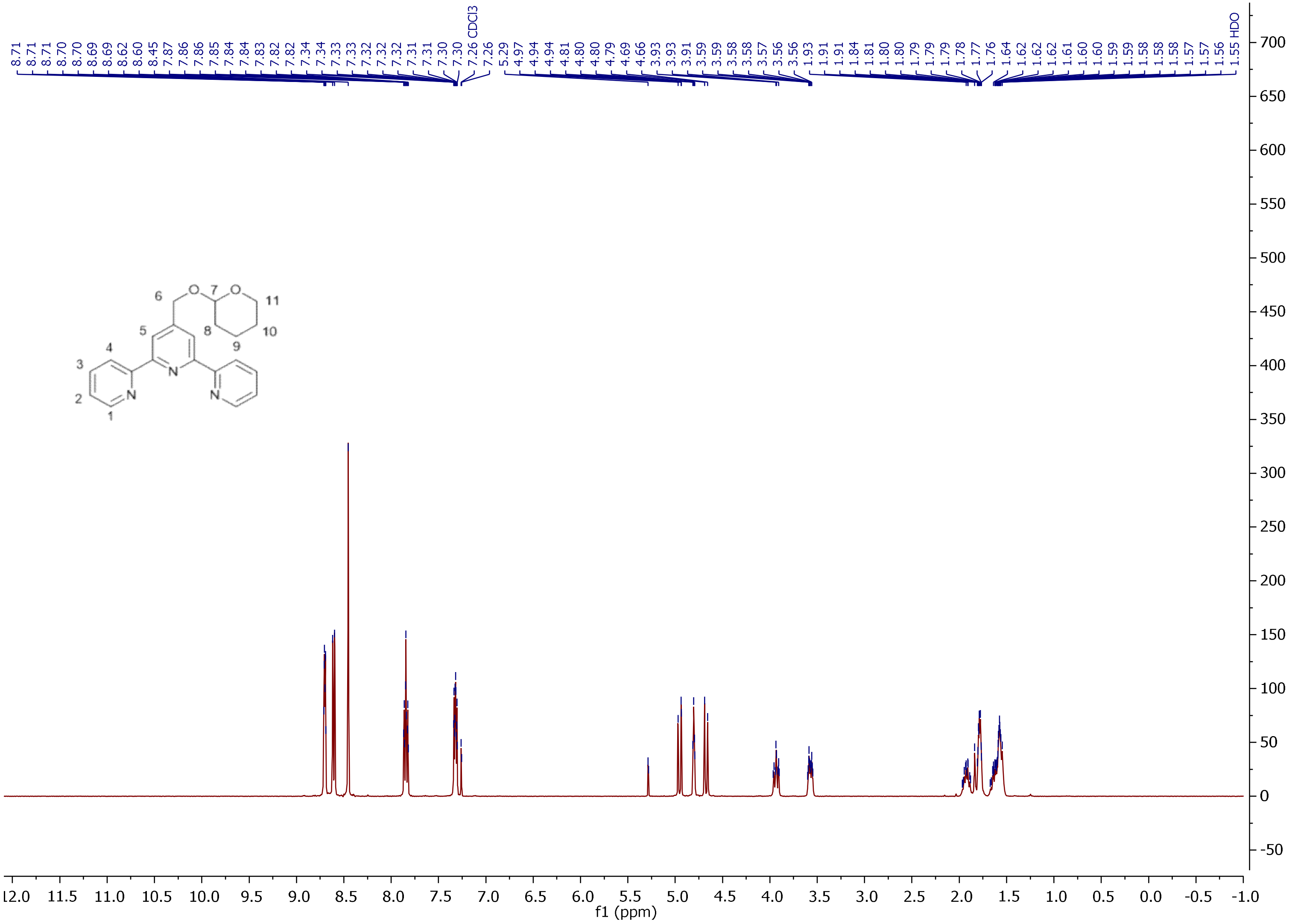

^{13}**C NMR** (101 MHz, $CDCl_3$) for 4'-(((tetrahydro-2H-pyran-2-yl)oxy)methyl)-2,2':6',2''-terpyridine

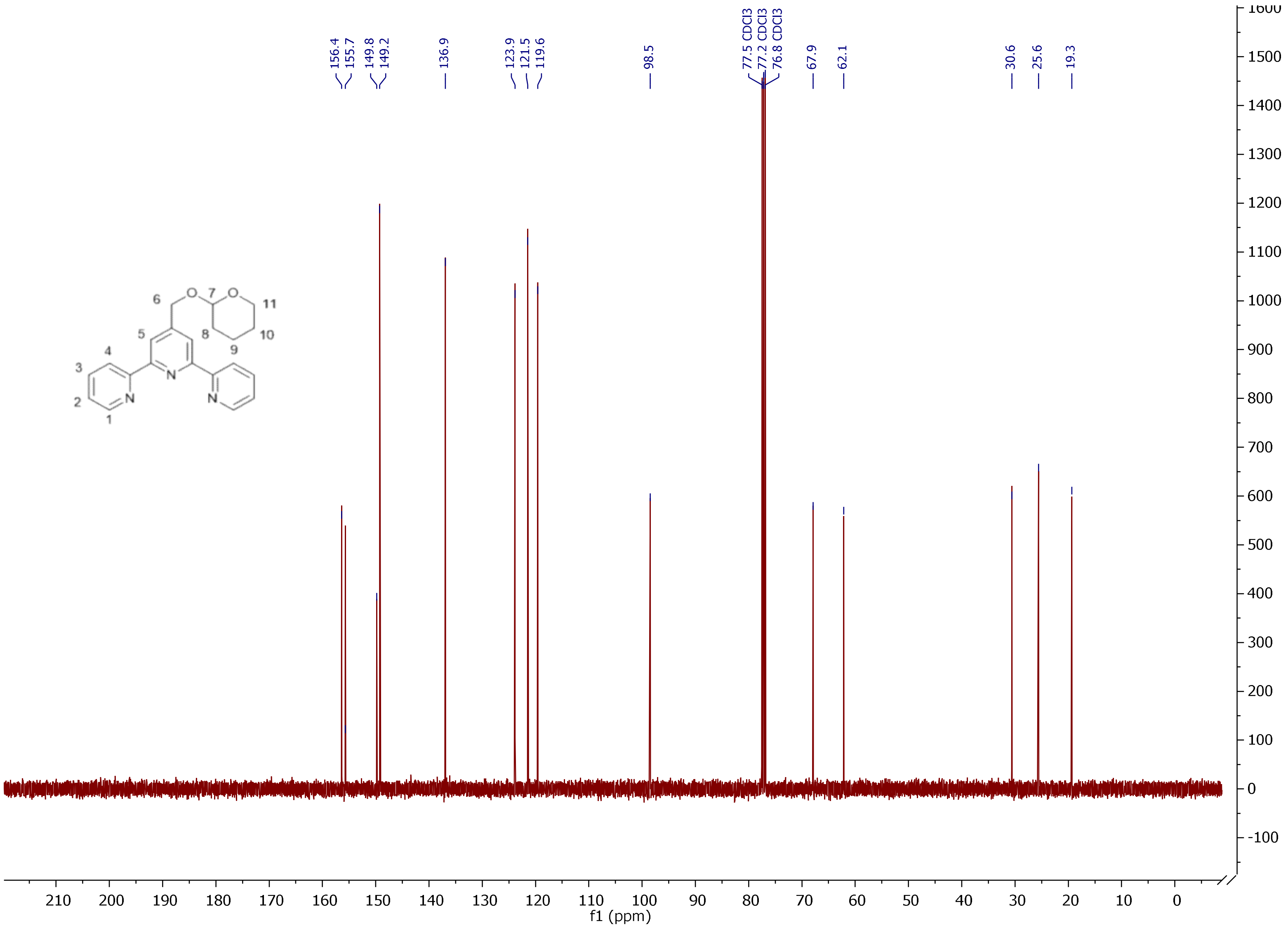

^{1}H NMR (500 MHz, $CDCl_3$) for 4'-(bromomethyl)-2,2':6',2"-terpyridine

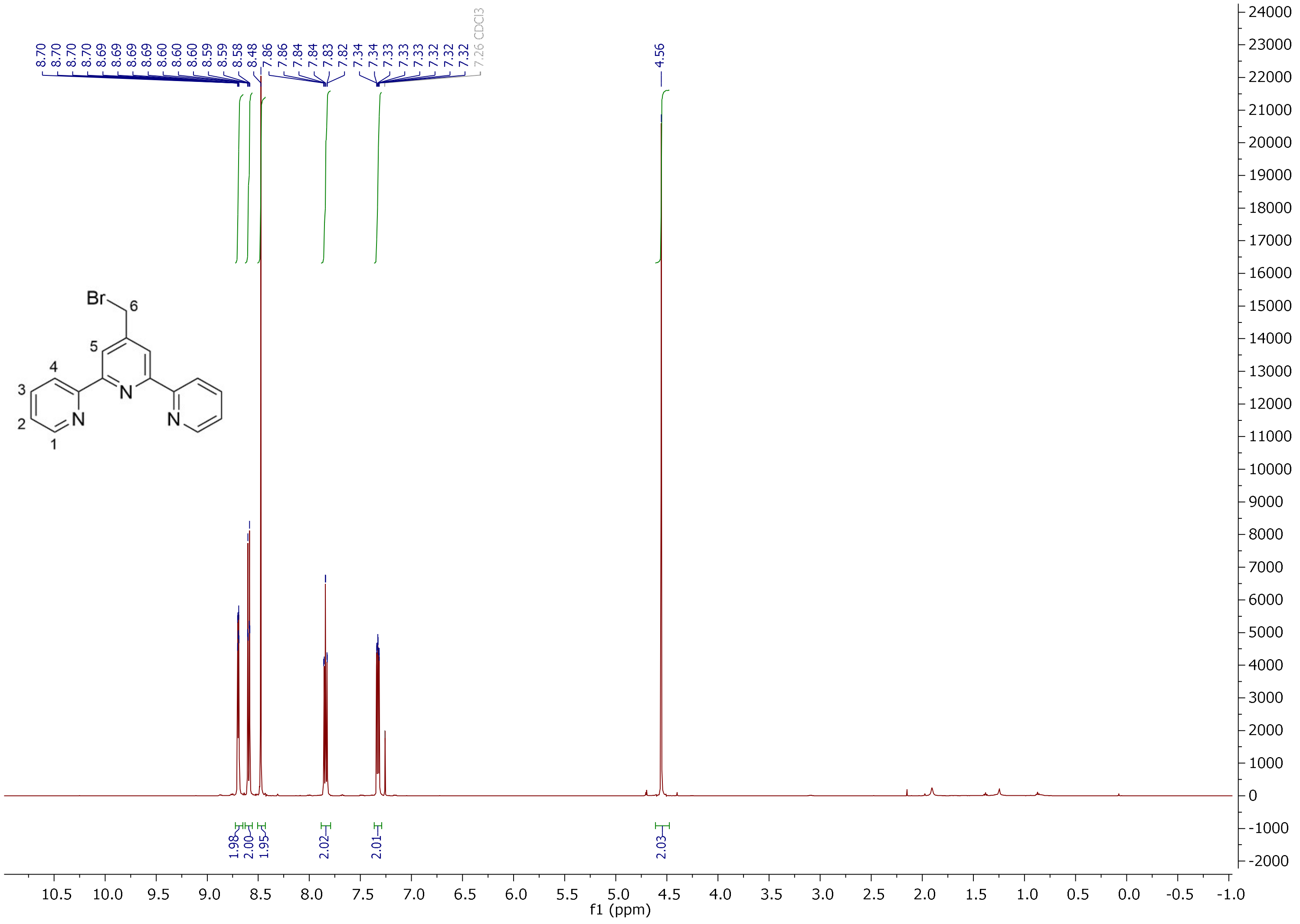

^{13}C NMR (126 MHz, $CDCl_3$) for 4'-(bromomethyl)-2,2':6',2"-terpyridine

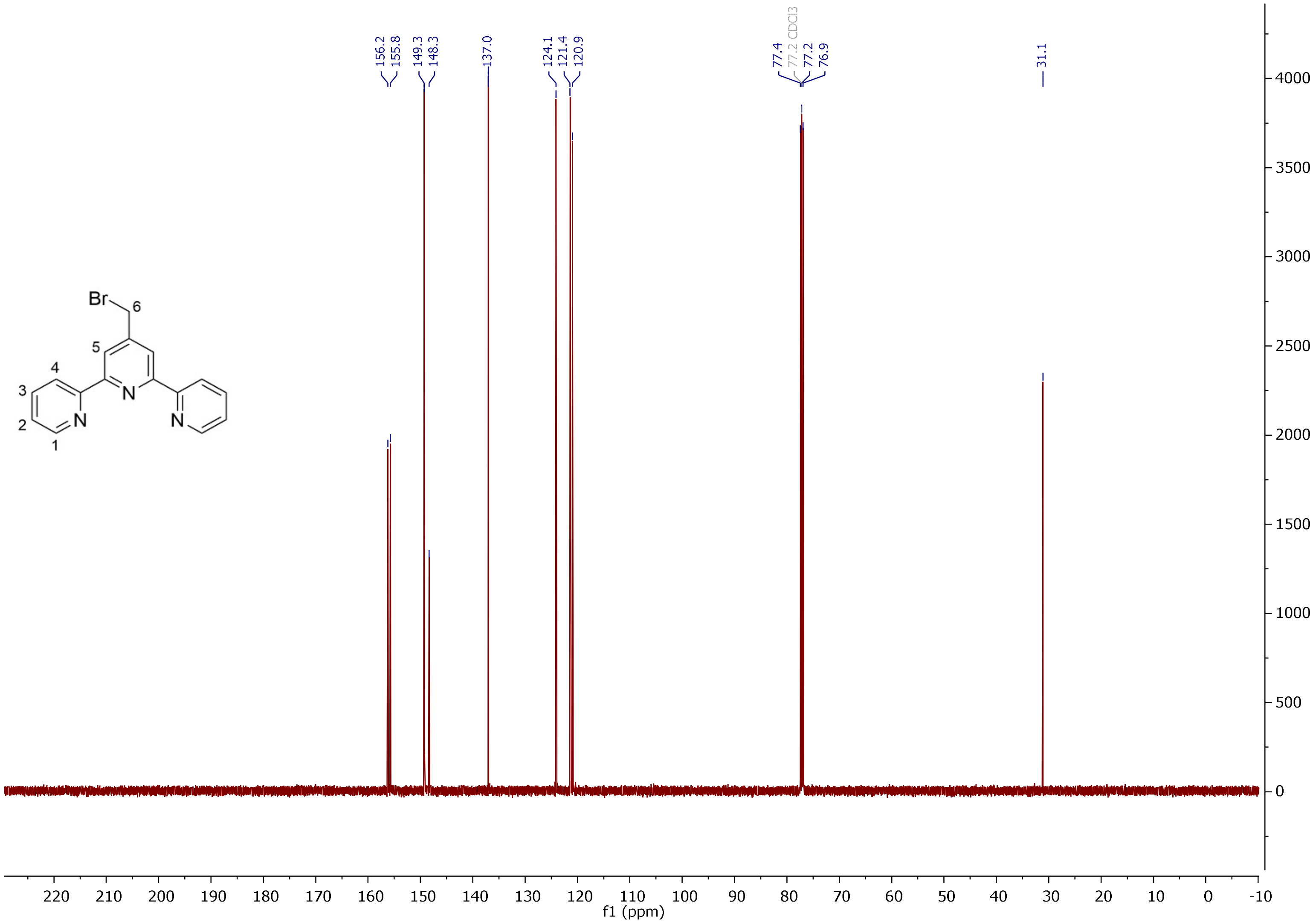

^{1}H NMR (500 MHz, $CDCl_3$) for tetrabutylammonium [2,2':6',2"-terpyridin]-6-ylmethanesulfonate **(L4)**

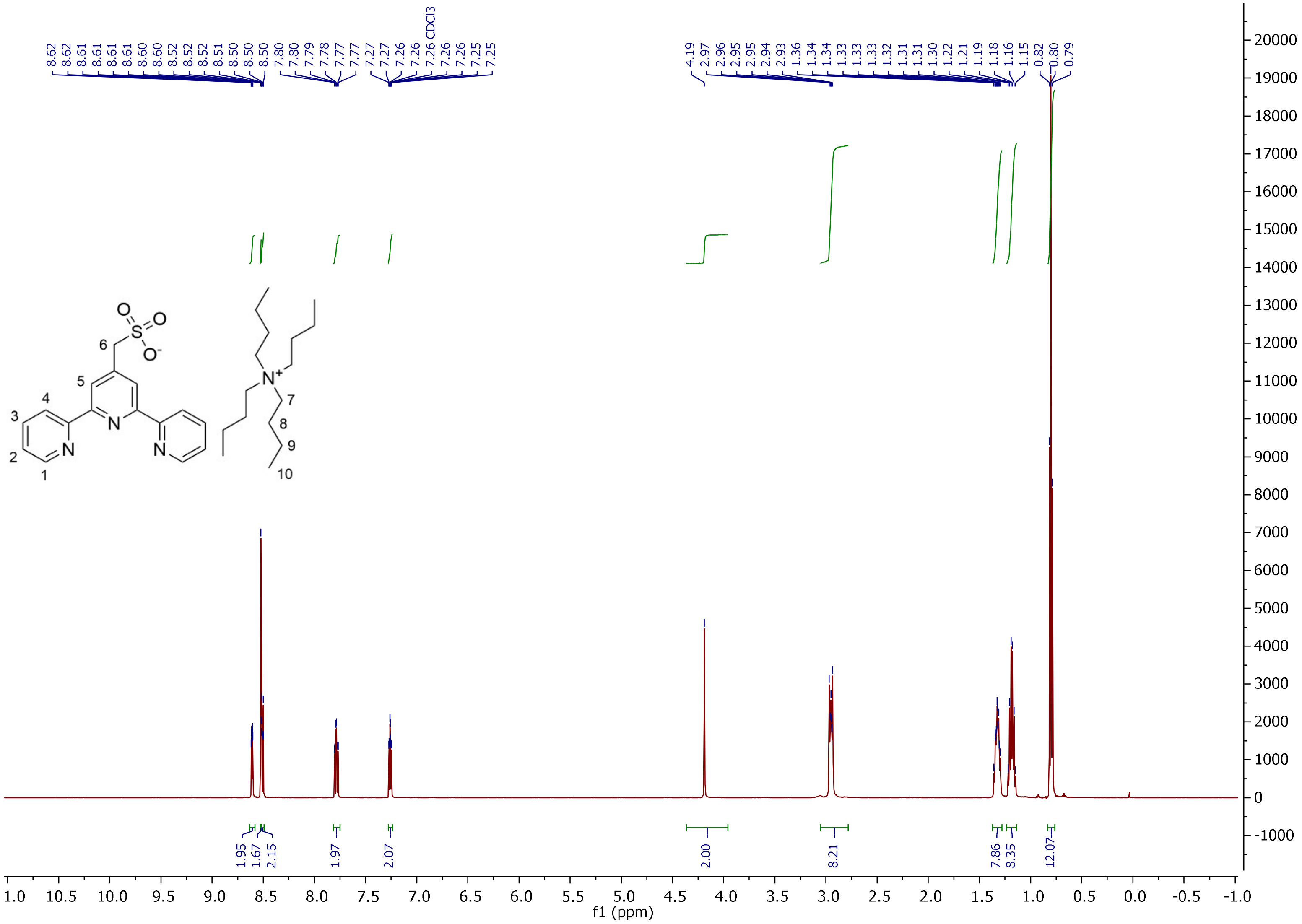

^{13}C NMR (126 MHz, $CDCl_3$) for tetrabutylammonium [2,2':6',2"-terpyridin]-6-ylmethanesulfonate **(L4)**

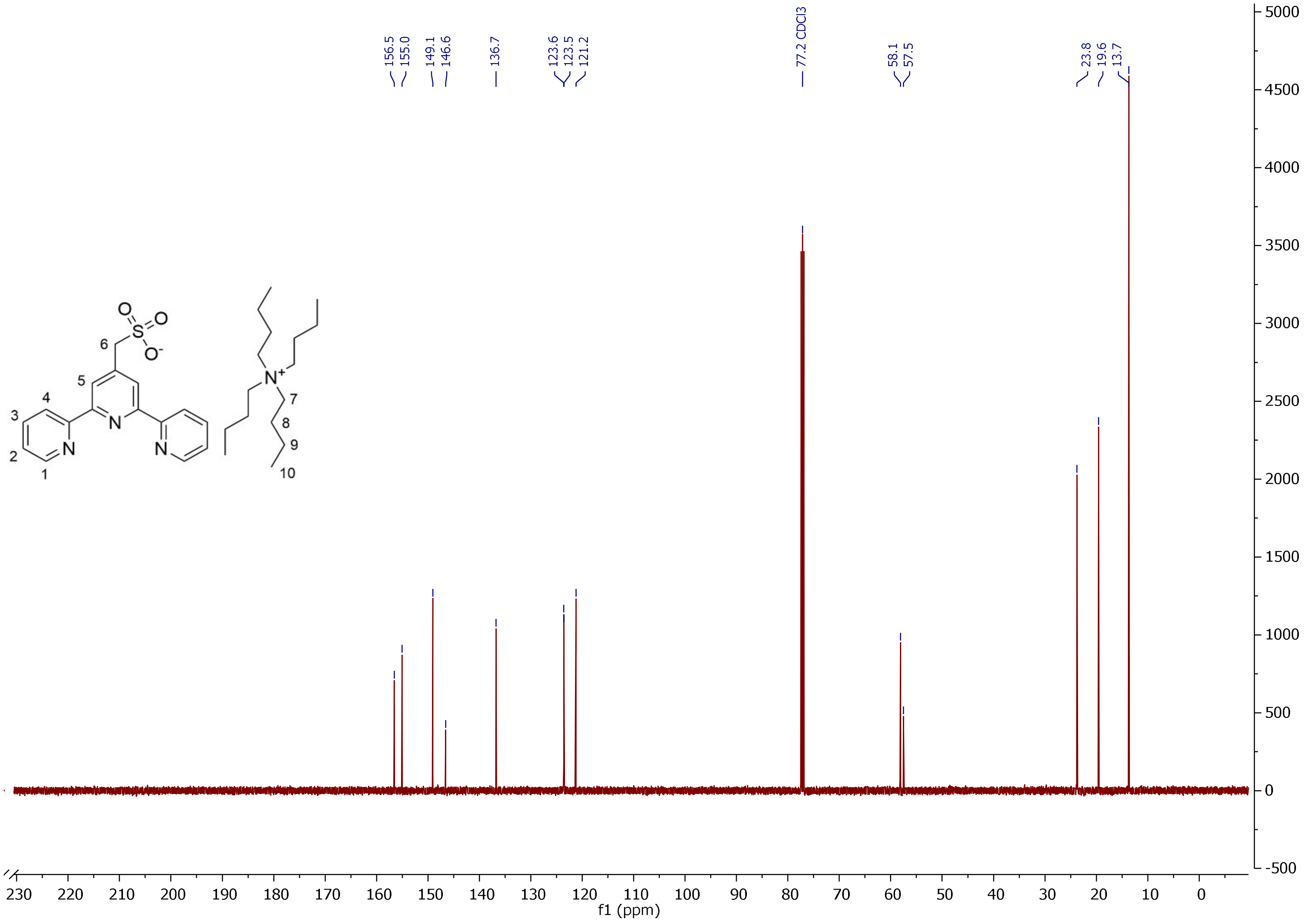

^{1}H NMR (500 MHz, $CDCl_3$) for 4'-methyl-2,2':6',2''-terpyridine

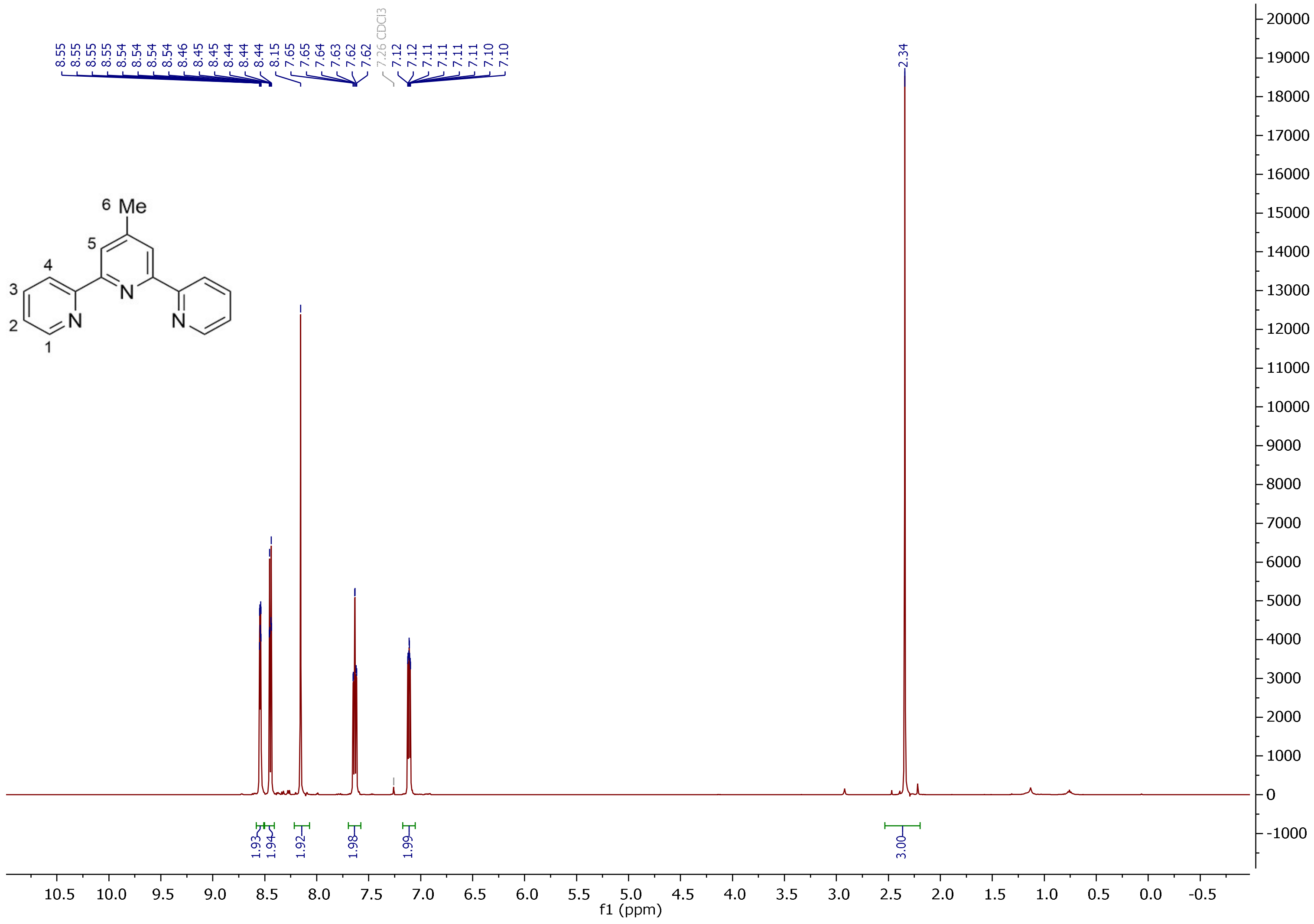

^{13}C NMR (101 MHz, $CDCl_3$) for 4'-methyl-2,2':6',2"-terpyridine

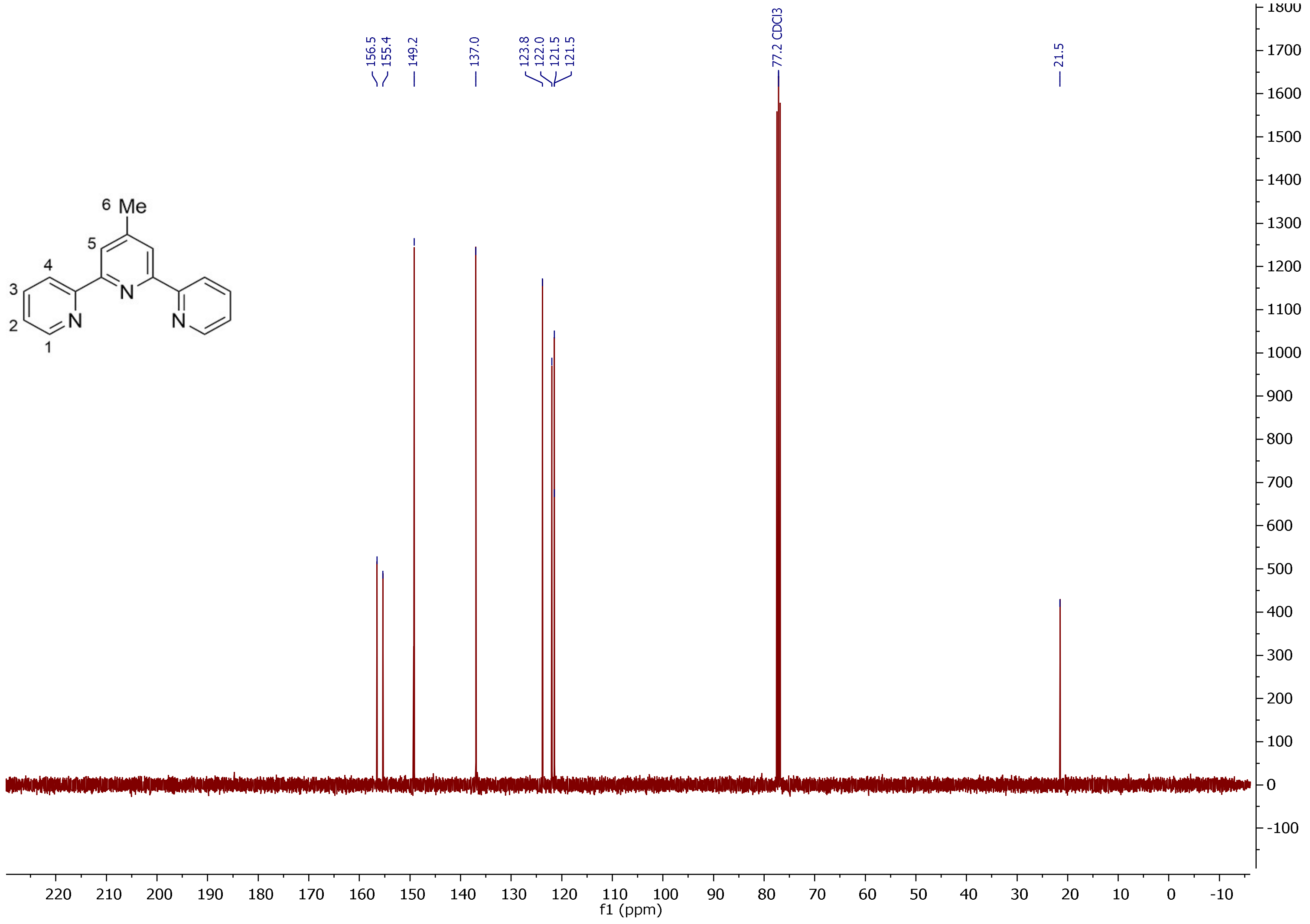

^{1}H NMR (400 MHz, $CDCl_3$) for 4'-(2-methoxyethyl)-2,2':6',2"-terpyridine

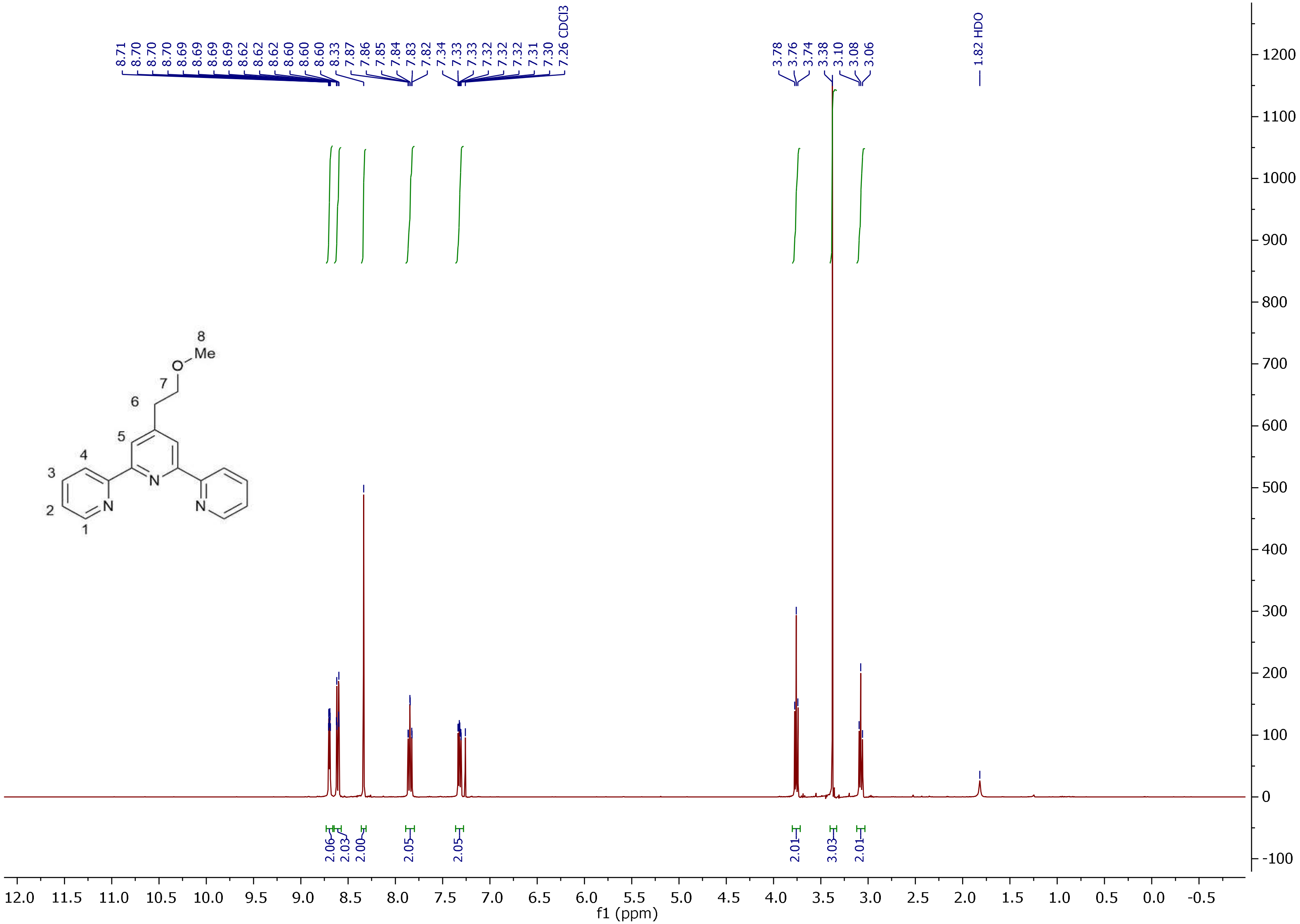

^{13}C NMR (101 MHz, $CDCl_3$) for 4'-(2-methoxyethyl)-2,2':6',2"-terpyridine

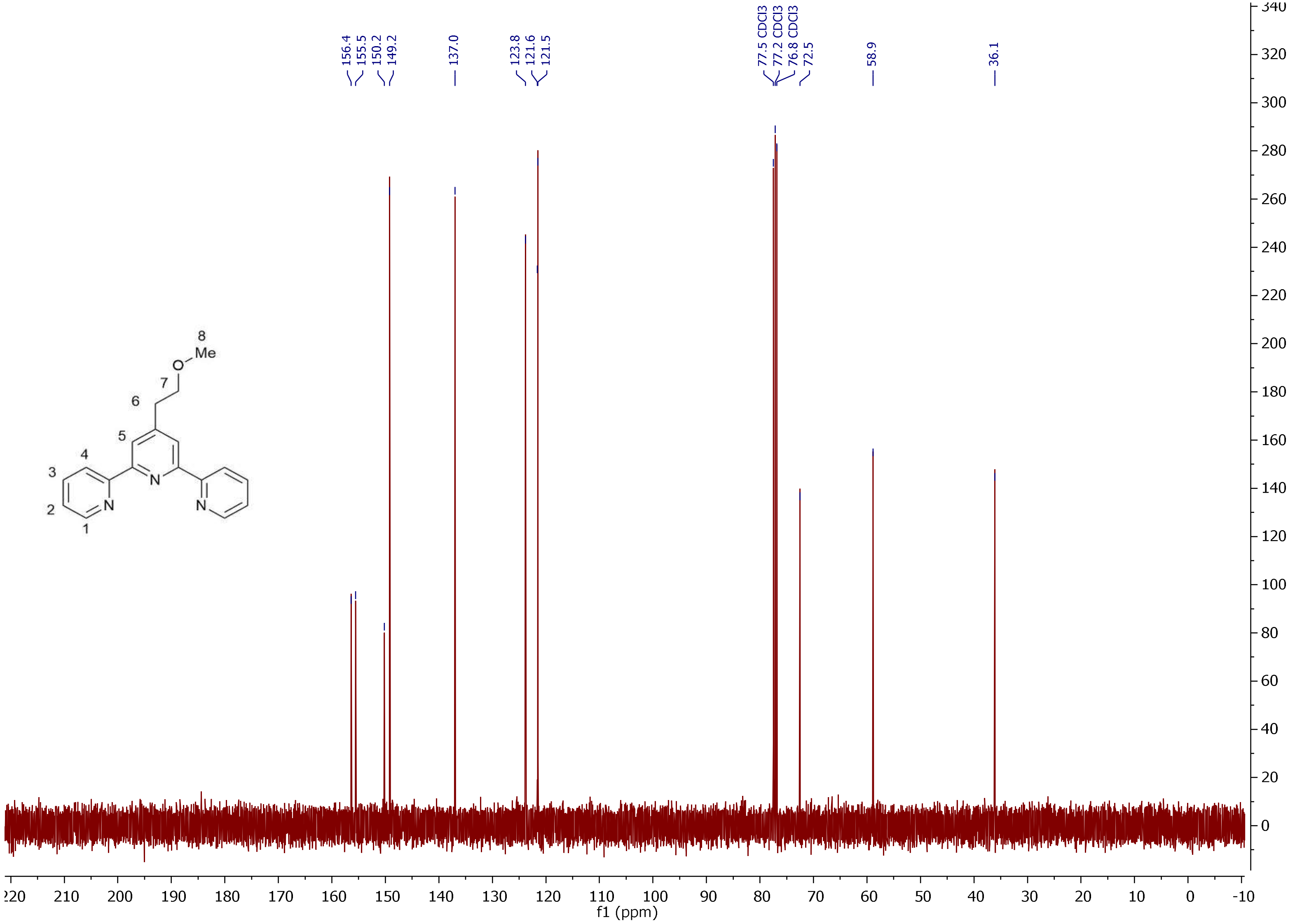

^{1}H NMR (500 MHz, $CDCl_3$) for 4'-(2-bromoethyl)-2,2':6',2"-terpyridine

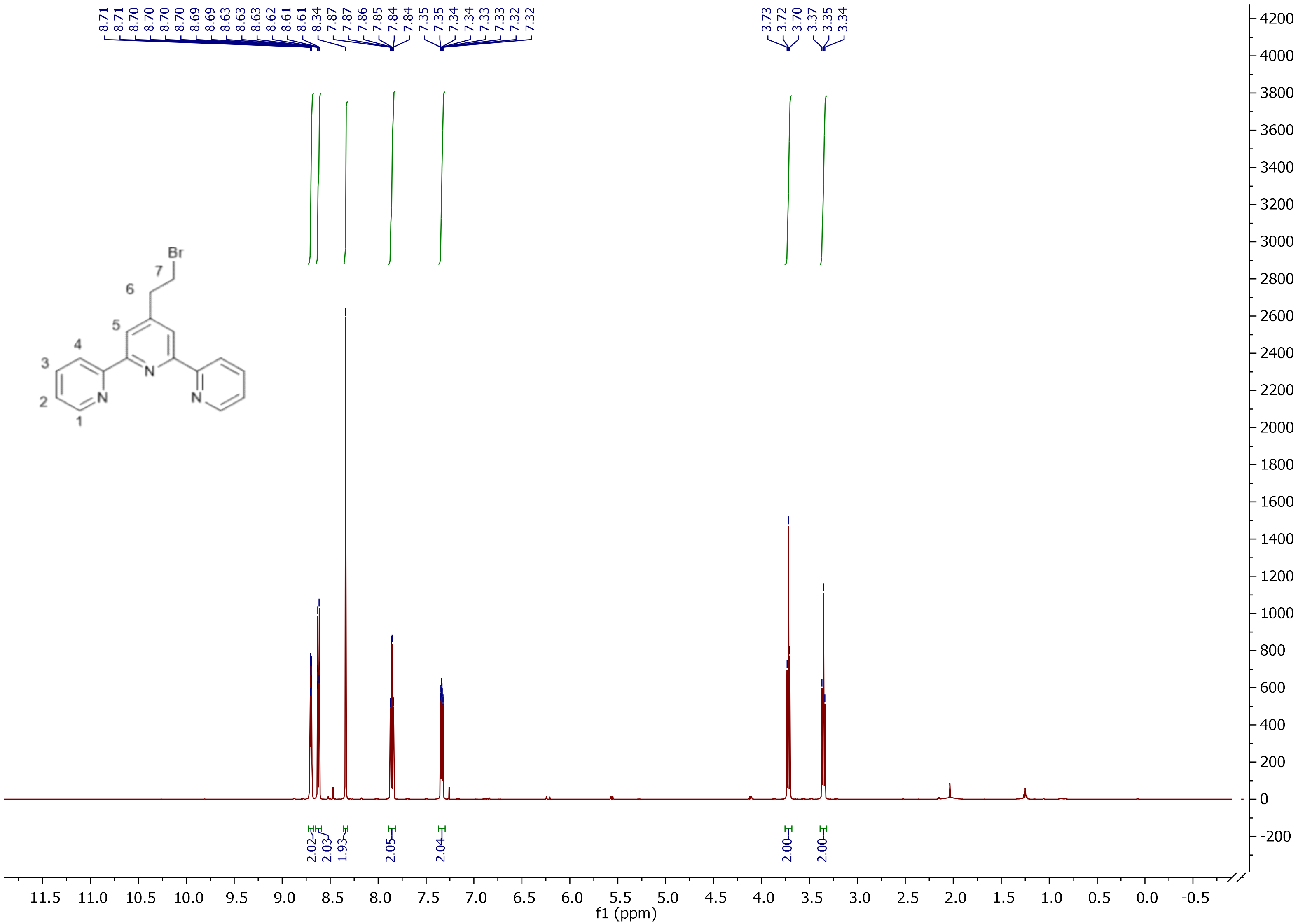

^{13}C NMR (126 MHz, $CDCl_3$) for 4'-(2-bromoethyl)-2,2':6',2"-terpyridine

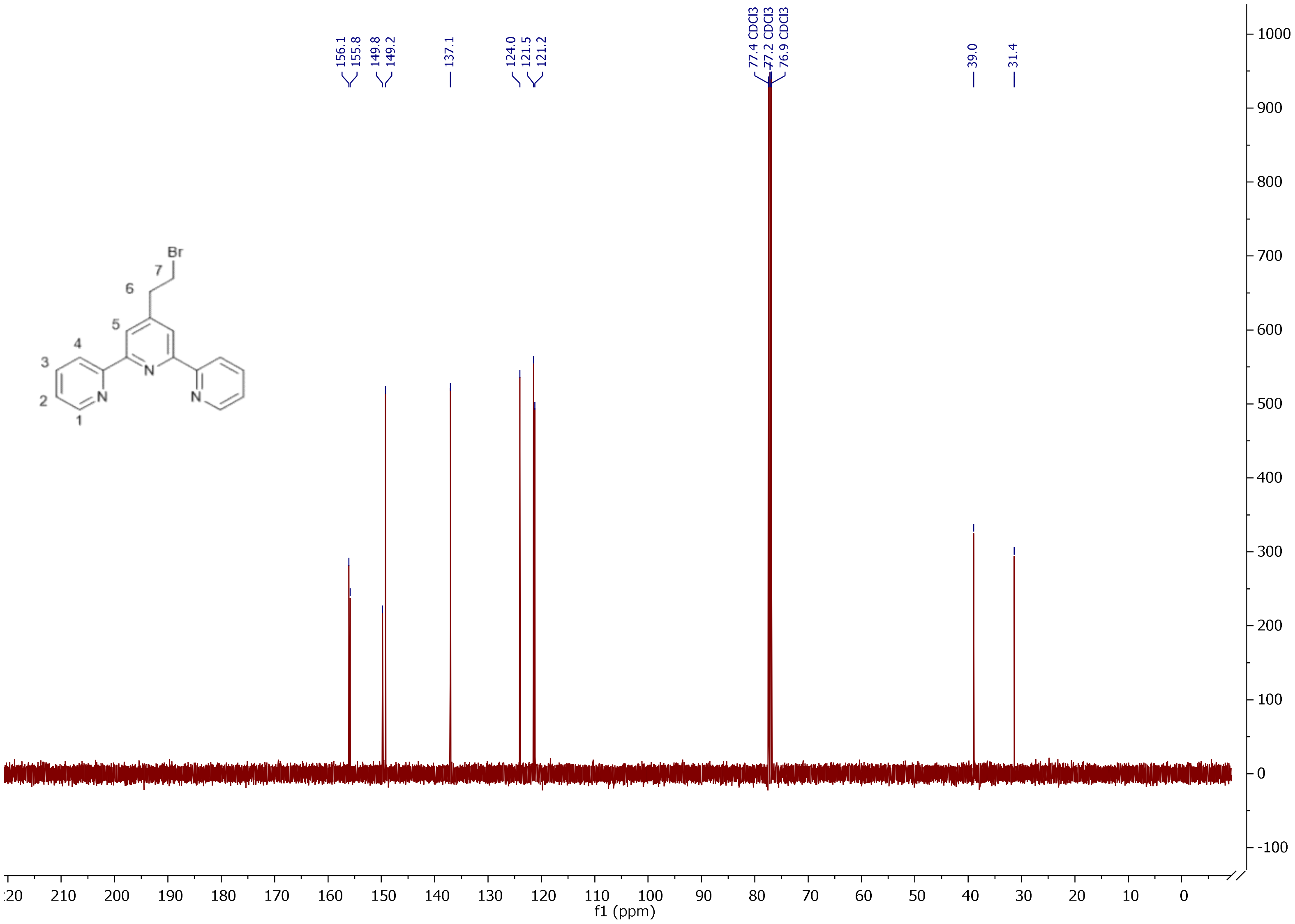

^{1}H NMR (400 MHz, $CDCl_3$) for tetrabutylammonium 2-([2,2':6',2''-terpyridin]-4'-yl)ethane-1-sulfonate **(L5)**

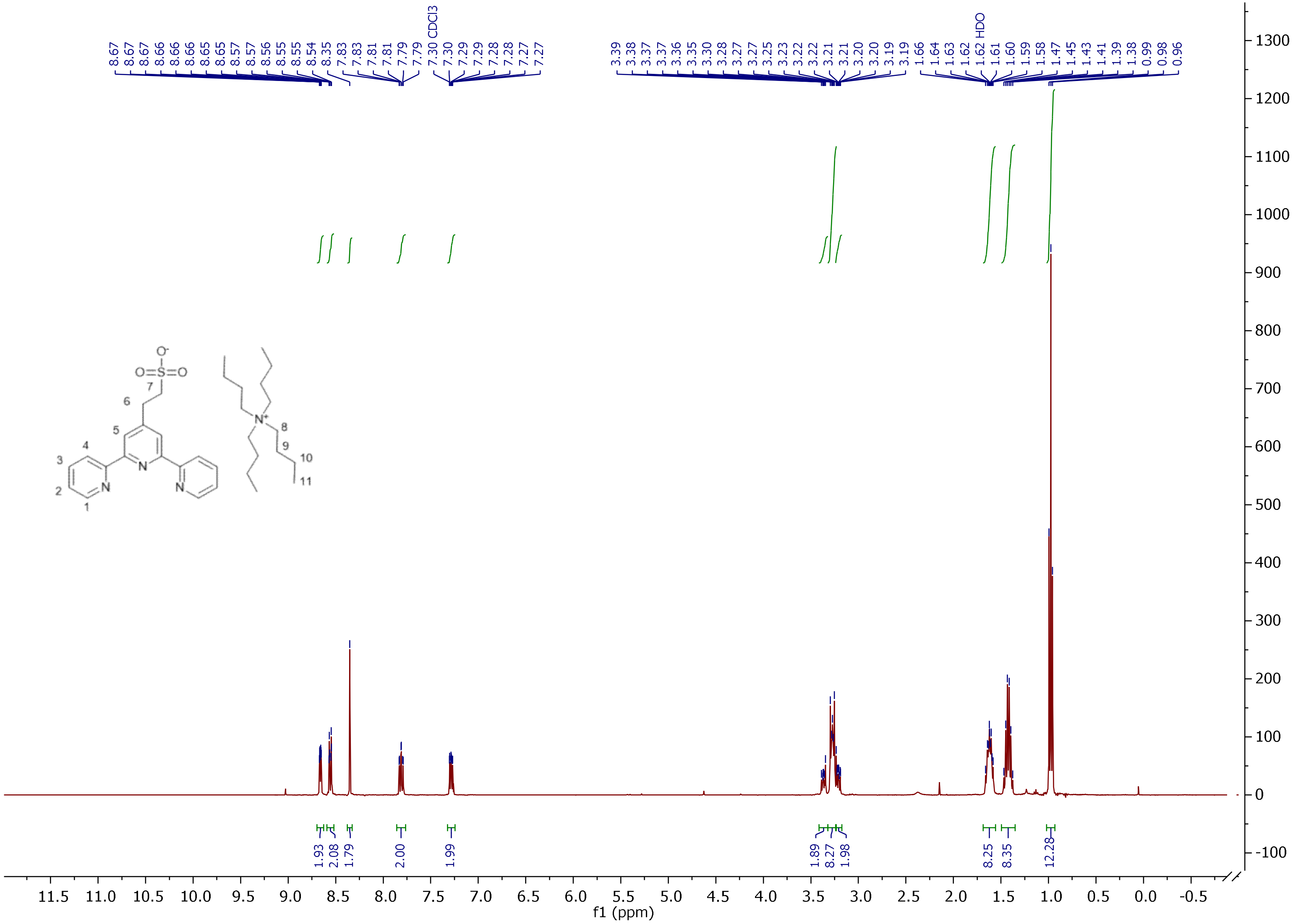

^{13}C NMR (101 MHz, $CDCl_3$) for tetrabutylammonium 2-([2,2':6',2"-terpyridin]-4'-yl)ethane-1-sulfonate **(L5)**

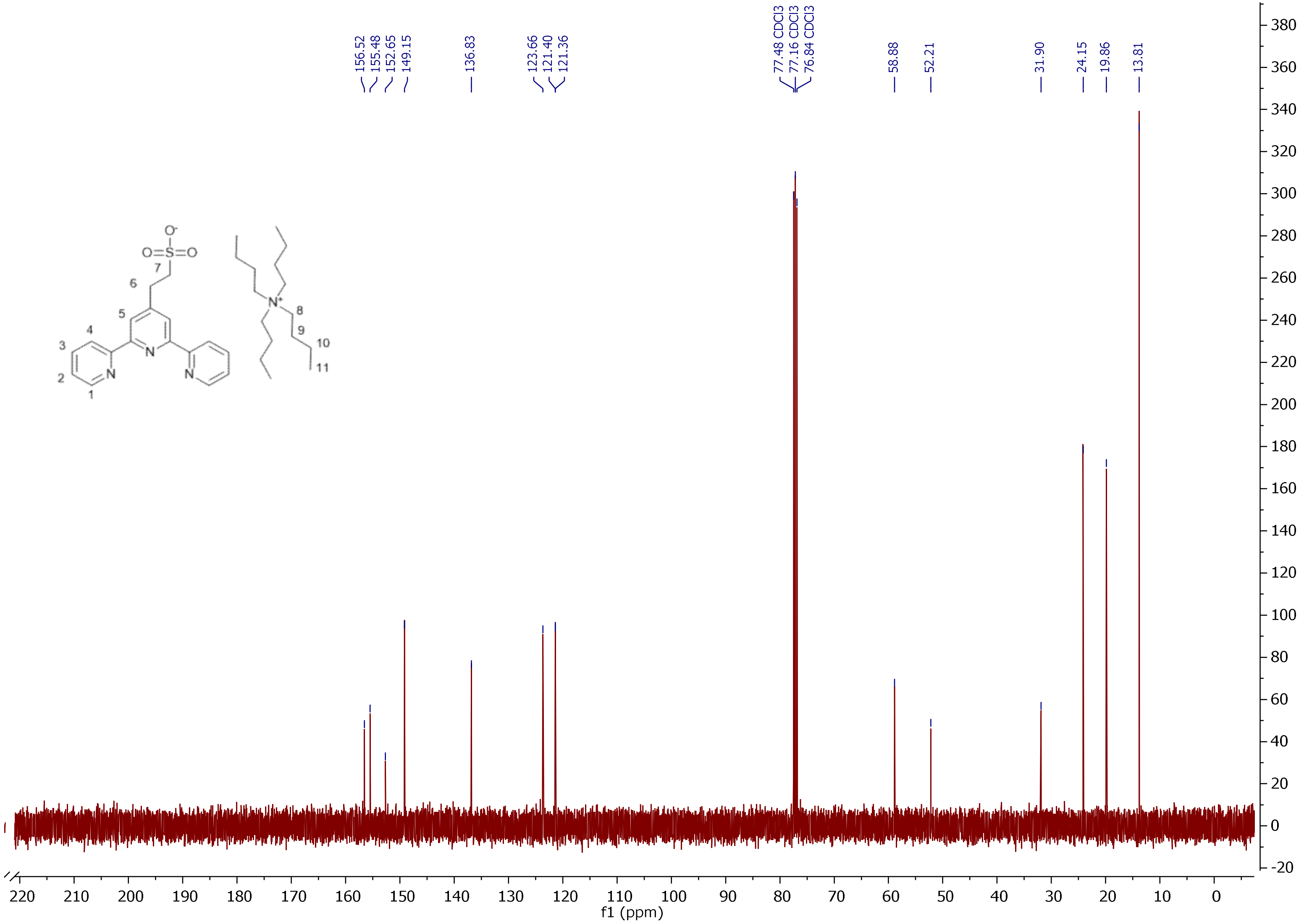

^{1}H NMR (400 MHz, DMSO-d^6) for sodium 2-([2,2':6',2''-terpyridin]-4'-yl)benzenesulfonate

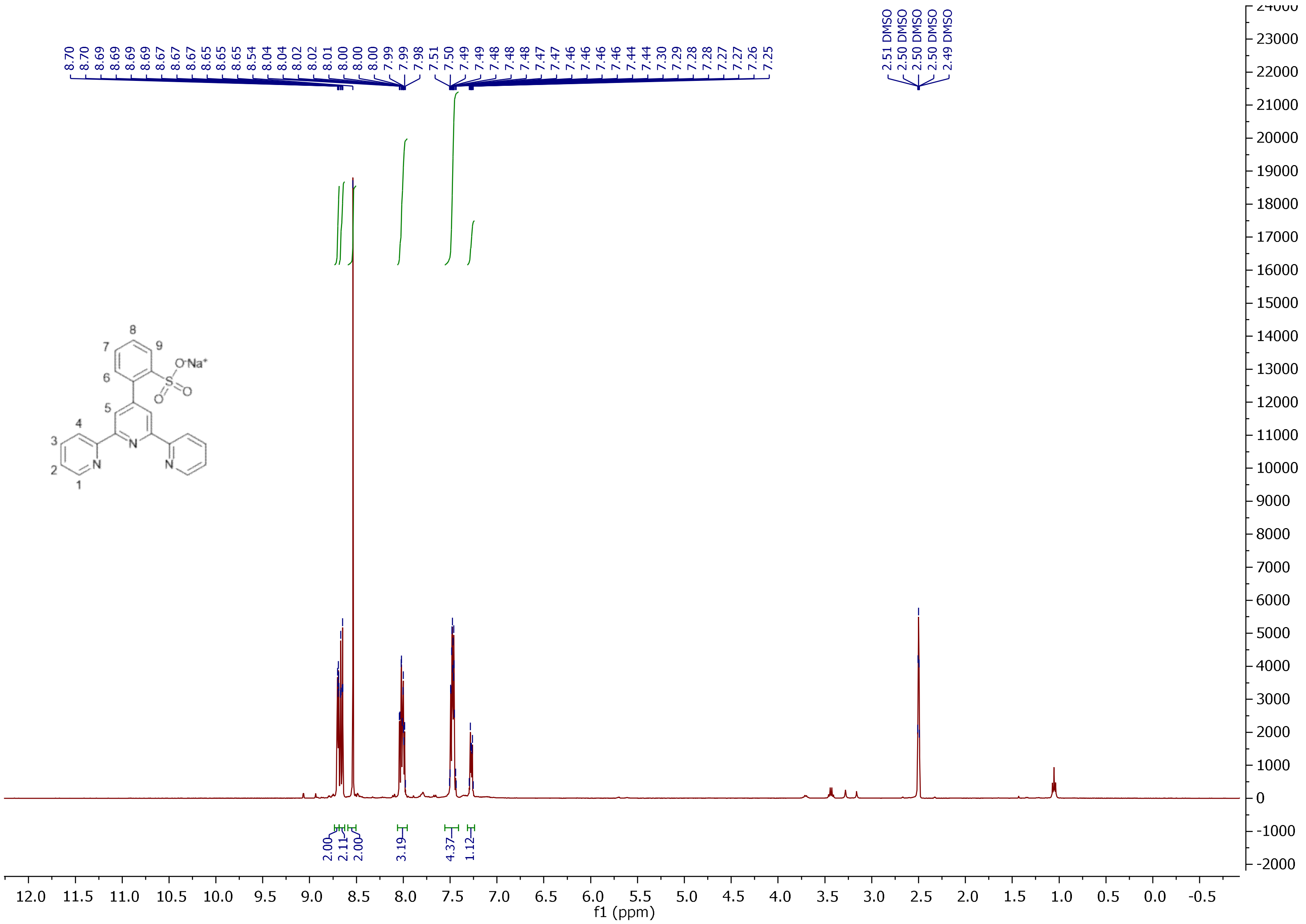

^{13}C NMR (101 MHz, DMSO-d^6) for sodium 2-([2,2':6',2"-terpyridin]-4'-yl)benzenesulfonate

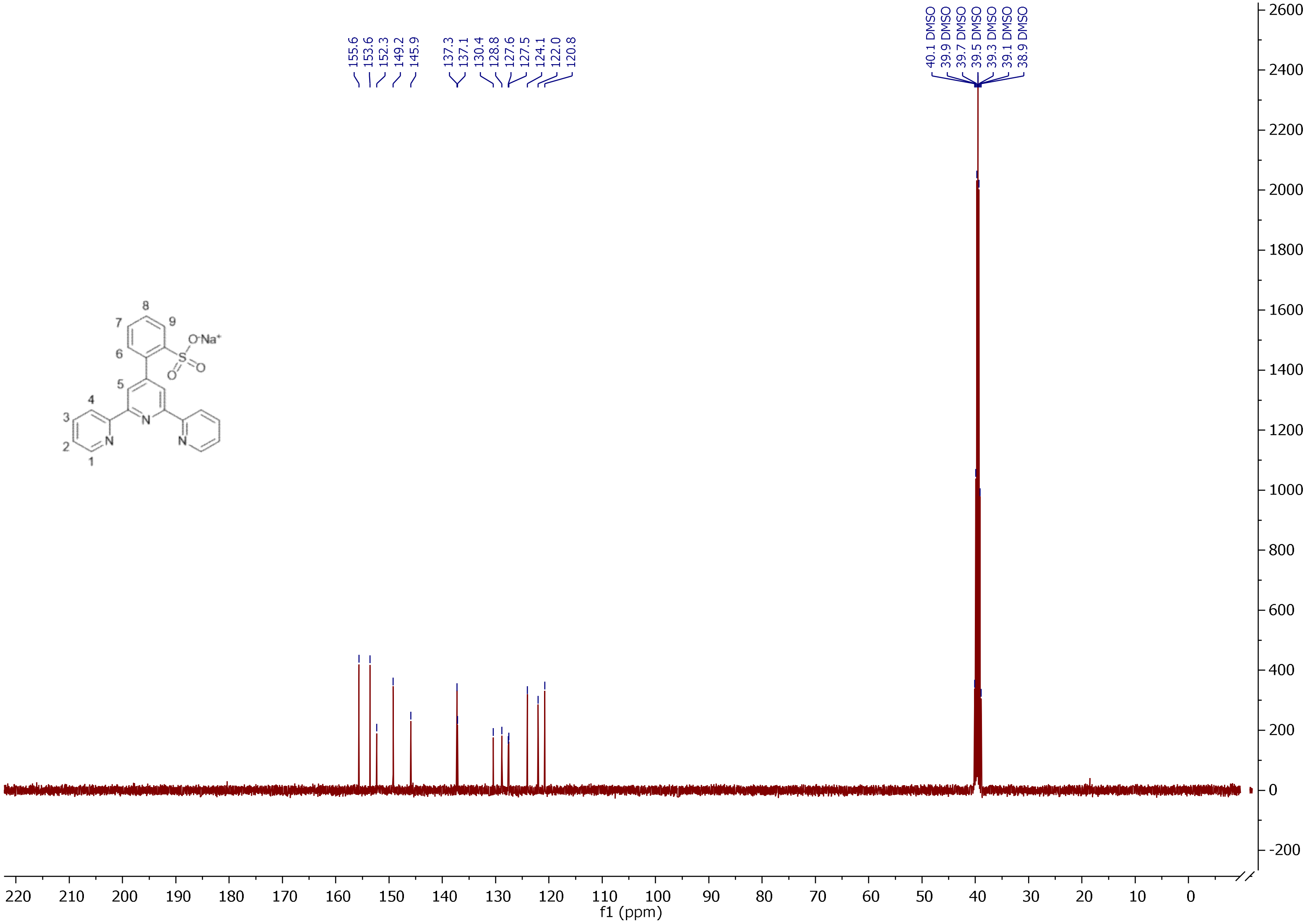

^{1}H NMR (400 MHz, $CDCl_3$) for tetrabutylammonium 2-([2,2':6',2"-terpyridin]-4'-yl)benzenesulfonate **(L6)**

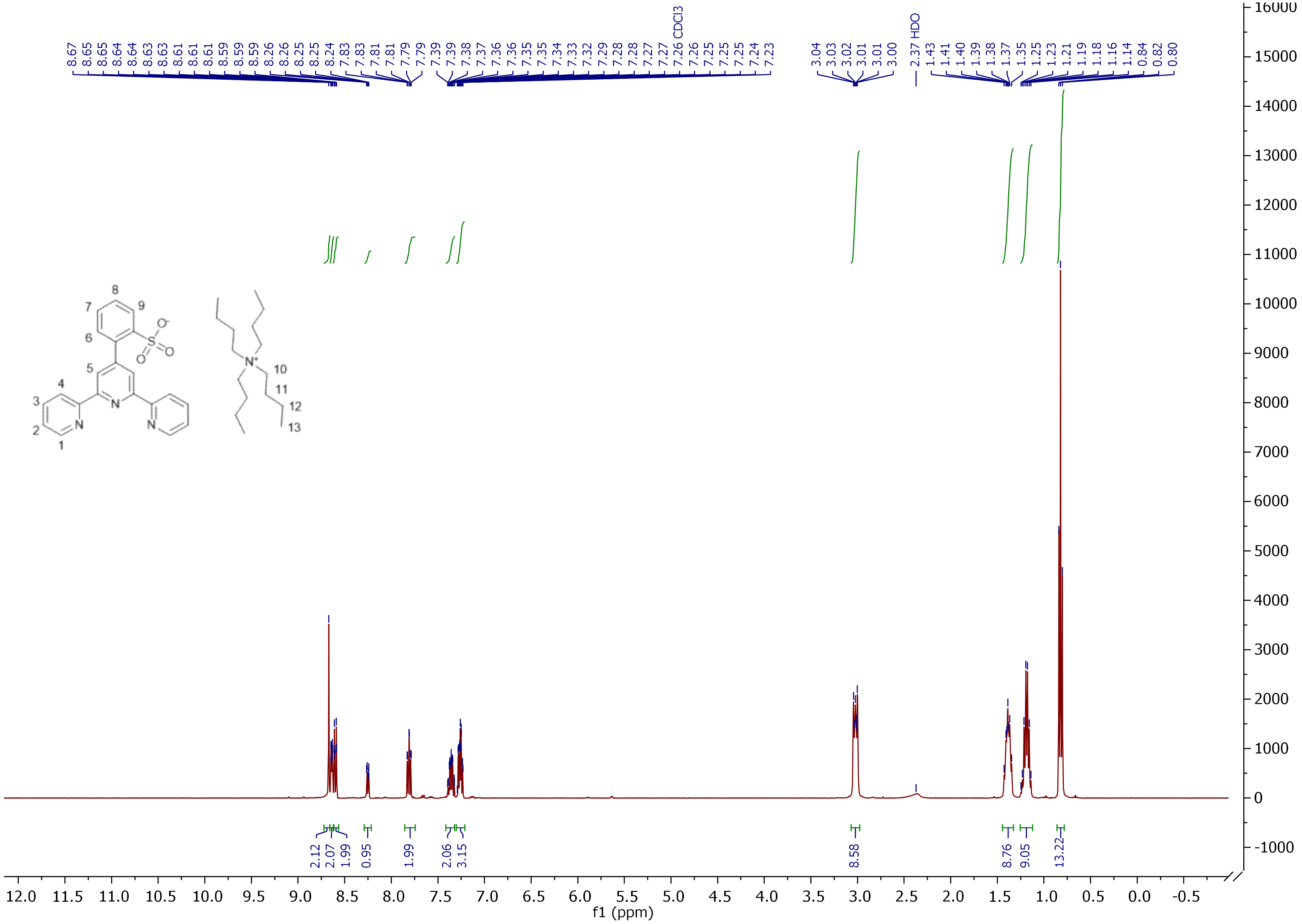

^{13}C NMR (101 MHz, $CDCl_3$) for tetrabutylammonium 2-([2,2':6',2"-terpyridin]-4'-yl)benzenesulfonate **(L6)**

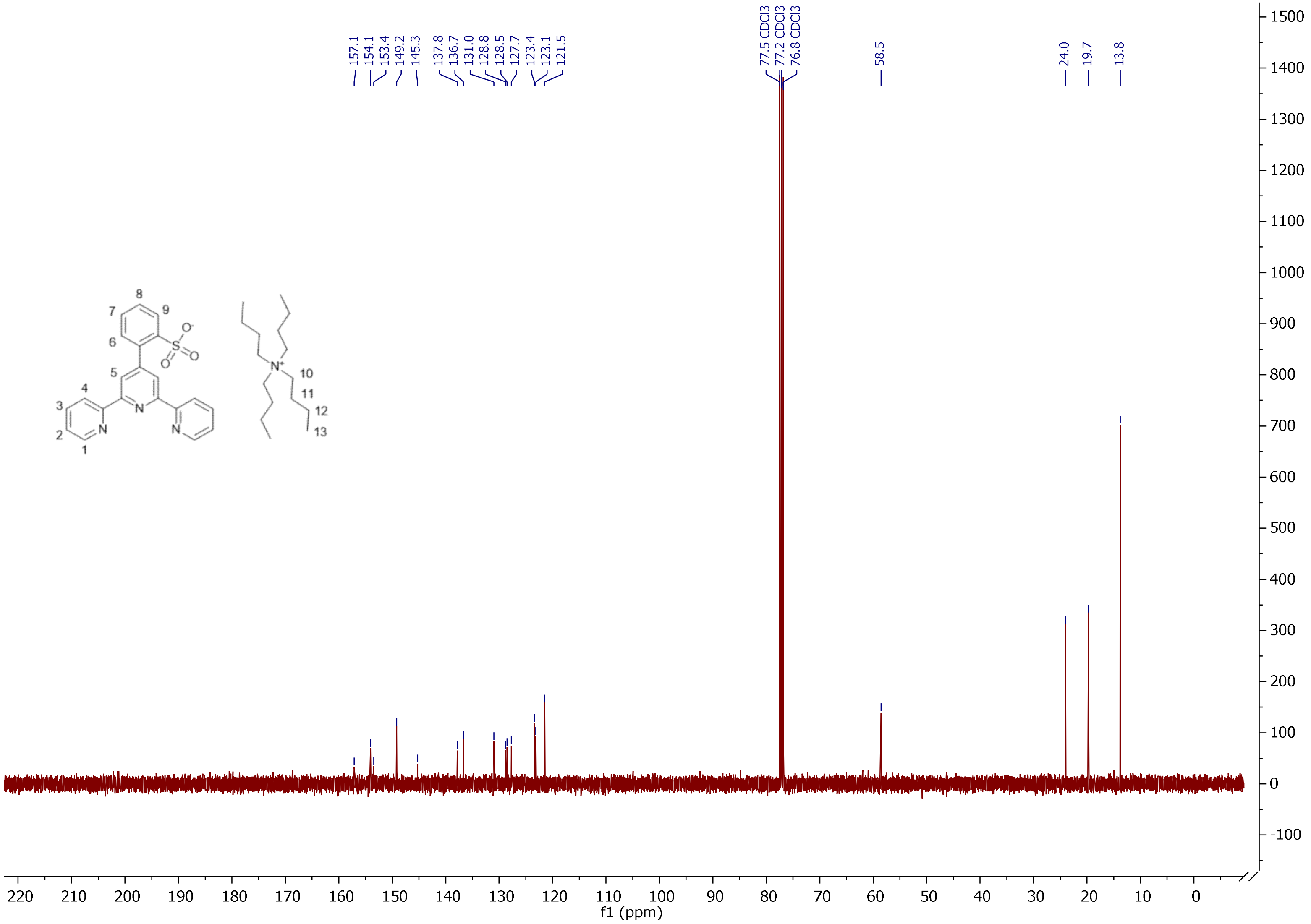

^{1}H NMR (400 MHz, $CDCl_3$) for 4'-(*m*-tolyl)-2,2':6',2"-terpyridine

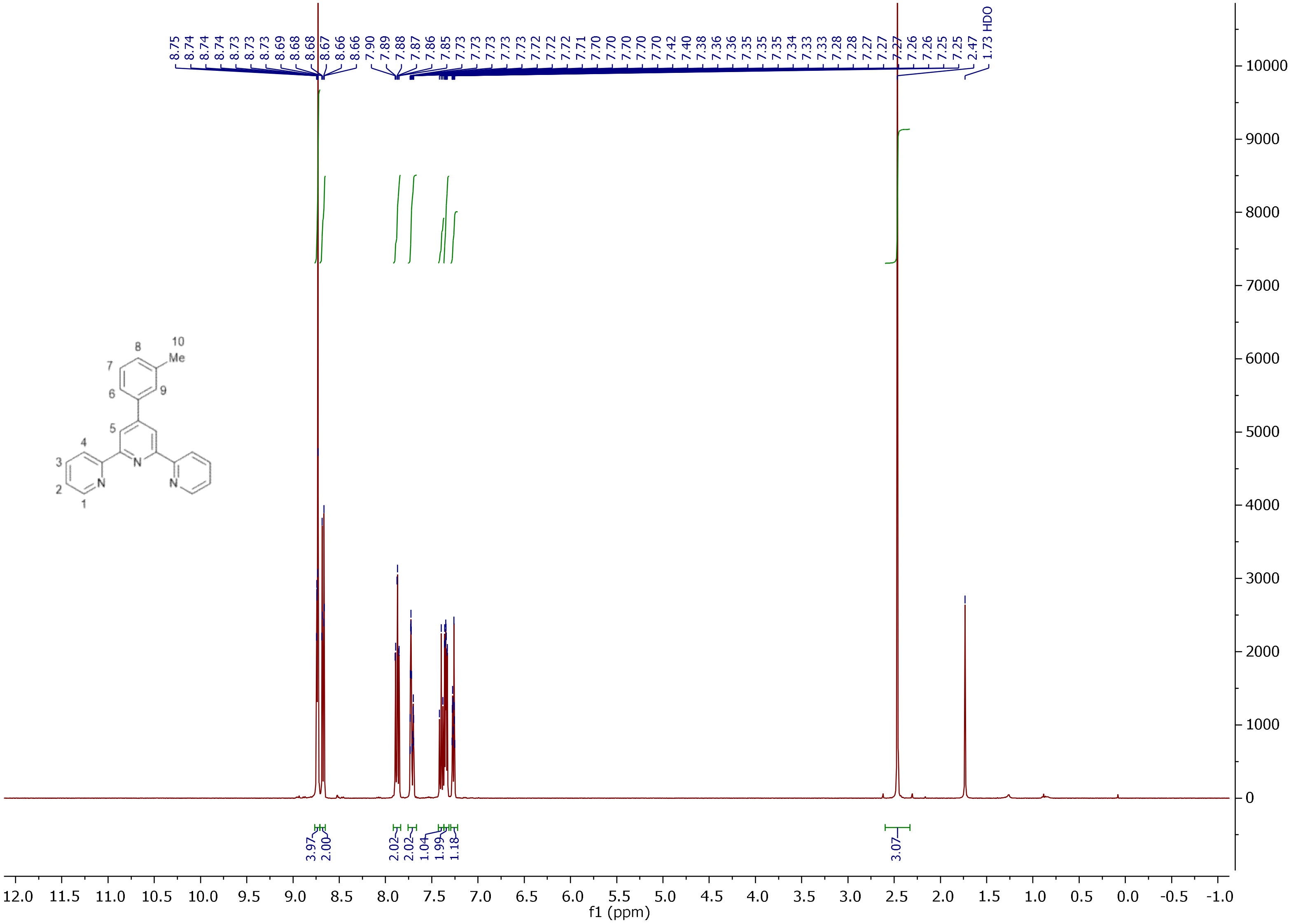

^{13}C NMR (101 MHz, $CDCl_3$) for 4'-(*m*-tolyl)-2,2':6',2"-terpyridine

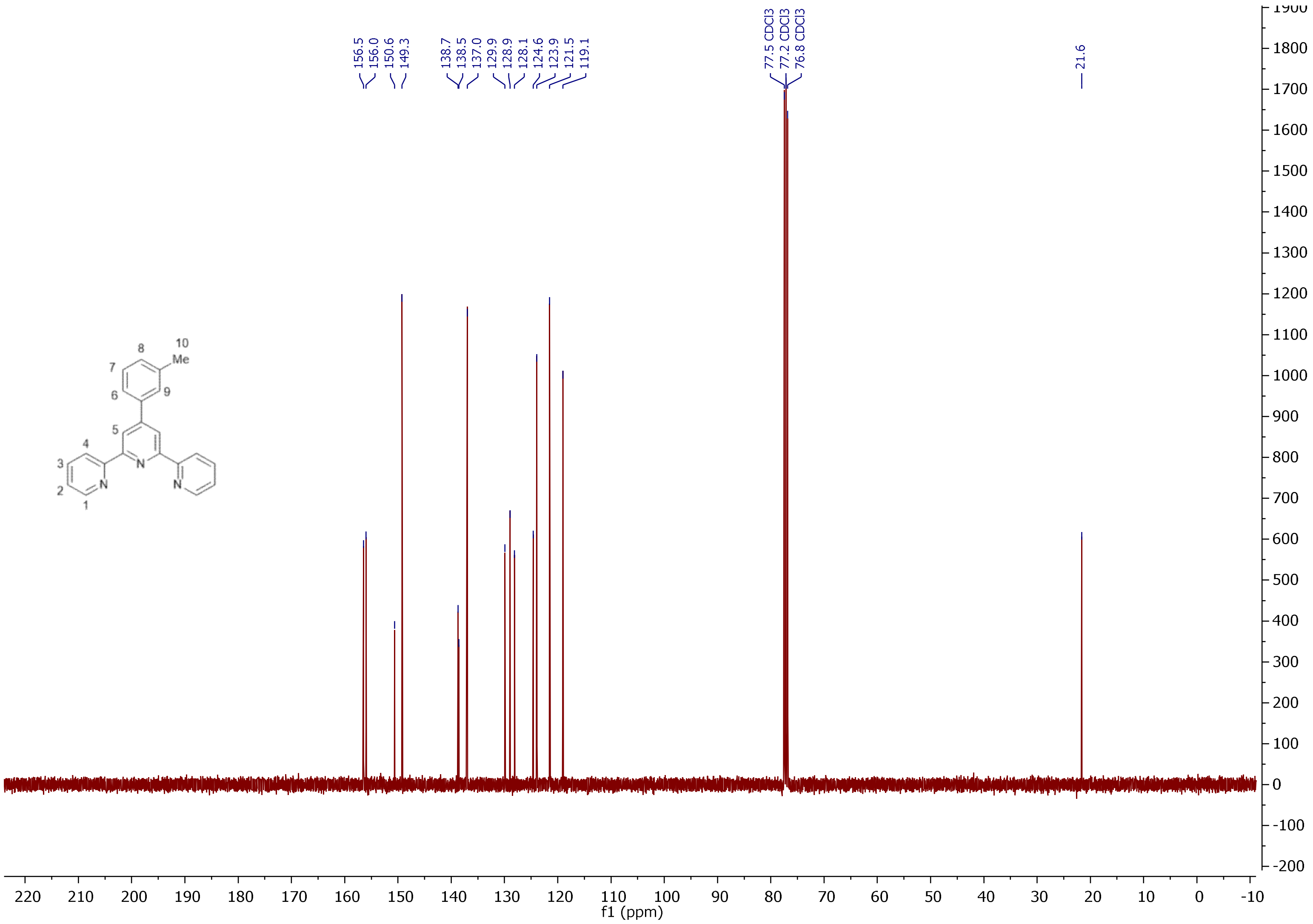

^{1}**H NMR** (500 MHz, $CDCl_3$) for tetrabutylammonium (3-([2,2':6',2"-terpyridin]-4'-yl)phenyl)methanesulfonate **(L7)**

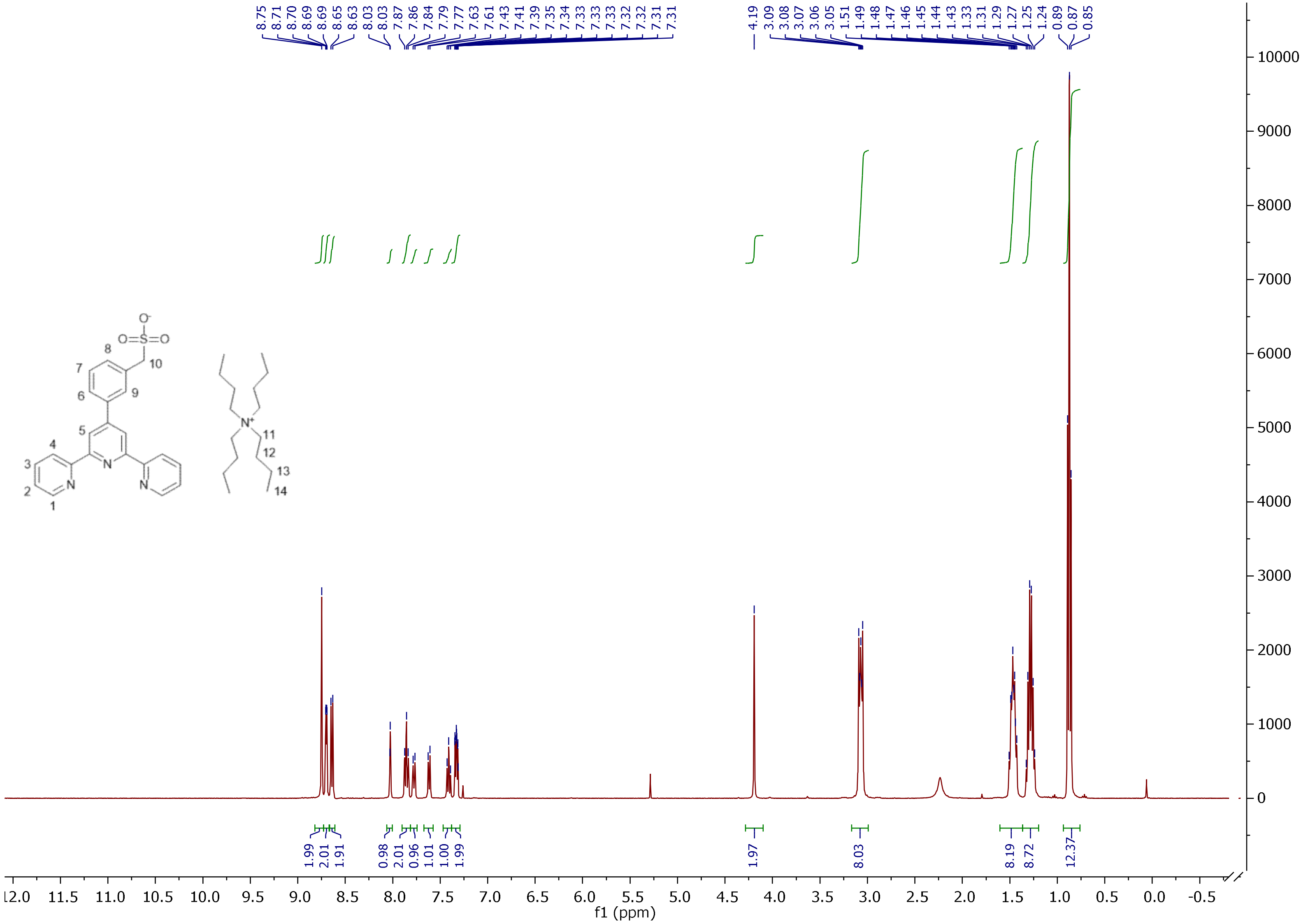

^{13}C NMR (126 MHz, $CDCl_3$) for tetrabutylammonium (3-([2,2':6',2''-terpyridin]-4'-yl)phenyl)methanesulfonate **(L7)**

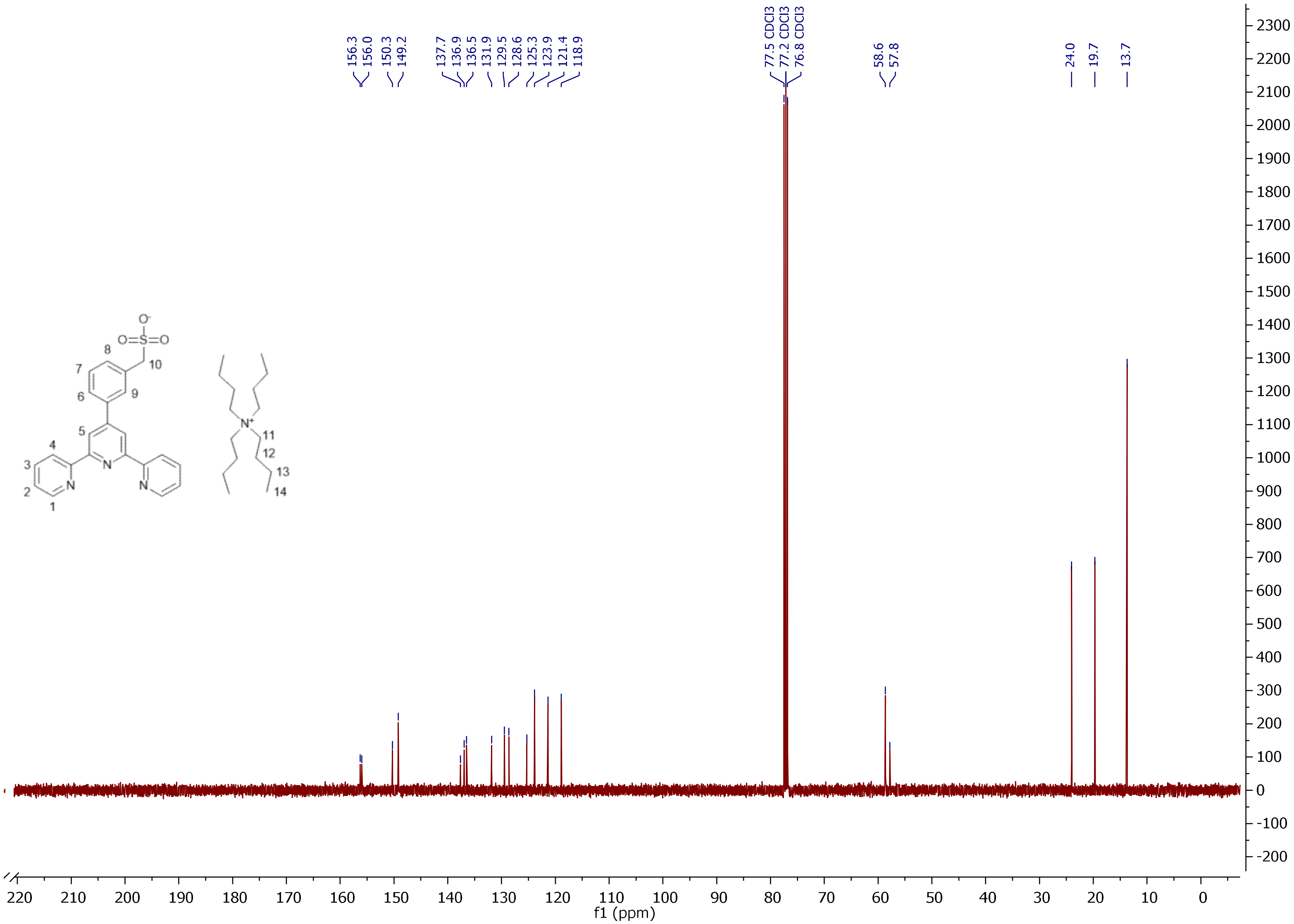

^{1}H NMR (400 MHz, $CDCl_3$) for 4'-(*p*-tolyl)-2,2':6',2''-terpyridine

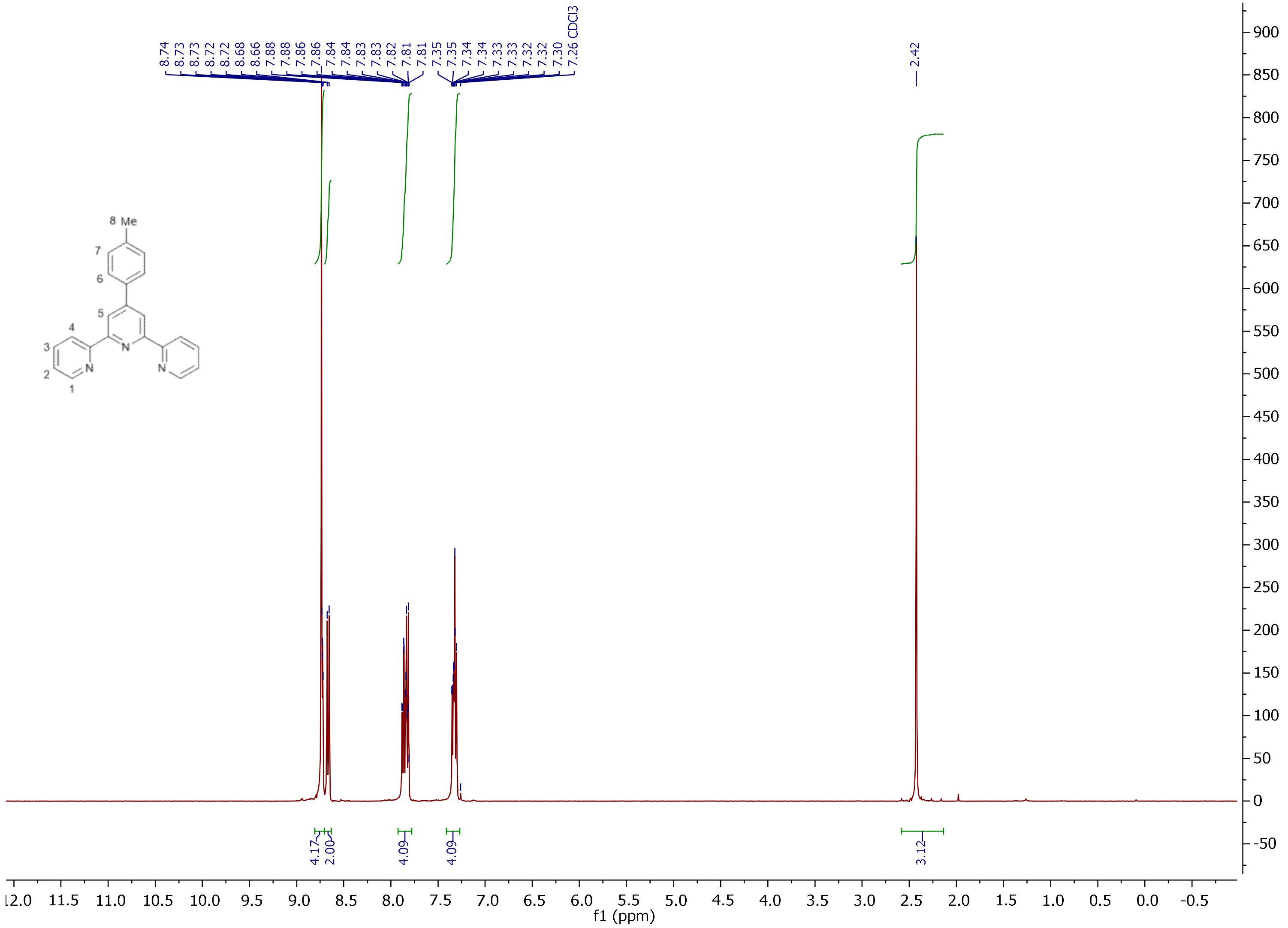

^{13}C NMR (101 MHz, $CDCl_3$) for 4'-(*p*-tolyl)-2,2':6',2"-terpyridine

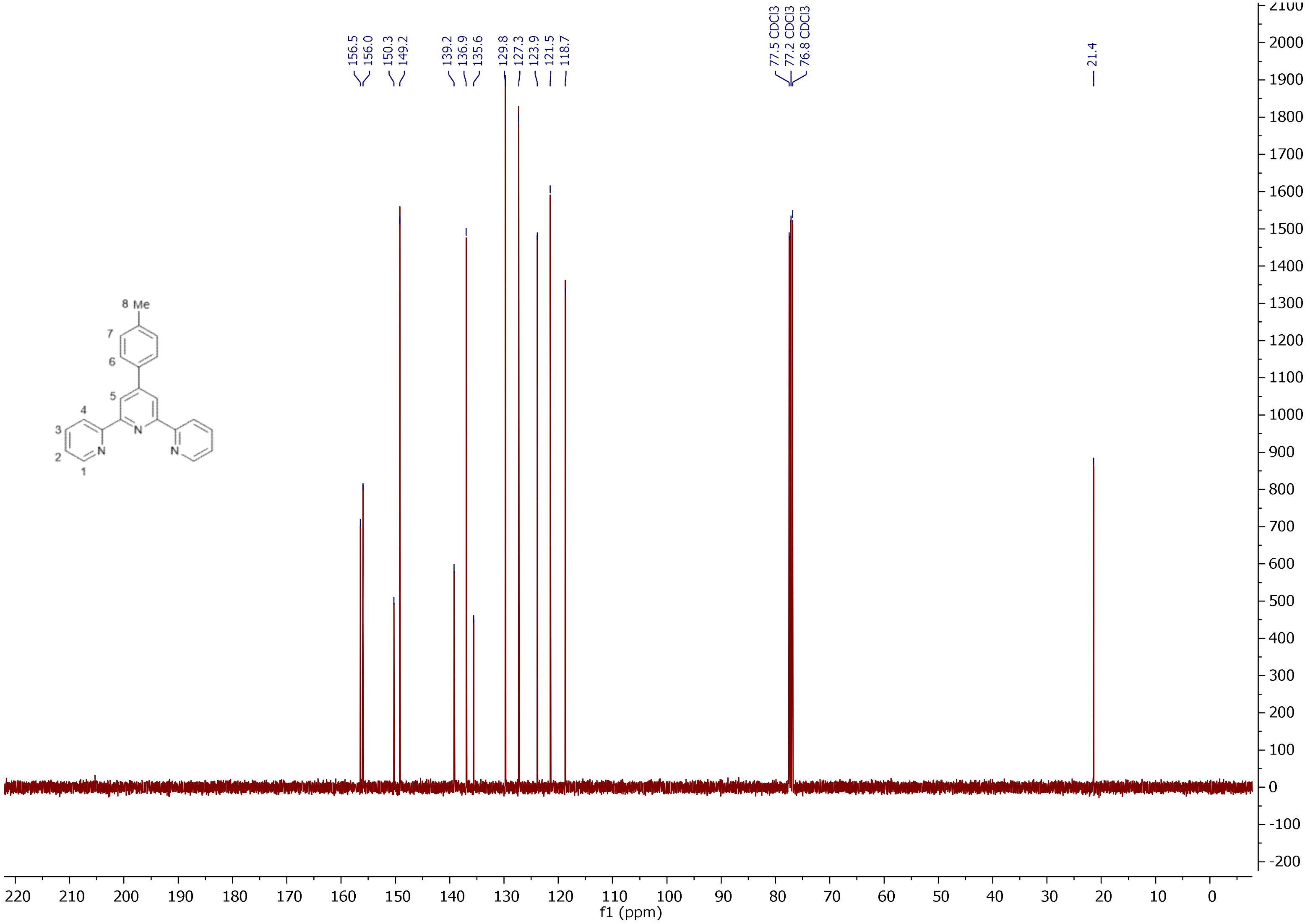

^{1}H NMR (400 MHz, $CDCl_3$) for tetrabutylammonium (3-([2,2':6',2''-terpyridin]-4'-yl)phenyl)methanesulfonate **(L8)**

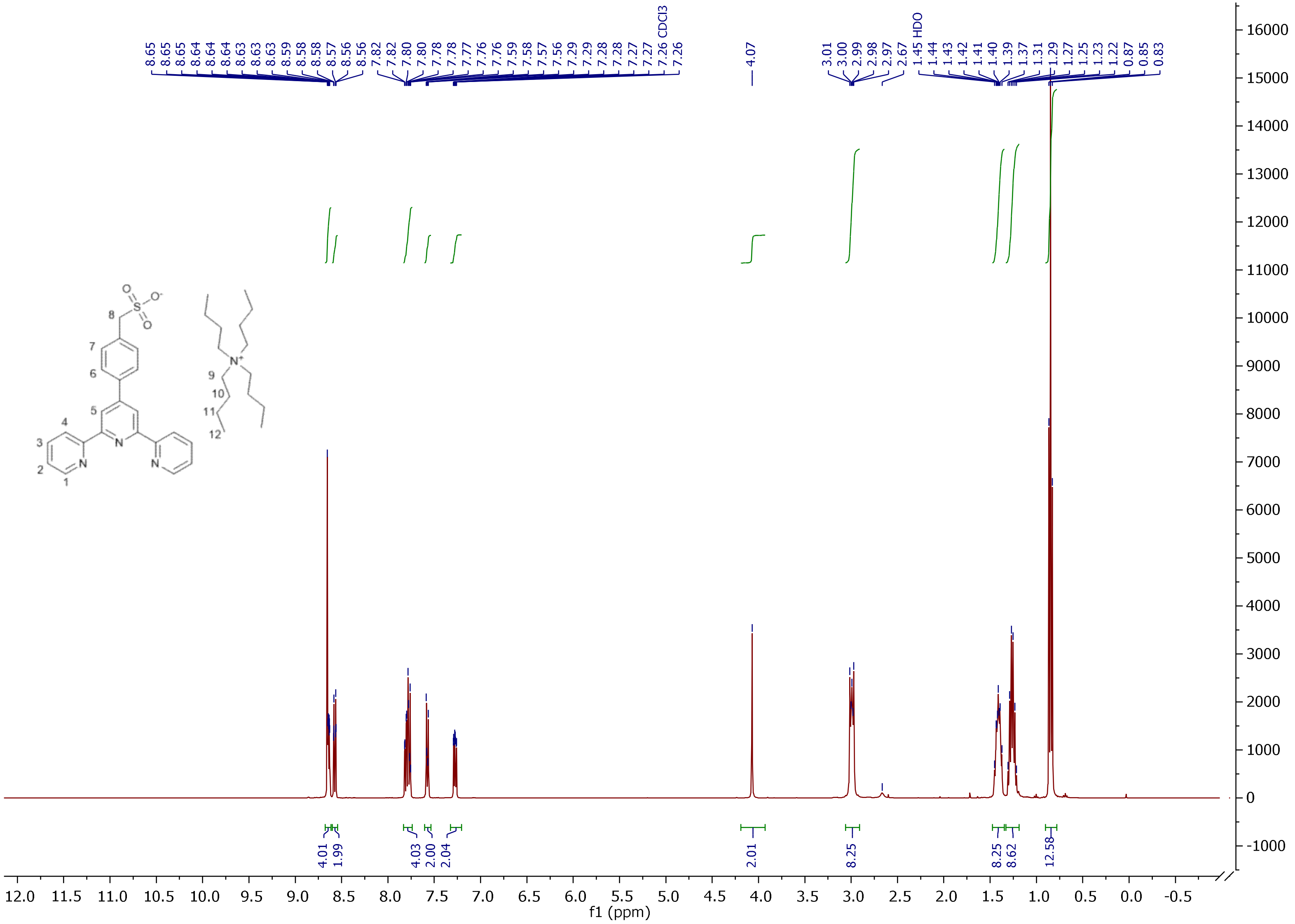

^{13}C NMR (101 MHz, $CDCl_3$) for tetrabutylammonium (3-([2,2':6',2''-terpyridin]-4'-yl)phenyl)methanesulfonate **(L8)**

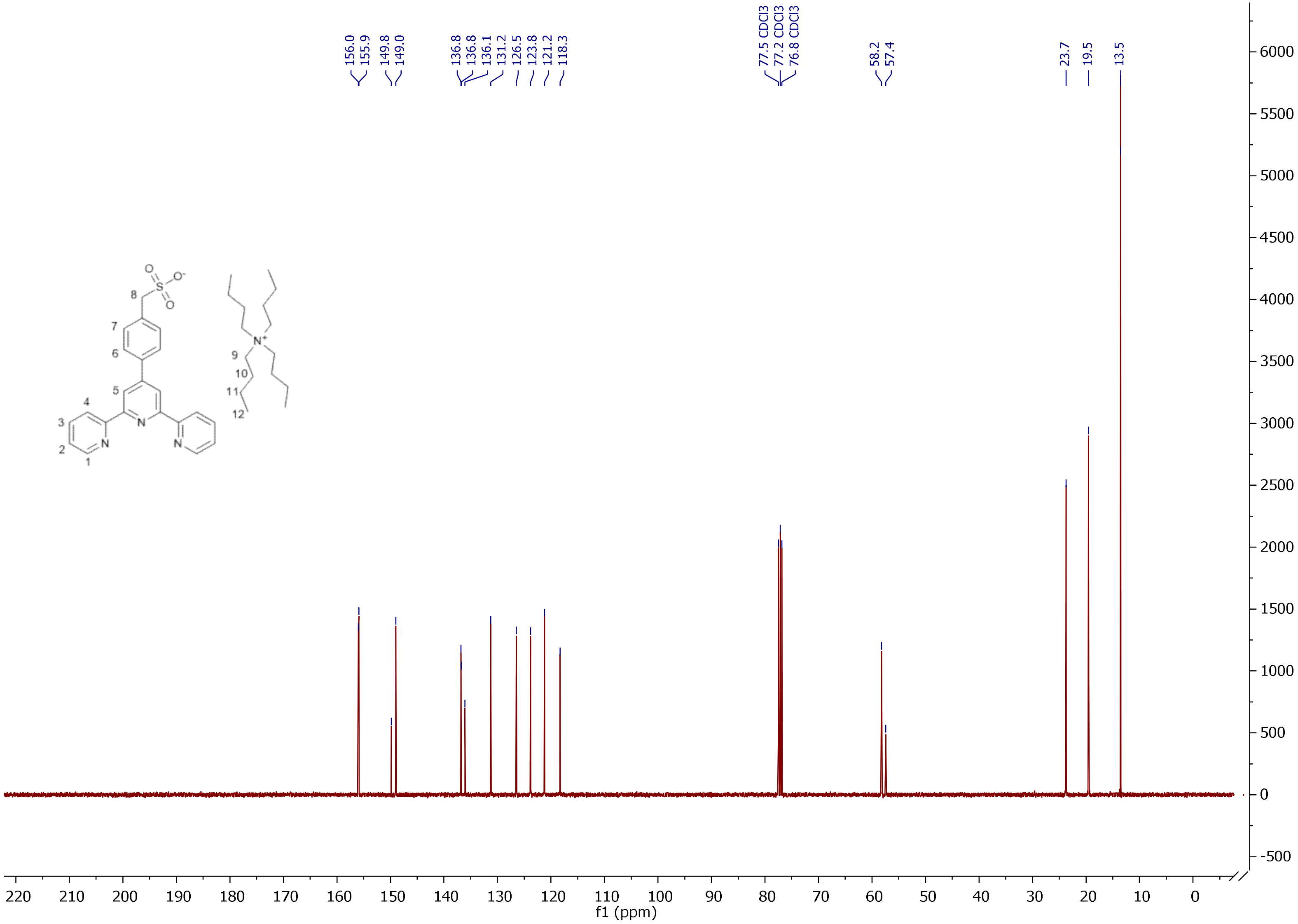

¹H NMR (500 MHz, $CDCl_3$) for 4'-(*o*-tolyl)-2,2':6',2"-terpyridine

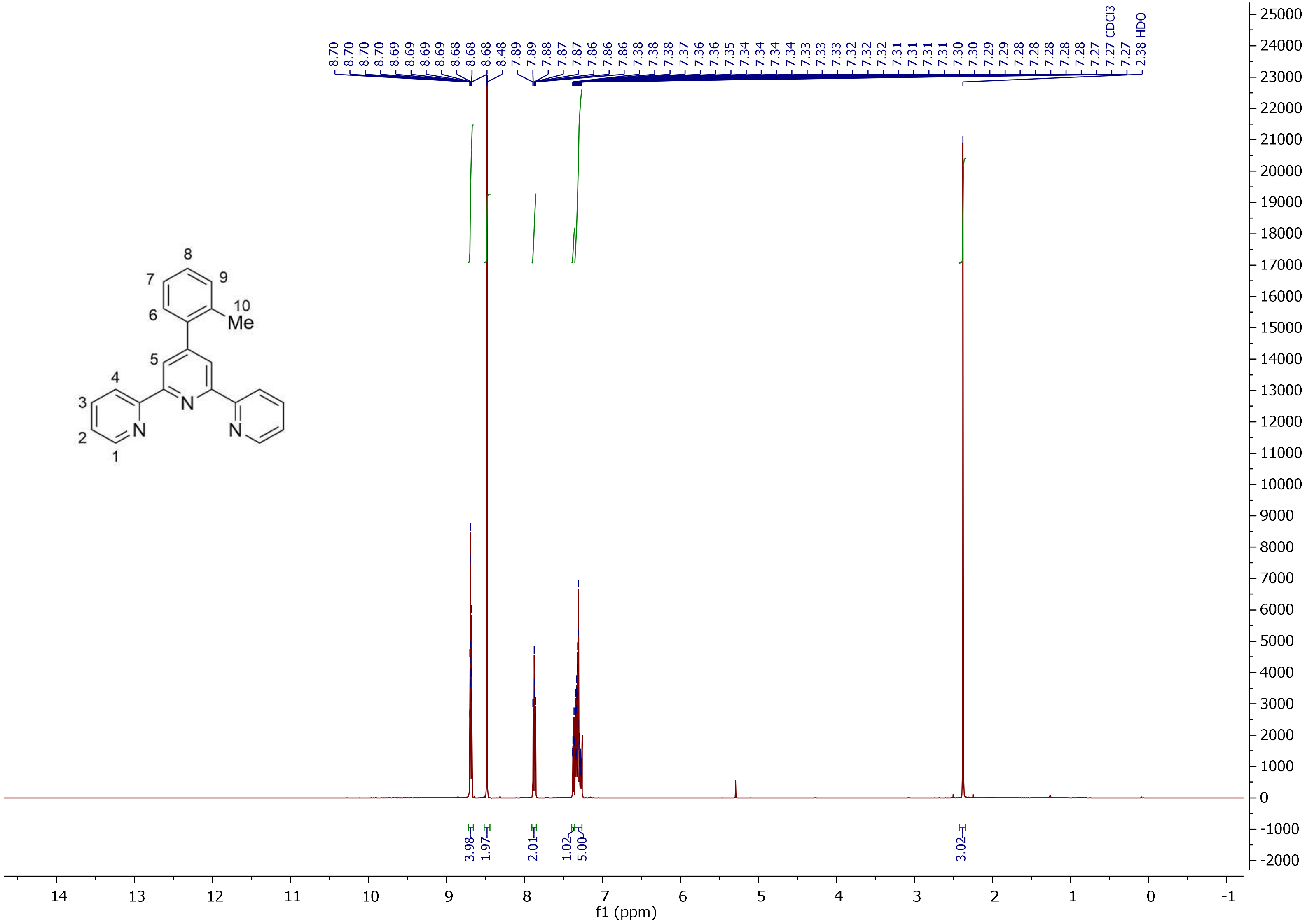

^{13}**C NMR** (126 MHz, $CDCl_3$) for 4'-(*o*-tolyl)-2,2':6',2''-terpyridine

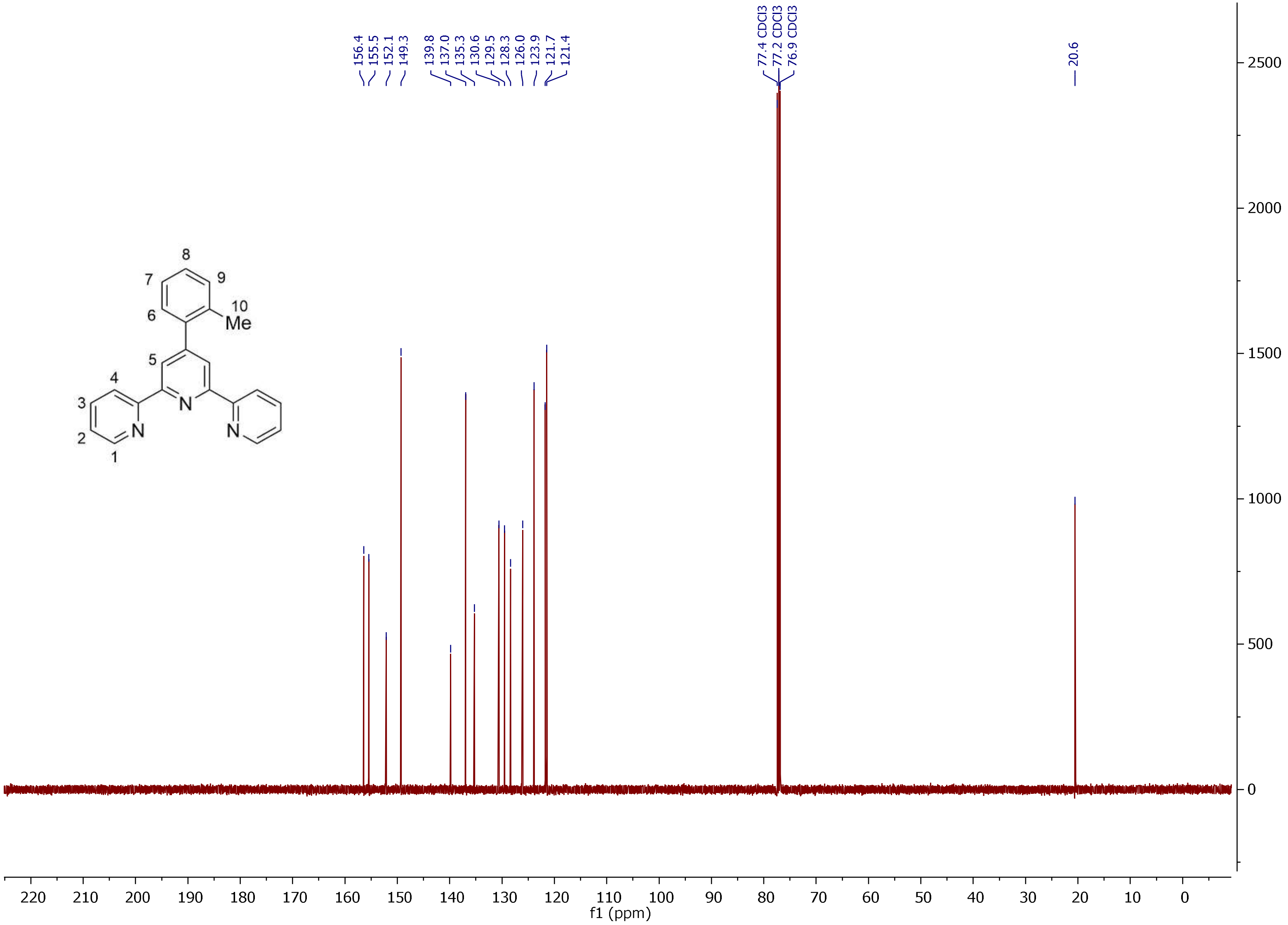

[1]**H NMR** (400 MHz, $CDCl_3$) for tetrabutylammonium (3-([2,2':6',2"-terpyridin]-4'-yl)phenyl)methanesulfonate **(L9)**

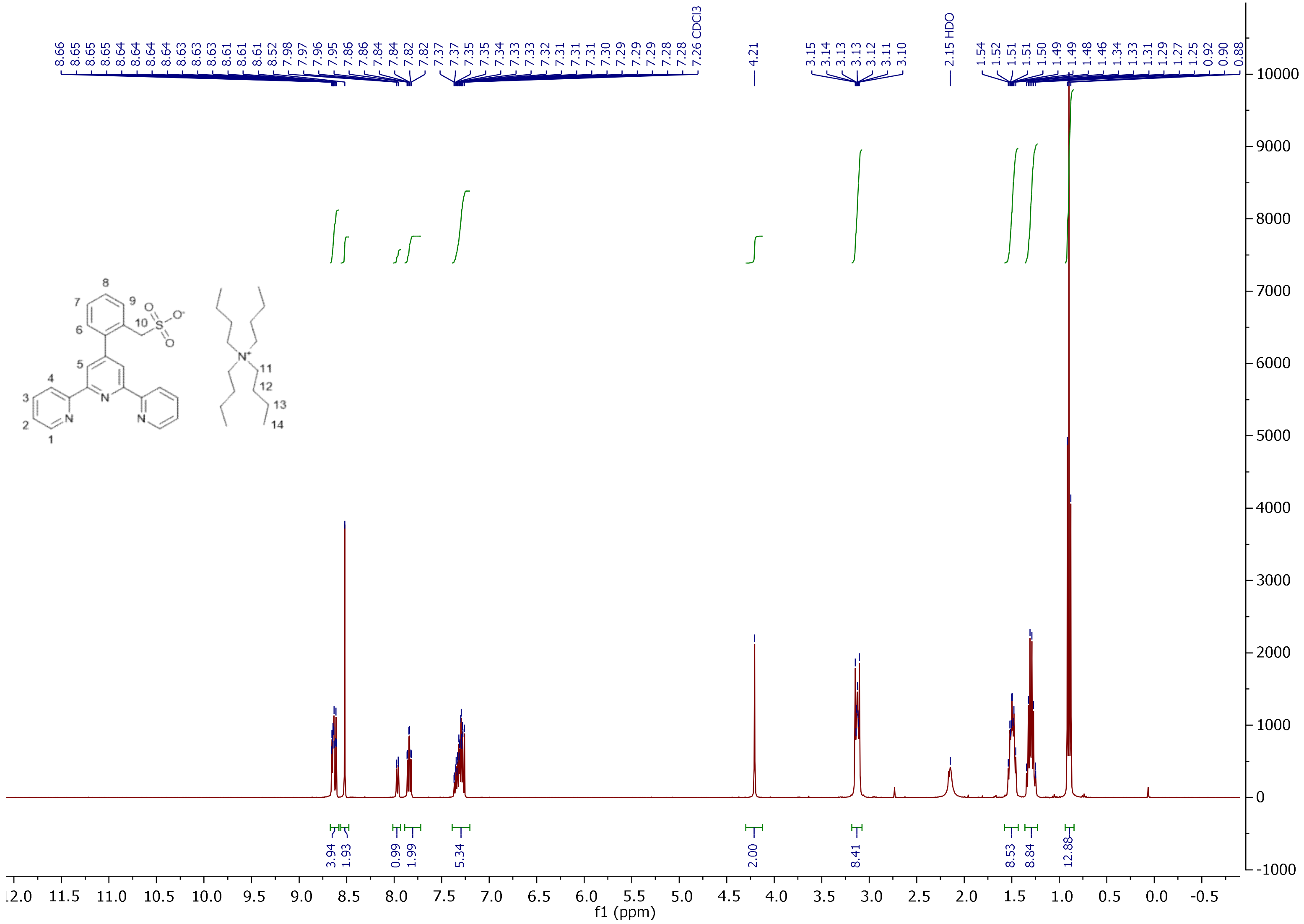

^{13}C NMR (101 MHz, $CDCl_3$) for tetrabutylammonium (3-([2,2':6',2''-terpyridin]-4'-yl)phenyl)methanesulfonate **(L9)**

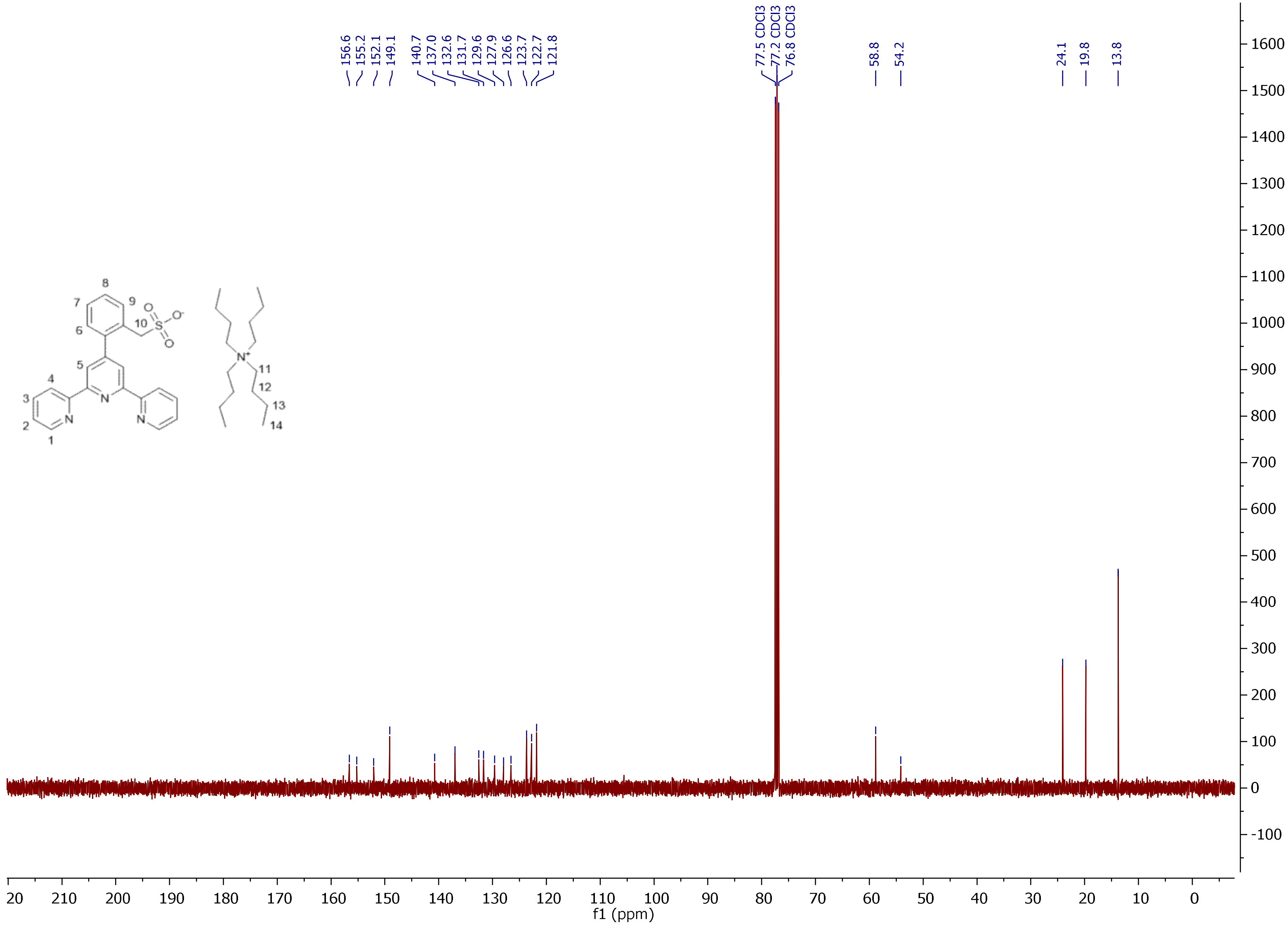

^{1}H NMR (400 MHz, $CDCl_3$) for 4,4''-di-*tert*-butyl-4'-(((tetrahydro-2H-pyran-2-yl)oxy)methyl)-2,2':6',2''-terpyridine

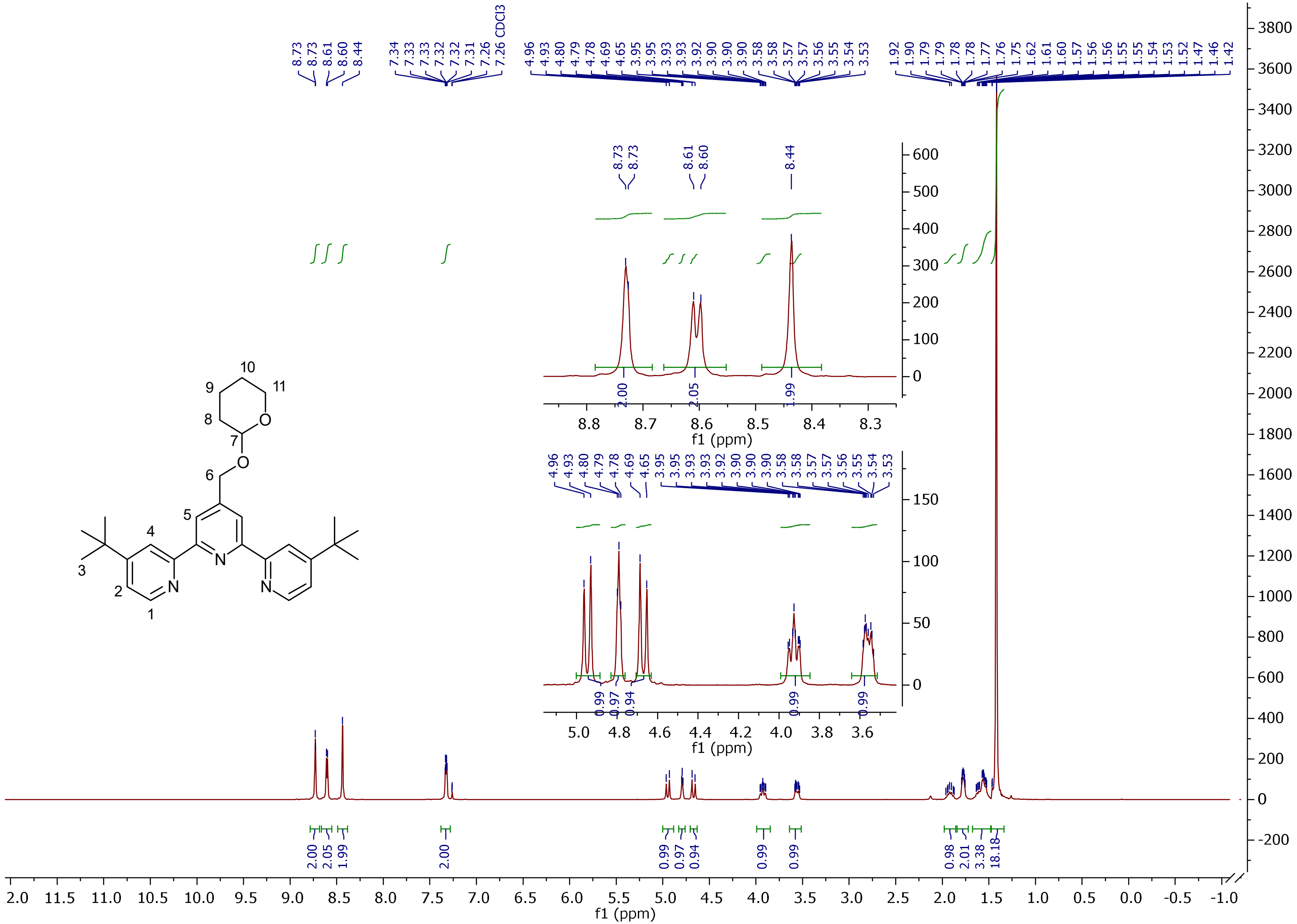

^{13}C NMR (101 MHz, $CDCl_3$) for 4,4''-di-*tert*-butyl-4'-(((tetrahydro-2H-pyran-2-yl)oxy)methyl)-2,2':6',2''-terpyridine

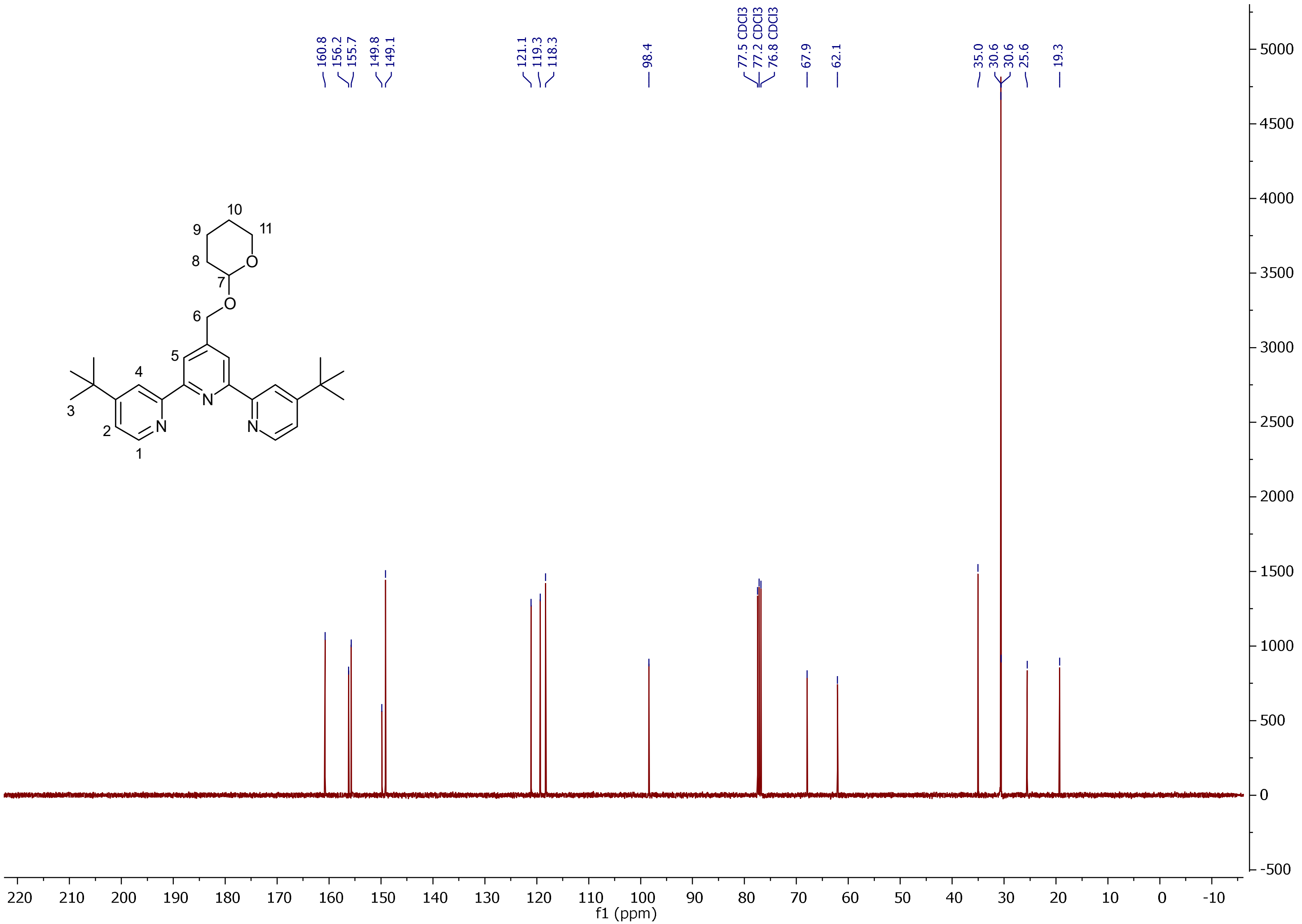

[1]**H NMR** (400 MHz, $CDCl_3$) for 4'-(bromomethyl)-4,4"-di-*tert*-butyl-2,2':6',2"-terpyridine

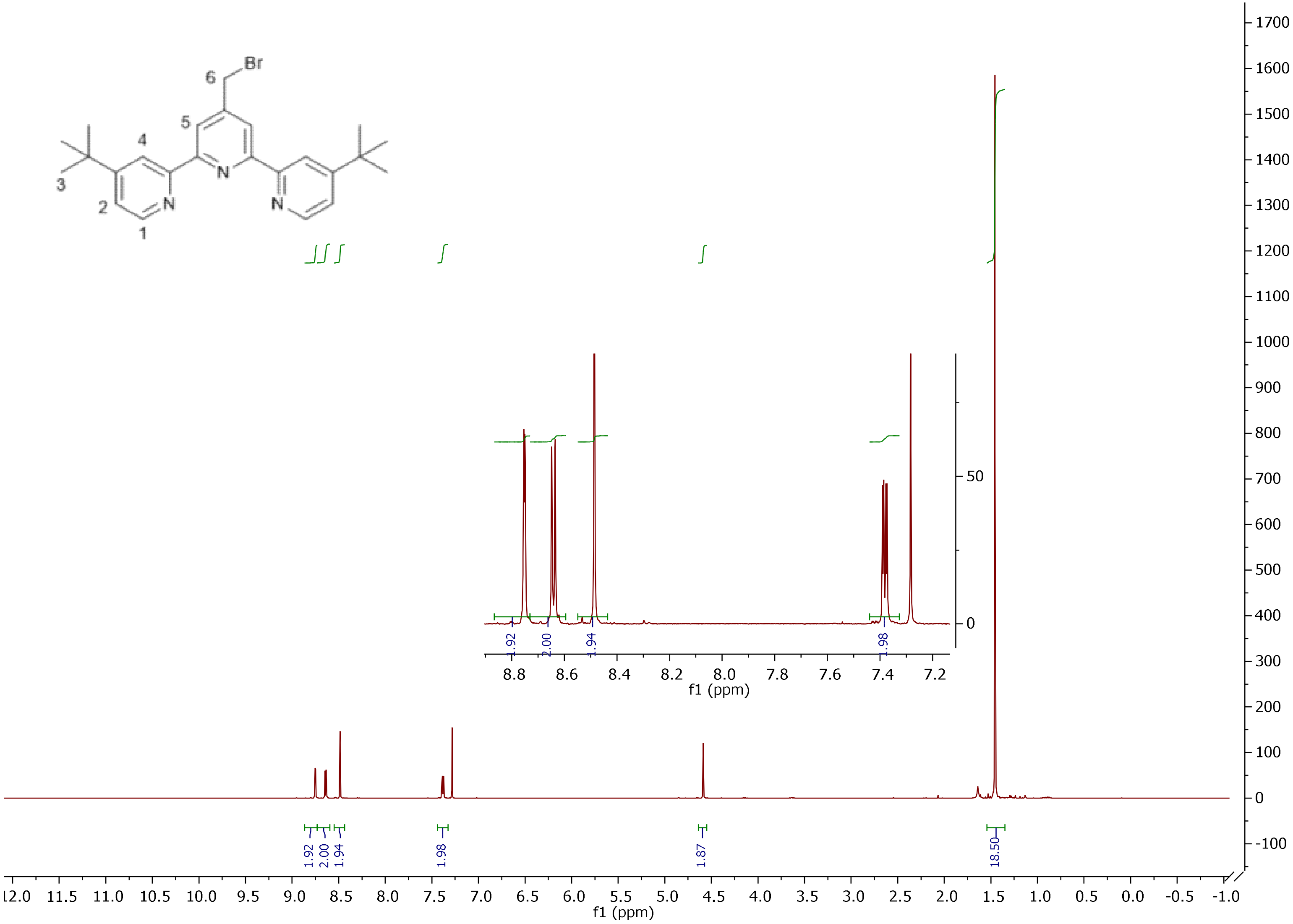

^{13}C NMR (101 MHz, $CDCl_3$) for 4'-(bromomethyl)-4,4''-di-*tert*-butyl-2,2':6',2''-terpyridine

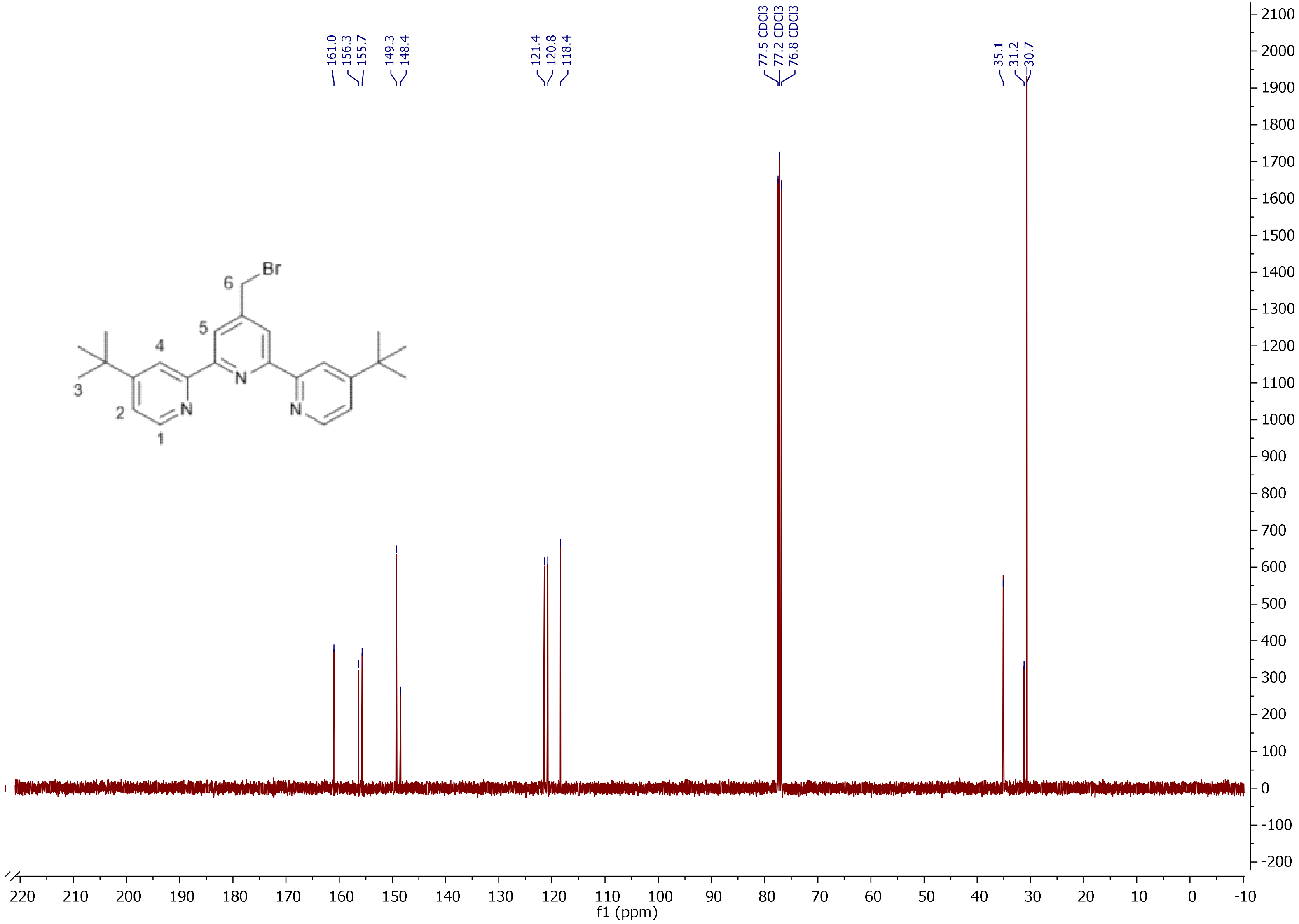

^{1}**H NMR** (400 MHz, DMSO-d^6) for sodium (4,4''-di-*tert*-butyl-[2,2':6',2''-terpyridin]-4'-yl)methanesulfonate **(L10)**

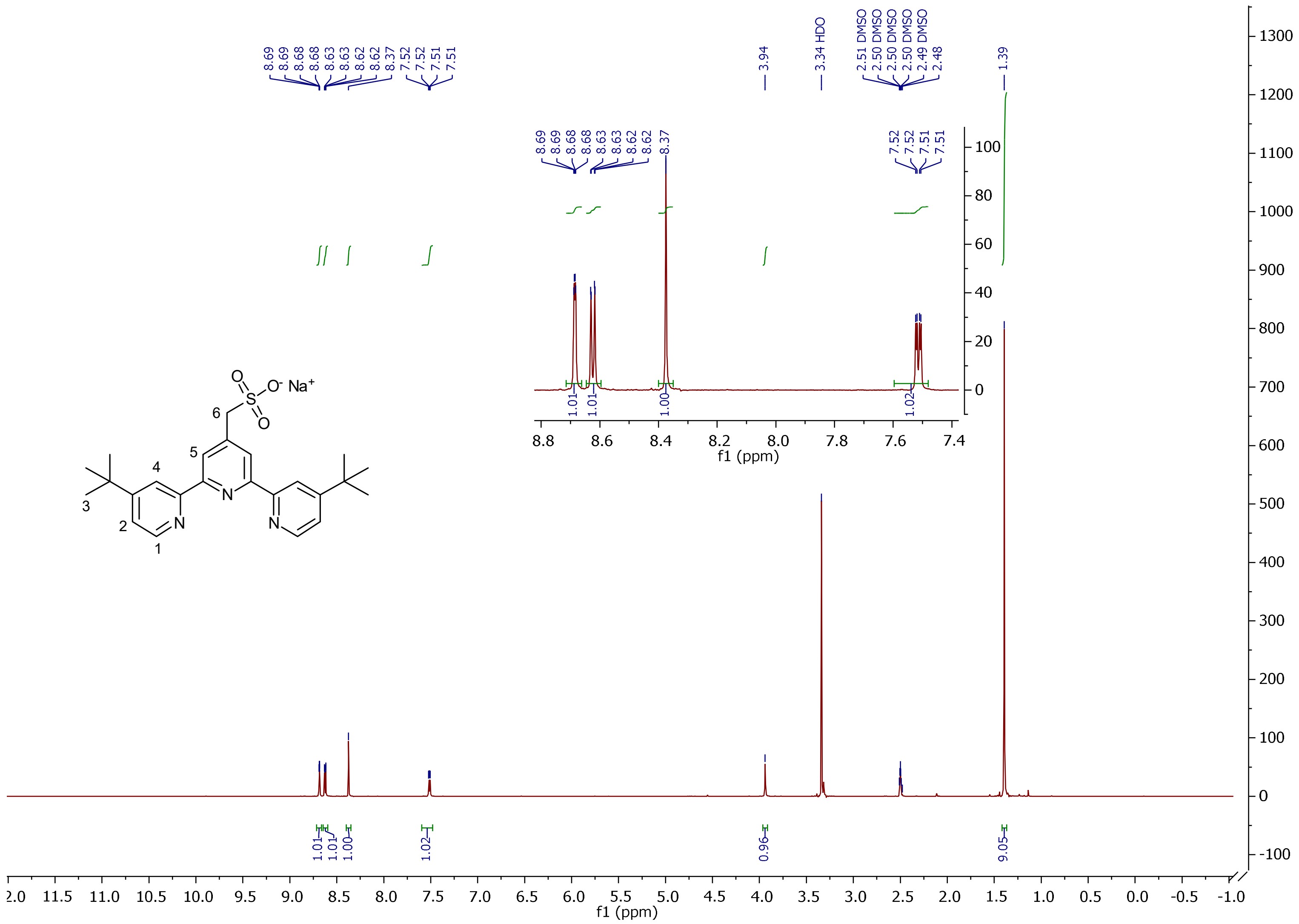

^{13}C NMR (101 MHz, DMSO-*d*^{6}) for sodium (4,4''-di-*tert*-butyl-[2,2':6',2''-terpyridin]-4'-yl)methanesulfonate **(L10)**

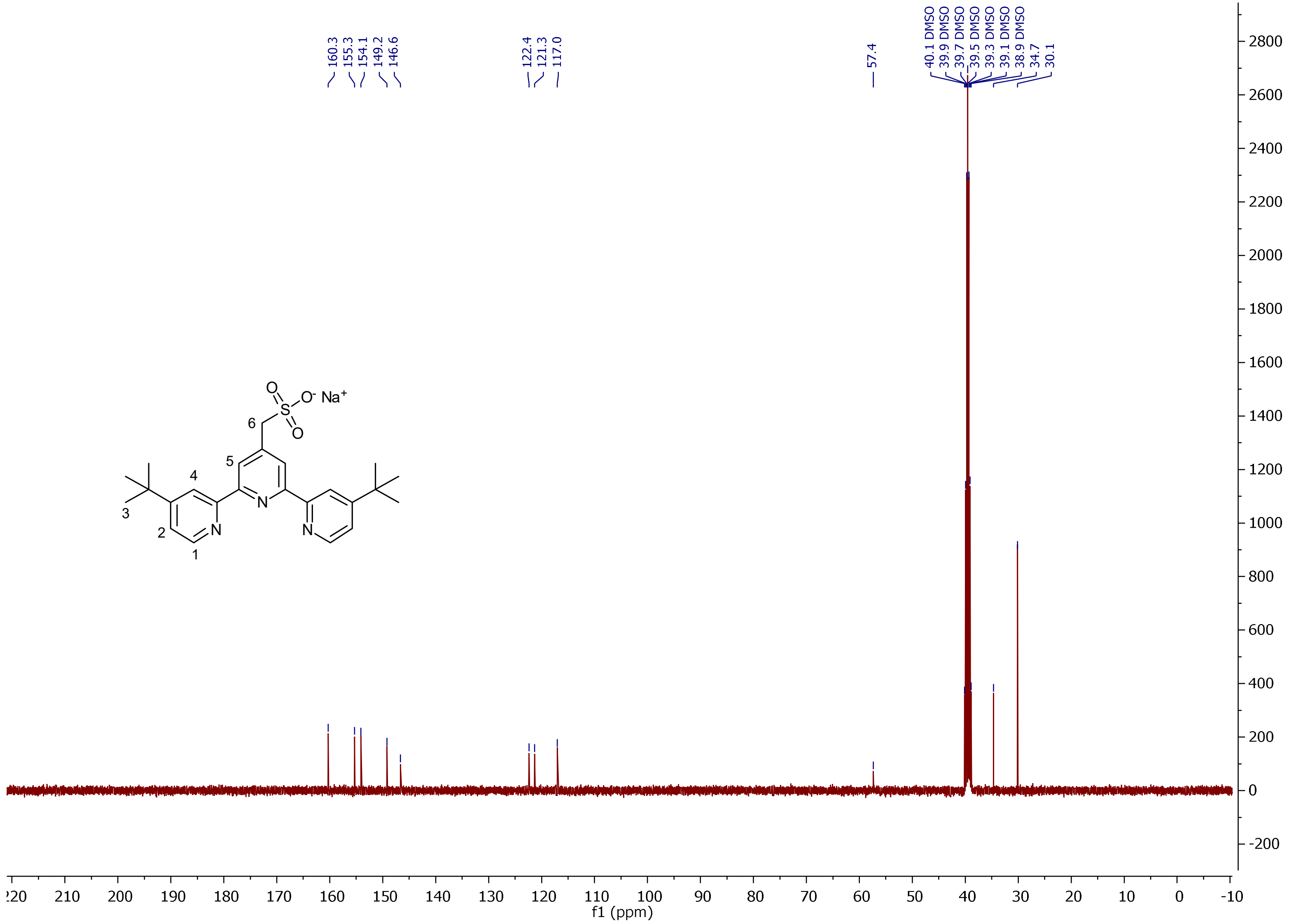

^{1}H NMR (500 MHz, CD_3CN) for [(2,2':6',2''-terpyridine)(acetonitrile)palladium](2+) tetrafluoroborate **(Complex 1)**

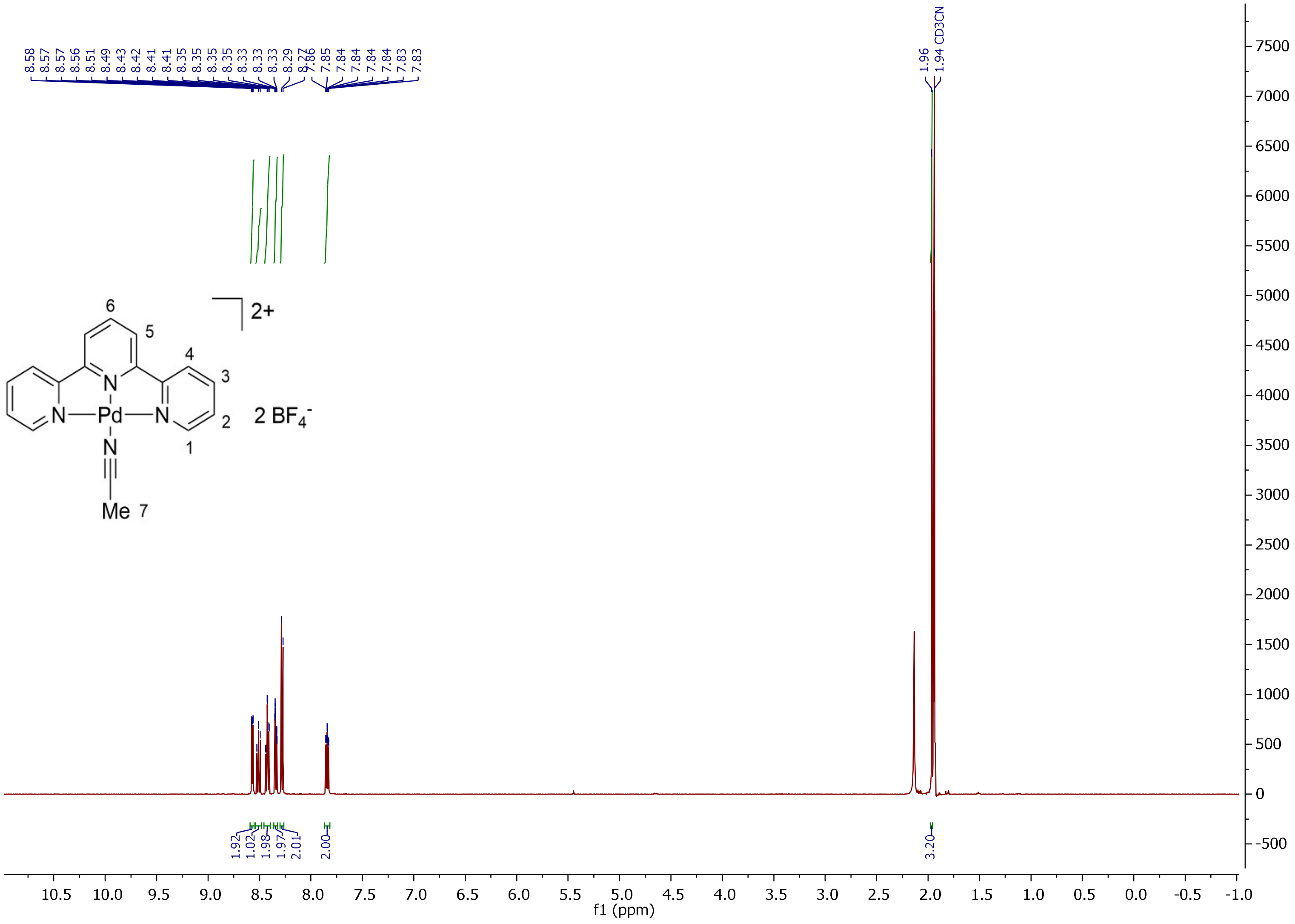

^{13}C NMR (126 MHz, DMSO-d^6) for [(2,2':6',2"-terpyridine)(acetonitrile)palladium](2+) tetrafluoroborate **(Complex 1)**

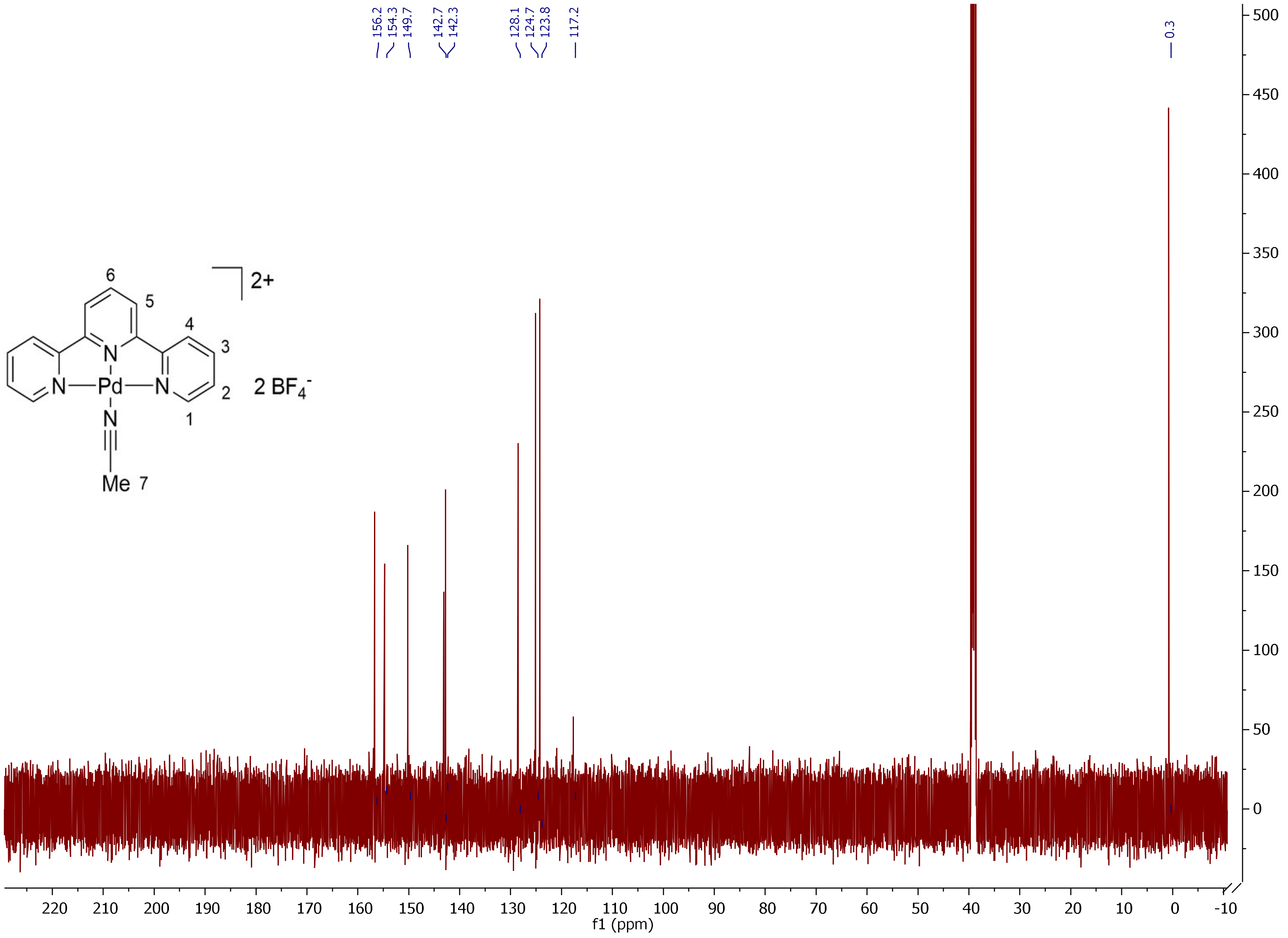

^{19}F NMR (471 MHz, DMSO-d^6) for [(2,2':6',2''-terpyridine)(acetonitrile)palladium](2+) tetrafluoroborate **(Complex 1)**

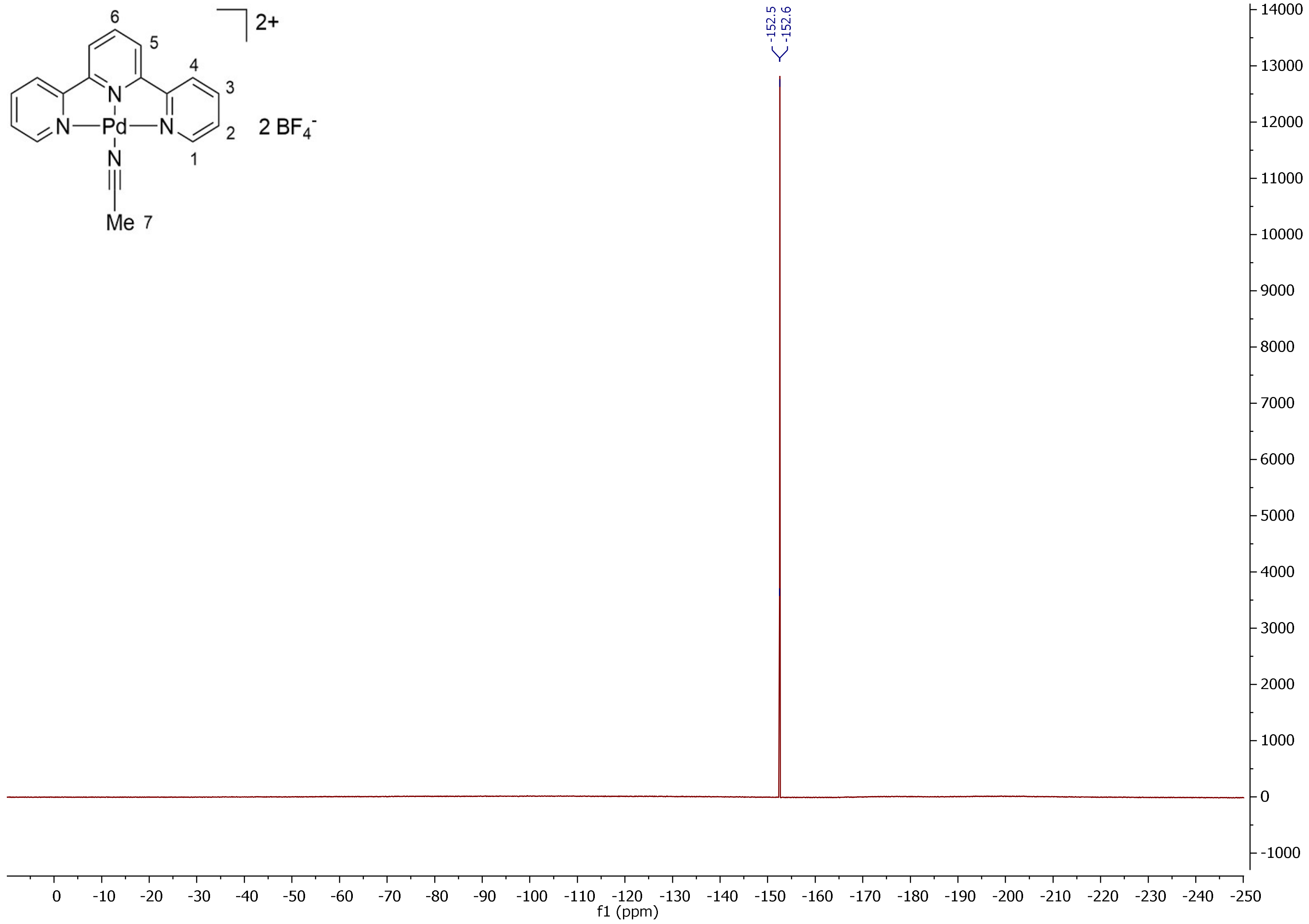

^{11}**B NMR** (160 MHz, DMSO-d^6) for [(2,2':6',2"-terpyridine)(acetonitrile)palladium](2+) tetrafluoroborate **(Complex 1)**

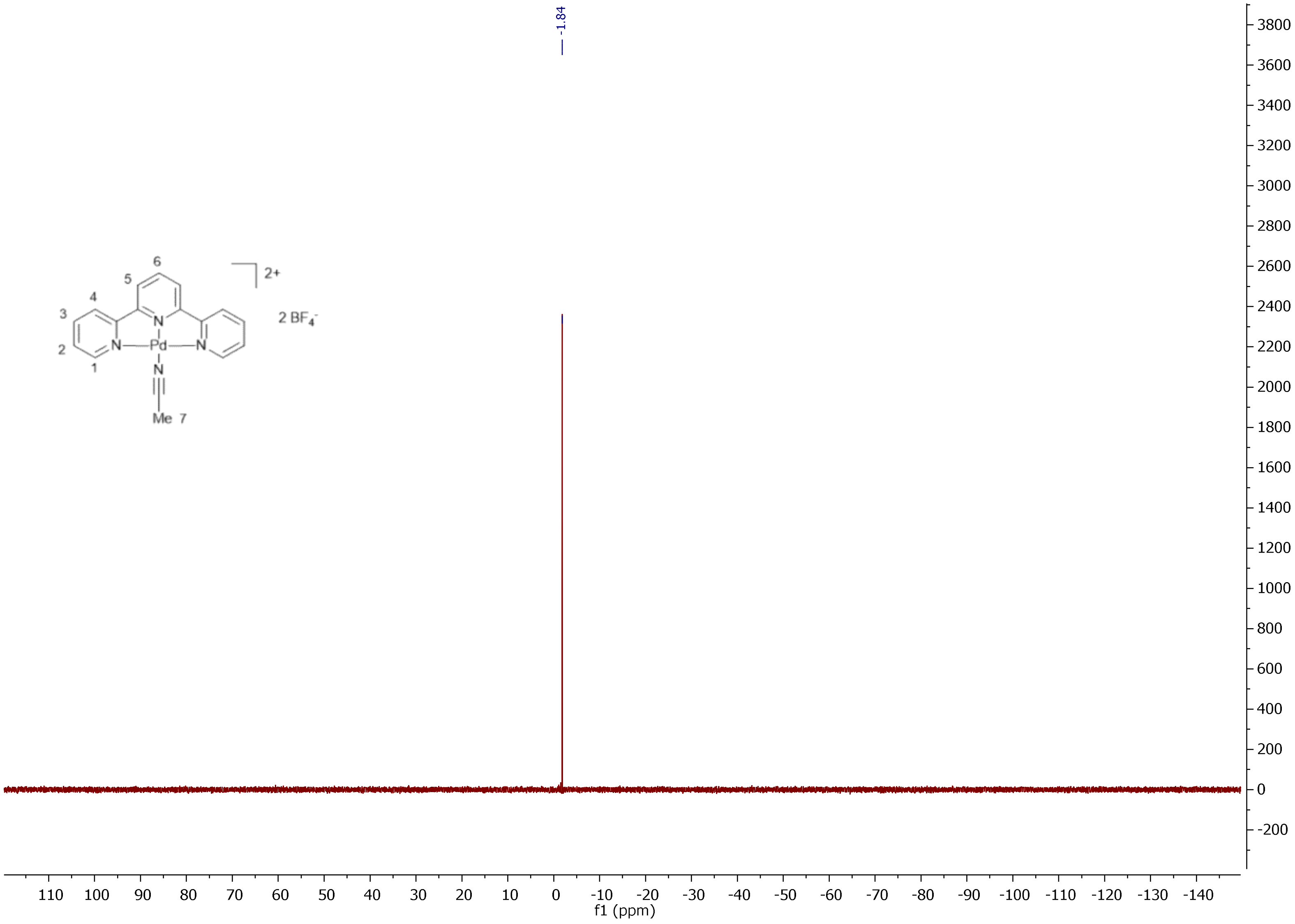

^{1}**H NMR** (400 MHz, DMSO-d^6) for [((2,2':6',2''-terpyridin)-4'-ylmethanesulfonate)(acetonitrile)palladium](+) trifluoromethanesulfonate **(Complex 2)**

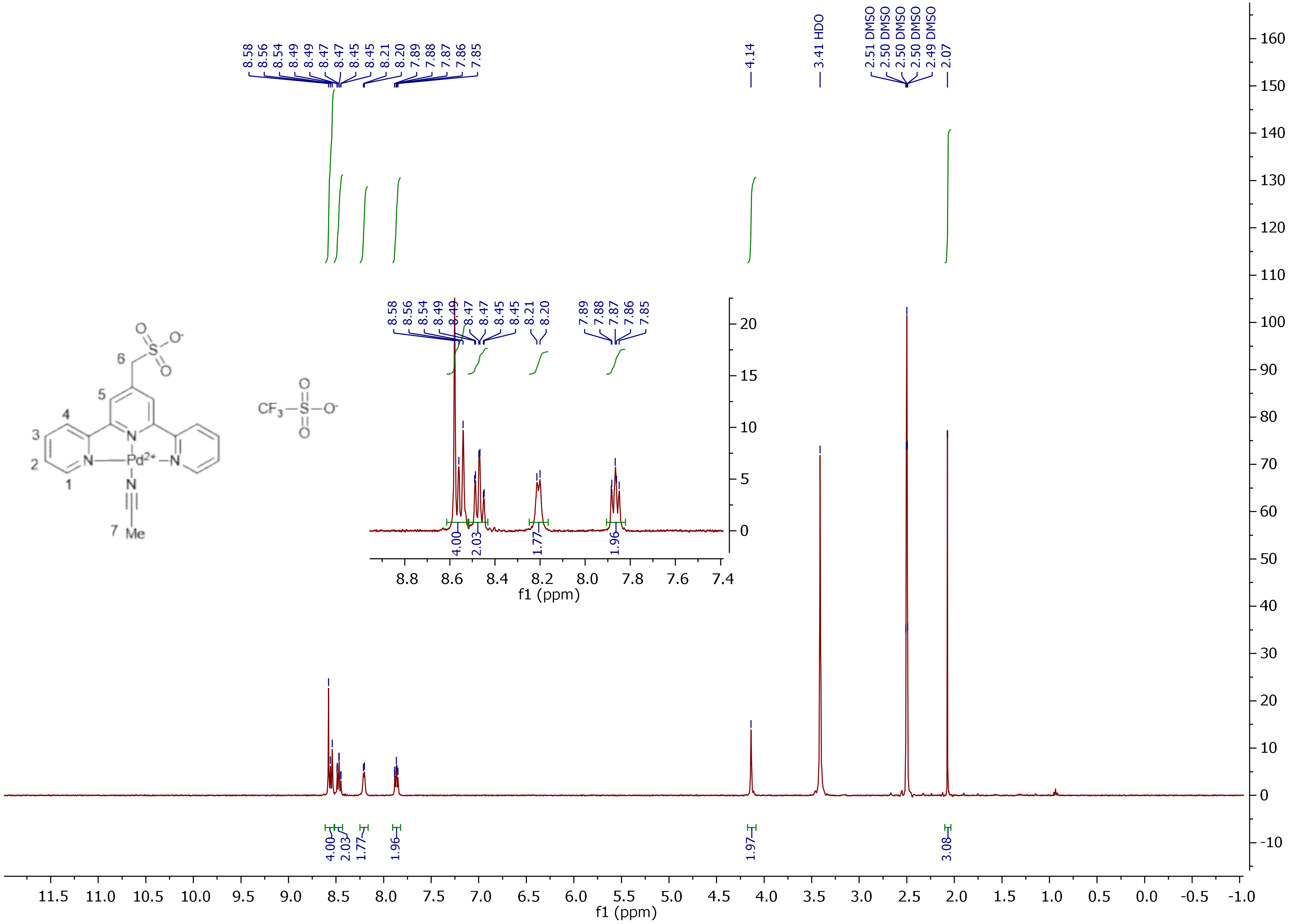

^{13}C NMR (176 MHz, DMSO-d^6) for [((2,2':6',2''-terpyridin)-4'-ylmethanesulfonate)(acetonitrile)palladium](+) trifluoromethanesulfonate **(Complex 2)**

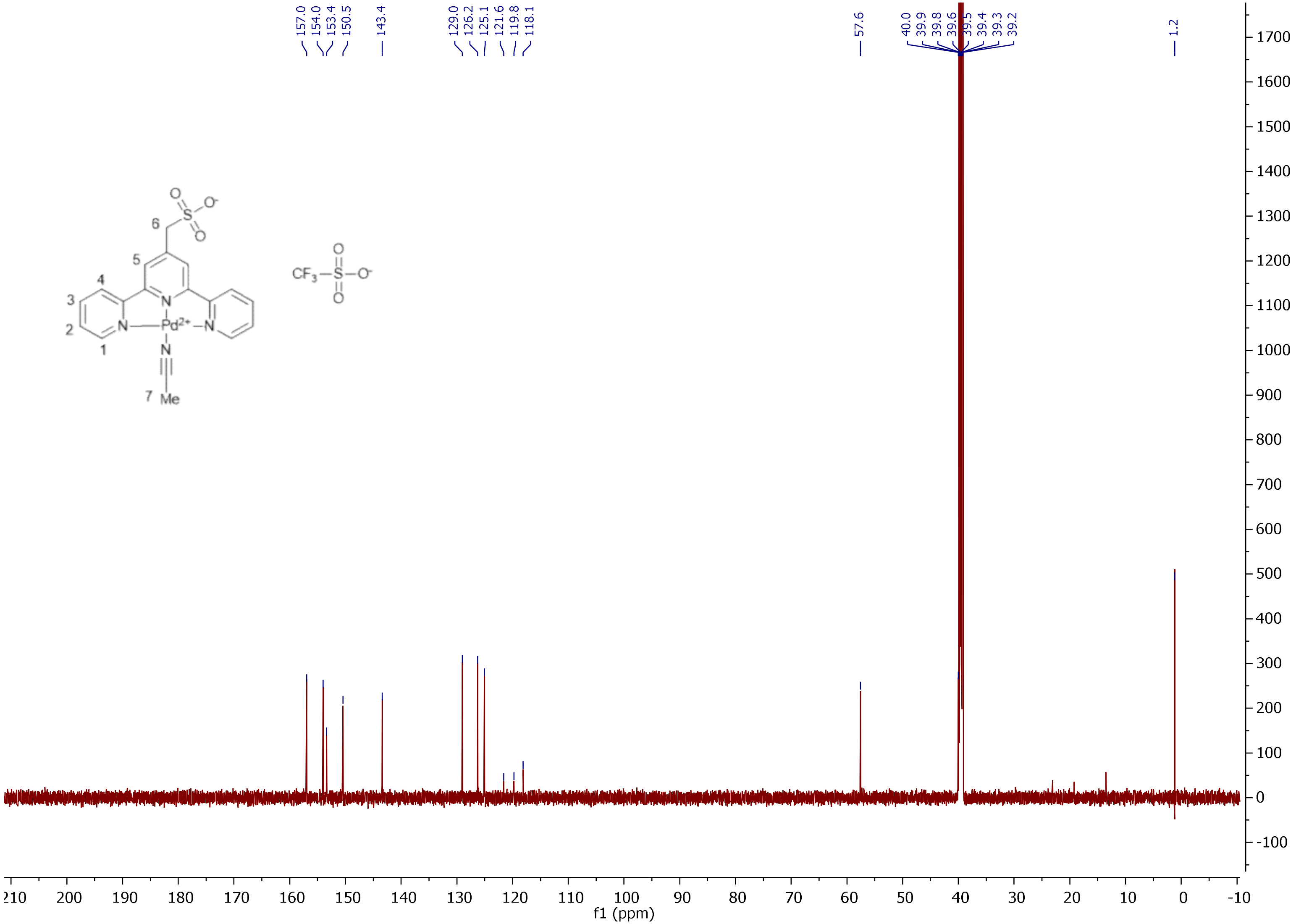

^{19}F NMR (471 MHz, DMSO-d^6) for [((2,2':6',2''-terpyridin)-4'-ylmethanesulfonate)(acetonitrile)palladium](+) trifluoromethanesulfonate **(Complex 2)**

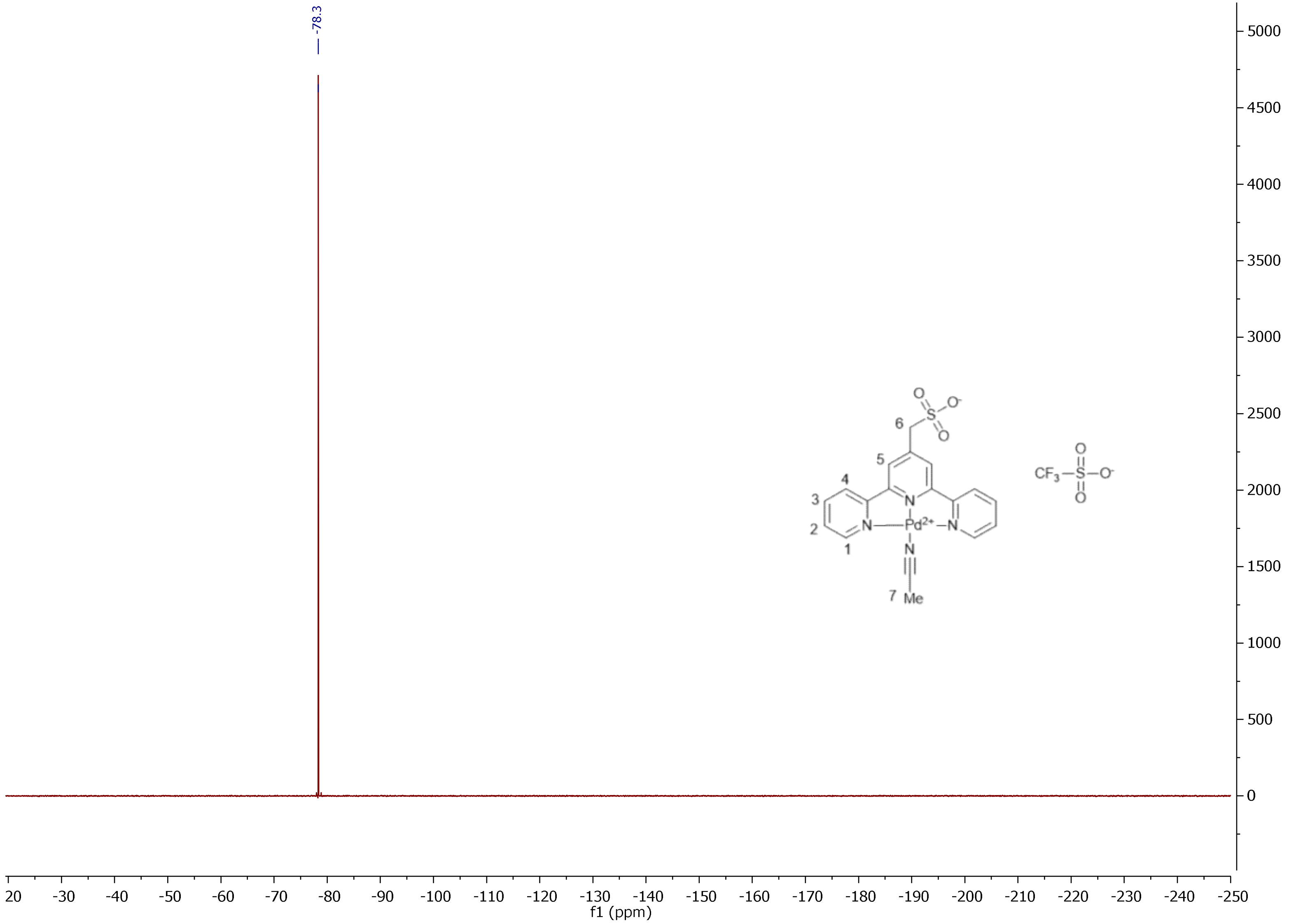

^{1}**H NMR** (500 MHz, CD_3CN) for [(4’-methyl-2,2':6',2"-terpyridine)(acetonitrile)palladium](2+) tetrafluoroborate **(Complex 3)**

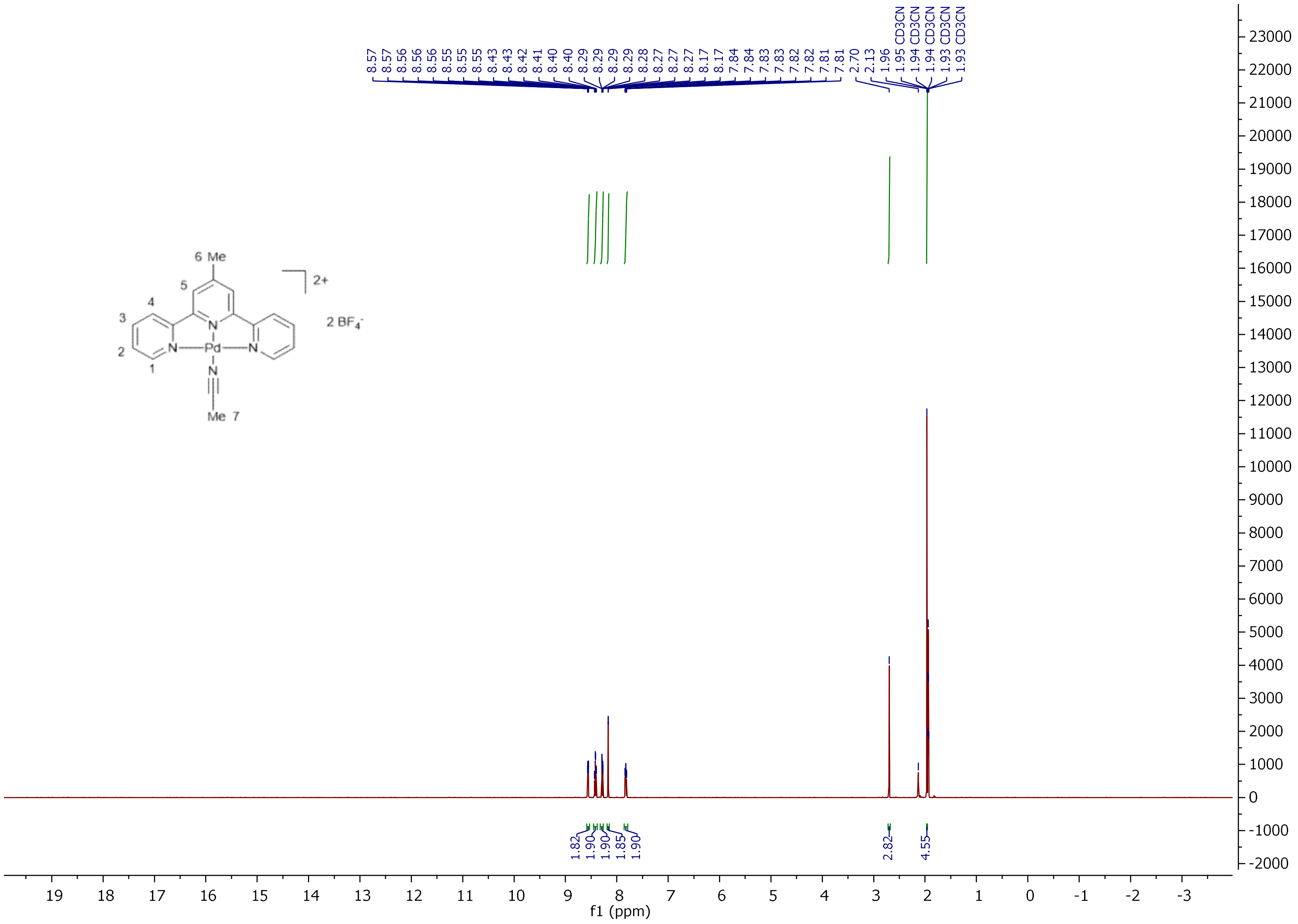

^{13}C NMR (126 MHz, DMSO-d^6) for [(4'-methyl-2,2':6',2"-terpyridine)(acetonitrile)palladium](2+) tetrafluoroborate **(Complex 3)**

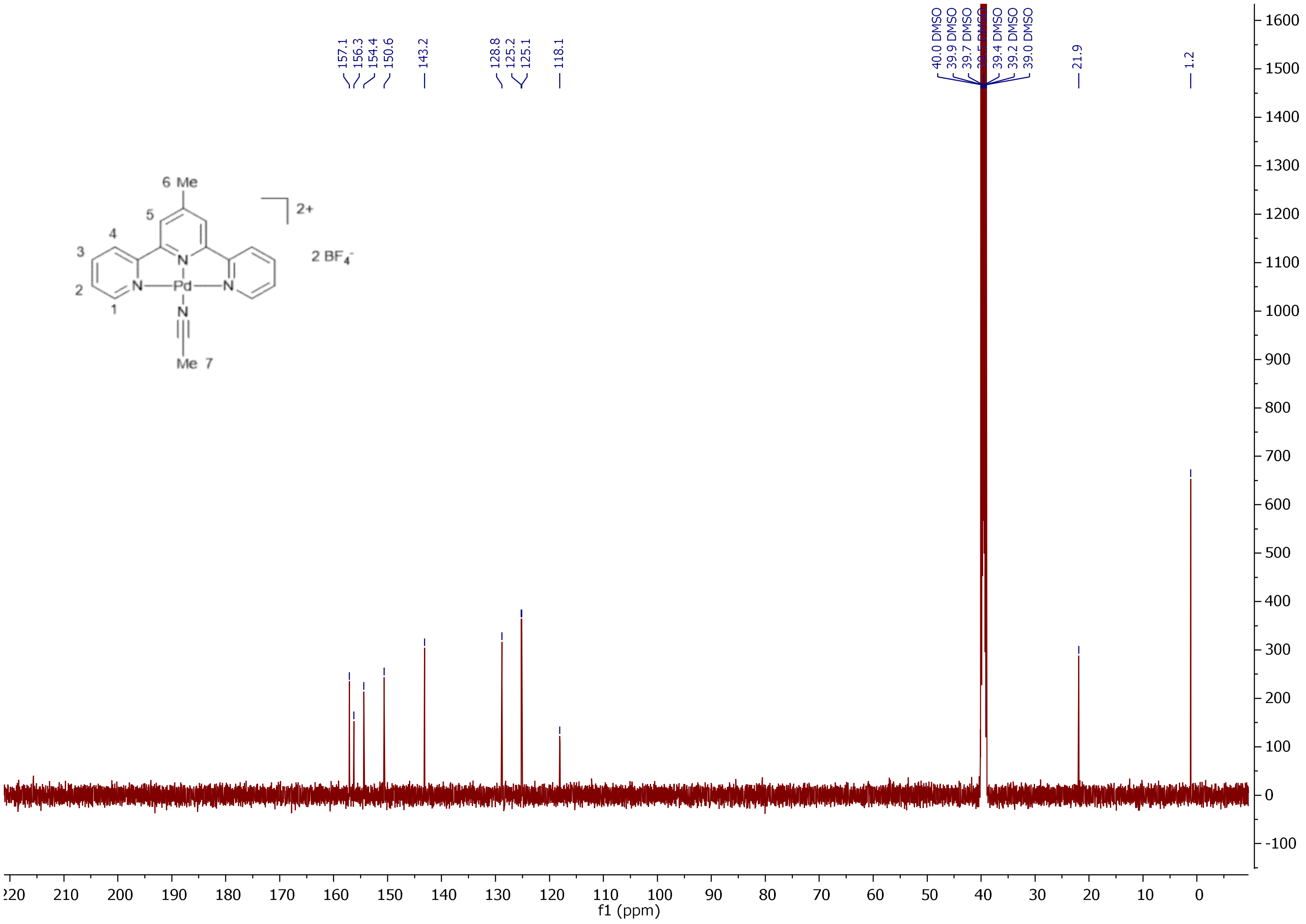

^{11}B NMR (160 MHz, DMSO-d^6) for [(2,2':6',2"-terpyridine)(acetonitrile)palladium](2+) tetrafluoroborate **(Complex 3)**

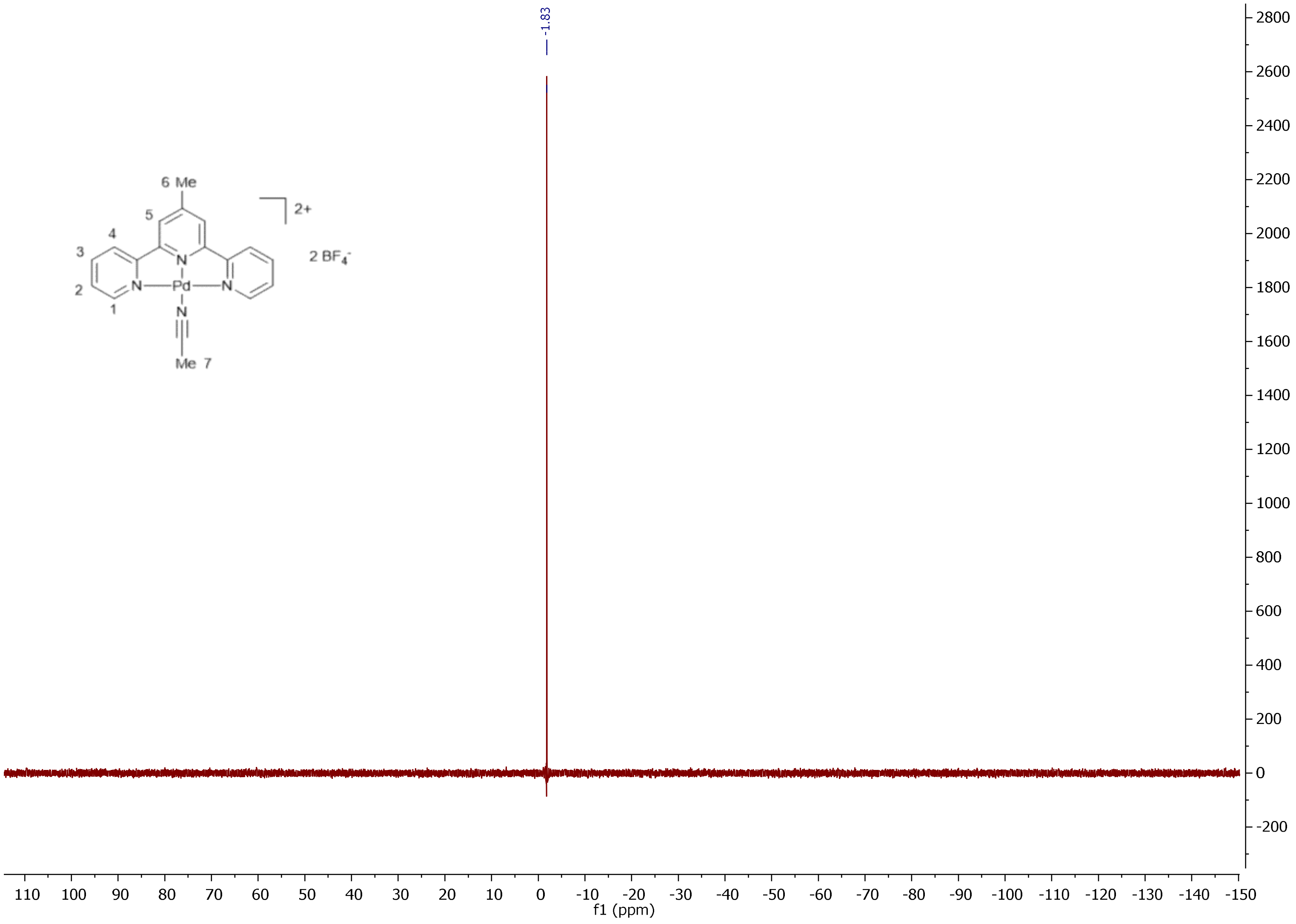

^{1}H NMR (400 MHz, DMSO-d^6) for [((4,4''-di-*tert*-butyl-[2,2':6',2''-terpyridin]-4'-yl)methanesulfonate)(acetonitrile)palladium](2+) tetrafluoroborate **(Complex 4)**

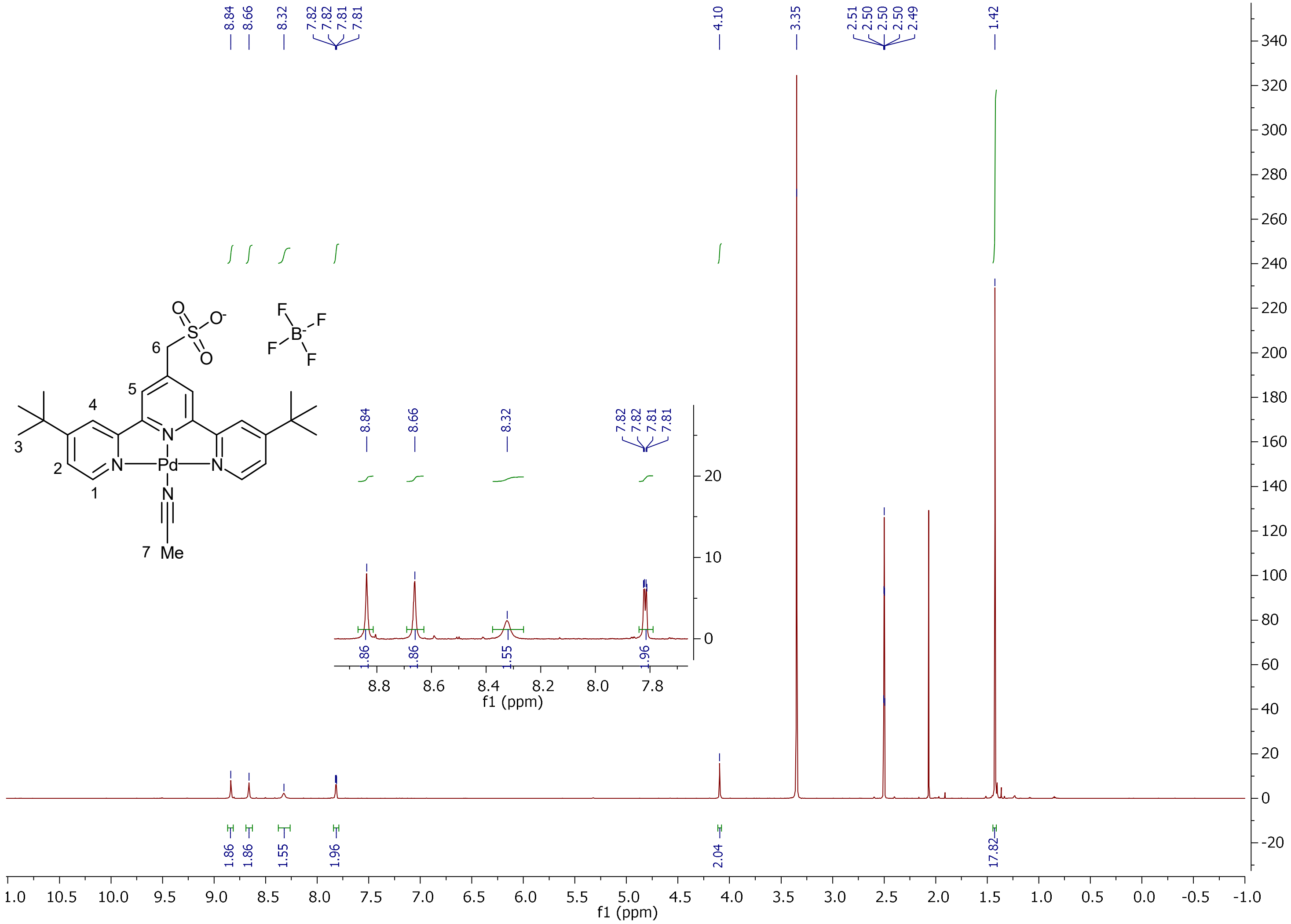

^{13}C NMR (176 MHz, DMSO-*d*^{6}) for [((4,4"-di-*tert*-butyl-[2,2':6',2"-terpyridin]-4'-yl)methanesulfonate)(acetonitrile)palladium](2+) tetrafluoroborate **(Complex 4)**

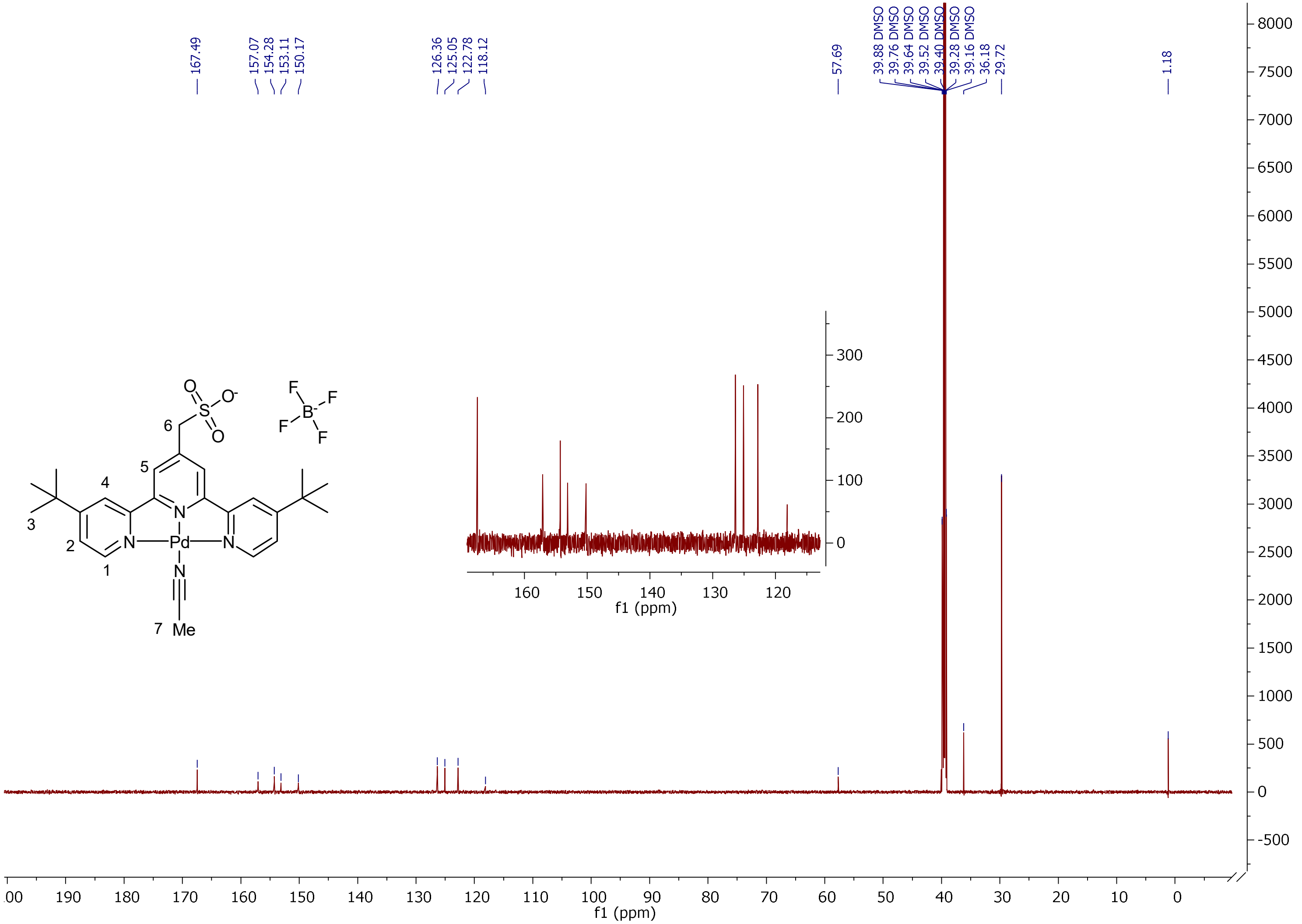

^{19}F NMR (471 MHz, DMSO-*d*^{6}) for [((4,4"-di-*tert*-butyl-[2,2':6',2"-terpyridin]-4'-yl)methanesulfonate)(acetonitrile)palladium](2+) tetrafluoroborate **(Complex 4)**

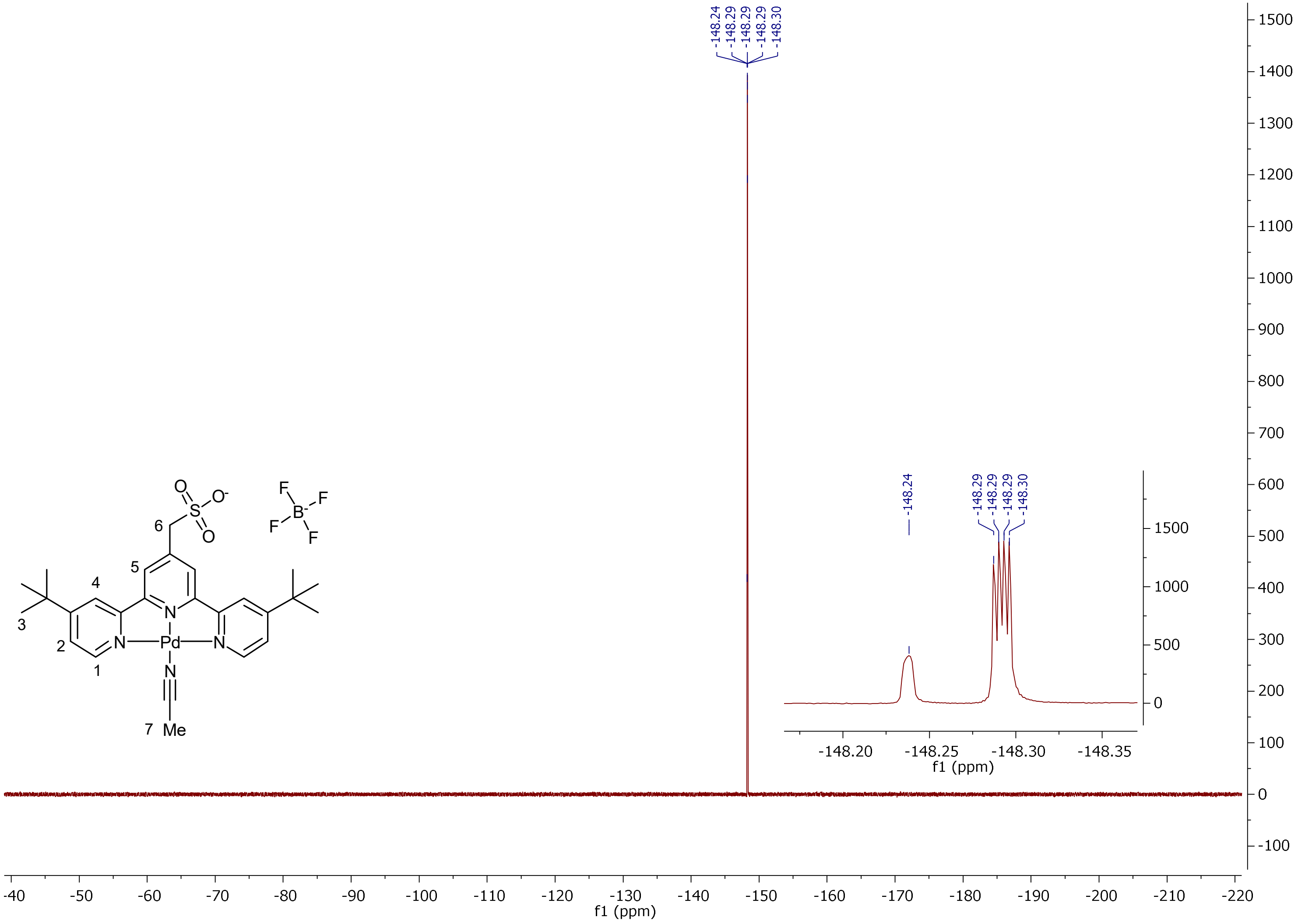

^{11}B NMR (160 MHz, DMSO-d^6) for [((4,4"-di-*tert*-butyl-[2,2':6',2"-terpyridin]-4'-yl)methanesulfonate)(acetonitrile)palladium](2+) tetrafluoroborate **(Complex 4)**

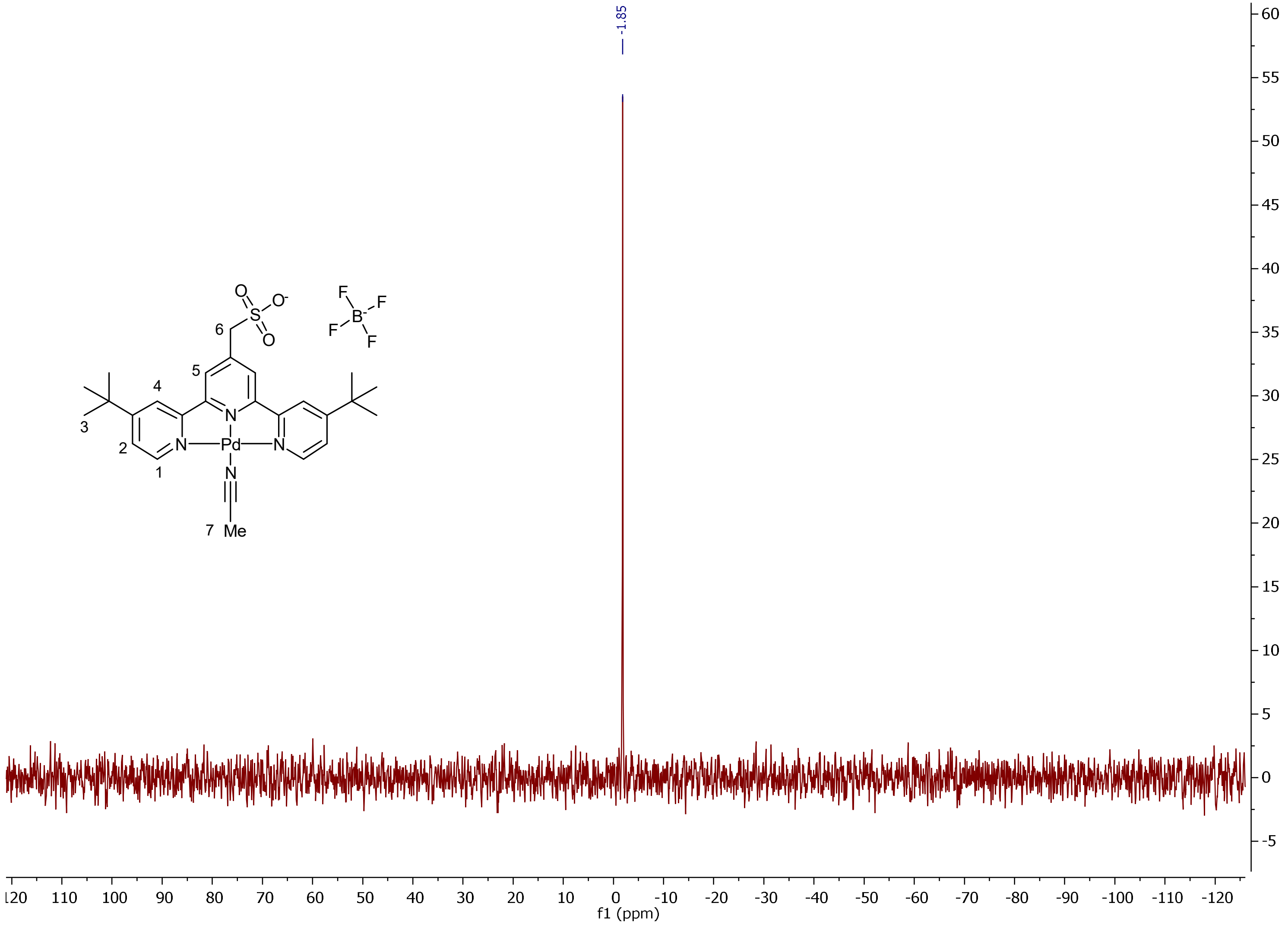

¹H NMR (400 MHz, $CDCl_3$) for 1,10-phenathrolin-2-amine

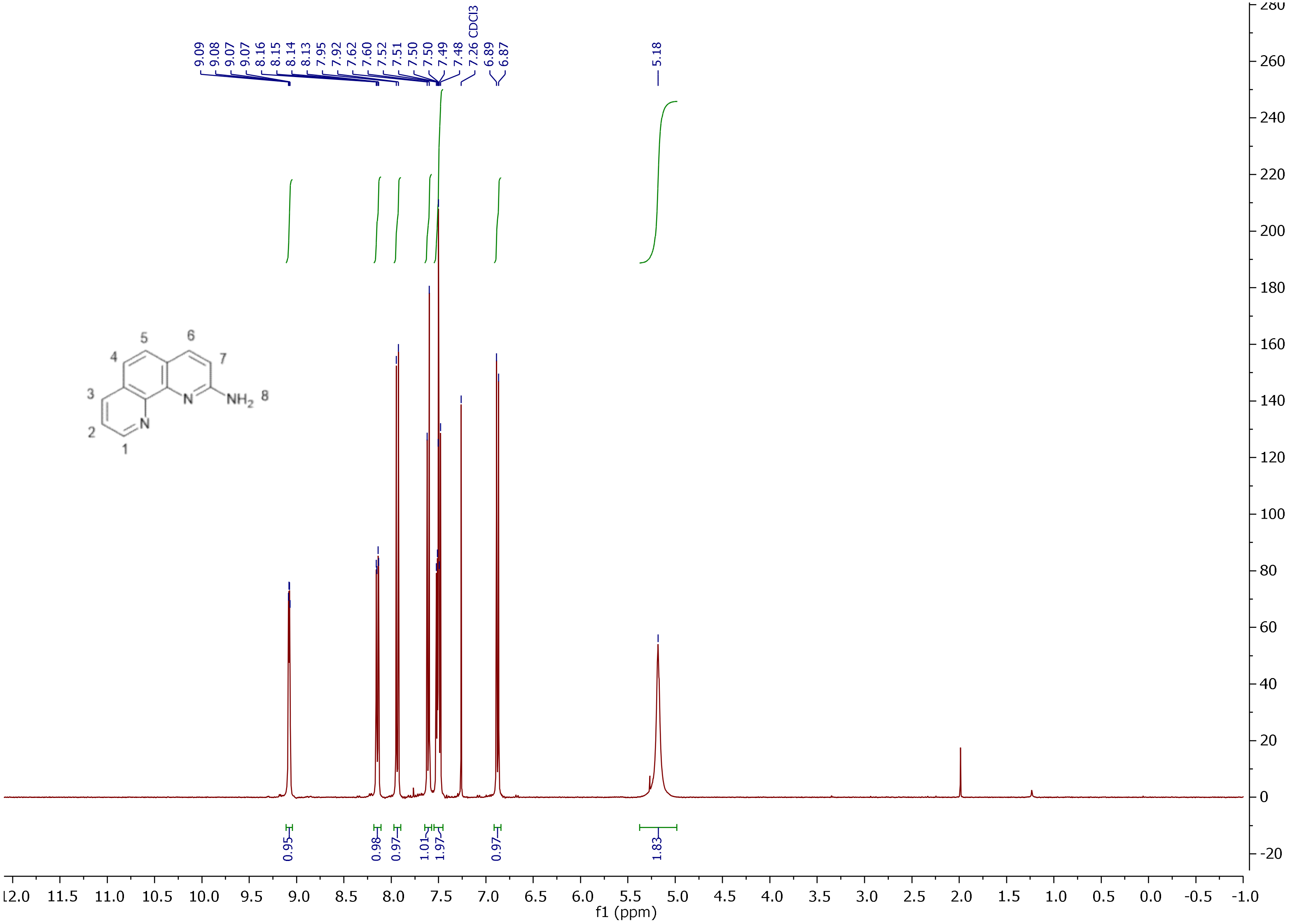

^{13}C NMR (101 MHz, $CDCl_3$) for 1,10-phenathrolin-2-amine

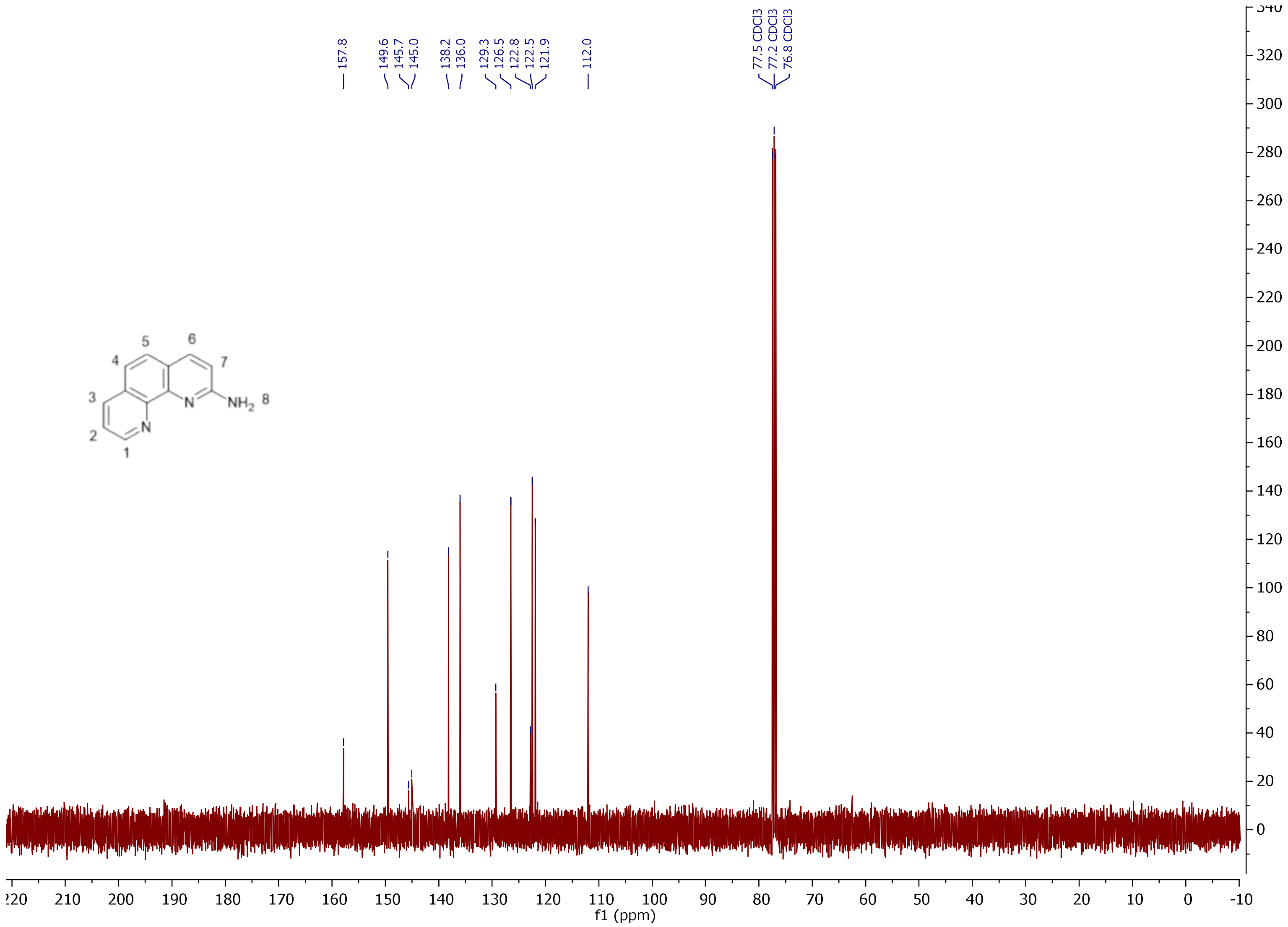

^{1}H NMR (500 MHz, $CDCl_3$) for 1,10-phenathroline-2-ol

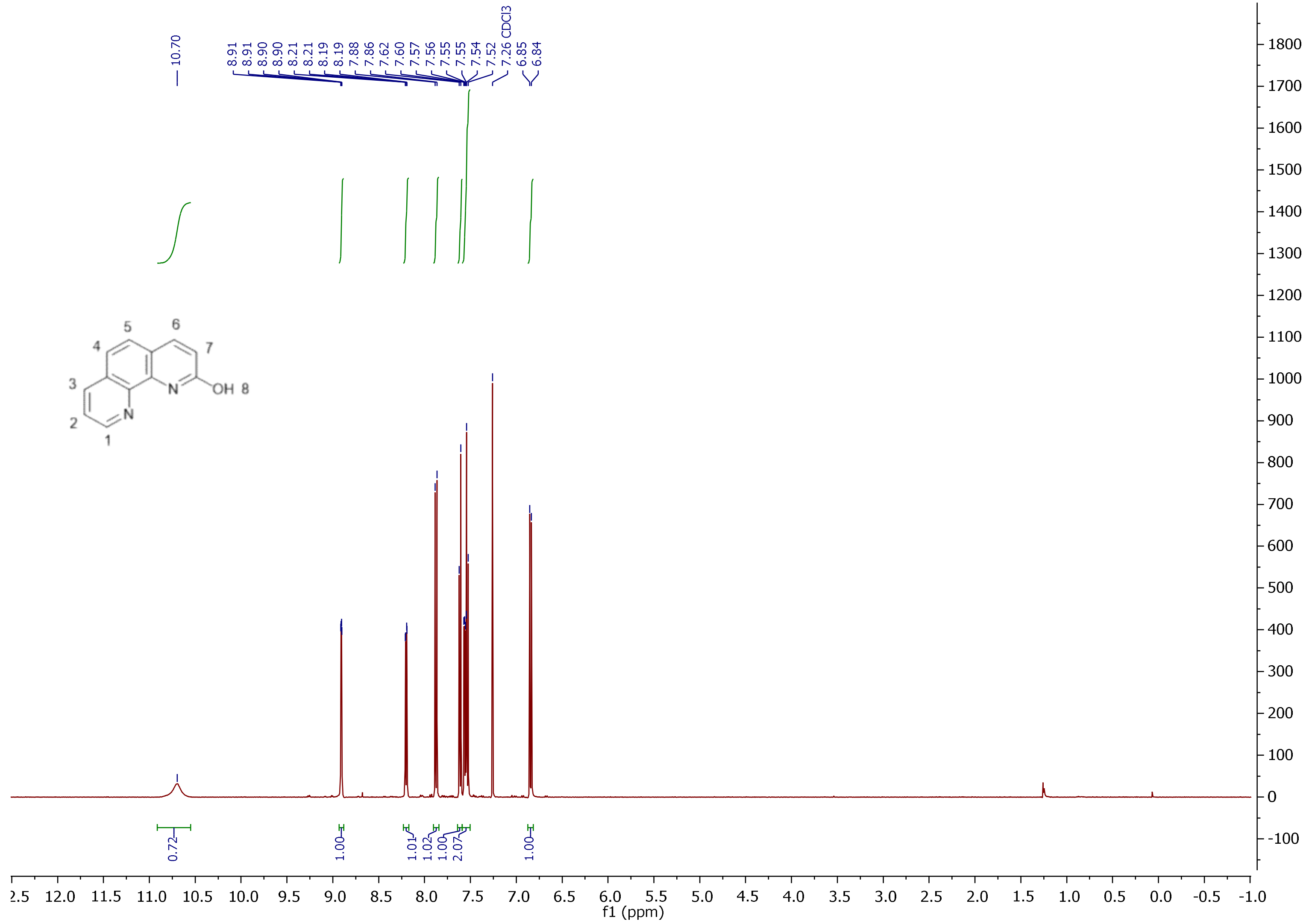

^{13}C NMR (126 MHz, $CDCl_3$) for 1,10-phenathroline-2-ol

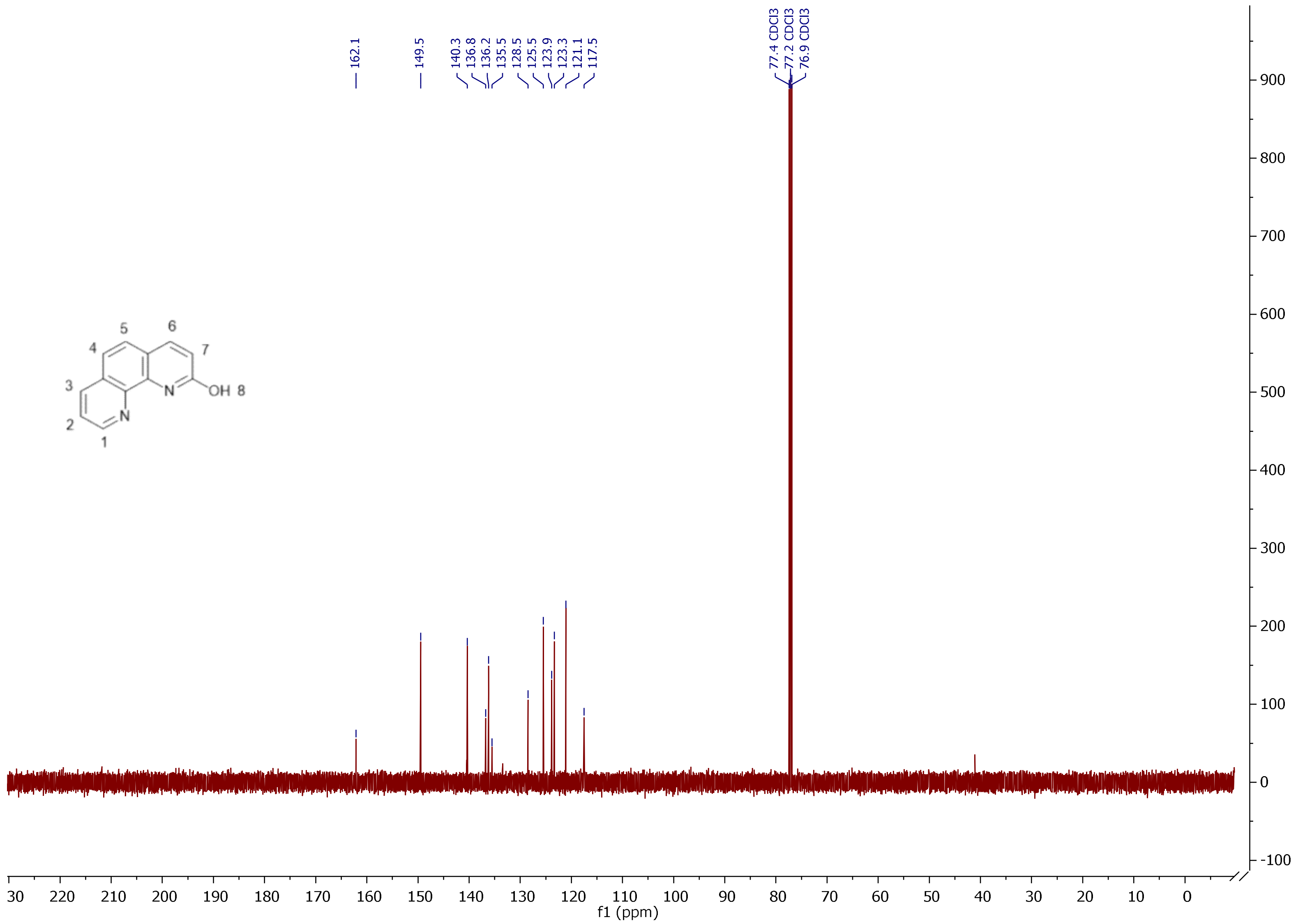

^{1}H NMR (400 MHz, $CDCl_3$) 1-(1,10-phenanthrolin-2-yl)ethan-1-one

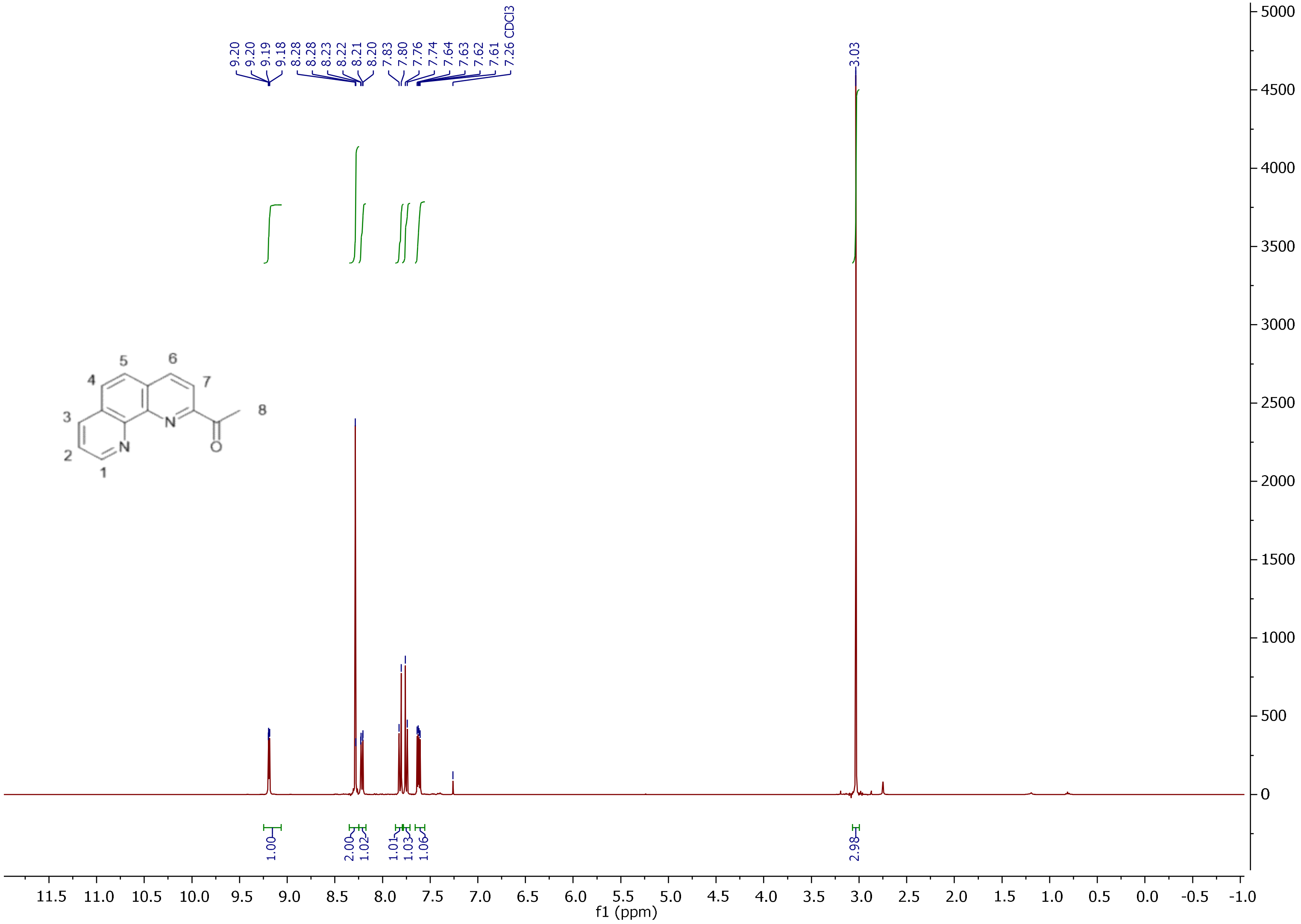

^{13}C NMR (126 MHz, $CDCl_3$) 1-(1,10-phenanthrolin-2-yl)ethan-1-one

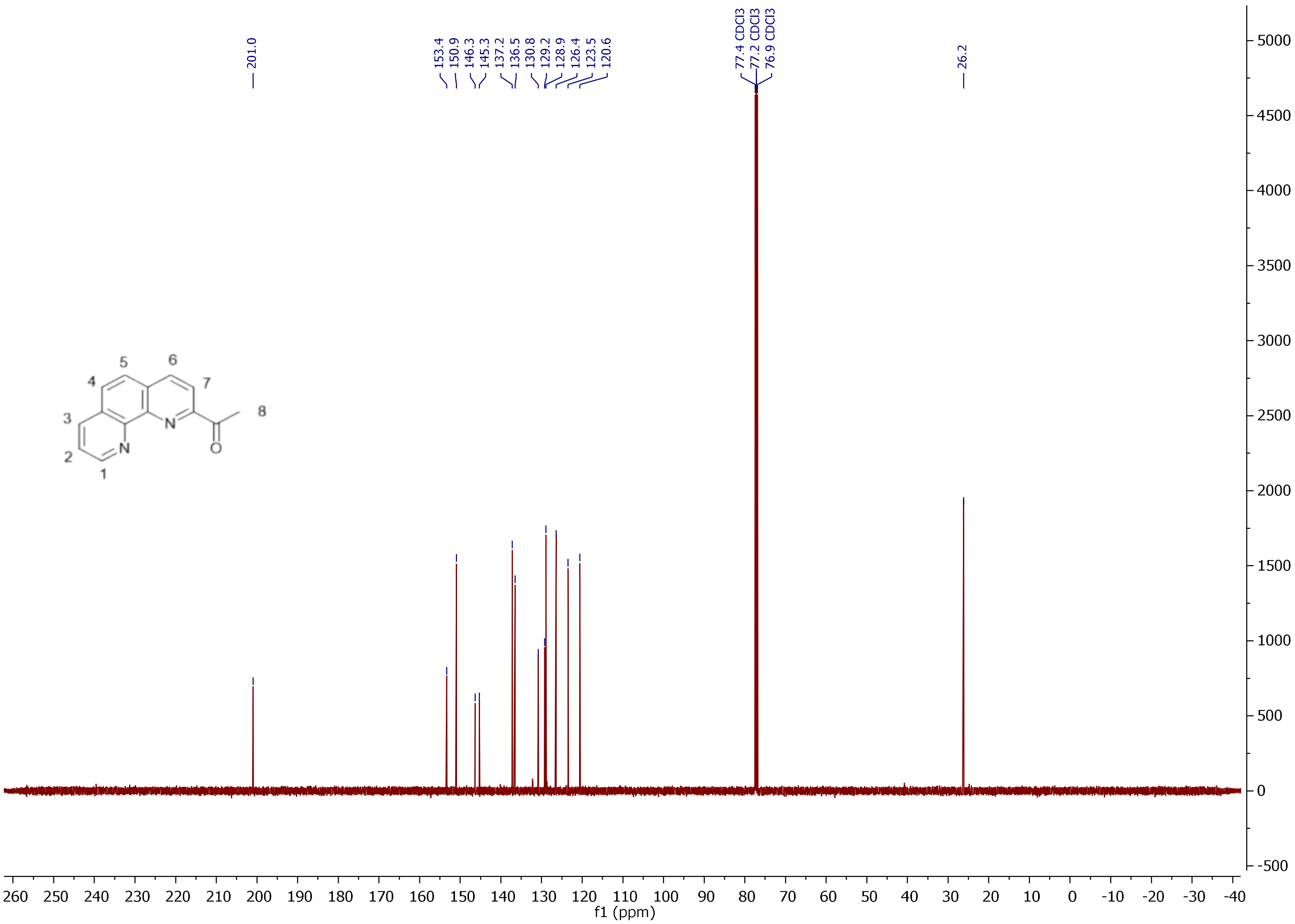

^{1}H NMR (700 MHz, $CDCl_3$) for 1,10-phenanthroline-1-oxide

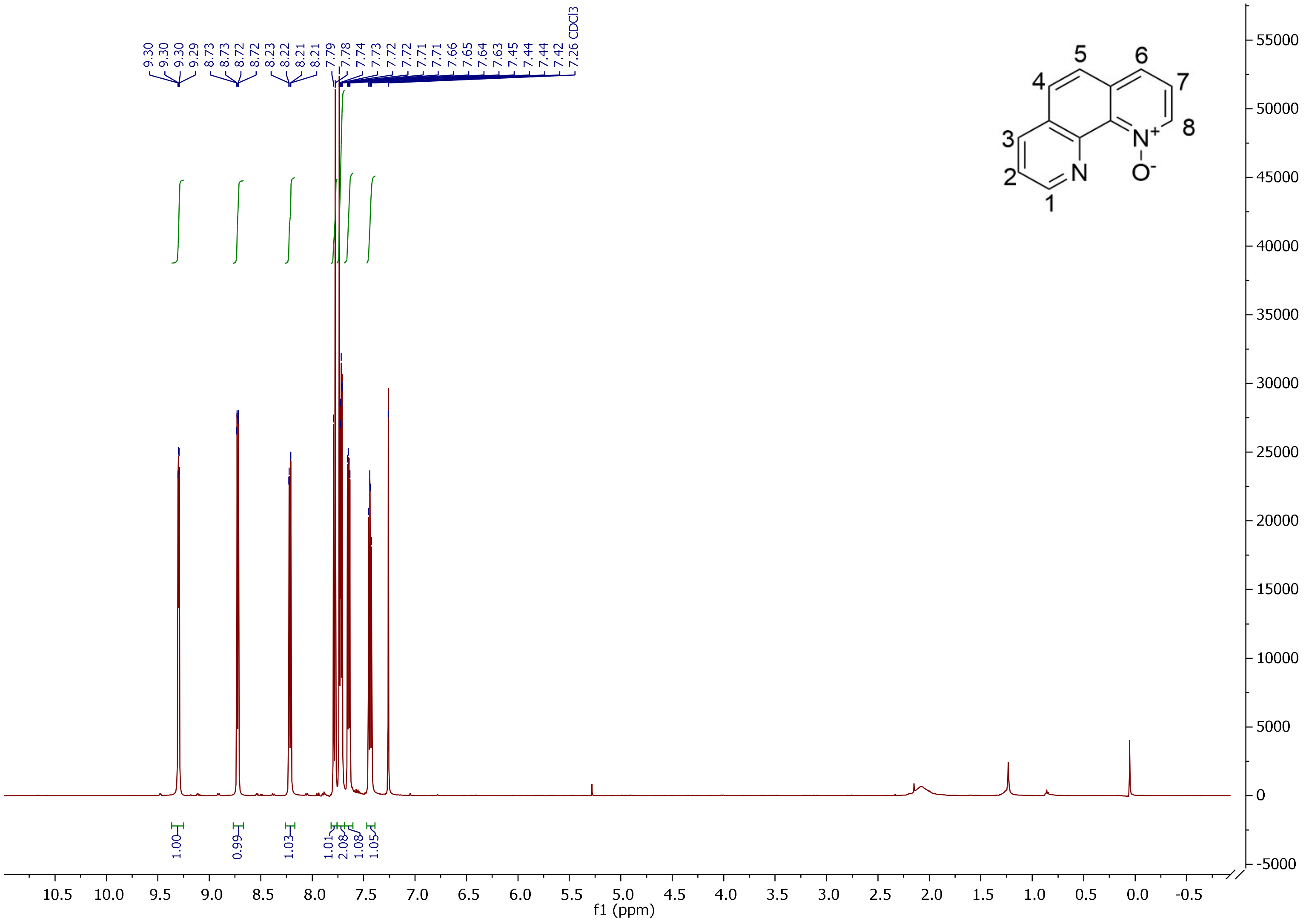

^{13}C NMR (175 MHz, $CDCl_3$) for 1,10-phenanthroline-1-oxide

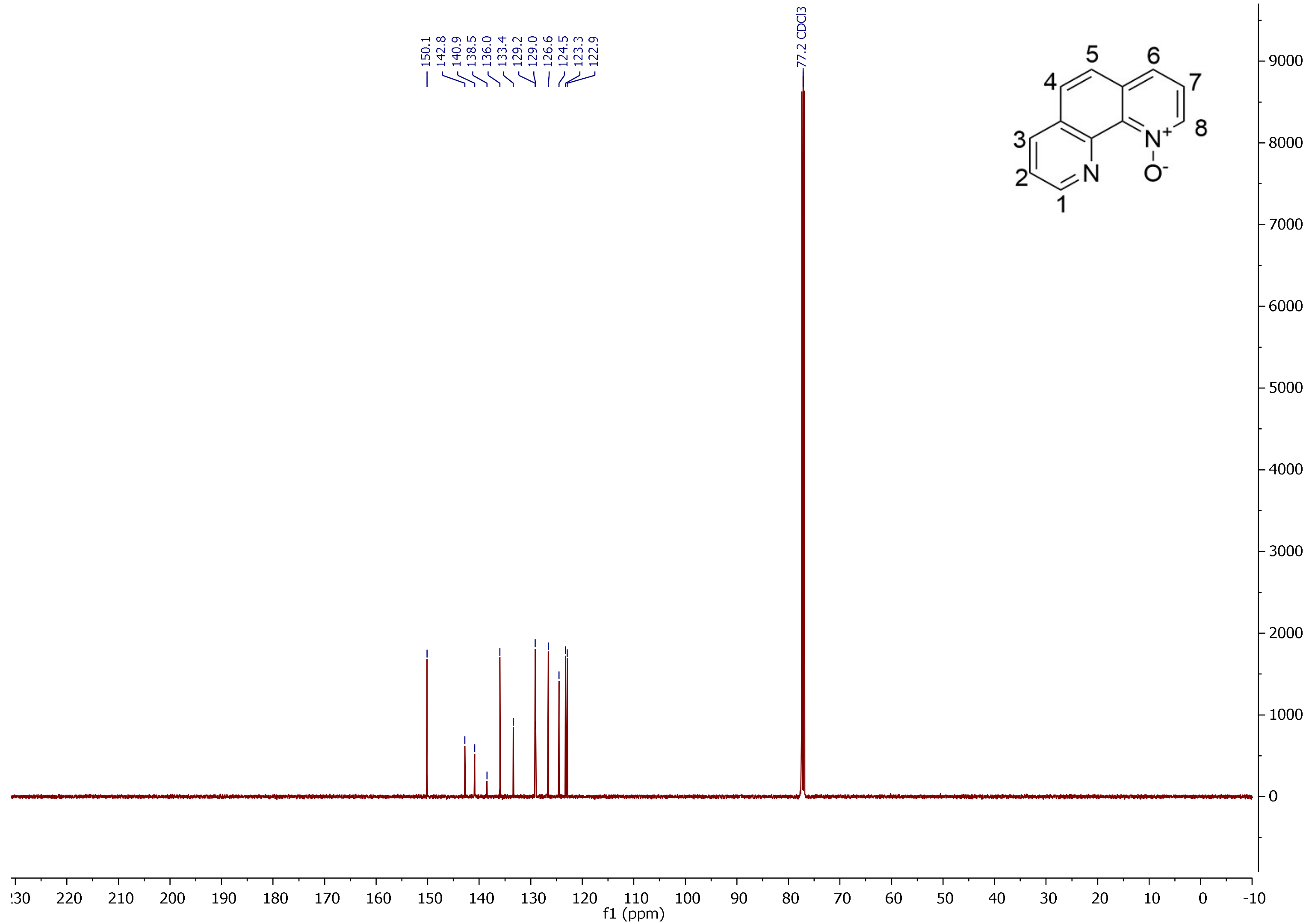

¹H NMR (500 MHz, CD_3CN) for *N*,*N*,*N*-trimethyl-1,10-phenanthrolin-2-aminium tetrafluoroborate

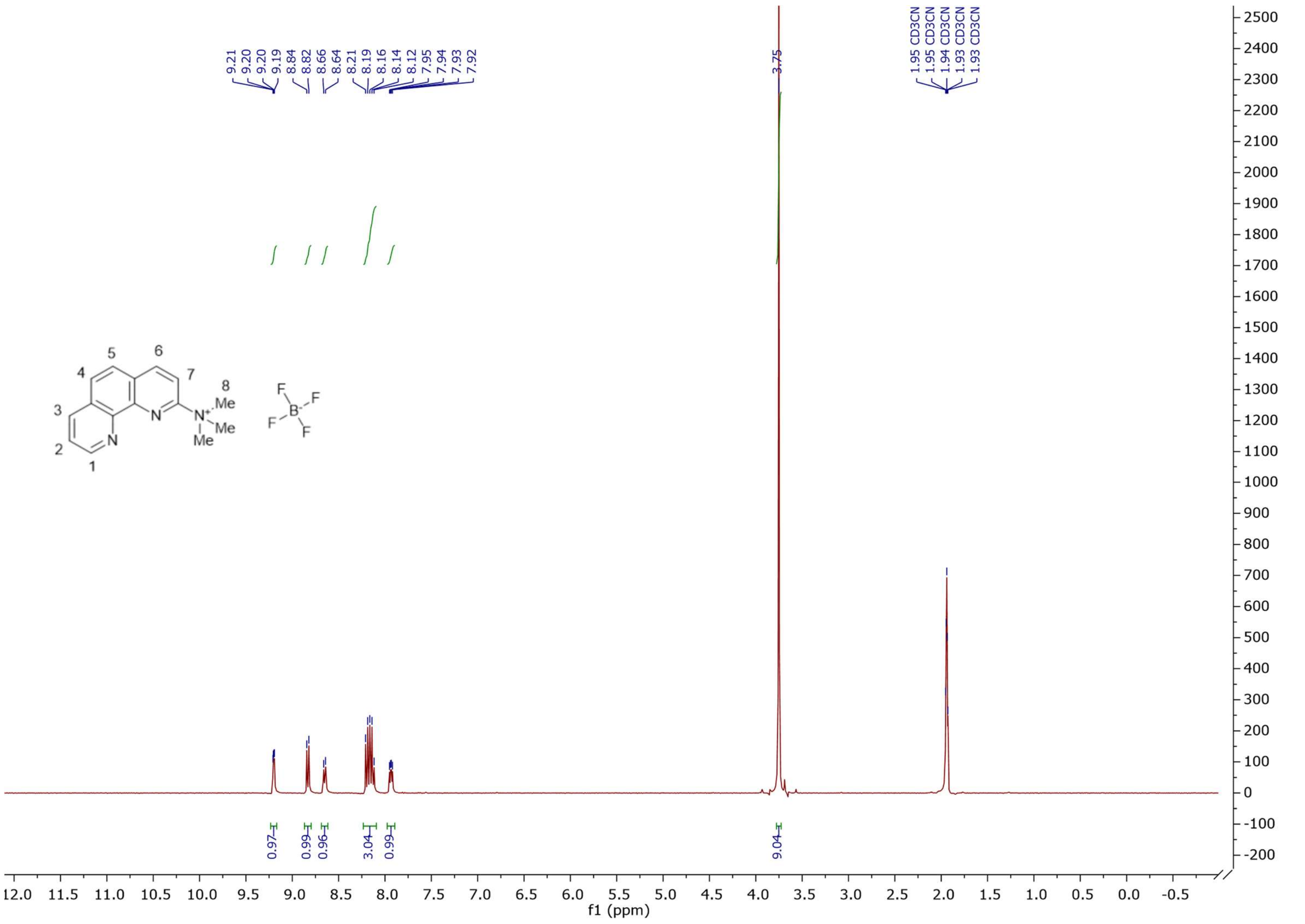

^{13}C NMR (176 MHz, CD_3CN) for *N*,*N*,*N*-trimethyl-1,10-phenanthrolin-2-aminium tetrafluoroborate

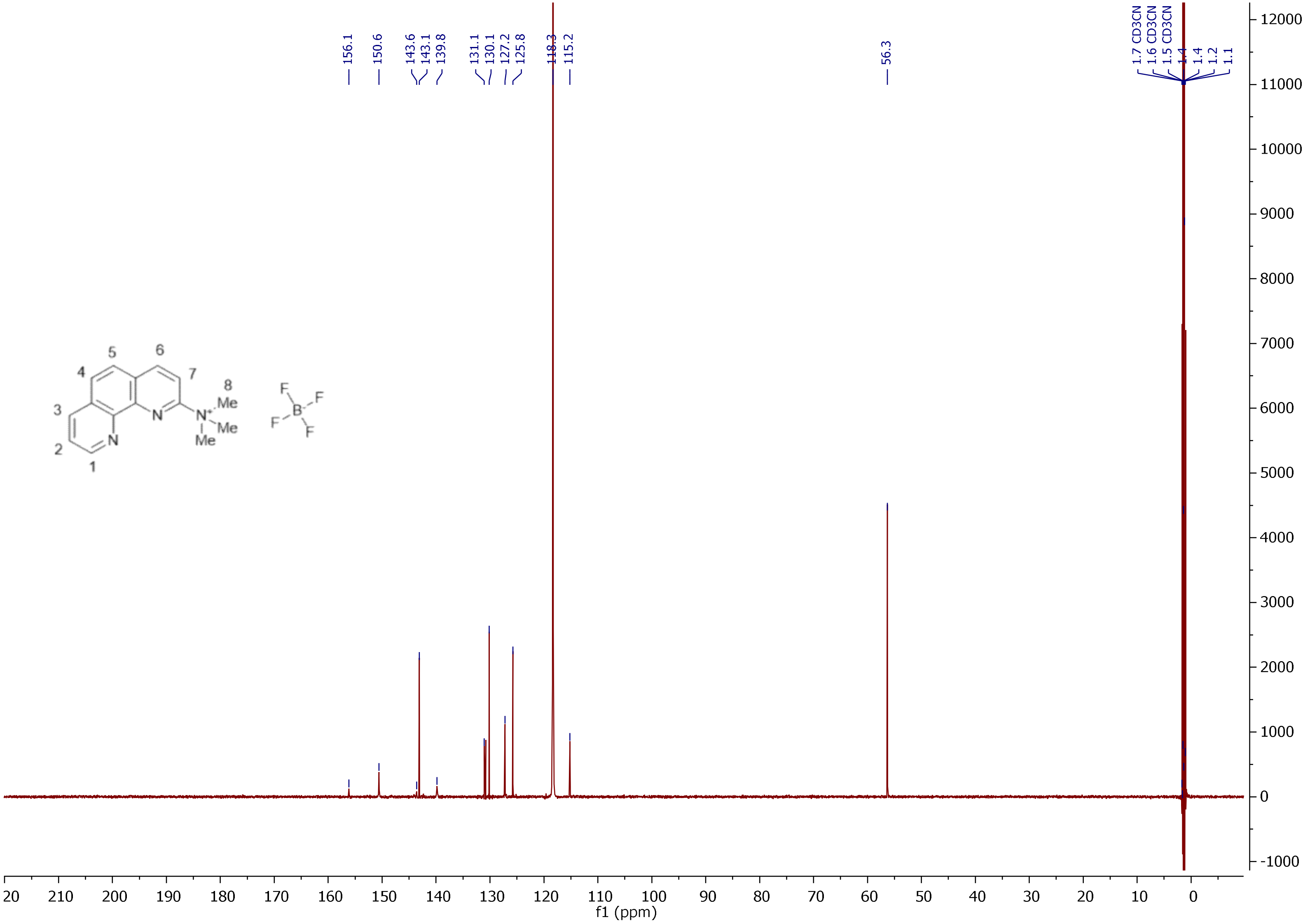

^{19}F NMR (376 MHz, CD_3CN) for *N*,*N*,*N*-trimethyl-1,10-phenanthrolin-2-aminium tetrafluoroborate

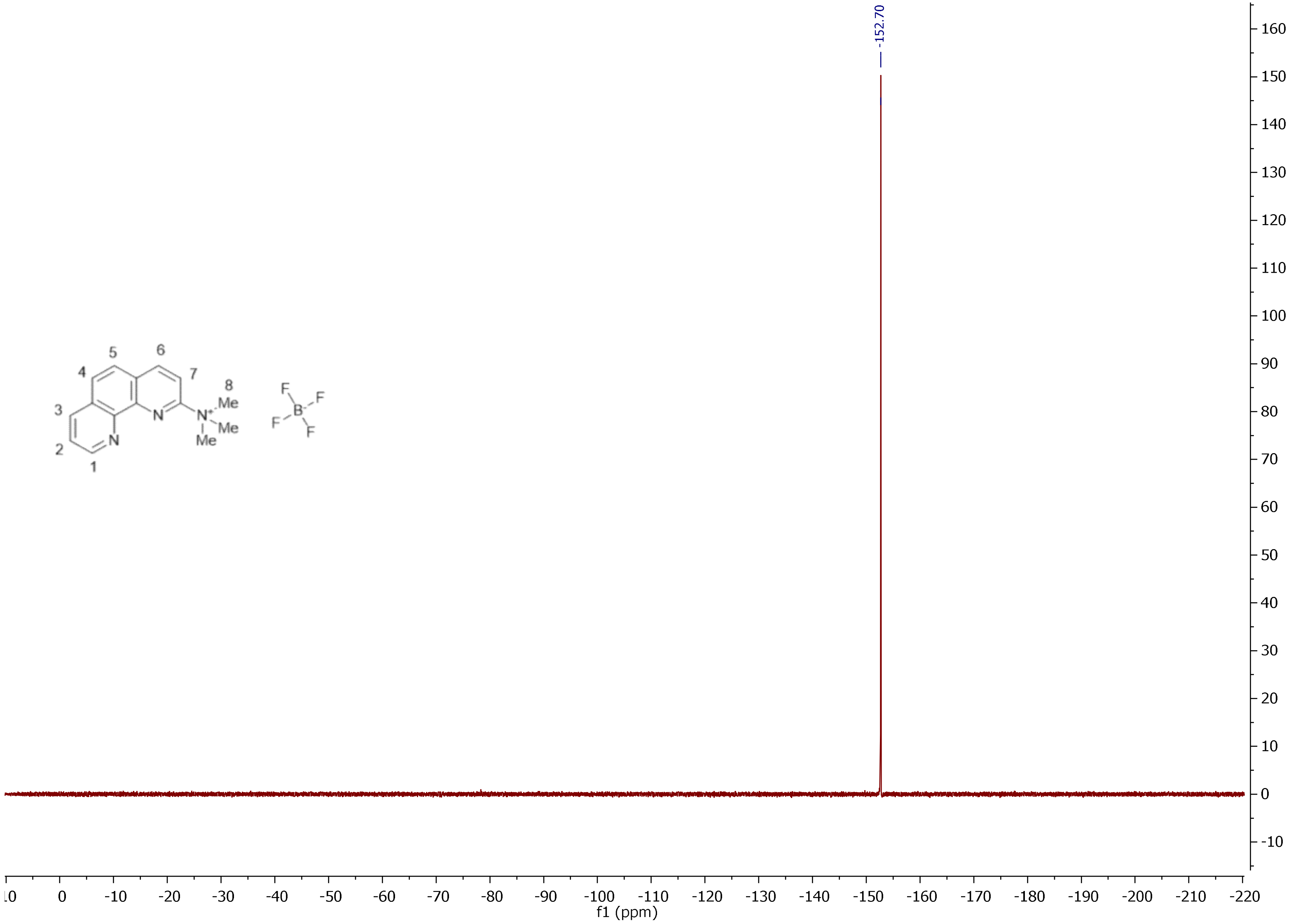

^{11}B NMR (160 MHz, CD_3CN) for *N*,*N*,*N*-trimethyl-1,10-phenanthrolin-2-aminium tetrafluoroborate

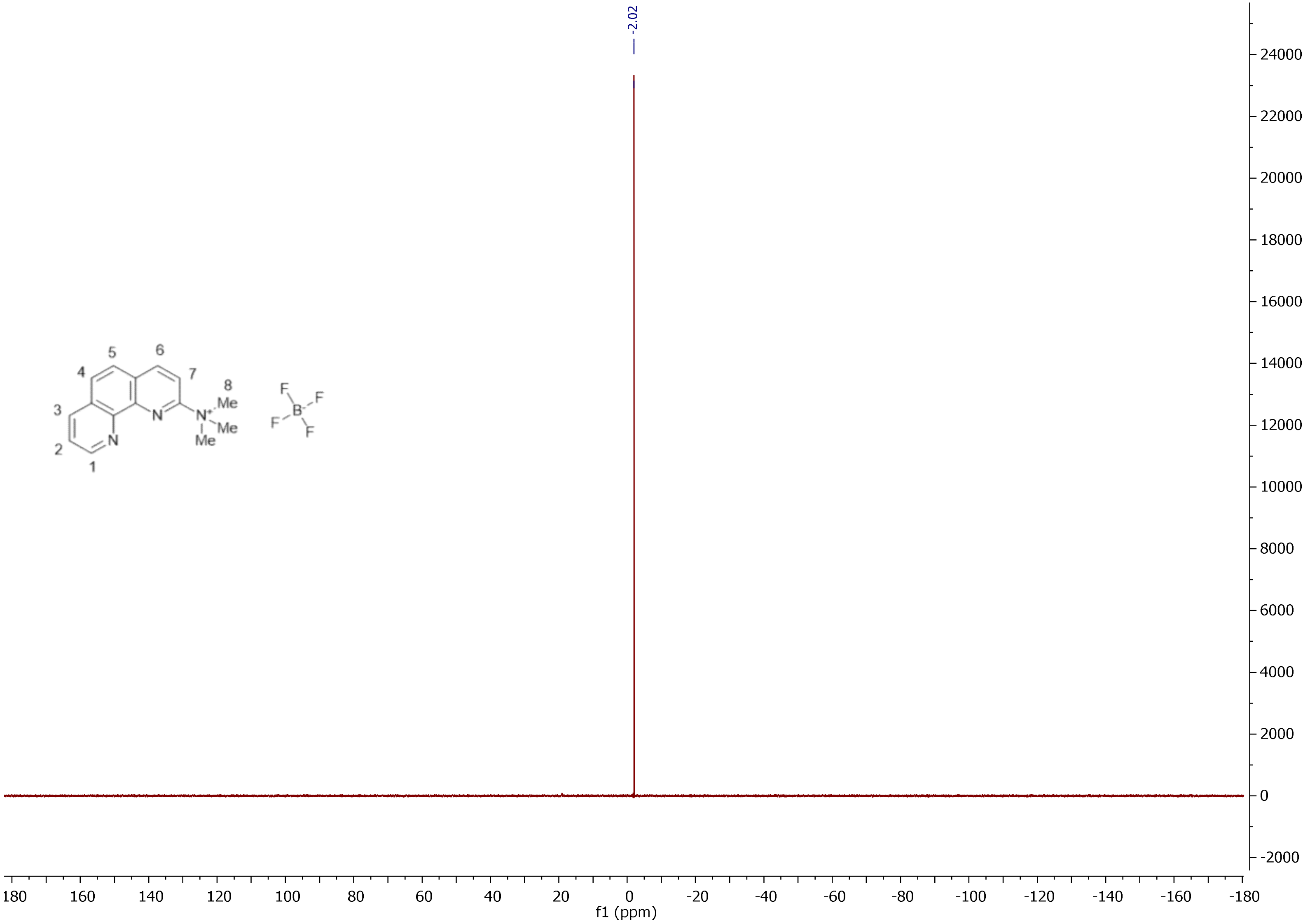

^{1}H NMR (500 MHz, $CDCl_3$) for 2-phenyl-1,10-phenanthroline

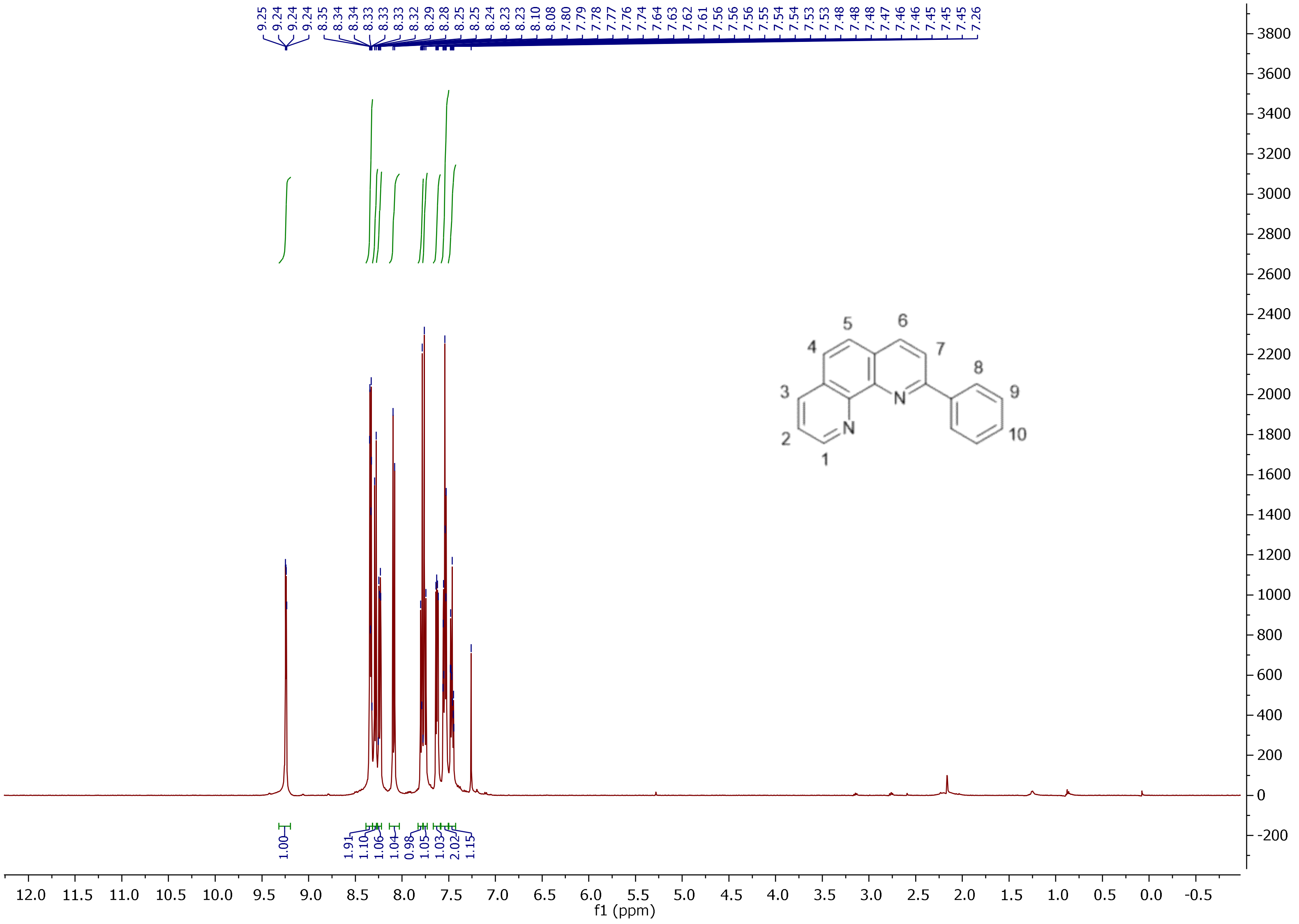

¹³C NMR (126 MHz, $CDCl_3$) for 2-phenyl-1,10-phenanthroline

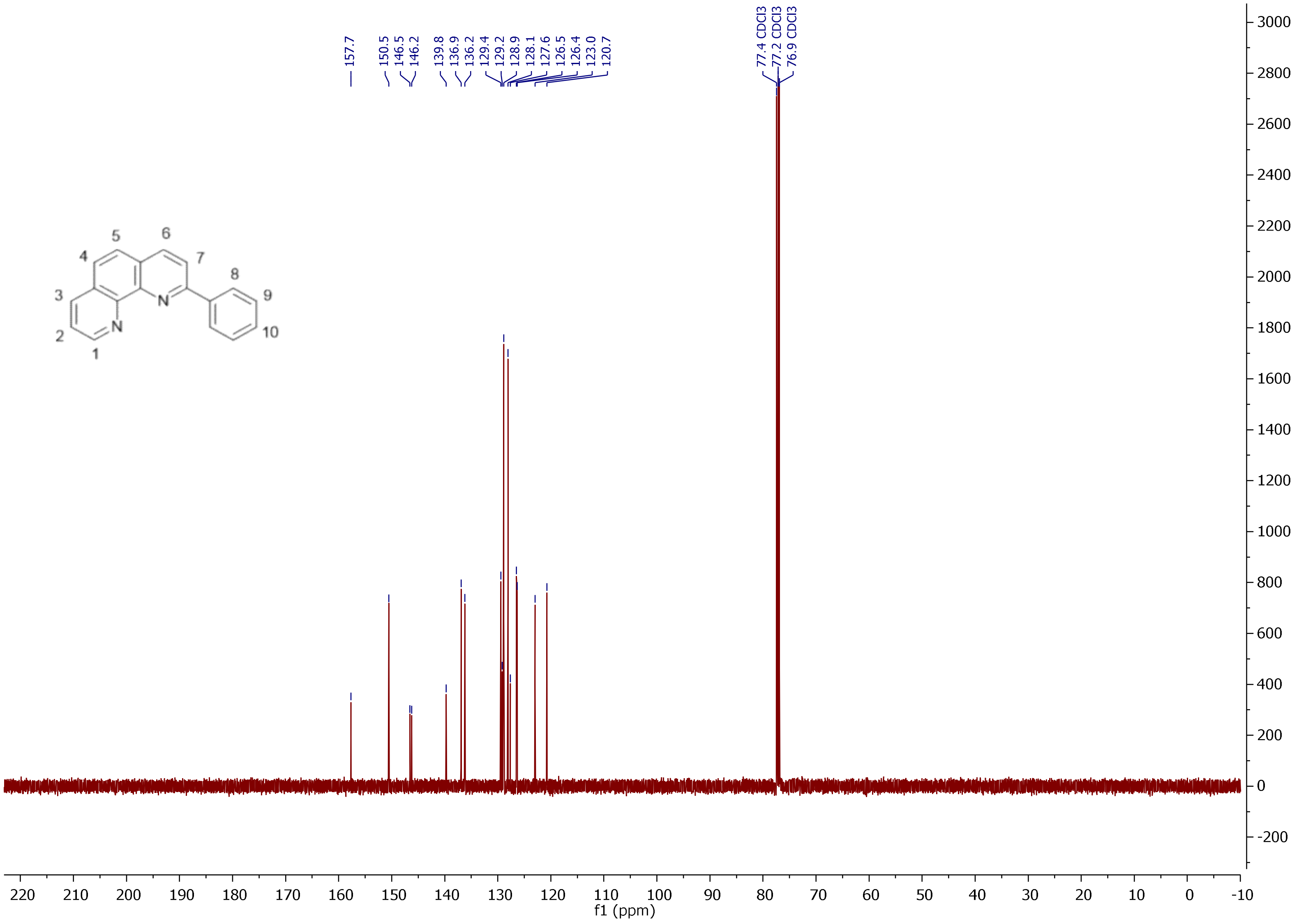

^{1}H NMR (700 MHz, $CDCl_3$) for 2-(4-(trifluoromethyl)phenyl)-1,10-phenanthroline

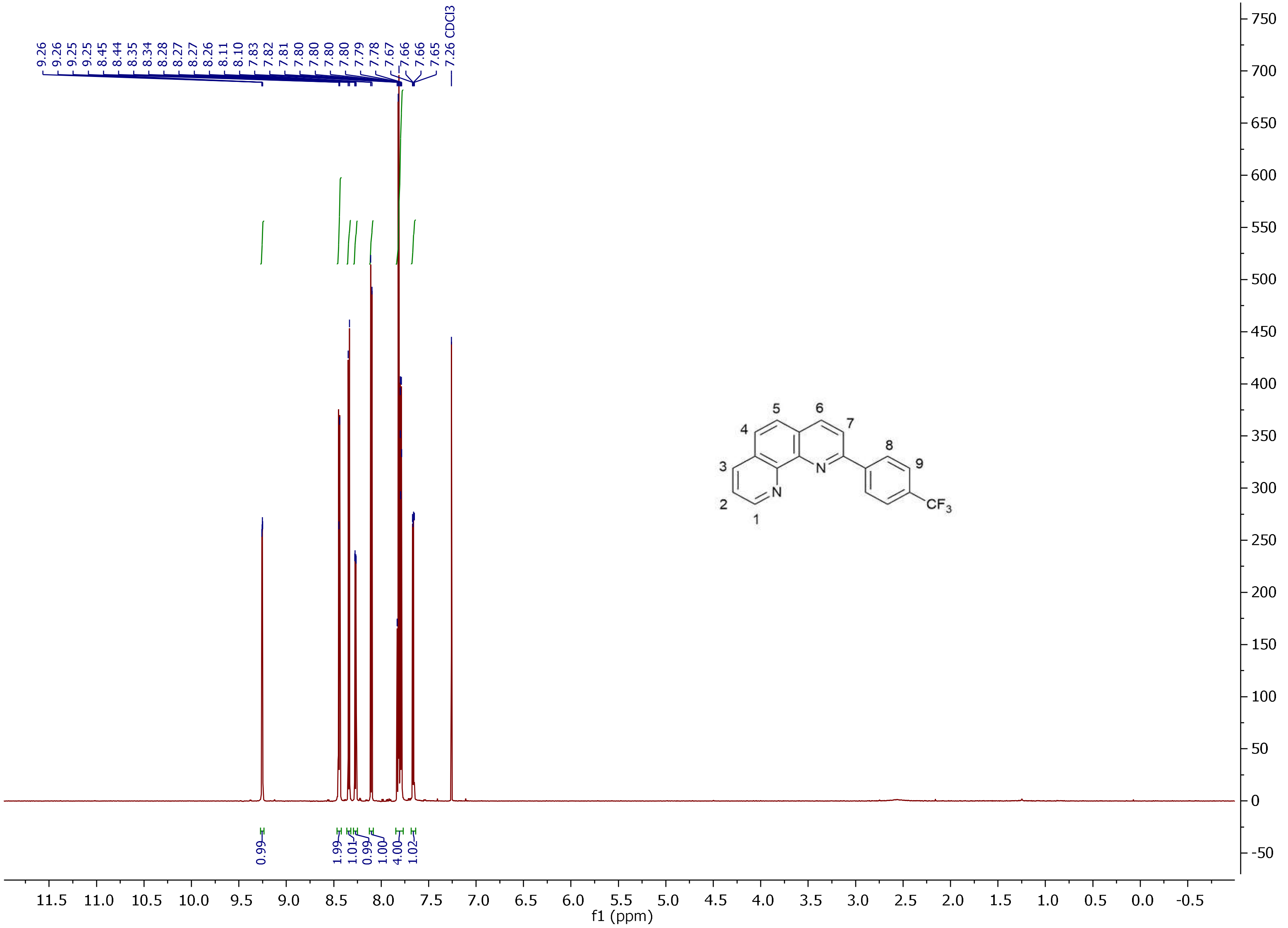

^{13}C NMR (176 MHz, $CDCl_3$) for 2-(4-(trifluoromethyl)phenyl)-1,10-phenanthroline

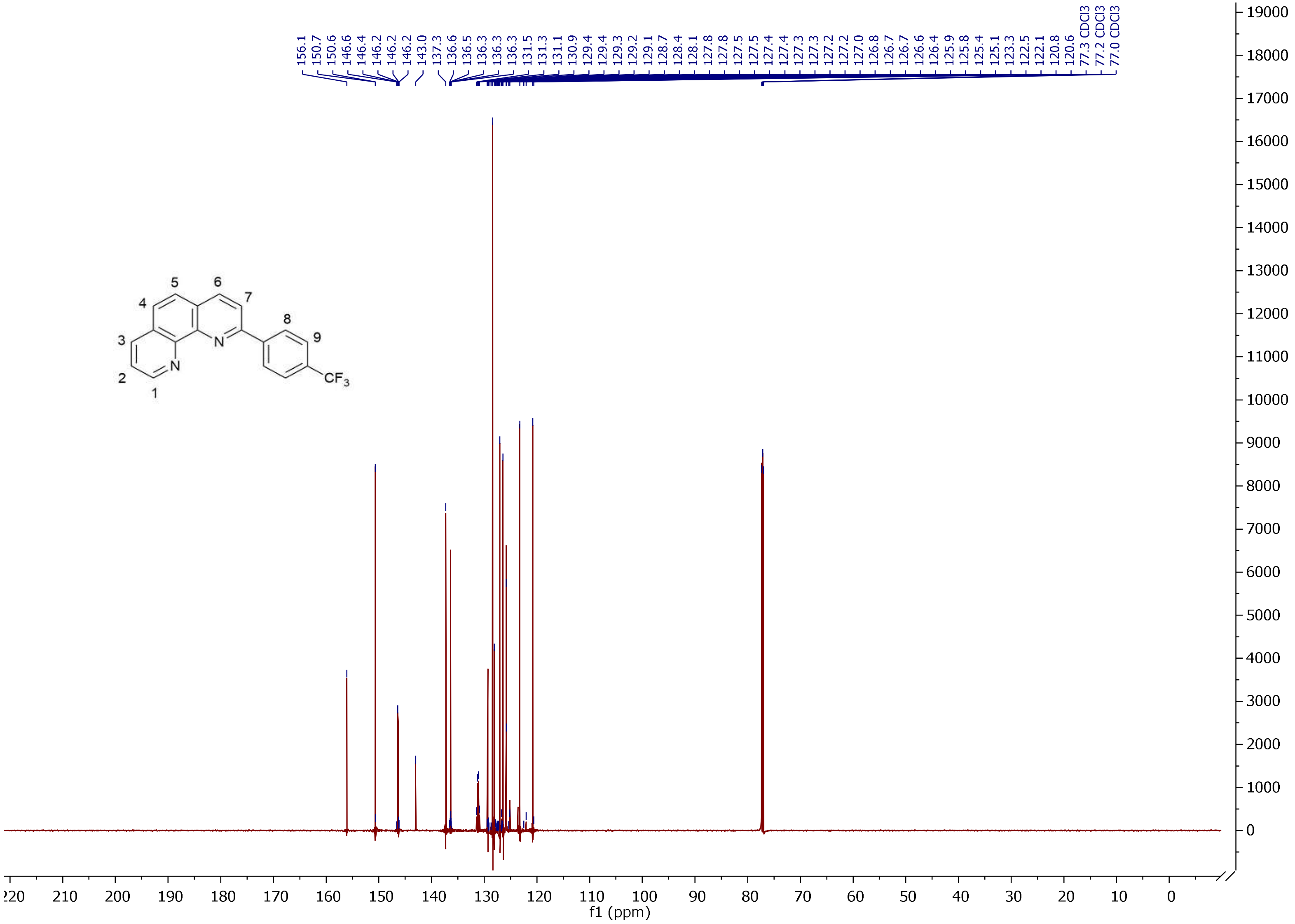

^{19}F NMR (471 MHz, $CDCl_3$) for 2-(4-(trifluoromethyl)phenyl)-1,10-phenanthroline

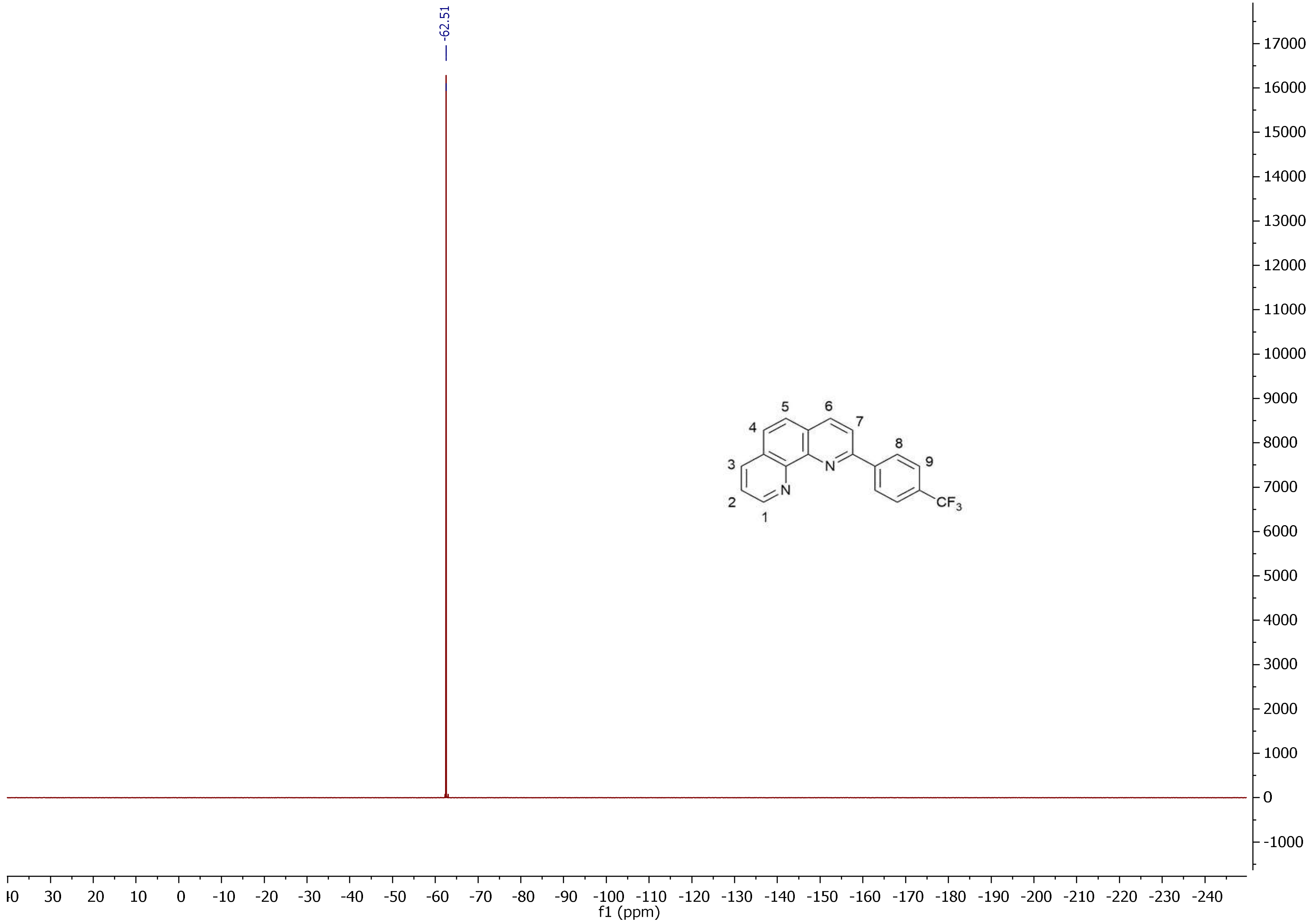

^{1}H NMR (700 MHz, $CDCl_3$) for 2-(2-(trifluoromethyl)phenyl)-1,10-phenanthroline

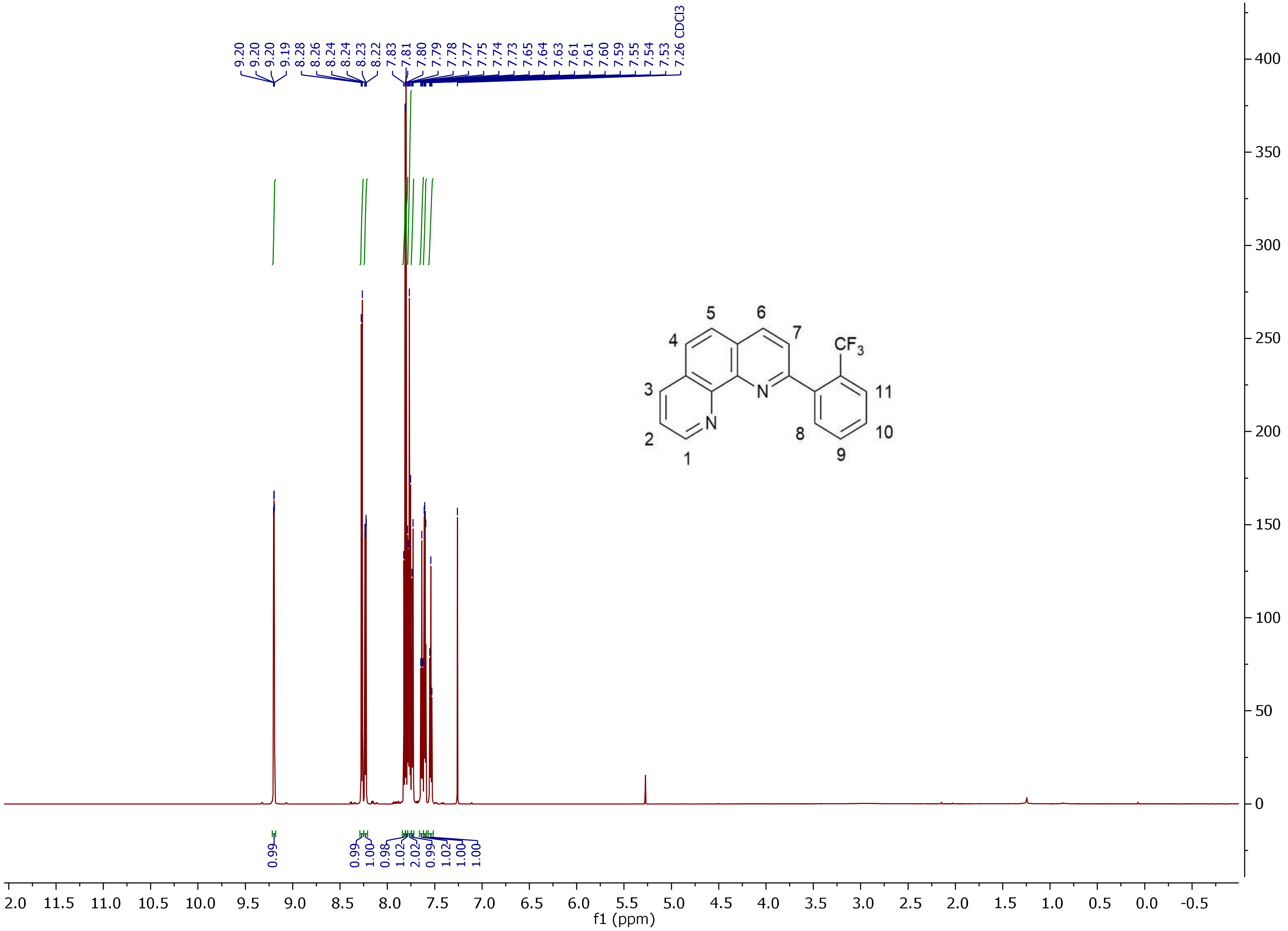

^{13}C NMR (176 MHz, $CDCl_3$) for 2-(2-(trifluoromethyl)phenyl)-1,10-phenanthroline

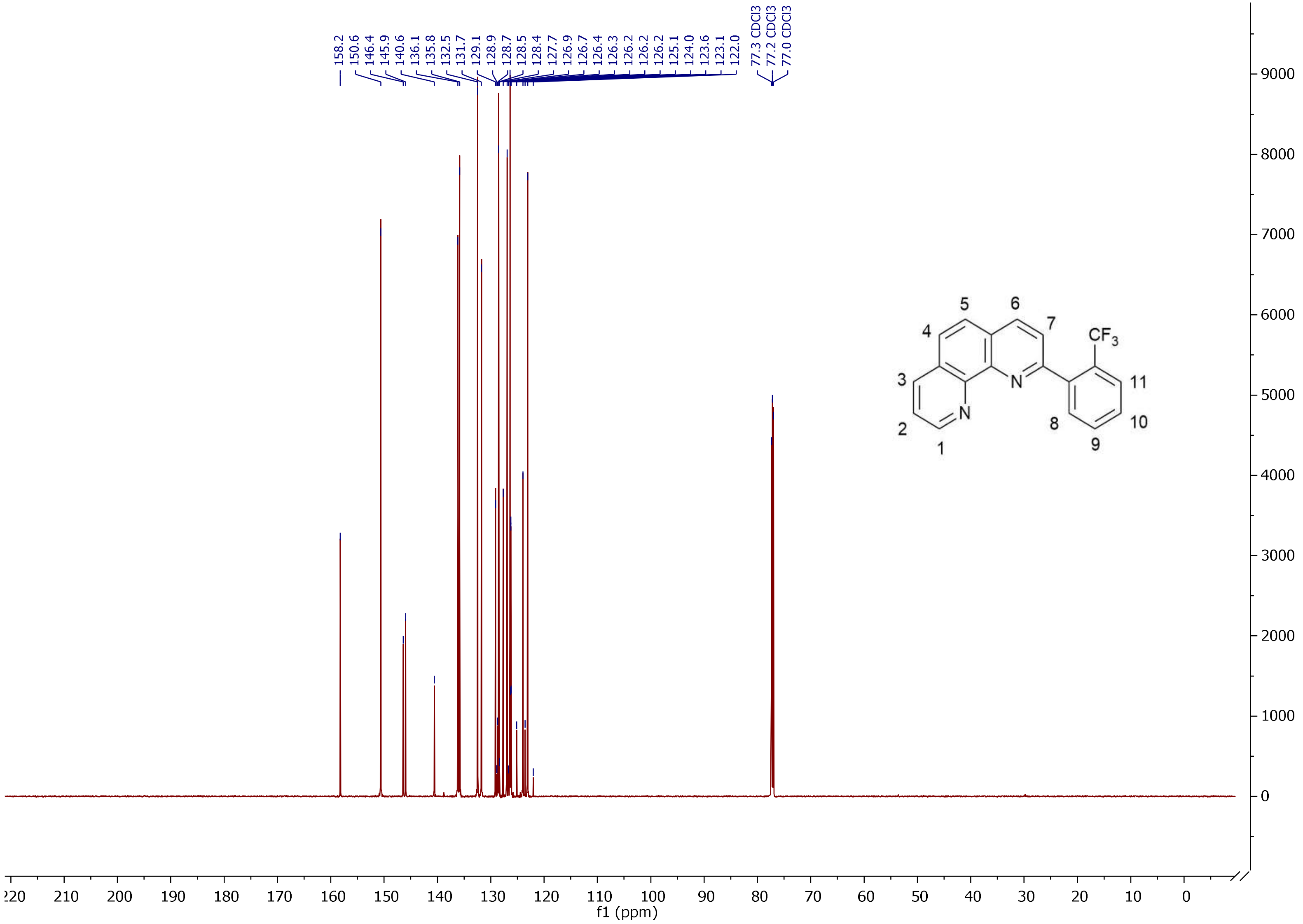

^{19}F NMR (376 MHz, $CDCl_3$) for 2-(2-(trifluoromethyl)phenyl)-1,10-phenanthroline

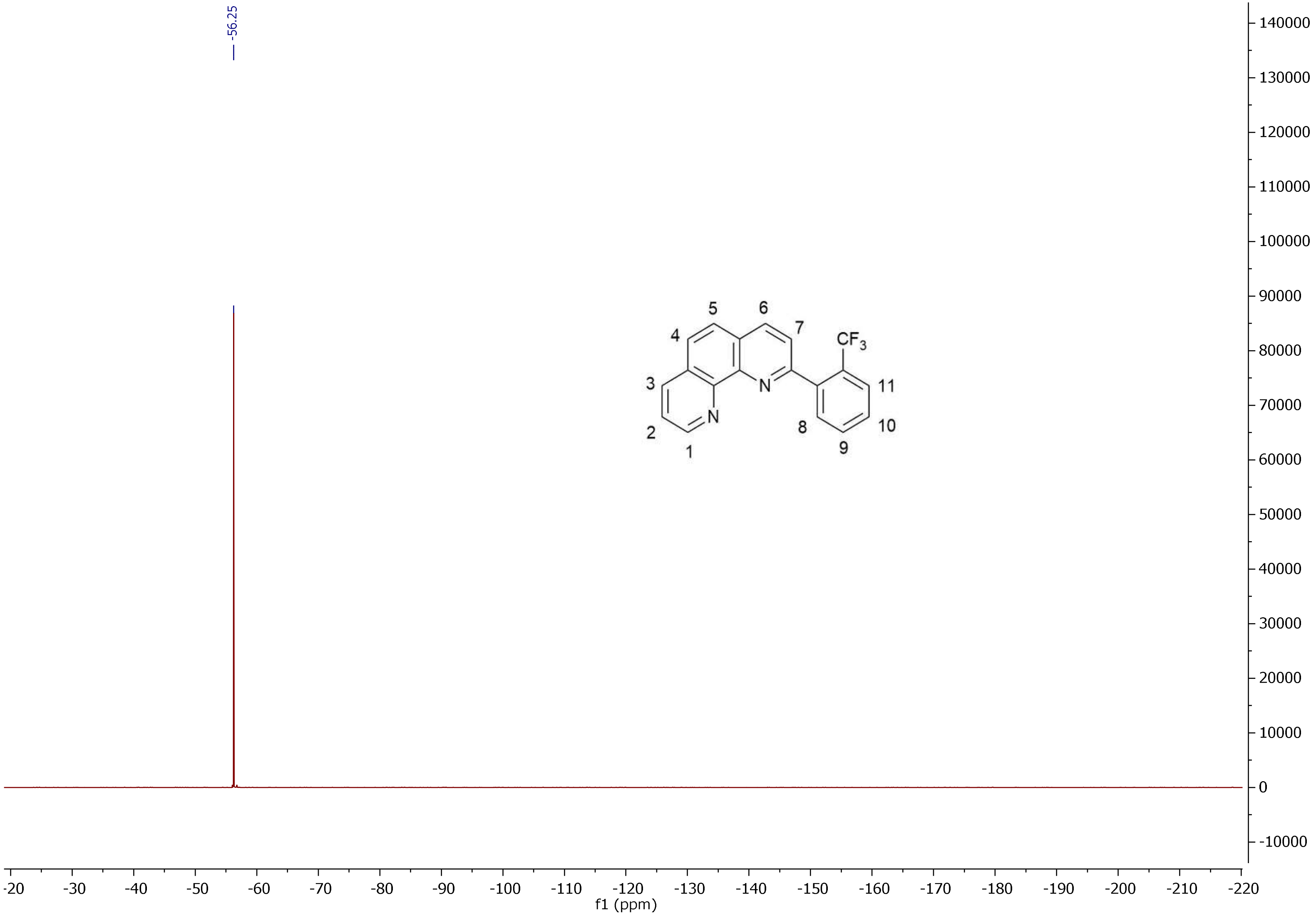

^{1}H NMR (400 MHz, $CDCl_3$) for 2-(3,5-bis(trifluoromethyl)phenyl)-1,10-phenanthroline

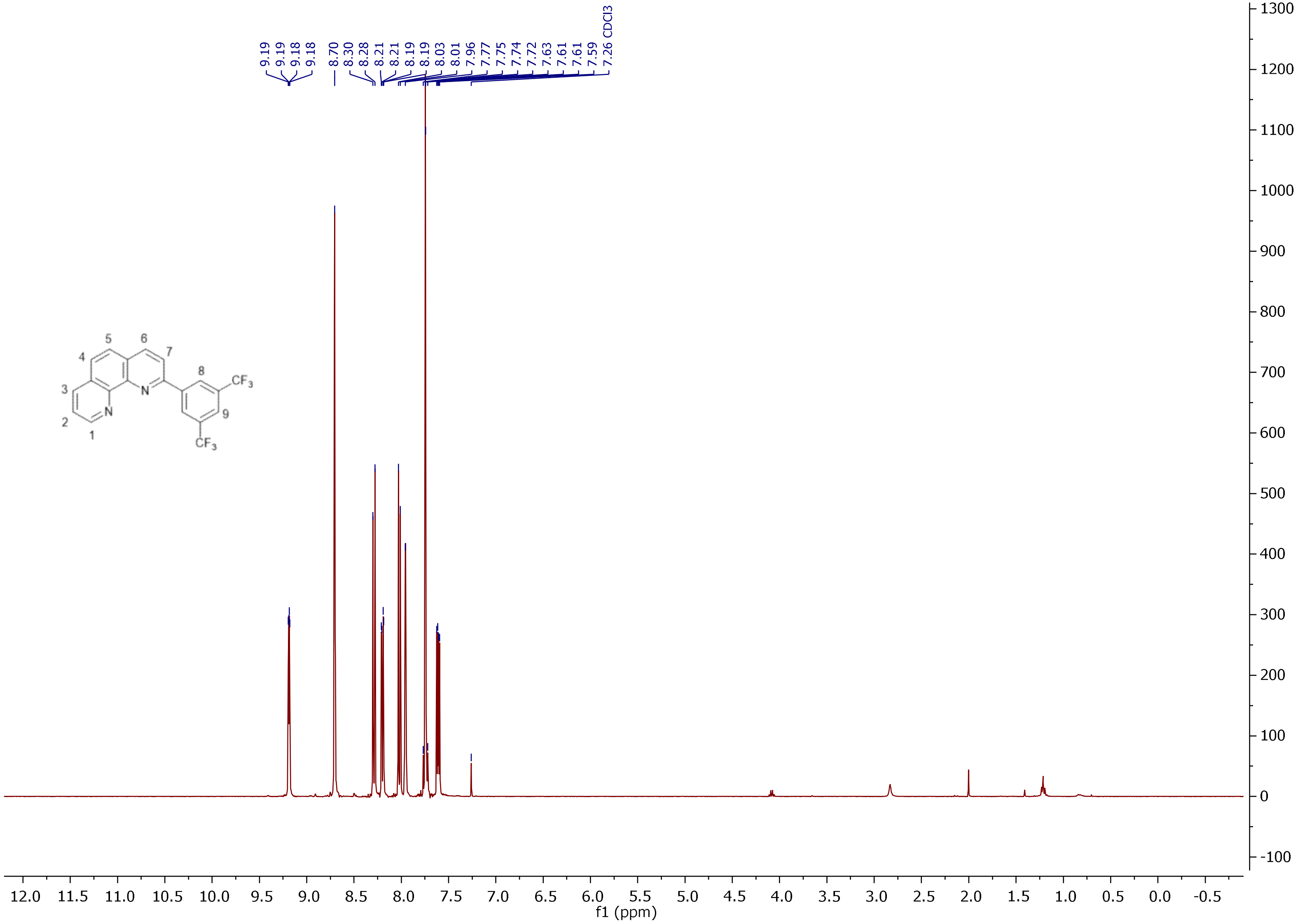

^{13}C NMR (101 MHz, $CDCl_3$) for 2-(3,5-bis(trifluoromethyl)phenyl)-1,10-phenanthroline

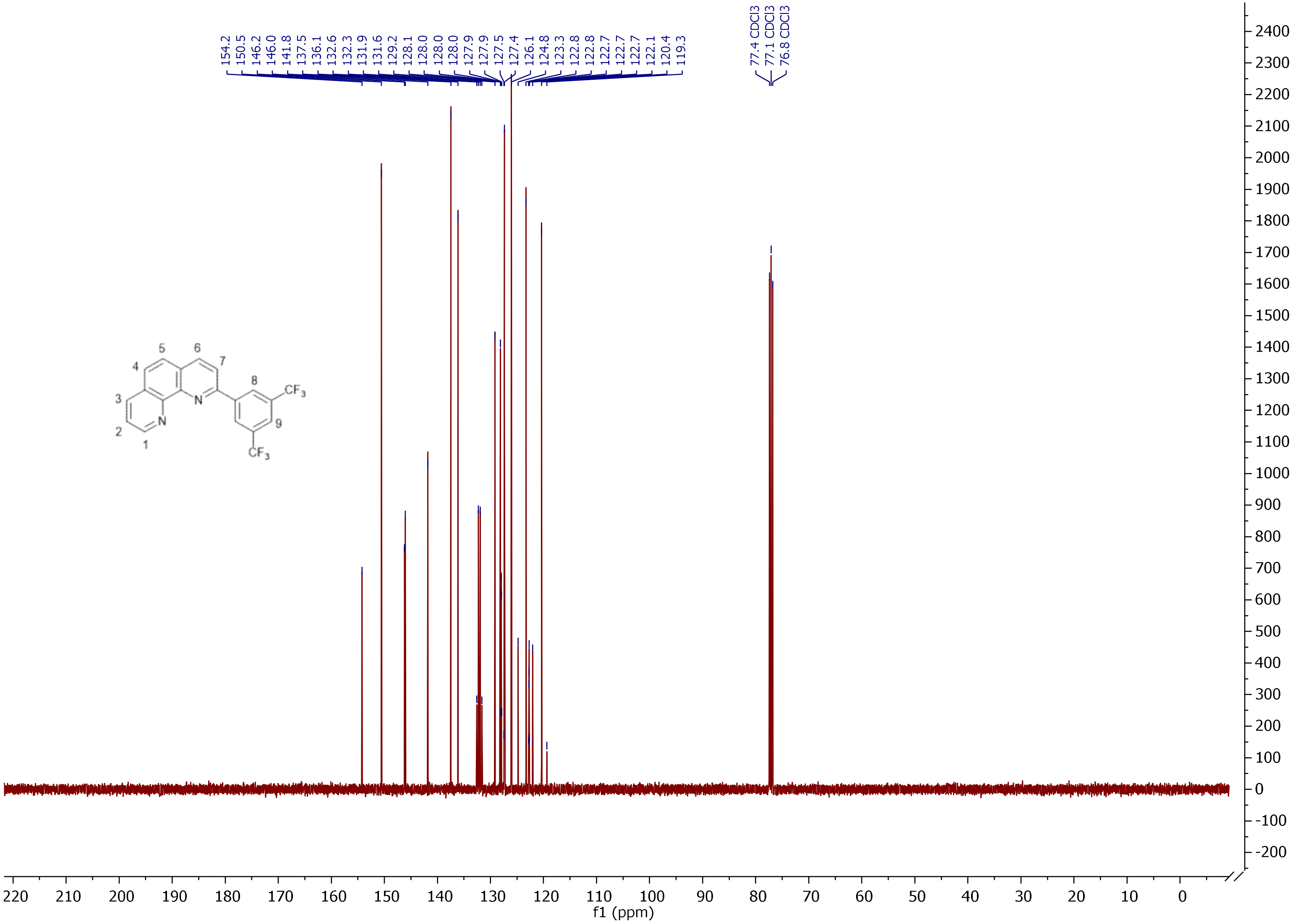

^{19}F NMR (376 MHz, $CDCl_3$) for 2-(3,5-bis(trifluoromethyl)phenyl)-1,10-phenanthroline

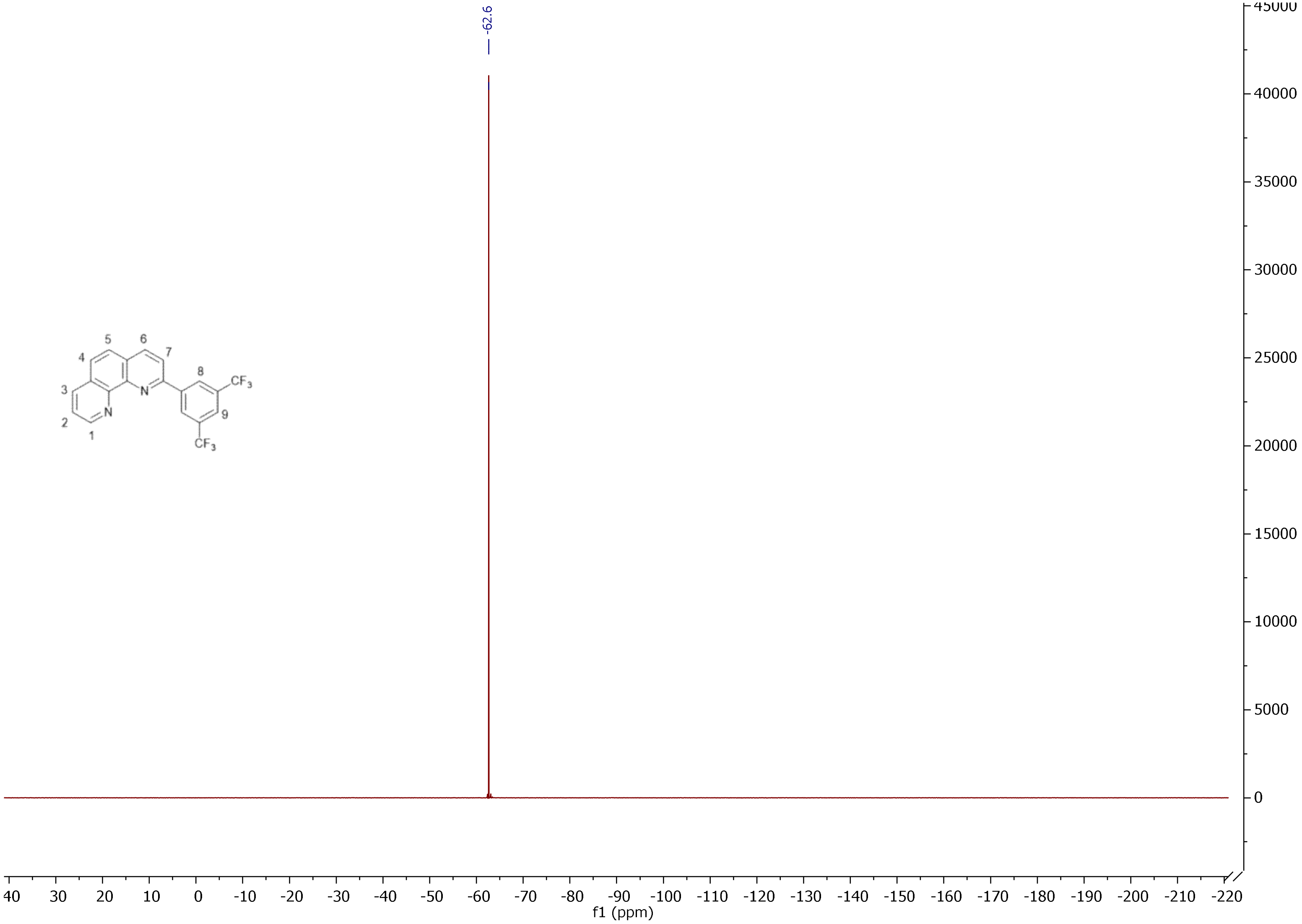

^{1}H NMR (500 MHz, $CDCl_3$) for 2-(3,4,5-trifluorophenyl)-1,10-phenanthroline

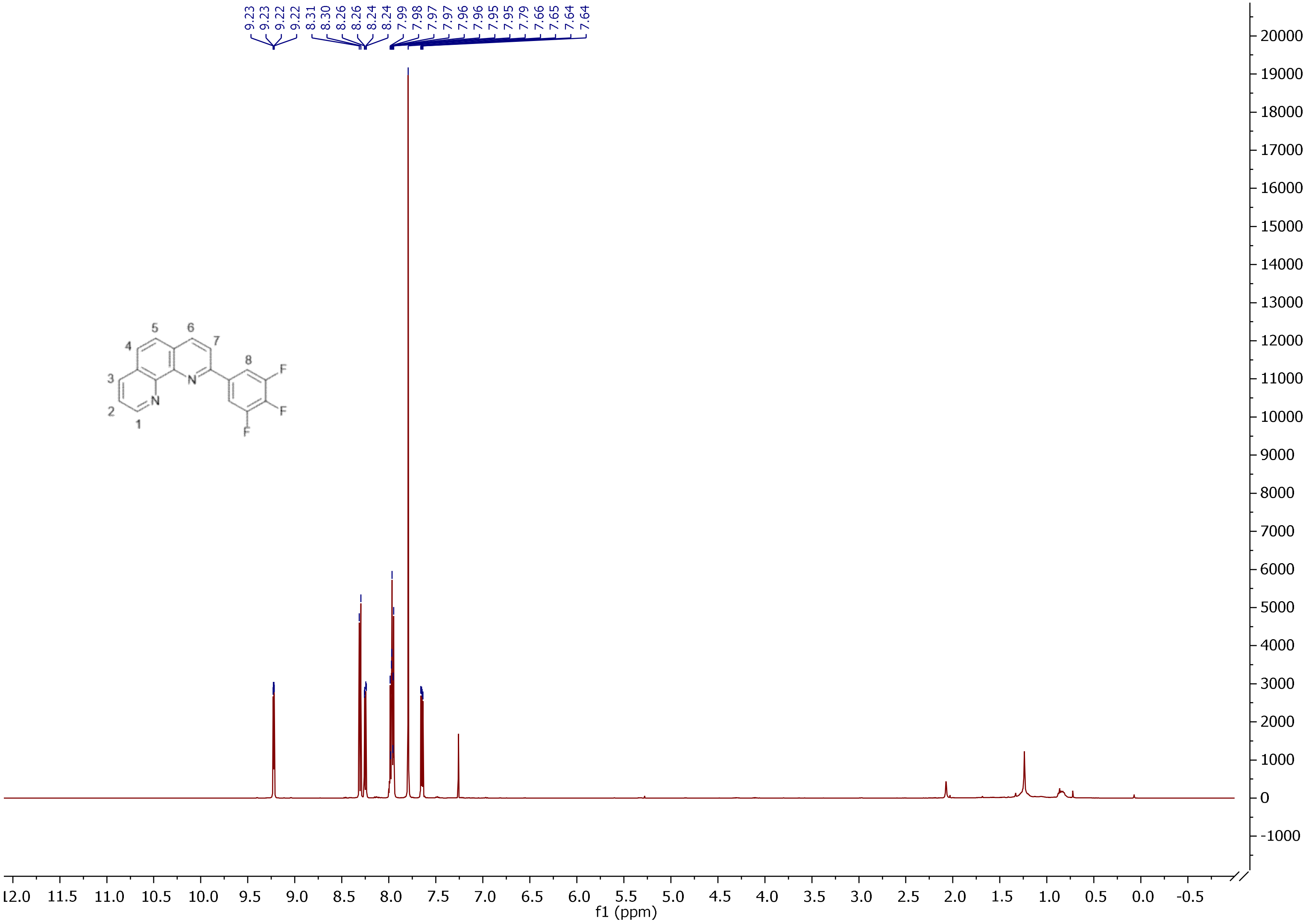

^{13}C NMR (126 MHz, $CDCl_3$) for 2-(3,4,5-trifluorophenyl)-1,10-phenanthroline

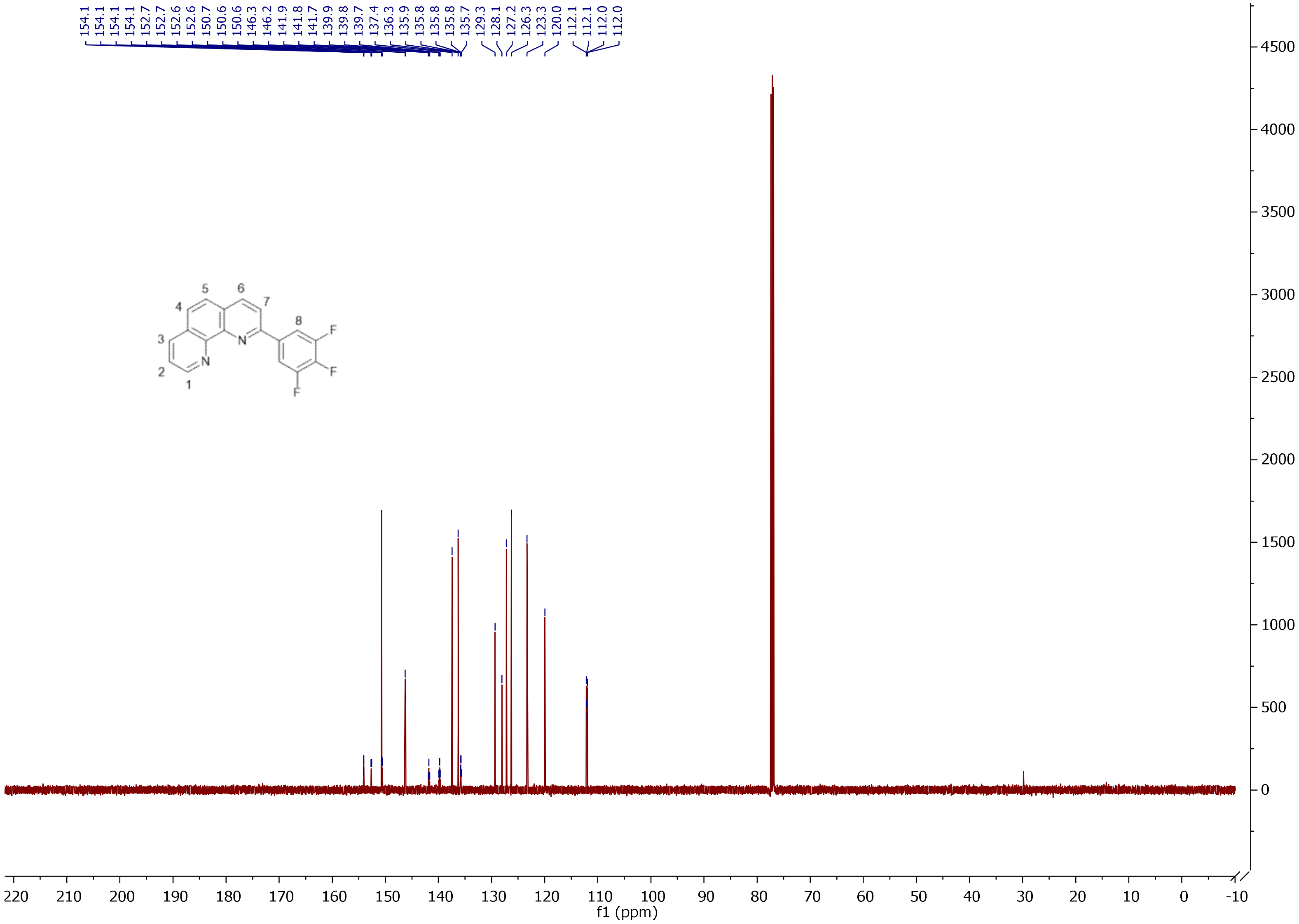

^{19}F NMR (476 MHz, $CDCl_3$) for 2-(3,4,5-trifluorophenyl)-1,10-phenanthroline

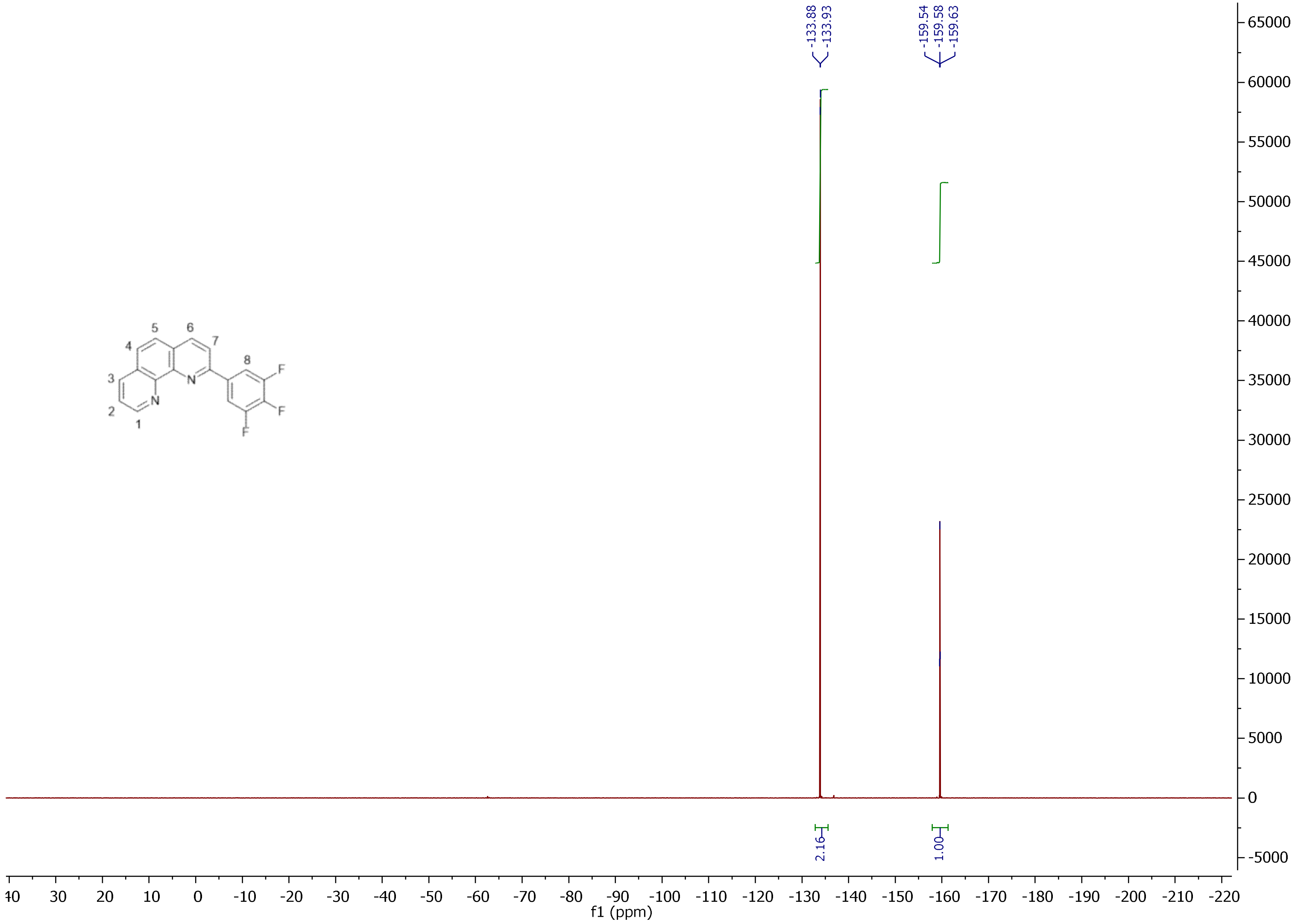

^{1}**H NMR** (500 MHz, $CDCl_3$) for 2-mesityl-1,10-phenanthroline

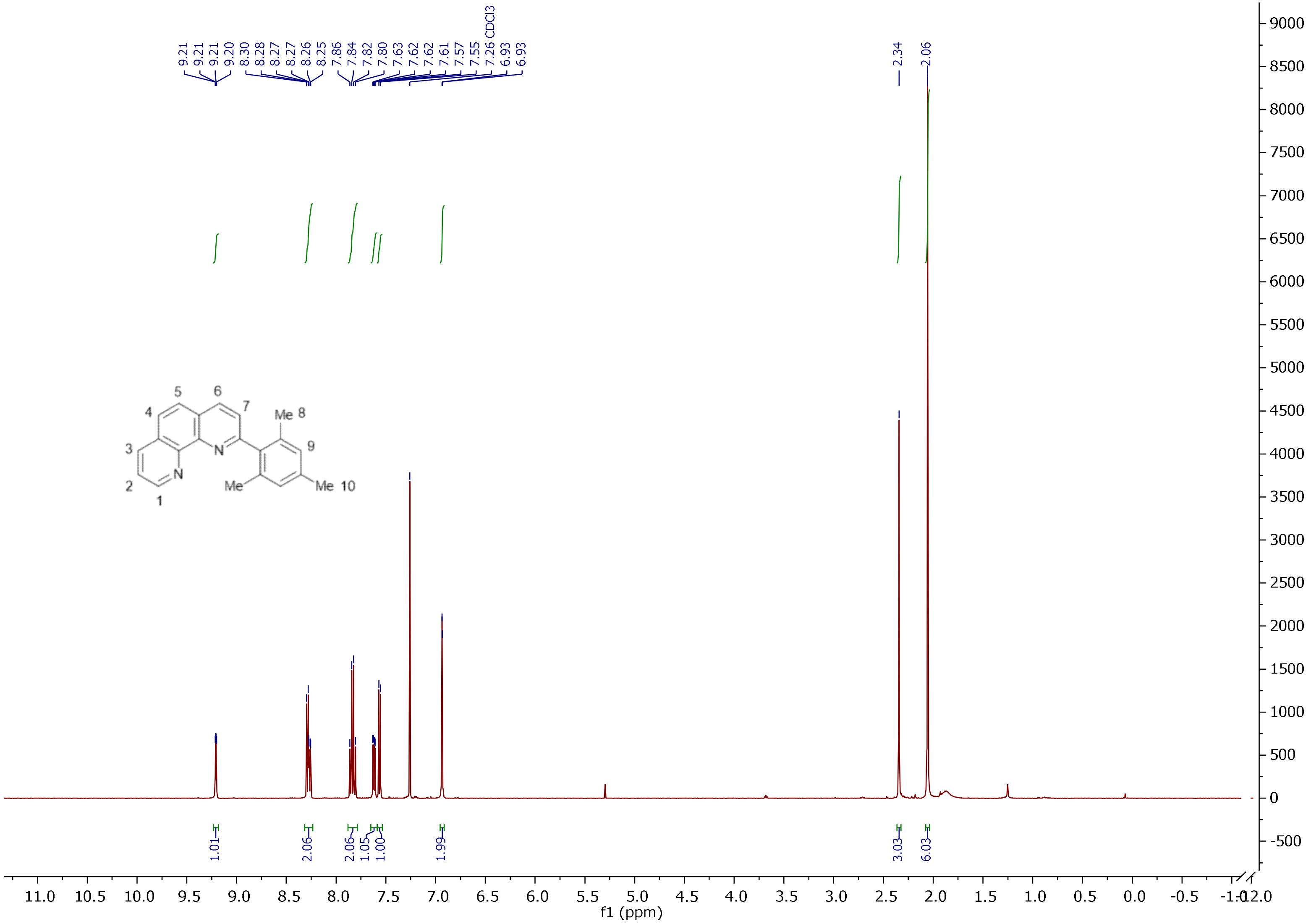

^{13}C NMR (126 MHz, $CDCl_3$) for 2-mesityl-1,10-phenanthroline

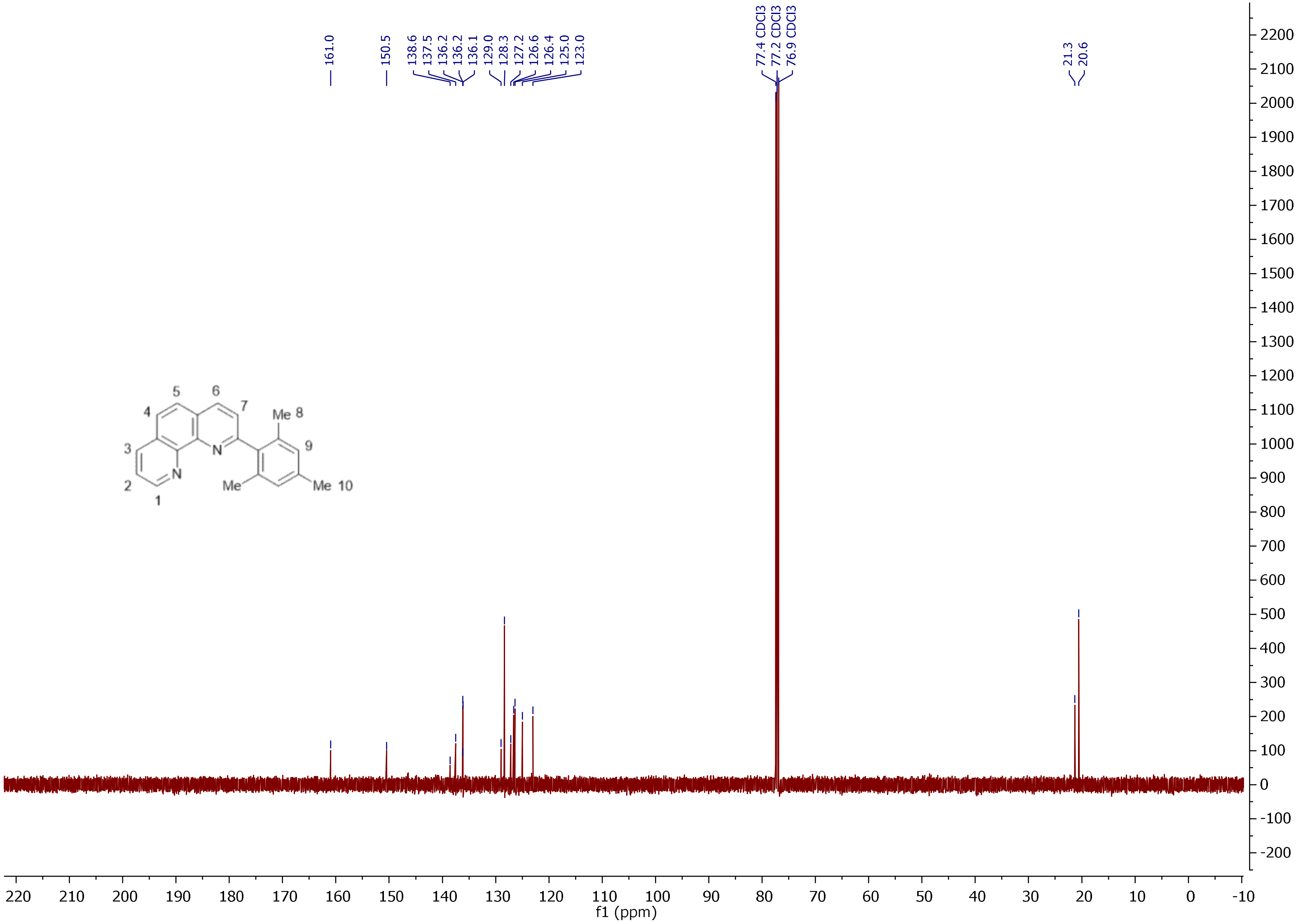

^{1}H NMR (400 MHz, $CDCl_3$) for 2-(3,5-di-*tert*-butylphenyl)-1,10-phenanthroline

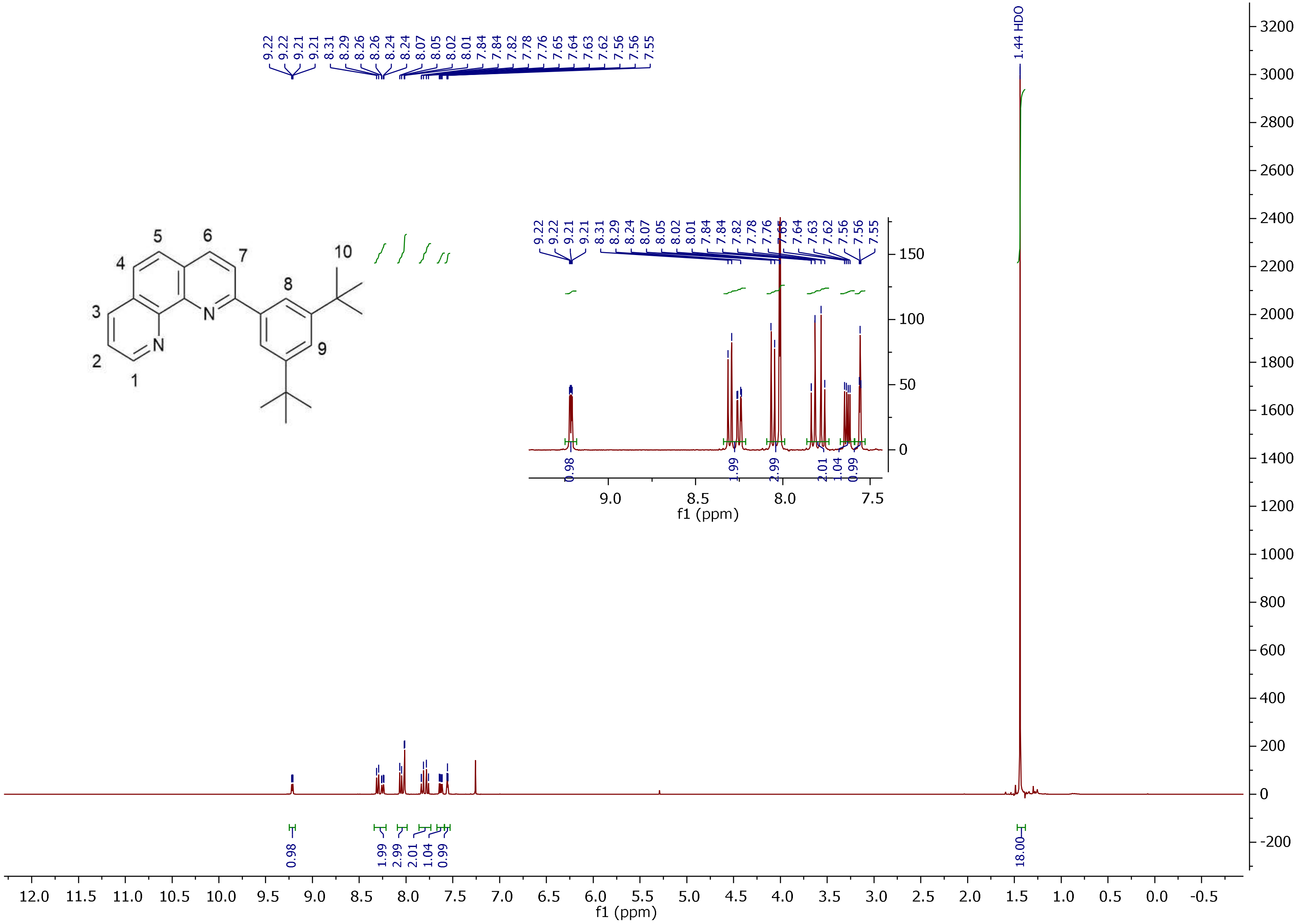

^{13}C NMR (101 MHz, $CDCl_3$) for 2-(3,5-di-*tert*-butylphenyl)-1,10-phenanthroline

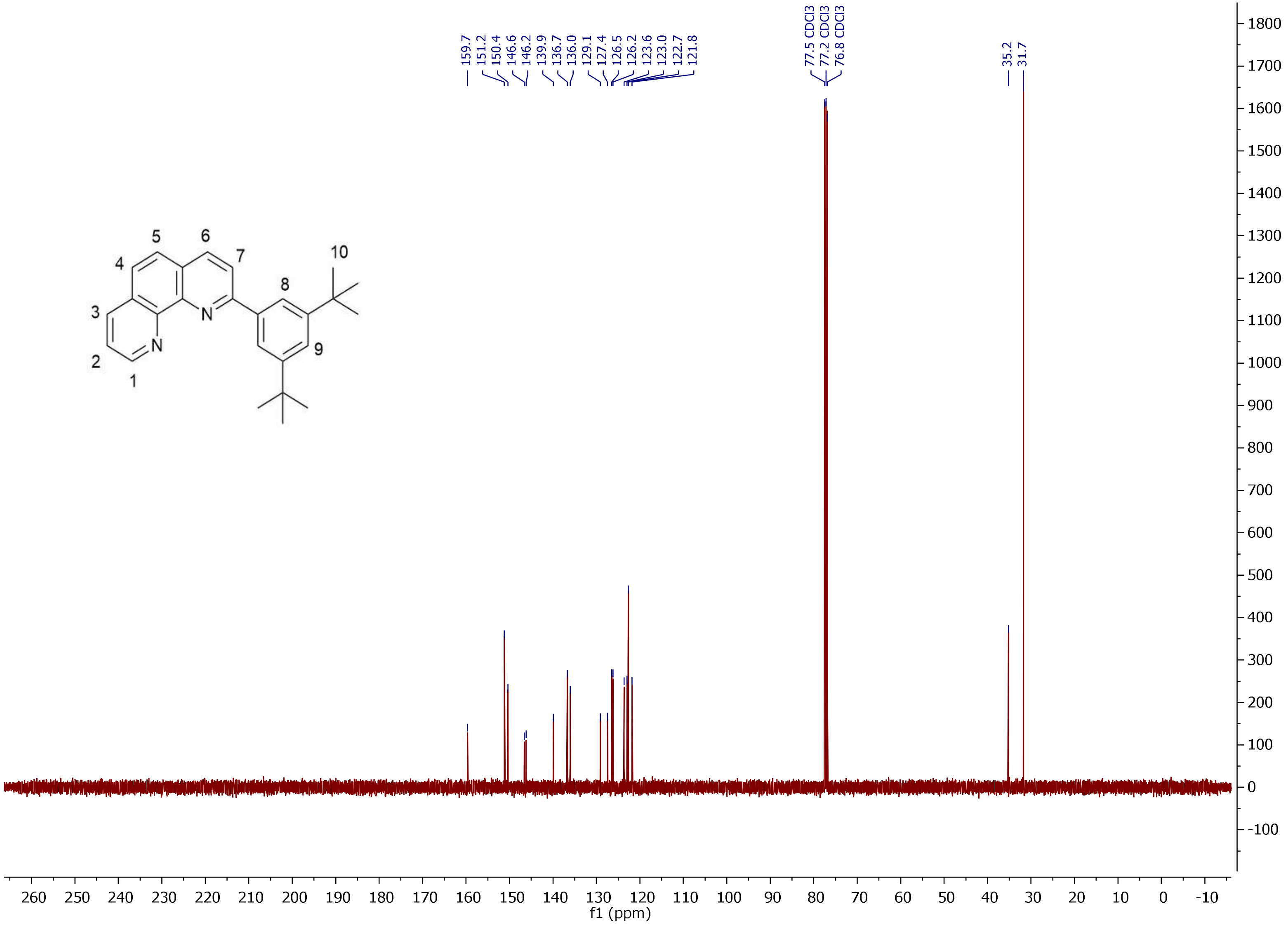

^{1}H NMR (500 MHz, $CDCl_3$) for 2-(4-methoxyphenyl)-1,10-phenanthroline

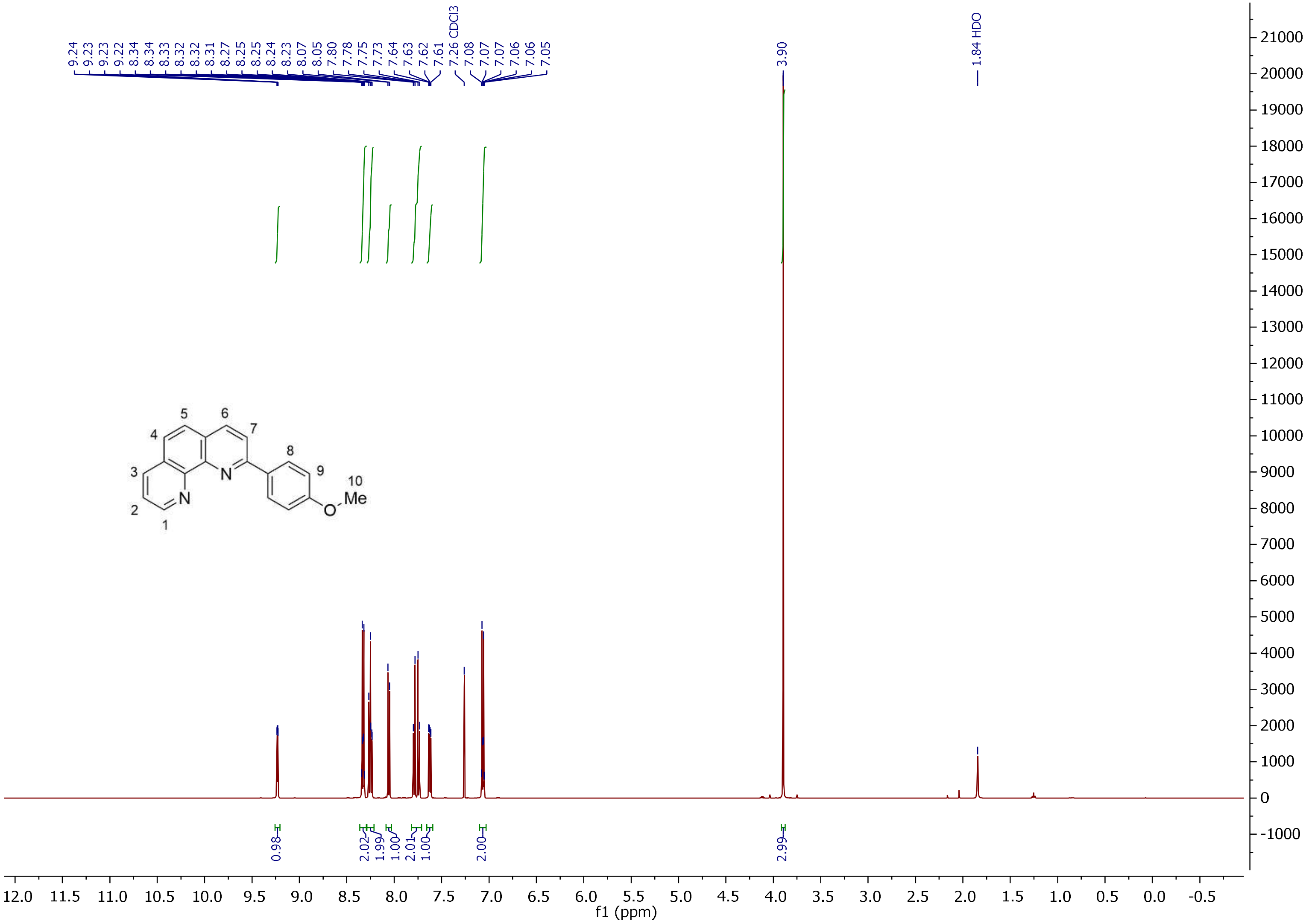

^{13}C NMR (126 MHz, $CDCl_3$) for 2-(4-methoxyphenyl)-1,10-phenanthroline

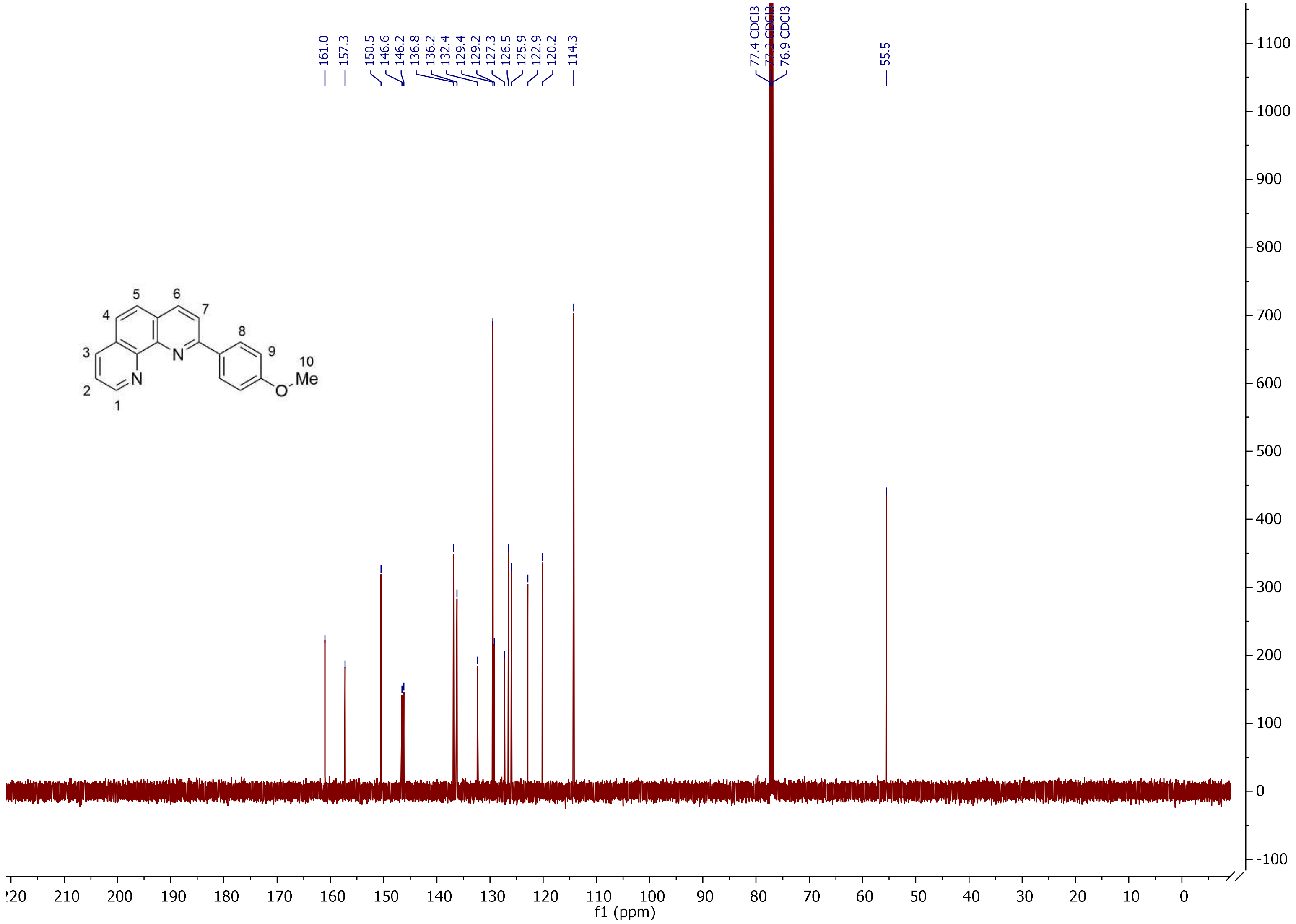

^{1}H NMR (700 MHz, $CDCl_3$) for 2,2,2-trifluoro-*N*-phenylacetamide **(1c)**

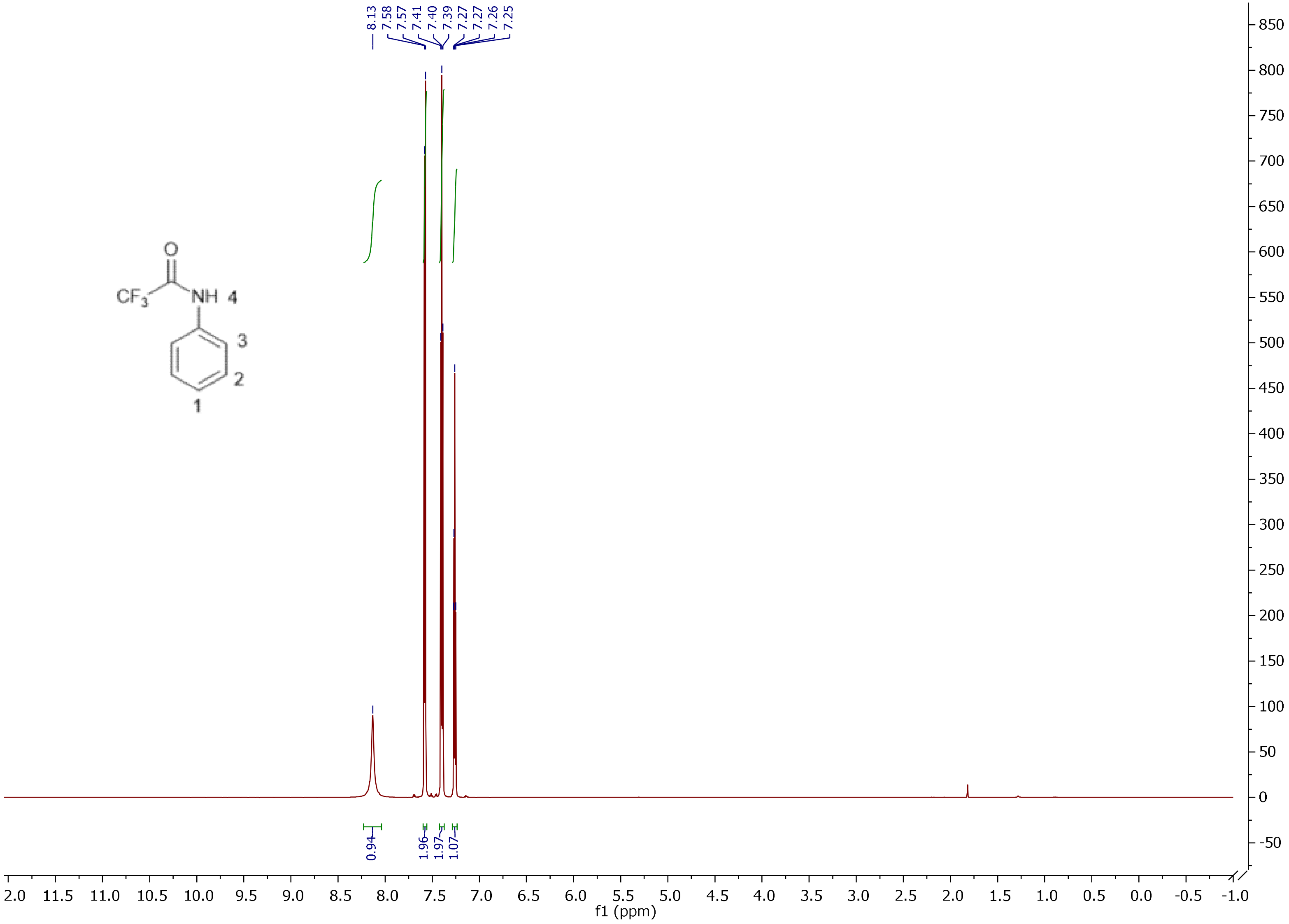

^{13}**C NMR** (176 MHz, $CDCl_3$) for 2,2,2-trifluoro-*N*-phenylacetamide **(1c)**

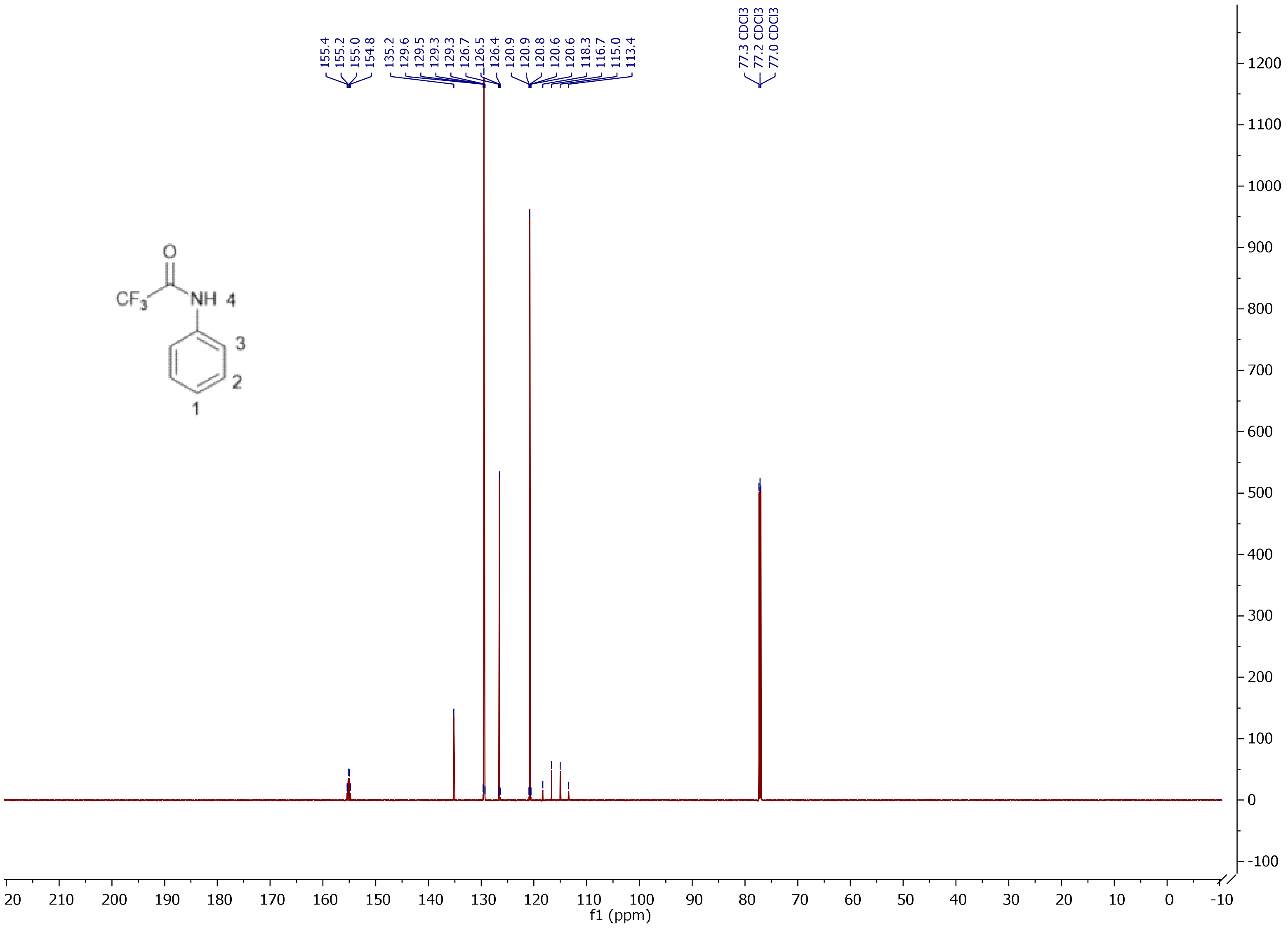

^{19}F NMR (376 MHz, $CDCl_3$) for 2,2,2-trifluoro-*N*-phenylacetamide **(1c)**

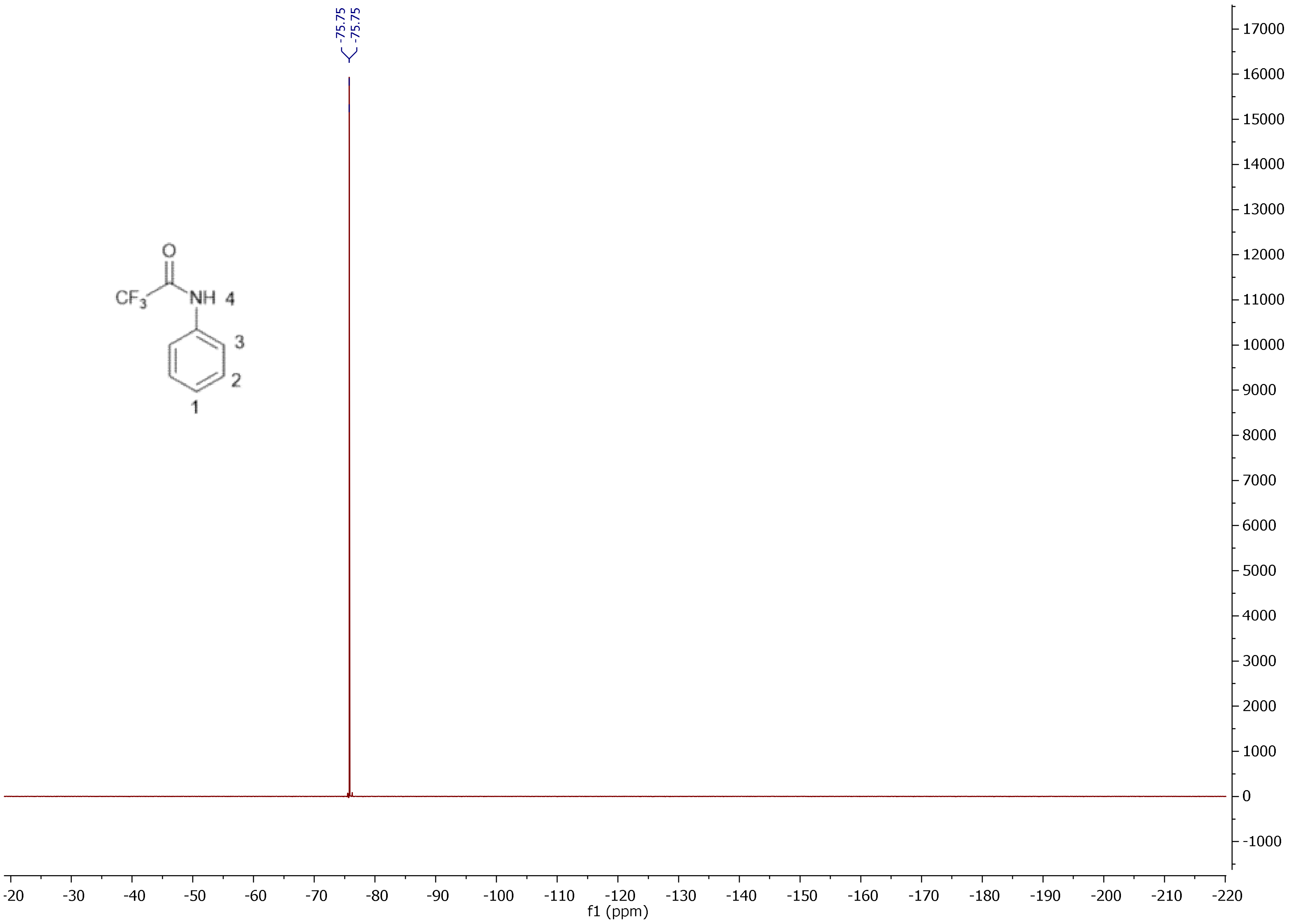

^{1}H NMR (400 MHz, $CDCl_3$) for 2,2,3,3,3-pentafluoro-*N*-phenylpropanamide **(1d)**

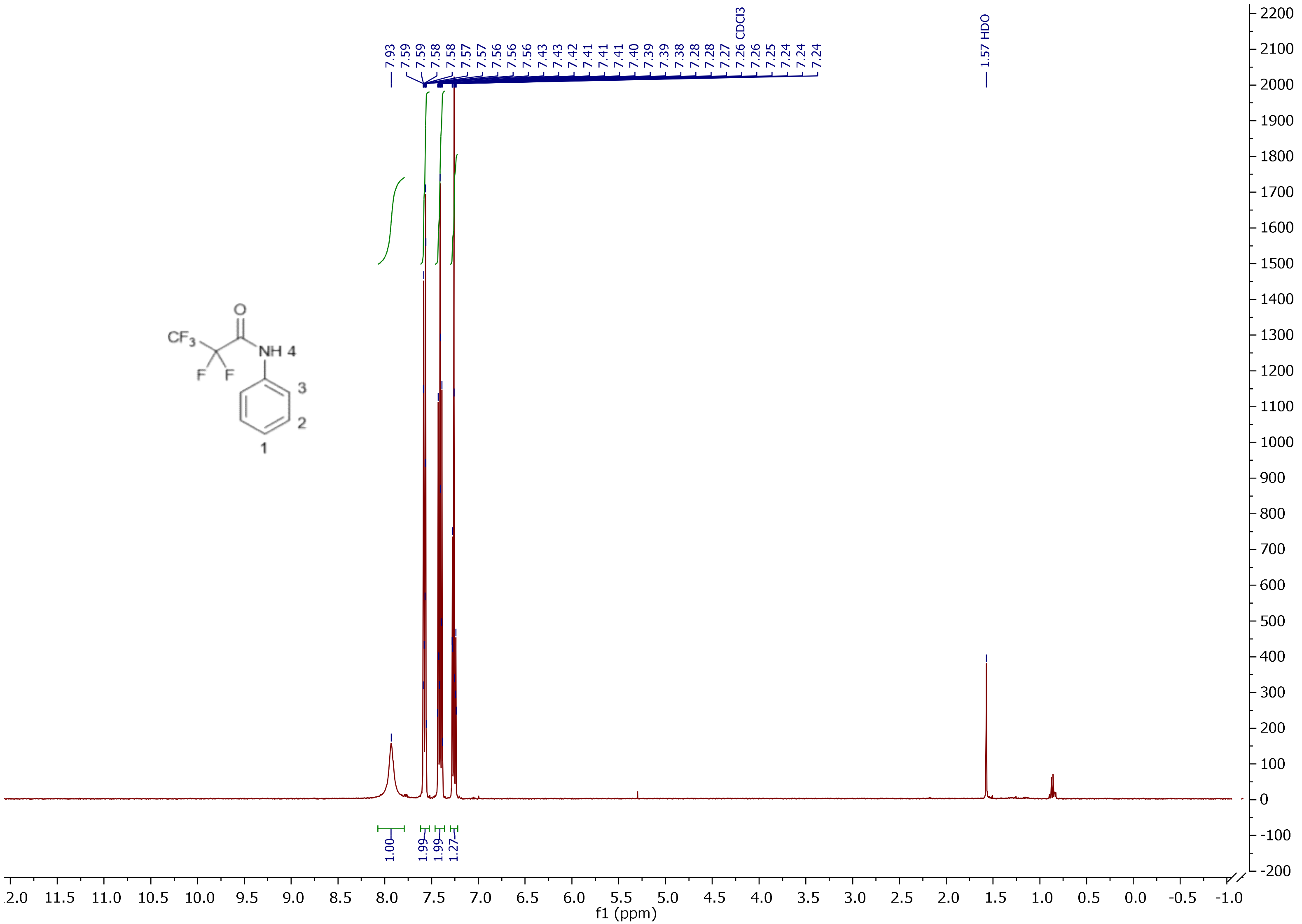

^{13}C NMR (176 MHz, $CDCl_3$) for 2,2,3,3,3-pentafluoro-*N*-phenylpropanamide **(1d)**

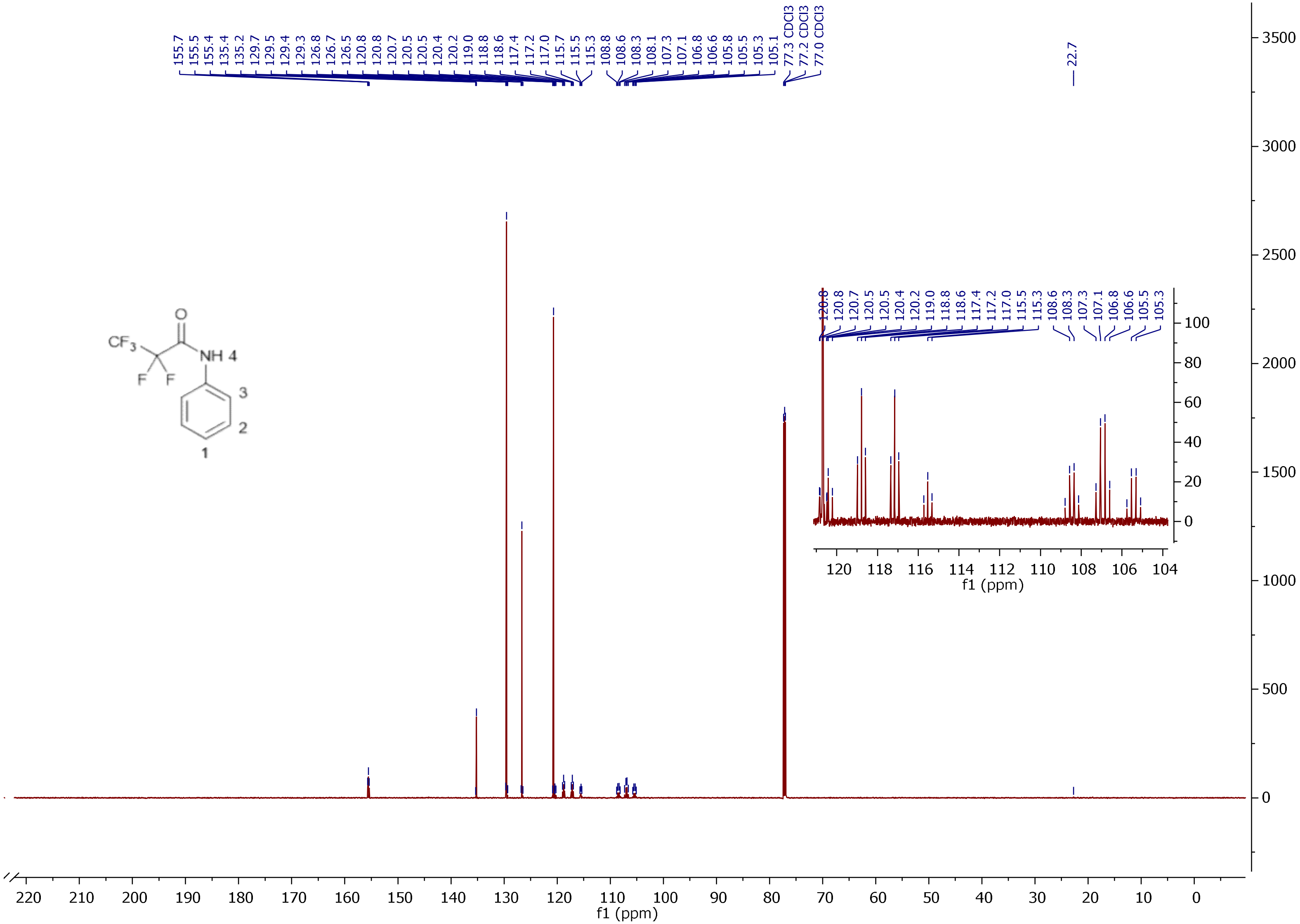

^{19}F NMR (376 MHz, $CDCl_3$) for 2,2,3,3,3-pentafluoro-*N*-phenylpropanamide **(1d)**

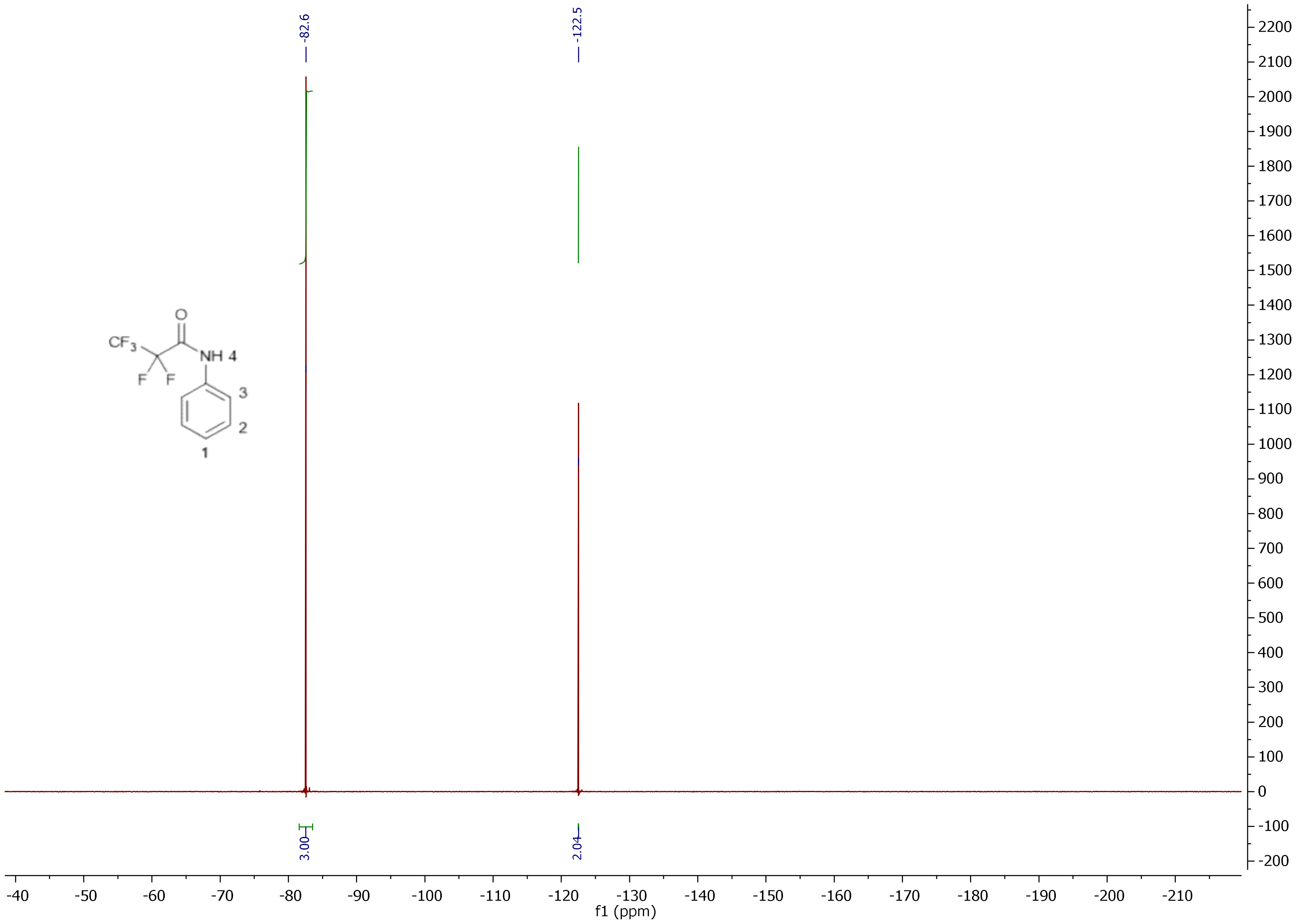

[1]**H NMR** (400 MHz, $CDCl_3$) for 2,2,3,3,4,4,4-heptafluoro-*N*-phenylbutanamide **(1e)**

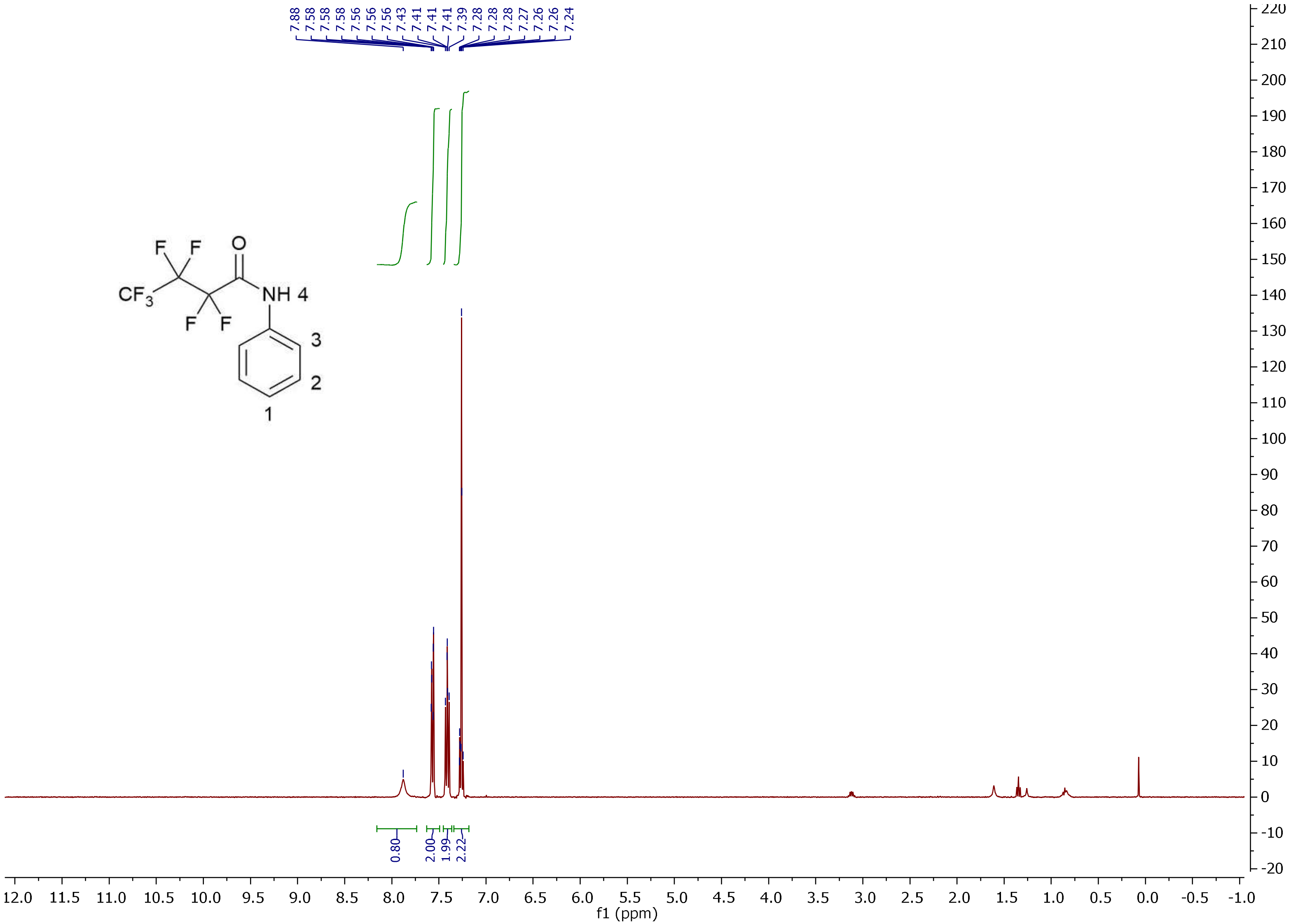

^{13}C NMR (176 MHz, $CDCl_3$) for 2,2,3,3,4,4,4-heptafluoro-*N*-phenylbutanamide **(1e)**

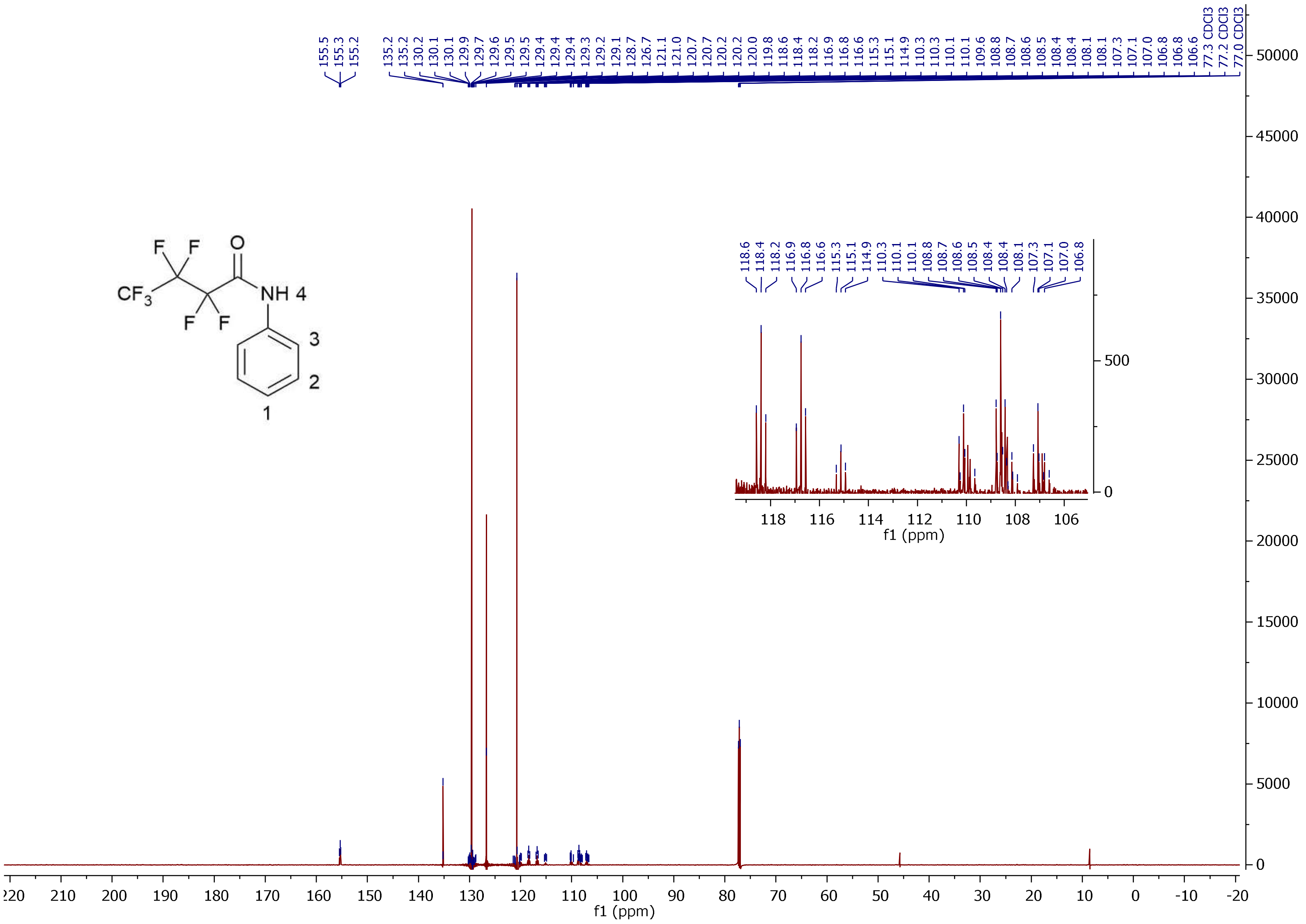

^{19}F NMR (376 MHz, $CDCl_3$) for 2,2,3,3,4,4,4-heptafluoro-*N*-phenylbutanamide **(1e)**

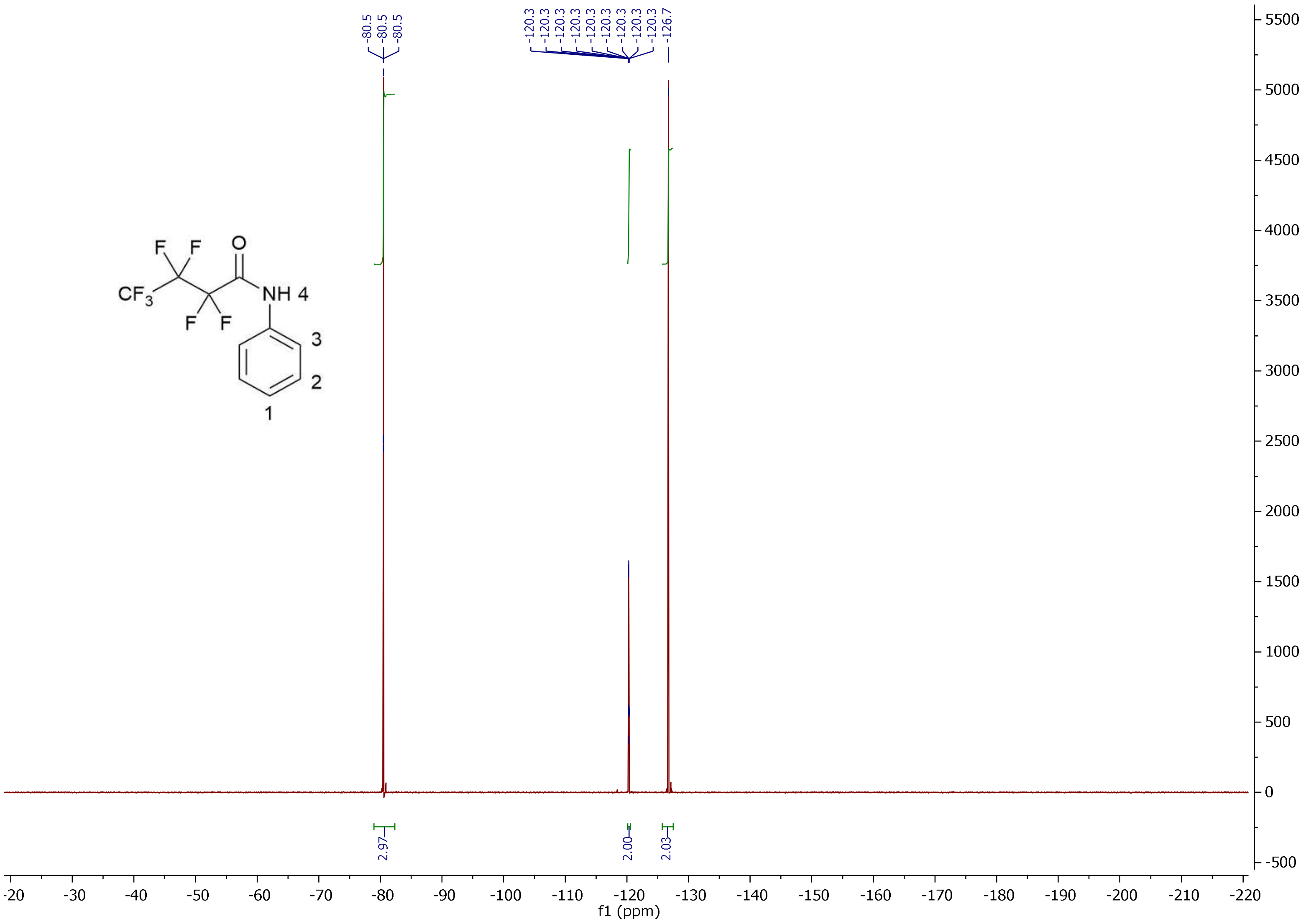

^{1}H NMR (400 MHz, $CDCl_3$) for 2,3,4,5,6-pentafluoro-*N*-phenylbenzamide **(1f)**

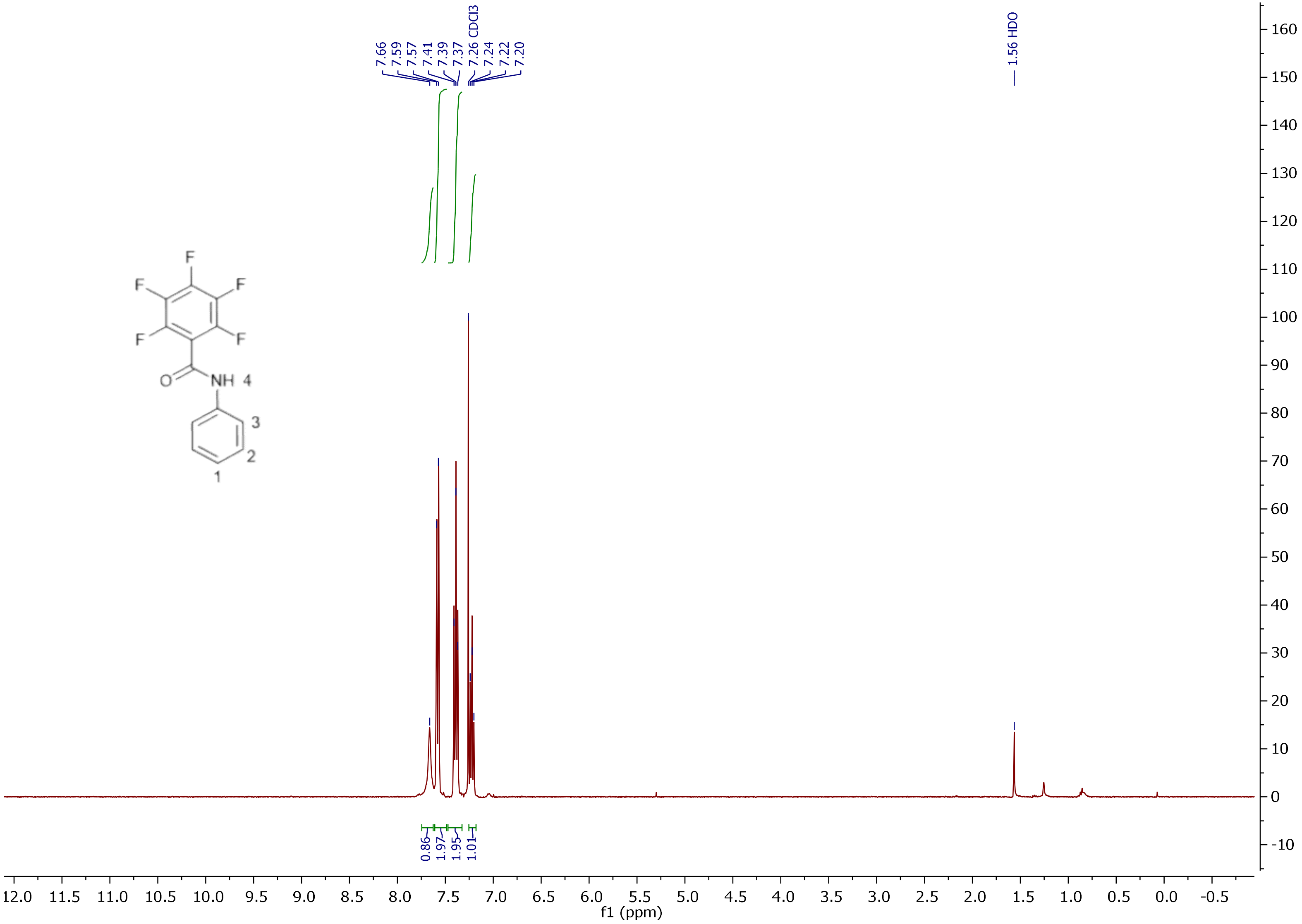

^{13}C NMR (176 MHz, CD_3CN) for 2,3,4,5,6-pentafluoro-*N*-phenylbenzamide **(1f)**

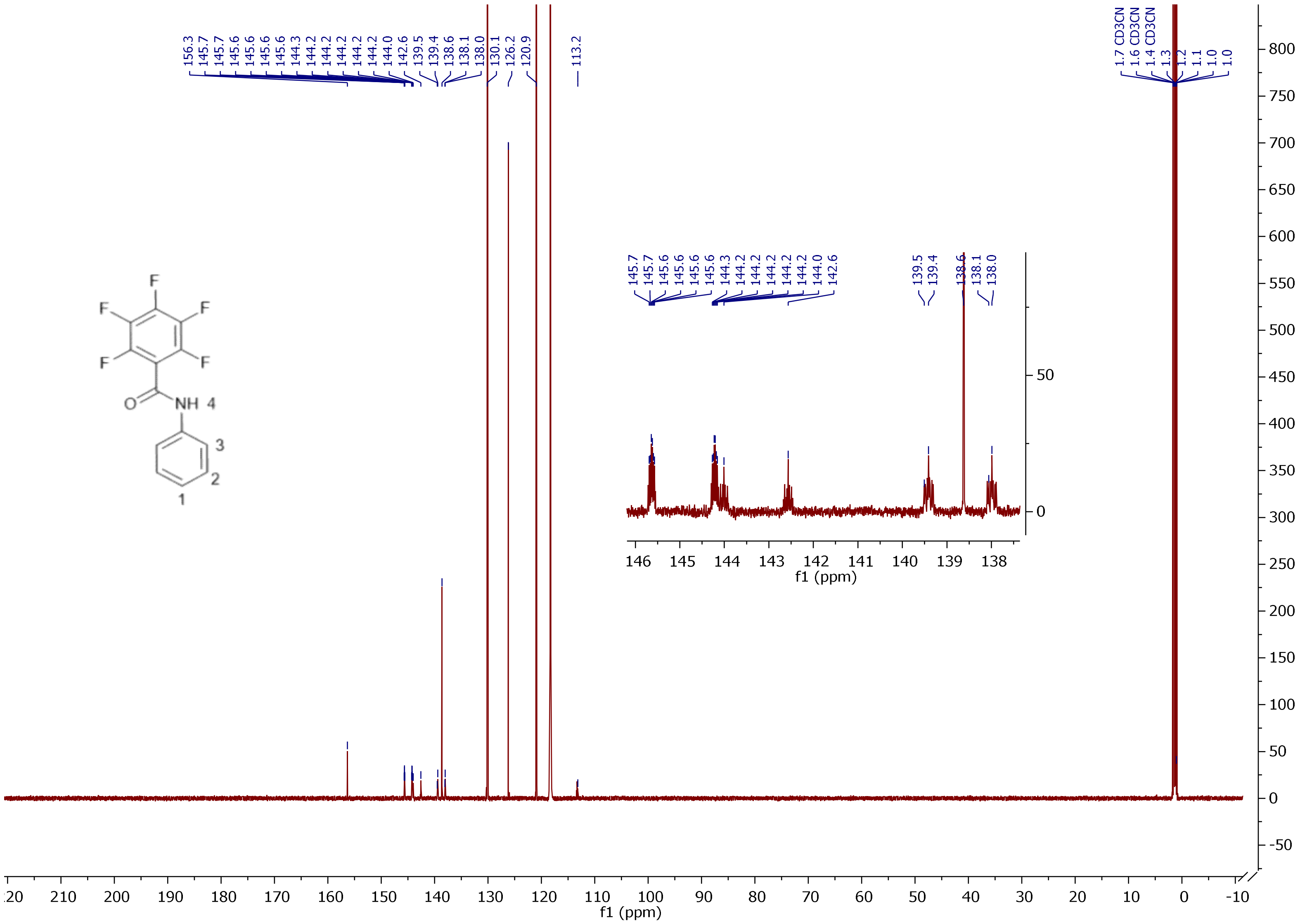

^{19}F NMR (376 MHz, $CDCl_3$) for 2,3,4,5,6-pentafluoro-*N*-phenylbenzamide **(1f)**

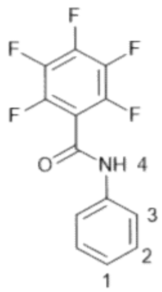

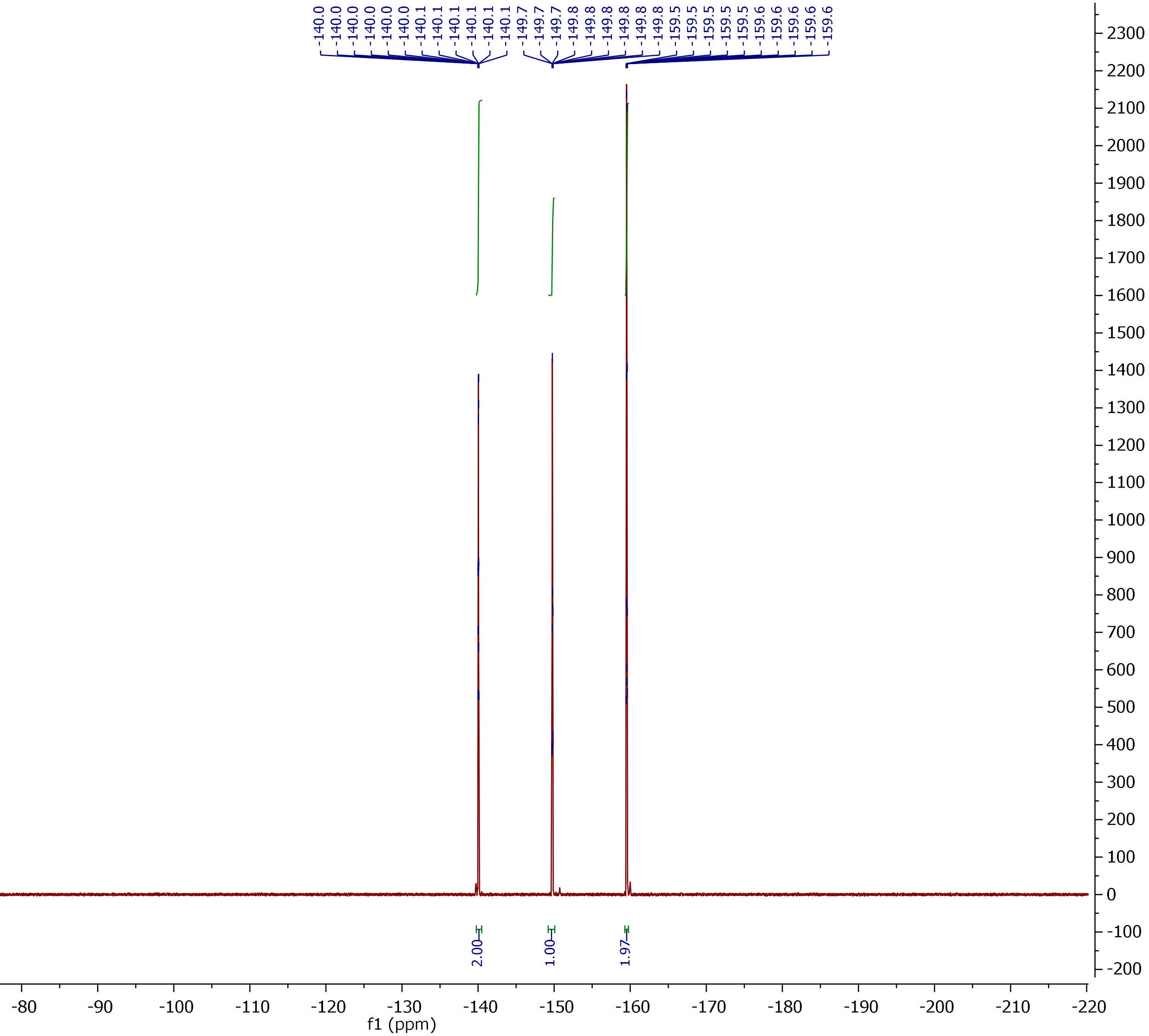

^{1}H NMR (500 MHz, $CDCl_3$) for 2,2,2-trichloro-*N*-phenylacetamide **(1g)**

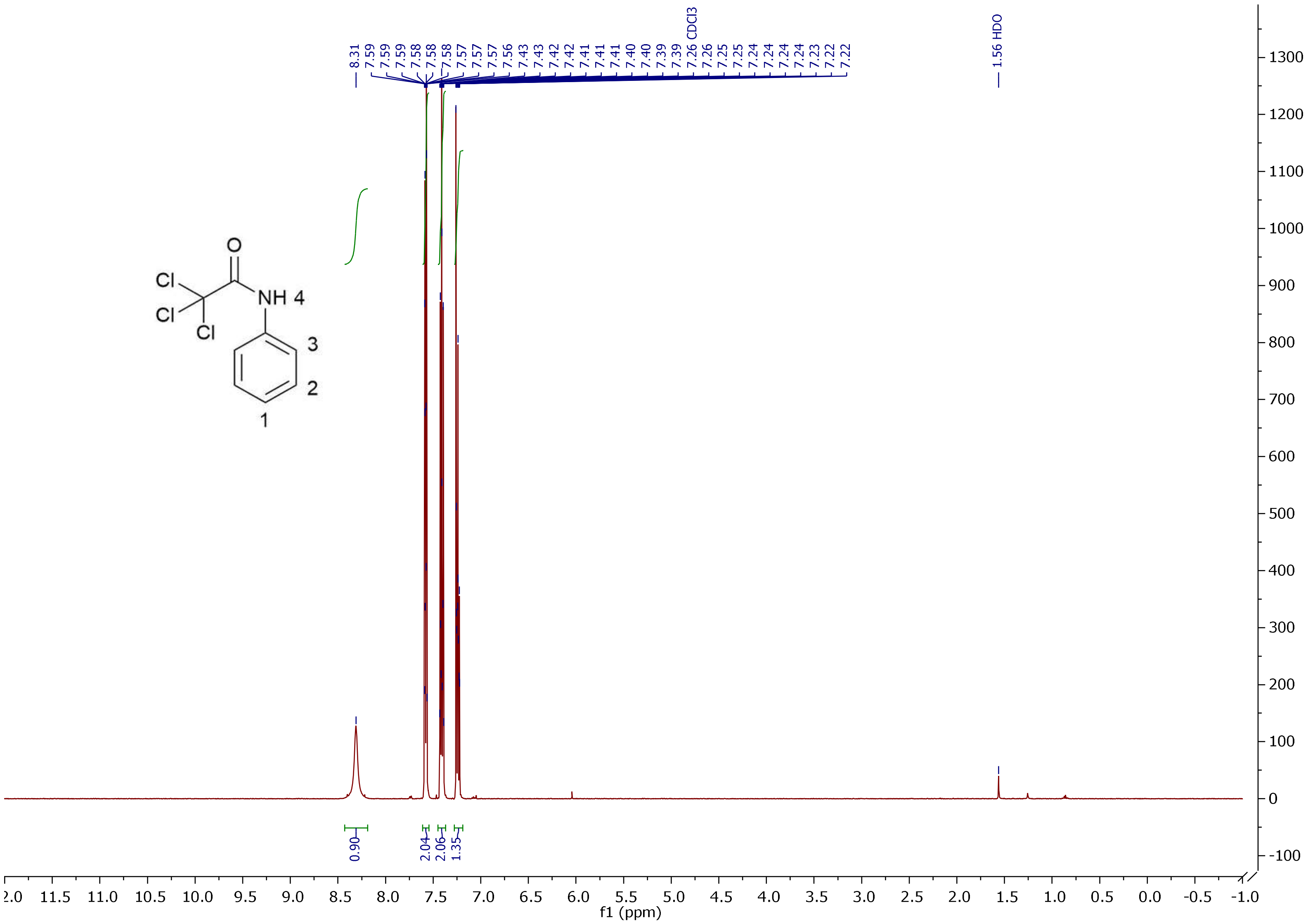

^{13}C NMR (126 MHz, $CDCl_3$) for 2,2,2-trichloro-*N*-phenylacetamide **(1g)**

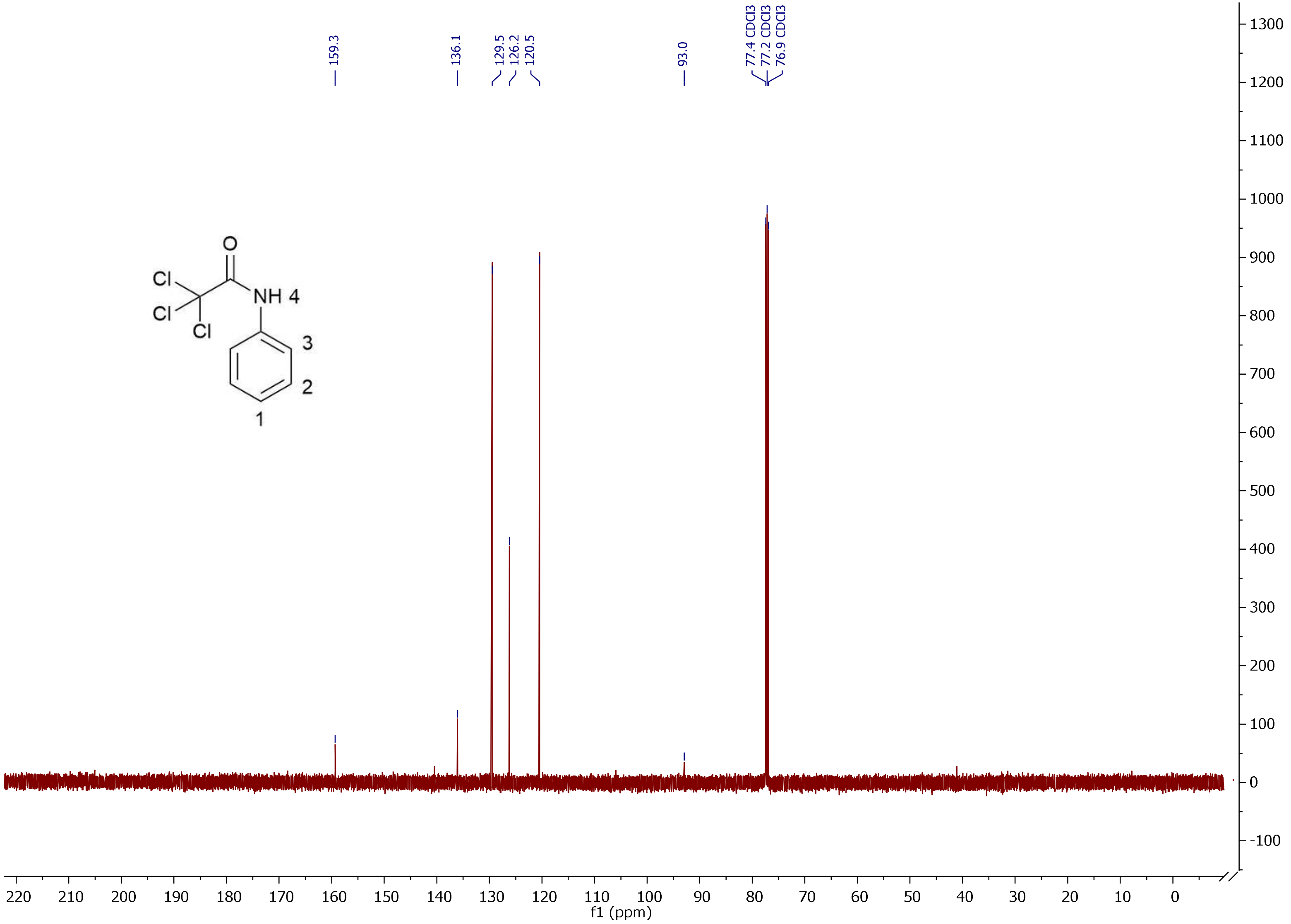

^{1}H NMR (500 MHz, $CDCl_3$) for 2,2,2-trichloroethyl phenylcarbamate **(1g)**

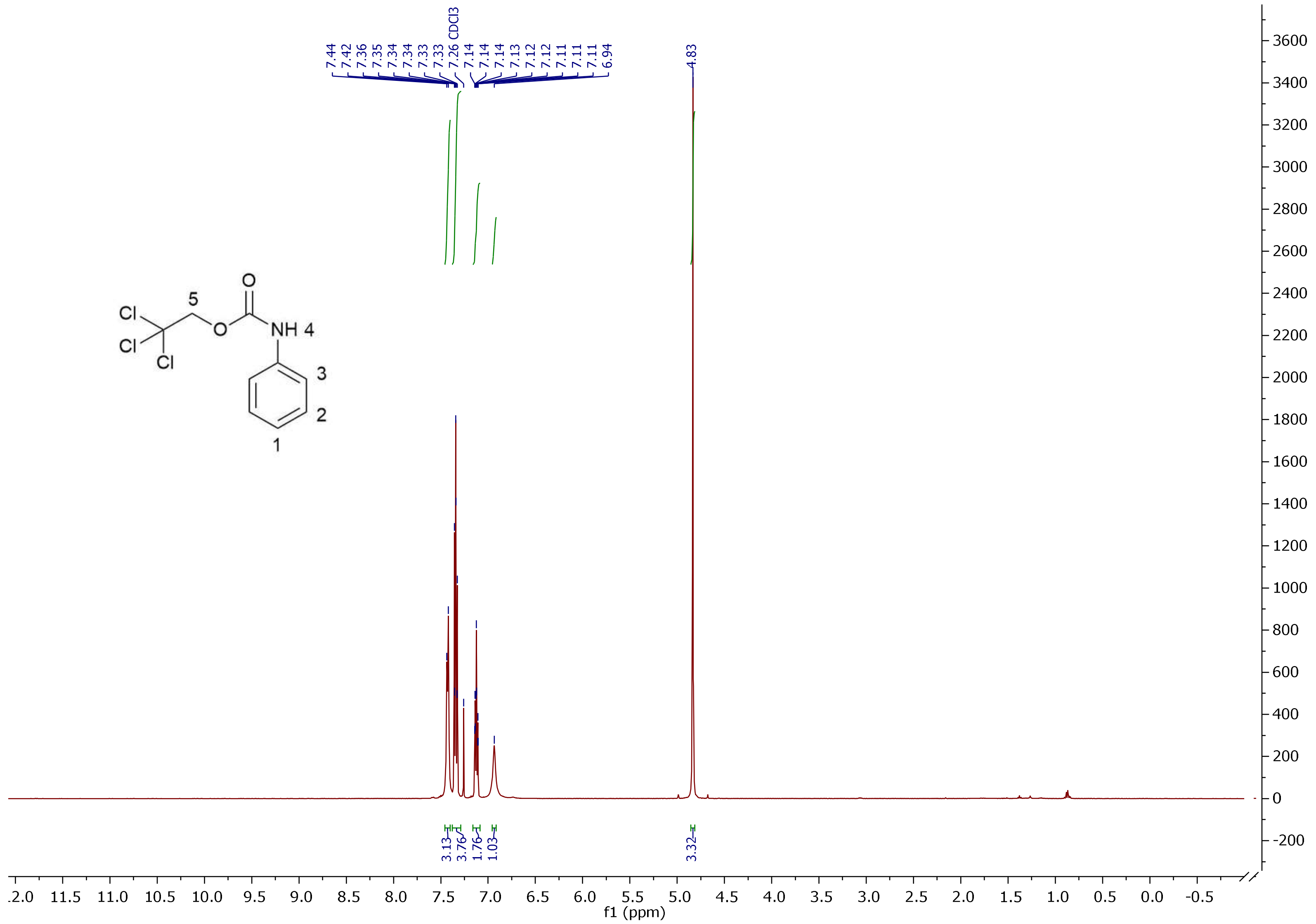

^{13}C NMR (126 MHz, $CDCl_3$) for 2,2,2-trichloroethyl phenylcarbamate **(1h)**

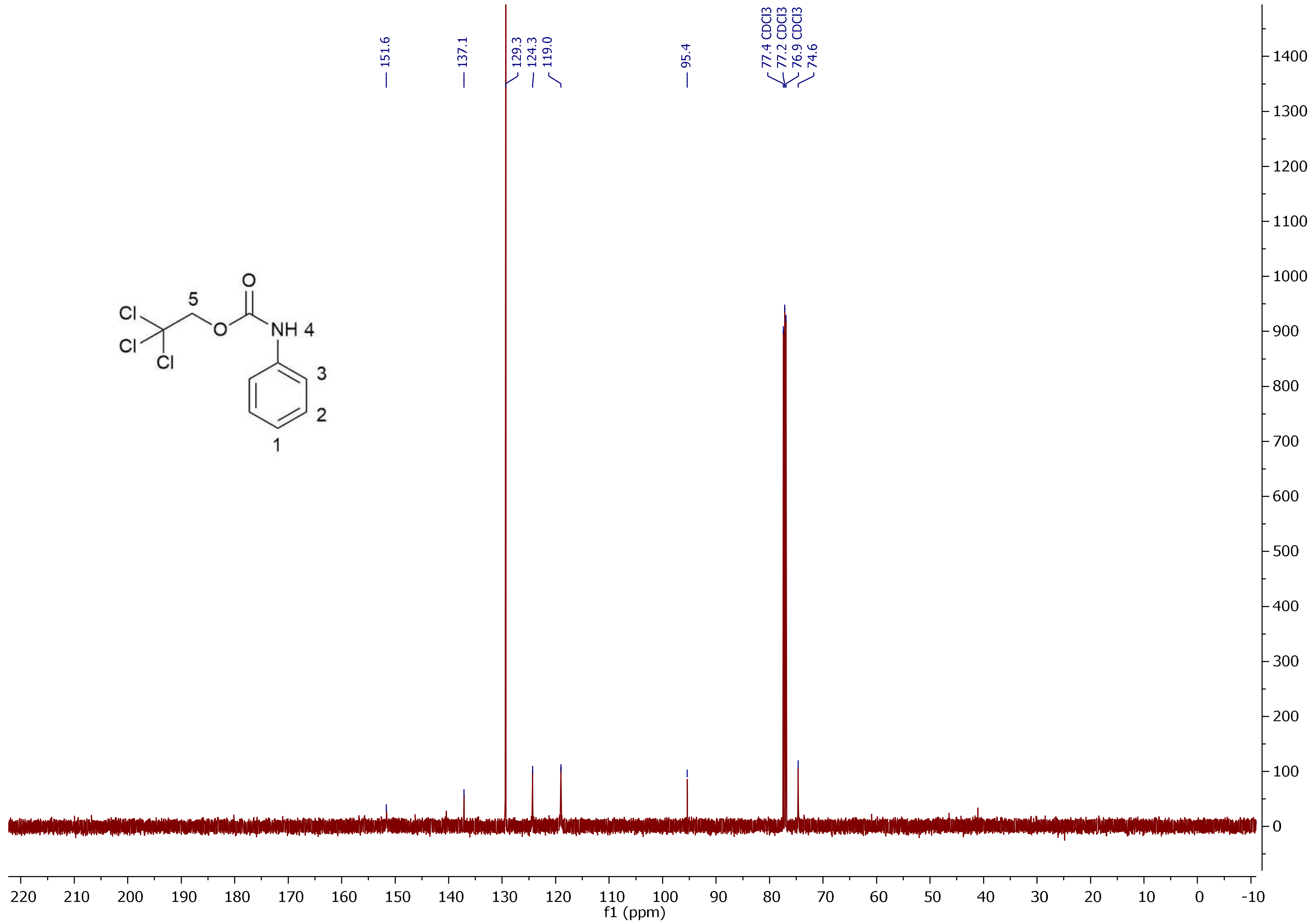

^{1}H NMR (400 MHz, $CDCl_3$) for 2,2,2-trifluoro-*N*-(*o*-tolyl)acetamide **(1i)**

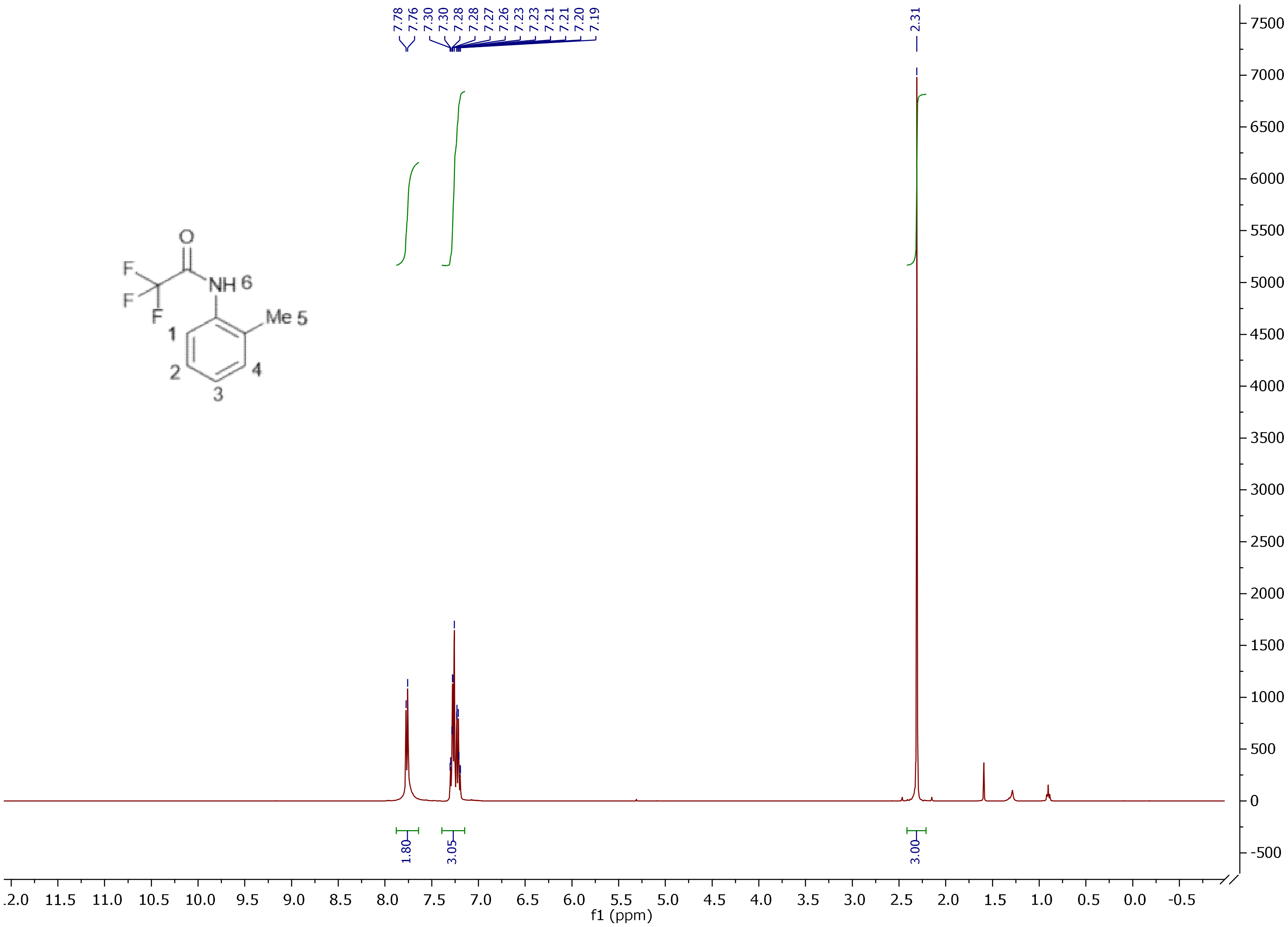

^{13}C NMR (126 MHz, $CDCl_3$) for 2,2,2-trifluoro-*N*-(*o*-tolyl)acetamide **(1i)**

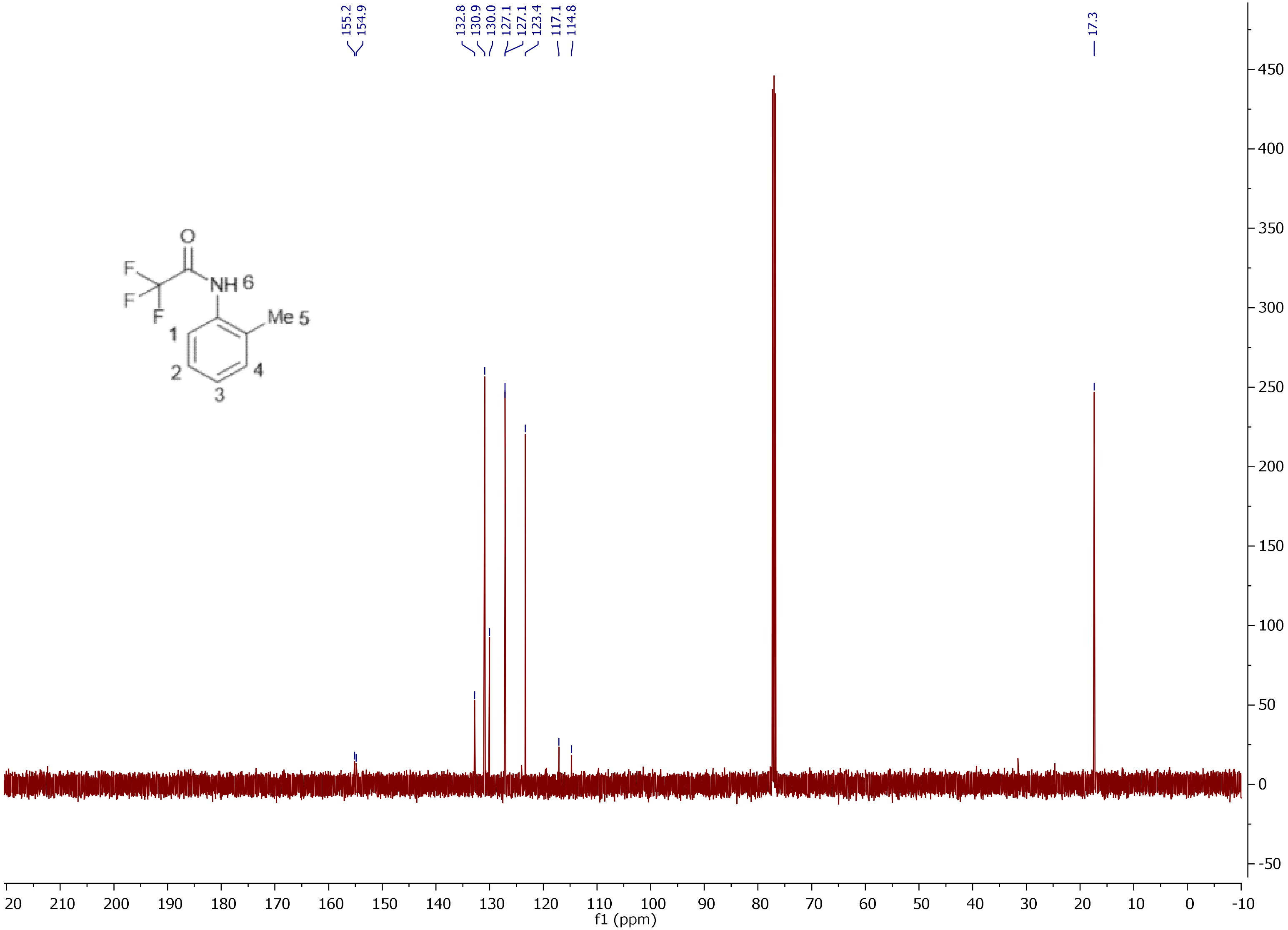

^{19}F NMR (376 MHz, $CDCl_3$) for 2,2,2-trifluoro-*N*-(*o*-tolyl)acetamide **(1i)**

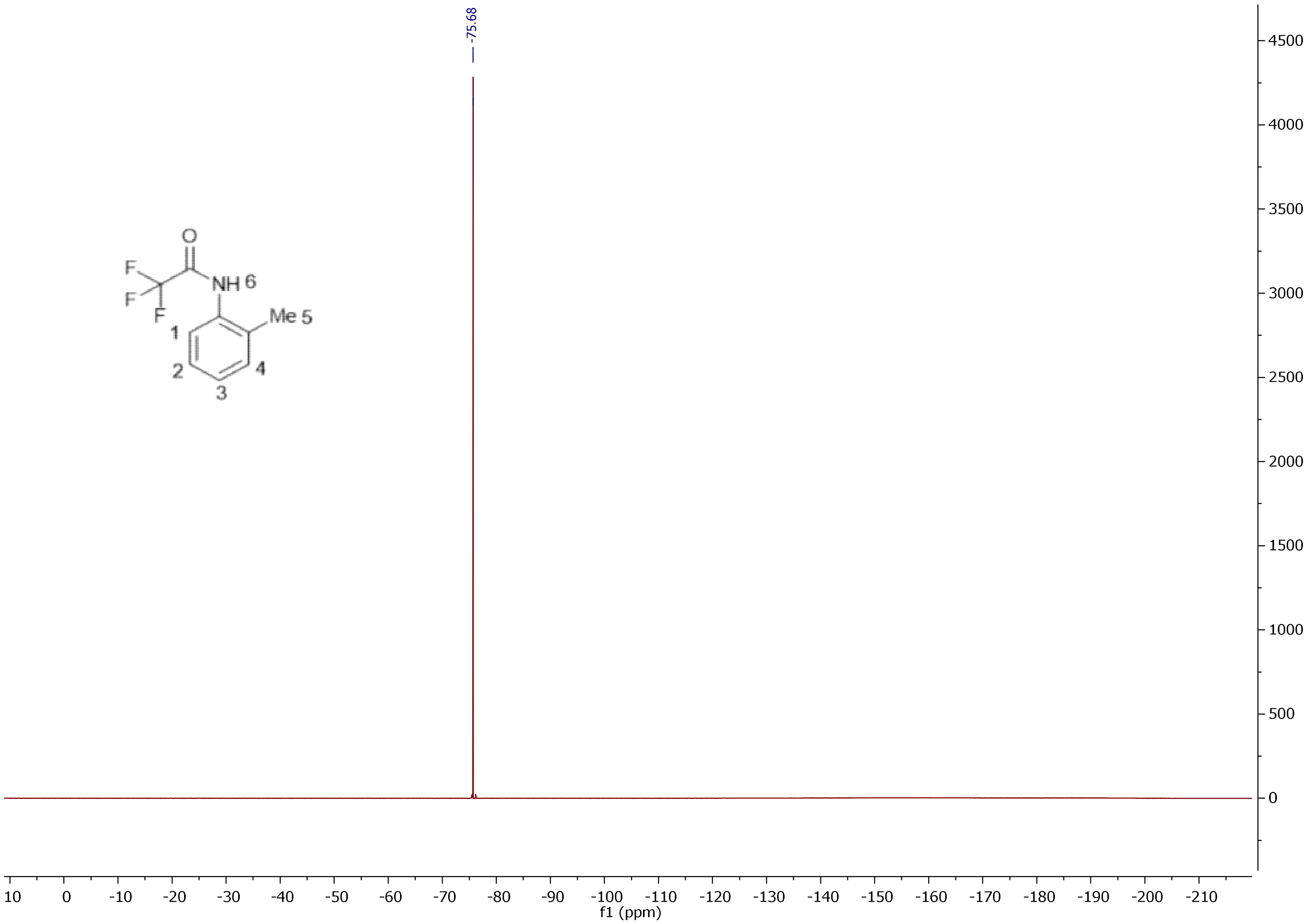

^{1}H NMR (400 MHz, $CDCl_3$) for 2,2,2-trifluoro-*N*-(*m*-tolyl)acetamide **(1j)**

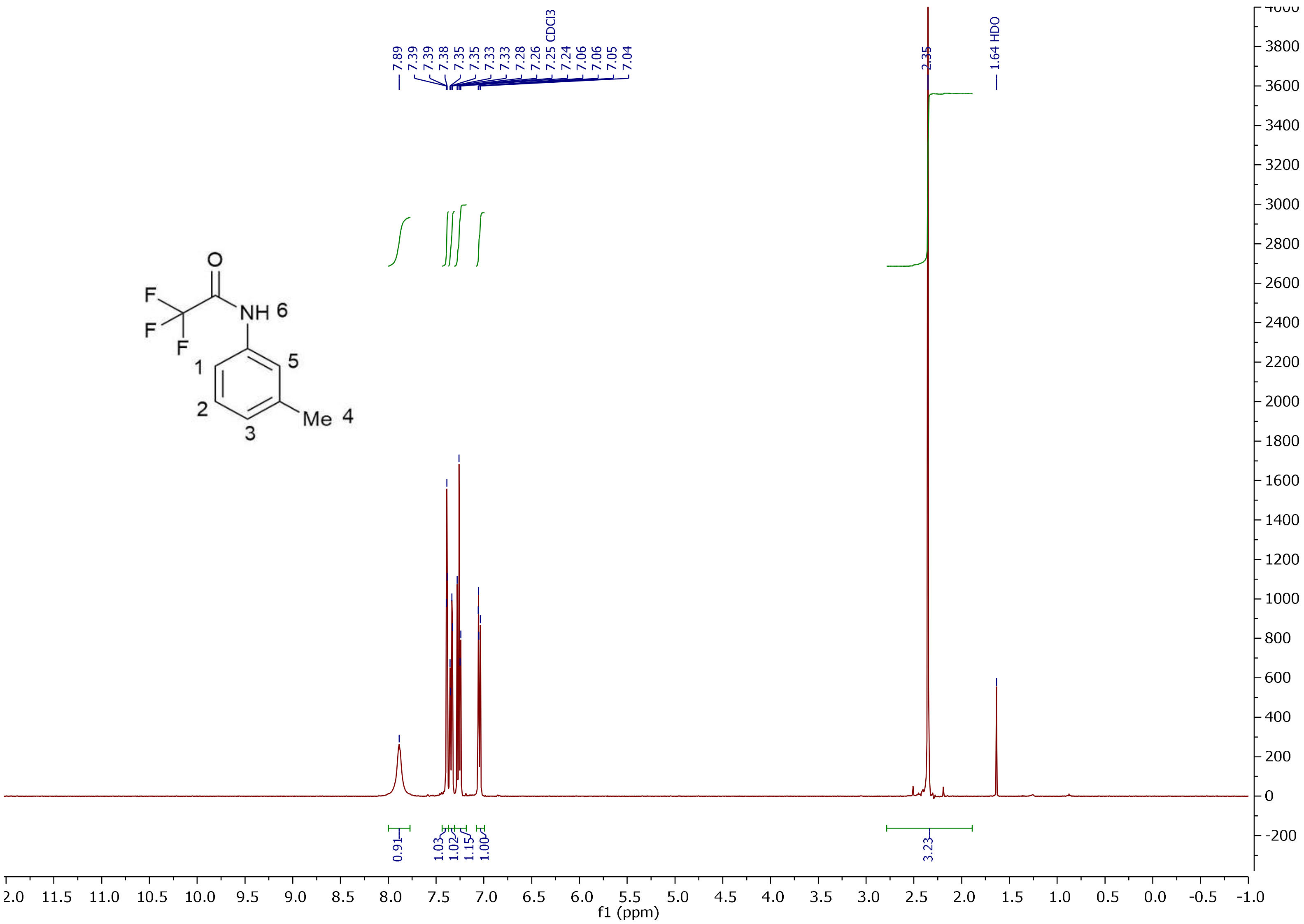

^{13}C NMR (126 MHz, $CDCl_3$) for 2,2,2-trifluoro-*N*-(*m*-tolyl)acetamide **(1j)**

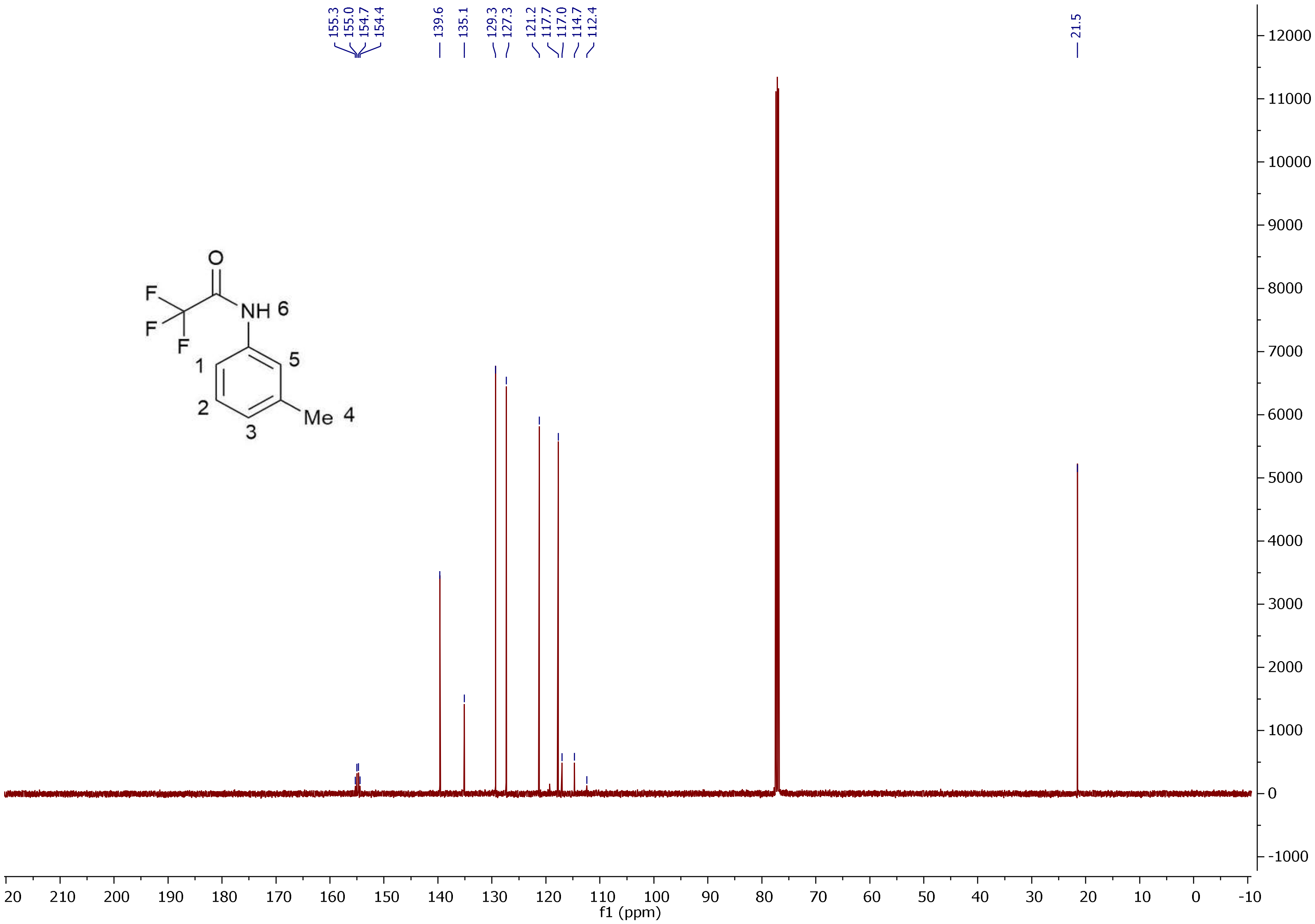

^{19}F NMR (376 MHz, $CDCl_3$) for 2,2,2-trifluoro-*N*-(*m*-tolyl)acetamide **(1j)**

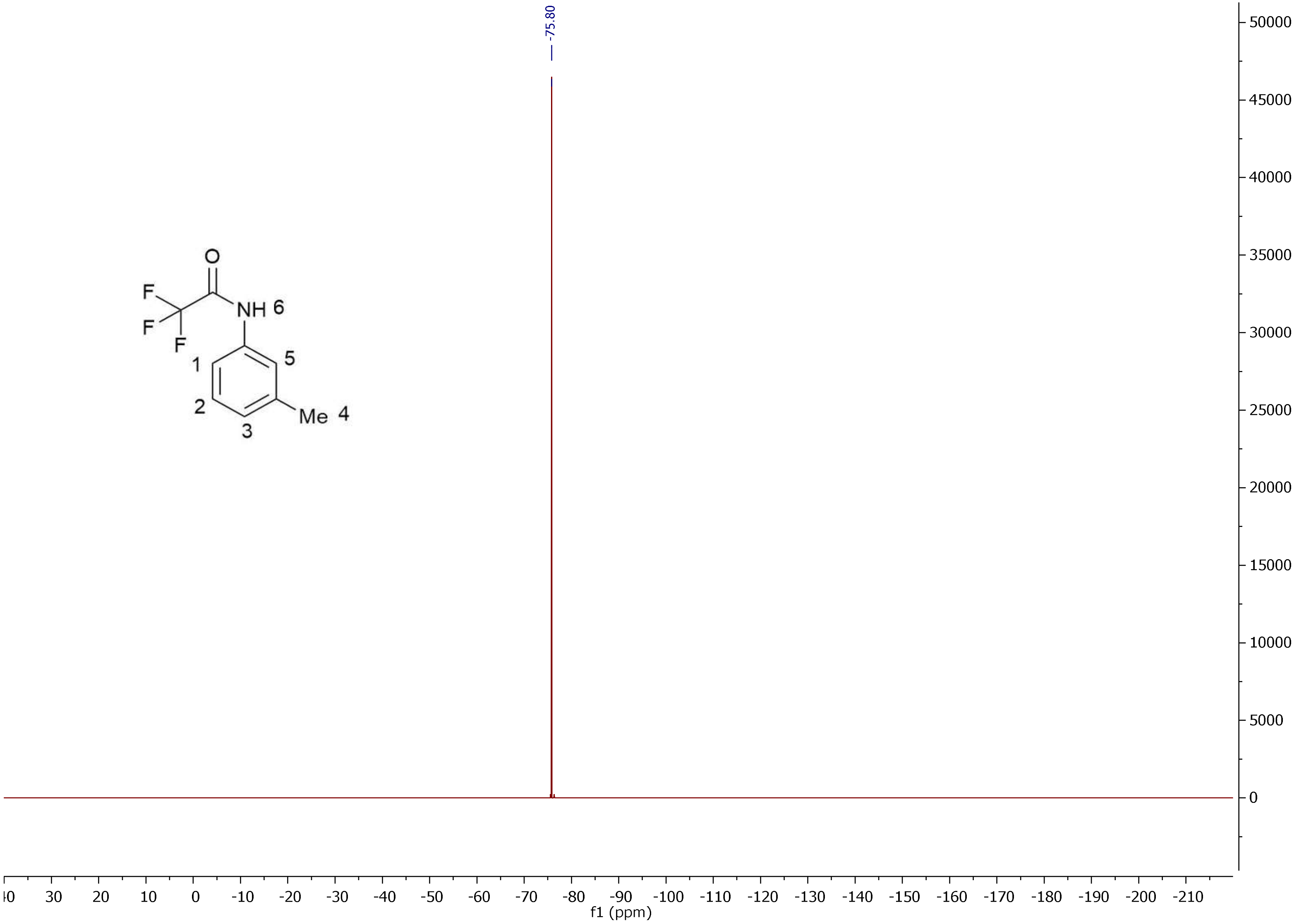

^{1}H NMR (400 MHz, $CDCl_3$) for *N*-(2,5-dimethylphenyl)-2,2,2-trifluoroacetamide **(1k)**

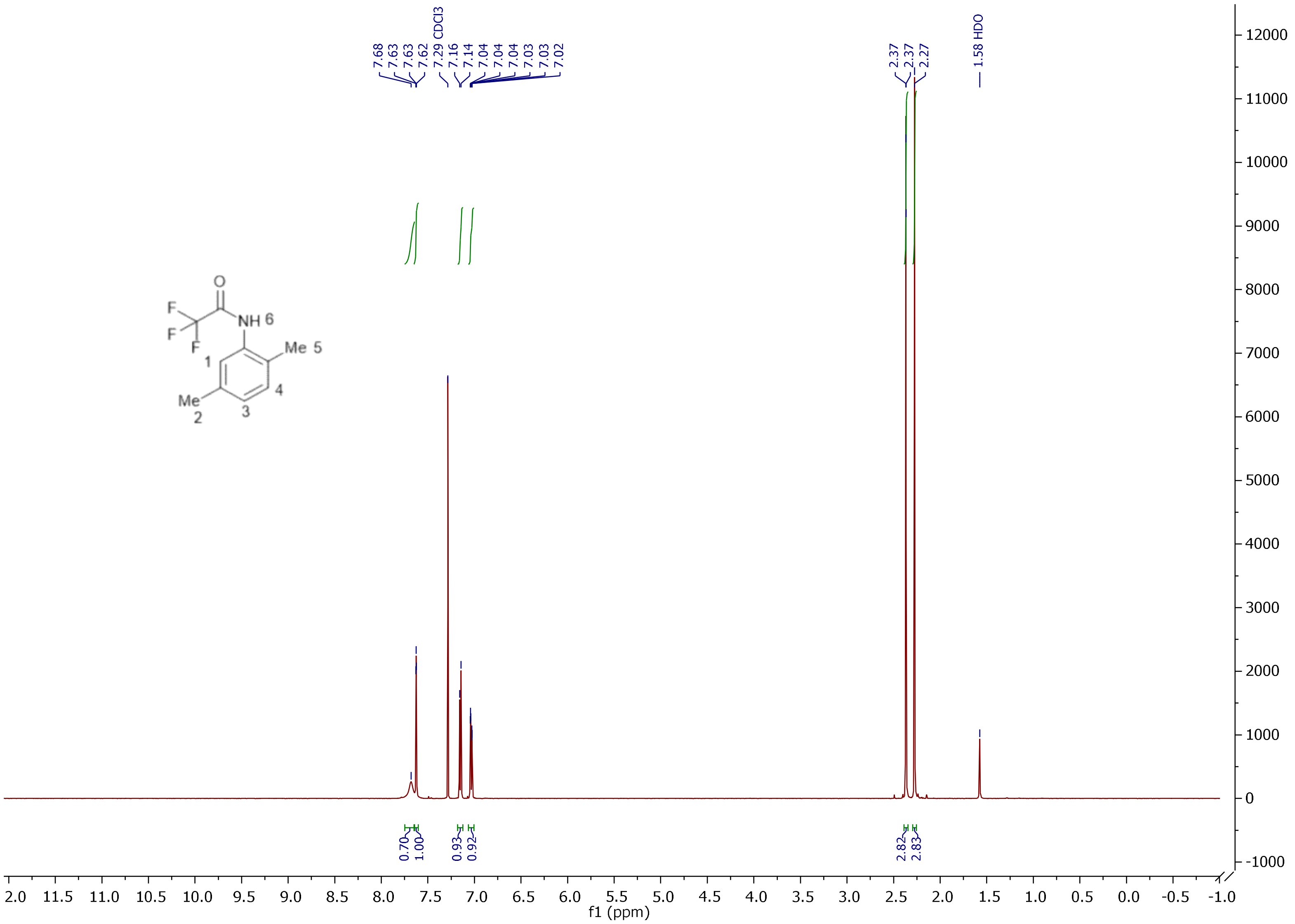

^{13}C NMR (126 MHz, $CDCl_3$) for *N*-(2,5-dimethylphenyl)-2,2,2-trifluoroacetamide **(1k)**

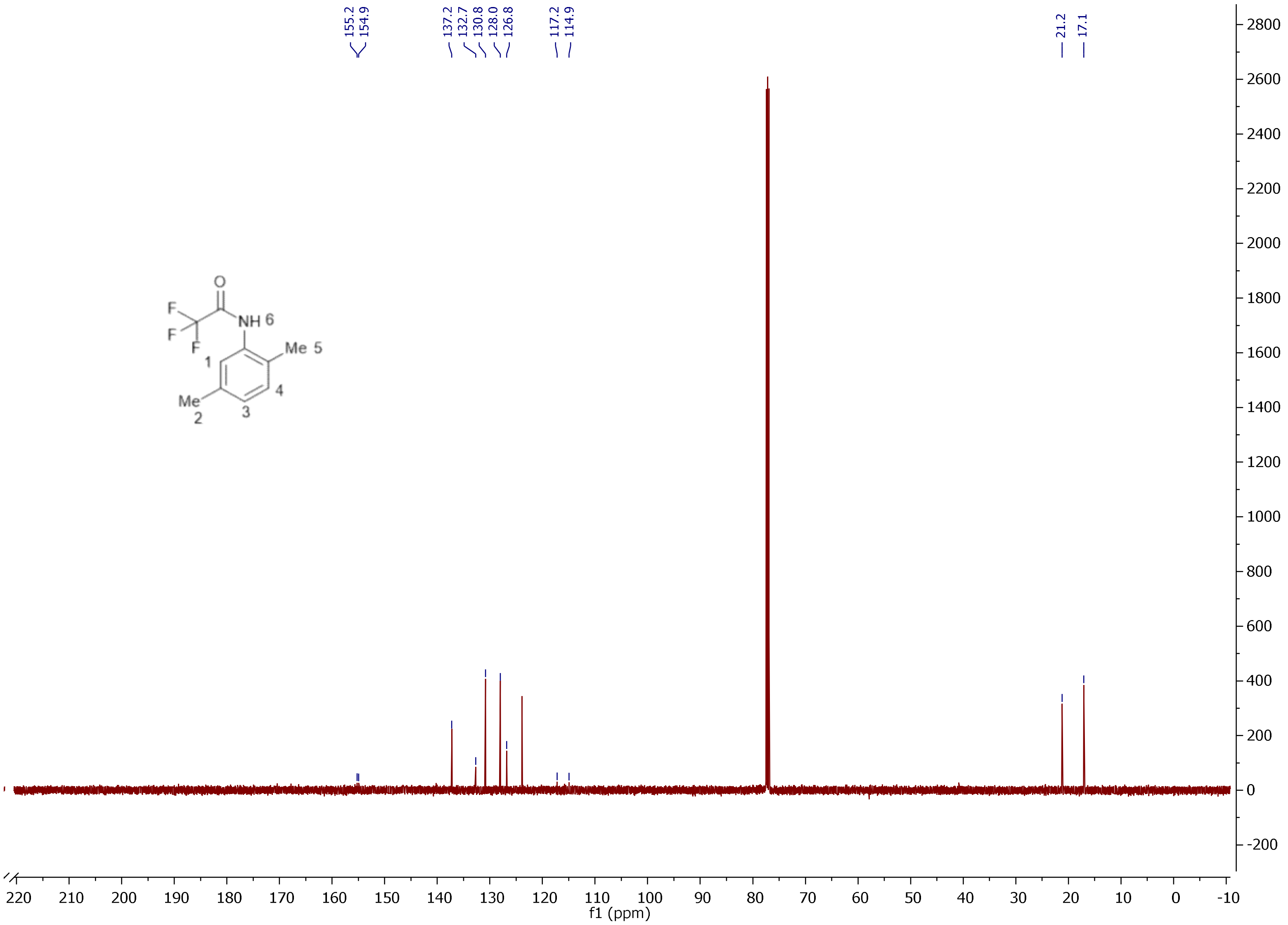

^{19}F NMR (376 MHz, $CDCl_3$) for *N*-(2,5-dimethylphenyl)-2,2,2-trifluoroacetamide **(1k)**

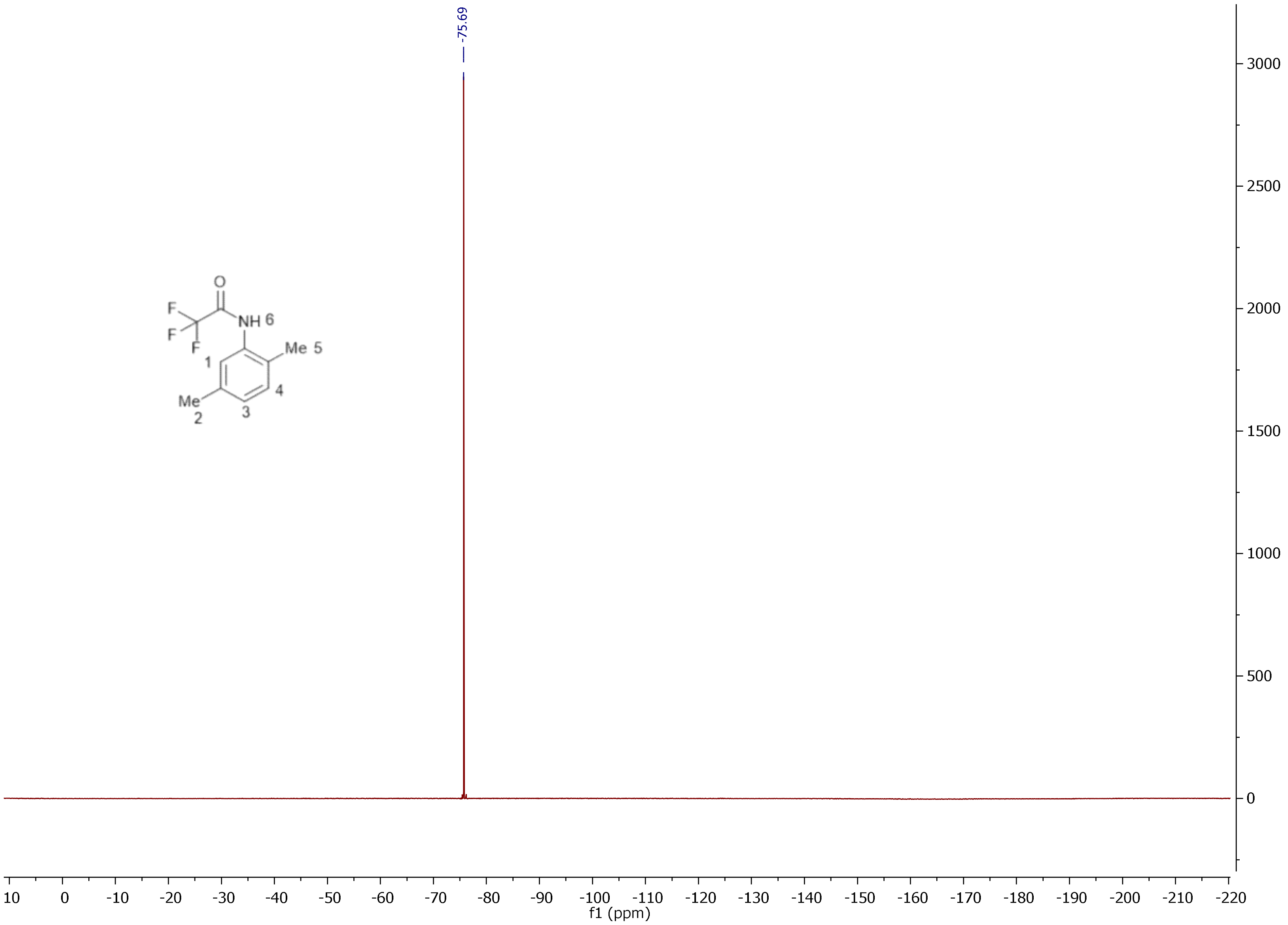

^{1}H NMR (400 MHz, $CDCl_3$) for 2,2,2-trifluoro-*N*-(5,6,7,8-tetrahydronaphthalen-1-yl)acetamide **(1l)**

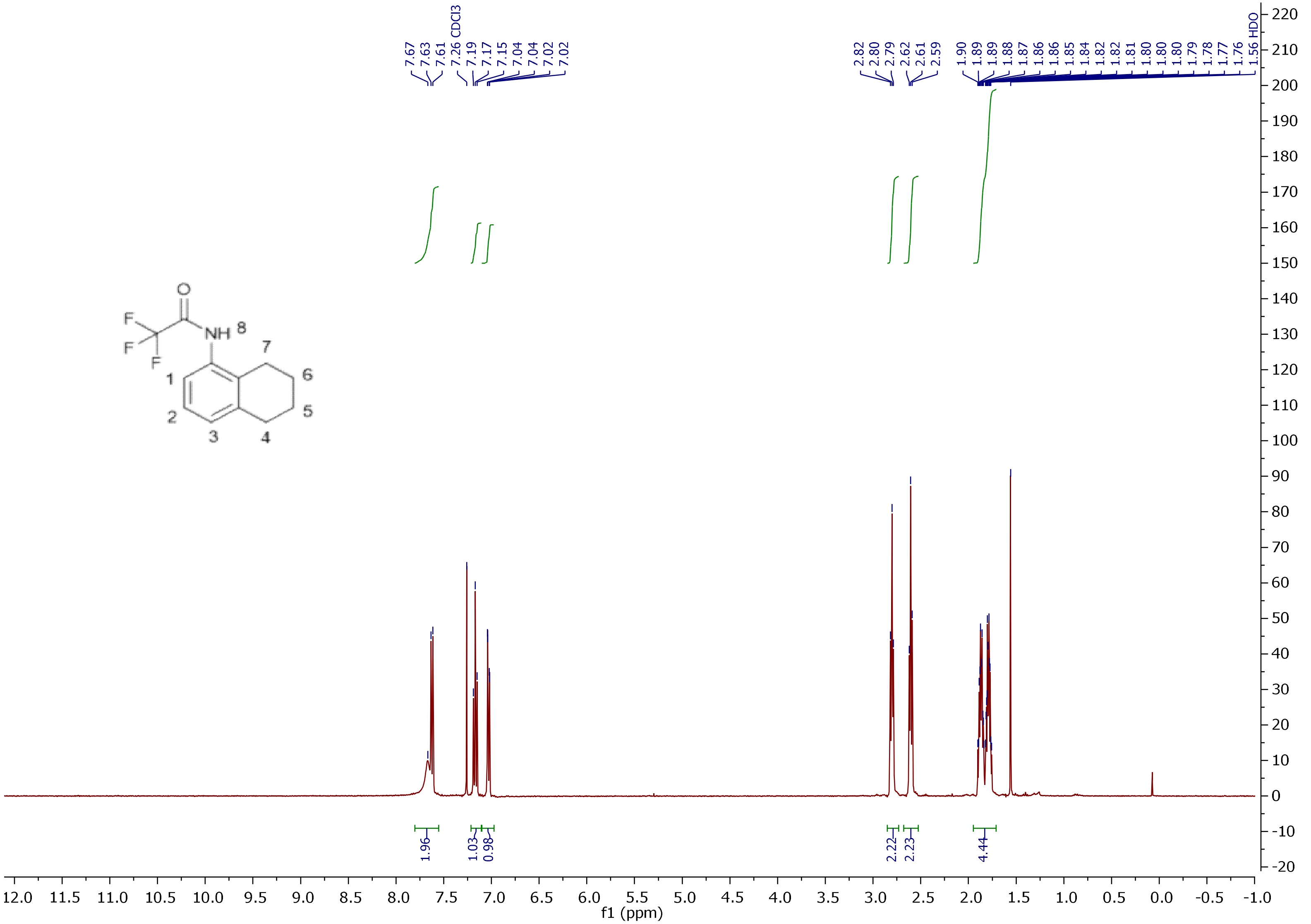

^{13}C NMR (126 MHz, $CDCl_3$) for 2,2,2-trifluoro-*N*-(5,6,7,8-tetrahydronaphthalen-1-yl)acetamide **(1l)**

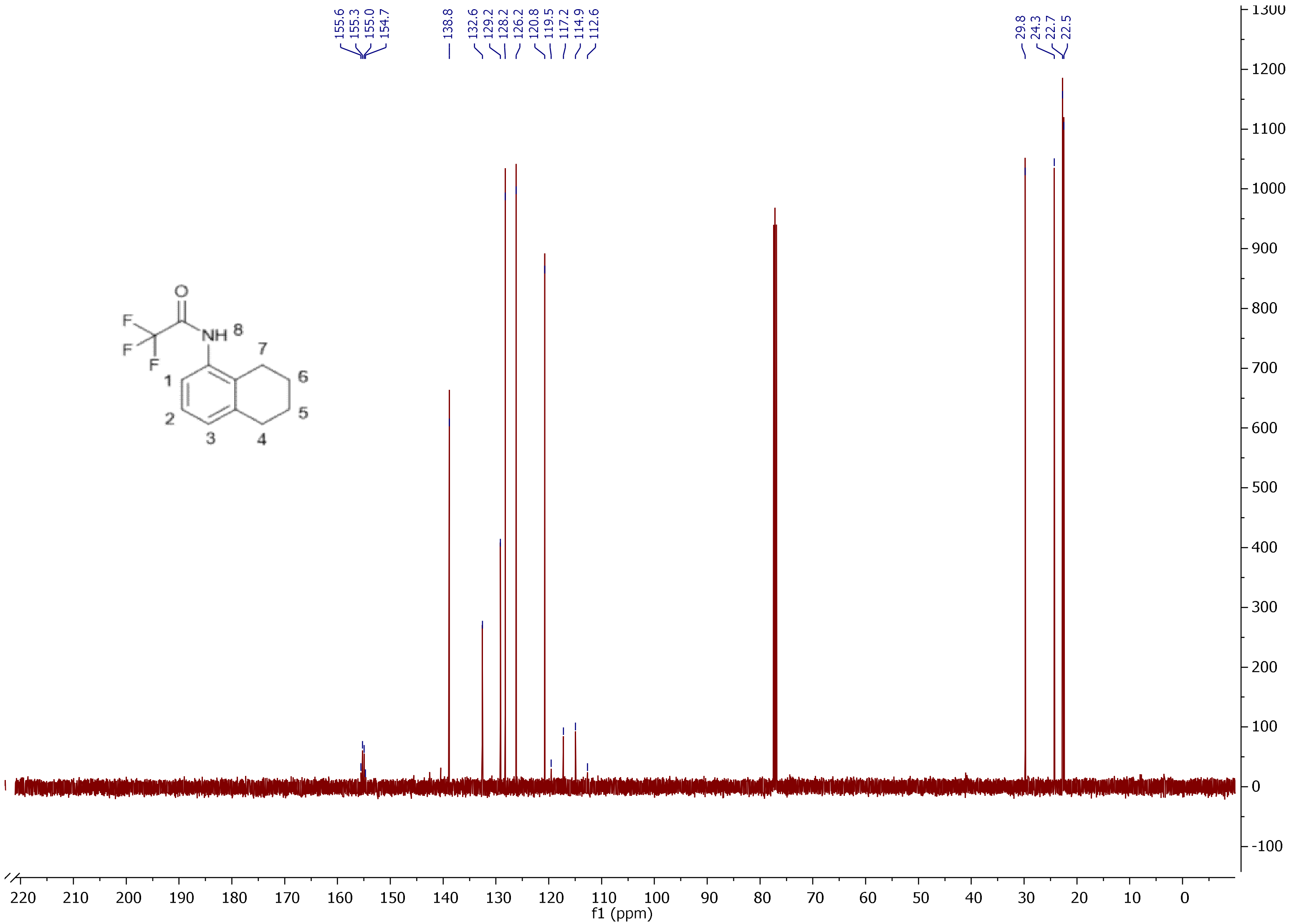

^{19}F NMR (376 MHz, $CDCl_3$) for 2,2,2-trifluoro-*N*-(5,6,7,8-tetrahydronaphthalen-1-yl)acetamide **(1l)**

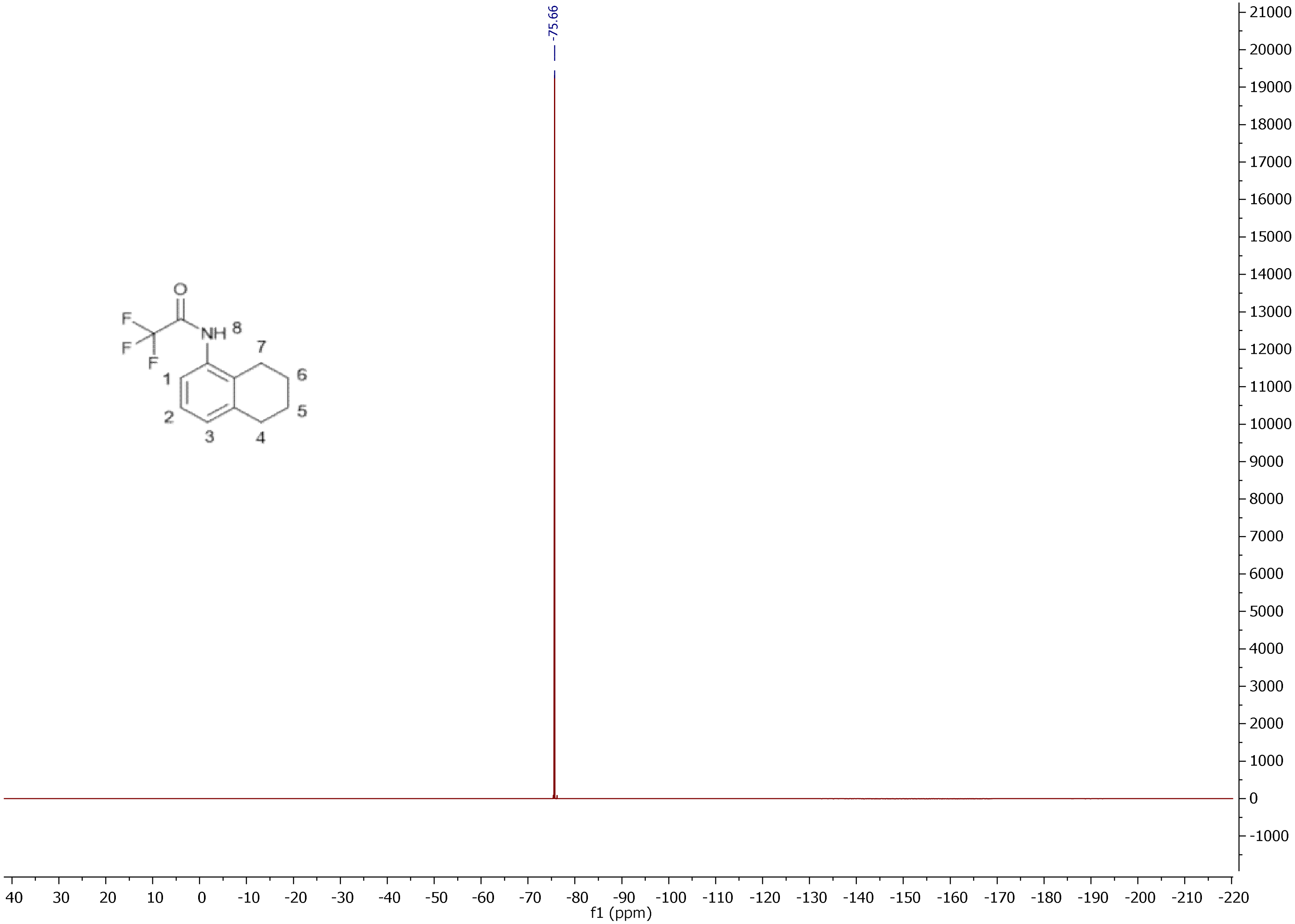

^{1}H NMR (400 MHz, $CDCl_3$) for 2,2,2-trifluoro-*N*-(2-isopropylphenyl)acetamide **(1m)**

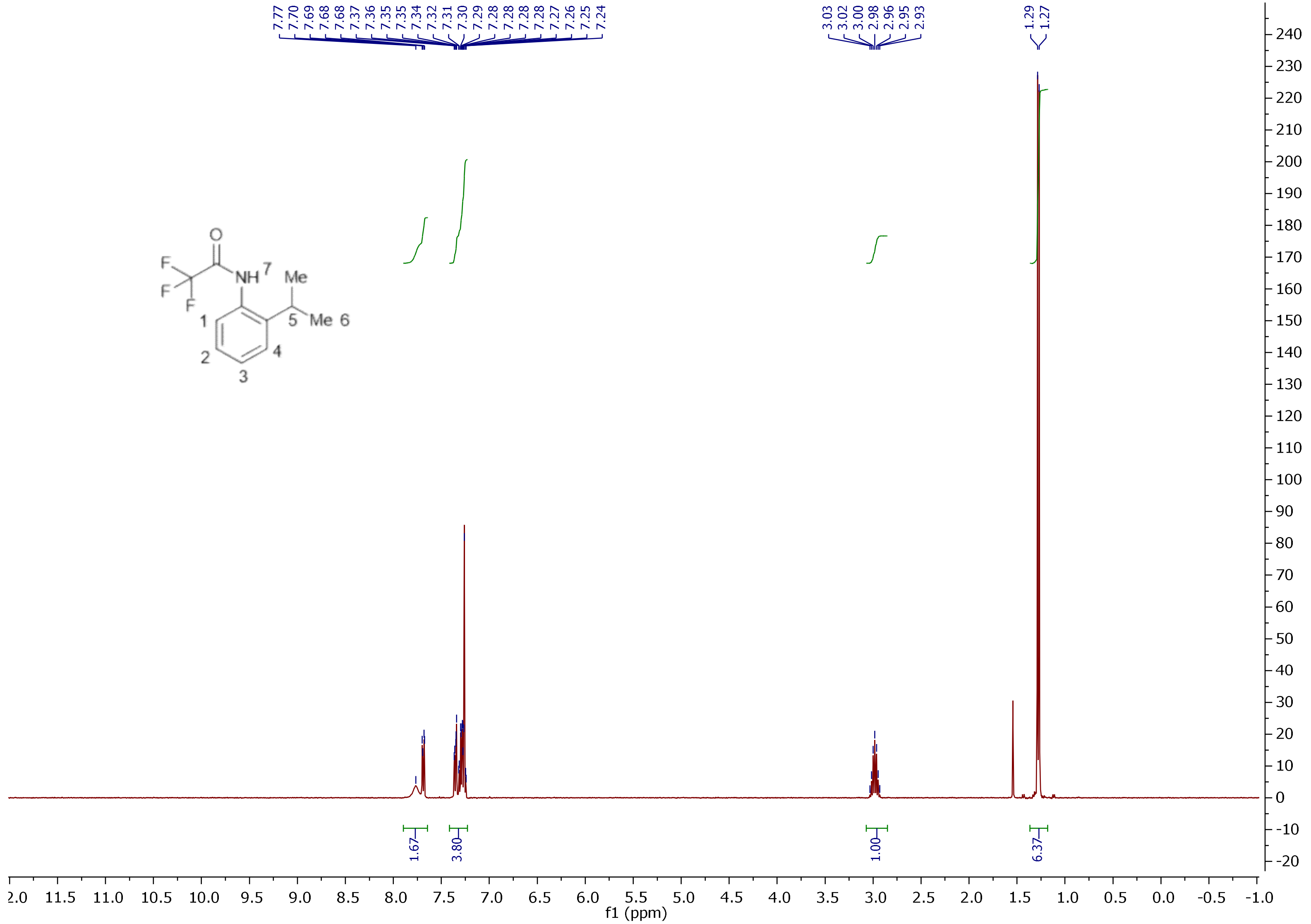

^{13}C NMR (126 MHz, $CDCl_3$) for 2,2,2-trifluoro-*N*-(2-isopropylphenyl)acetamide **(1m)**

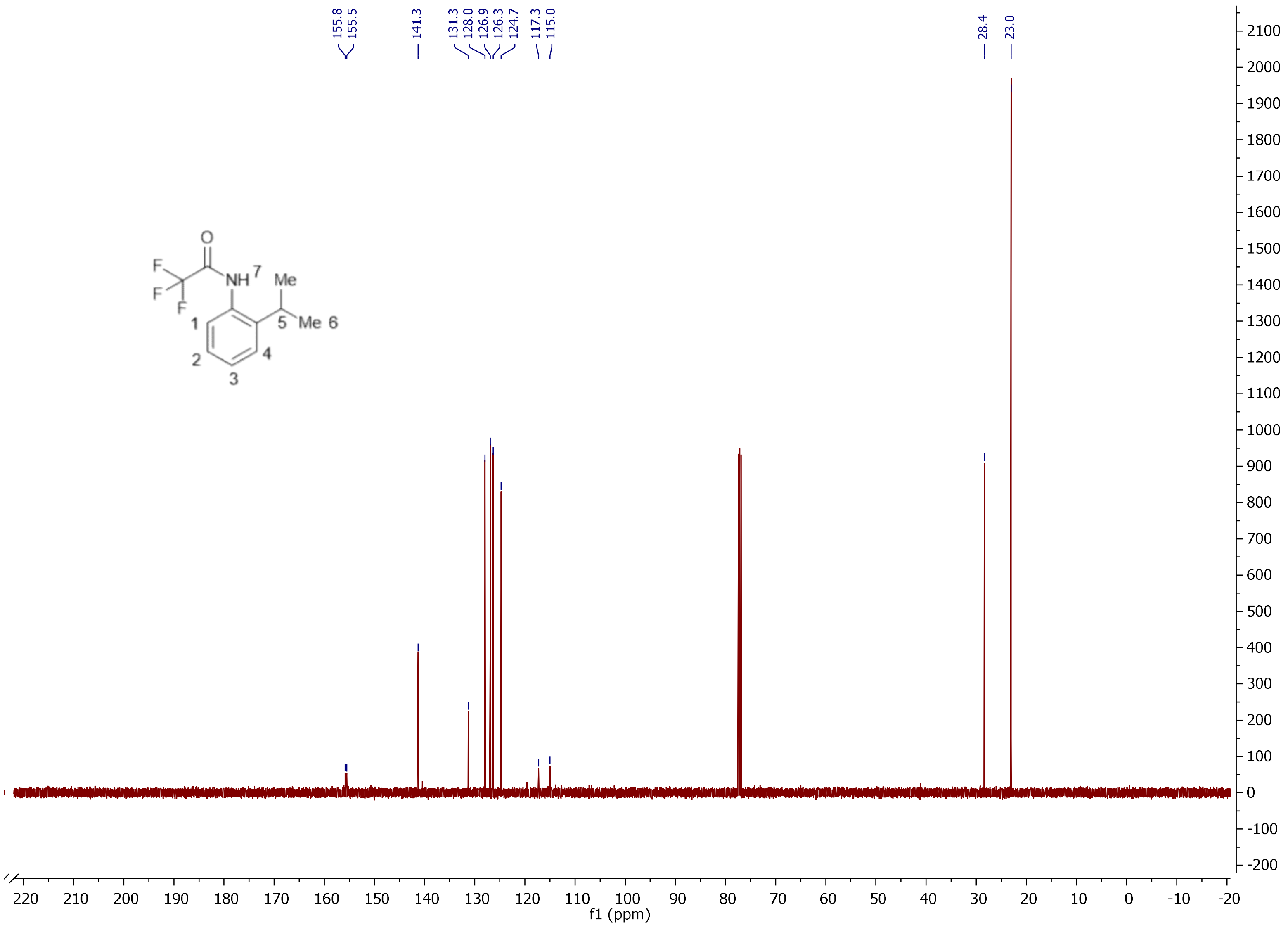

^{19}F NMR (376 MHz, $CDCl_3$) for 2,2,2-trifluoro-*N*-(2-isopropylphenyl)acetamide **(1m)**

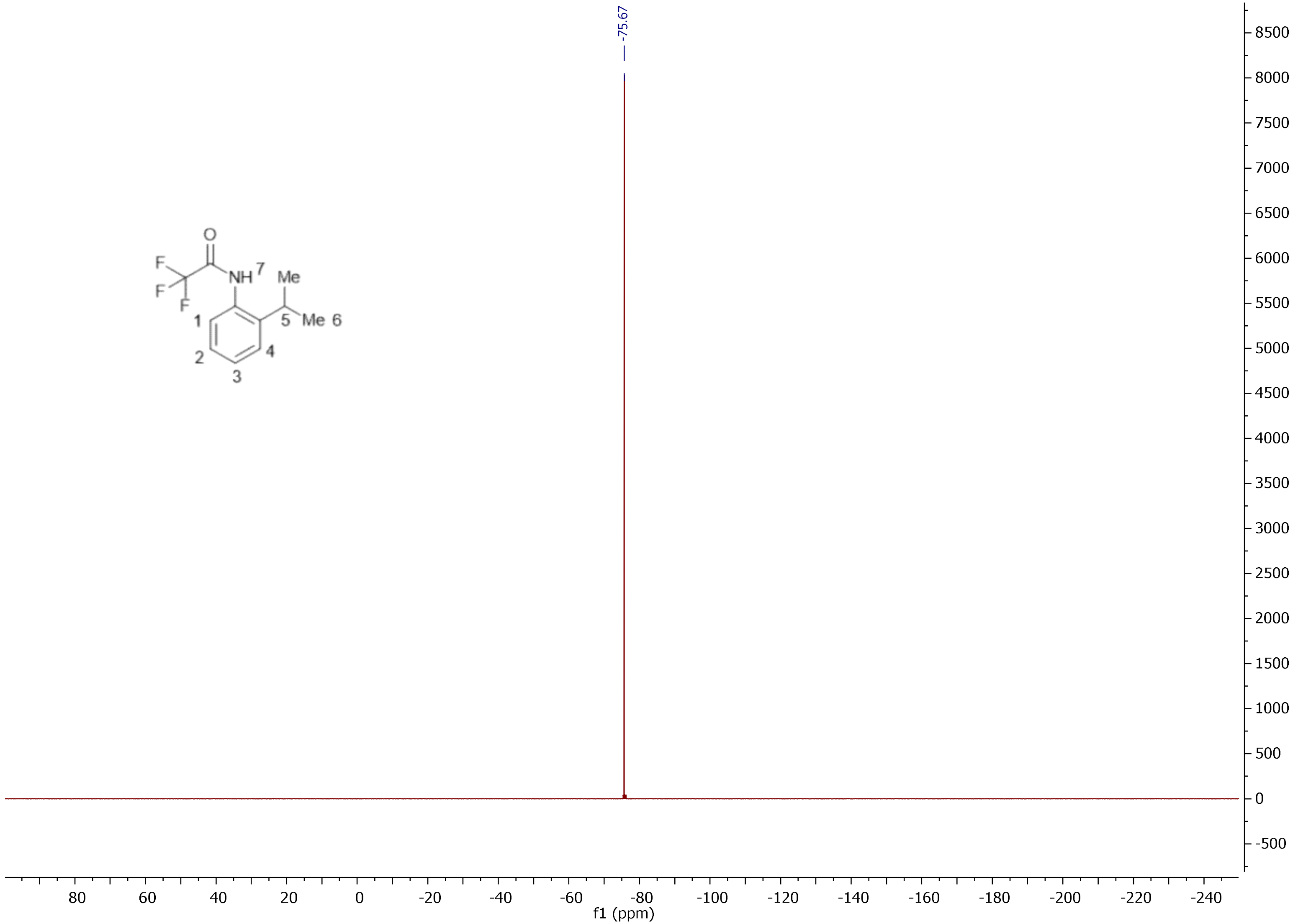

^{1}H NMR (400 MHz, $CDCl_3$) for 2,2,2-trifluoro-*N*-(3-fluorophenyl)acetamide **(1n)**

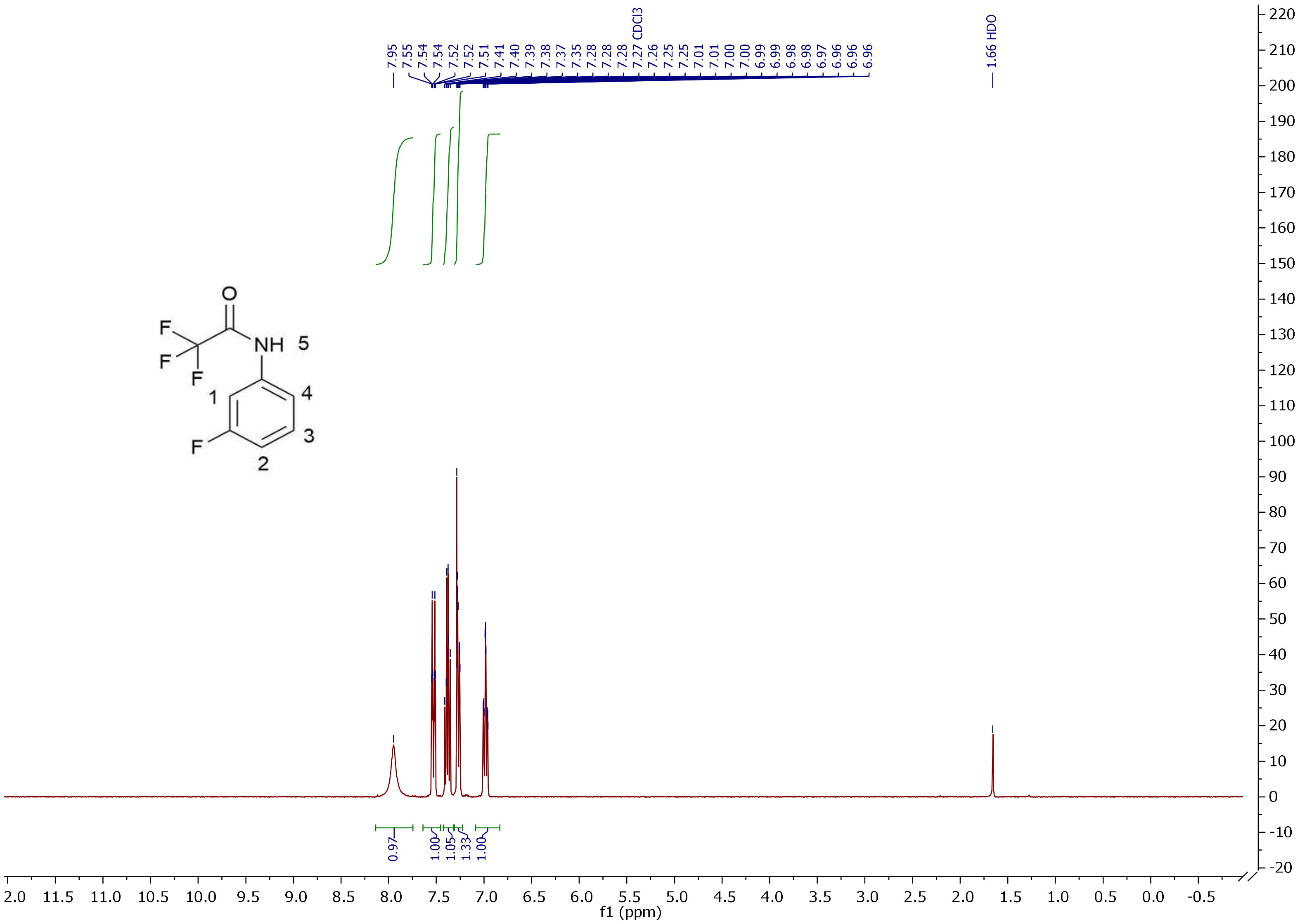

^{13}C NMR (126 MHz, $CDCl_3$) for 2,2,2-trifluoro-*N*-(3-fluorophenyl)acetamide **(1n)**

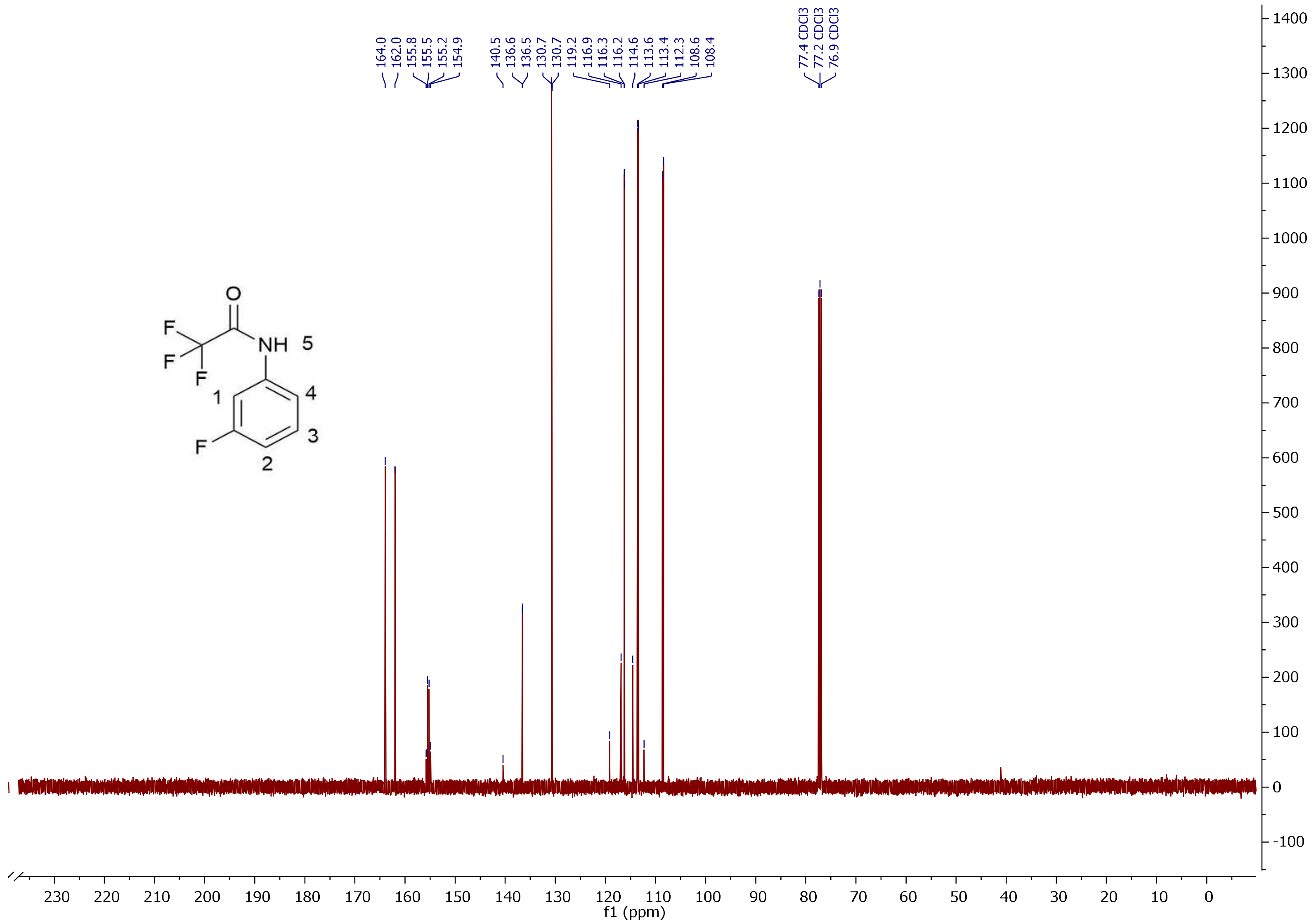

^{19}F NMR (376 MHz, $CDCl_3$) for 2,2,2-trifluoro-*N*-(3-fluorophenyl)acetamide **(1n)**

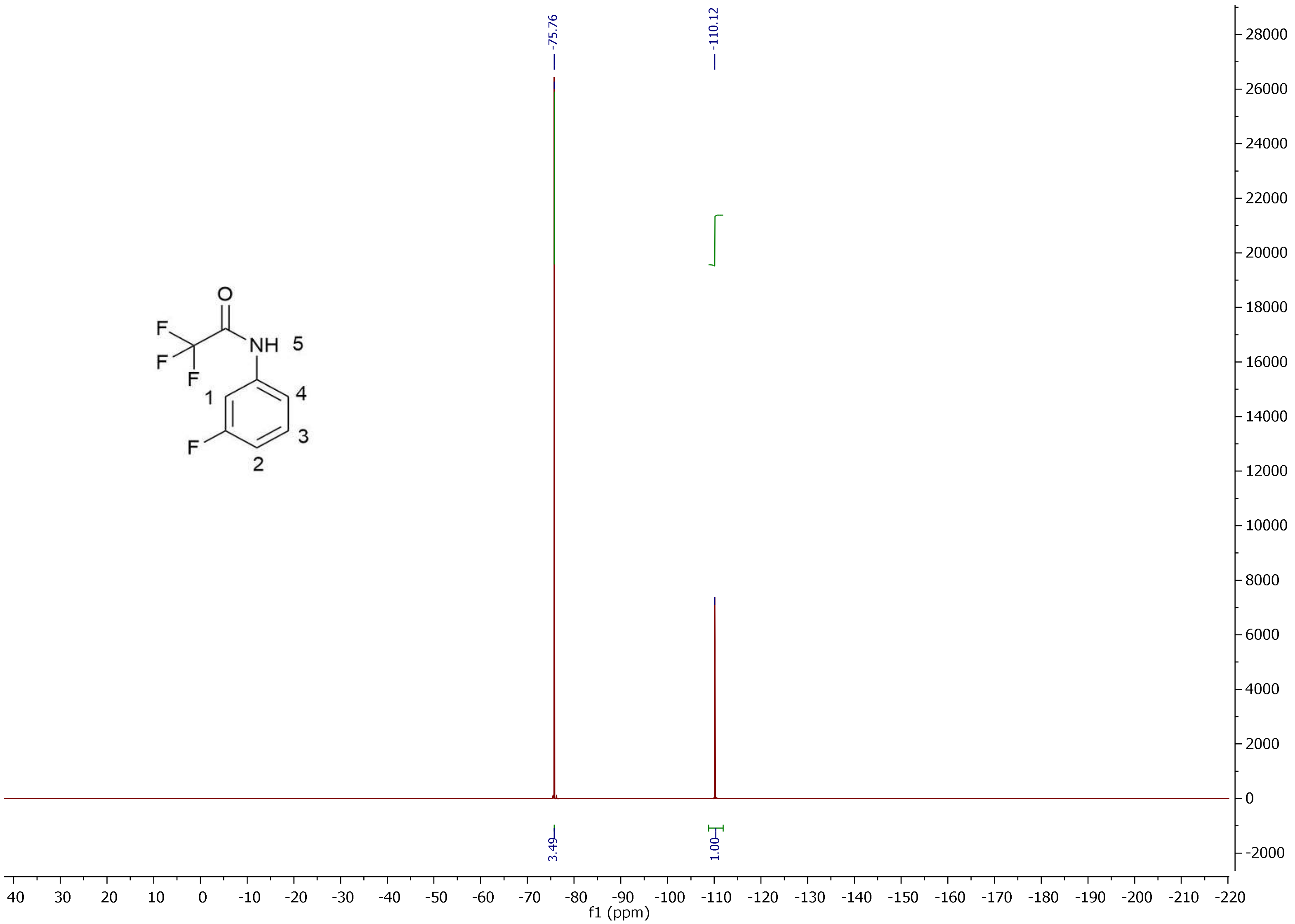

^{1}H NMR (400 MHz, $CDCl_3$) for *N*-(3-chlorophenyl)-2,2,2-trifluoroacetamide **(1o)**

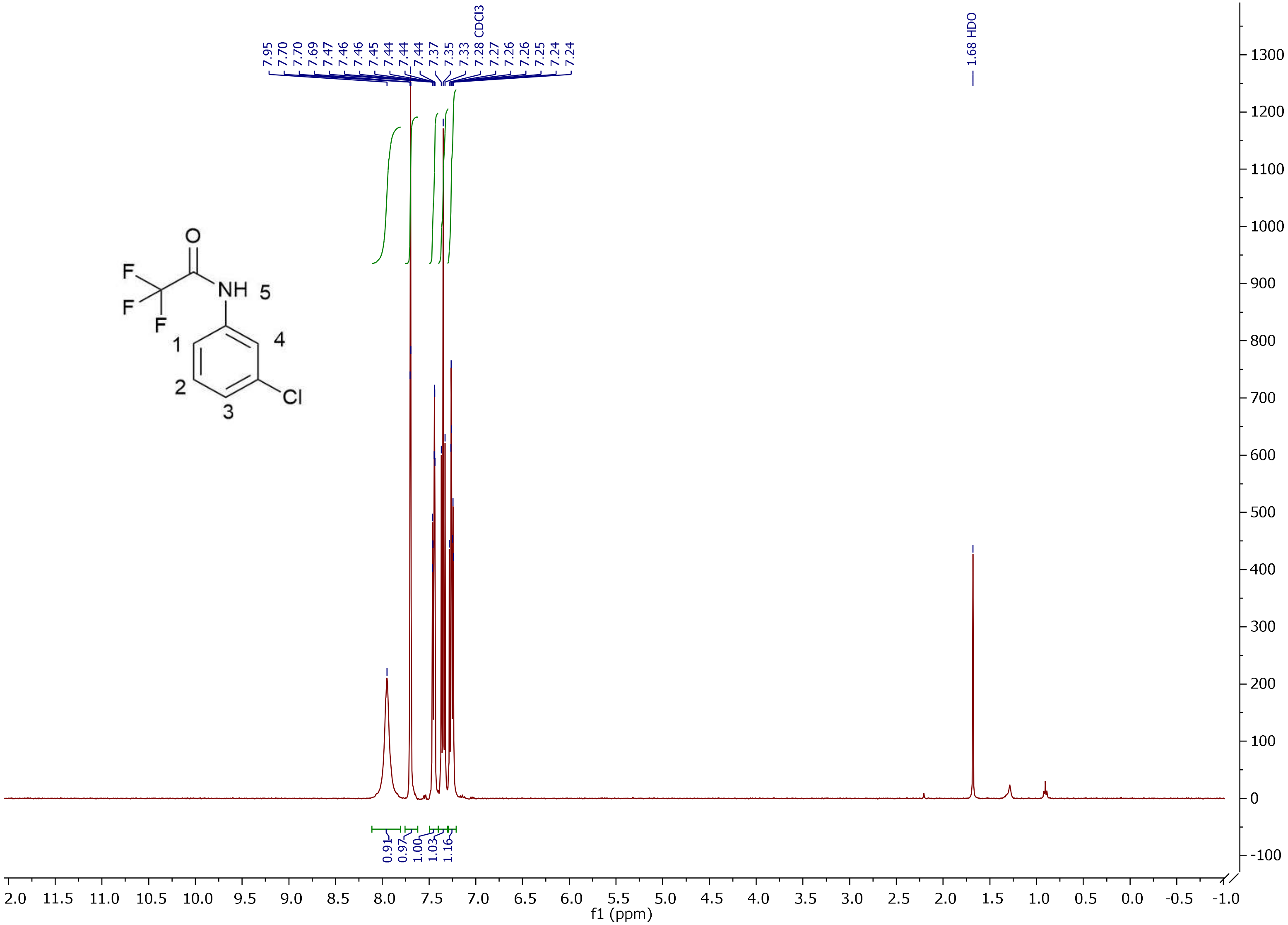

^{13}**C NMR** (126 MHz, $CDCl_3$) for *N*-(3-chlorophenyl)-2,2,2-trifluoroacetamide **(1o)**

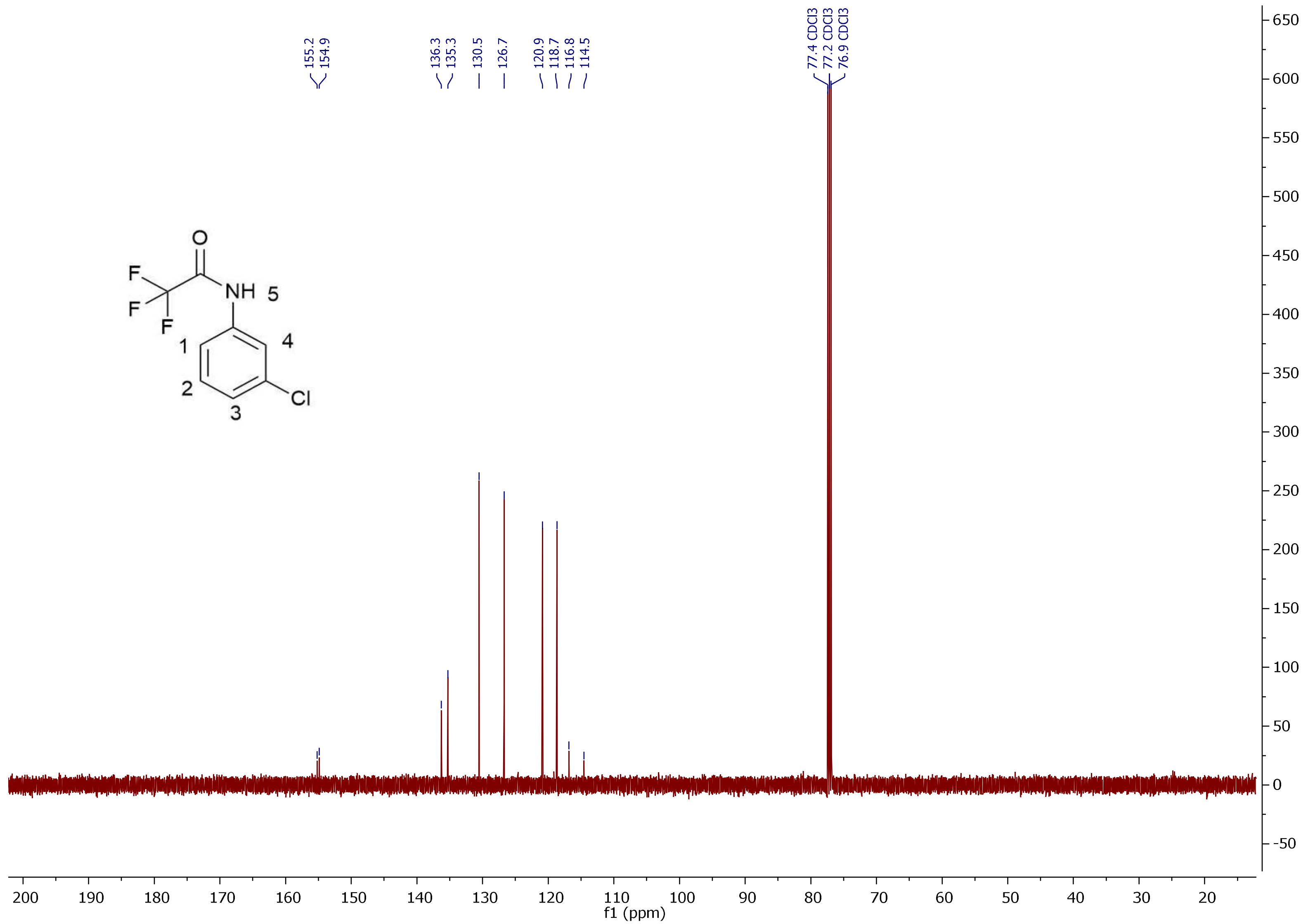

^{19}F NMR (376 MHz, $CDCl_3$) for *N*-(3-chlorophenyl)-2,2,2-trifluoroacetamide **(1o)**

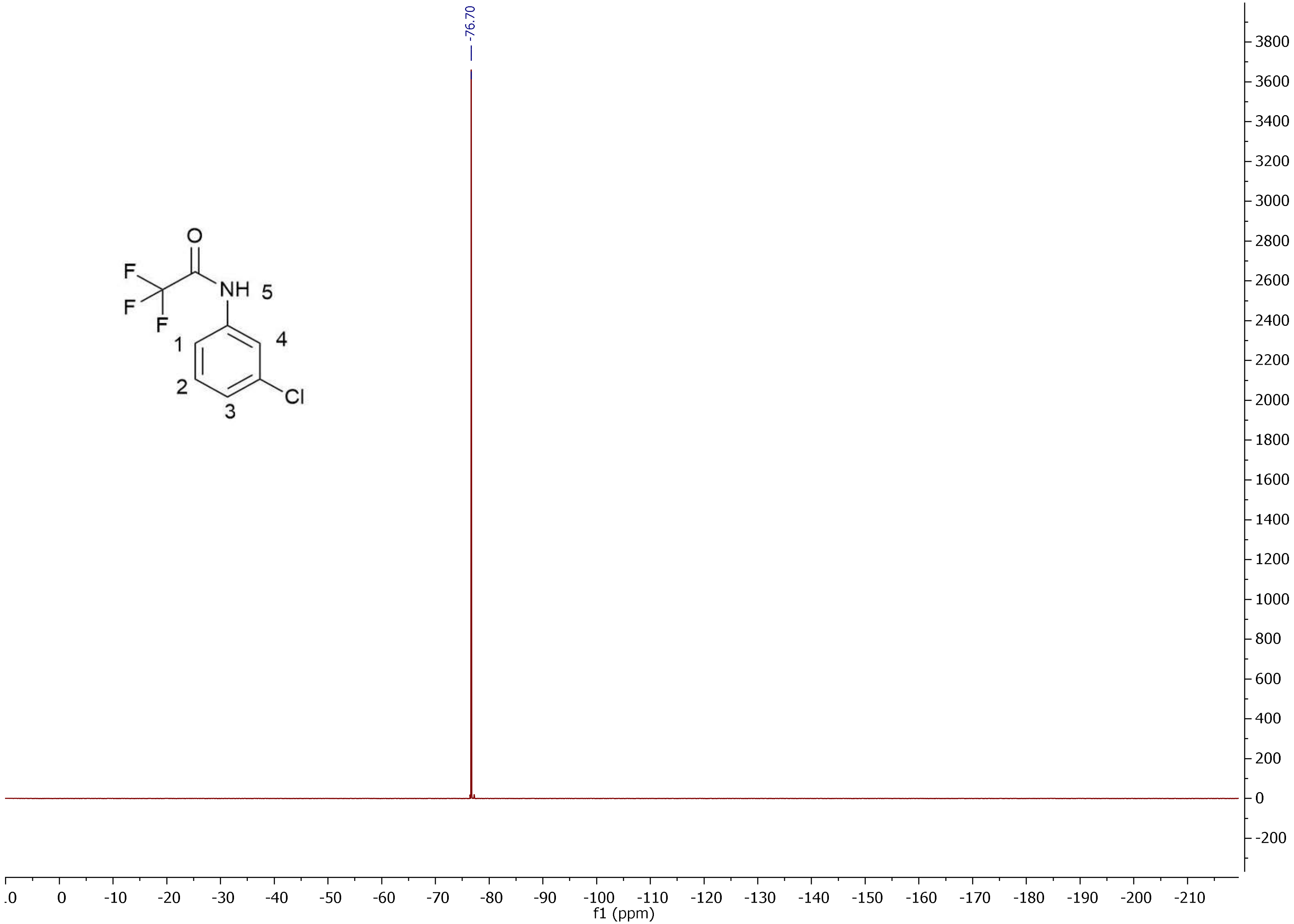

^{1}H NMR (500 MHz, $CDCl_3$) for *N*-(3-bromophenyl)-2,2,2-trifluoroacetamide **(1p)**

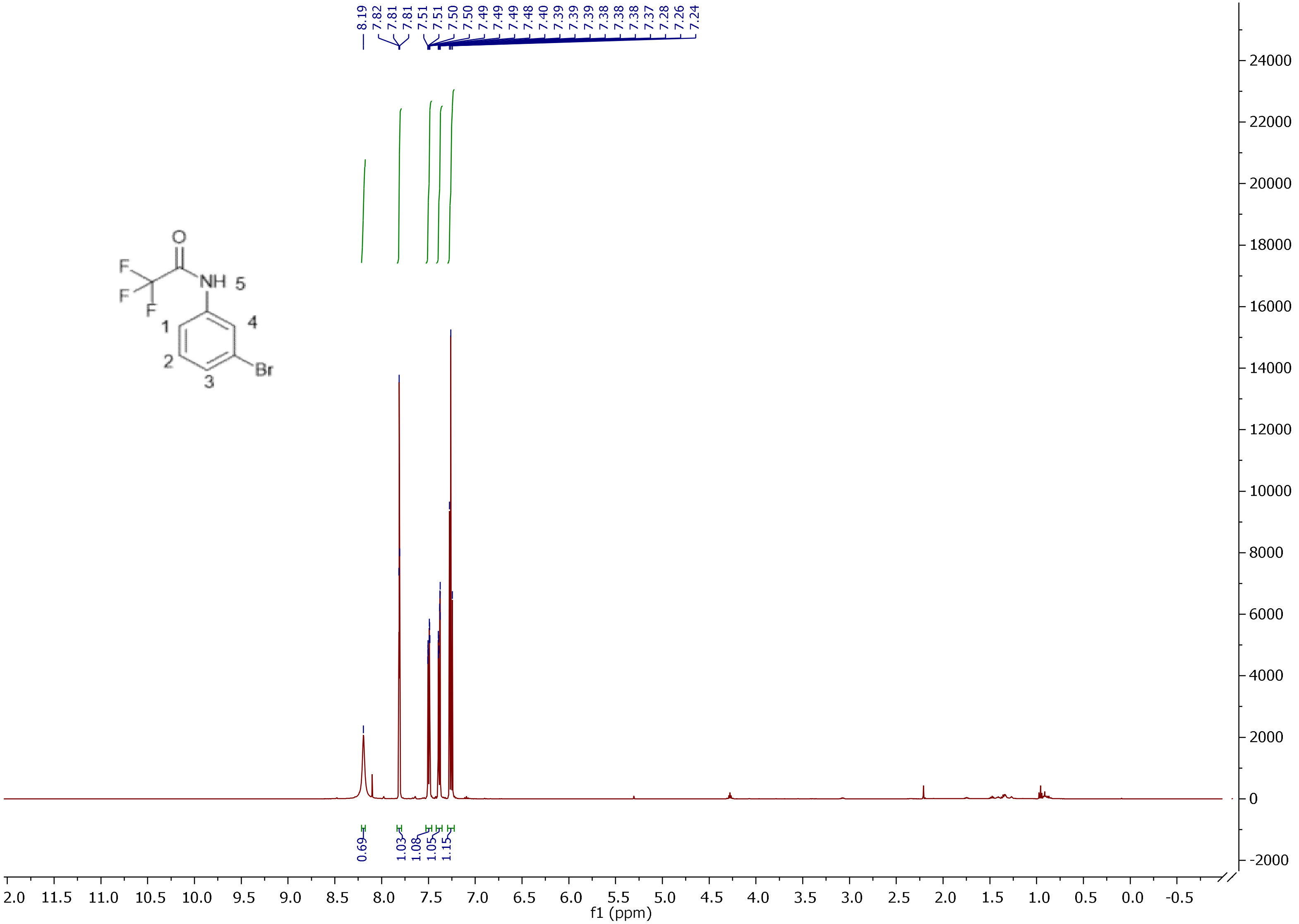

^{13}C NMR (126 MHz, $CDCl_3$) for *N*-(3-bromophenyl)-2,2,2-trifluoroacetamide **(1p)**

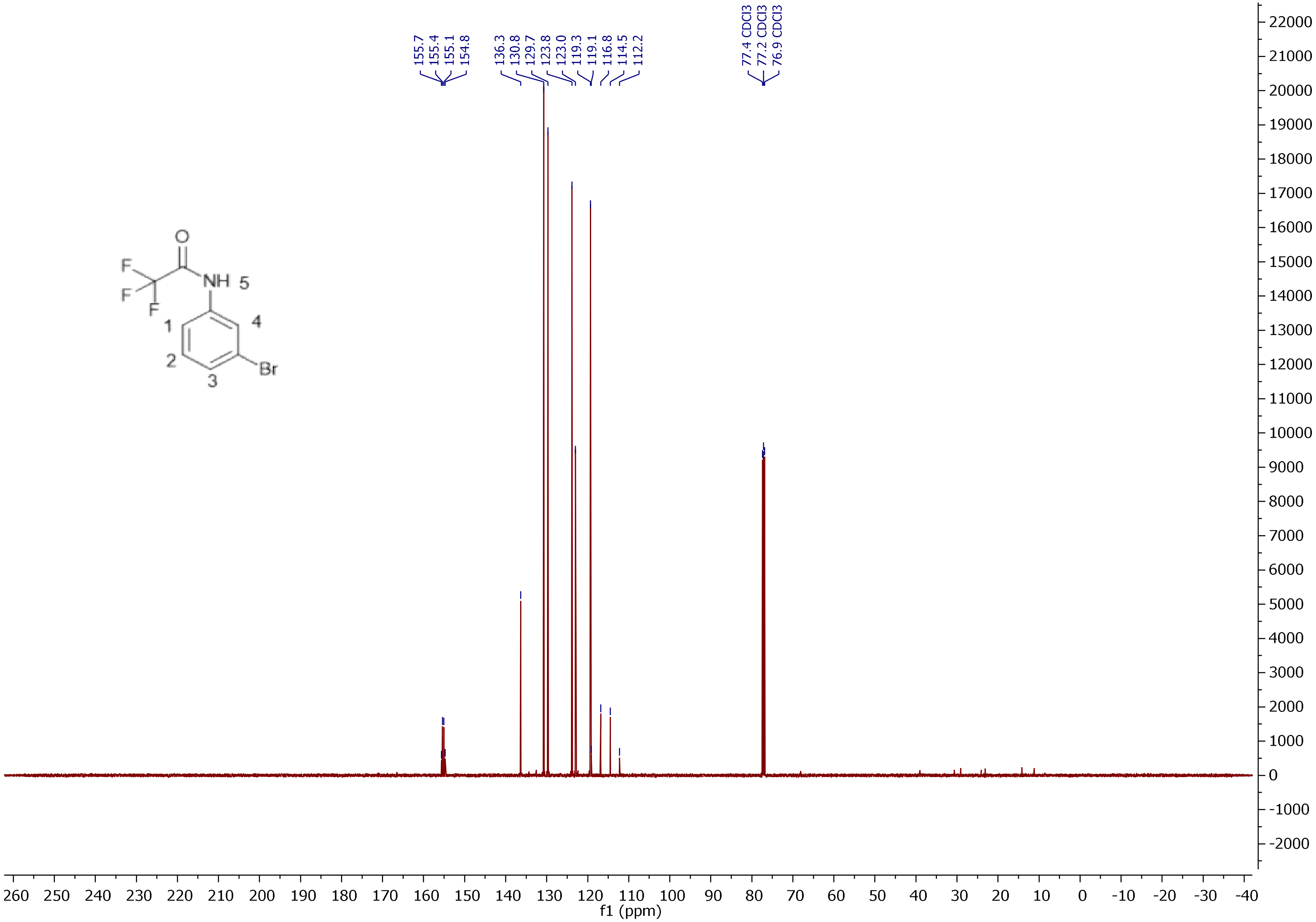

^{19}F NMR (376 MHz, $CDCl_3$) for *N*-(3-bromophenyl)-2,2,2-trifluoroacetamide **(1p)**

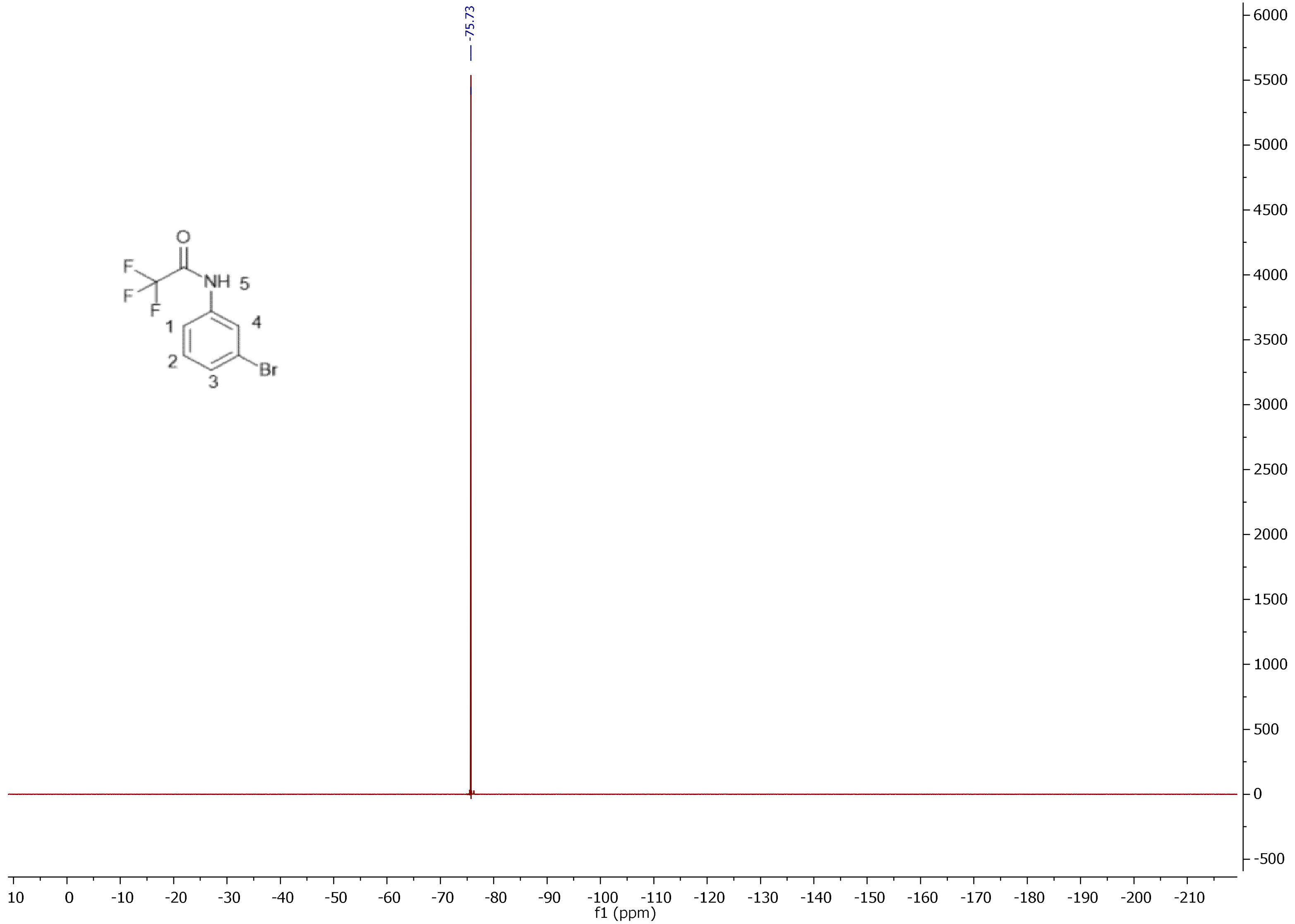

^{1}H NMR (400 MHz, $CDCl_3$) for *N*-(3,5-dichlorophenyl)-2,2,2-trifluoroacetamide **(1q)**

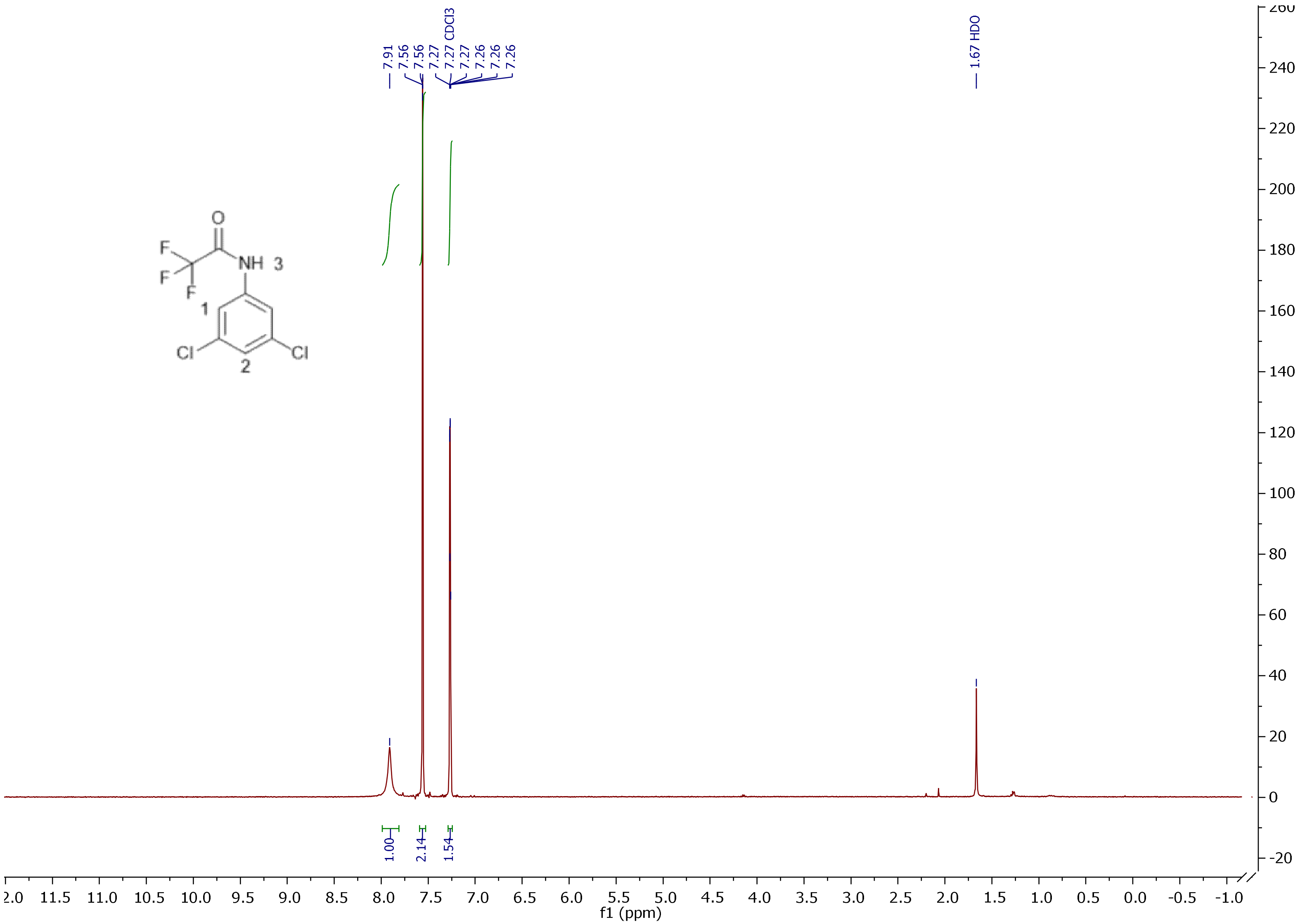

^{13}C NMR (101 MHz, $CDCl_3$) for *N*-(3,5-dichlorophenyl)-2,2,2-trifluoroacetamide **(1q)**

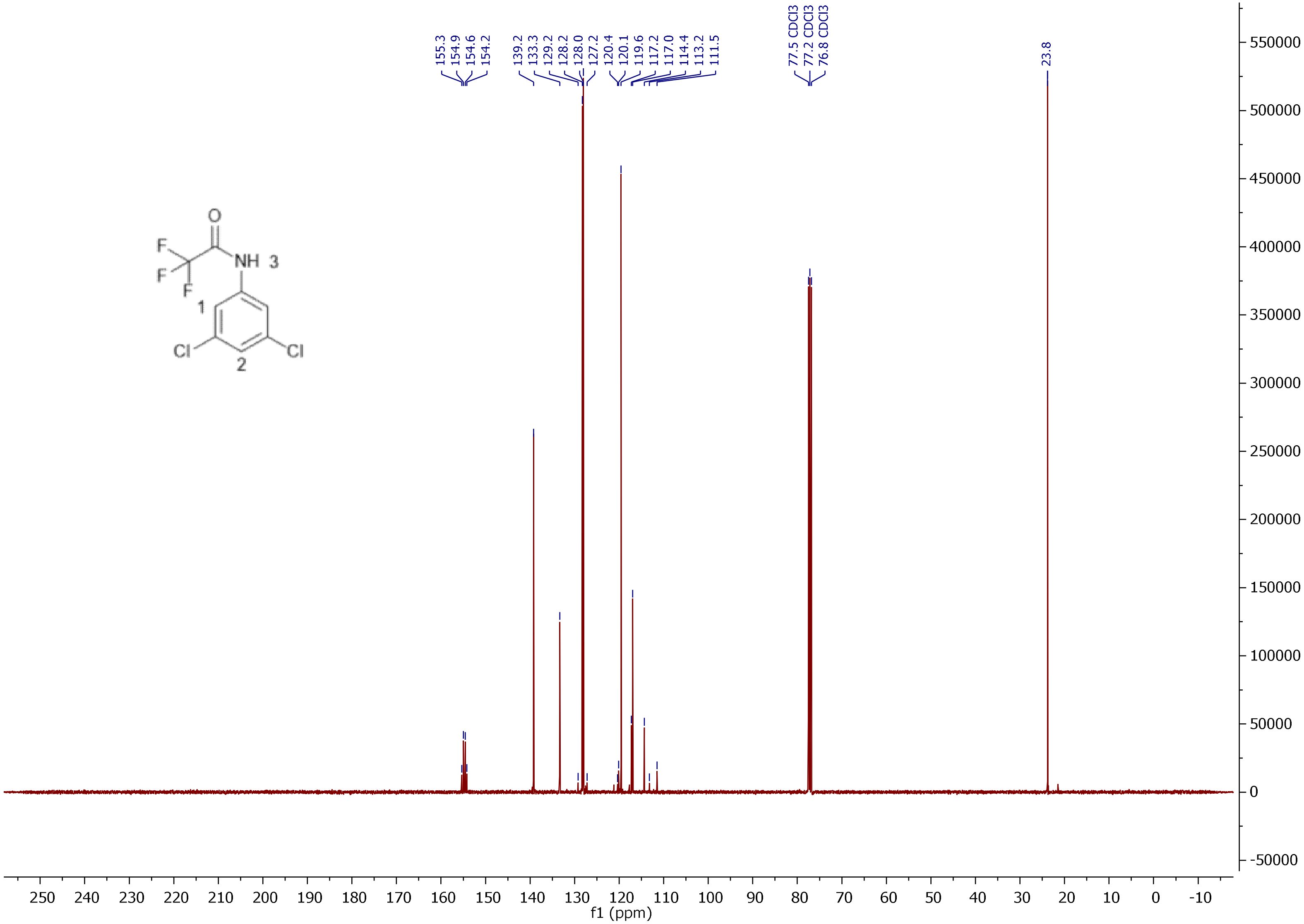

^{19}F NMR (376 MHz, $CDCl_3$) for *N*-(3,5-dichlorophenyl)-2,2,2-trifluoroacetamide **(1q)**

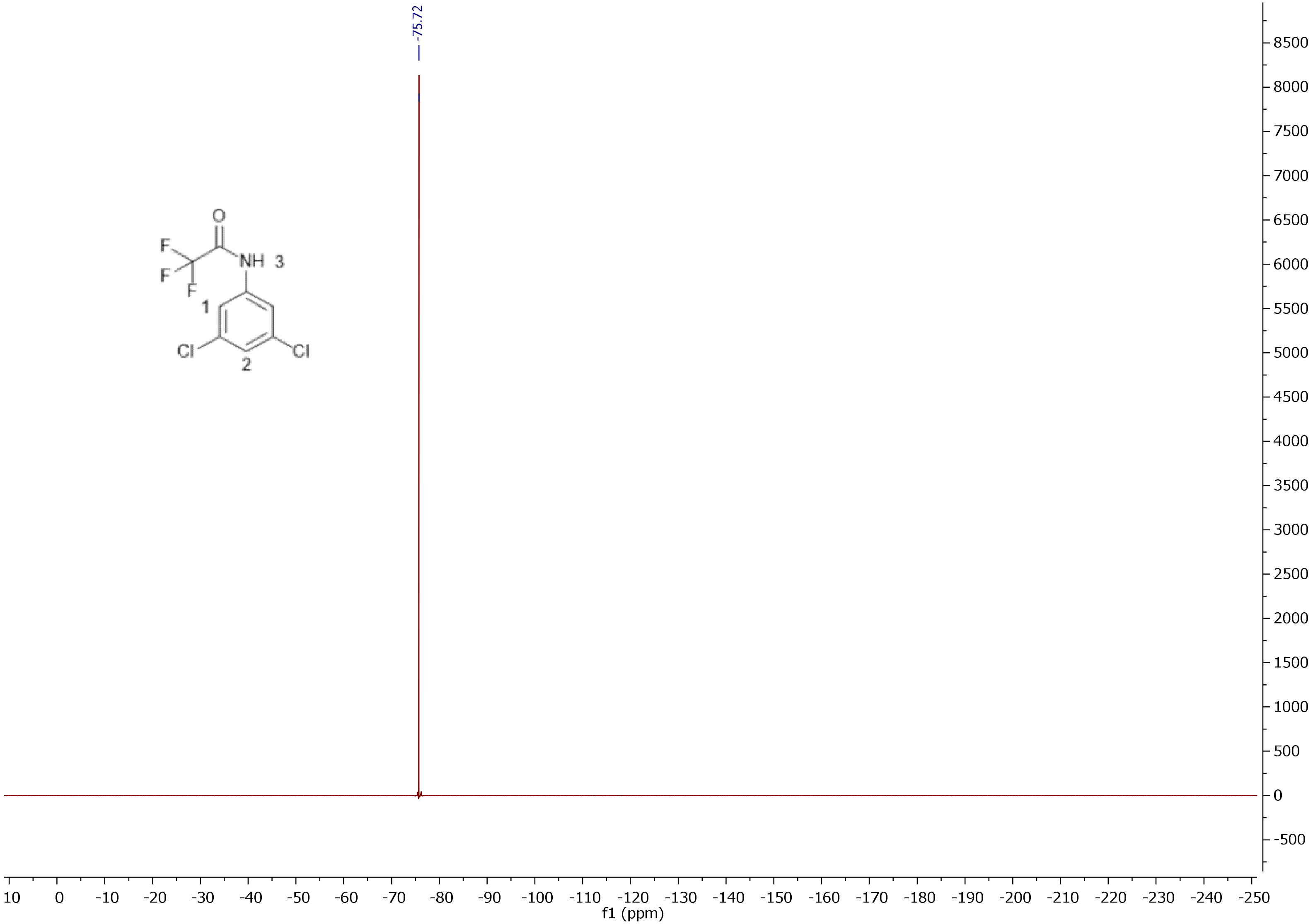

¹H NMR (400 MHz, $CDCl_3$) for *N*-(2-bromo-3-methylphenyl)-2,2,2-trifluoroacetamide **(1r)**

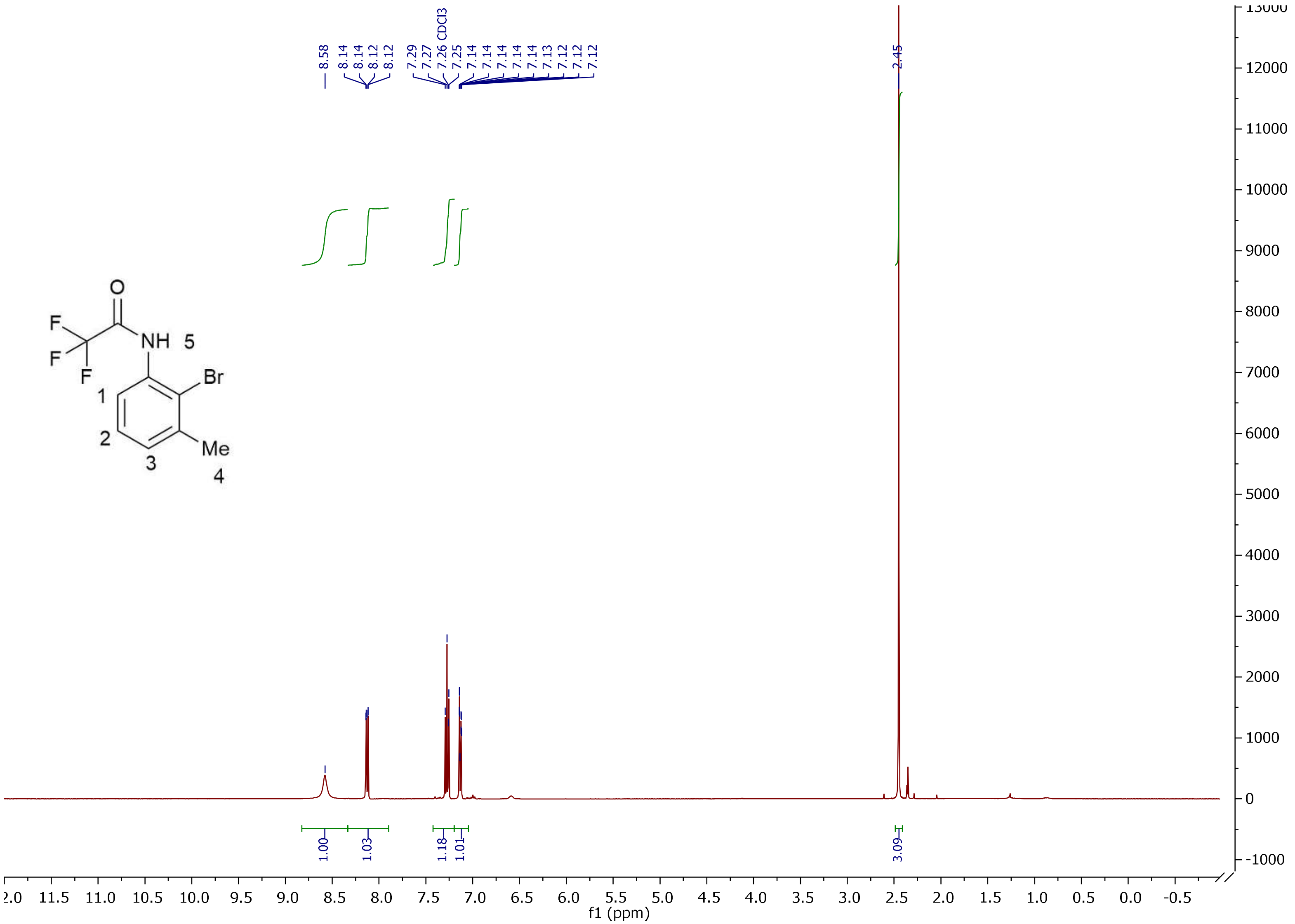

^{13}C NMR (101 MHz, $CDCl_3$) for *N*-(2-bromo-3-methylphenyl)-2,2,2-trifluoroacetamide **(1r)**

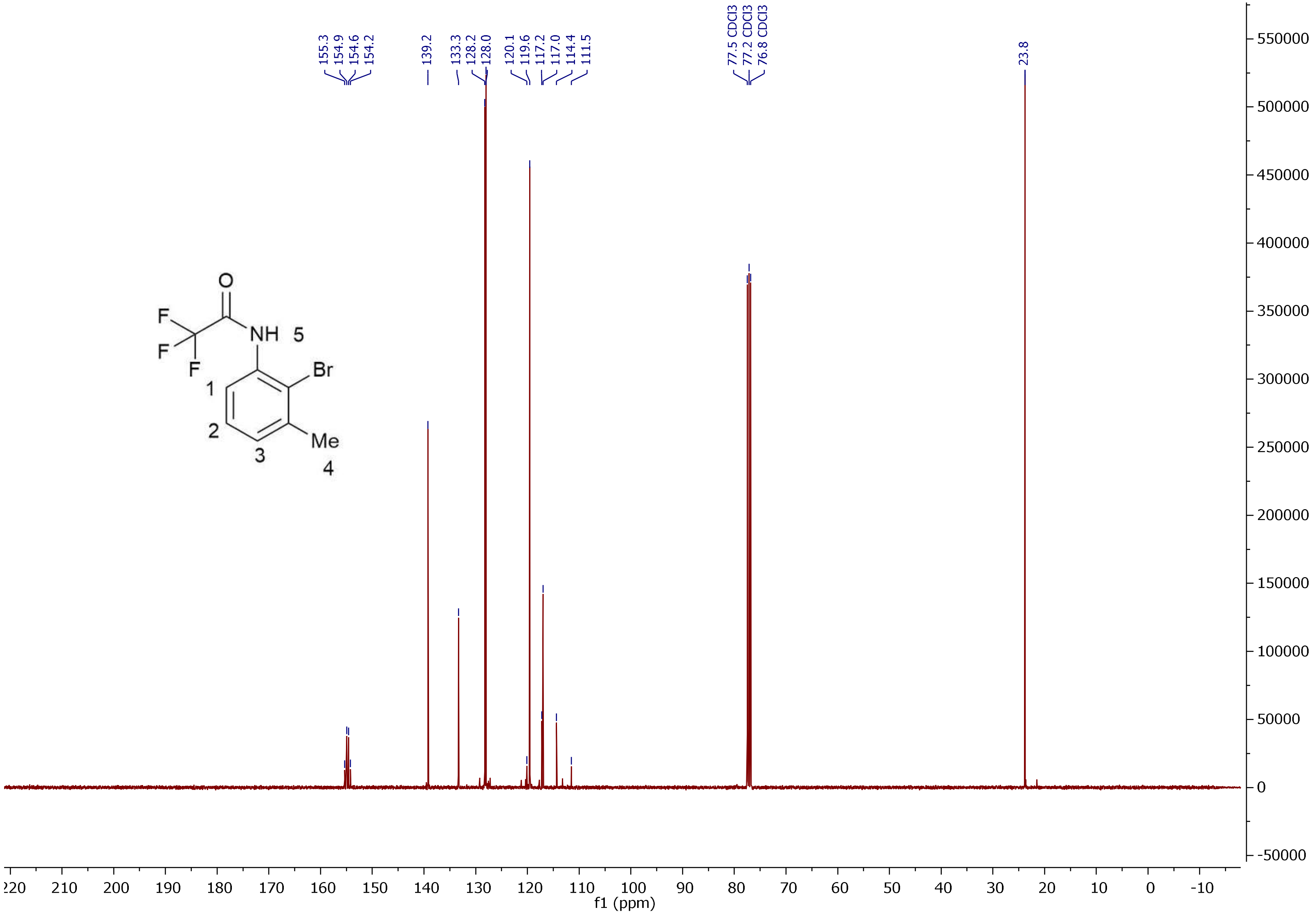

^{19}F NMR (376 MHz, $CDCl_3$) for *N*-(2-bromo-3-methylphenyl)-2,2,2-trifluoroacetamide **(1r)**

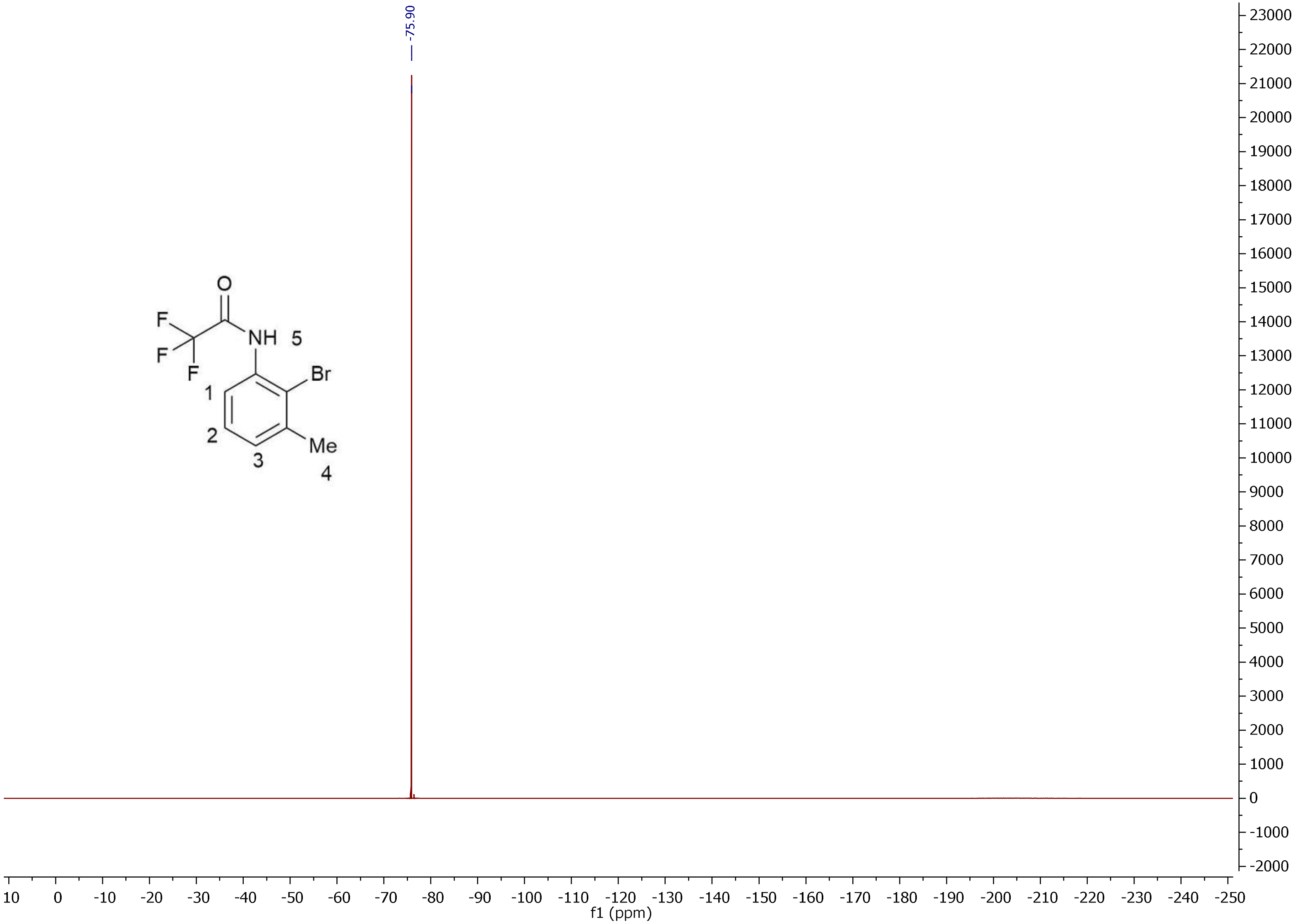

^{1}H NMR (500 MHz, $CDCl_3$) for 2,2,2-trifluoro-*N*-(3-(trifluoromethyl)phenyl)acetamide **(1s)**

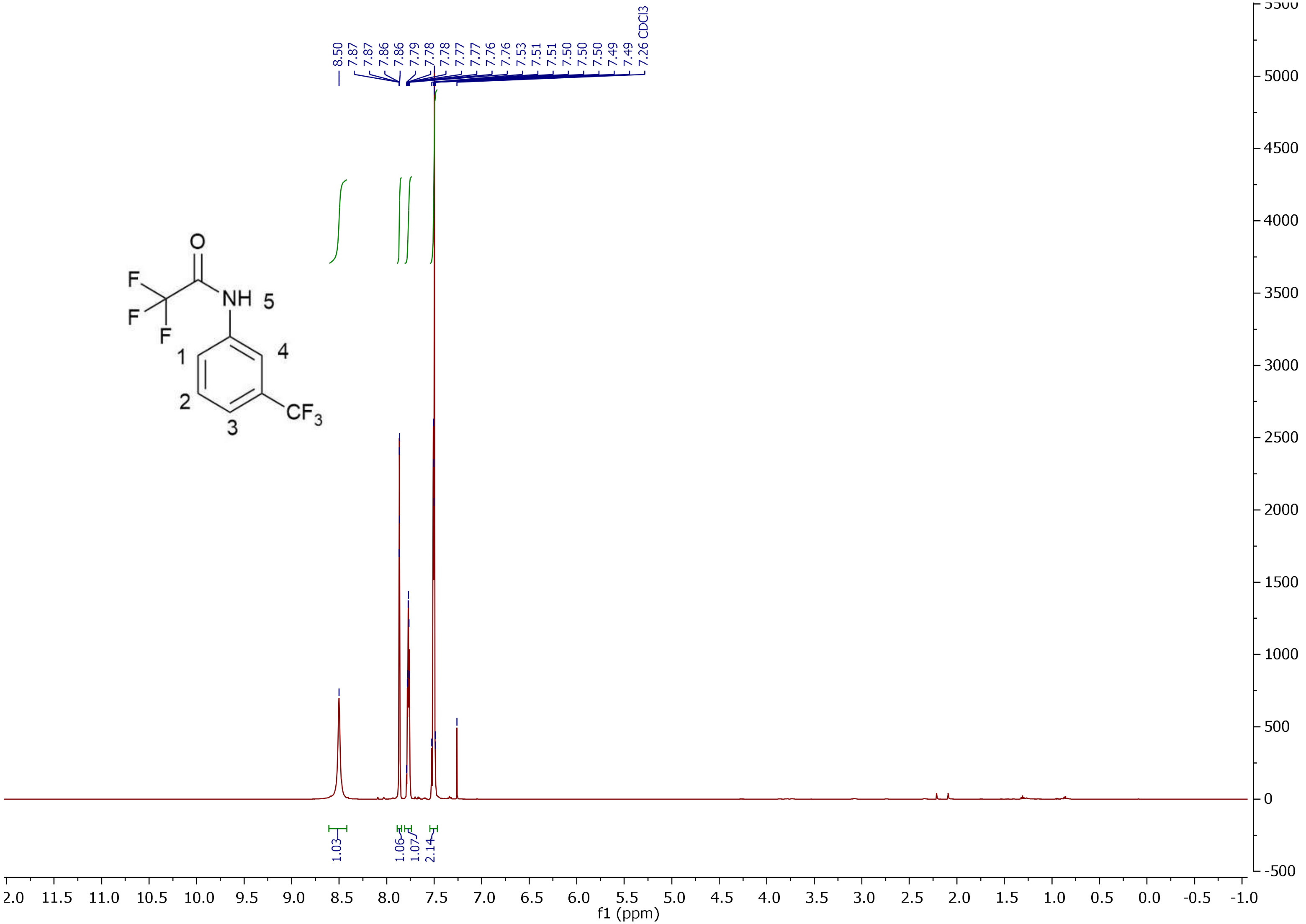

^{13}C NMR (126 MHz, $CDCl_3$) for 2,2,2-trifluoro-*N*-(3-(trifluoromethyl)phenyl)acetamide **(1s)**

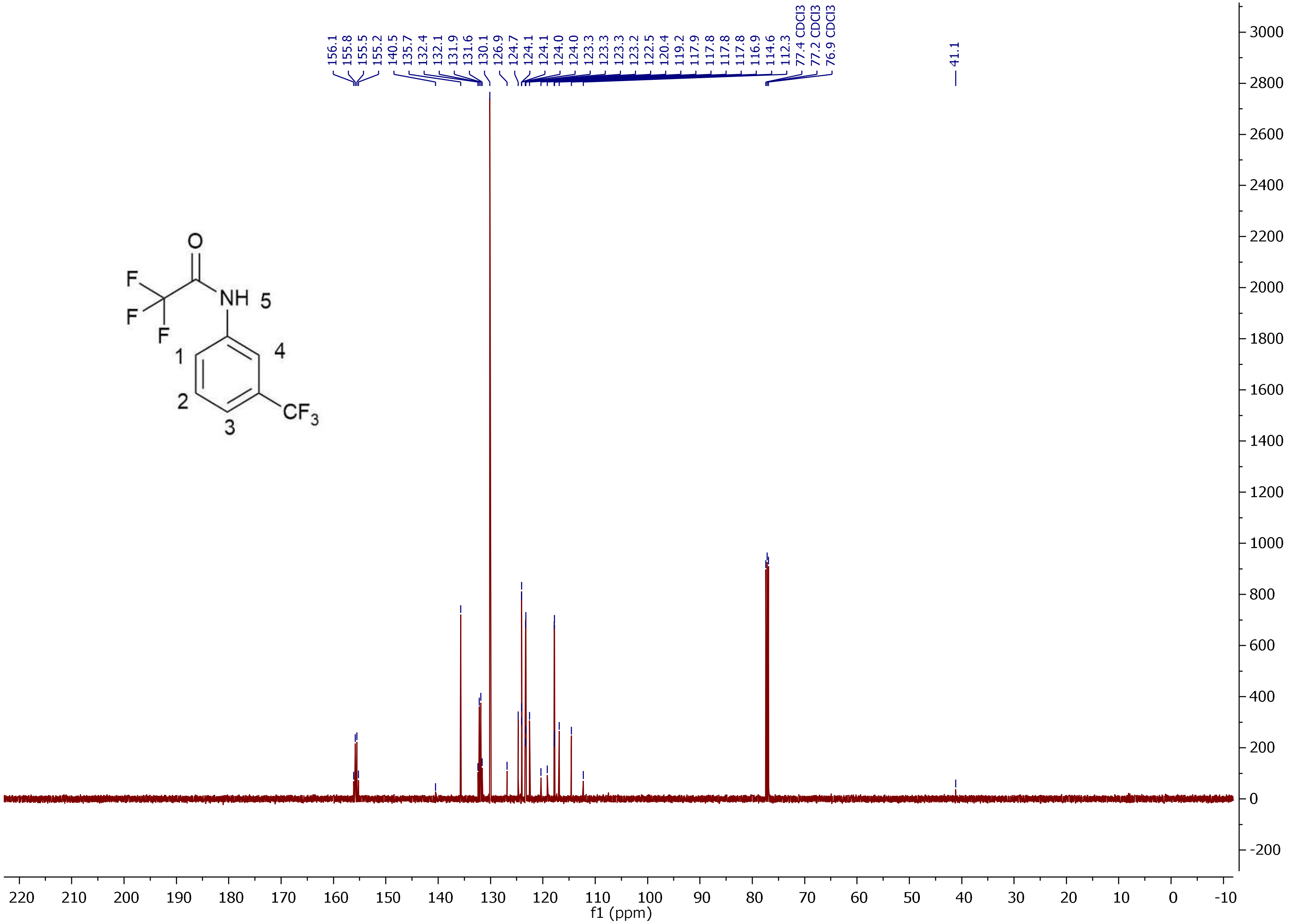

^{19}F NMR (376 MHz, $CDCl_3$) for 2,2,2-trifluoro-*N*-(3-(trifluoromethyl)phenyl)acetamide **(1s)**

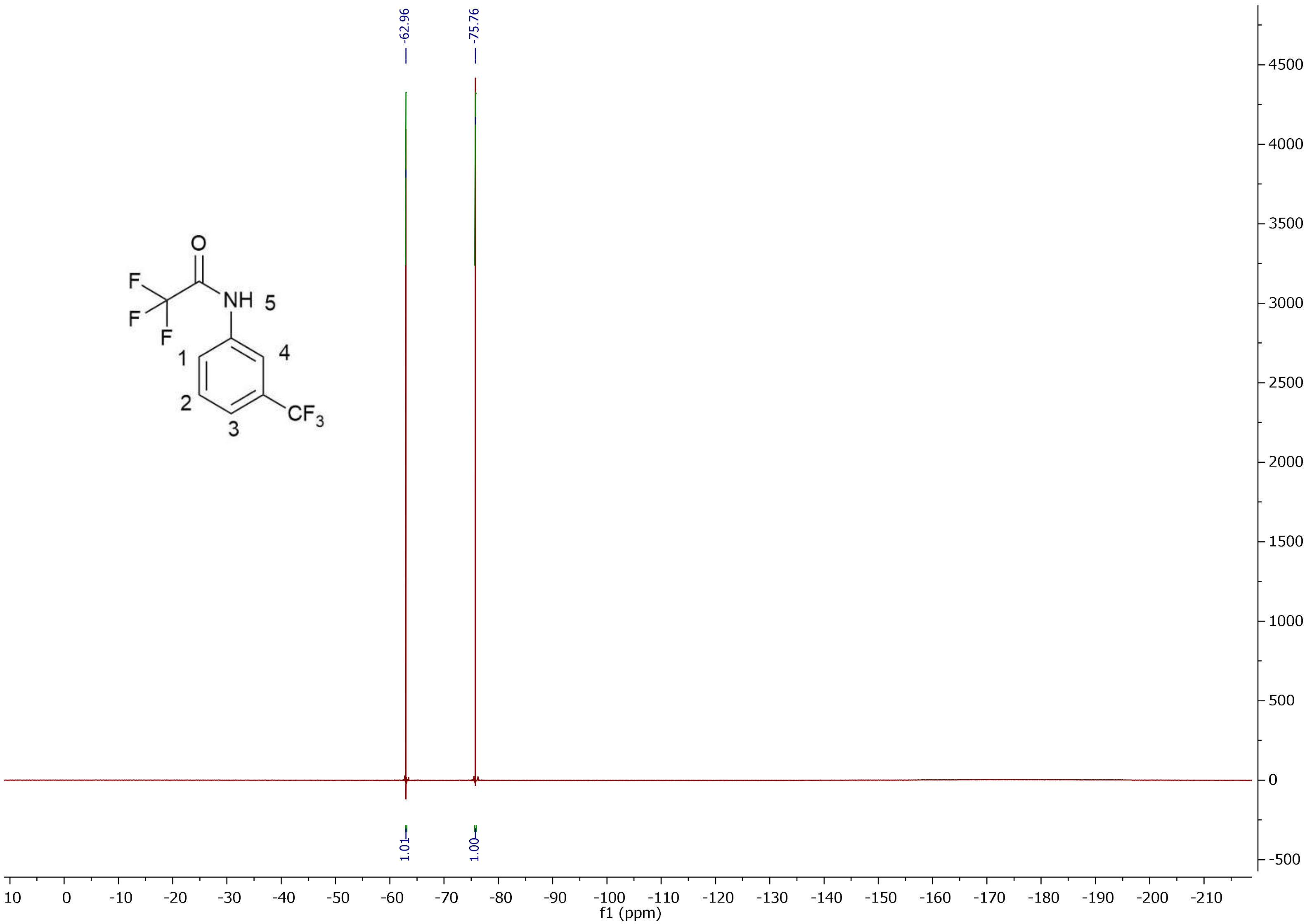

^{1}H NMR (400 MHz, $CDCl_3$) for *N*-(2-benzoylphenyl)-2,2,2-trifluoroacetamide **(1t)**

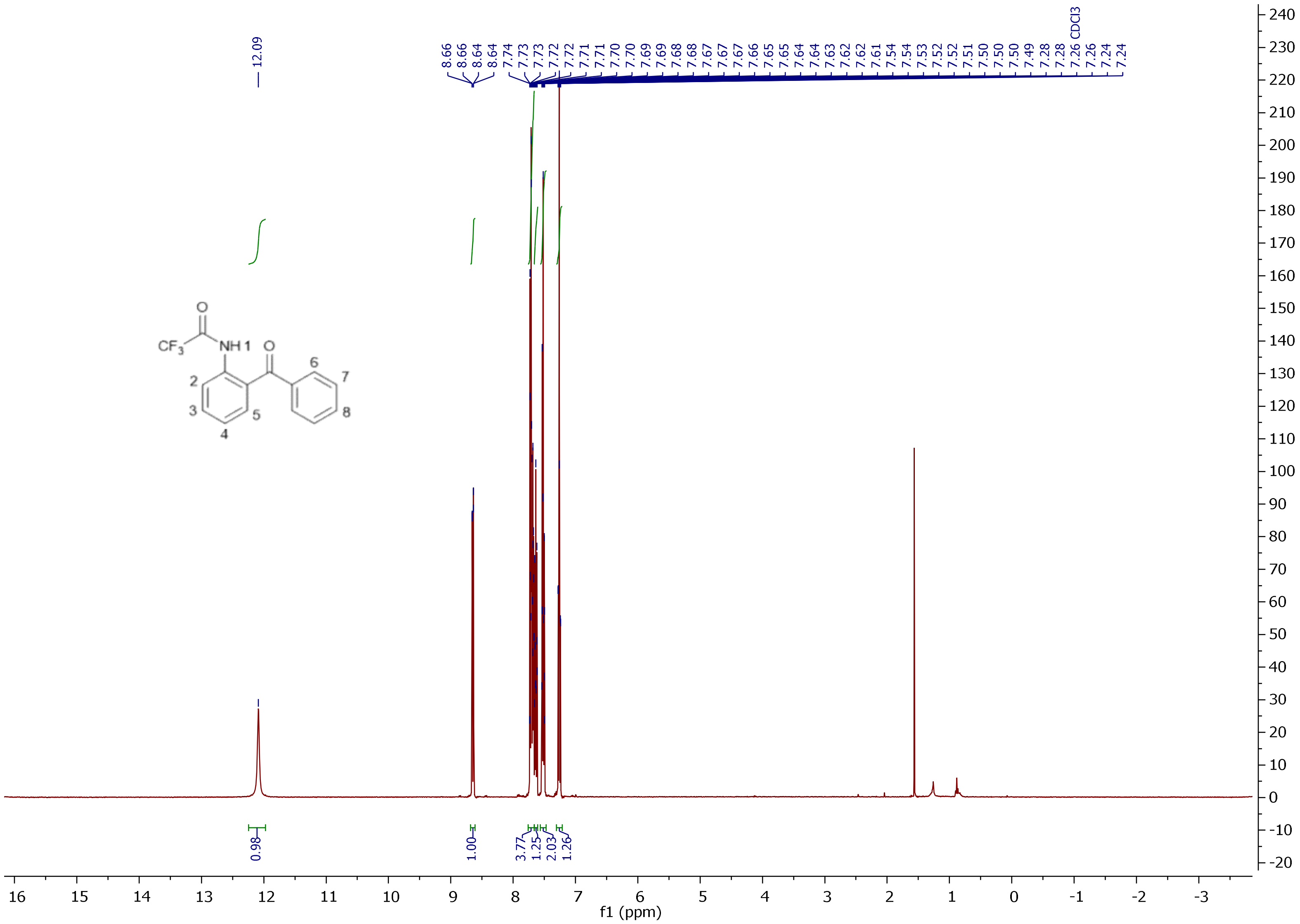

^{13}C NMR (126 MHz, $CDCl_3$) for *N*-(2-benzoylphenyl)-2,2,2-trifluoroacetamide **(1t)**

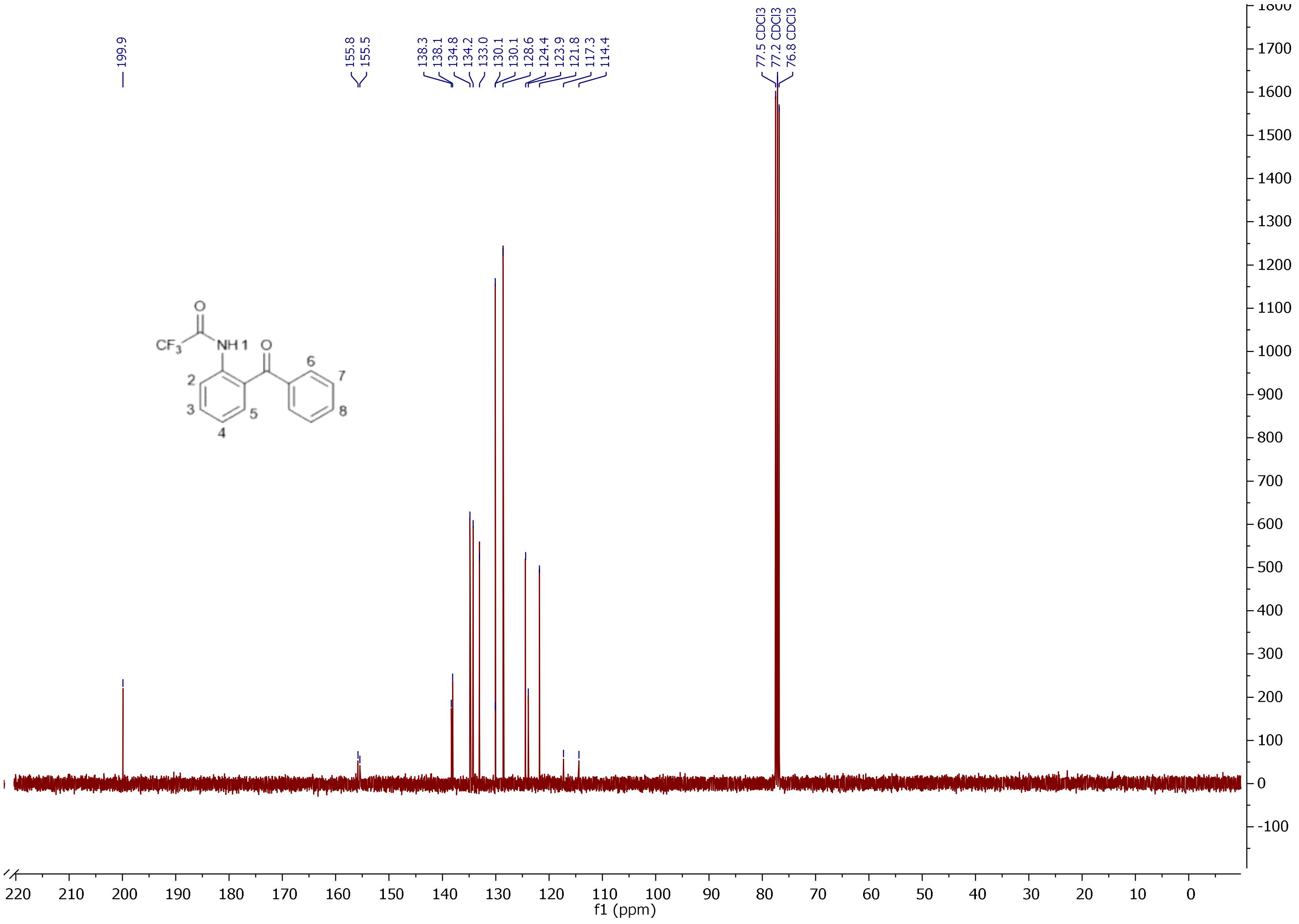

^{19}F NMR (376 MHz, $CDCl_3$) for *N*-(2-benzoylphenyl)-2,2,2-trifluoroacetamide **(1t)**

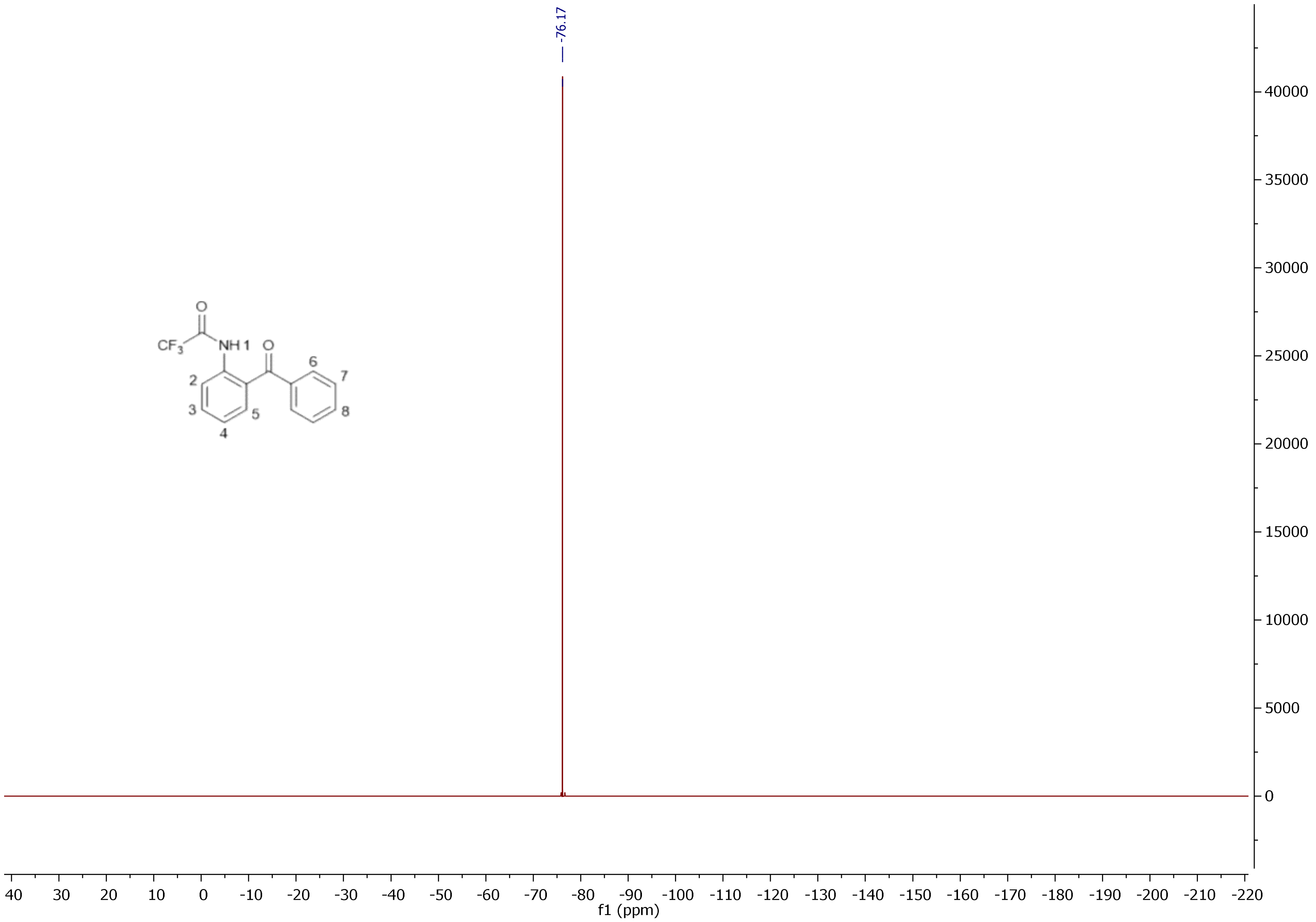

^{1}H NMR (700 MHz, $CDCl_3$) for methyl 3-(2,2,2-trifluoroacetamido)benzoate **(1u)**

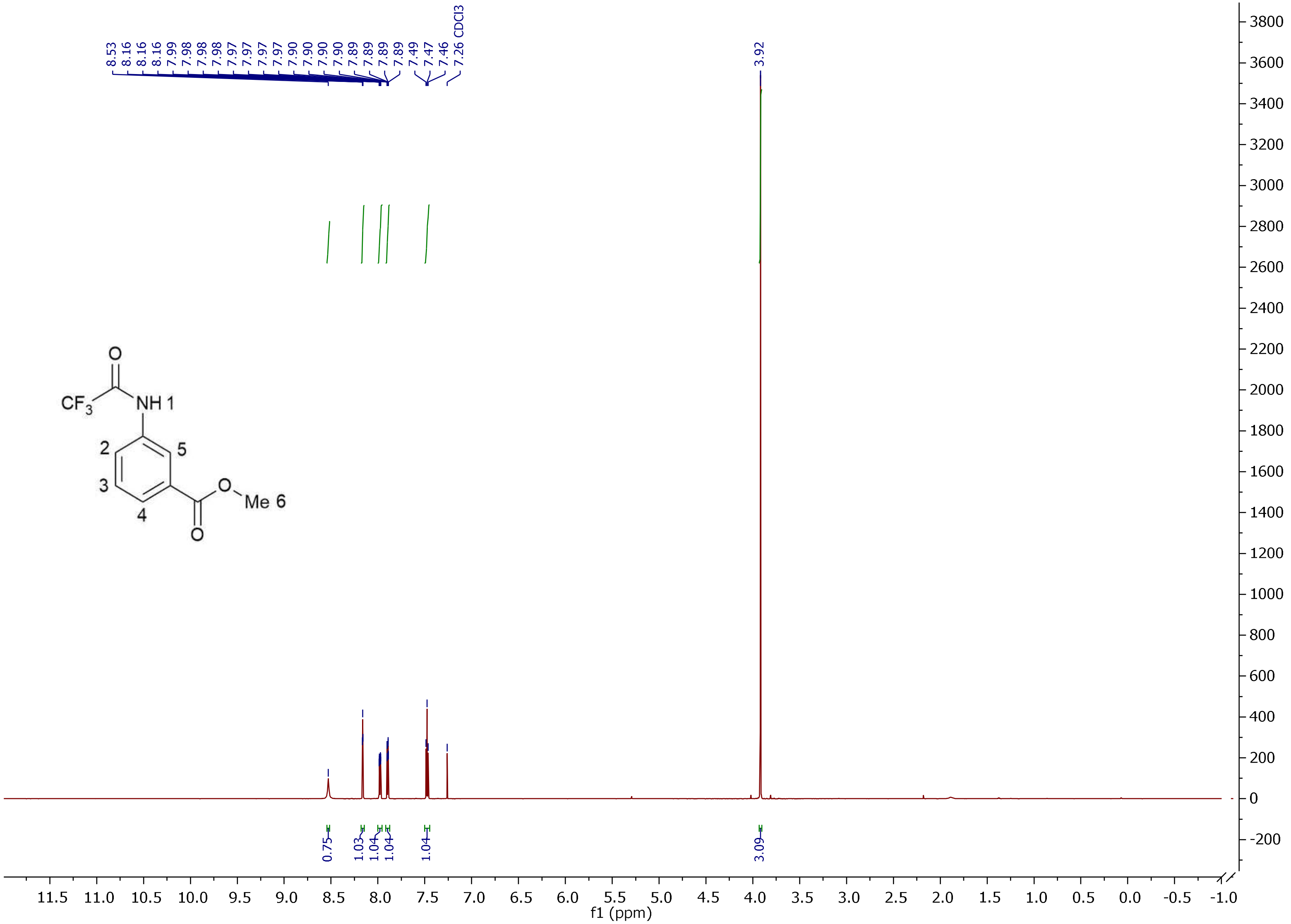

^{13}C NMR (126 MHz, $CDCl_3$) for methyl 3-(2,2,2-trifluoroacetamido)benzoate **(1u)**

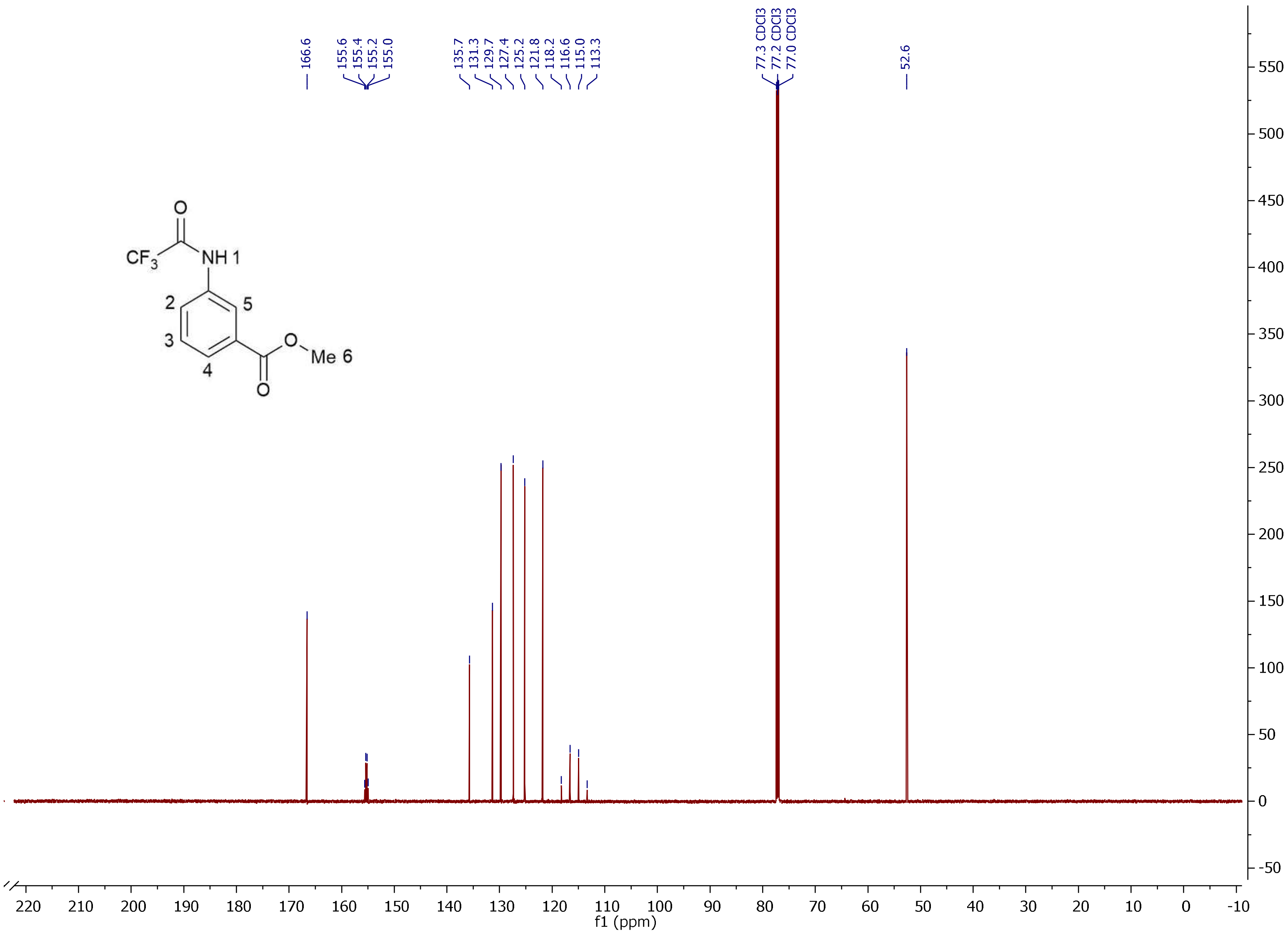

^{19}F NMR (376 MHz, $CDCl_3$) for methyl 3-(2,2,2-trifluoroacetamido)benzoate **(1u)**

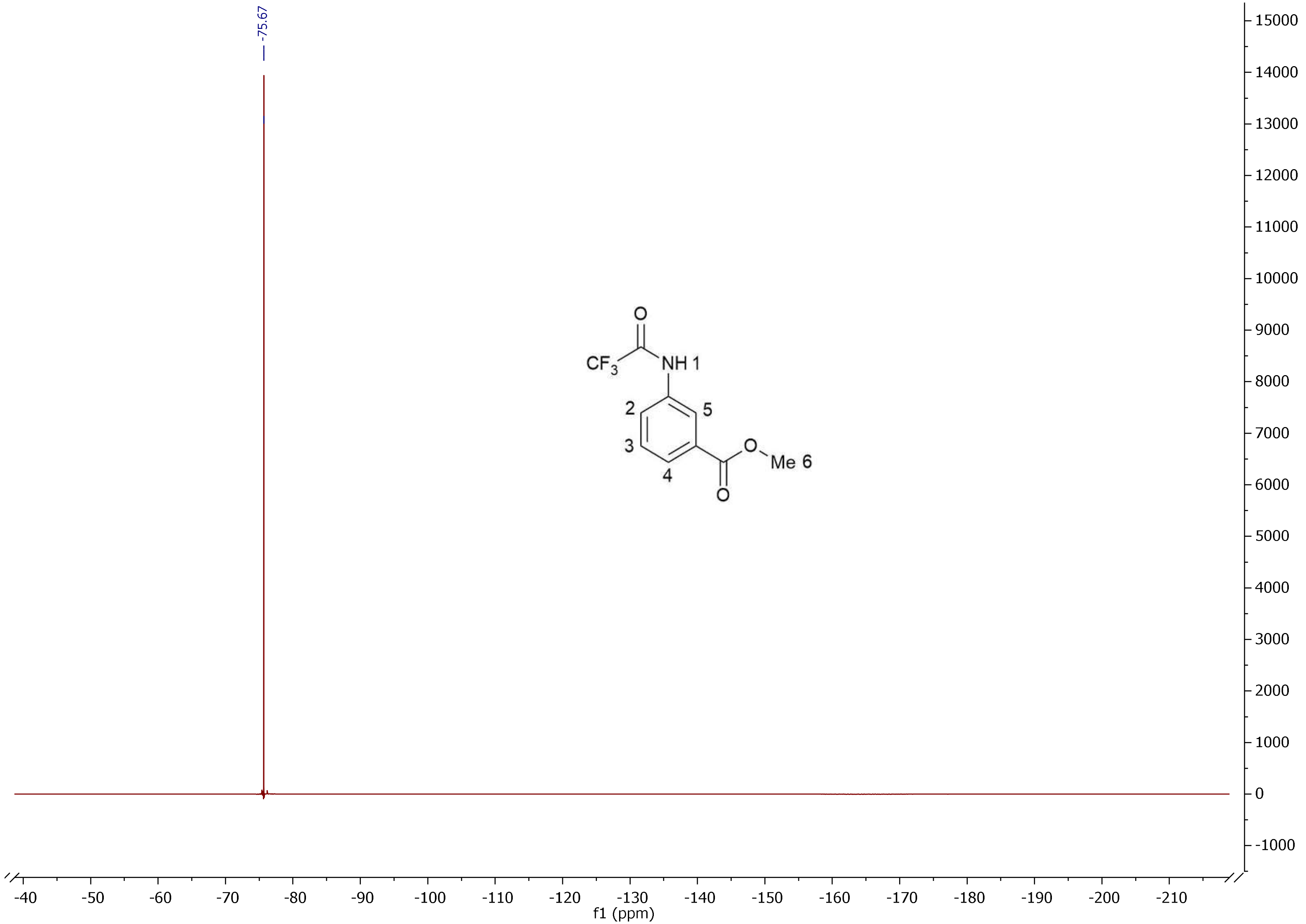

^{1}H NMR (400 MHz, $CDCl_3$) for *N*-(3-cyanophenyl)-2,2,2-trifluoroacetamide **(1v)**

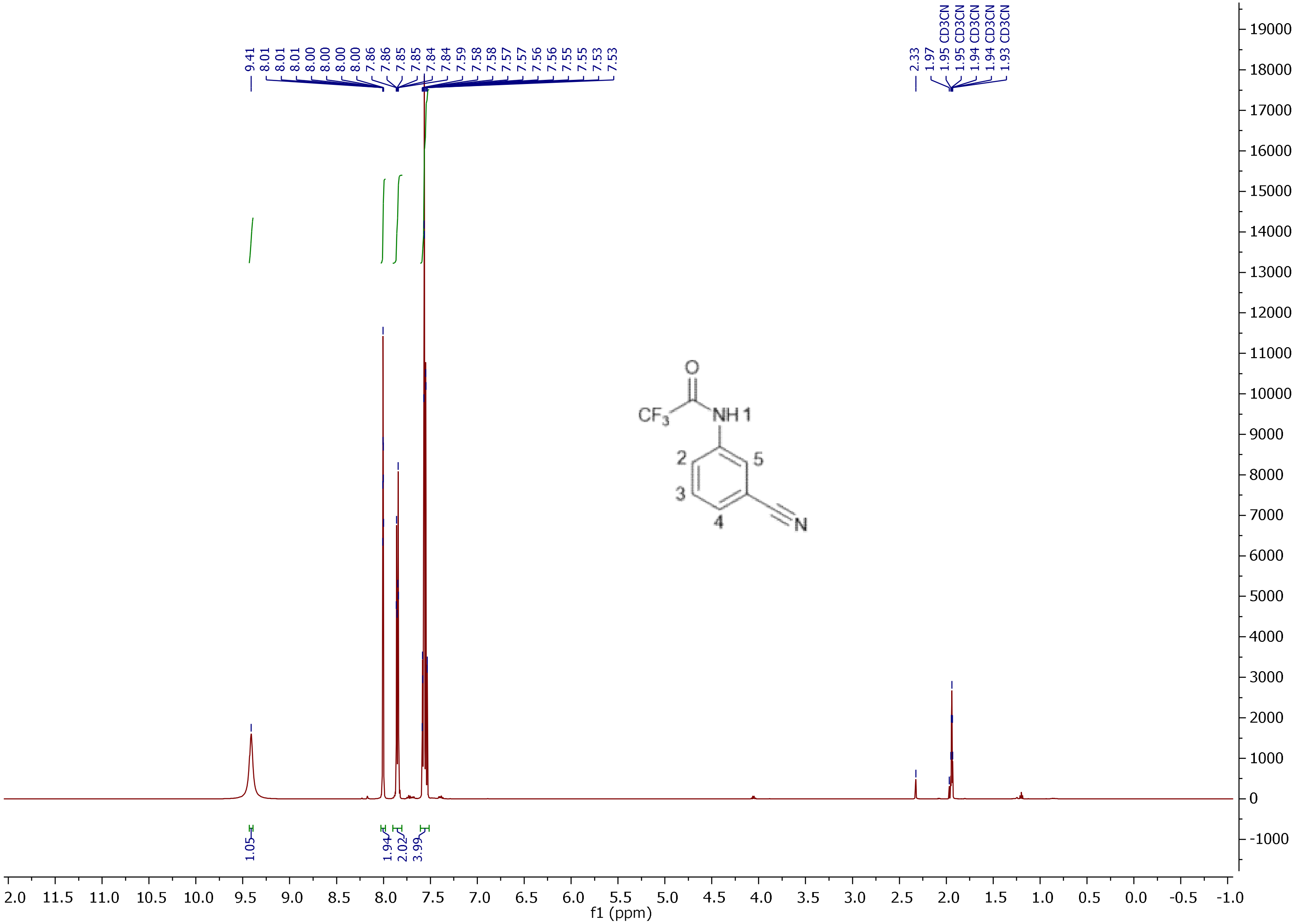

^{13}C NMR (126 MHz, $CDCl_3$) for *N*-(3-cyanophenyl)-2,2,2-trifluoroacetamide **(1v)**

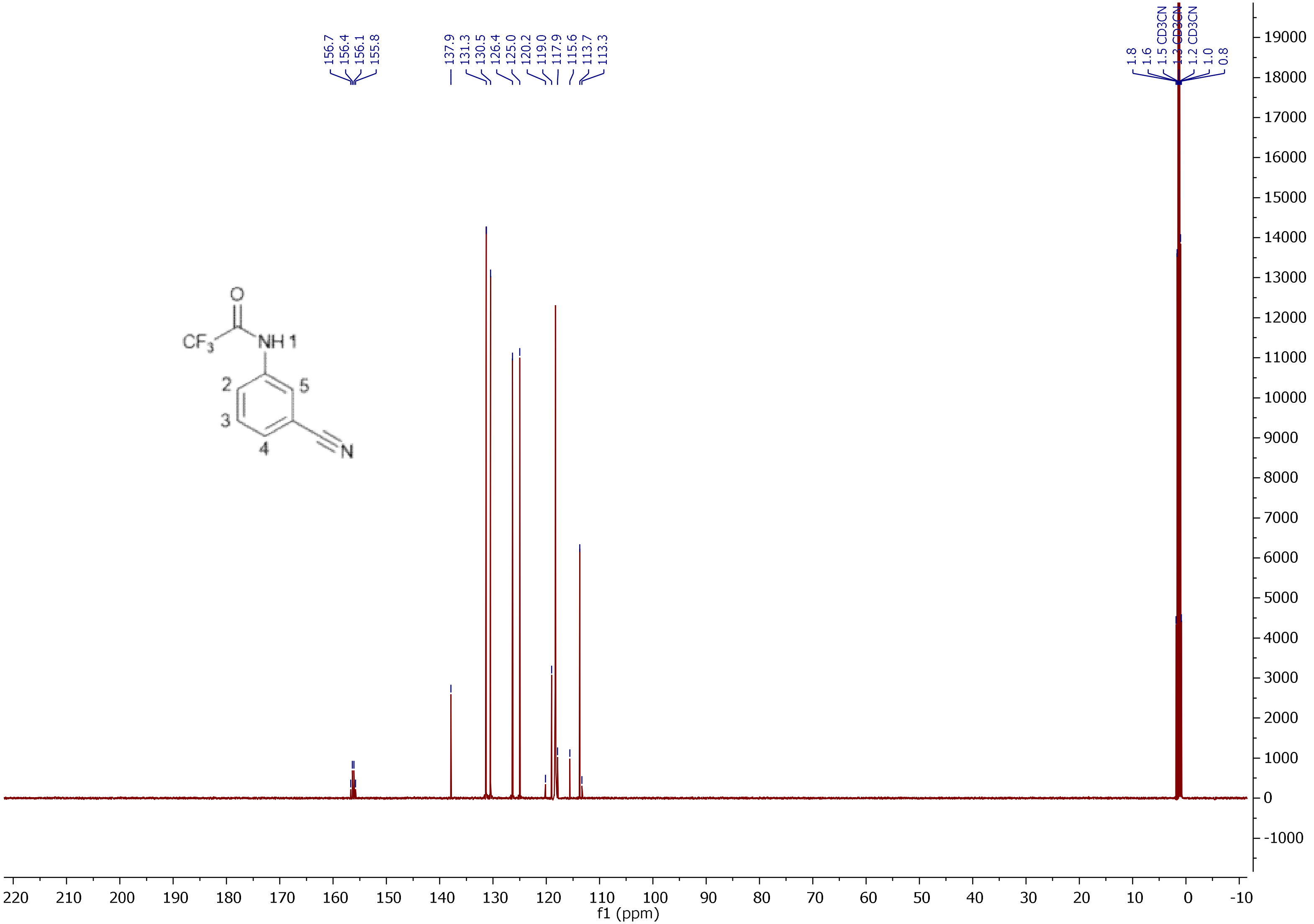

^{19}F NMR (376 MHz, $CDCl_3$) for *N*-(3-cyanophenyl)-2,2,2-trifluoroacetamide **(1v)**

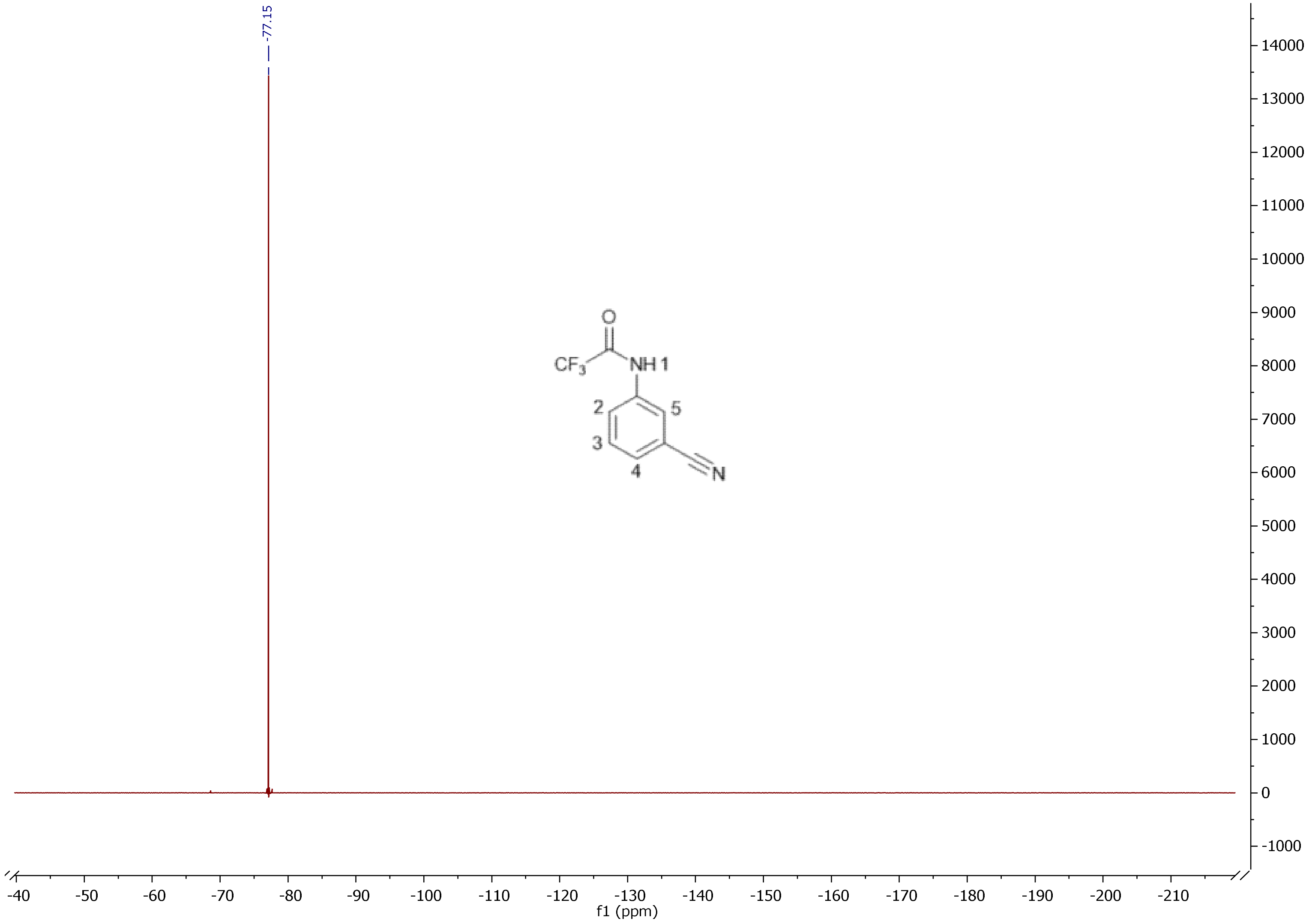

^{1}H NMR (400 MHz, $CDCl_3$) for 2,2,2-trifluoro-*N*-(3-(4,4,5,5-tetramethyl-1,3,2-dioxaborolan-2-yl)phenyl)acetamide **(1w)**

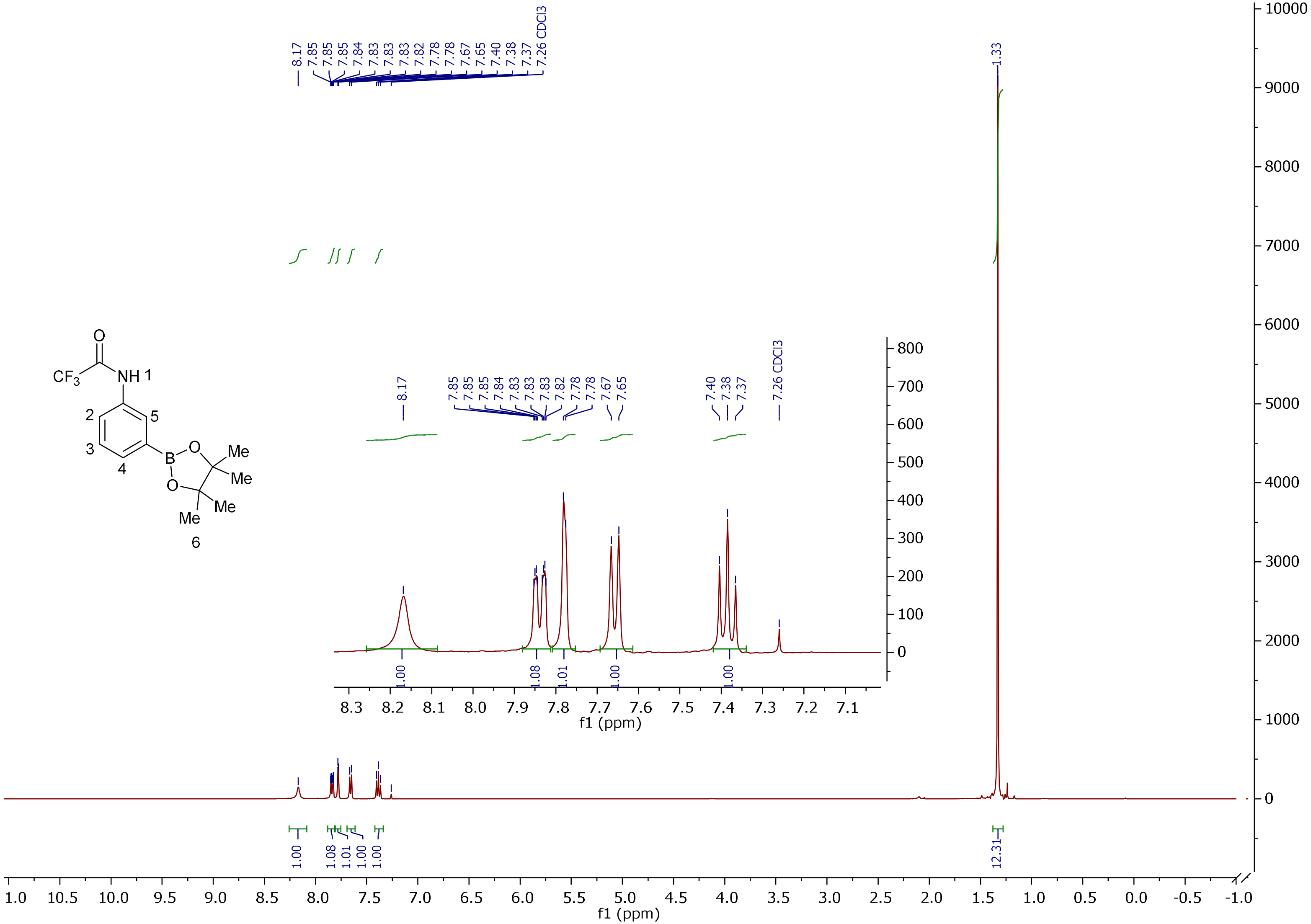

^{13}C NMR (101 MHz, $CDCl_3$) for 2,2,2-trifluoro-*N*-(3-(4,4,5,5-tetramethyl-1,3,2-dioxaborolan-2-yl)phenyl)acetamide **(1w)**

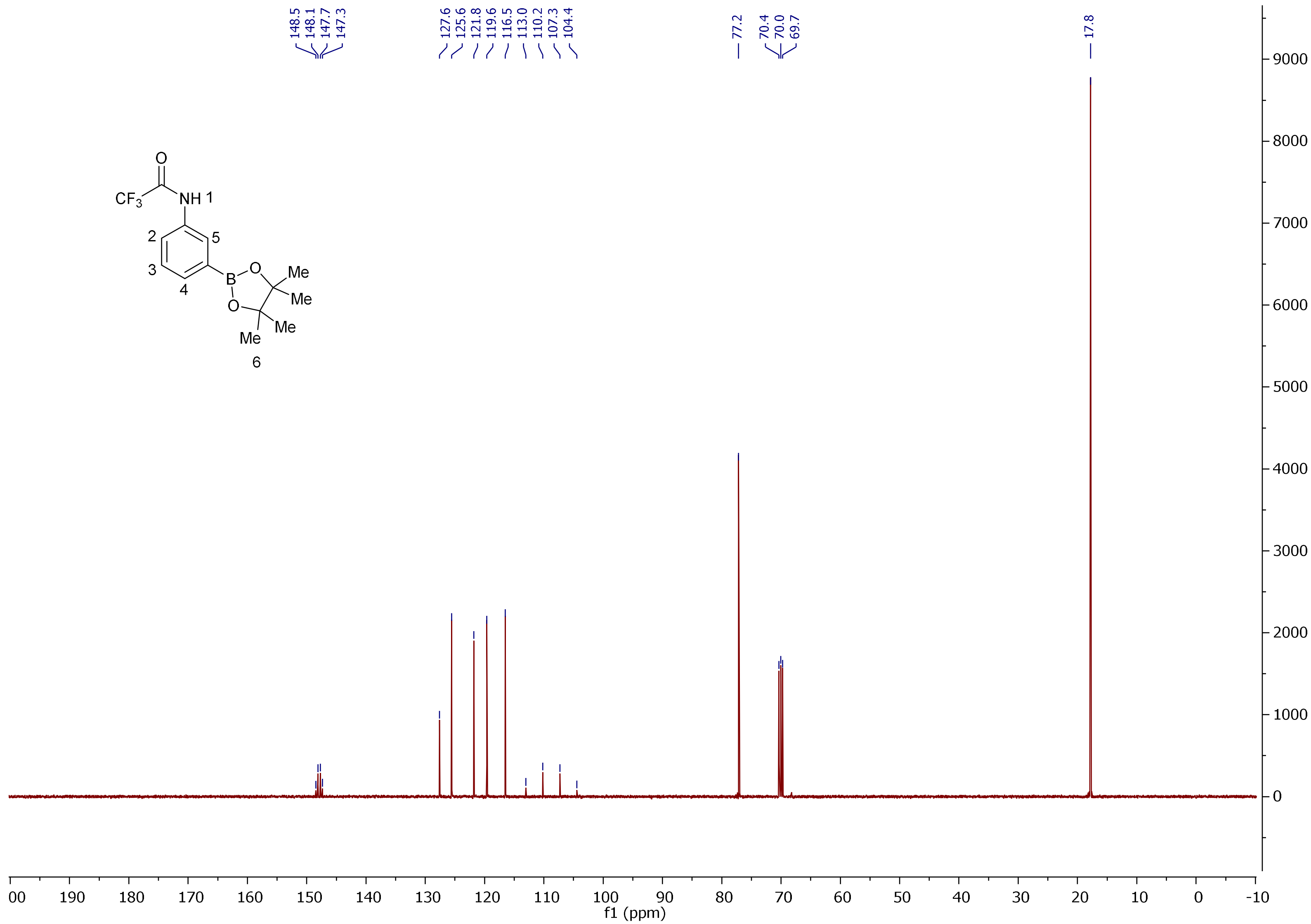

^{19}F NMR (400 MHz, $CDCl_3$) for 2,2,2-trifluoro-*N*-(3-(4,4,5,5-tetramethyl-1,3,2-dioxaborolan-2-yl)phenyl)acetamide **(1w)**

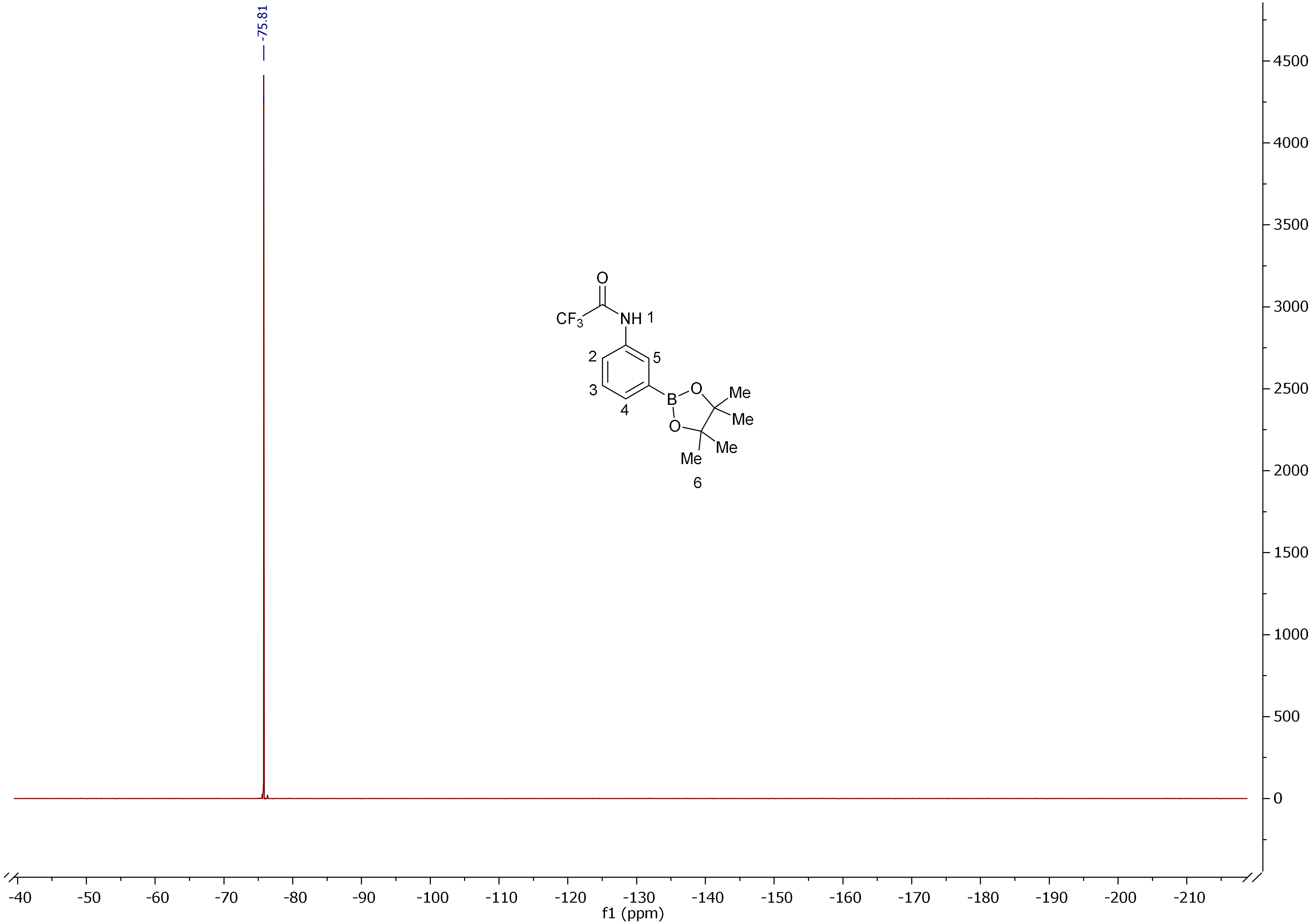

^{11}B NMR (128 MHz, $CDCl_3$) for 2,2,2-trifluoro-*N*-(3-(4,4,5,5-tetramethyl-1,3,2-dioxaborolan-2-yl)phenyl)acetamide **(1w)**

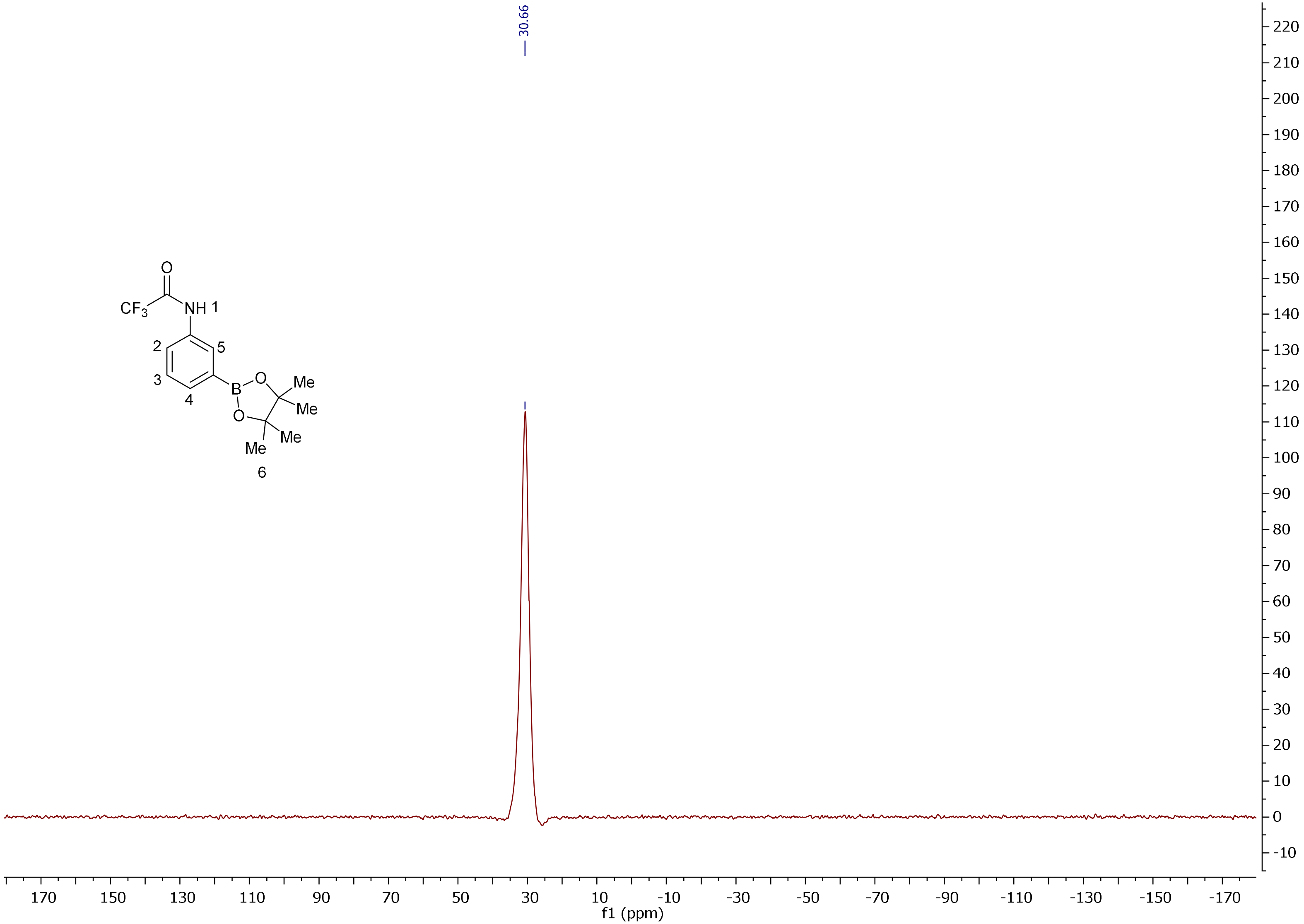

^{1}H NMR (400 MHz, $CDCl_3$) for 3'-amino-[1,1'-biphenyl]-2-carbonitrile

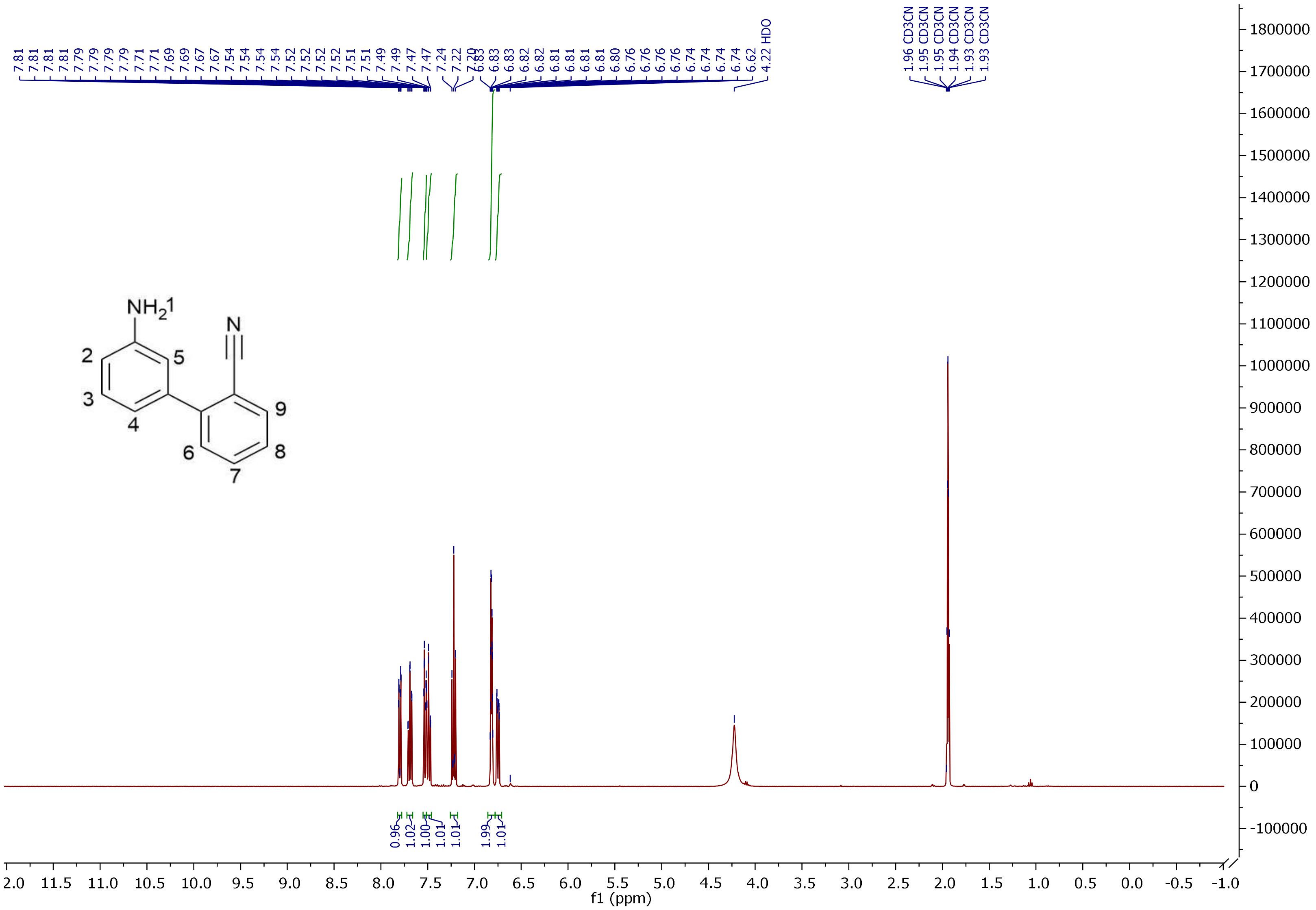

^{13}C NMR (101 MHz, $CDCl_3$) for 3'-amino-[1,1'-biphenyl]-2-carbonitrile

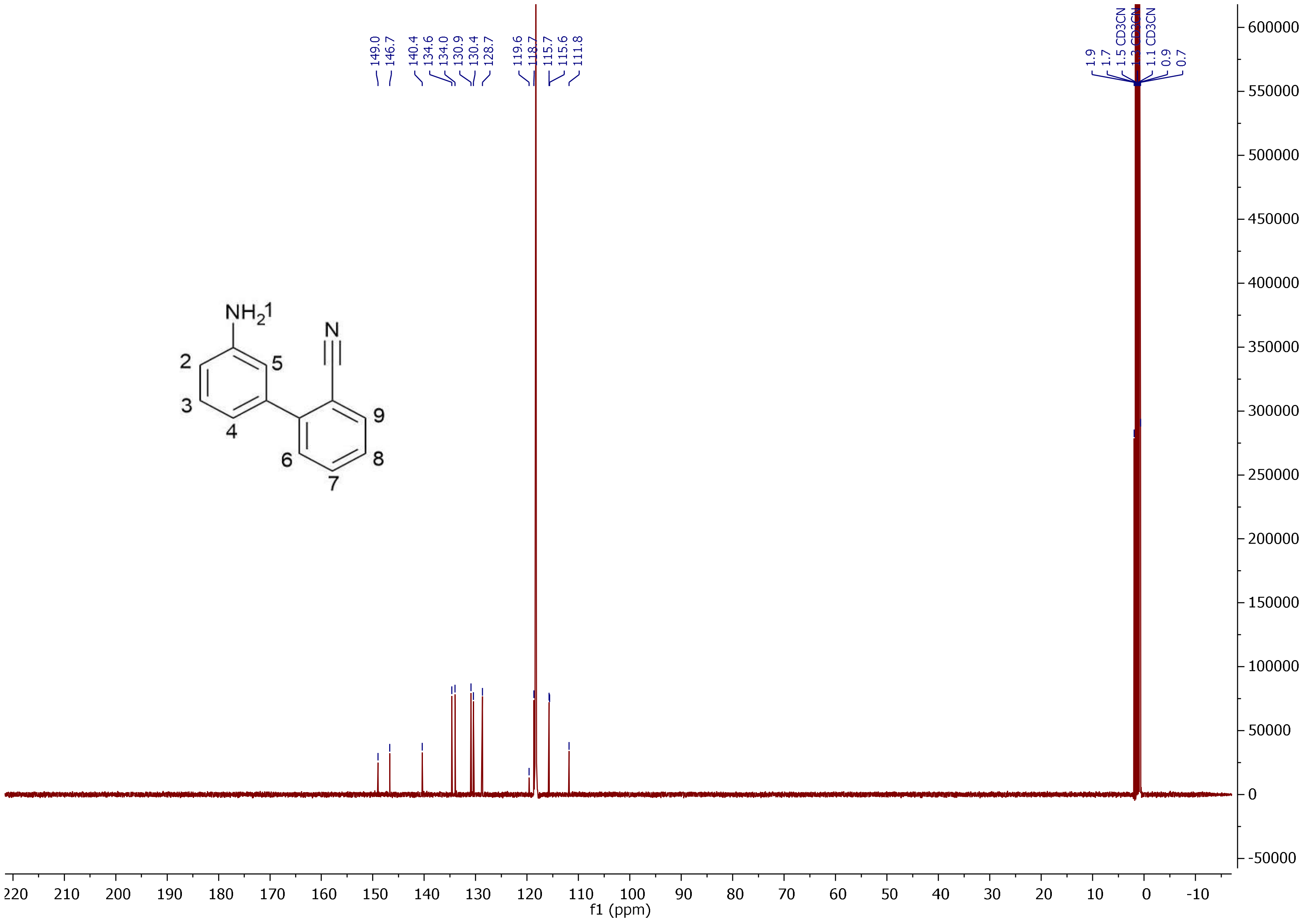

¹H NMR (500 MHz, $CDCl_3$) for *N*-(2'-cyano-[1,1'-biphenyl]-3-yl)-2,2,2-trifluoroacetamide **(1x)**

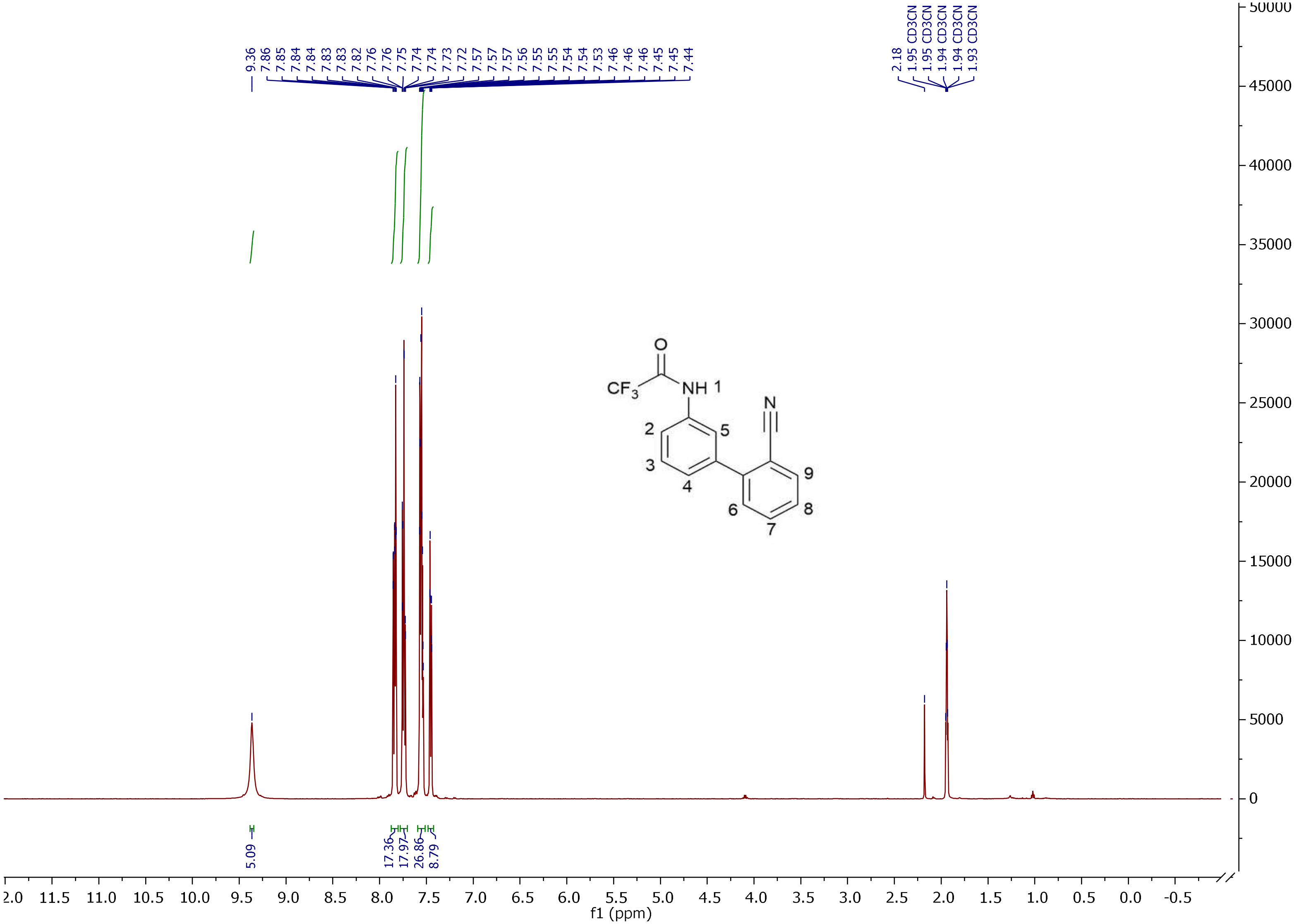

^{13}C NMR (126 MHz, $CDCl_3$) for *N*-(2'-cyano-[1,1'-biphenyl]-3-yl)-2,2,2-trifluoroacetamide **(1x)**

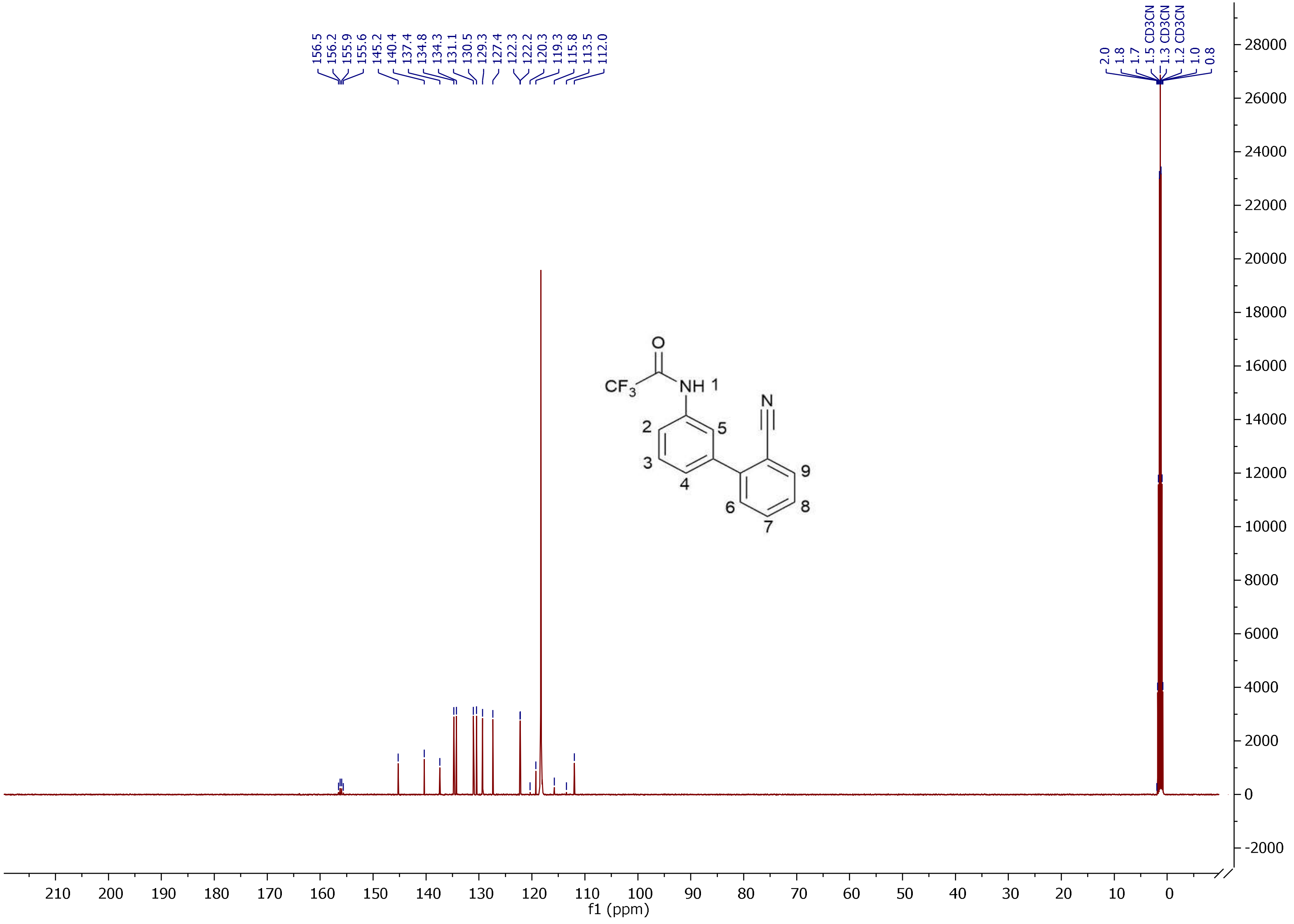

^{19}F NMR (376 MHz, $CDCl_3$) for *N*-(2'-cyano-[1,1'-biphenyl]-3-yl)-2,2,2-trifluoroacetamide **(1x)**

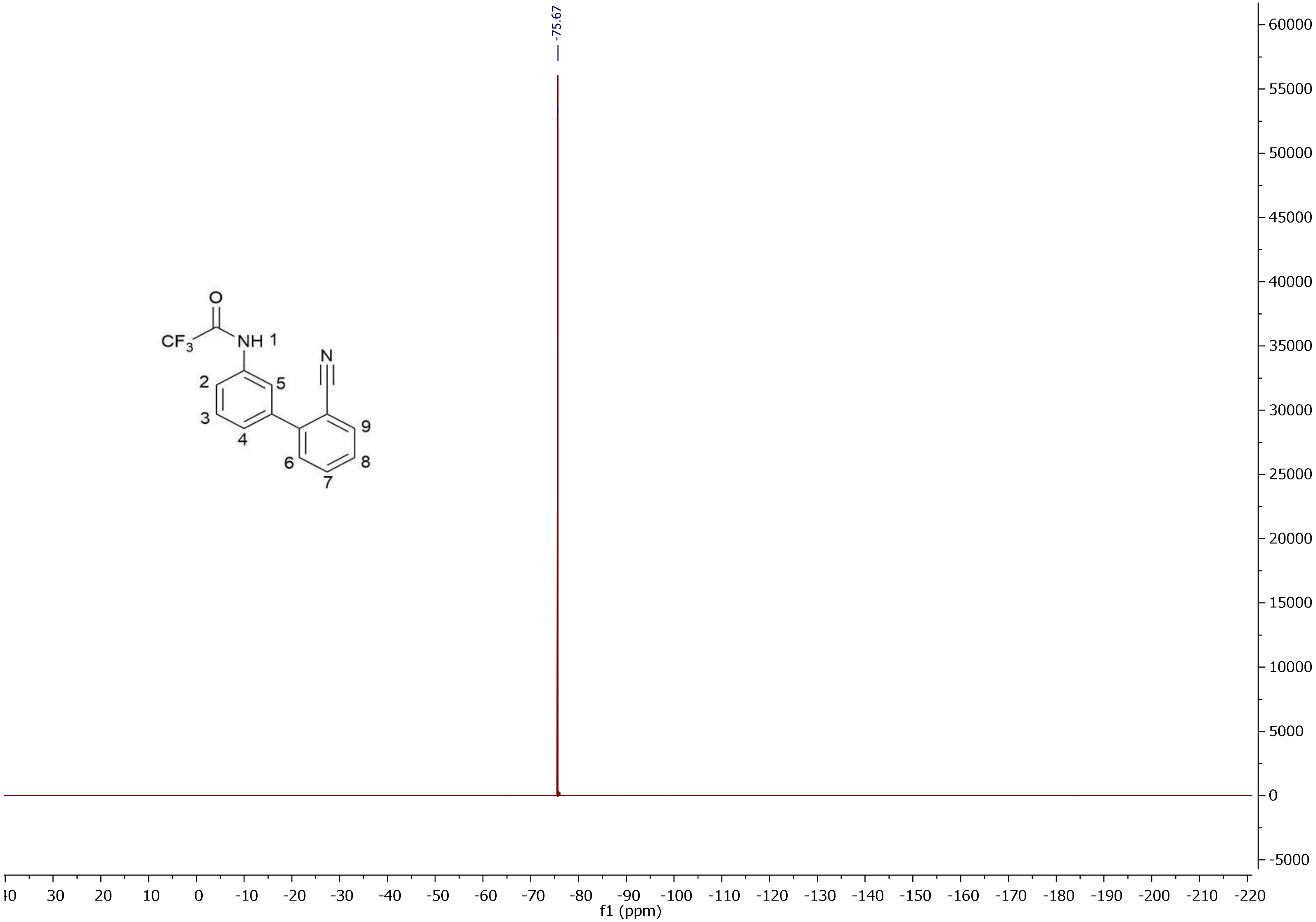

^{1}H NMR (500 MHz, $CDCl_3$) for 2,2,2-trifluoro-*N*-(3-(pyrimidin-5-yl)phenyl)acetamide **(1y)**

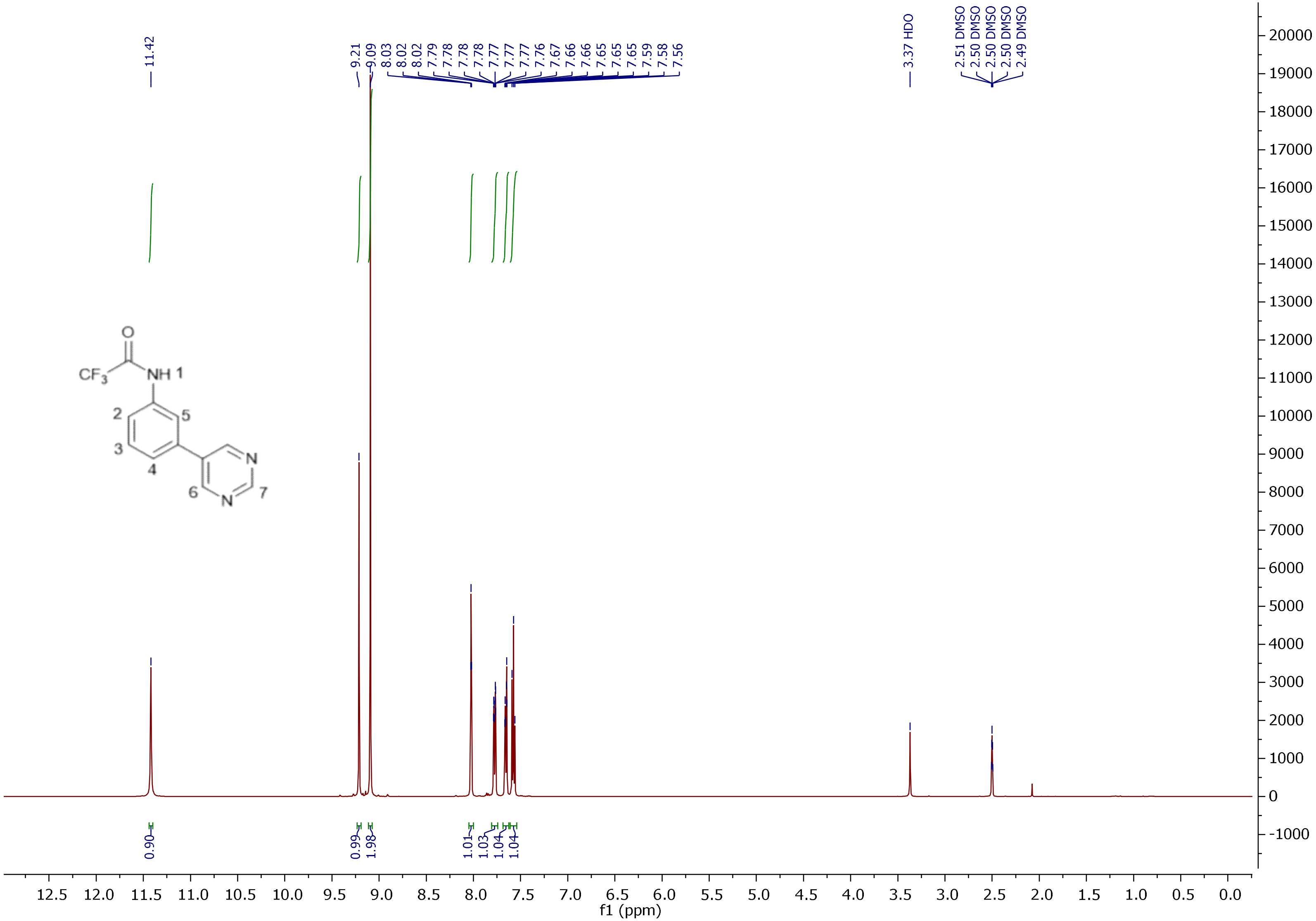

^{13}C NMR (126 MHz, $CDCl_3$) for 2,2,2-trifluoro-*N*-(3-(pyrimidin-5-yl)phenyl)acetamide **(1y)**

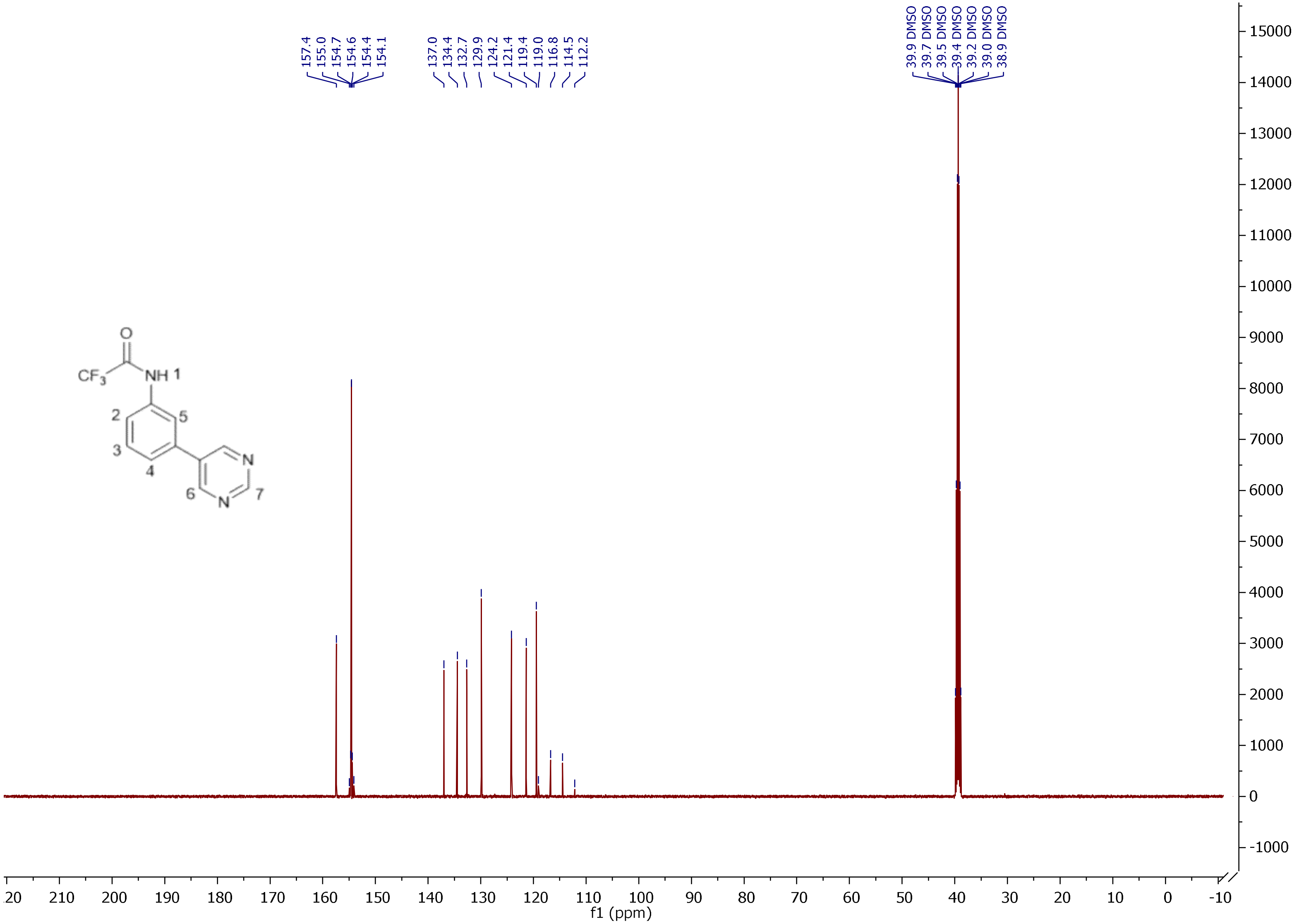

^{19}F NMR (376 MHz, $CDCl_3$) for 2,2,2-trifluoro-*N*-(3-(pyrimidin-5-yl)phenyl)acetamide **(1y)**

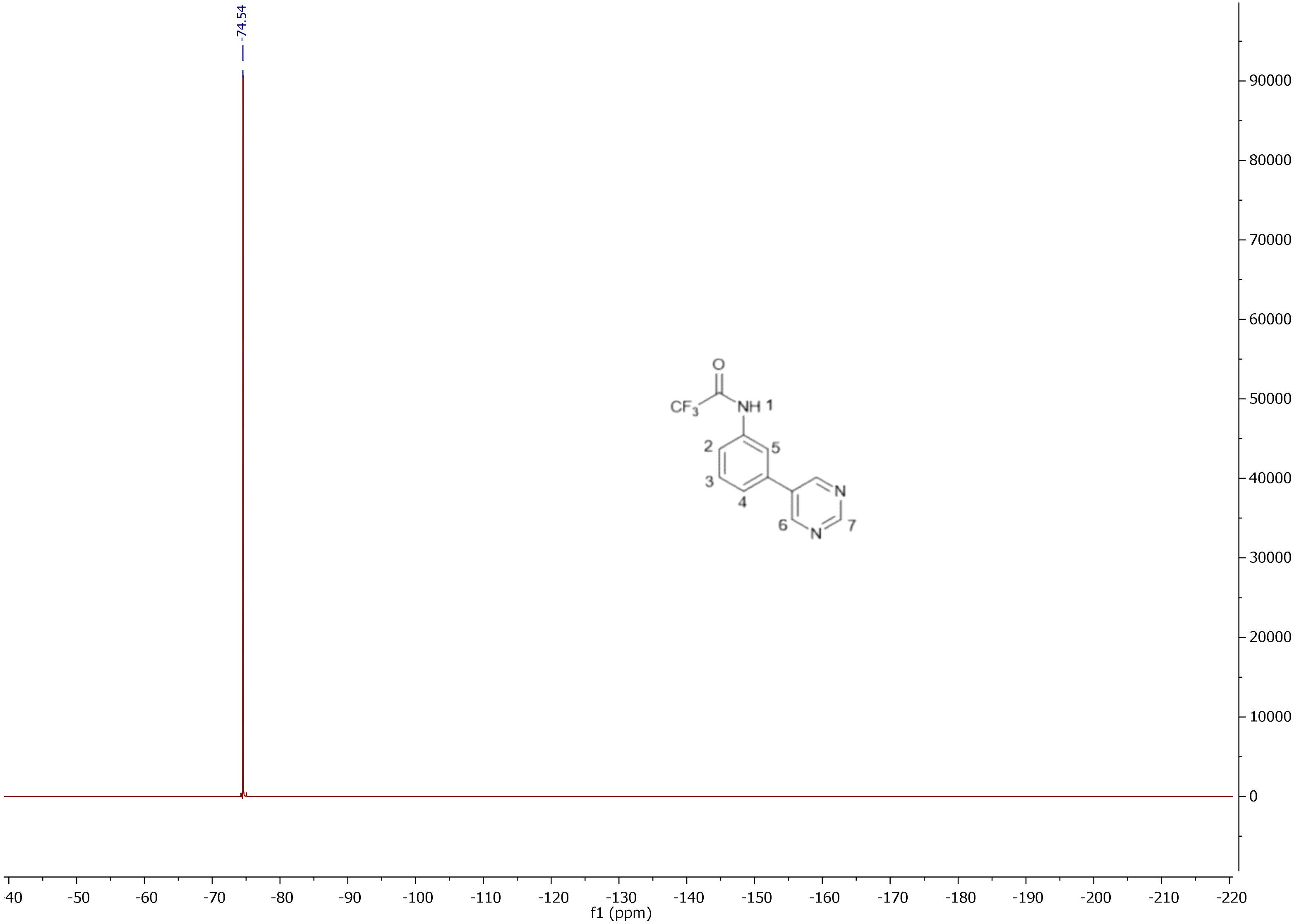

^{1}H NMR (400 MHz, $CDCl_3$) for methyl 2-(4-isobutylphenyl)propanoate

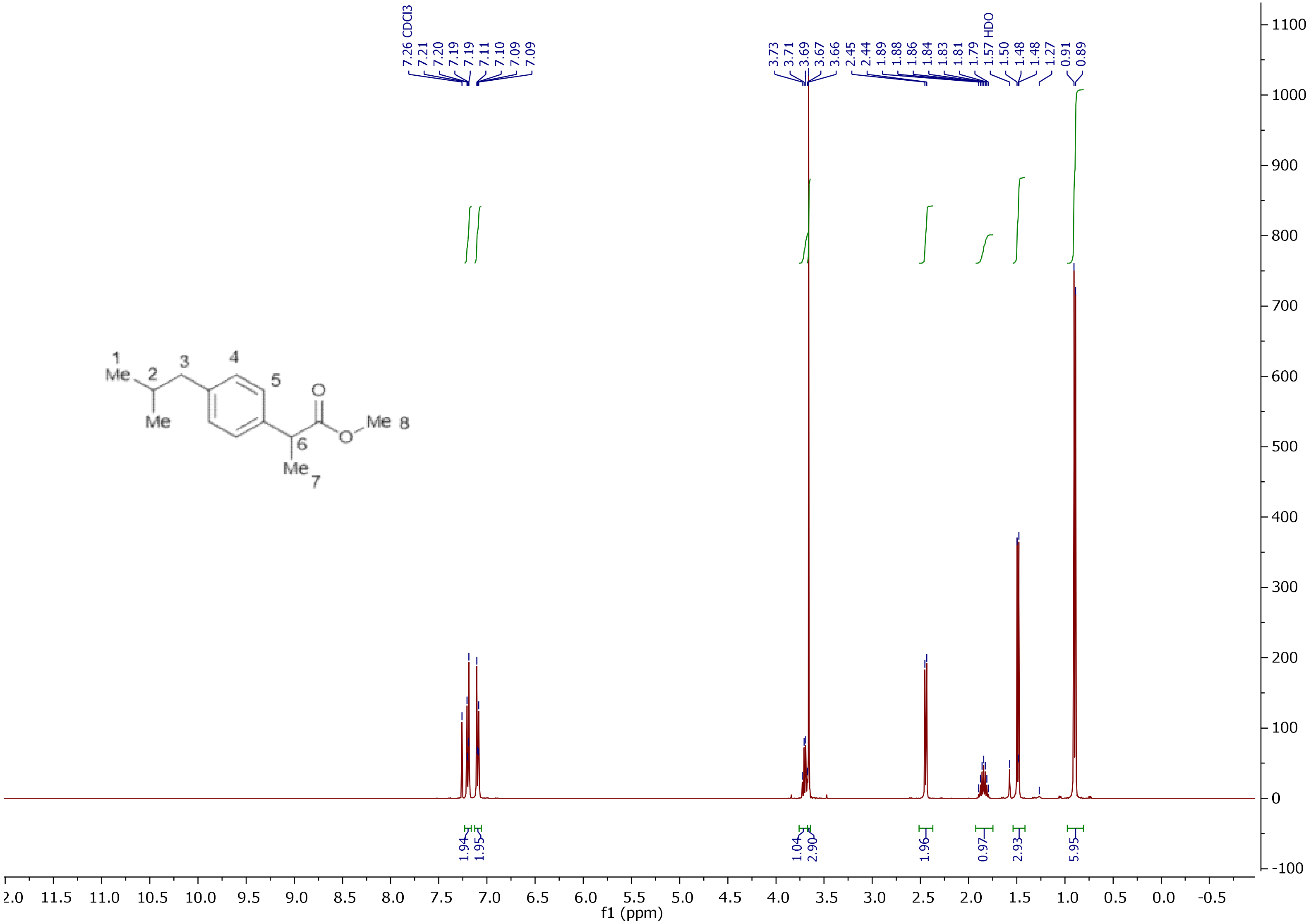

^{13}C NMR (101 MHz, $CDCl_3$) for methyl 2-(4-isobutylphenyl)propanoate

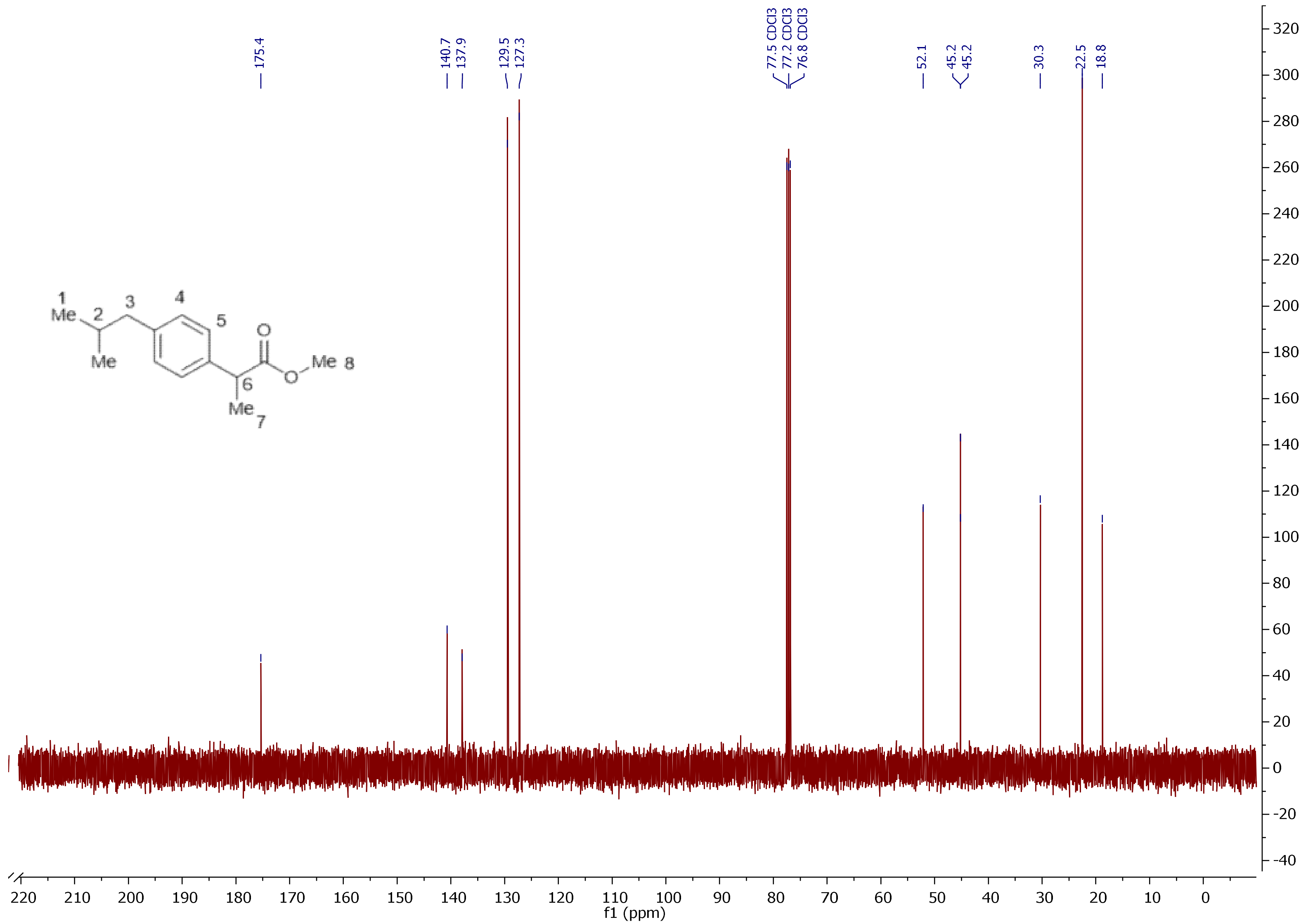

[1]**H NMR** (700 MHz, $CDCl_3$) for methyl 2-(4-isobutyl-3-(2,2,2-trifluoroacetamido)phenyl)propanoate **(1z)**

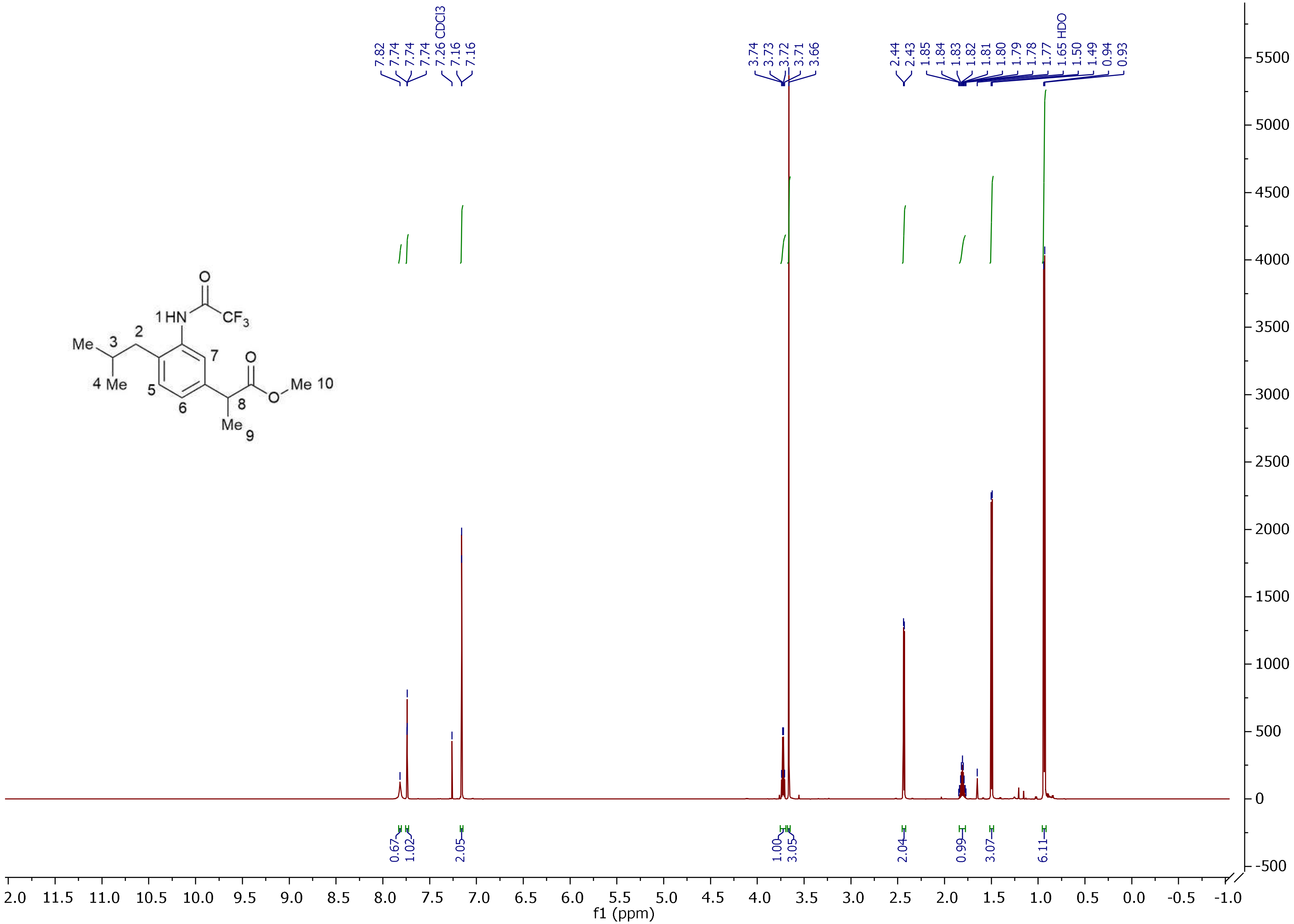

^{13}C NMR (176 MHz, $CDCl_3$) for methyl 2-(4-isobutyl-3-(2,2,2-trifluoroacetamido)phenyl)propanoate **(1z)**

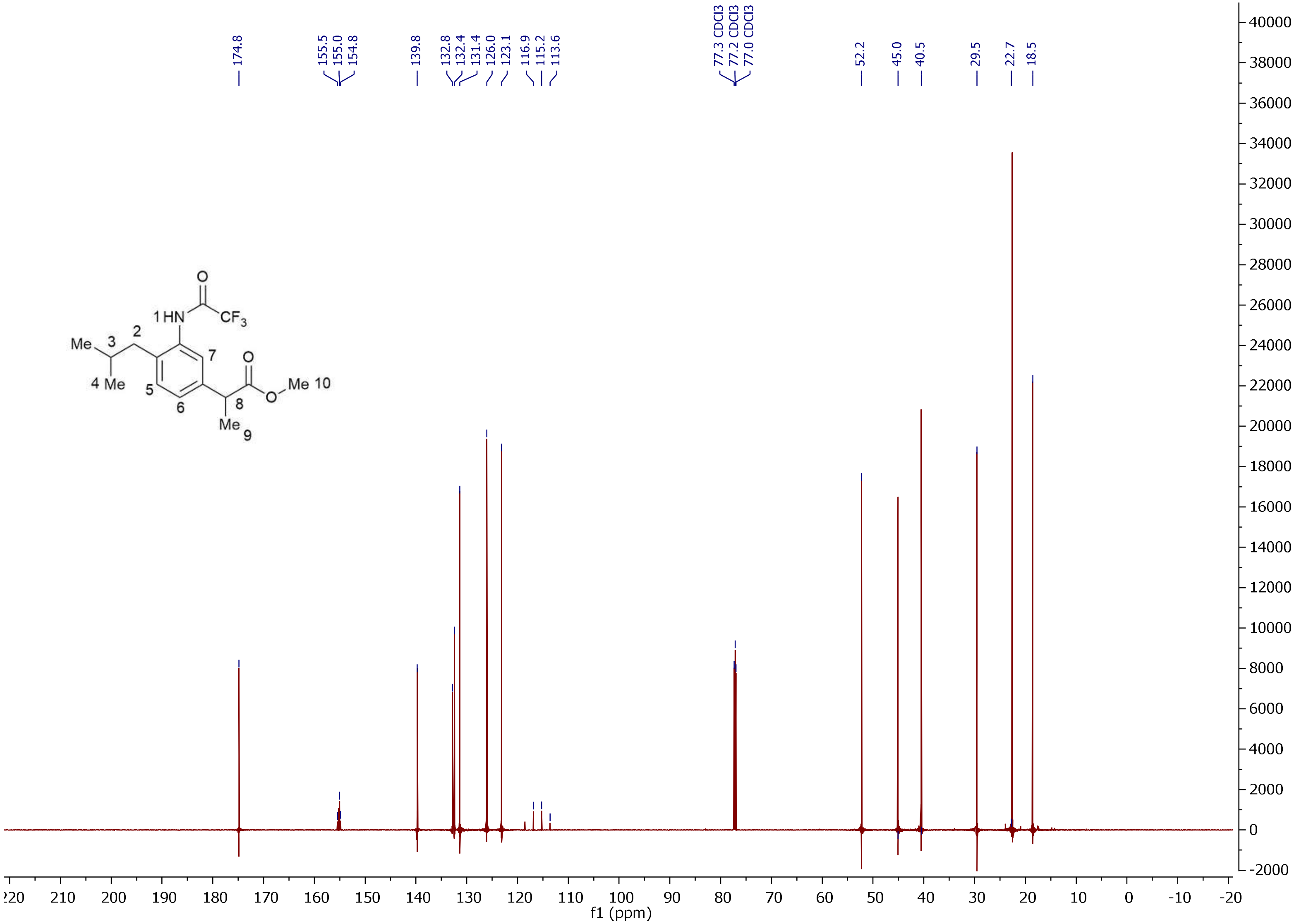

^{19}F NMR (376 MHz, $CDCl_3$) for methyl 2-(4-isobutyl-3-(2,2,2-trifluoroacetamido)phenyl)propanoate **(1z)**

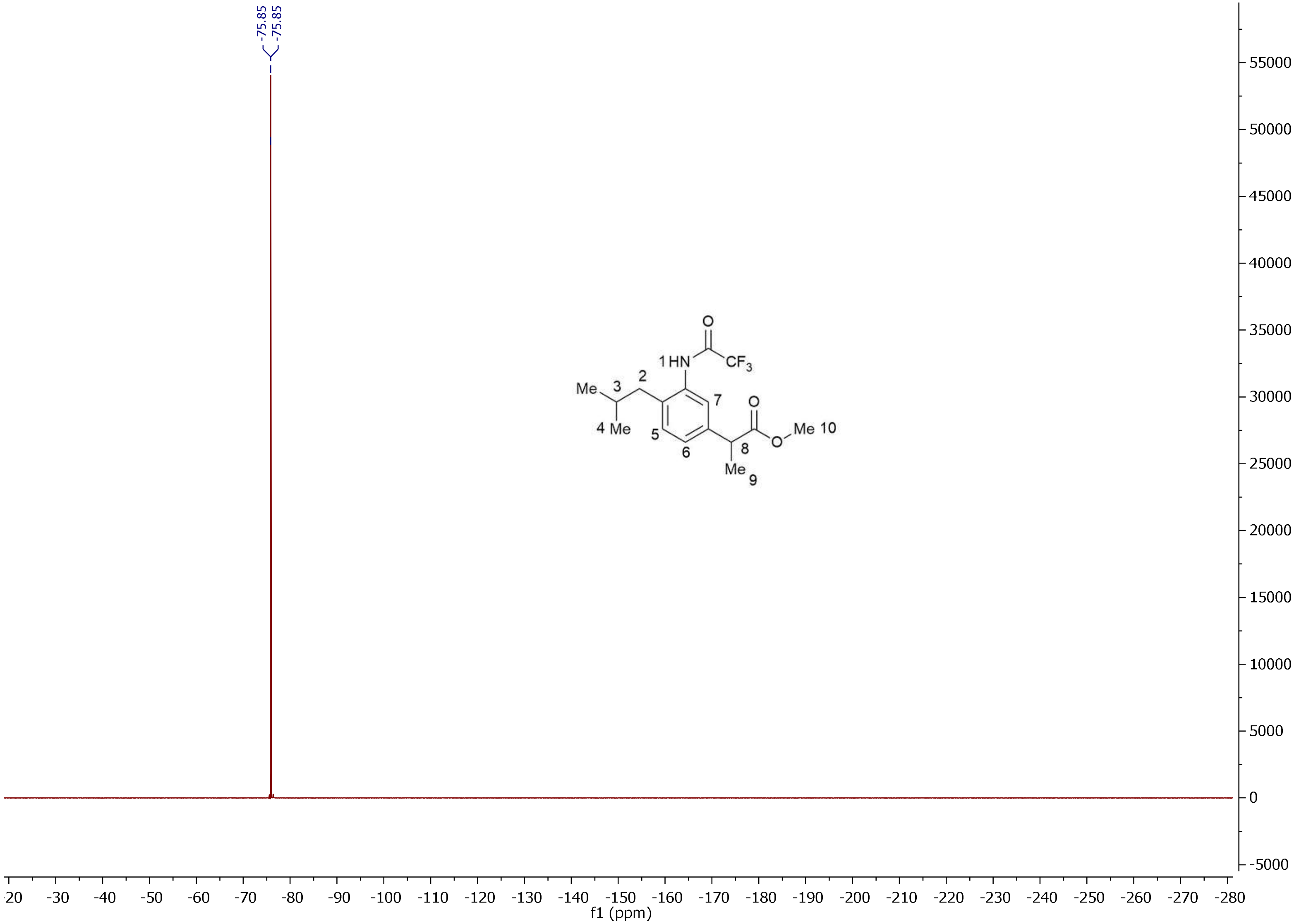

^{1}H NMR (400 MHz, acetone-d^6) for 3-(2,2,2-trifluoroacetamido)benzoic acid

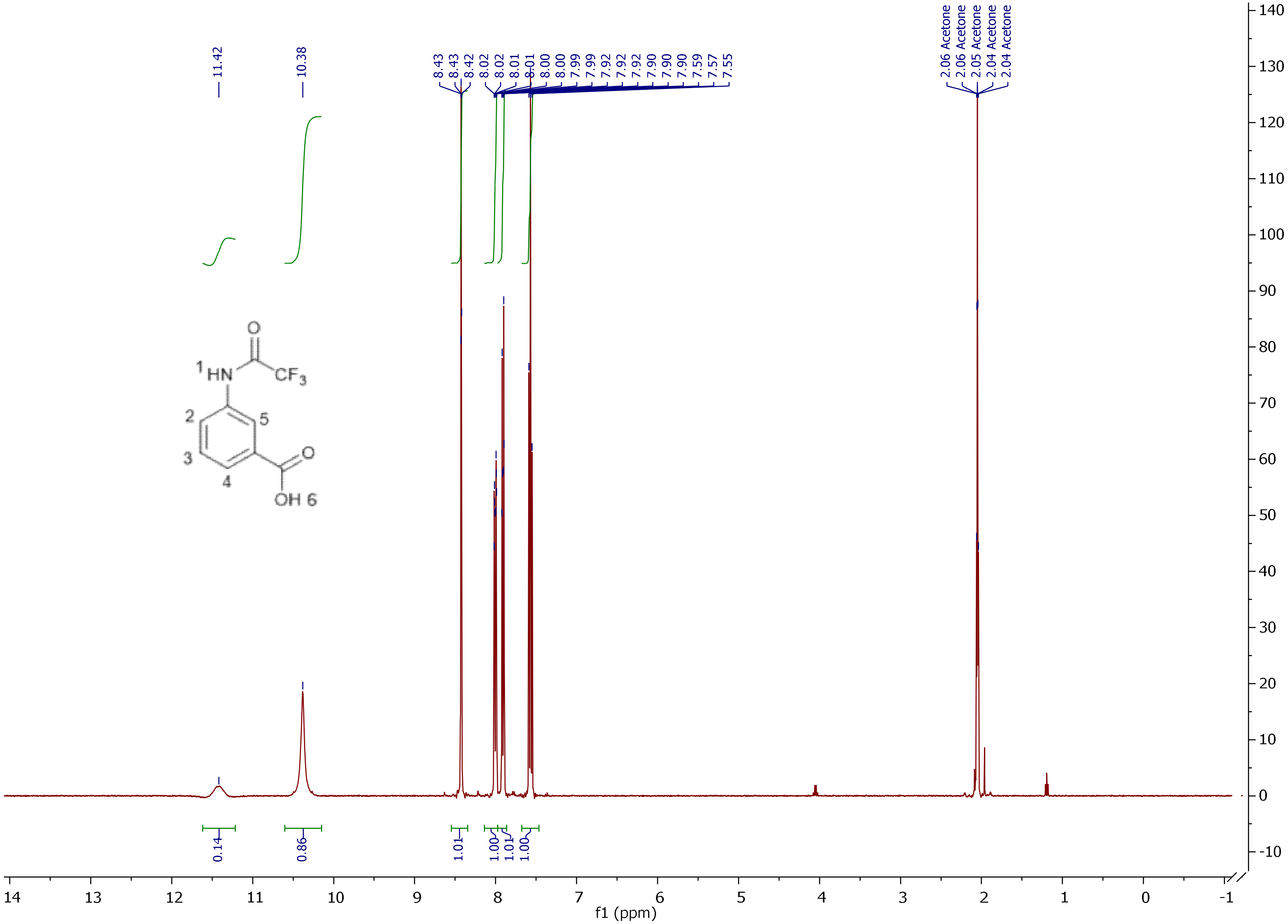

^{13}C NMR (176 MHz, acetone-d^6) for 3-(2,2,2-trifluoroacetamido)benzoic acid

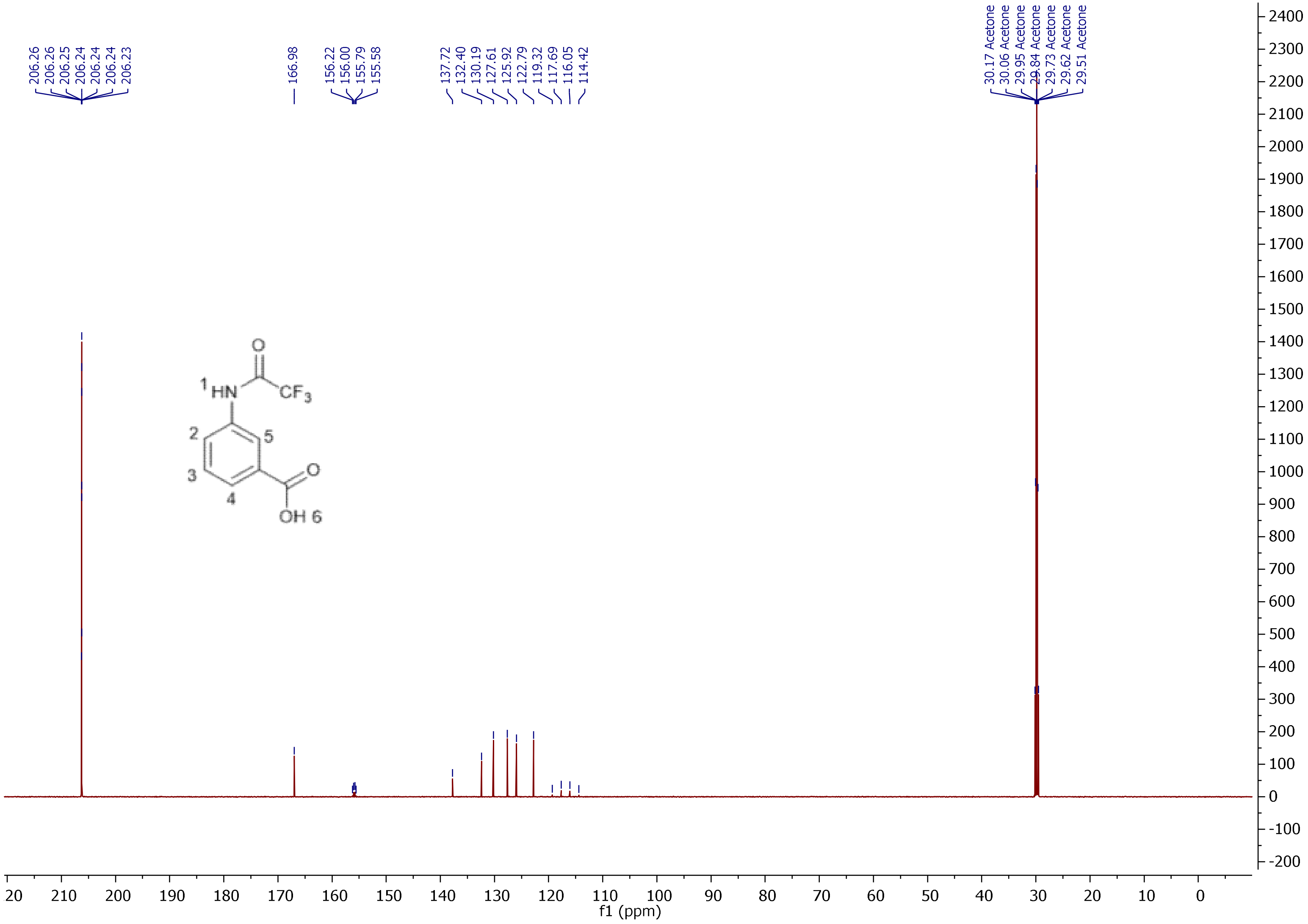

^{19}F NMR (376 MHz, acetone-d^6) for 3-(2,2,2-trifluoroacetamido)benzoic acid

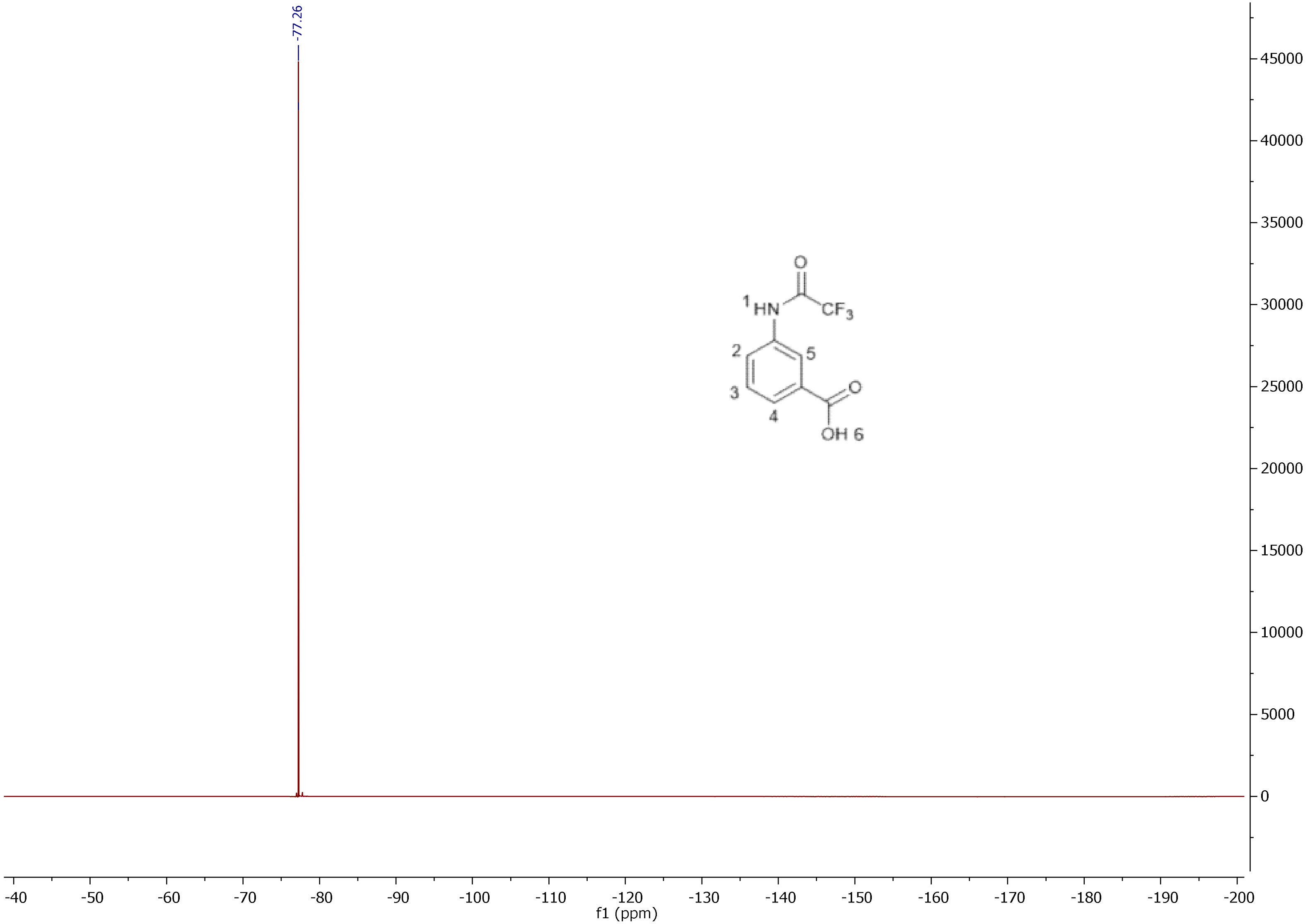

¹H NMR (700 MHz, CD_3CN) for (*R*)-3-methoxy-2-methyl-3-oxopropyl 3-(2,2,2-trifluoroacetamido)benzoate **(1aa)**

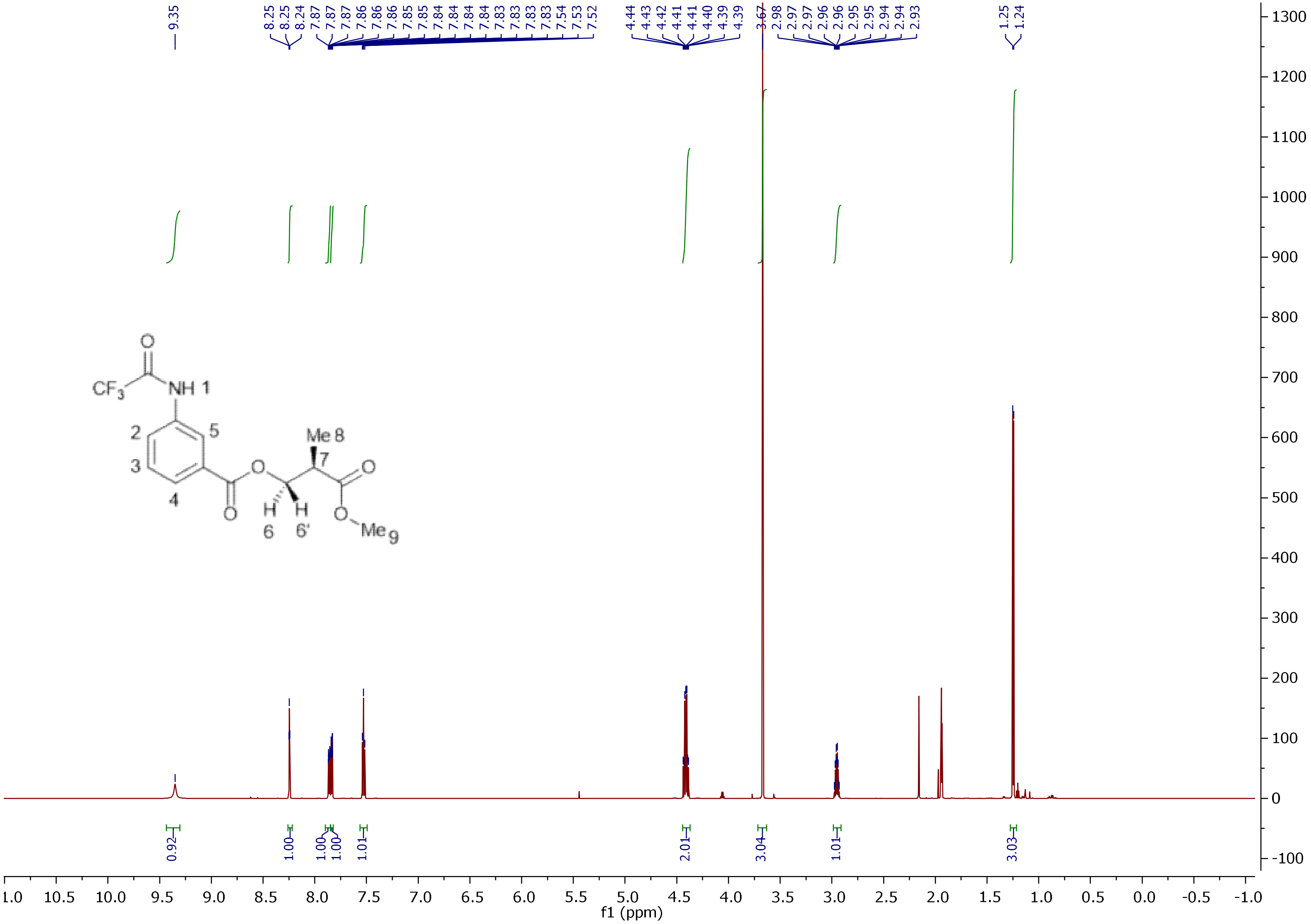

^{13}C NMR (176 MHz, CD_3CN) for (*R*)-3-methoxy-2-methyl-3-oxopropyl 3-(2,2,2-trifluoroacetamido)benzoate **(1aa)**

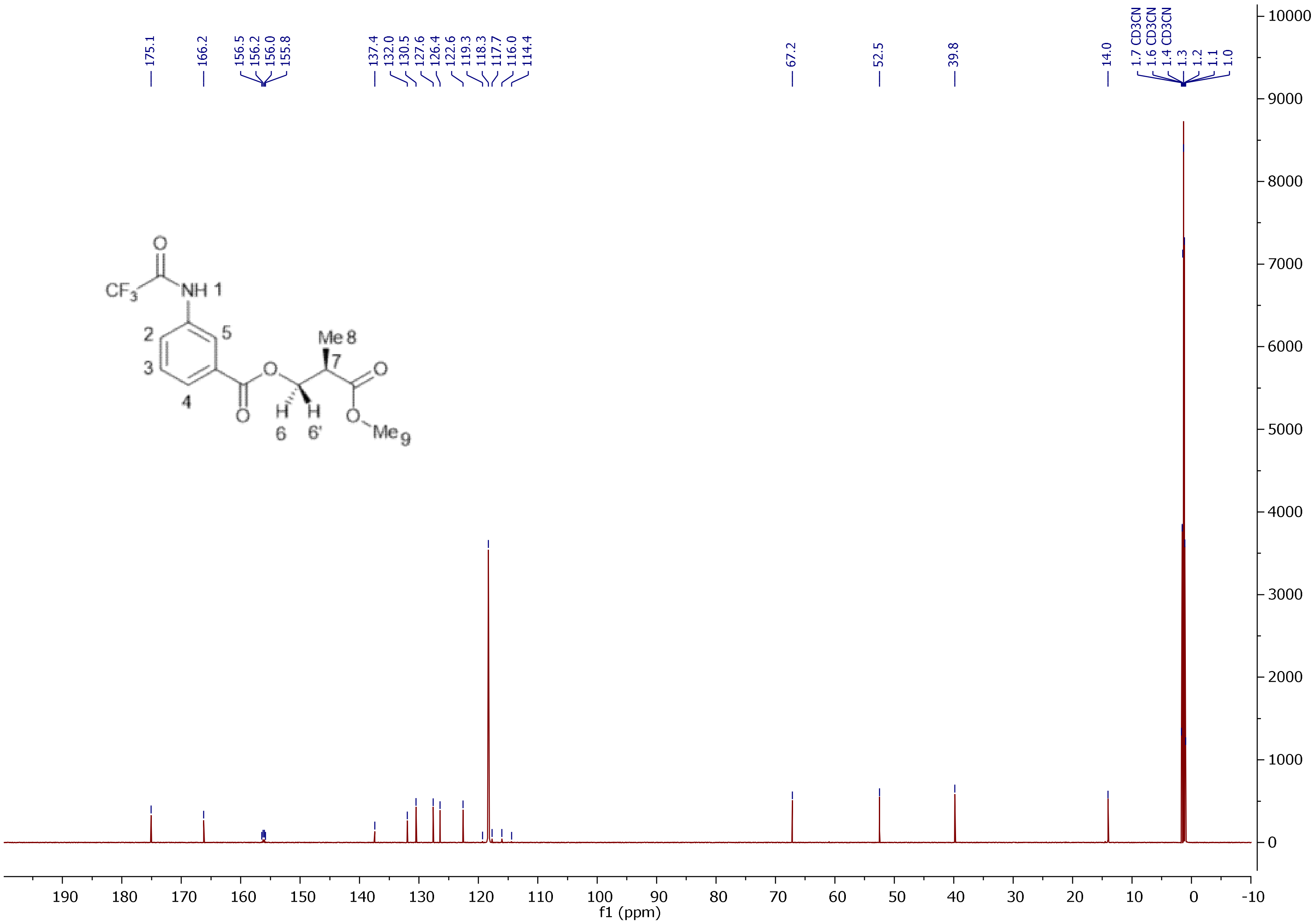

^{19}F NMR (376 MHz, $CDCl_3$) for (*R*)-3-methoxy-2-methyl-3-oxopropyl 3-(2,2,2-trifluoroacetamido)benzoate **(1aa)**

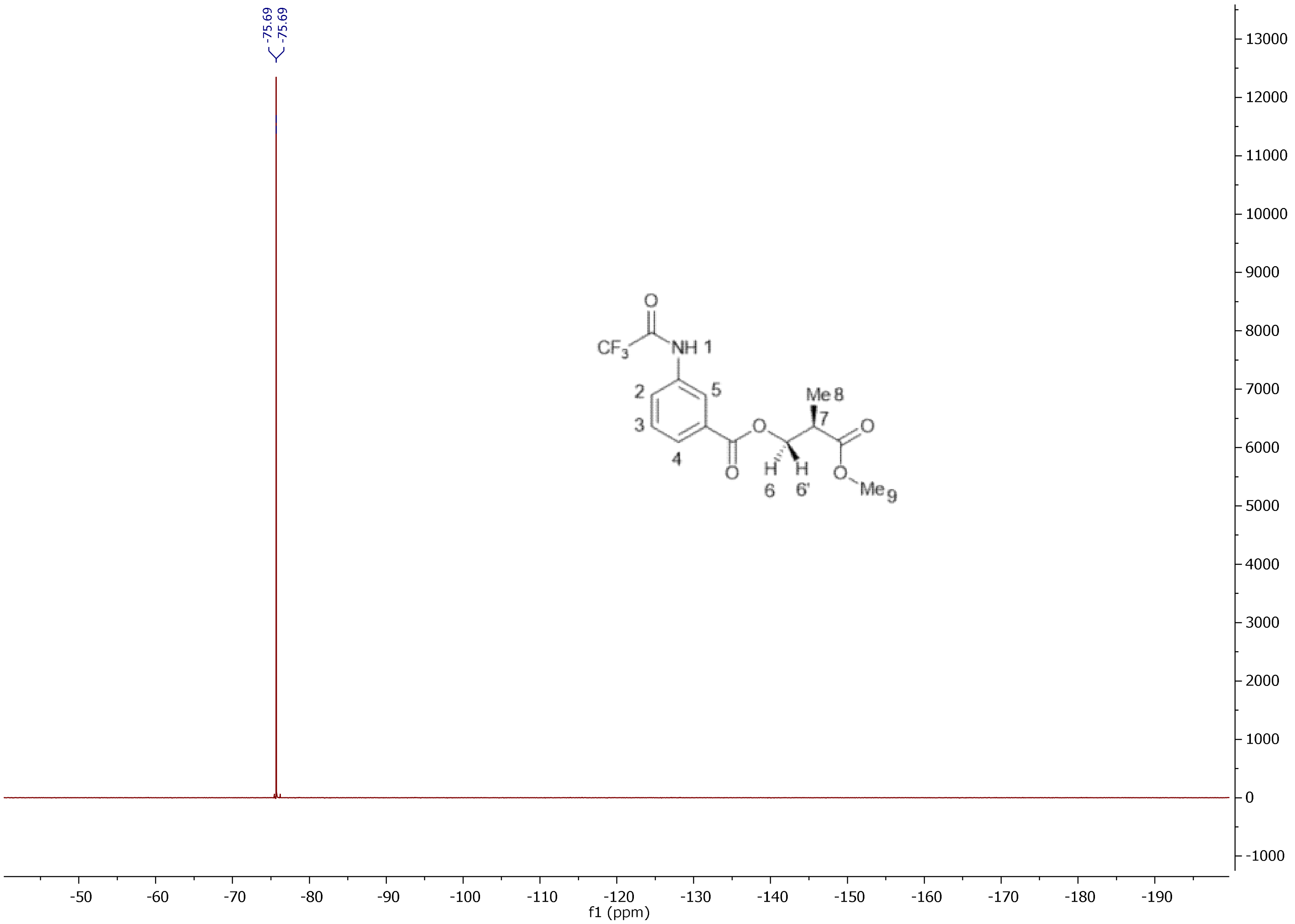

^{1}H NMR (700 MHz, CD_3CN) for fluorination of 2,2,2-trifluoro-*N*-phenylacetamide **(3c)**

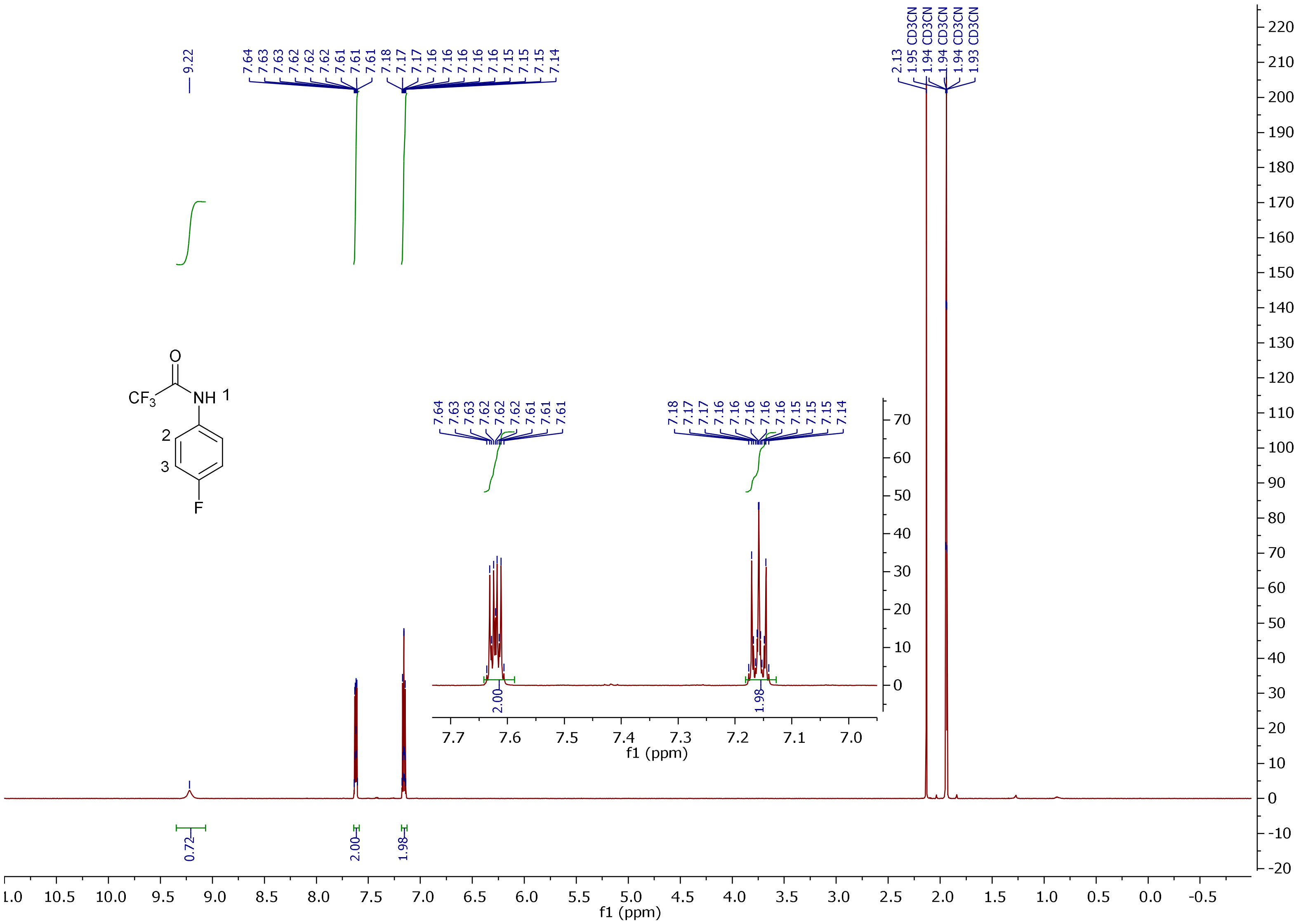

^{13}C NMR (176 MHz, CD_3CN) for fluorination of 2,2,2-trifluoro-*N*-phenylacetamide **(3c)**

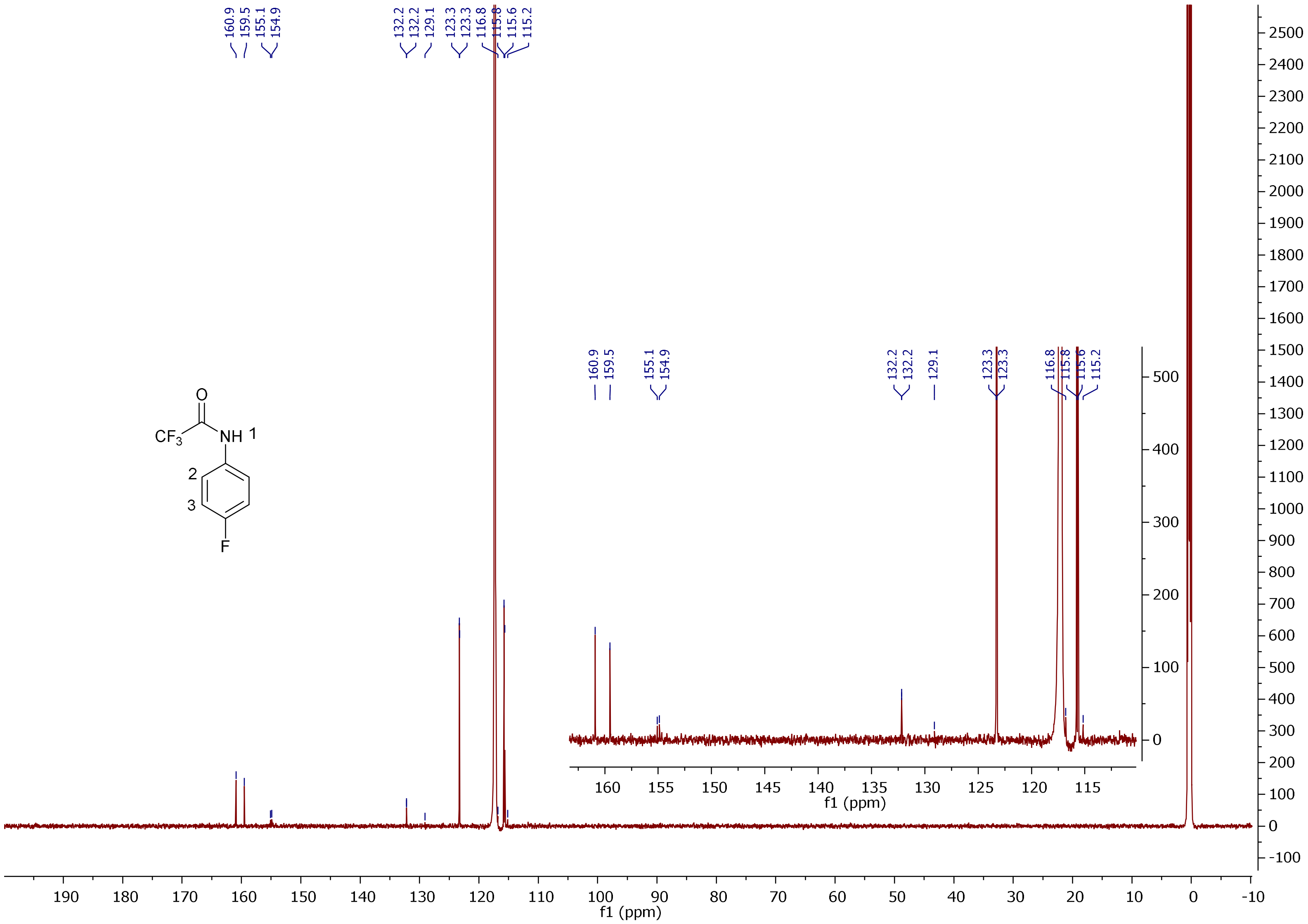

^{19}F{^{1}H} NMR (376 MHz, CD_3CN) for fluorination of 2,2,2-trifluoro-*N*-phenylacetamide **(3c)**

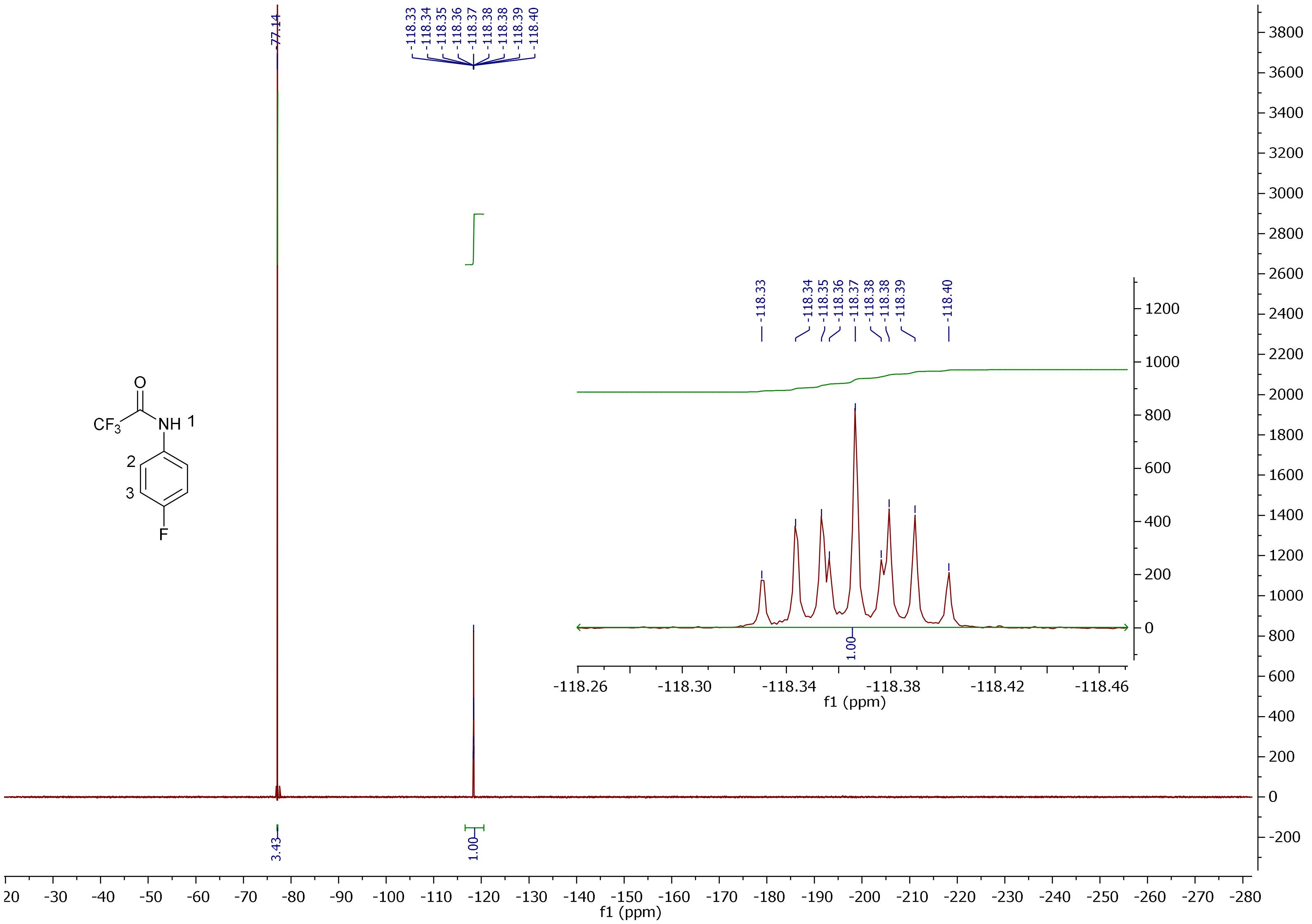

^{1}H NMR (700 MHz, CD_3CN) for 2,2,2-trifluoro-*N*-(2-fluorophenyl)acetamide **(2c)**

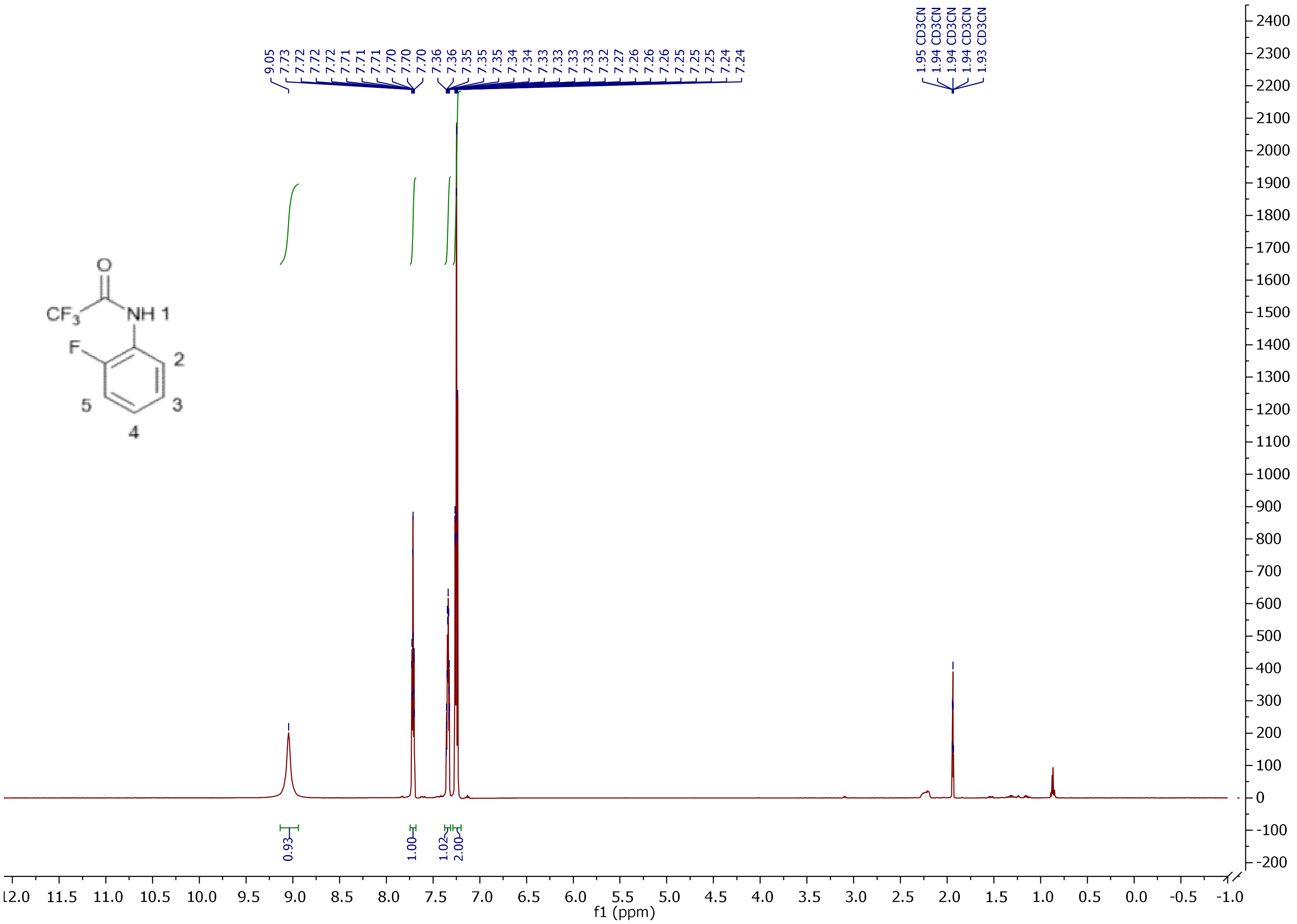

^{13}C NMR (176 MHz, CD_3CN) for 2,2,2-trifluoro-*N*-(2-fluorophenyl)acetamide **(2c)**

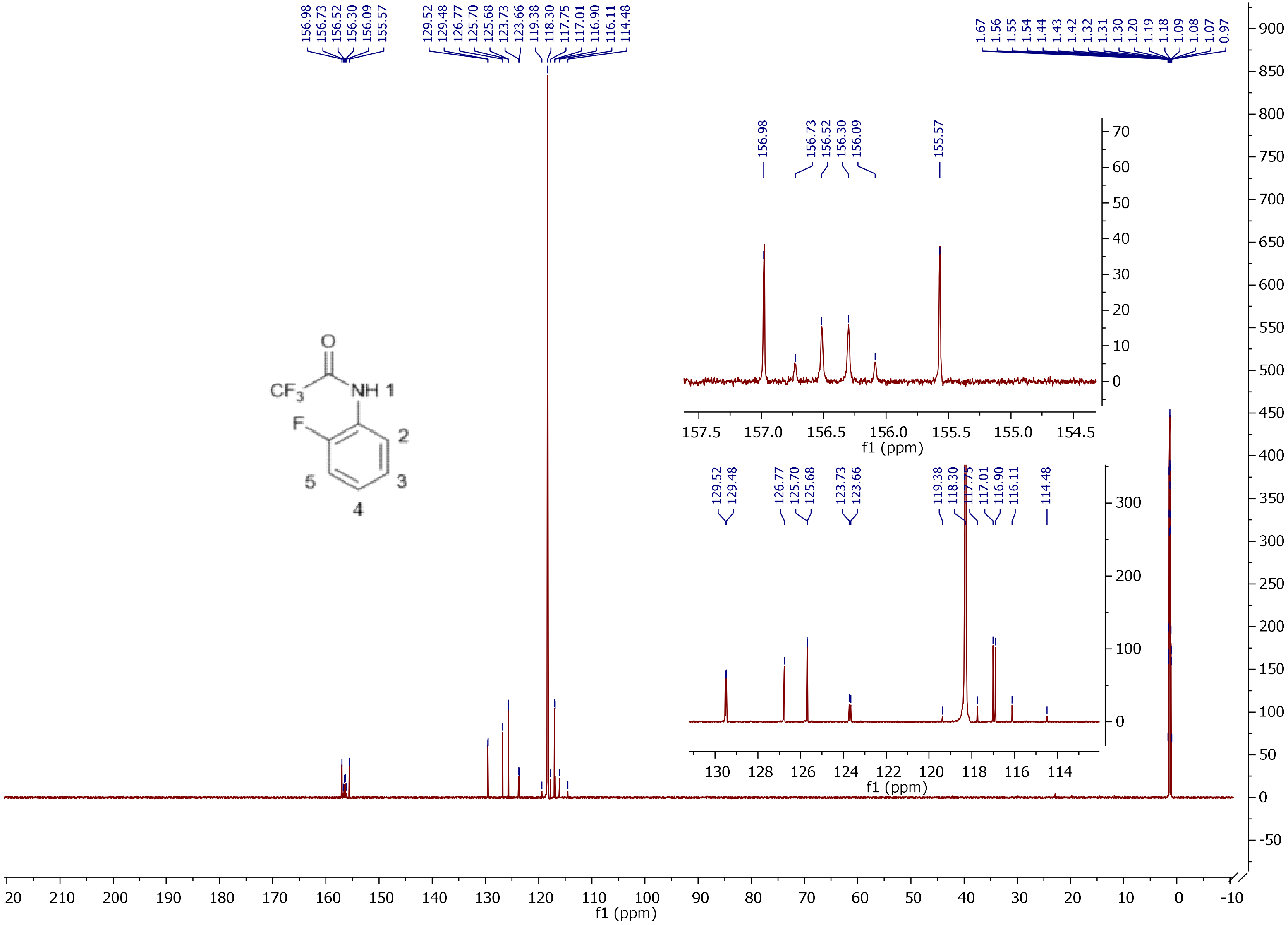

^{19}F{^{1}H} NMR (376 MHz, CD_3CN) for 2,2,2-trifluoro-*N*-(2-fluorophenyl)acetamide **(2c)**

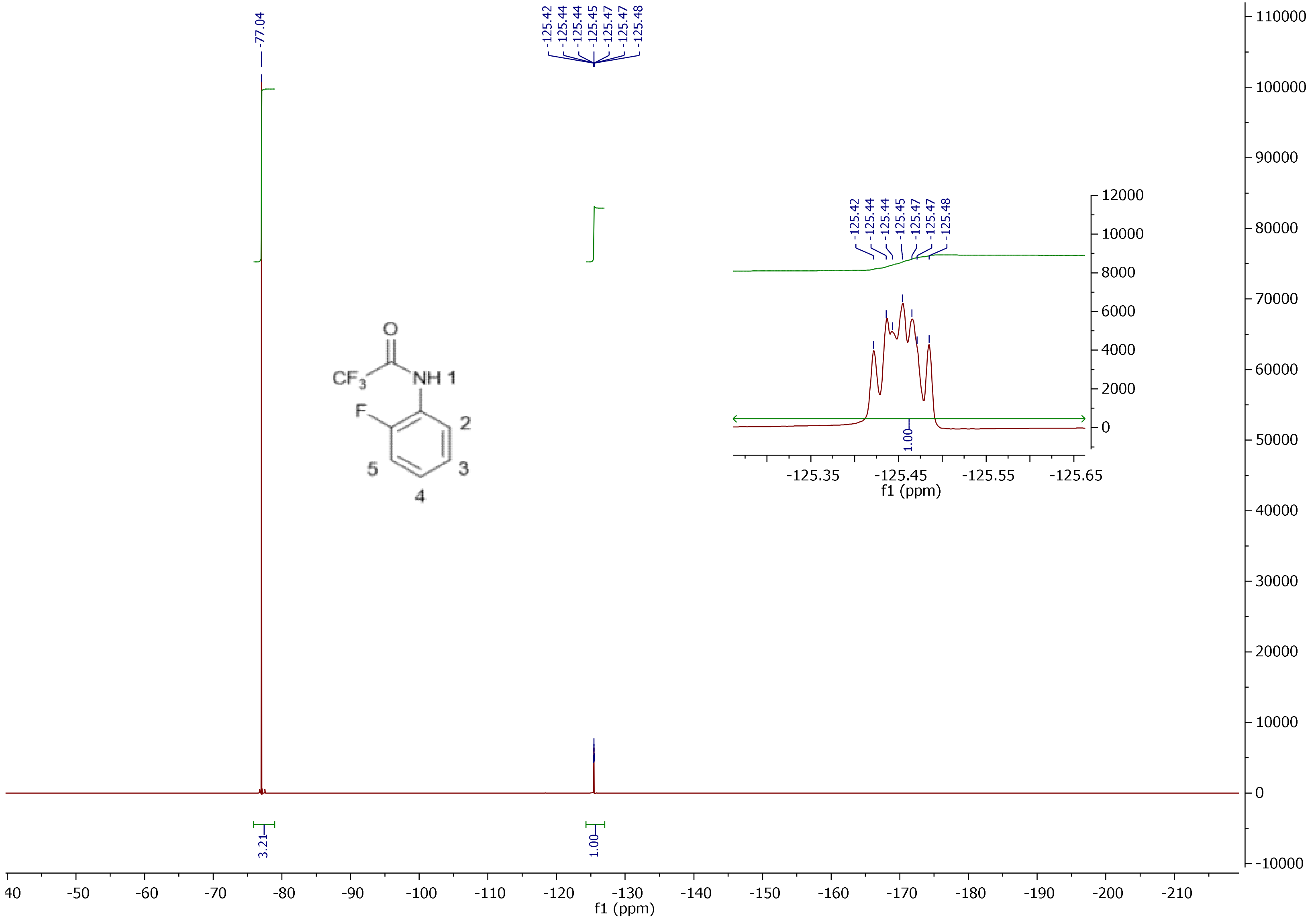

^{1}H NMR (700 MHz, CD_3CN) for fluorination of 2,2,3,3,3-pentafluoro-*N*-phenylpropanamide **(3d)**

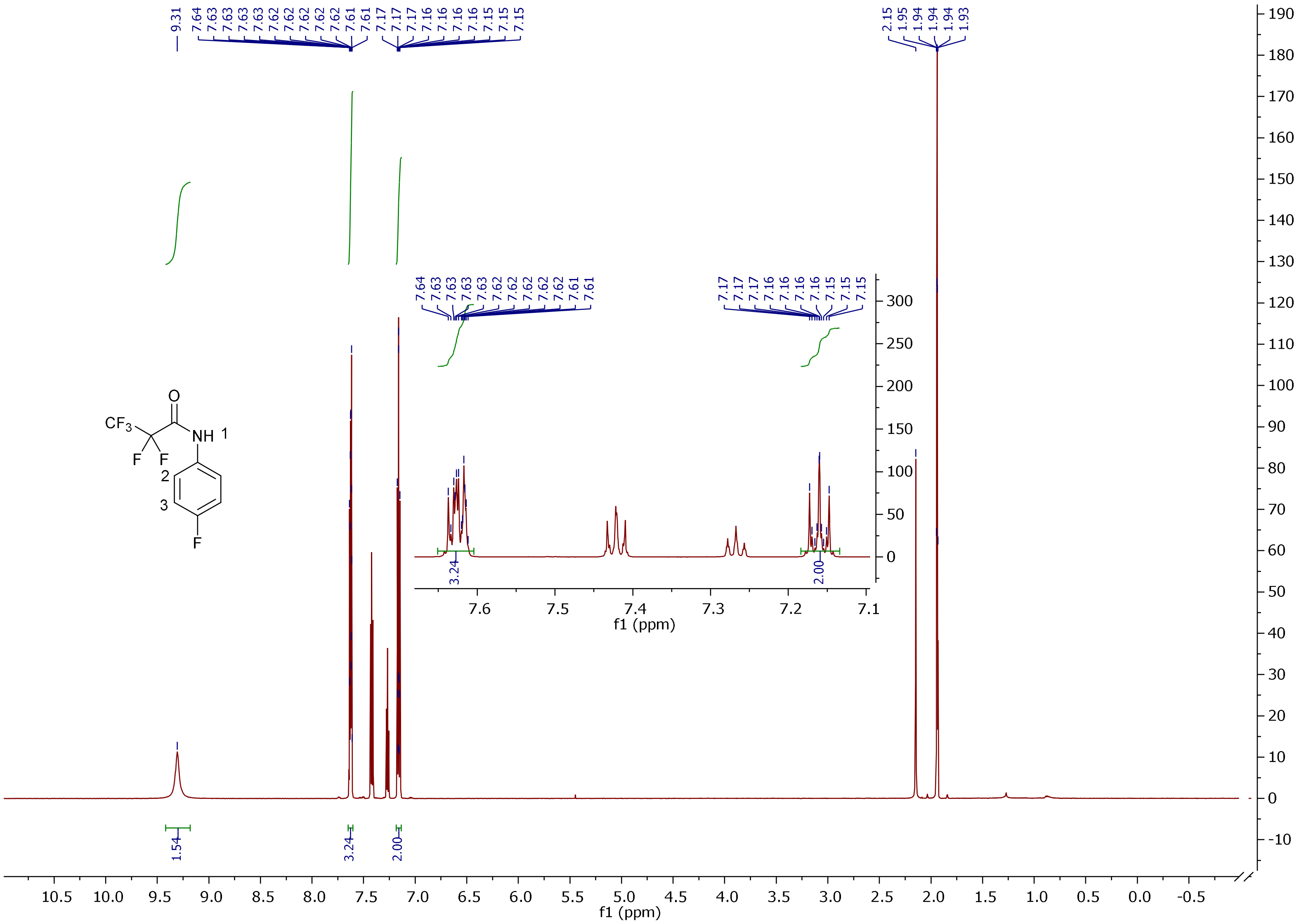

^{13}C NMR (176 MHz, CD_3CN) for fluorination of 2,2,3,3,3-pentafluoro-*N*-phenylpropanamide **(3d)**

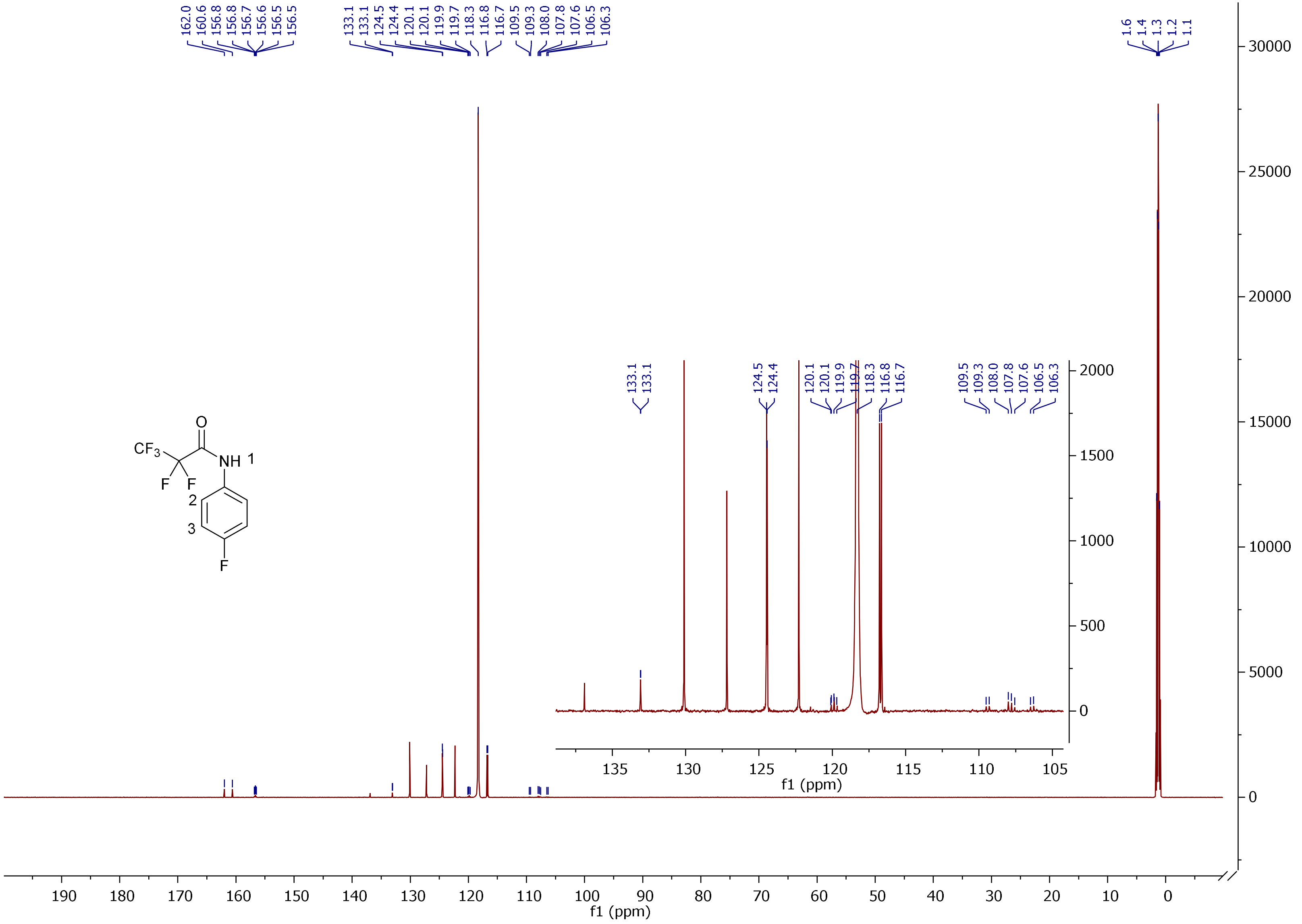

^{19}F{^{1}H} NMR (376 MHz, CD_3CN) for fluorination of 2,2,3,3,3-pentafluoro-*N*-phenylpropanamide **(3d)**

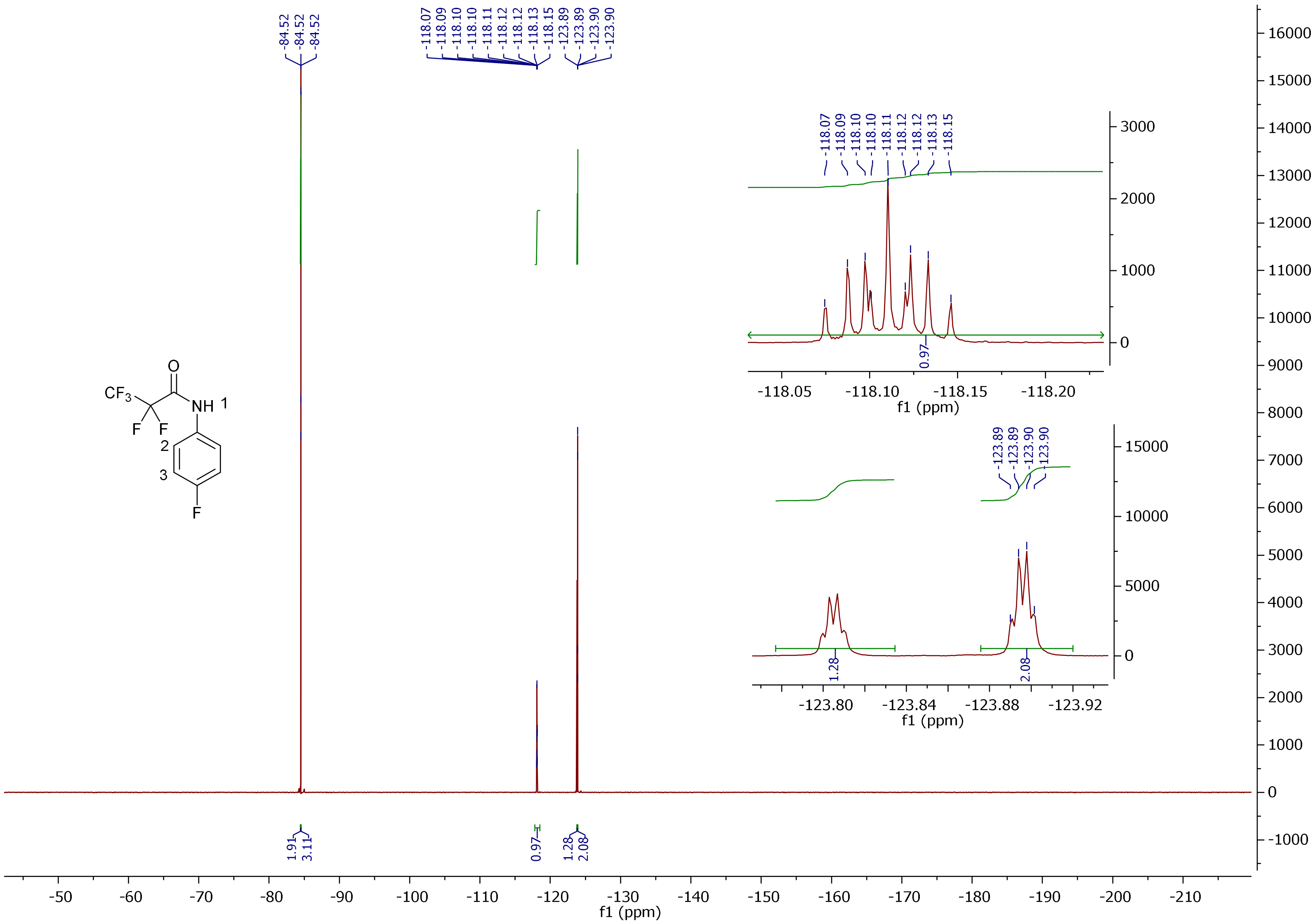

¹H NMR (700 MHz, CD_3CN) for fluorination of 2,2,3,3,4,4,4-heptafluoro-*N*-phenylbutanamide **(3e)**

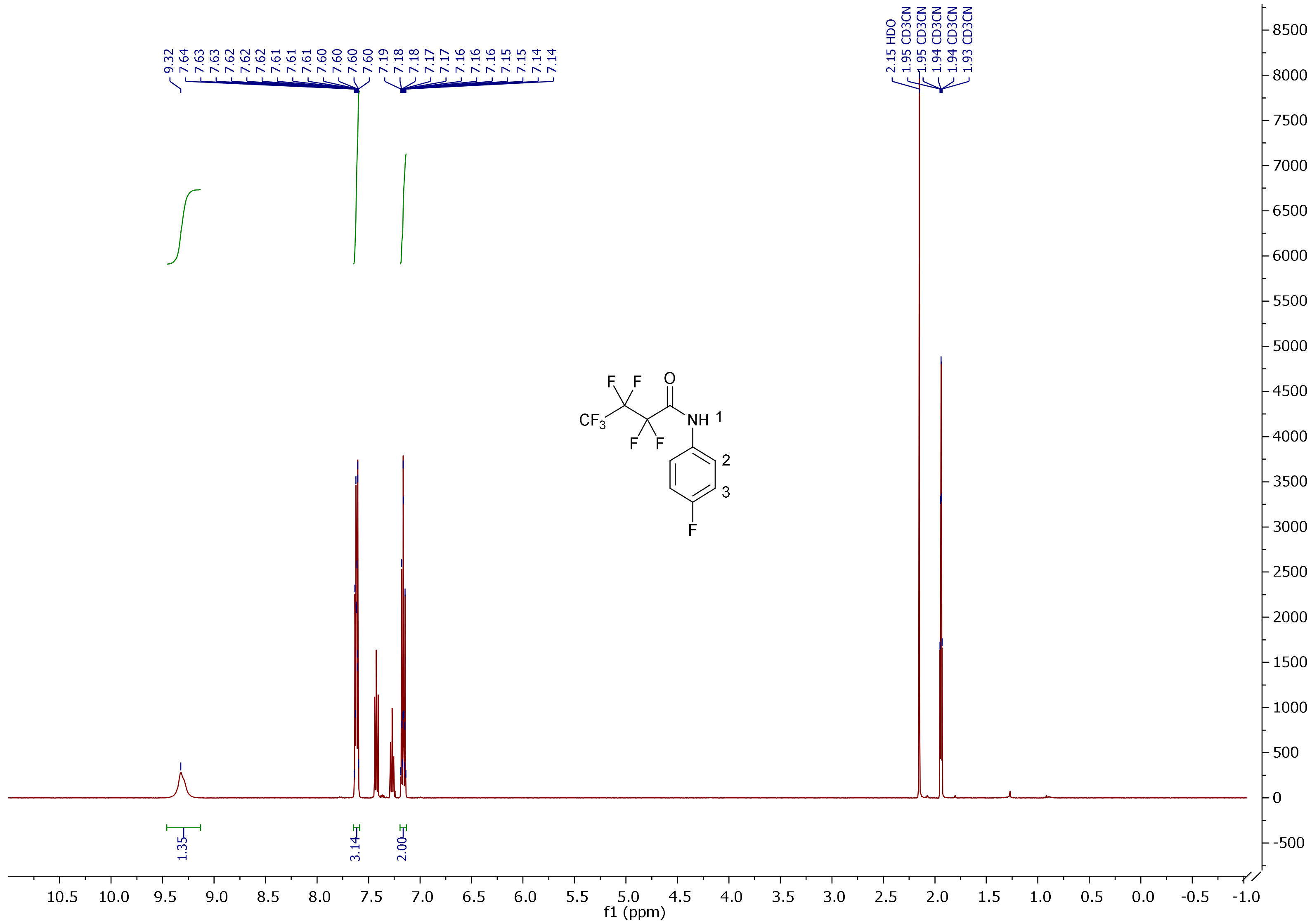

^{13}C NMR (176 MHz, CD_3CN) for fluorination of 2,2,3,3,4,4,4-heptafluoro-*N*-phenylbutanamide **(3e)**

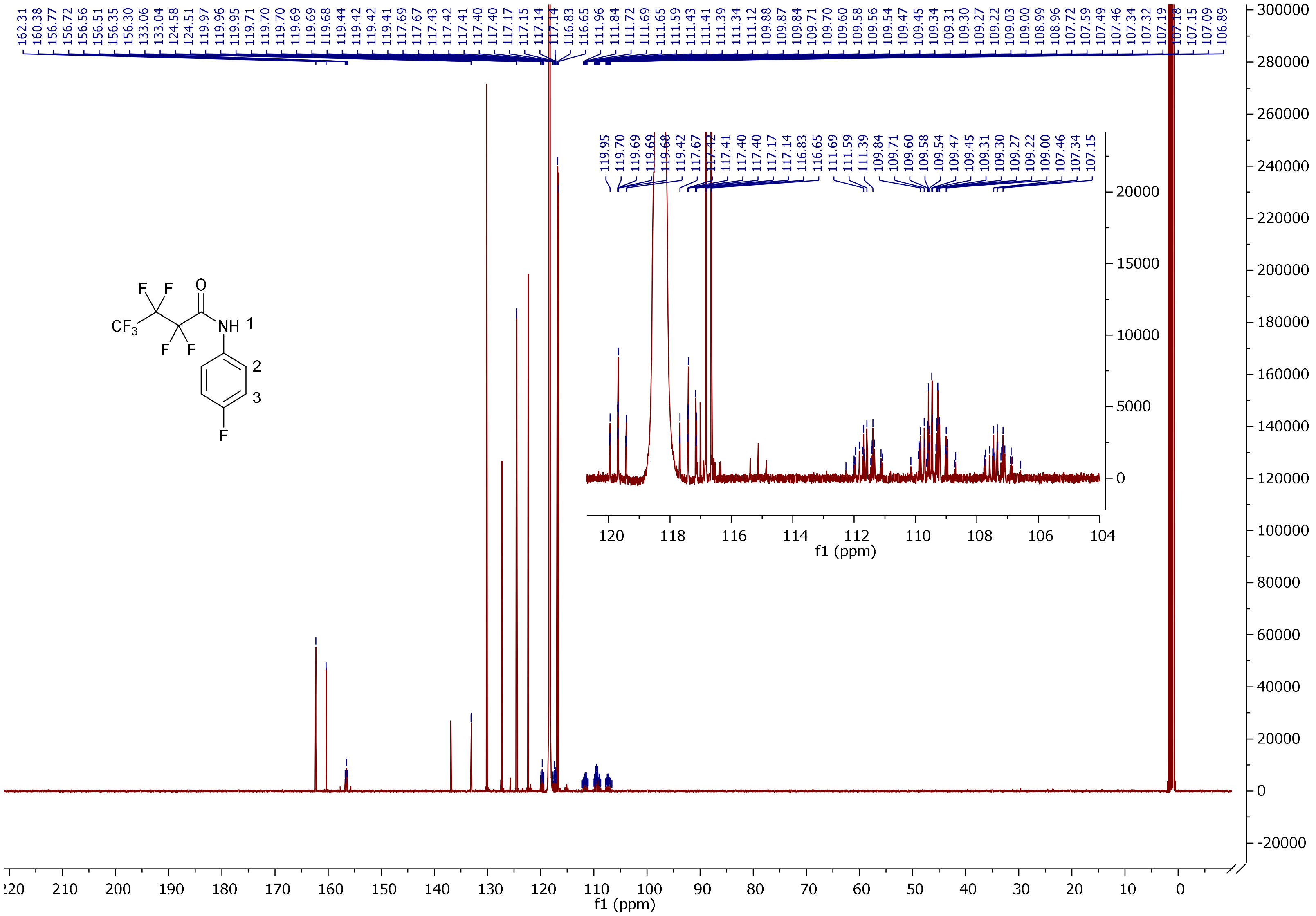

$^{19}F\{^1H\}$ NMR (376 MHz, CD_3CN) for fluorination of 2,2,3,3,4,4,4-heptafluoro-*N*-phenylbutanamide **(3e)**

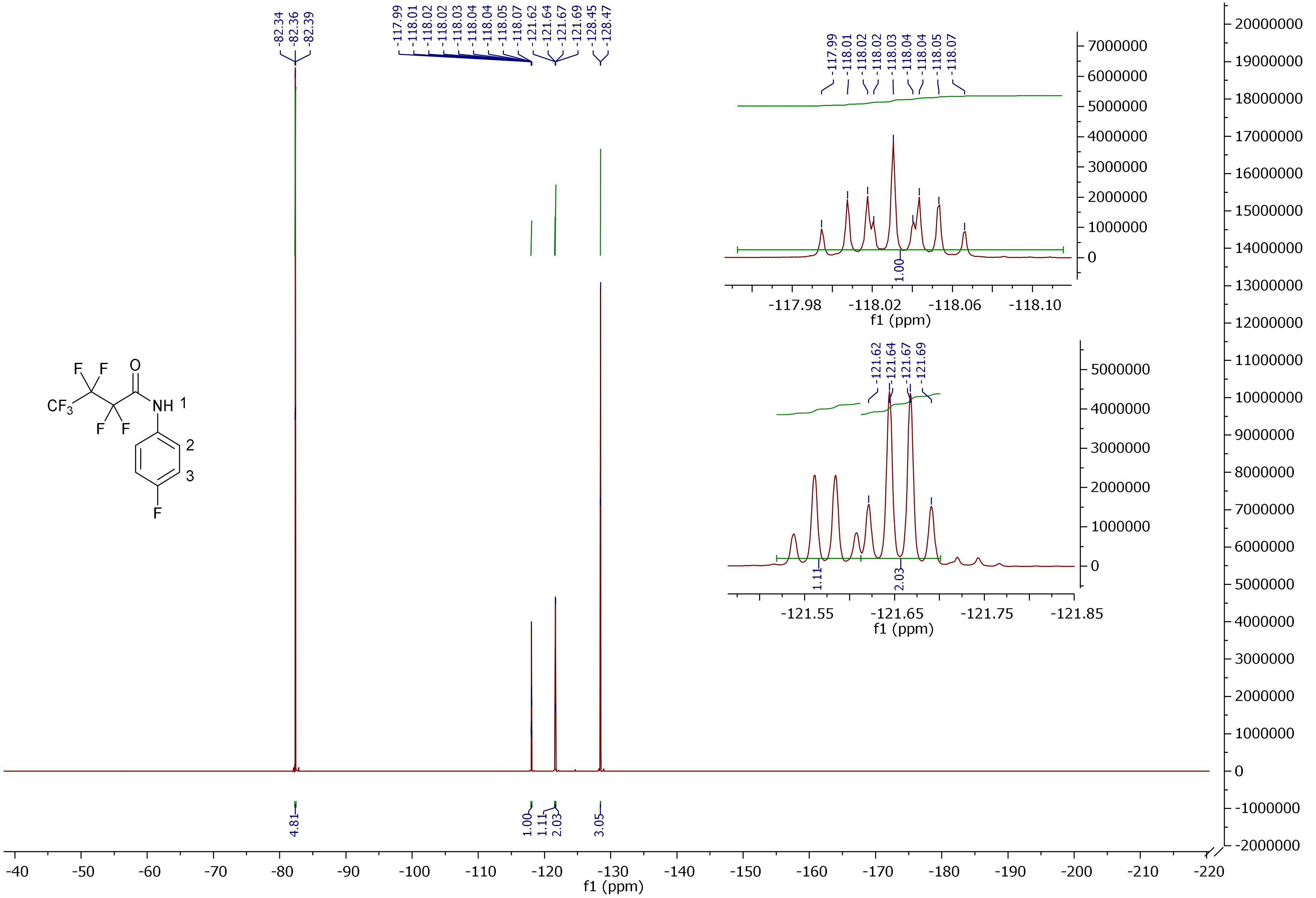

^{1}H NMR (700 MHz, CD_3CN) for fluorination of 2,3,4,5,6-pentafluoro-*N*-phenylbenzamide **(2f and 3f)**

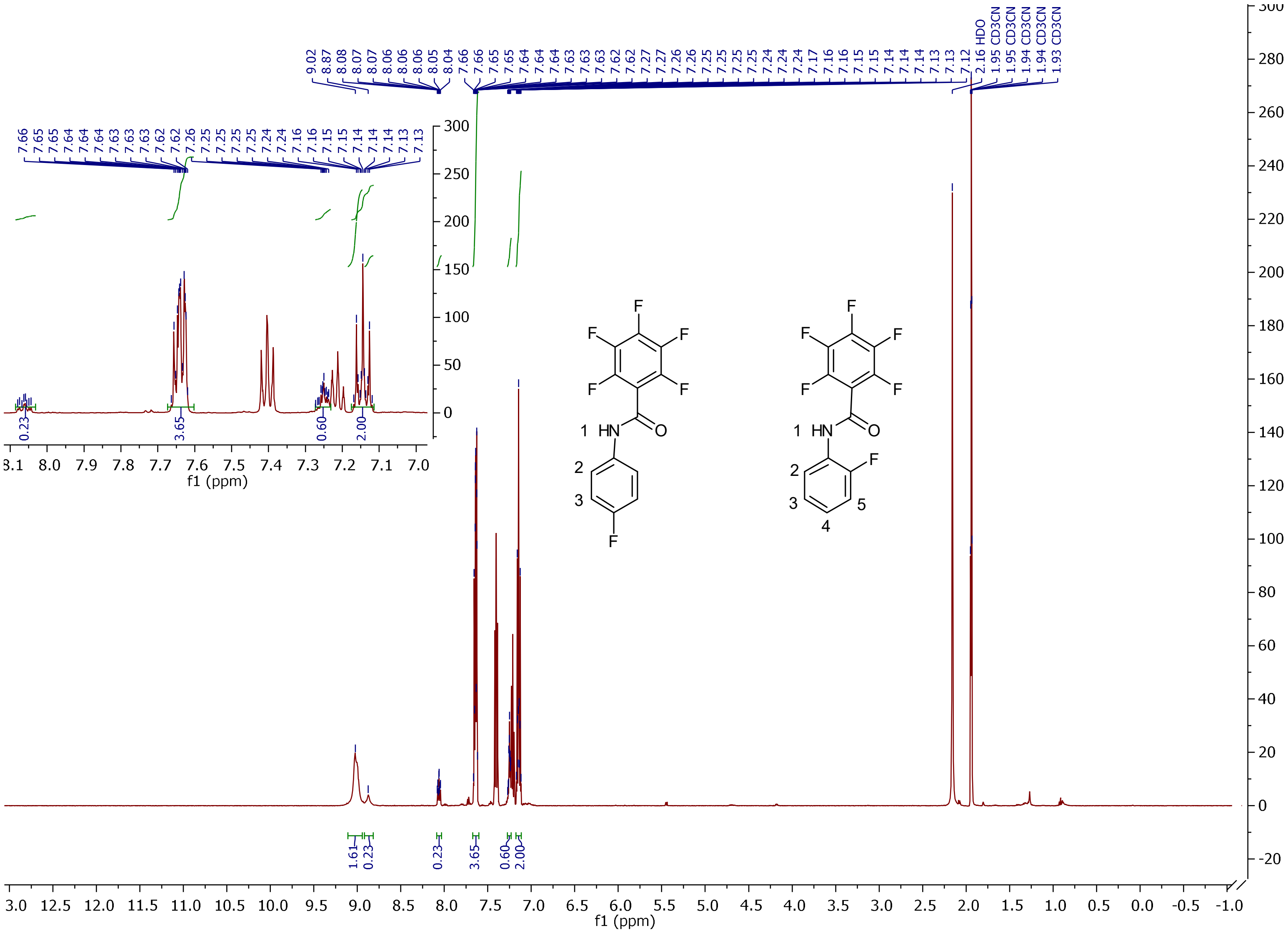

^{13}C NMR (176 MHz, CD_3CN) for fluorination of 2,3,4,5,6-pentafluoro-*N*-phenylbenzamide **(2f and 3f)**

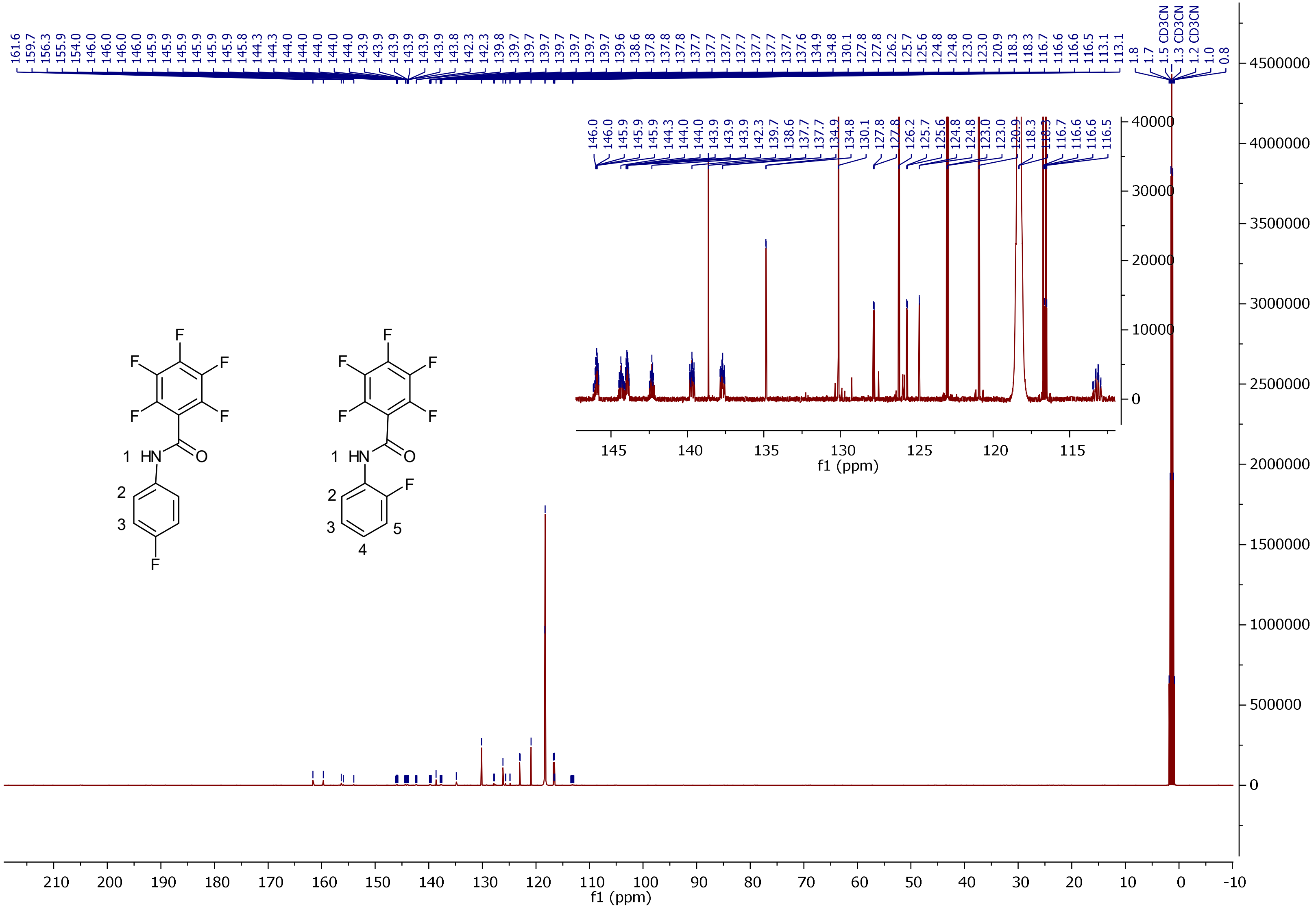

$^{19}F\{^{1}H\}$ **NMR** (376 MHz, CD_3CN) for fluorination of 2,3,4,5,6-pentafluoro-*N*-phenylbenzamide **(2f and 3f)**

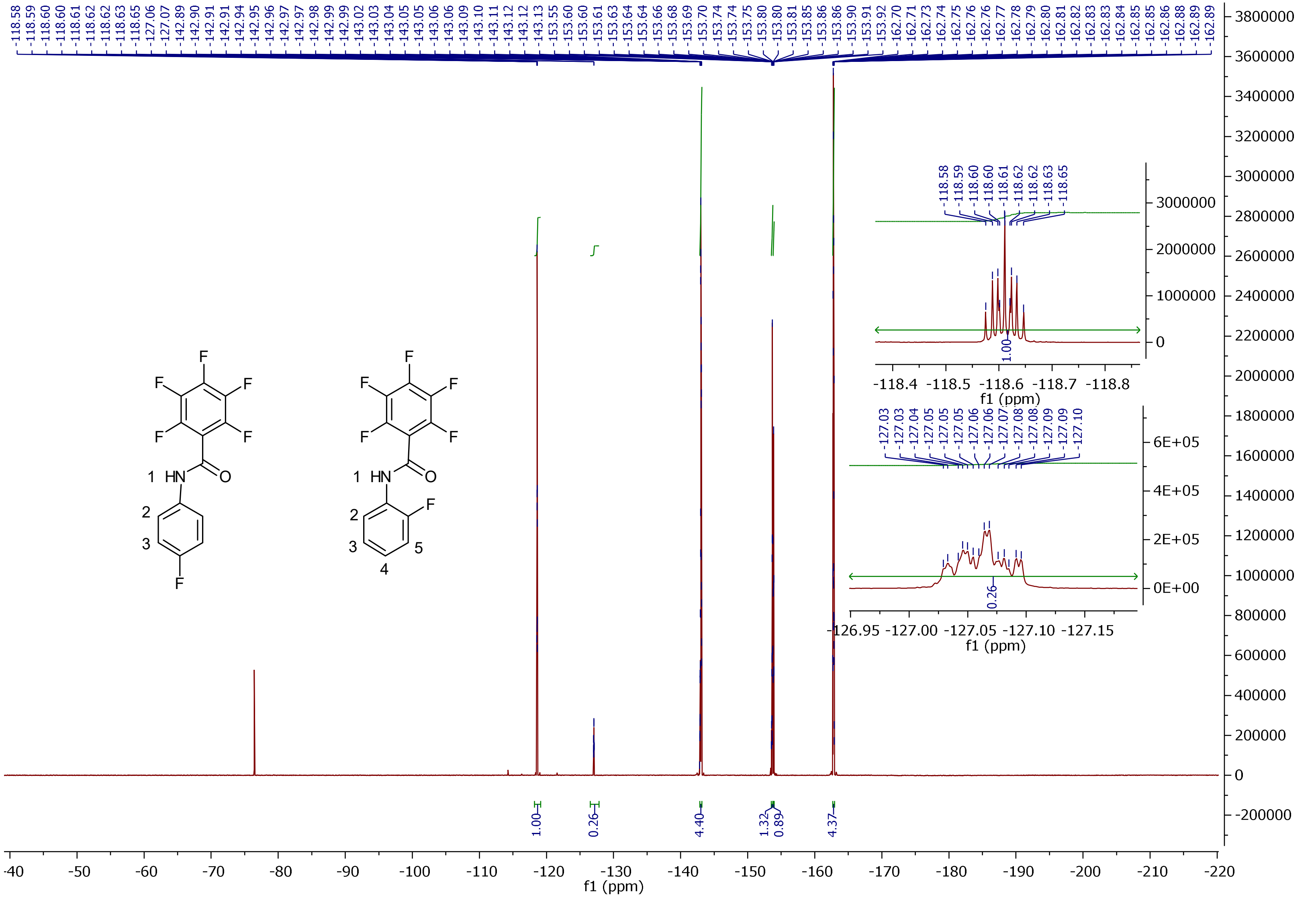

^{1}H NMR (700 MHz, CD_3CN) for fluorination of 2,2,2-trichloro-*N*-phenylacetamide **(3g)**

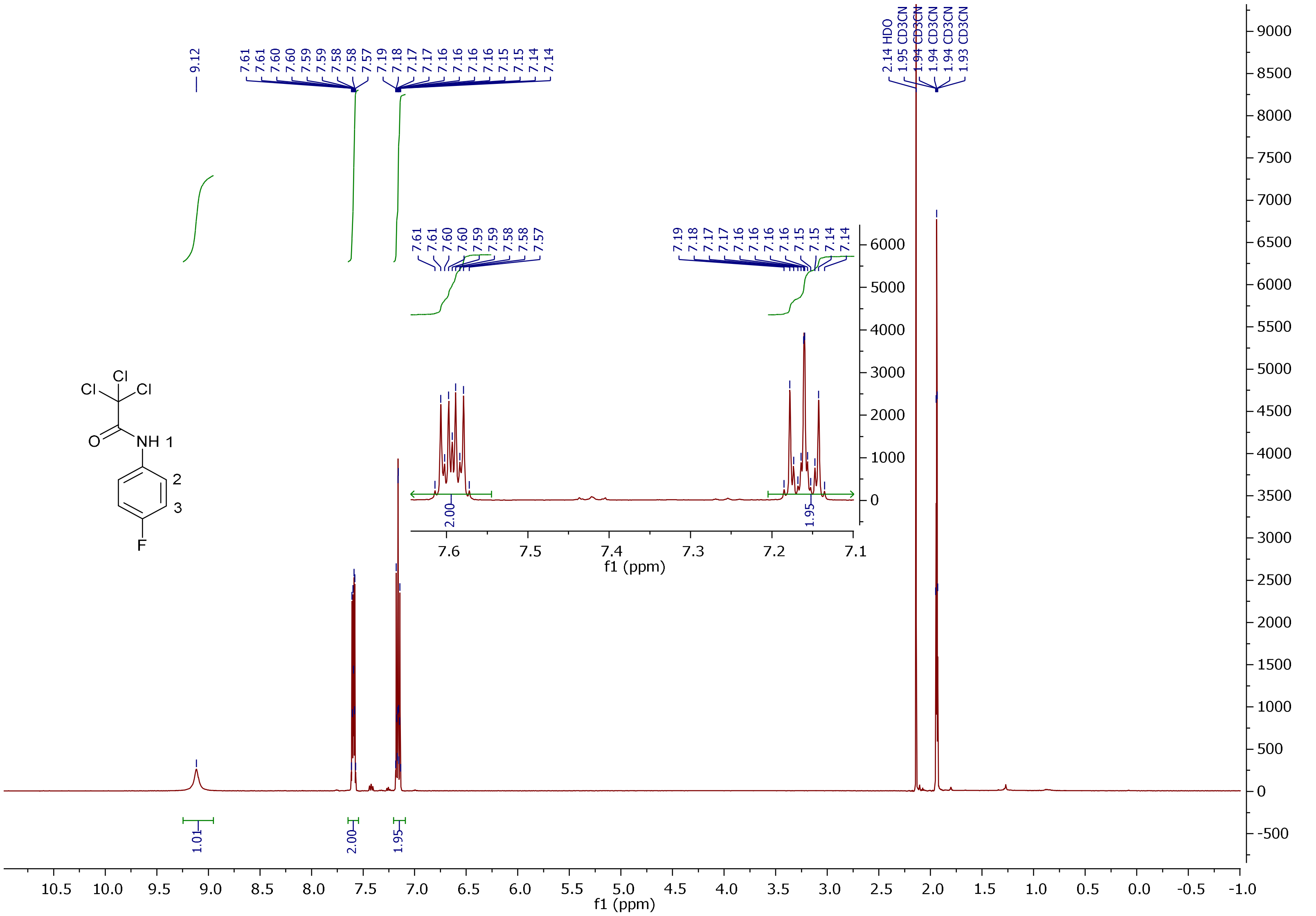

^{13}C NMR (176 MHz, CD_3CN) for fluorination of 2,2,2-trichloro-*N*-phenylacetamide **(3g)**

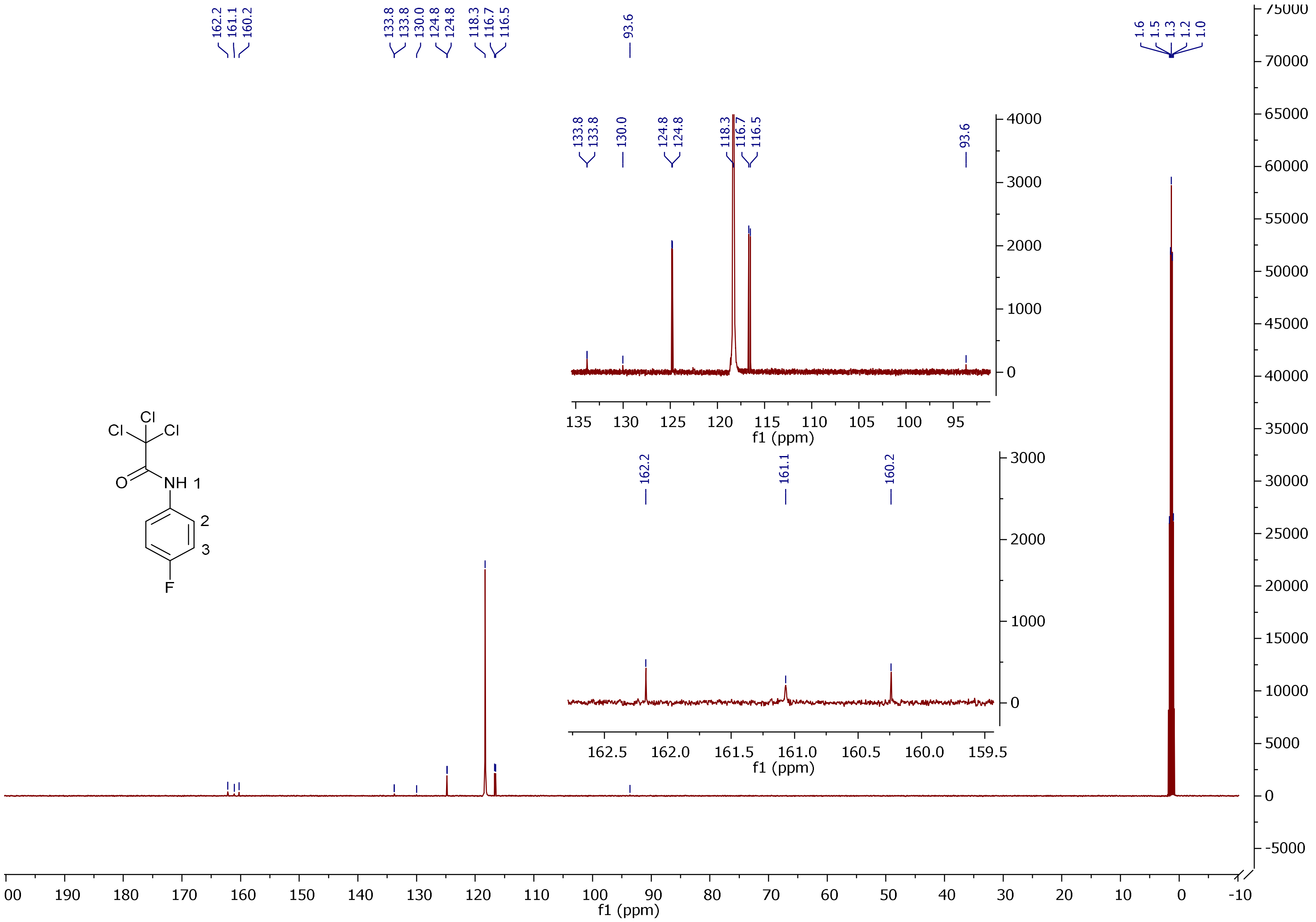

$^{19}F\{^{1}H\}$ NMR (376 MHz, CD_3CN) for fluorination of 2,2,2-trichloro-*N*-phenylacetamide **(3g)**

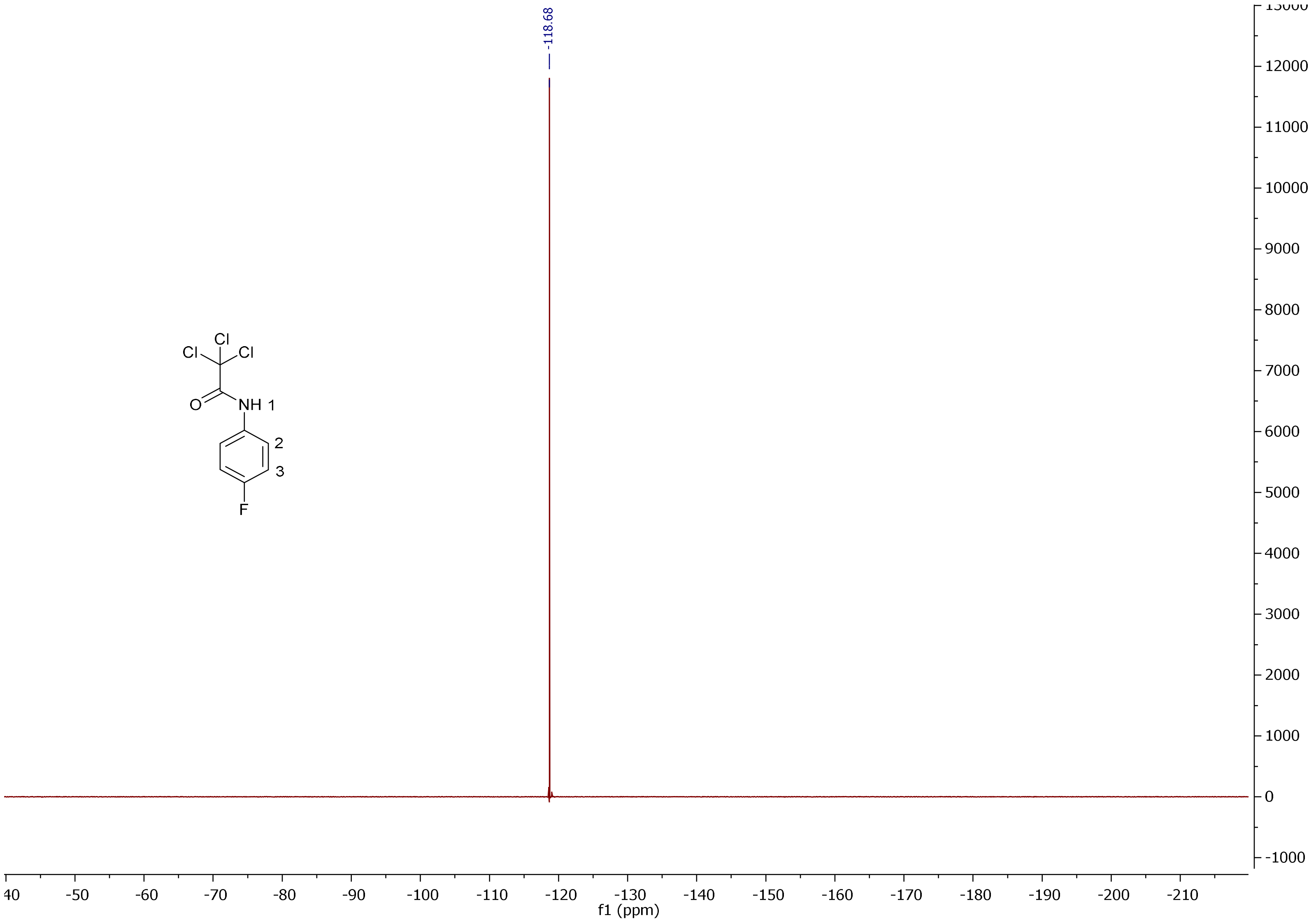

^{1}H NMR (700 MHz, CD_3CN) for fluorination of 2,2,2-trichloroethyl phenylcarbamate **(3h)**

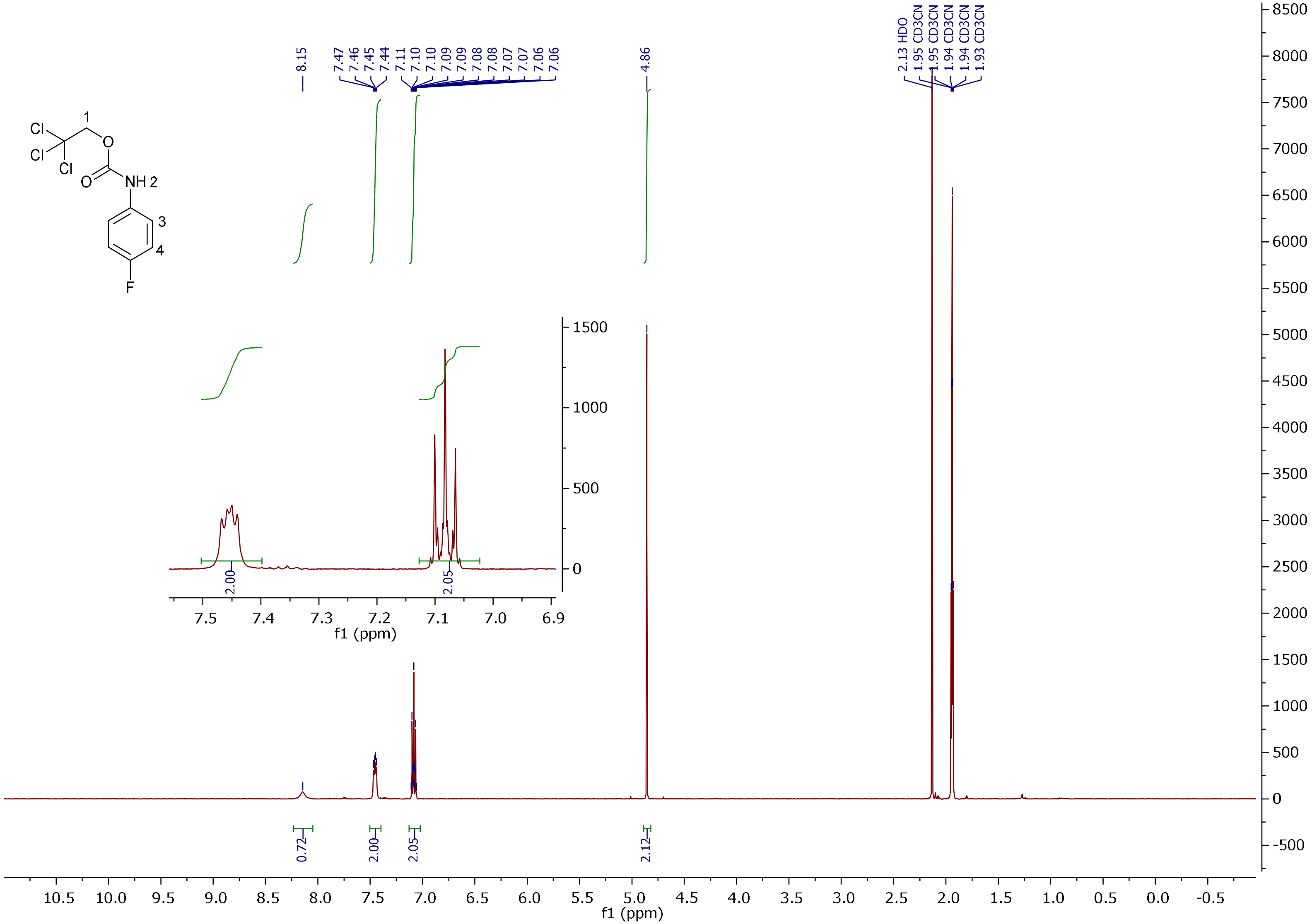

^{13}C NMR (176 MHz, CD_3CN) for fluorination of 2,2,2-trichloroethyl phenylcarbamate **(3h)**

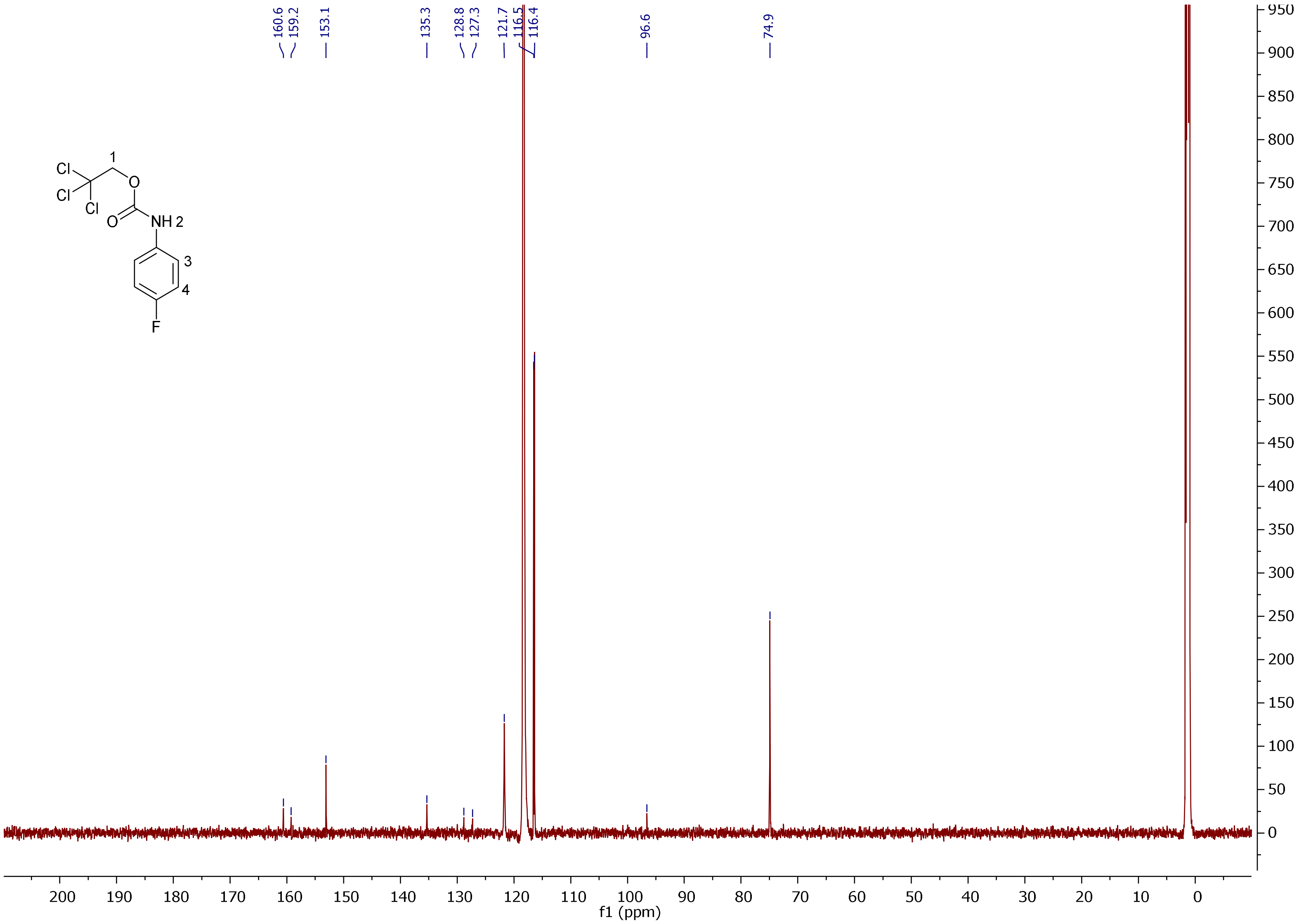

$^{19}F\{^{1}H\}$ NMR (376 MHz, CD_3CN) for fluorination of 2,2,2-trichloroethyl phenylcarbamate **(3h)**

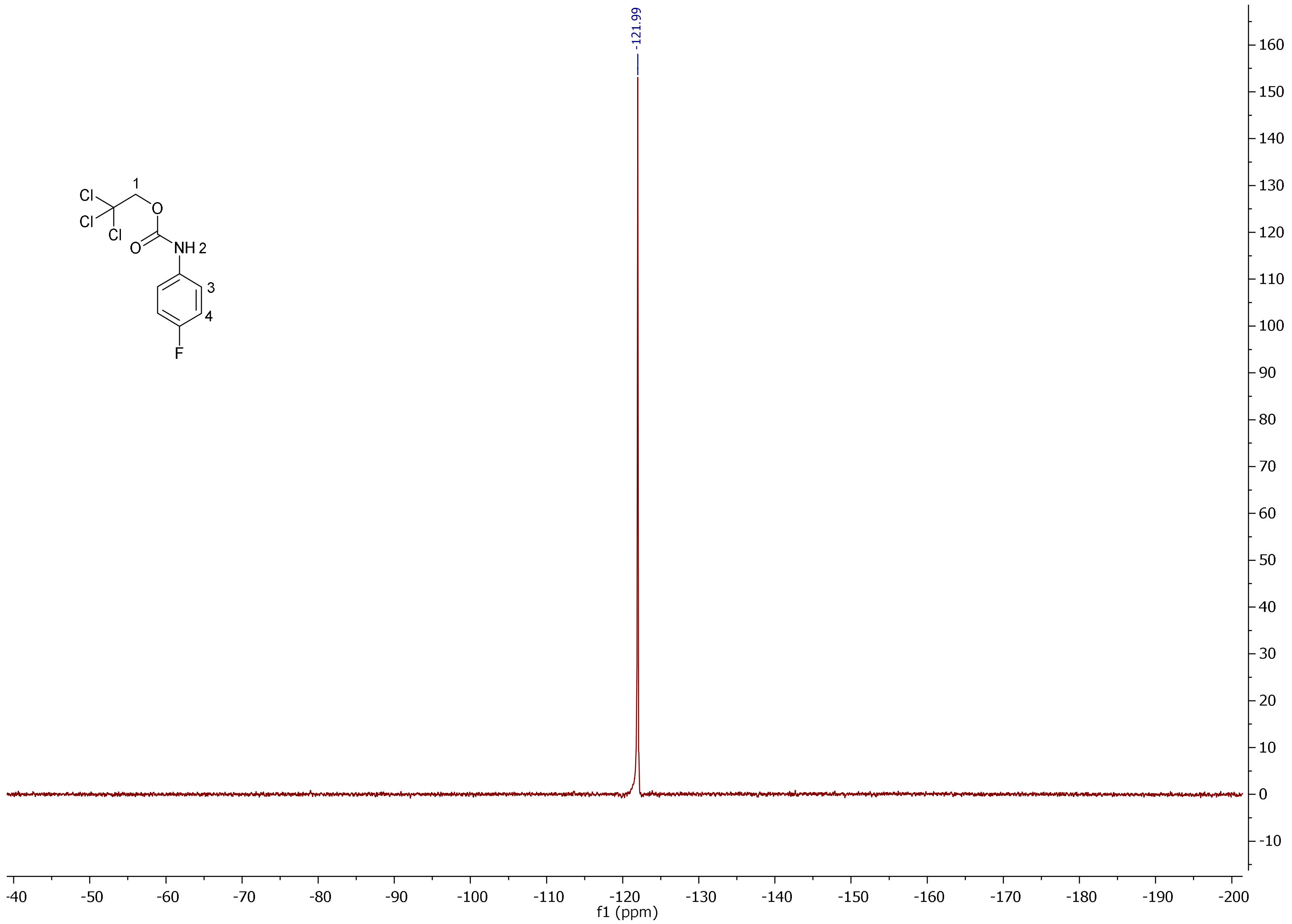

^{1}H NMR (700 MHz, CD_3CN) for fluorination of 2,2,2-trifluoro-*N*-(*o*-tolyl)acetamide **(3i)**

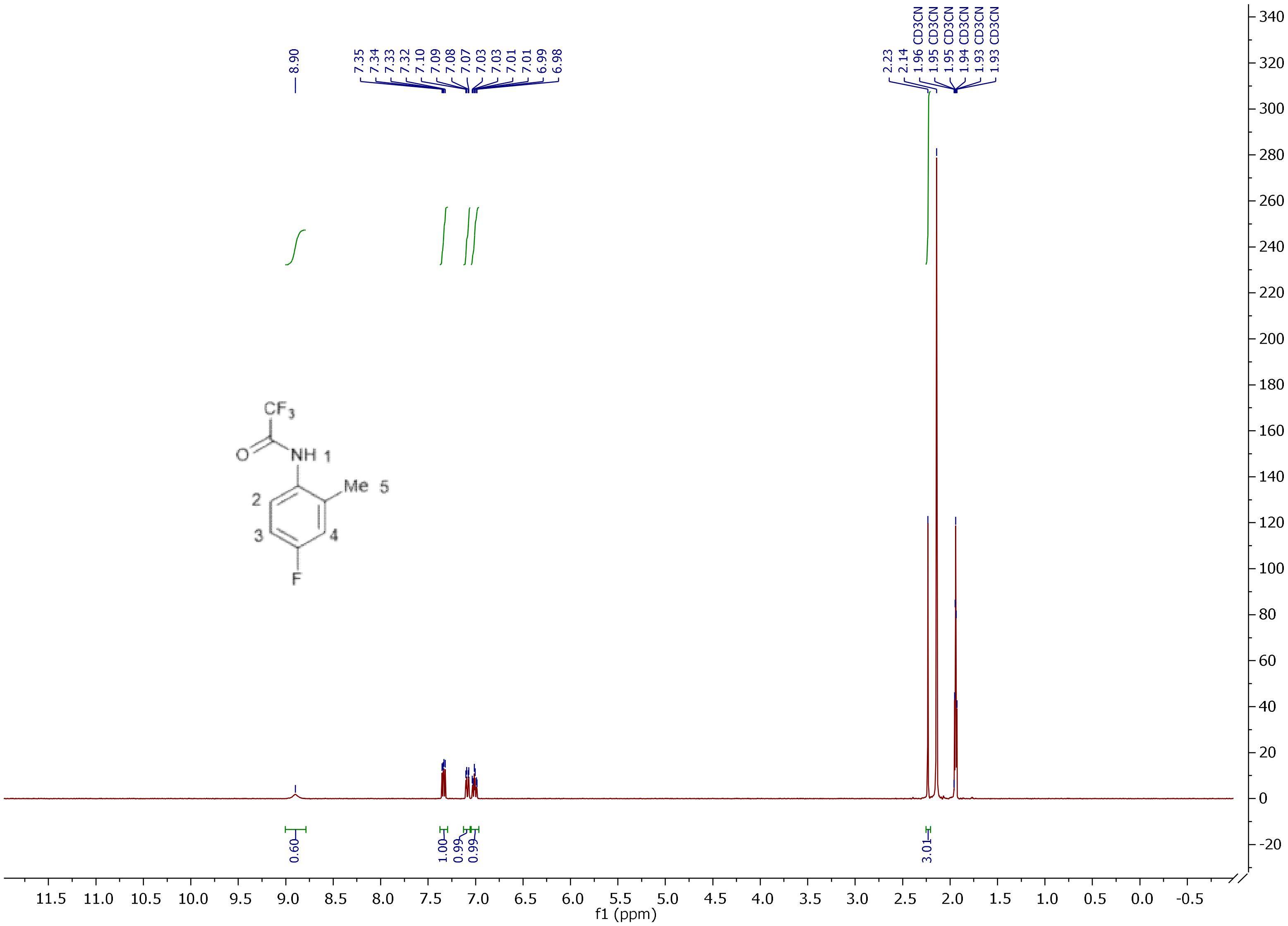

^{13}C NMR (176 MHz, CD_3CN) for fluorination of 2,2,2-trifluoro-*N*-(*o*-tolyl)acetamide **(3i)**

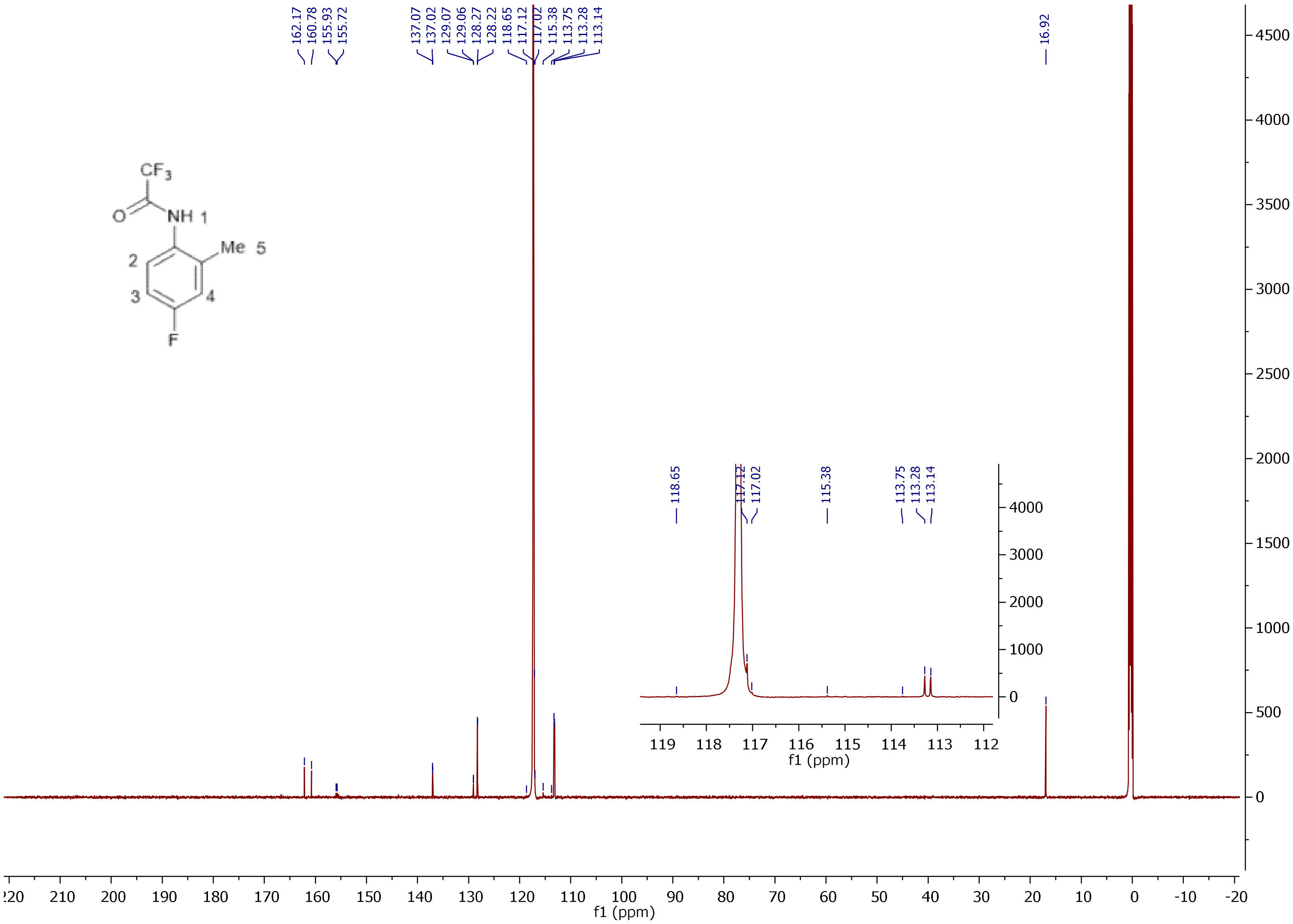

$^{19}F\{^1H\}$ NMR (376 MHz, CD_3CN) for fluorination of 2,2,2-trifluoro-*N*-(*o*-tolyl)acetamide **(3i)**

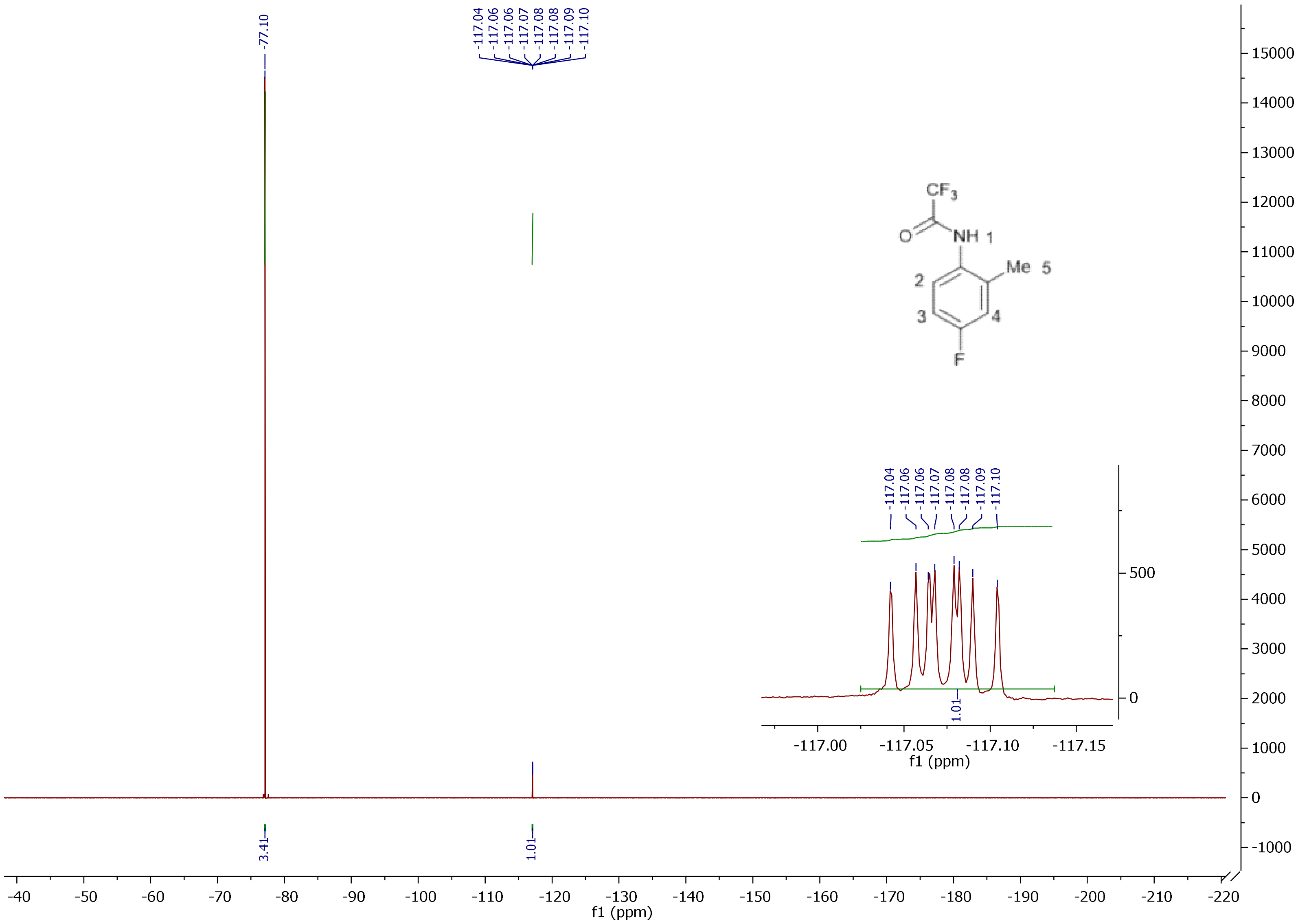

¹H NMR (700 MHz, CD_3CN) for 2,2,2-trifluoro-*N*-(2-fluoro-6-methylphenyl)acetamide **(2i)**

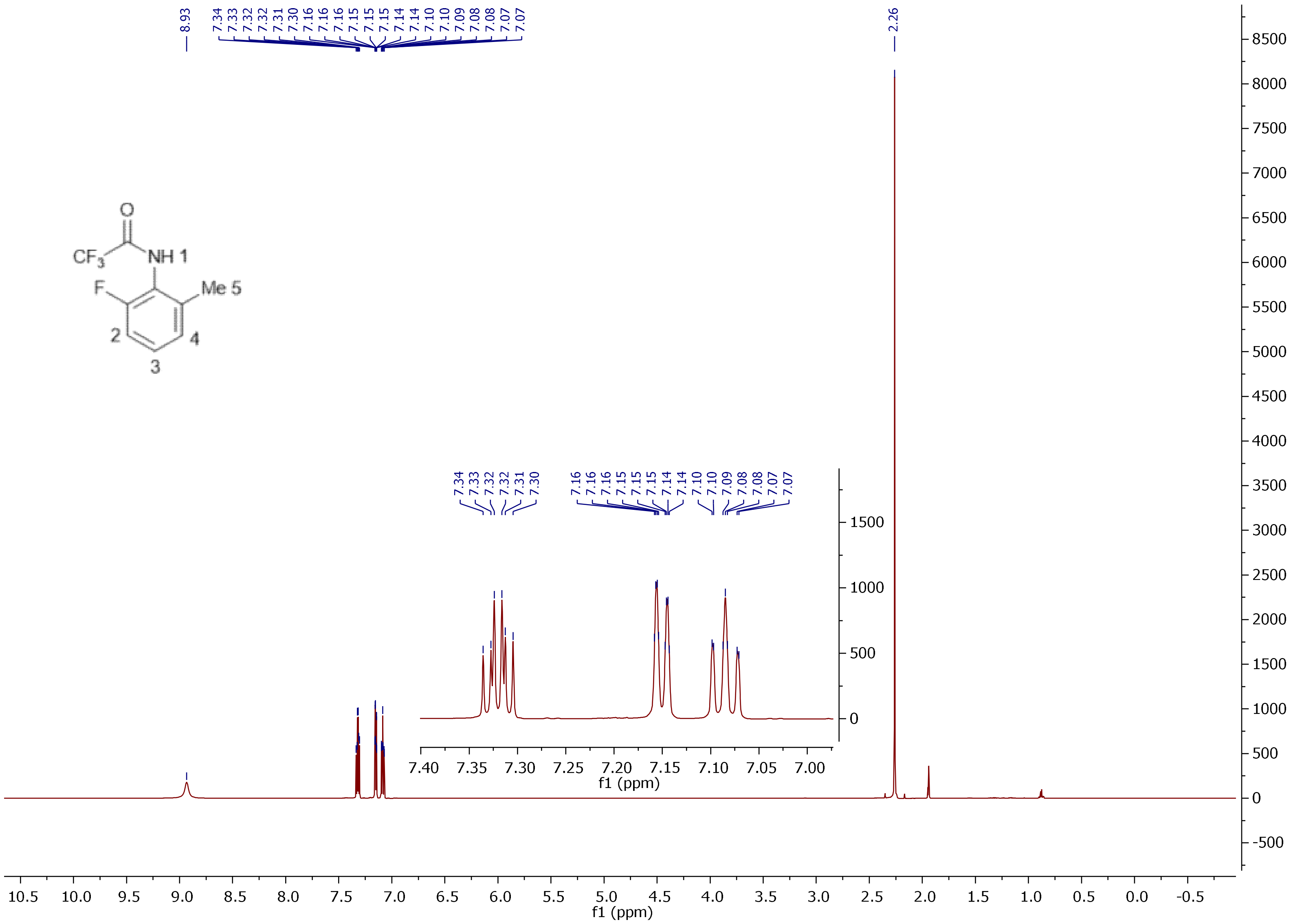

^{13}C NMR (176 MHz, CD_3CN) for 2,2,2-trifluoro-*N*-(2-fluoro-6-methylphenyl)acetamide **(2i)**

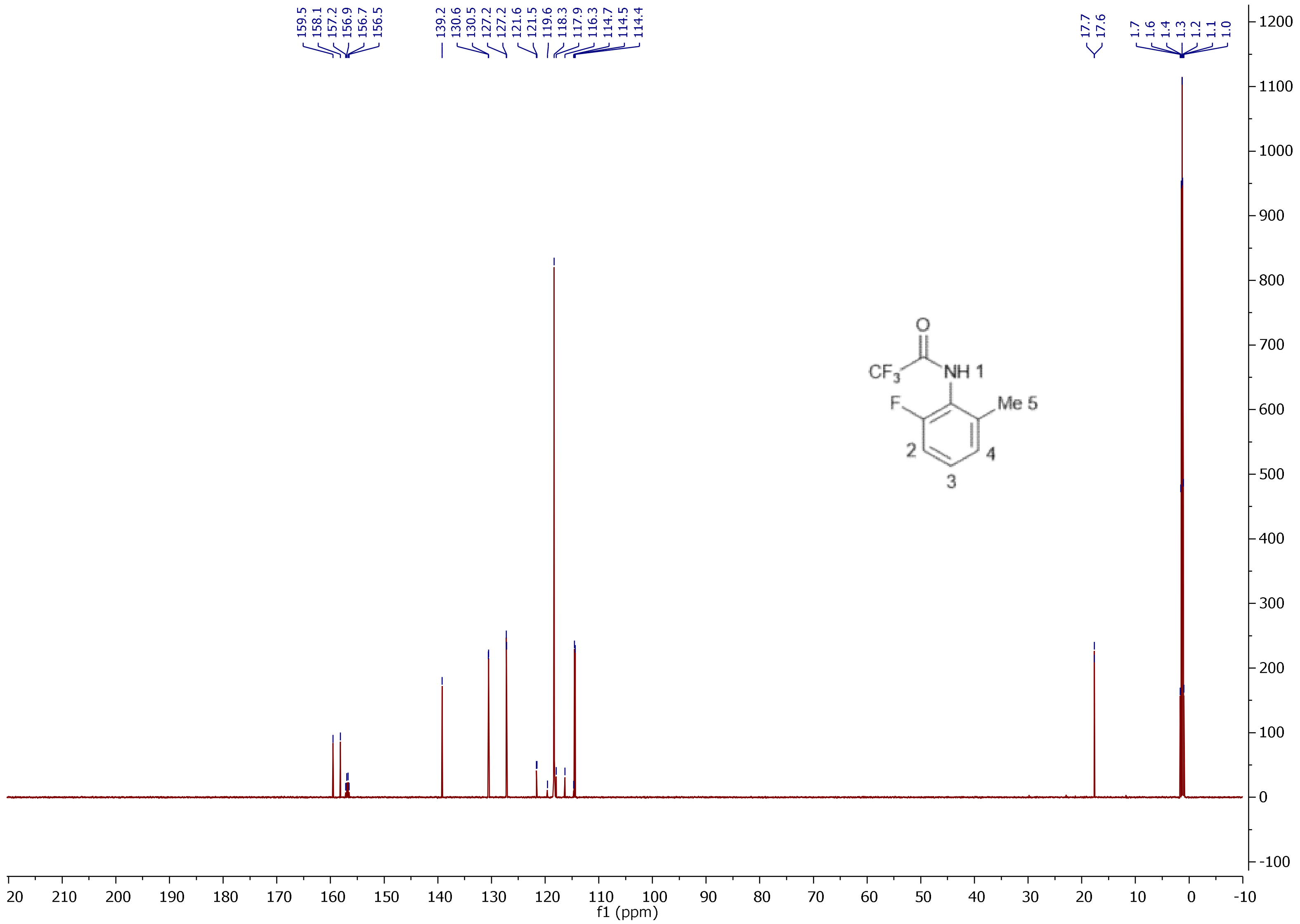

^{19}**F{^{1}H} NMR** (376 MHz, CD_3CN) for 2,2,2-trifluoro-*N*-(2-fluoro-6-methylphenyl)acetamide **(2i)**

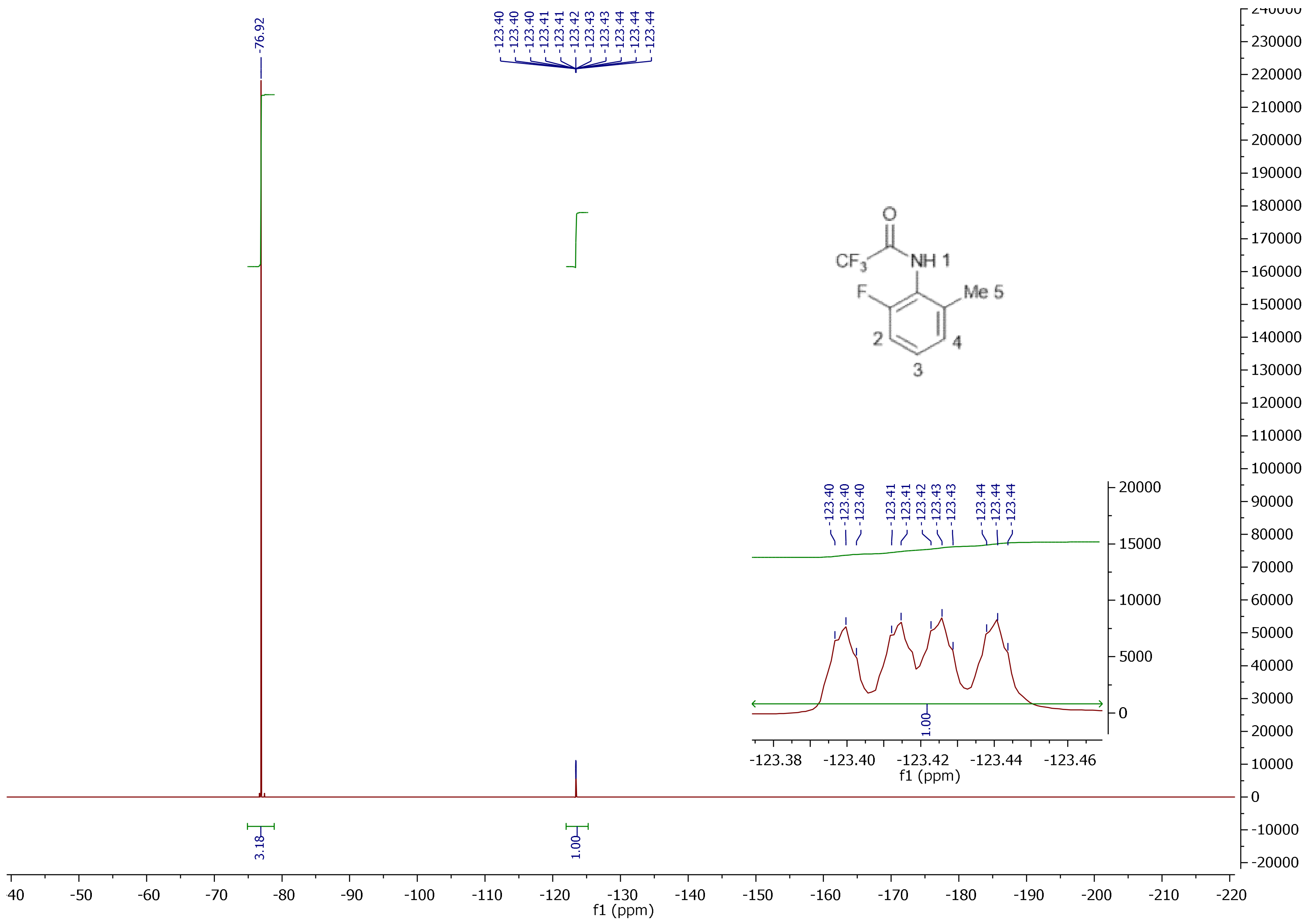

^{1}H NMR (700 MHz, CD_3CN) for fluorination of 2,2,2-trifluoro-*N*-(*m*-tolyl)acetamide **(3j)**

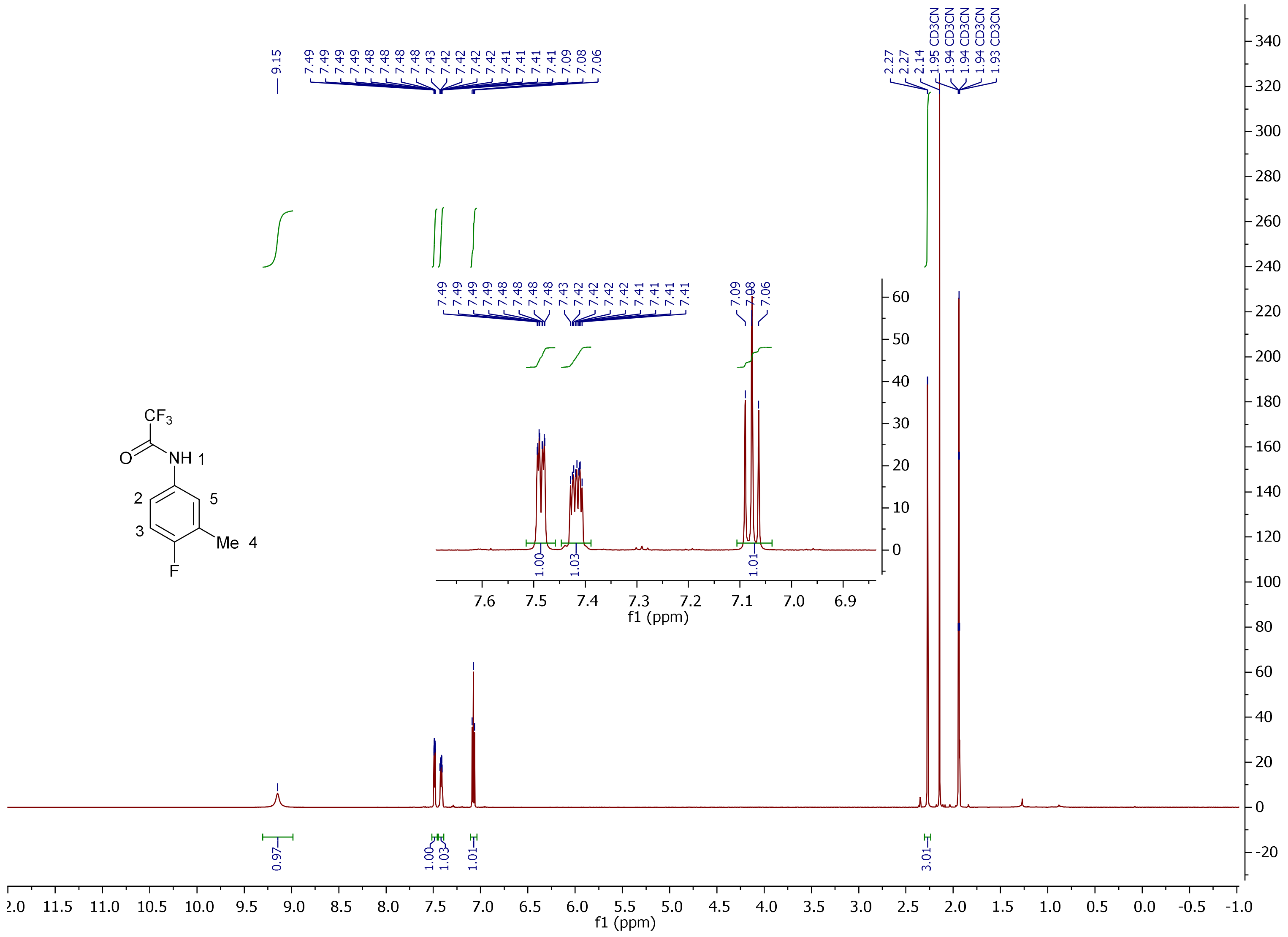

^{13}C NMR (176 MHz, CD_3CN) for fluorination of 2,2,2-trifluoro-*N*-(*m*-tolyl)acetamide **(3j)**

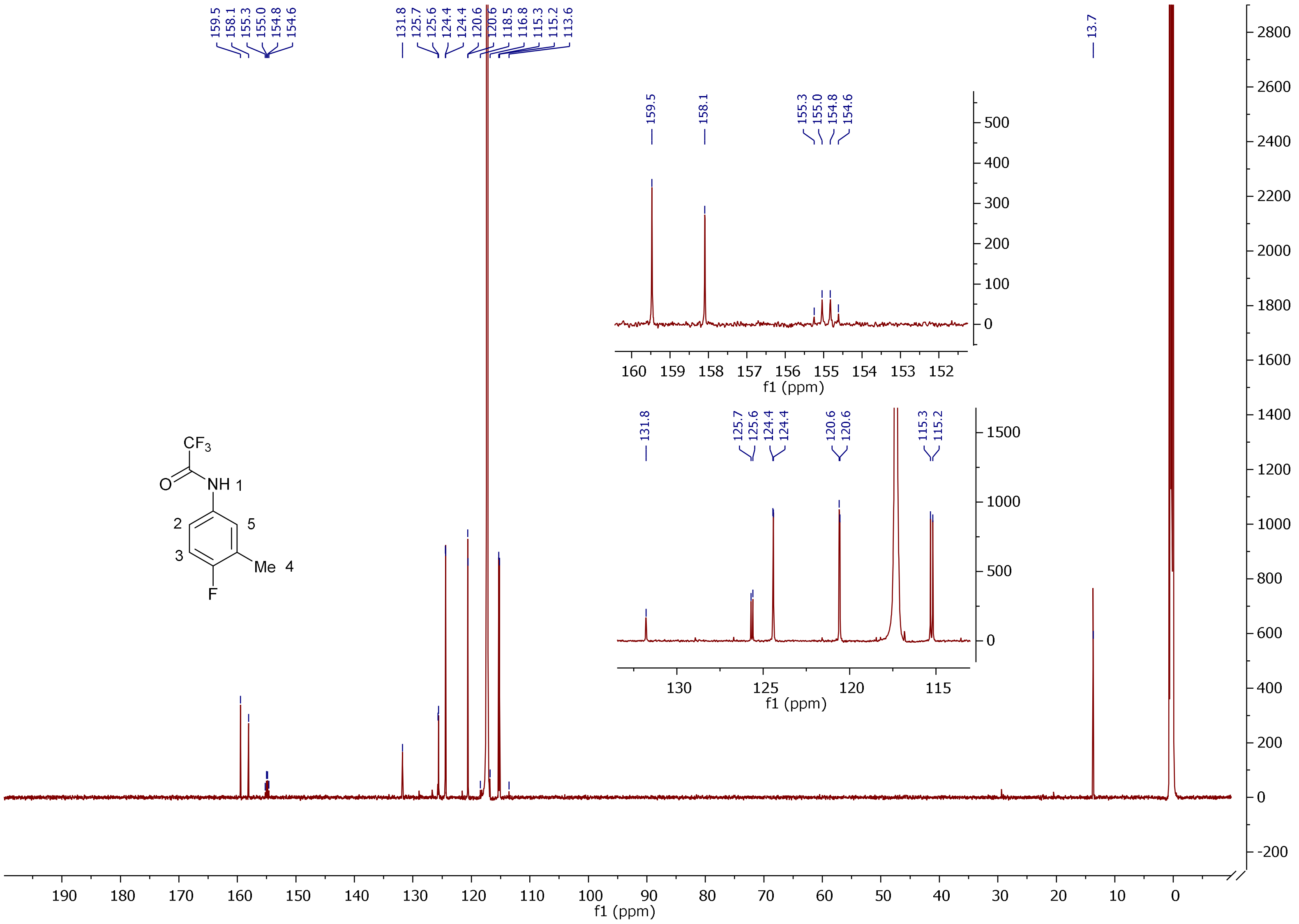

^{19}**F{^{1}H} NMR** (376 MHz, CD_3CN) for fluorination of 2,2,2-trifluoro-*N*-(*m*-tolyl)acetamide **(3j)**

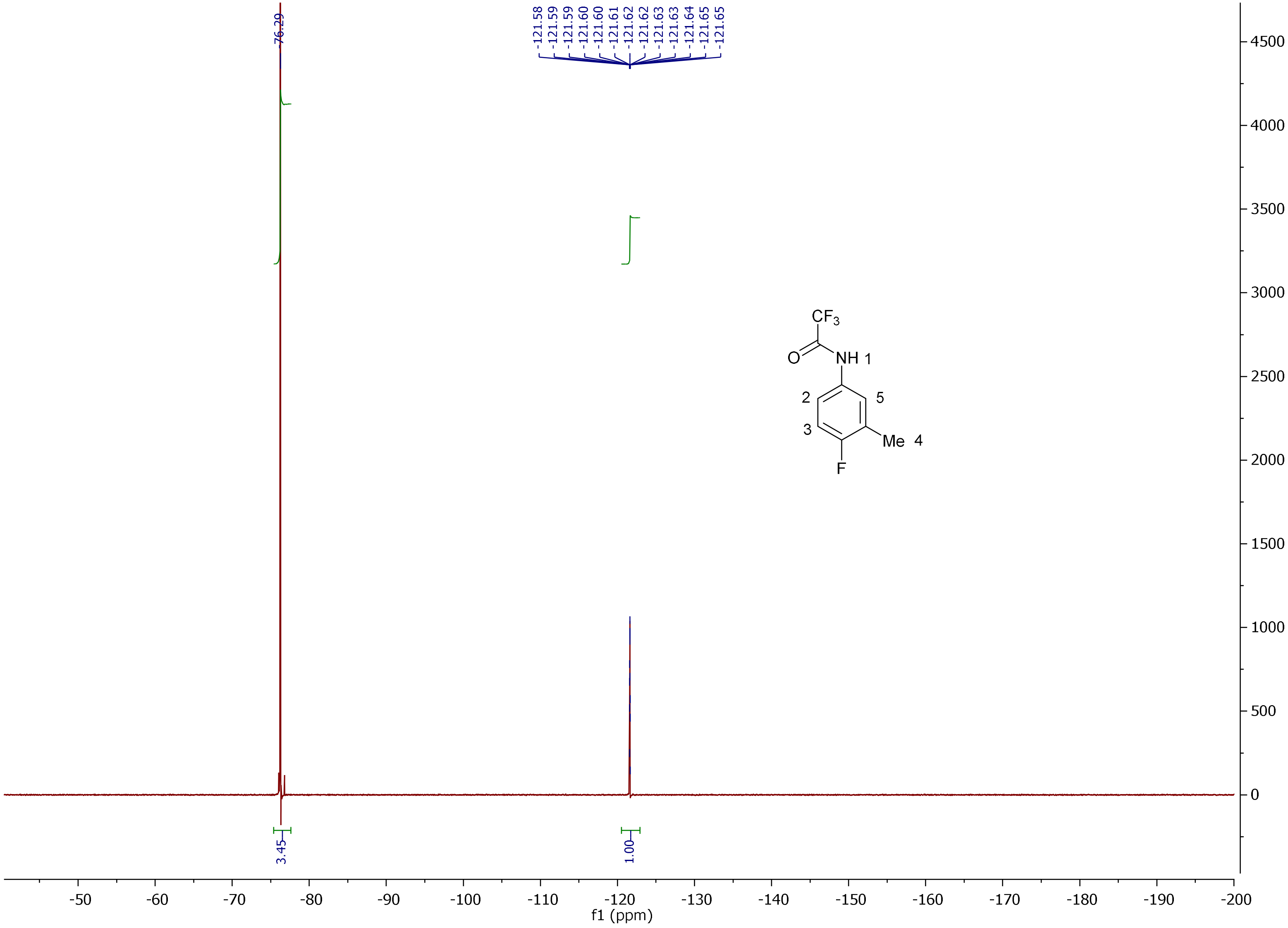

^{1}H NMR (700 MHz, CD_3CN) for 2,2,2-trifluoro-*N*-(2-fluoro-3-methylphenyl)acetamide **(2j)**

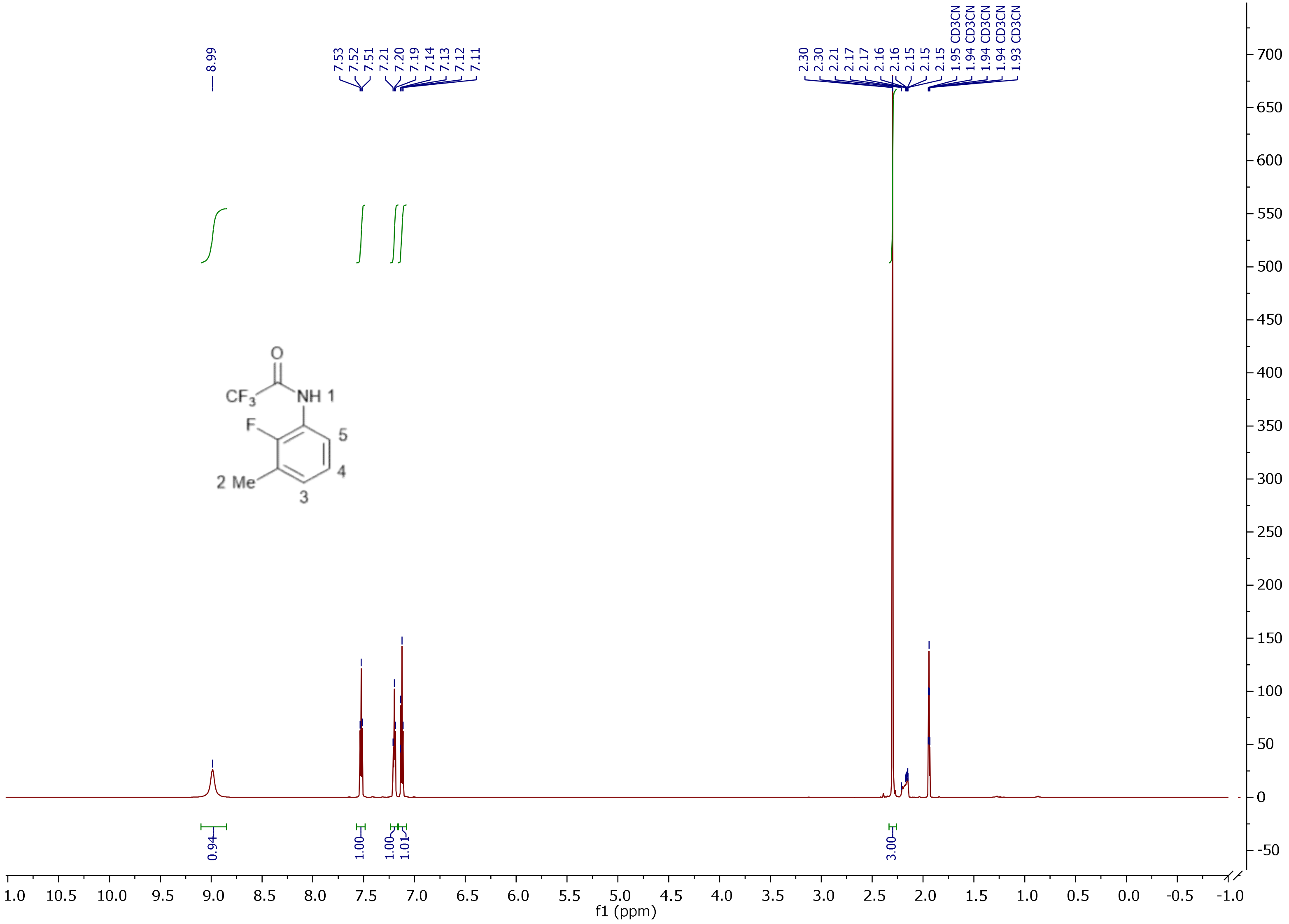

^{13}C NMR (176 MHz, CD_3CN) for 2,2,2-trifluoro-*N*-(2-fluoro-3-methylphenyl)acetamide **(2j)**

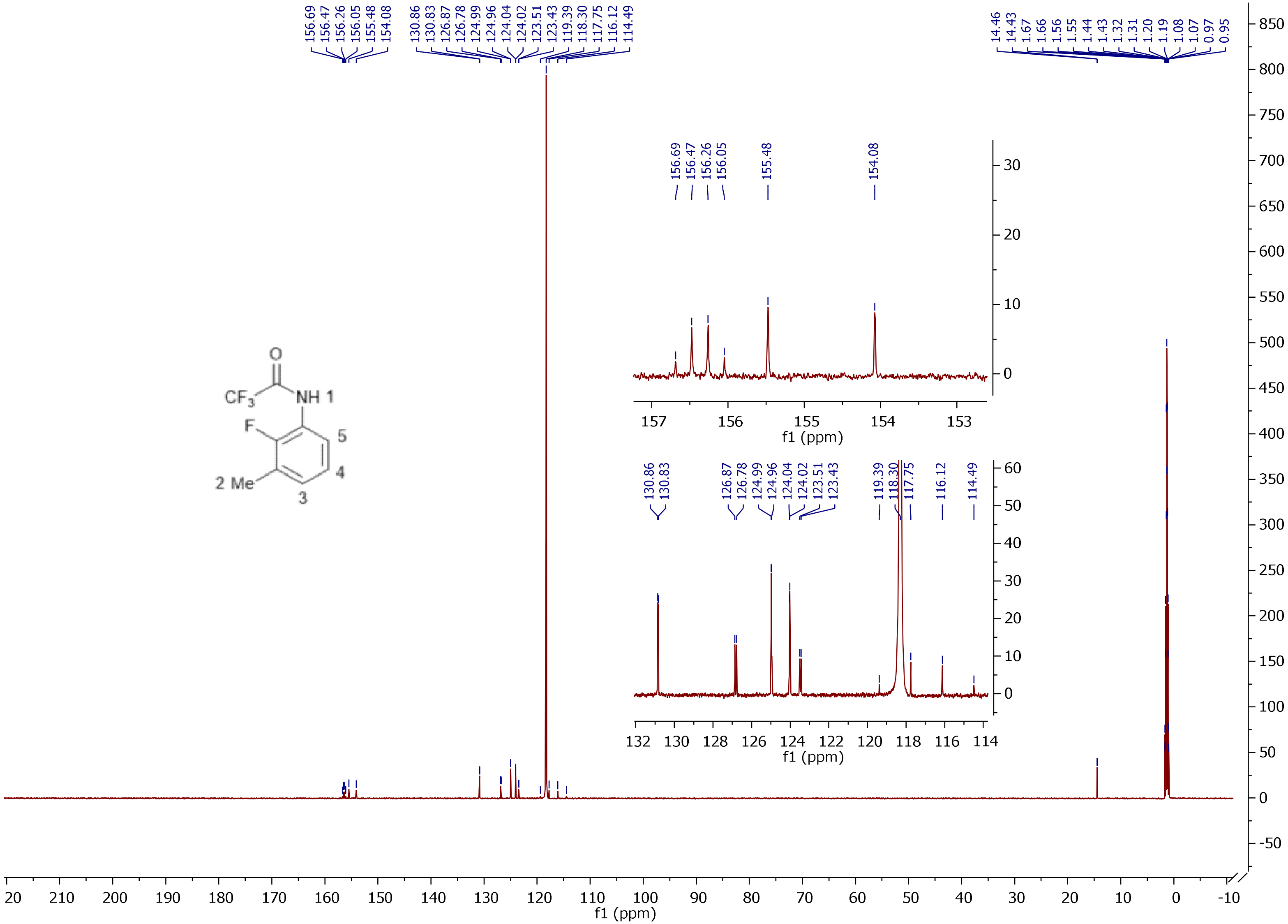

^{19}F{^{1}H} NMR (376 MHz, CD_3CN) for 2,2,2-trifluoro-*N*-(2-fluoro-3-methylphenyl)acetamide **(2j)**

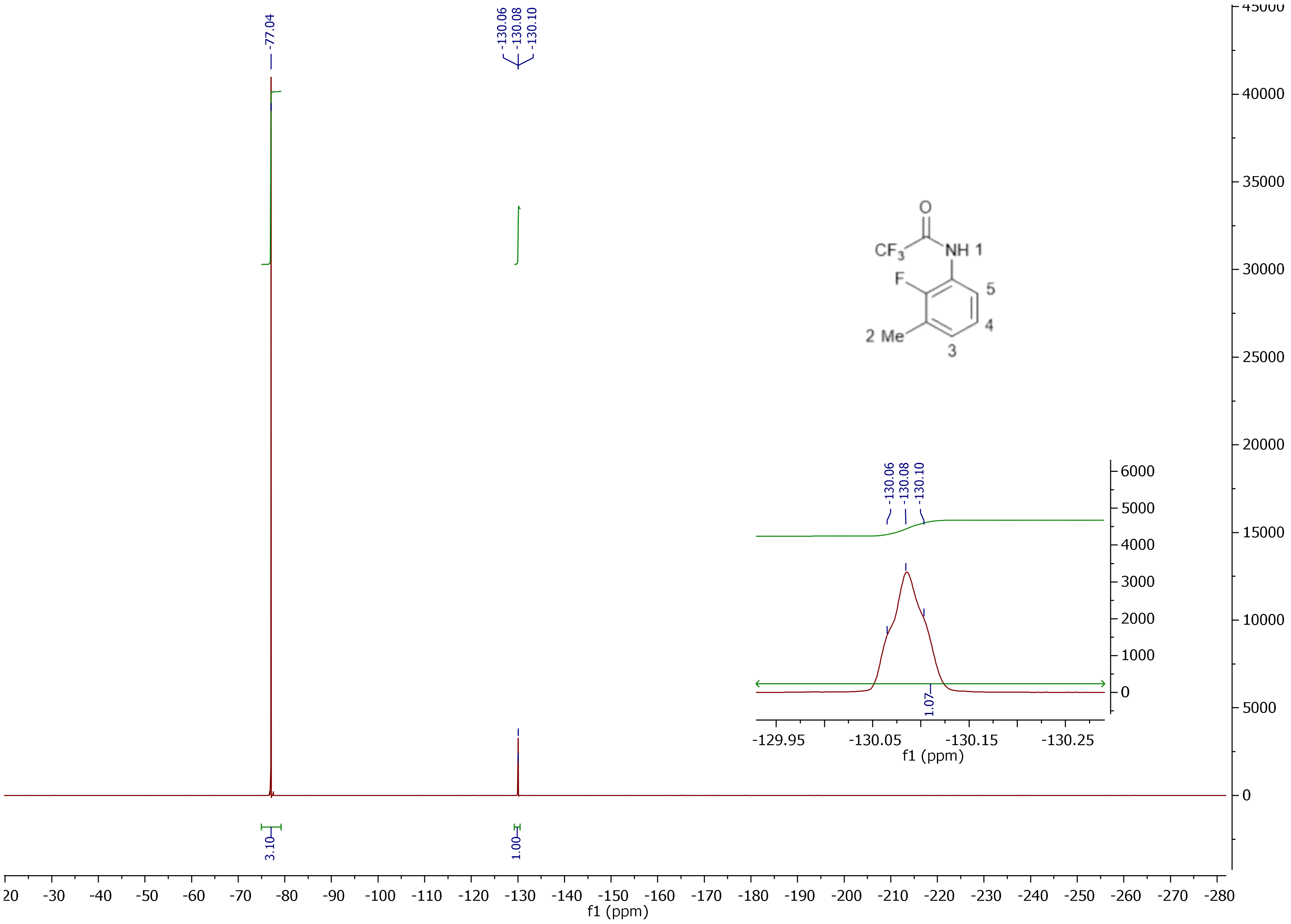

^{1}H NMR (700 MHz, CD_3CN) for 2,2,2-trifluoro-*N*-(2-fluoro-5-methylphenyl)acetamide **(2j')**

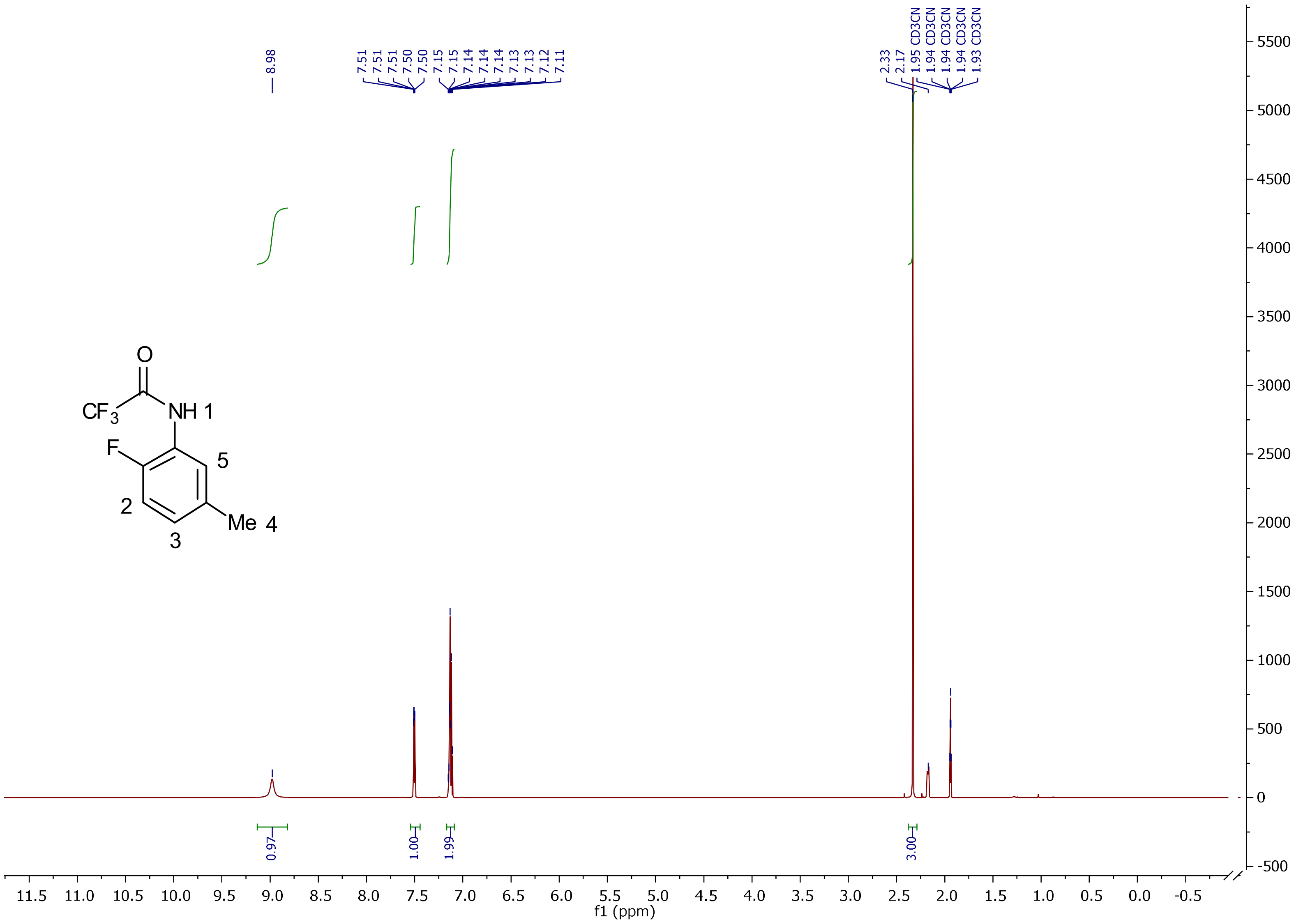

^{13}C NMR (176 MHz, CD_3CN) for 2,2,2-trifluoro-*N*-(2-fluoro-5-methylphenyl)acetamide **(2j')**

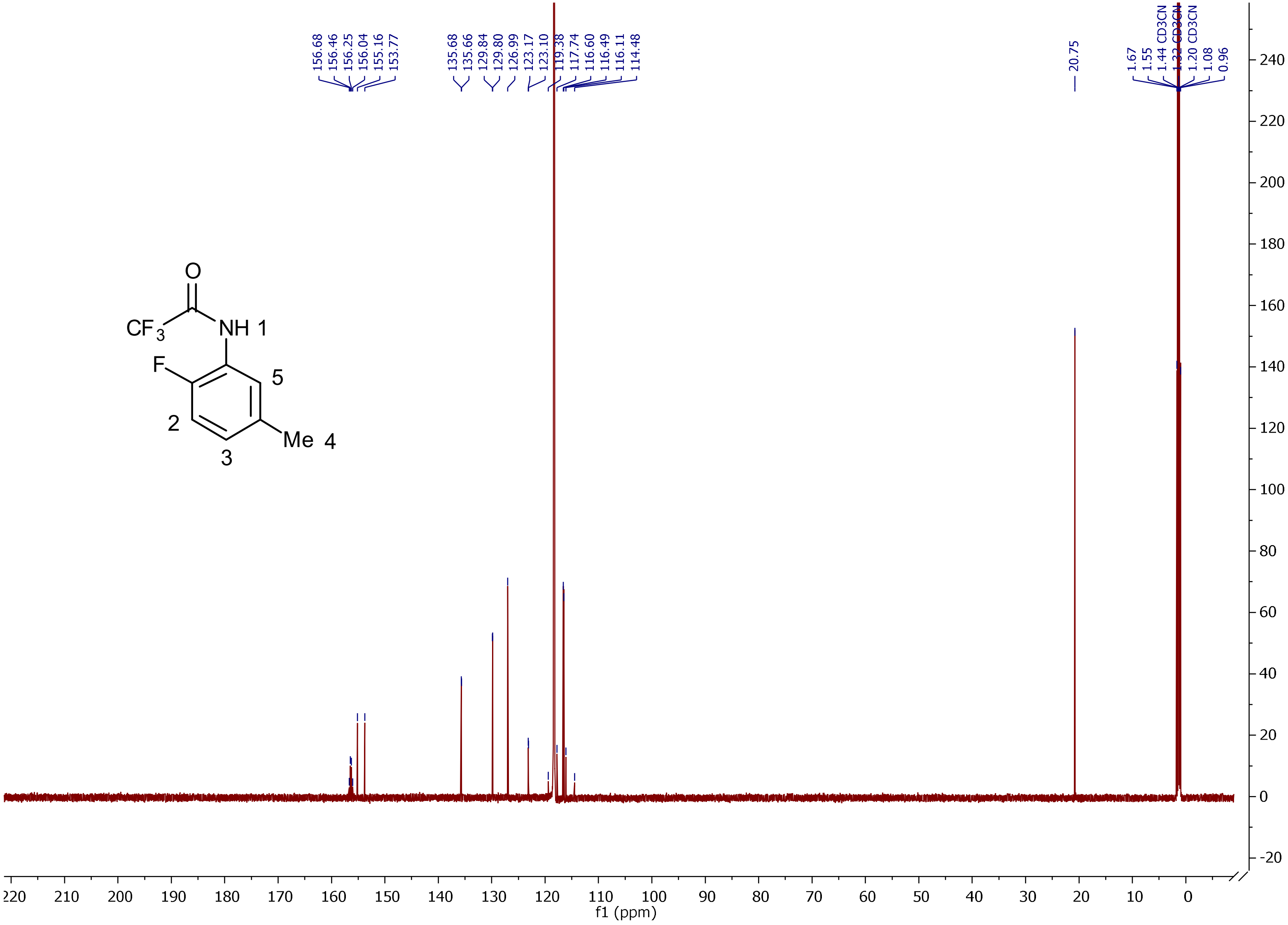

^{19}F{^{1}H} NMR (376 MHz, CD_3CN) for 2,2,2-trifluoro-*N*-(2-fluoro-5-methylphenyl)acetamide **(2j')**

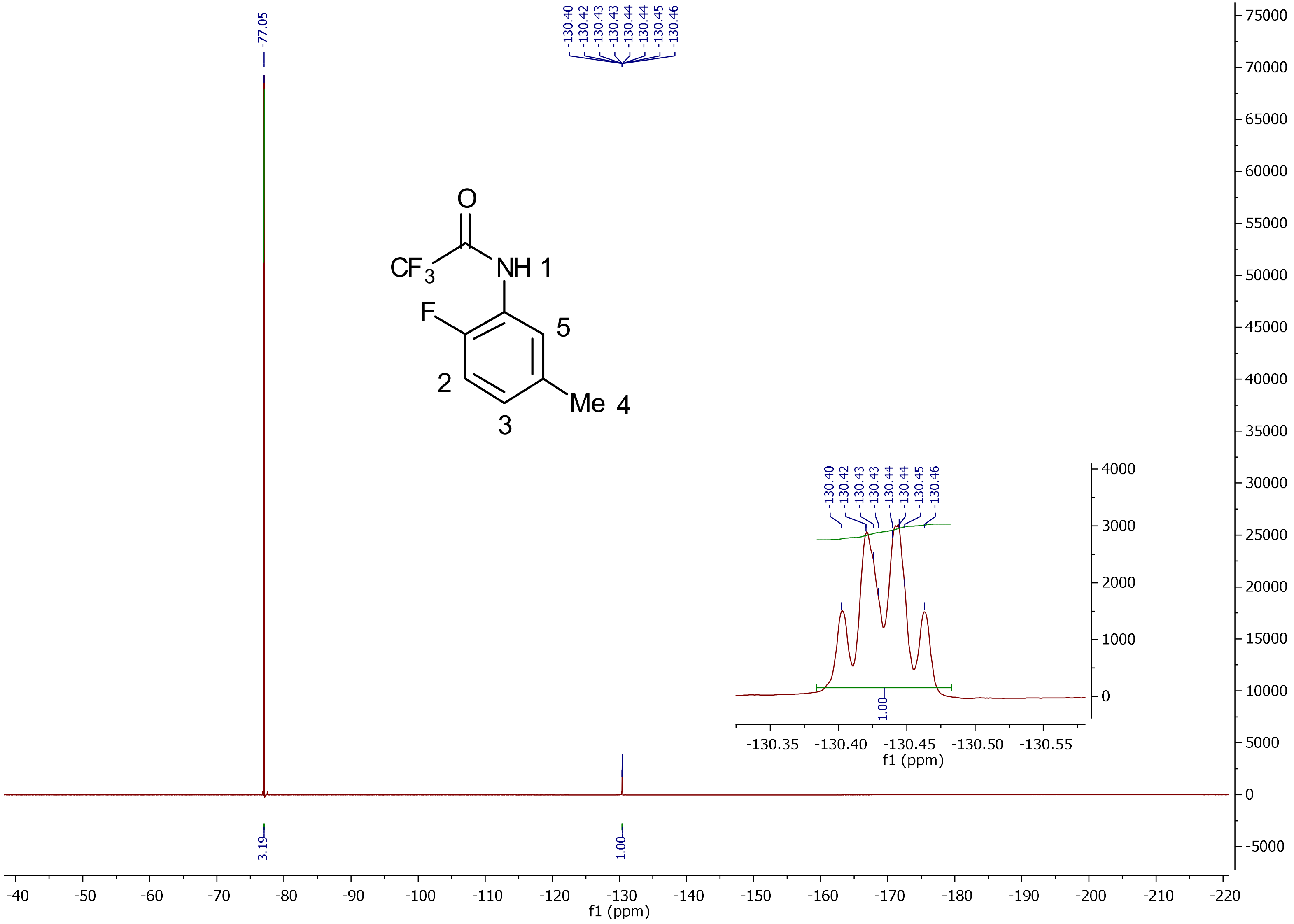

^{1}H NMR (700 MHz, CD_3CN) for fluorination of *N*-(2,5-dimethylphenyl)-2,2,2-trifluoroacetamide **(3k)**

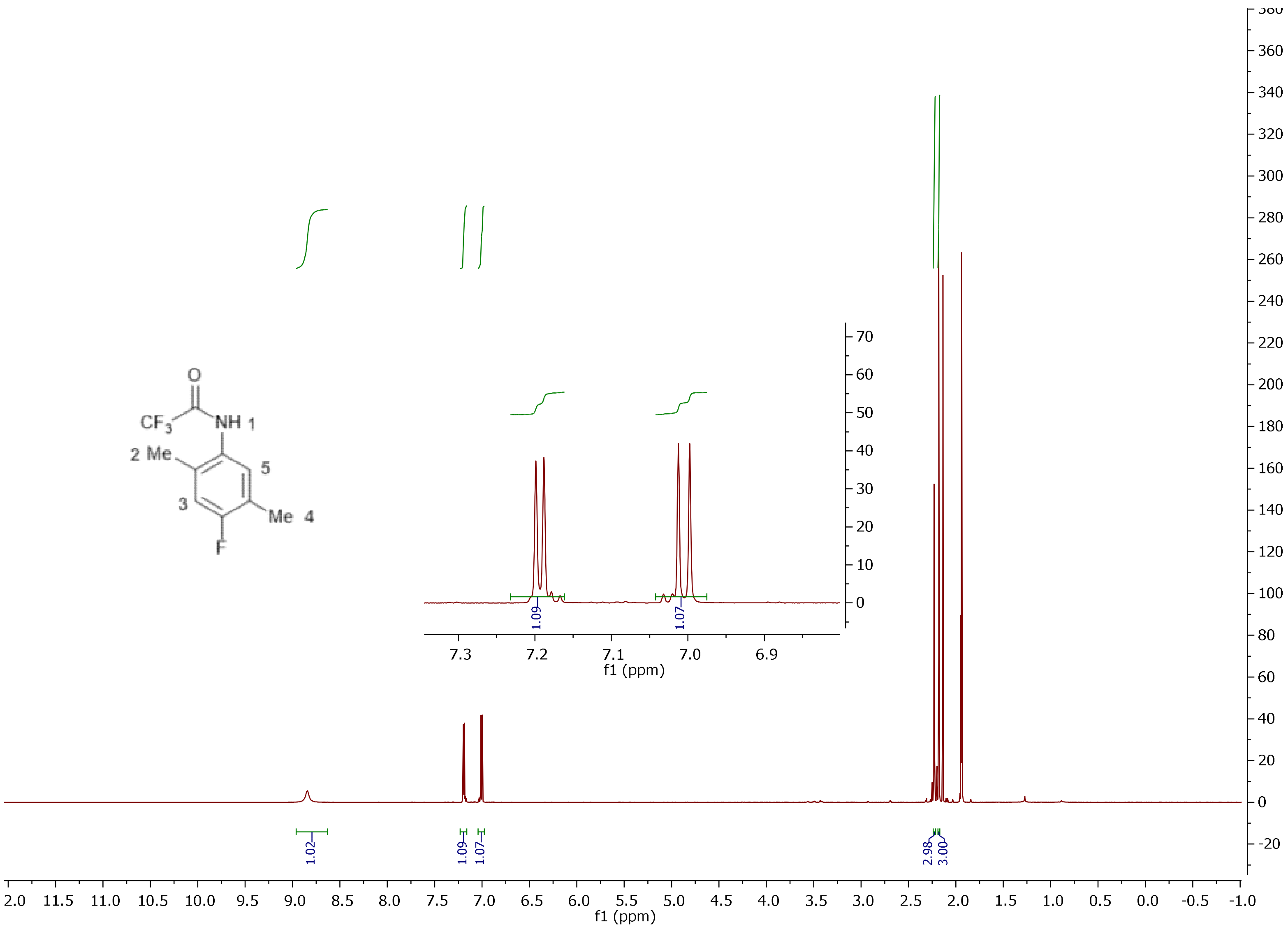

^{13}**C NMR** (176 MHz, CD_3CN) for fluorination of *N*-(2,5-dimethylphenyl)-2,2,2-trifluoroacetamide **(3k)**

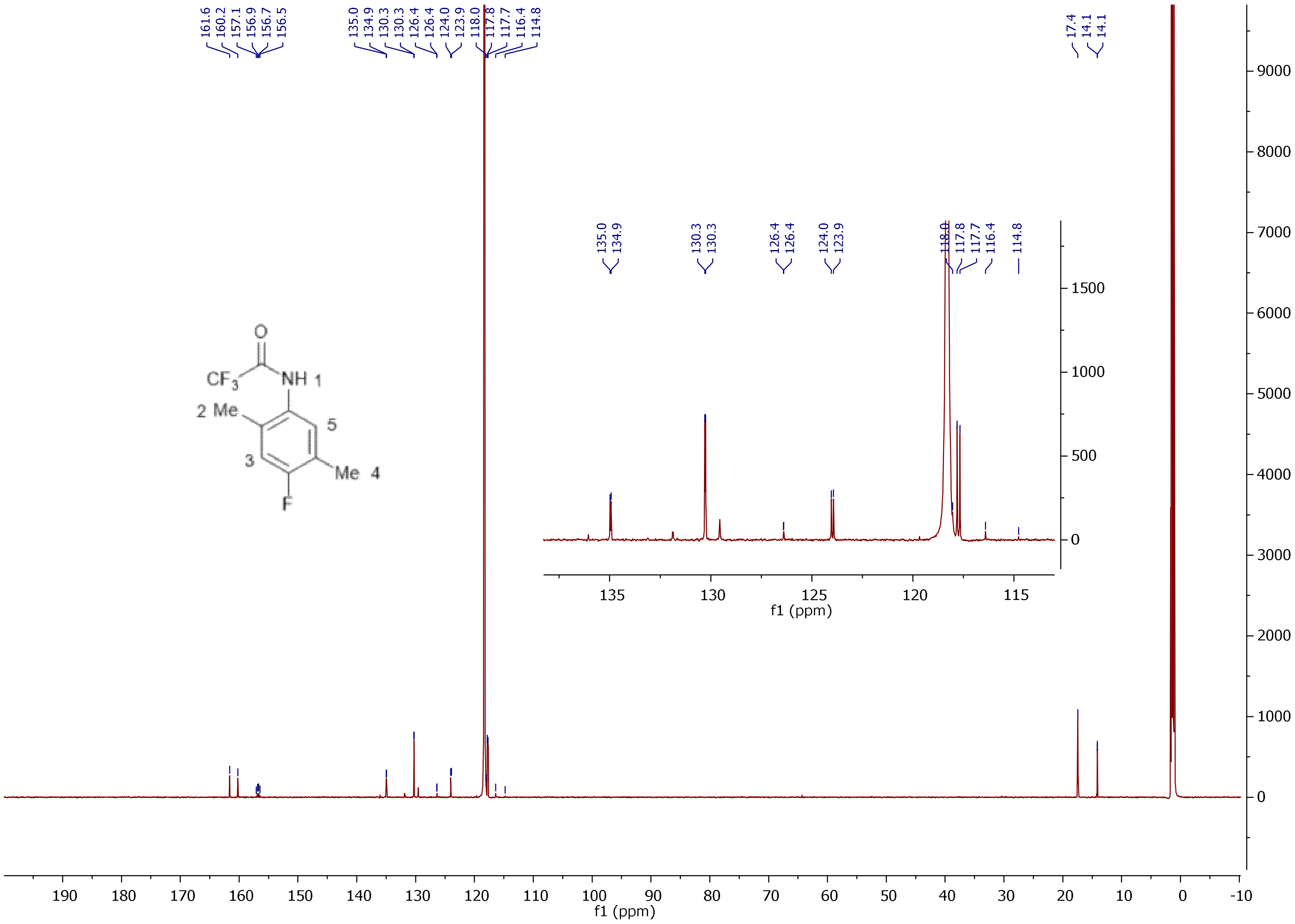

$^{19}F\{^{1}H\}$ NMR (376 MHz, CD_3CN) for fluorination of *N*-(2,5-dimethylphenyl)-2,2,2-trifluoroacetamide **(3k)**

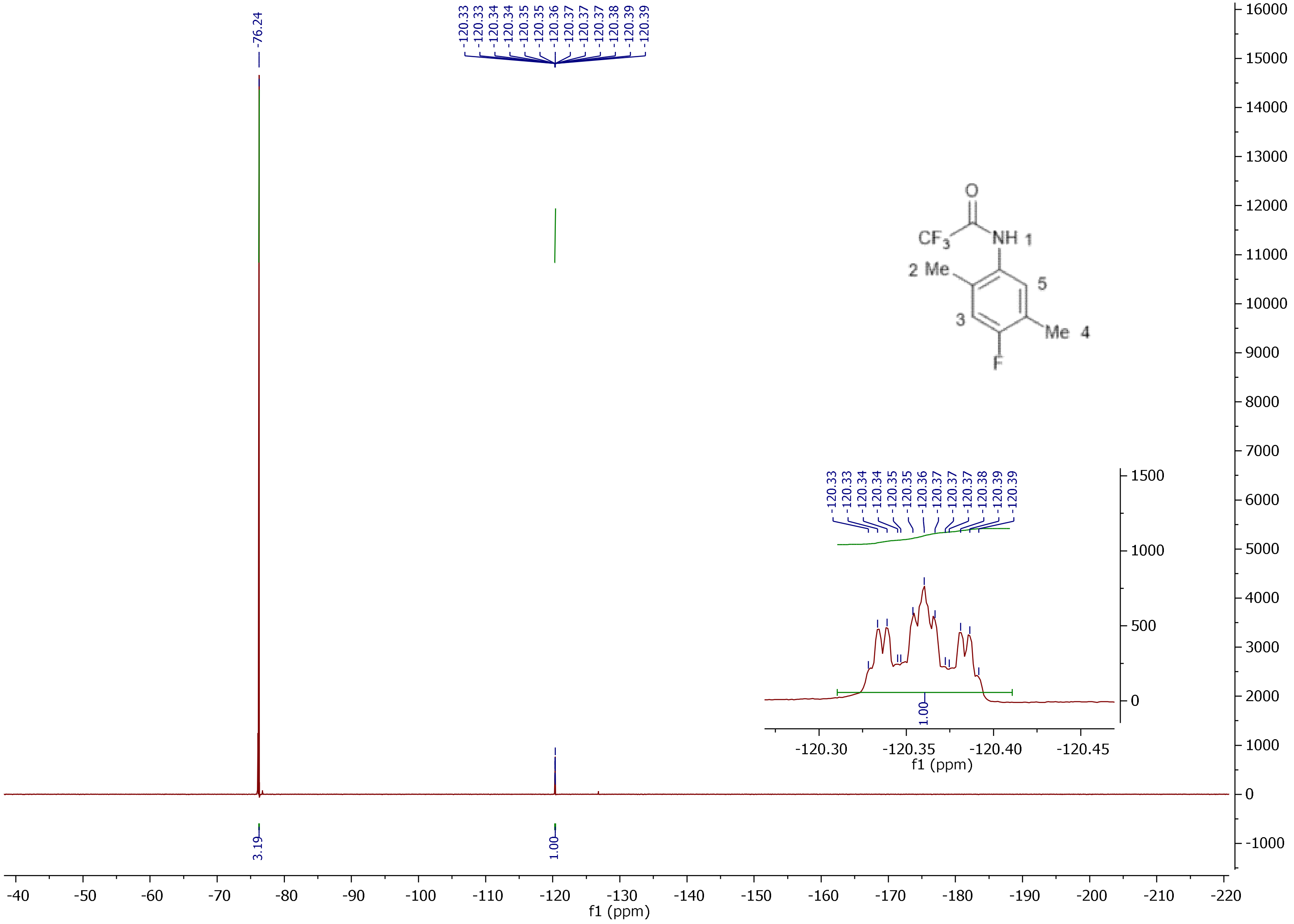

HMBC NMR (CD_3CN) for fluorination of *N*-(2,5-dimethylphenyl)-2,2,2-trifluoroacetamide **(3k)**

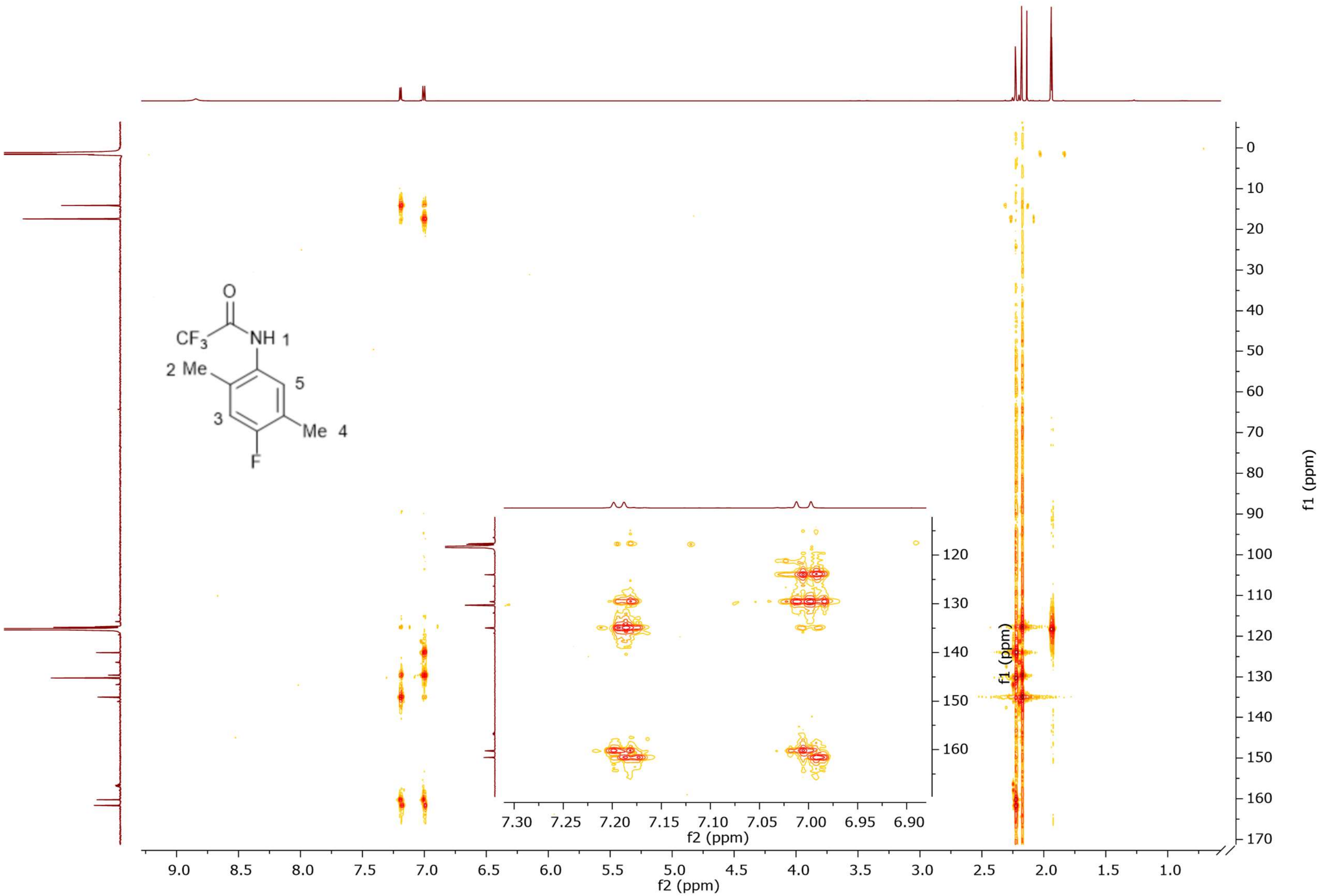

^{1}H NMR (700 MHz, CD_3CN) for fluorination of 2,2,2-trifluoro-*N*-(5,6,7,8-tetrahydronaphthalen-1-yl)acetamide **(2l and 3l)**

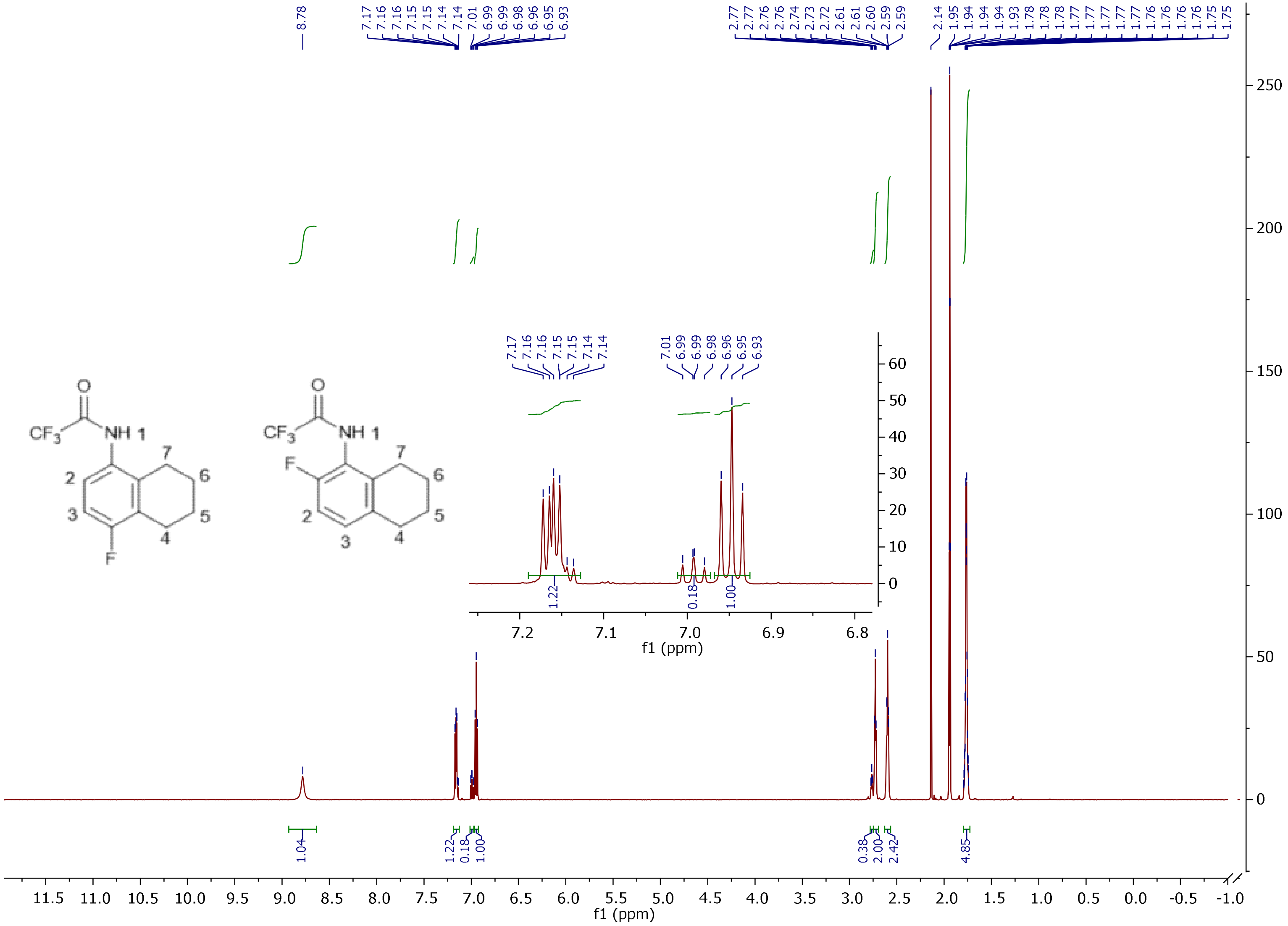

^{13}**C NMR** (176 MHz, CD_3CN) for fluorination of 2,2,2-trifluoro-*N*-(5,6,7,8-tetrahydronaphthalen-1-yl)acetamide **(2l and 3l)**

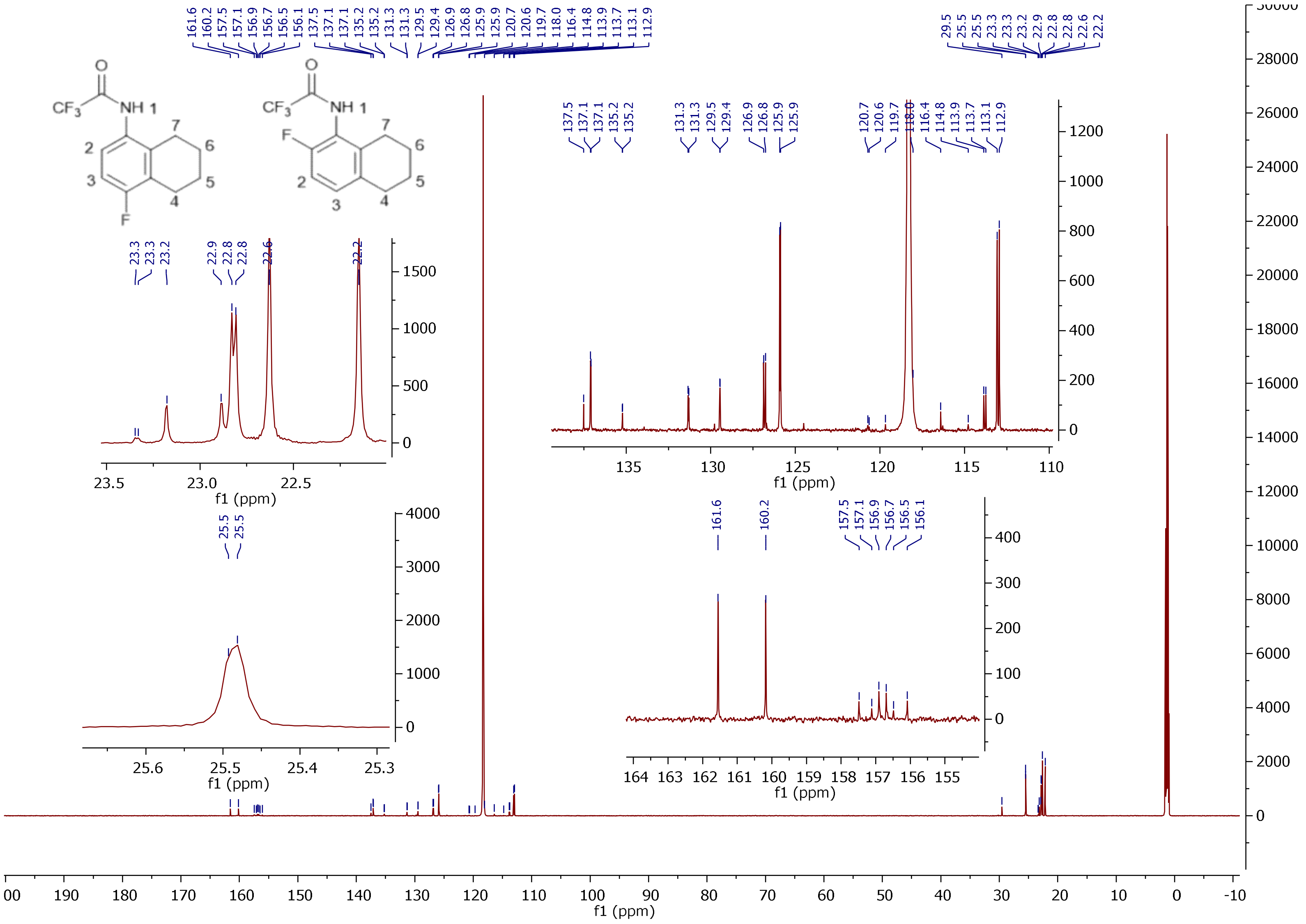

$^{19}F\{^1H\}$ NMR (376 MHz, CD_3CN) for fluorination of 2,2,2-trifluoro-*N*-(5,6,7,8-tetrahydronaphthalen-1-yl)acetamide **(2l and 3l)**

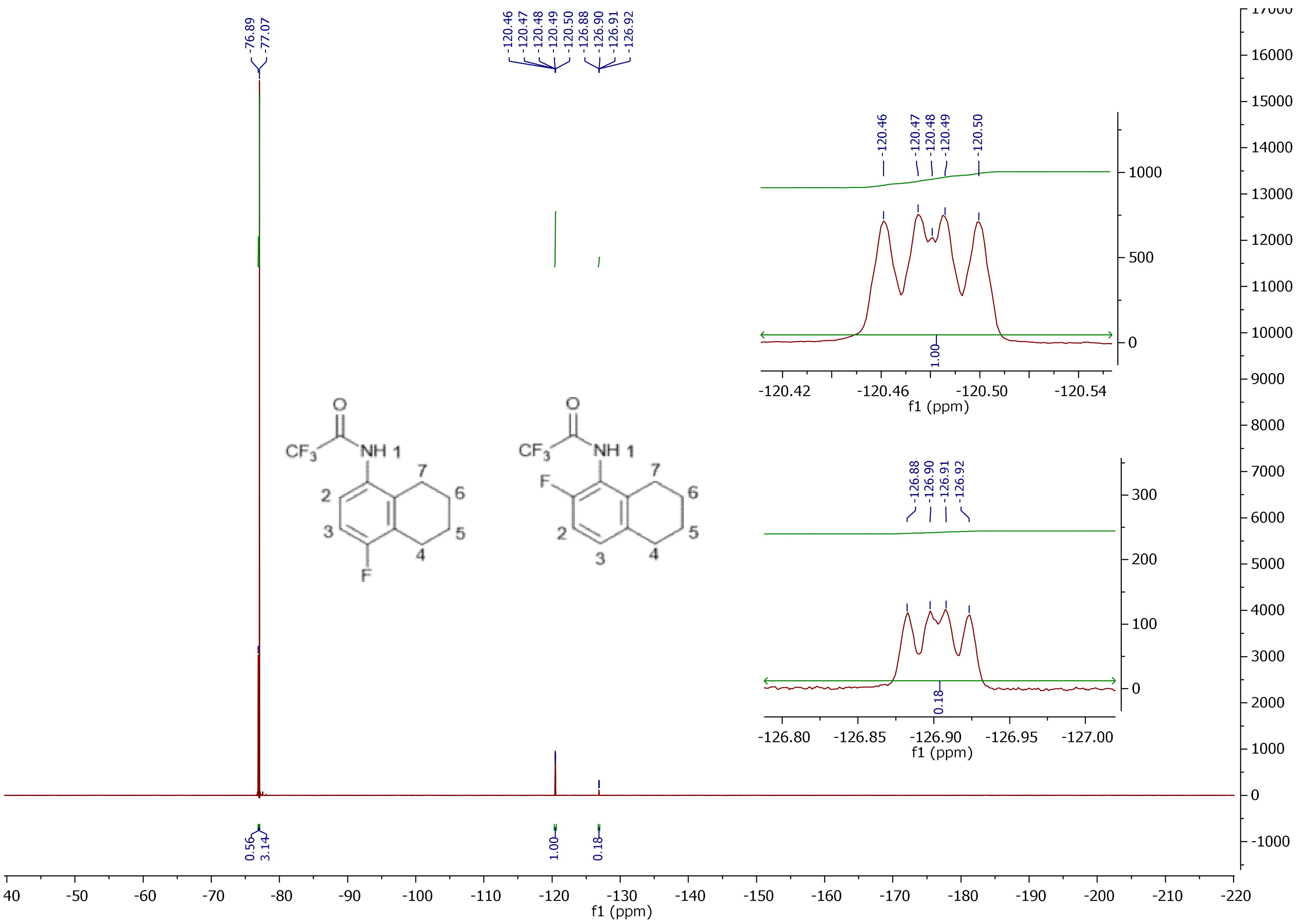

HMBC NMR (CD_3CN) for fluorination of 2,2,2-trifluoro-*N*-(5,6,7,8-tetrahydronaphthalen-1-yl)acetamide **(2l and 3l)**

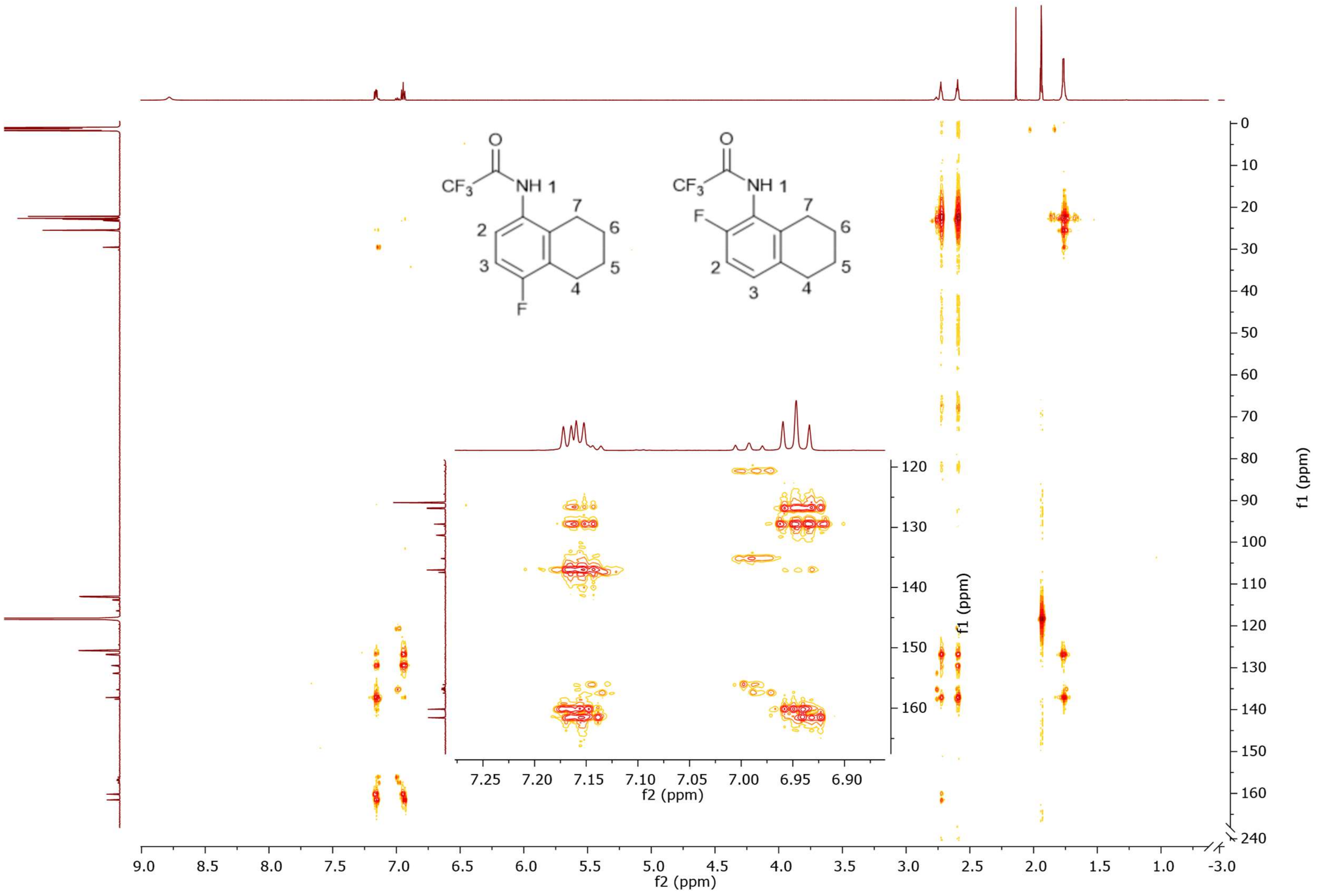

¹H NMR (700 MHz, CD_3CN) for fluorination of 2,2,2-trifluoro-*N*-(2-isopropylphenyl)acetamide **(2m and 3m)**

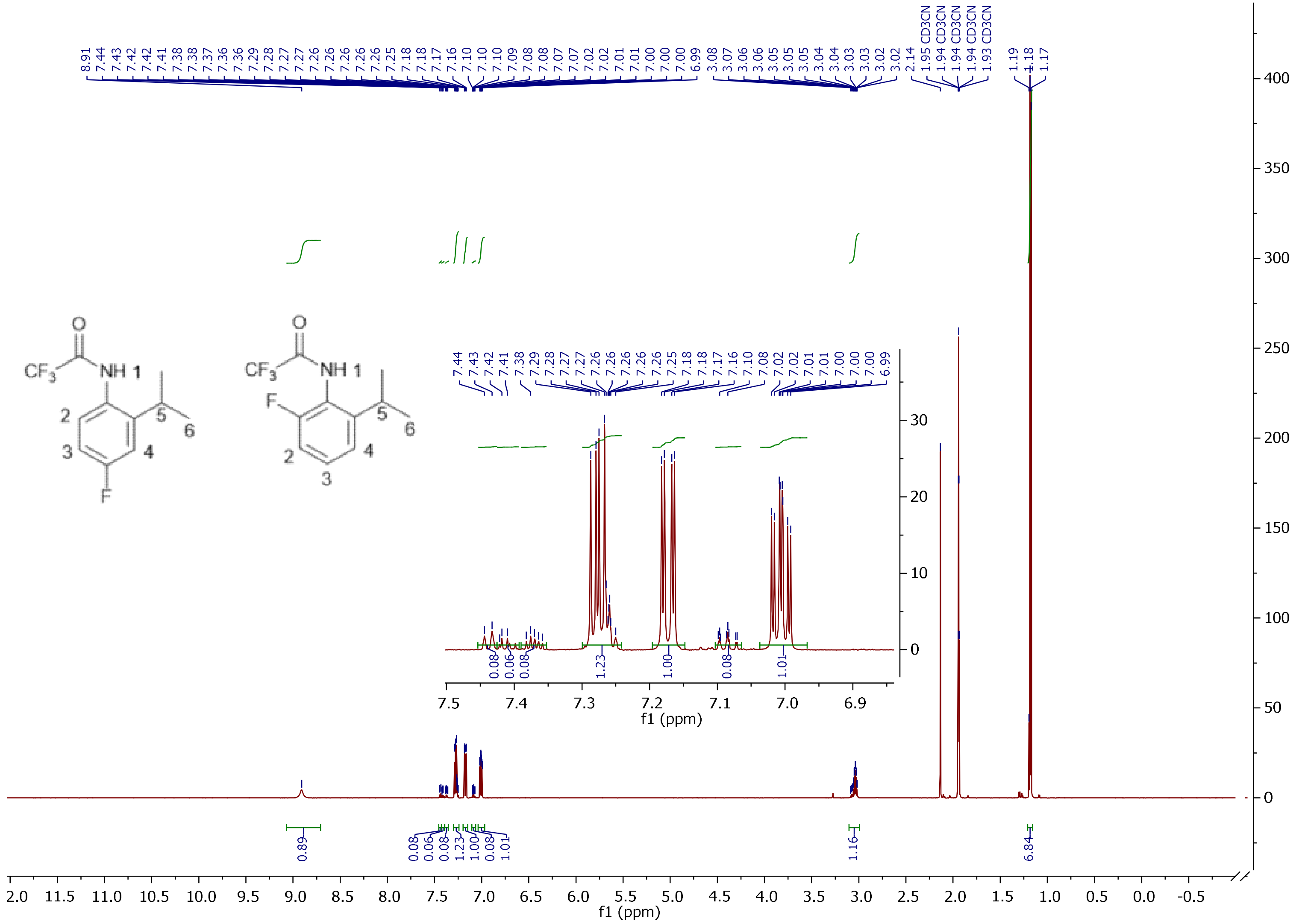

^{13}C NMR (176 MHz, CD_3CN) for fluorination of 2,2,2-trifluoro-*N*-(2-isopropylphenyl)acetamide **(2m and 3m)**

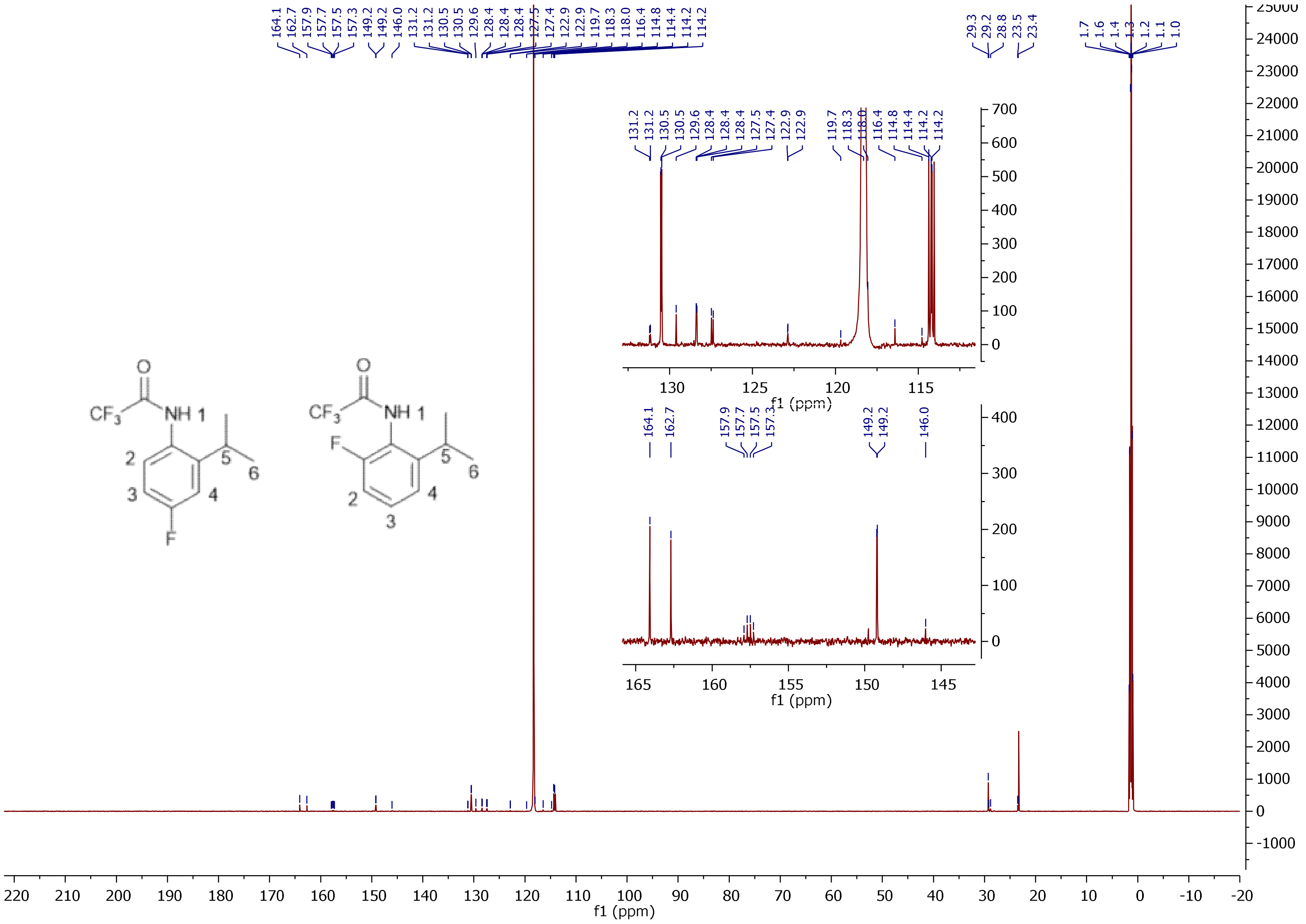

$^{19}F\{^1H\}$ NMR (376 MHz, CD_3CN) for fluorination of 2,2,2-trifluoro-*N*-(2-isopropylphenyl)acetamide **(2m and 3m)**

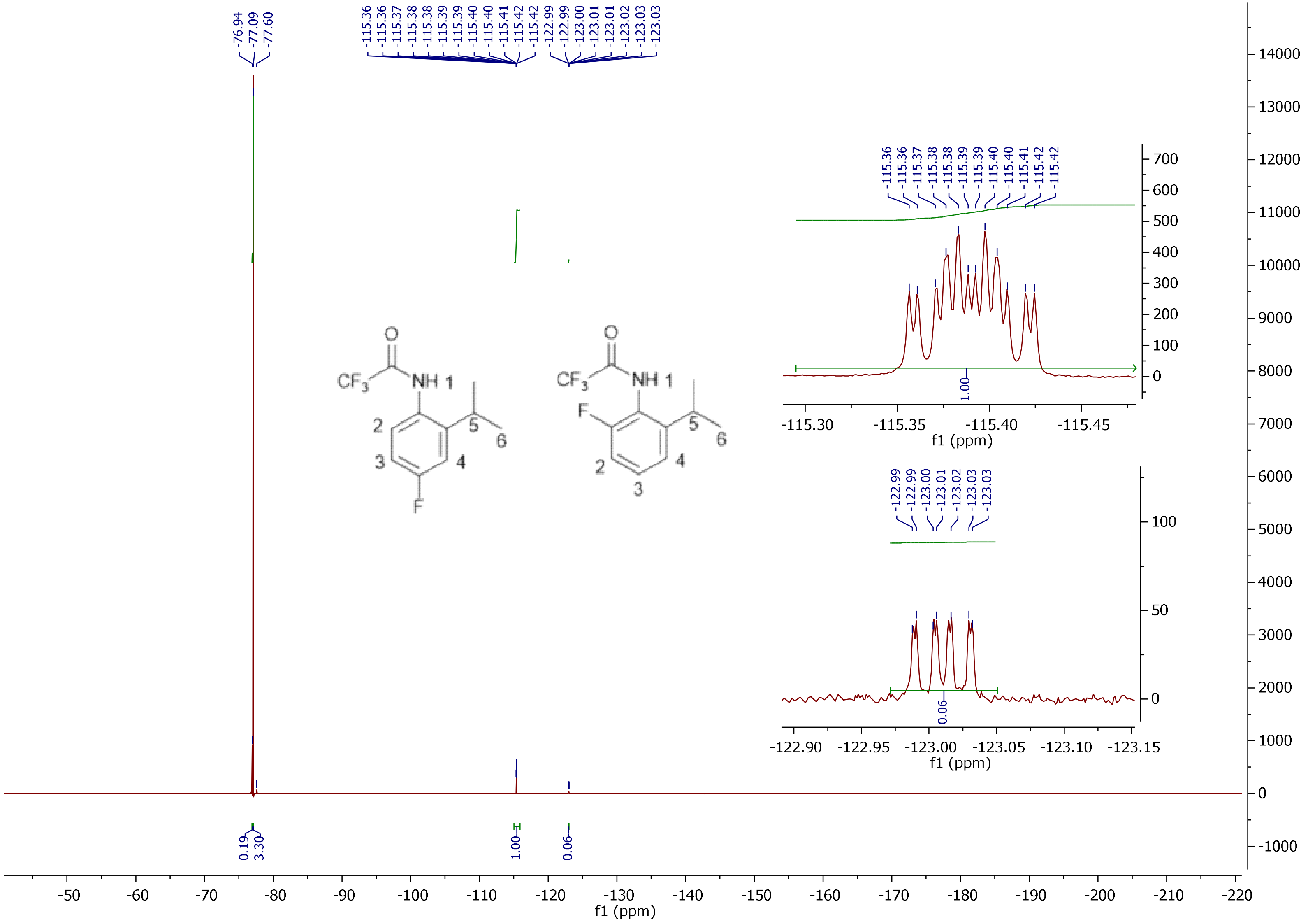

COSY NMR (CD_3CN) for fluorination of 2,2,2-trifluoro-*N*-(2-isopropylphenyl)acetamide **(2m and 3m)**

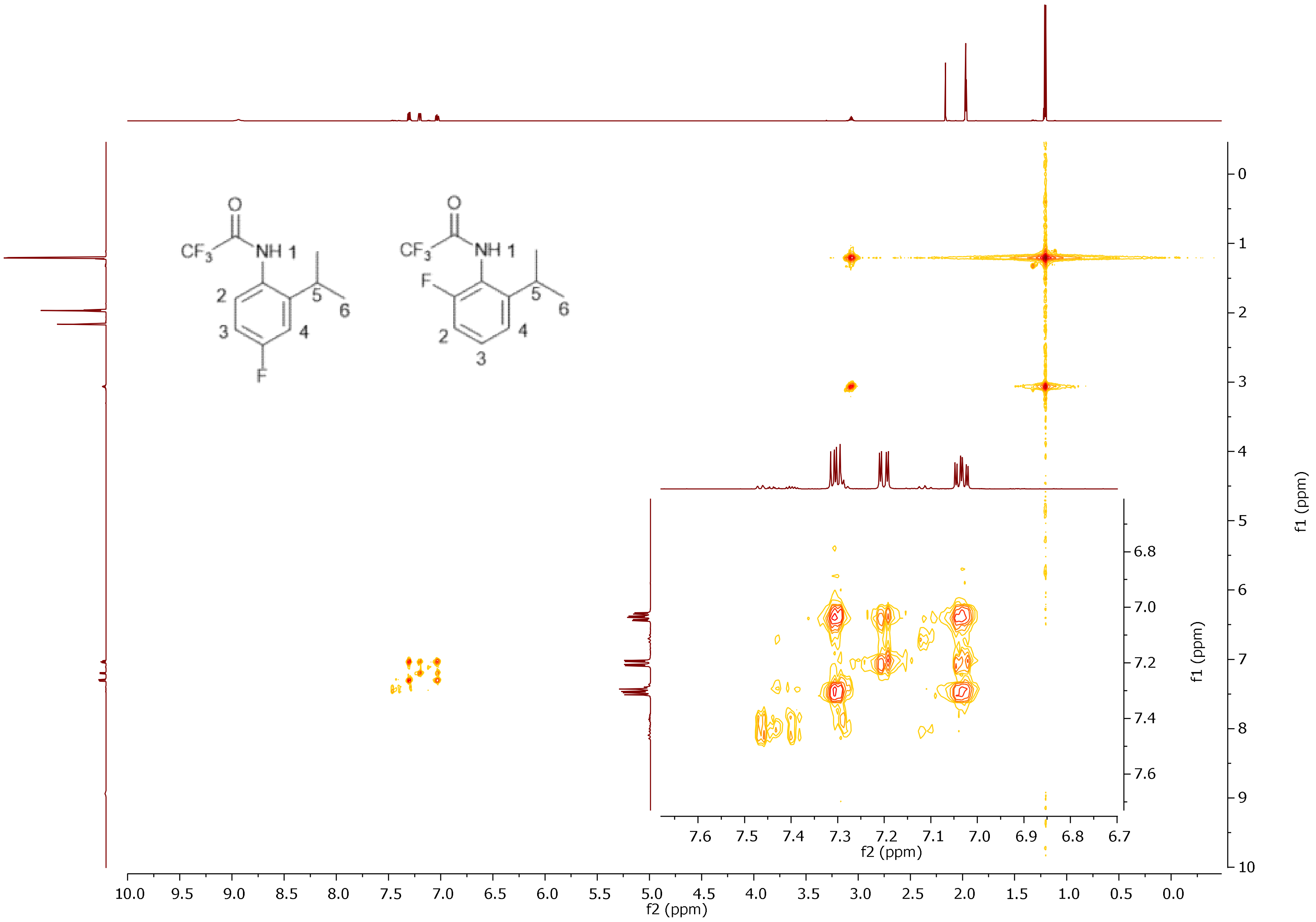

^{1}H NMR (700 MHz, CD_3CN) for fluorination of 2,2,2-trifluoro-*N*-(3-fluorophenyl)acetamide **(3n)**

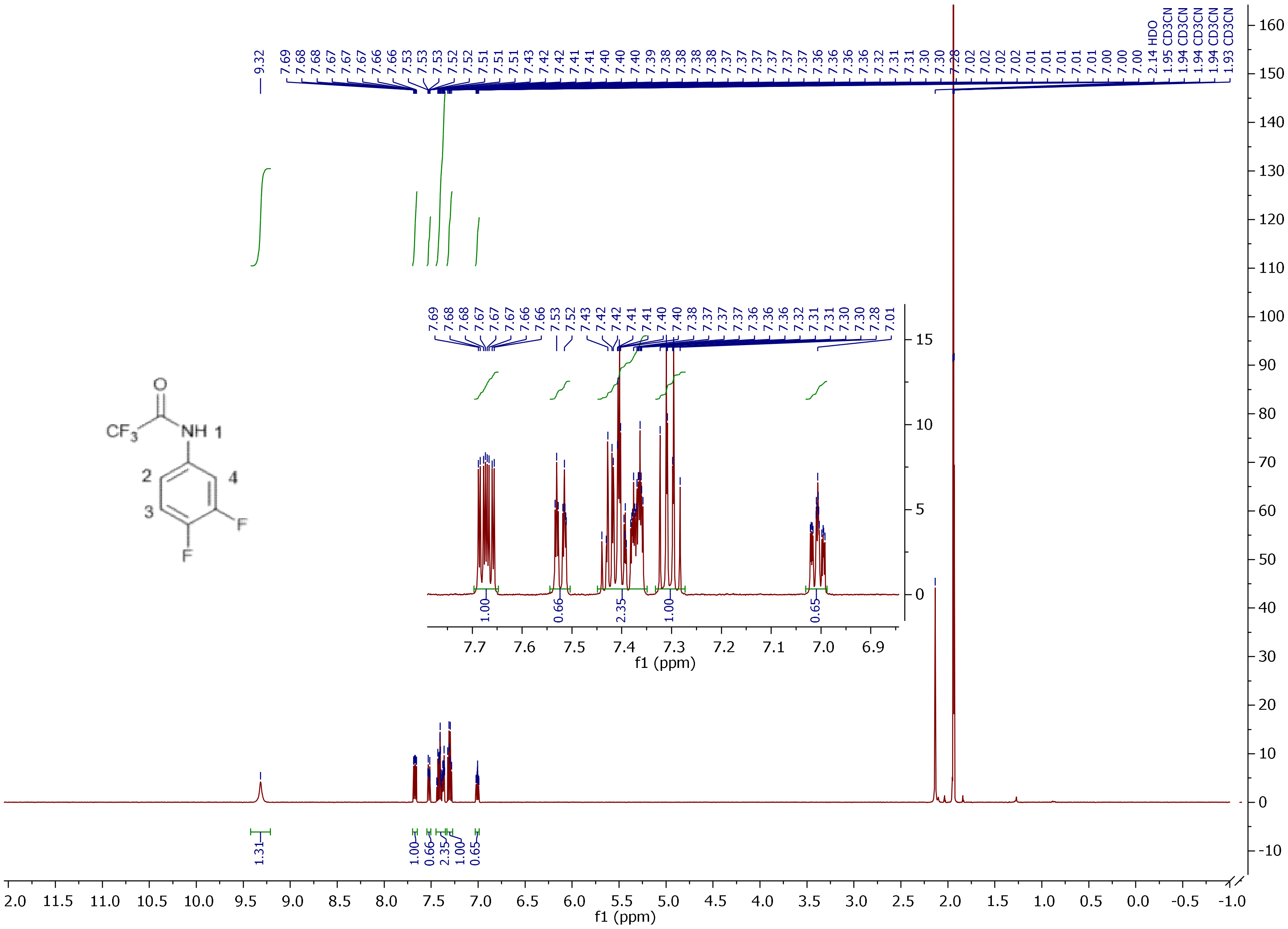

^{13}C NMR (176 MHz, CD_3CN) for fluorination of 2,2,2-trifluoro-*N*-(3-fluorophenyl)acetamide **(3n)**

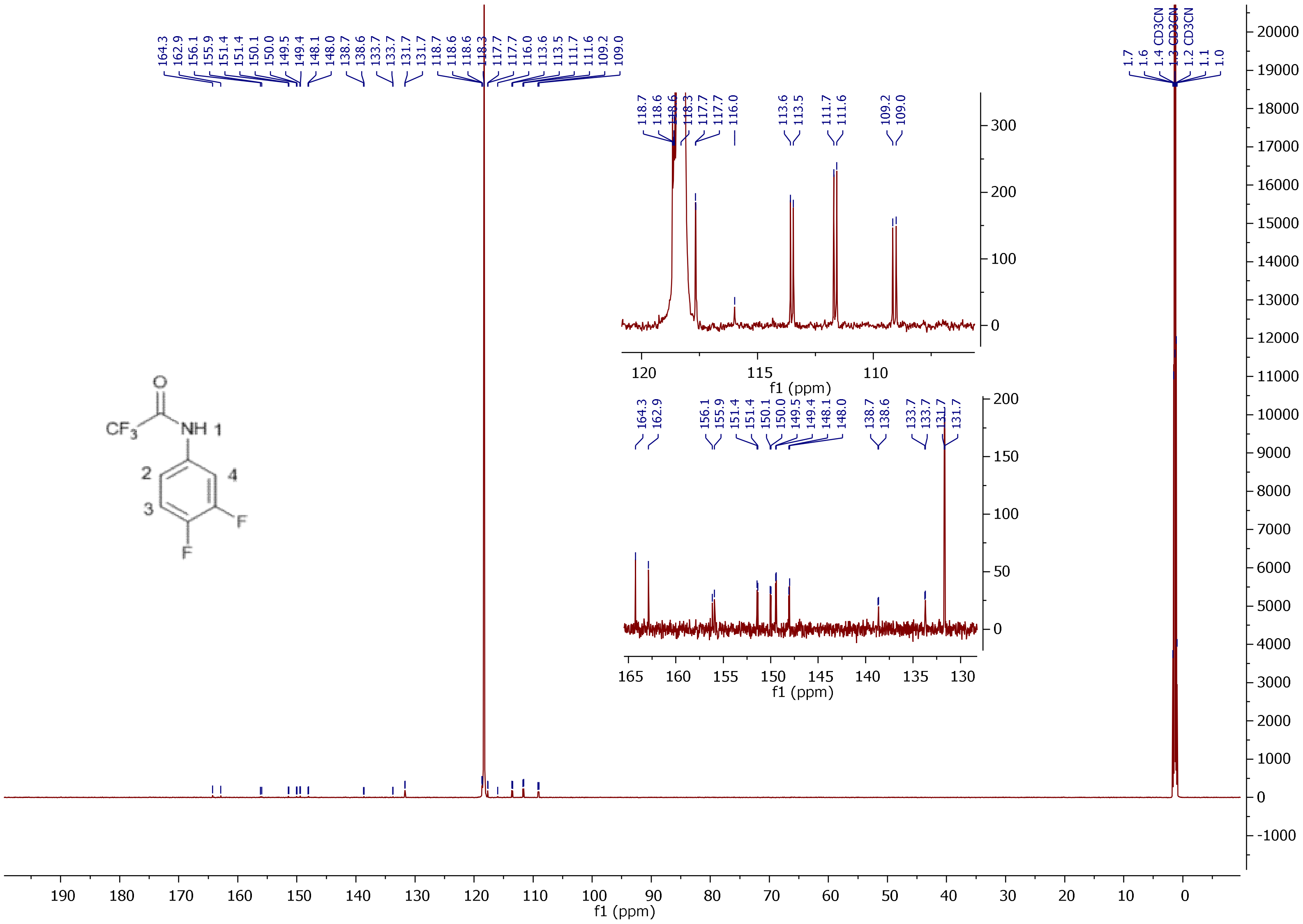

$^{19}F\{^{1}H\}$ NMR (376 MHz, CD_3CN) for fluorination of 2,2,2-trifluoro-*N*-(3-fluorophenyl)acetamide **(3n)**

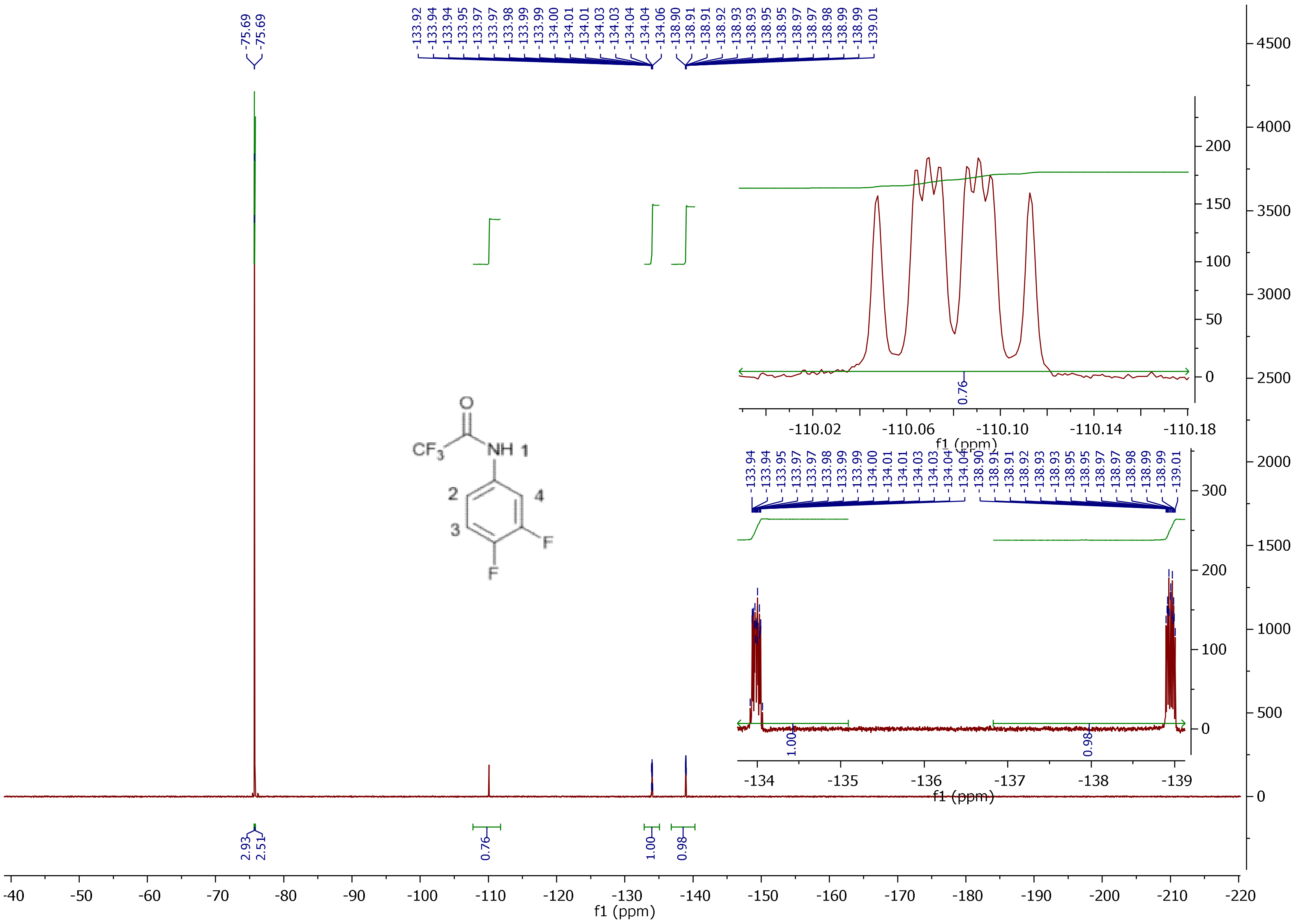

^{1}H NMR (700 MHz, CD_3CN) for *N*-(2,3-difluorophenyl)-2,2,2-trifluoroacetamide **(2n)**

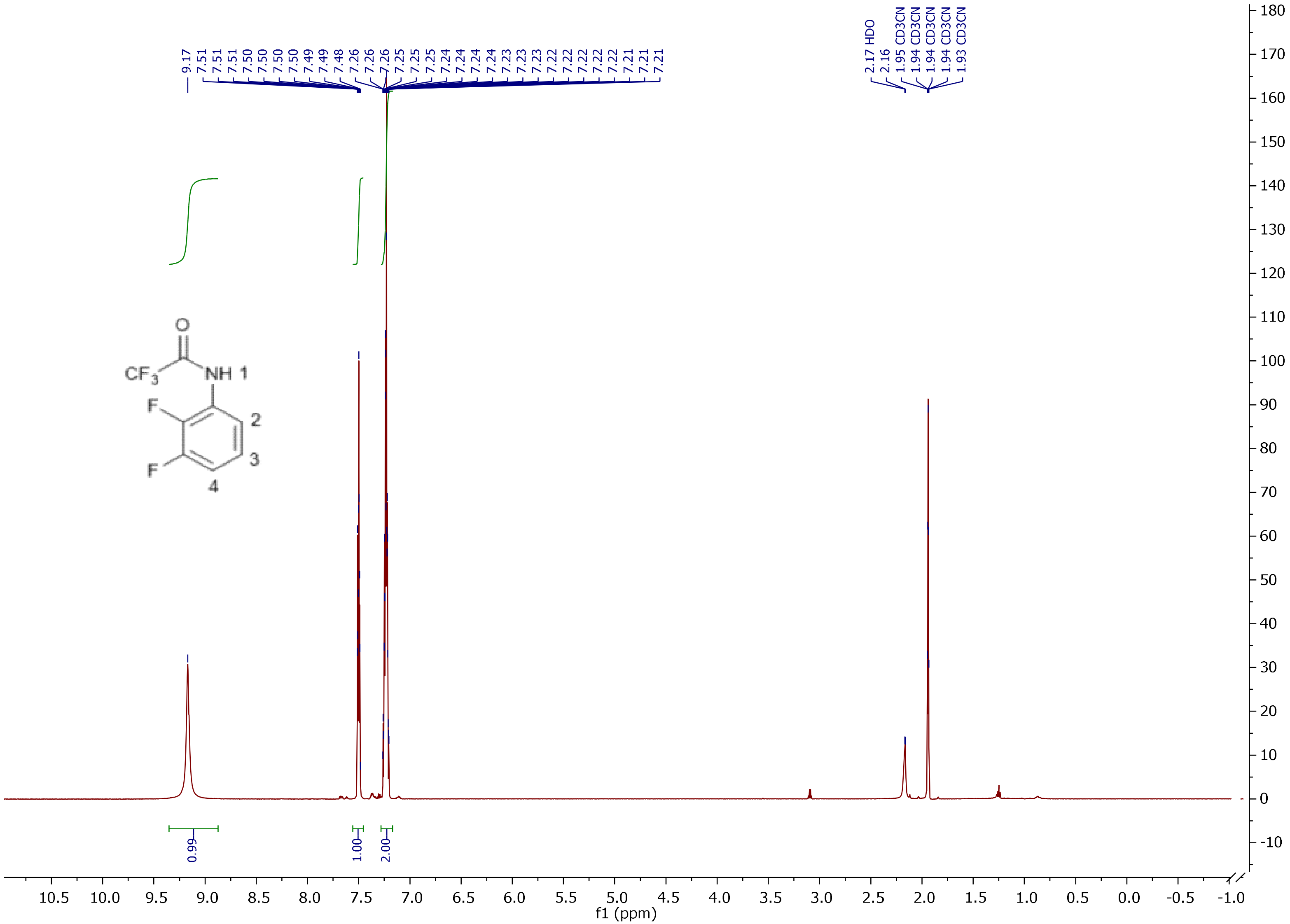

^{13}C NMR (176 MHz, CD_3CN) for *N*-(2,3-difluorophenyl)-2,2,2-trifluoroacetamide **(2n)**

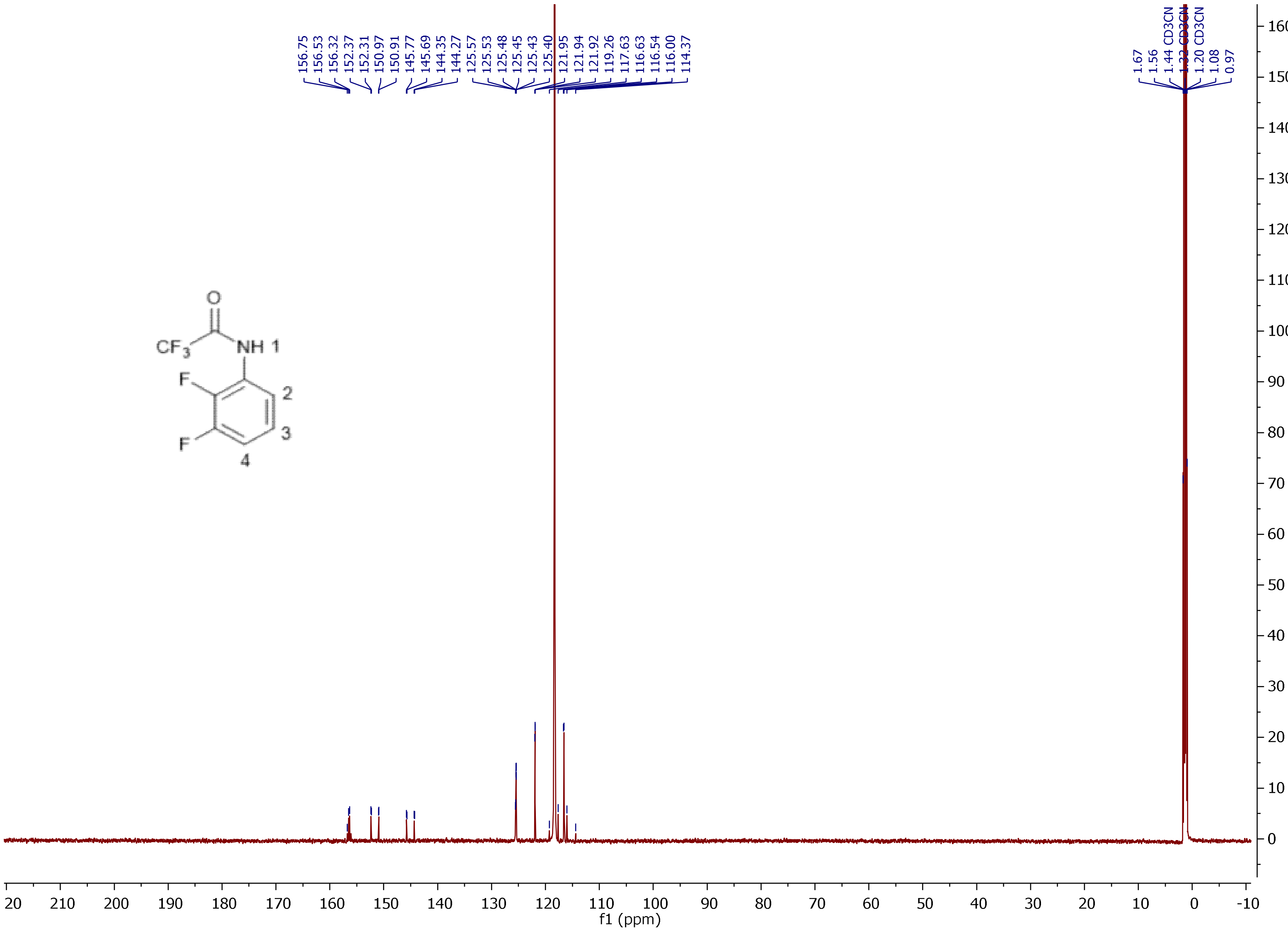

$^{19}F\{^{1}H\}$ **NMR** (376 MHz, CD_3CN) for *N*-(2,3-difluorophenyl)-2,2,2-trifluoroacetamide **(2n)**

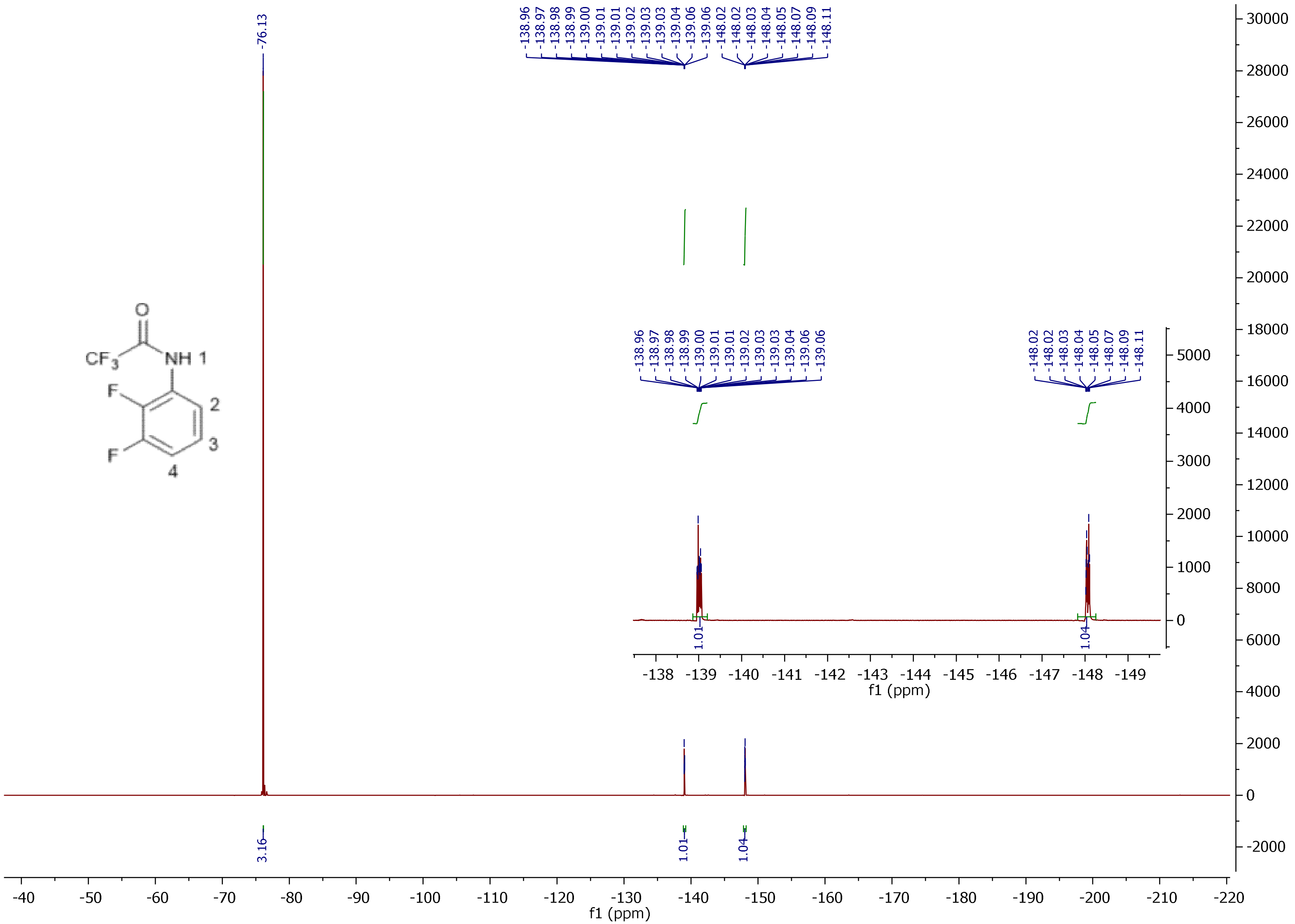

^{1}H NMR (700 MHz, CD_3CN) for *N*-(2,5-difluorophenyl)-2,2,2-trifluoroacetamide **(2n')**

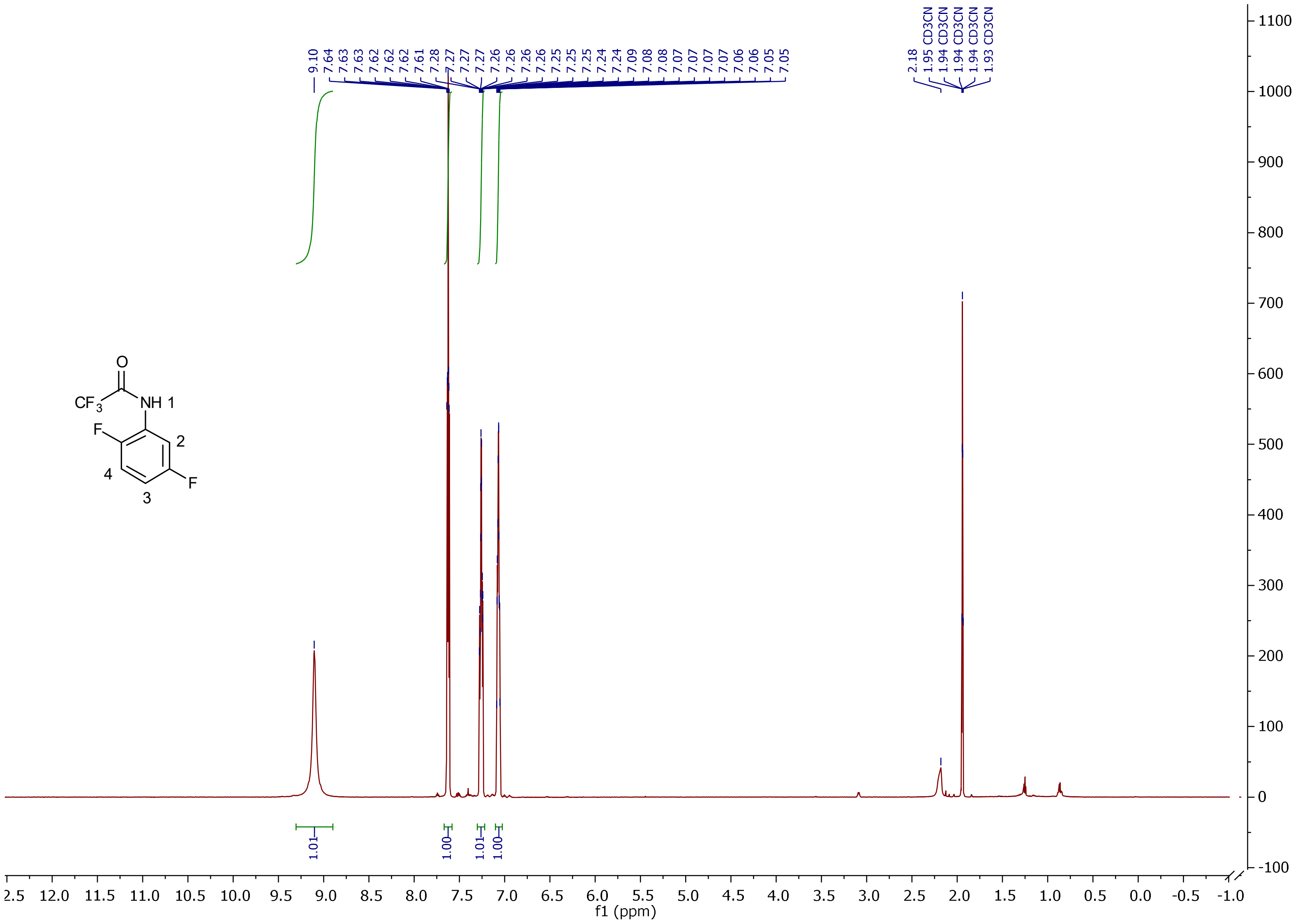

^{13}**C NMR** (176 MHz, CD_3CN) for *N*-(2,5-difluorophenyl)-2,2,2-trifluoroacetamide **(2n')**

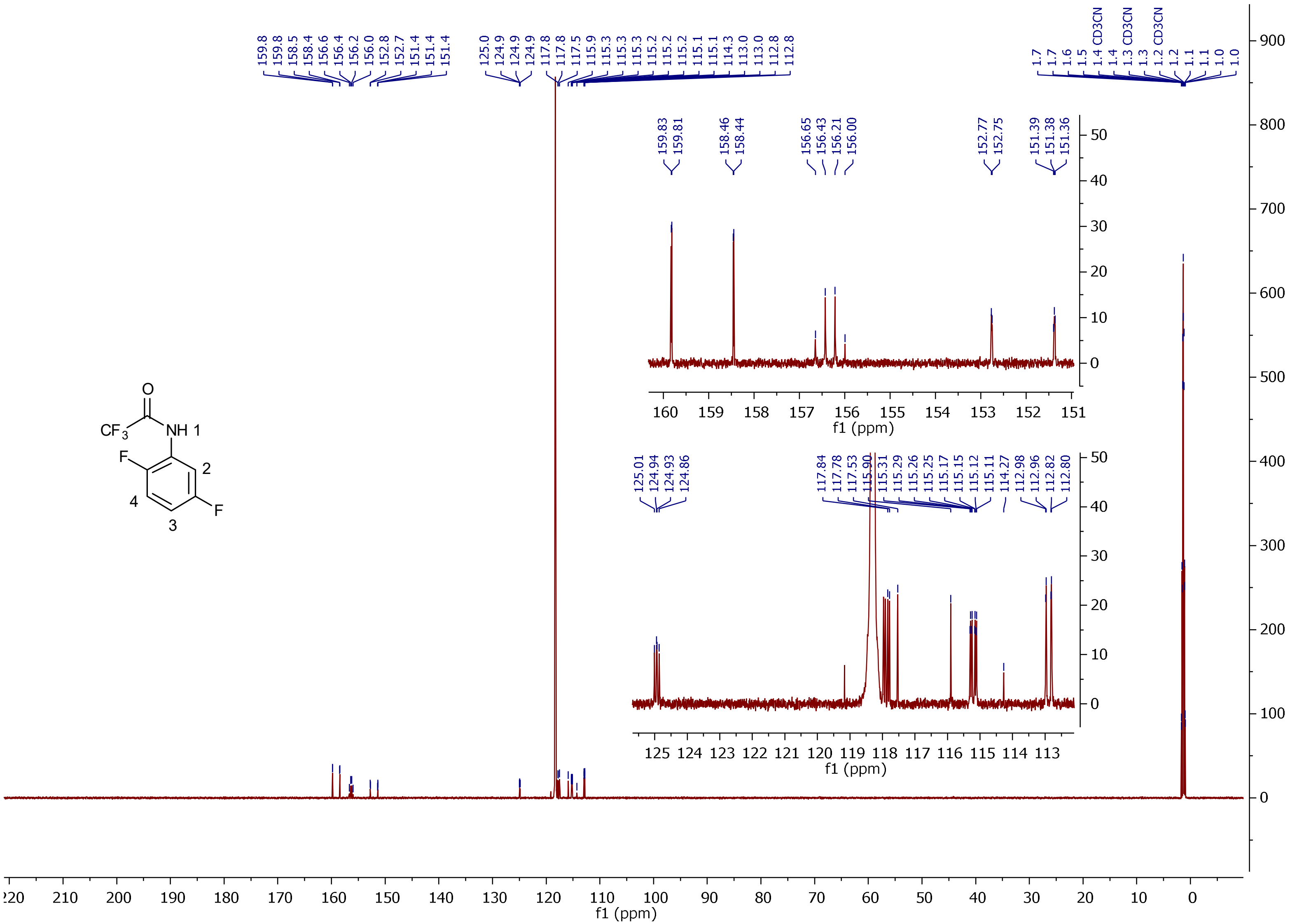

$^{19}F\{^{1}H\}$ NMR (376 MHz, CD_3CN) for *N*-(2,5-difluorophenyl)-2,2,2-trifluoroacetamide **(2n')**

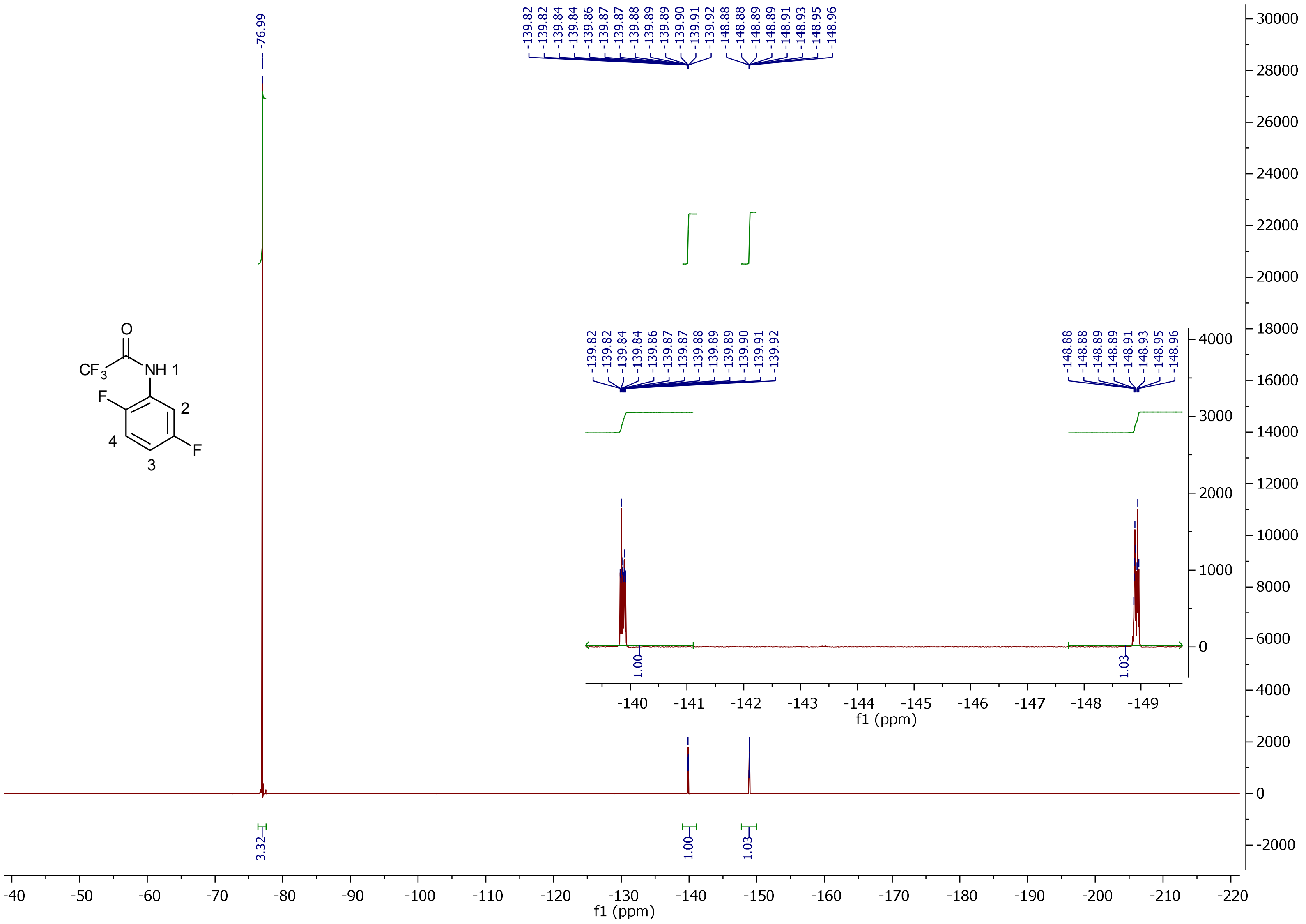

[1]**H NMR** (700 MHz, CD_3CN) for fluorination of *N*-(3-chlorophenyl)-2,2,2-trifluoroacetamide **(3o)**

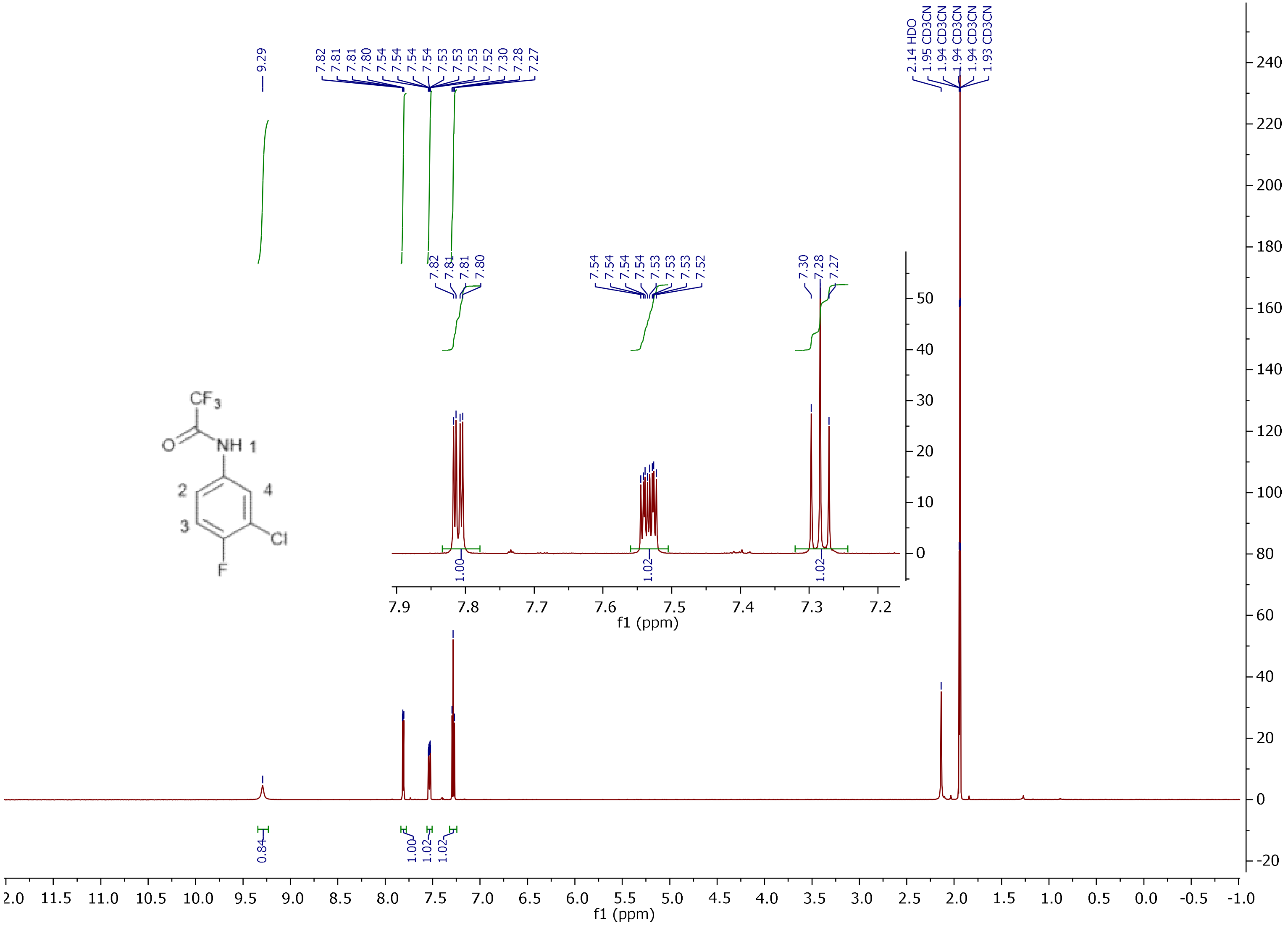

^{13}C NMR (176 MHz, CD_3CN) for fluorination of *N*-(3-chlorophenyl)-2,2,2-trifluoroacetamide **(3o)**

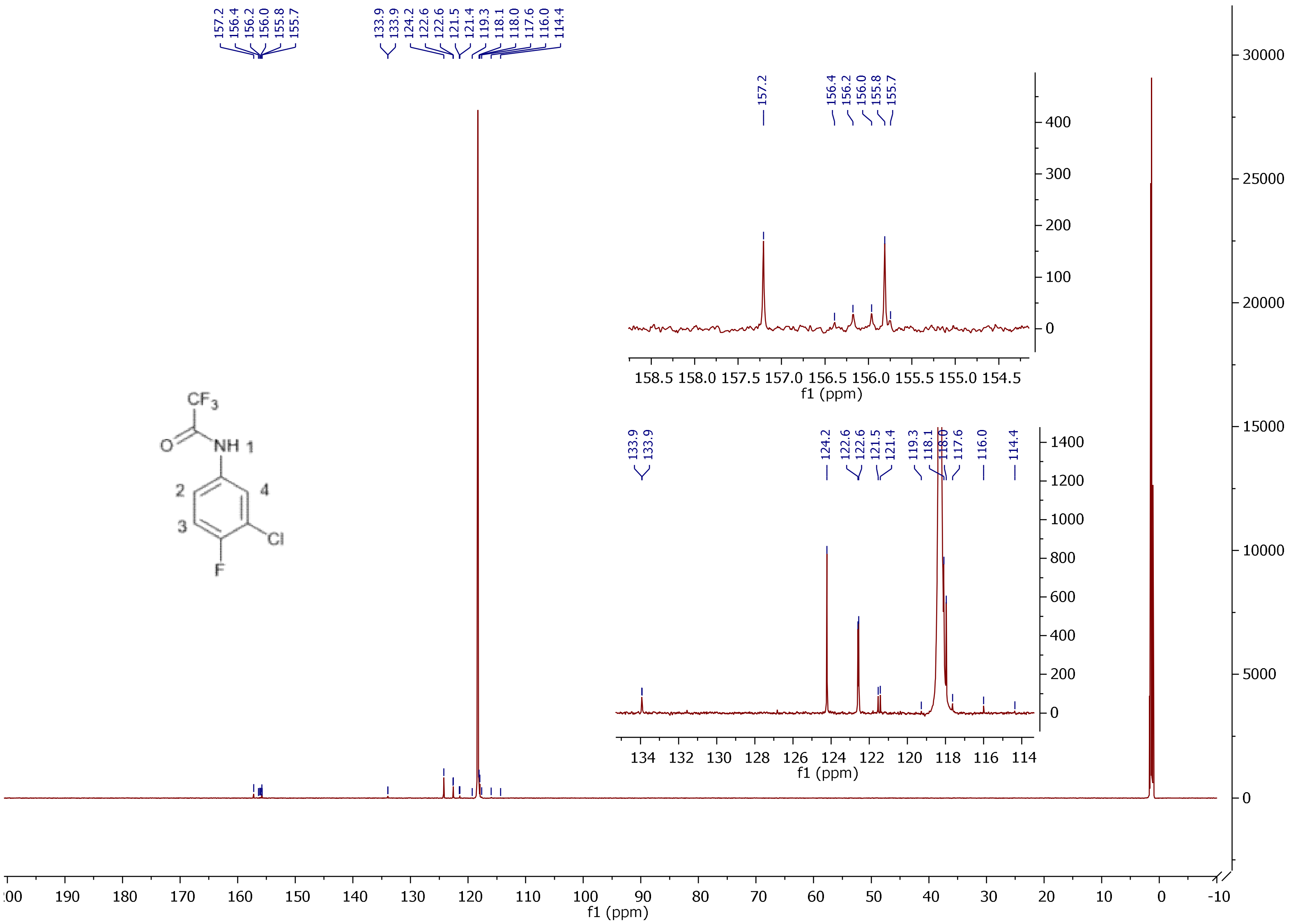

$^{19}F\{^{1}H\}$ NMR (376 MHz, CD_3CN) for fluorination of *N*-(3-chlorophenyl)-2,2,2-trifluoroacetamide **(3o)**

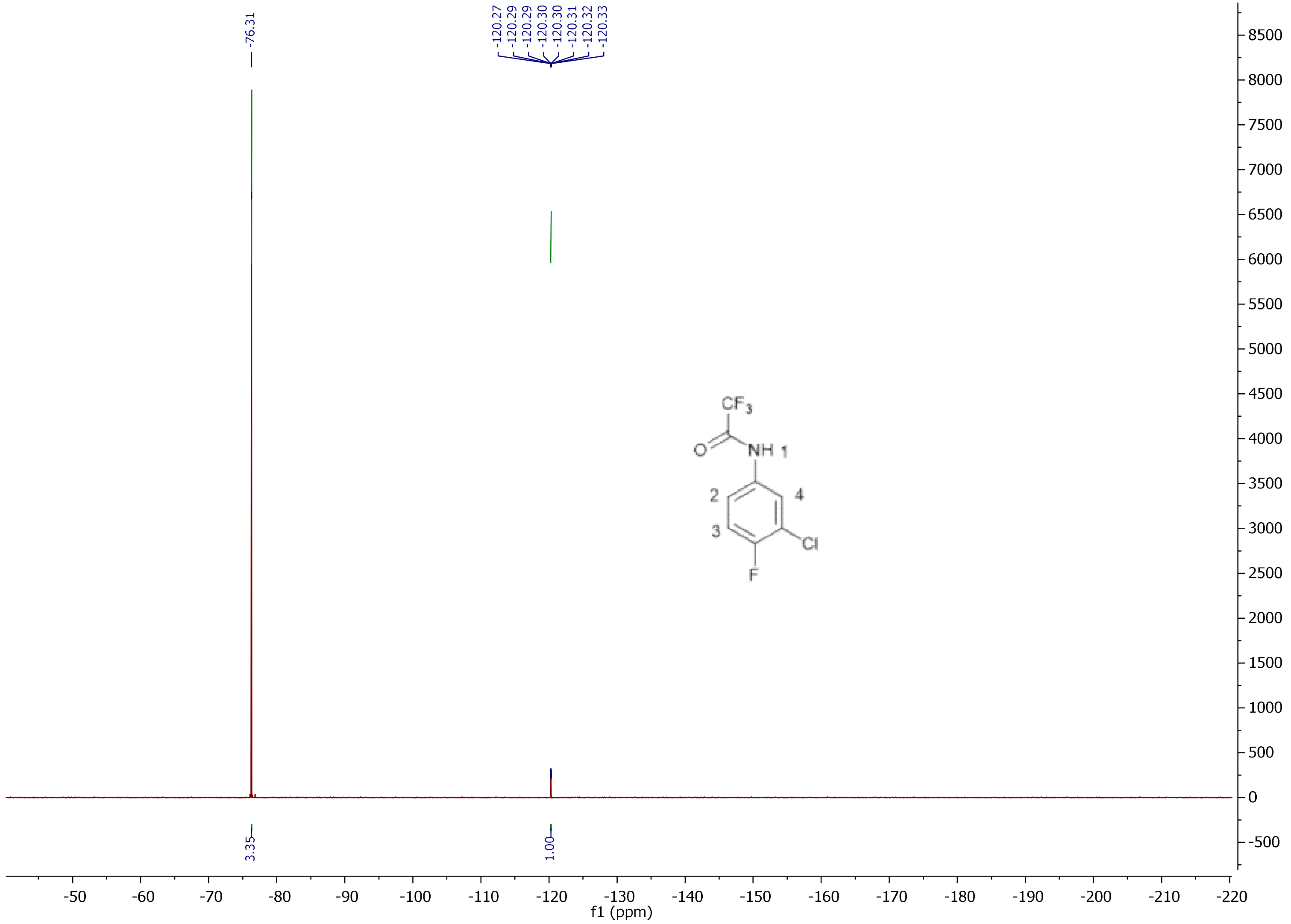

HMBC NMR (CD_3CN) for fluorination of *N*-(3-chlorophenyl)-2,2,2-trifluoroacetamide **(3o)**

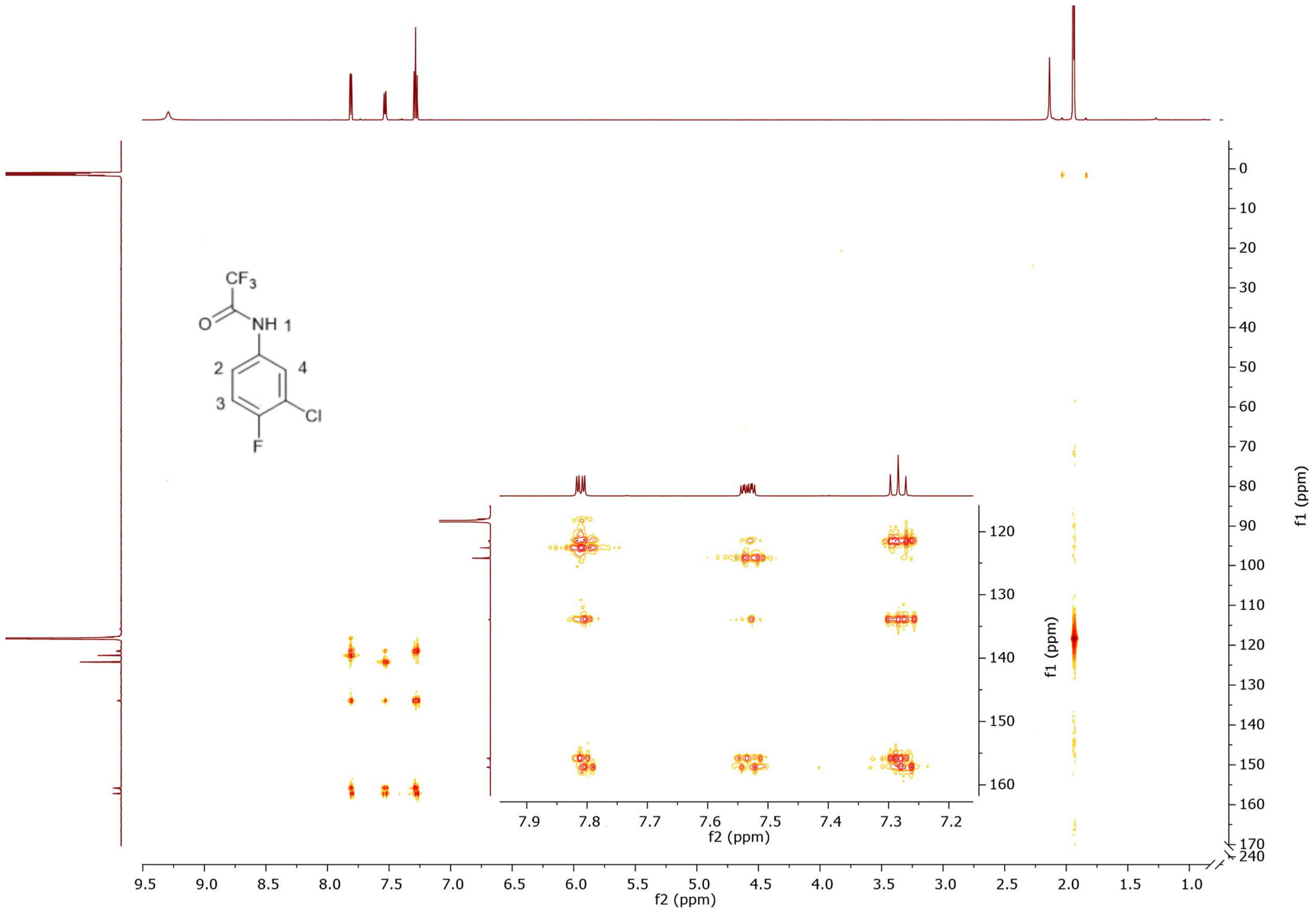

¹H NMR (400 MHz, CD_3CN) for 2,2,2-trifluoro-*N*-(3-chloro-2-fluorophenyl)acetamide **(2o)**

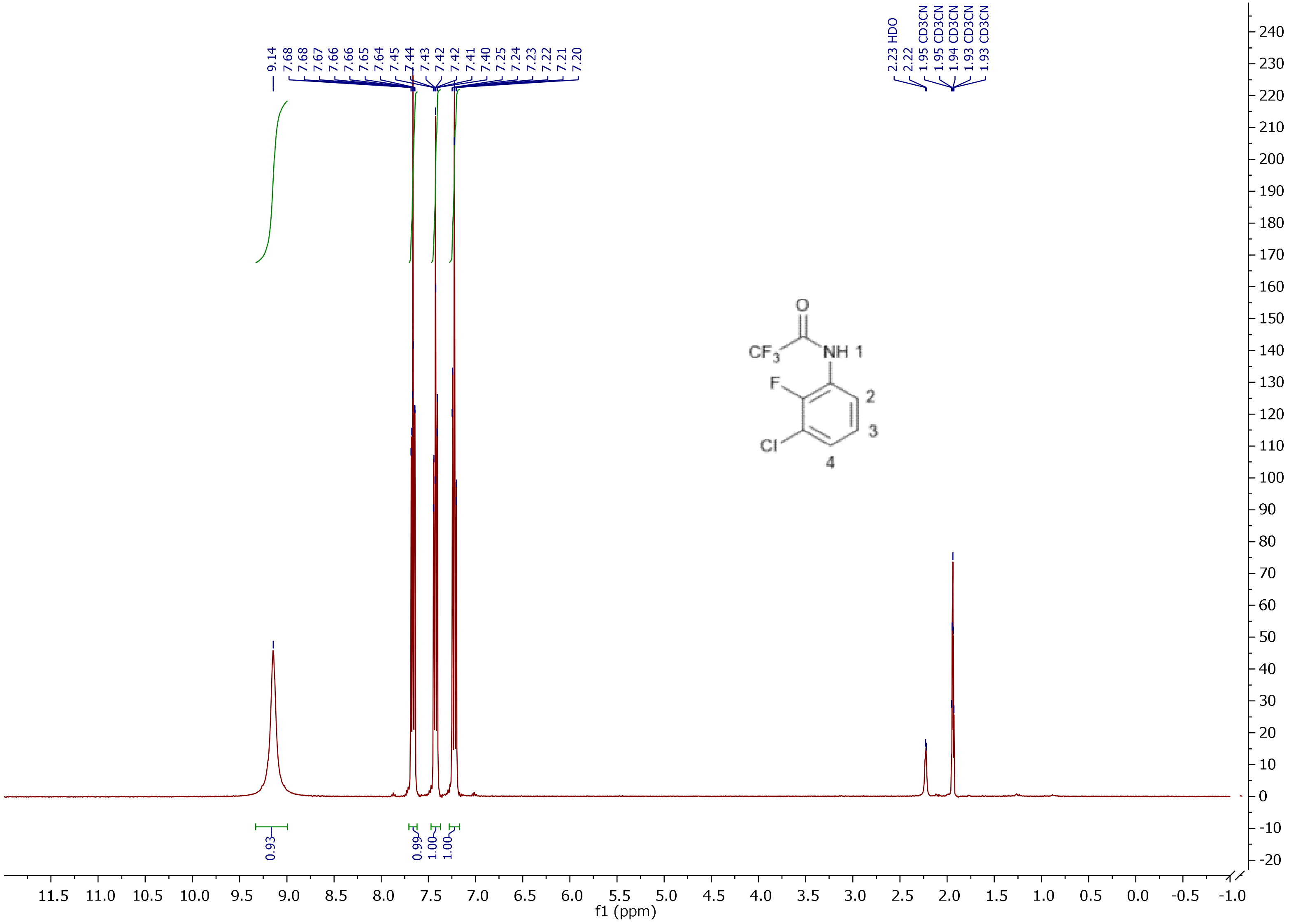

^{13}C NMR (101 MHz, CD_3CN) for 2,2,2-trifluoro-*N*-(3-chloro-2-fluorophenyl)acetamide **(2o)**

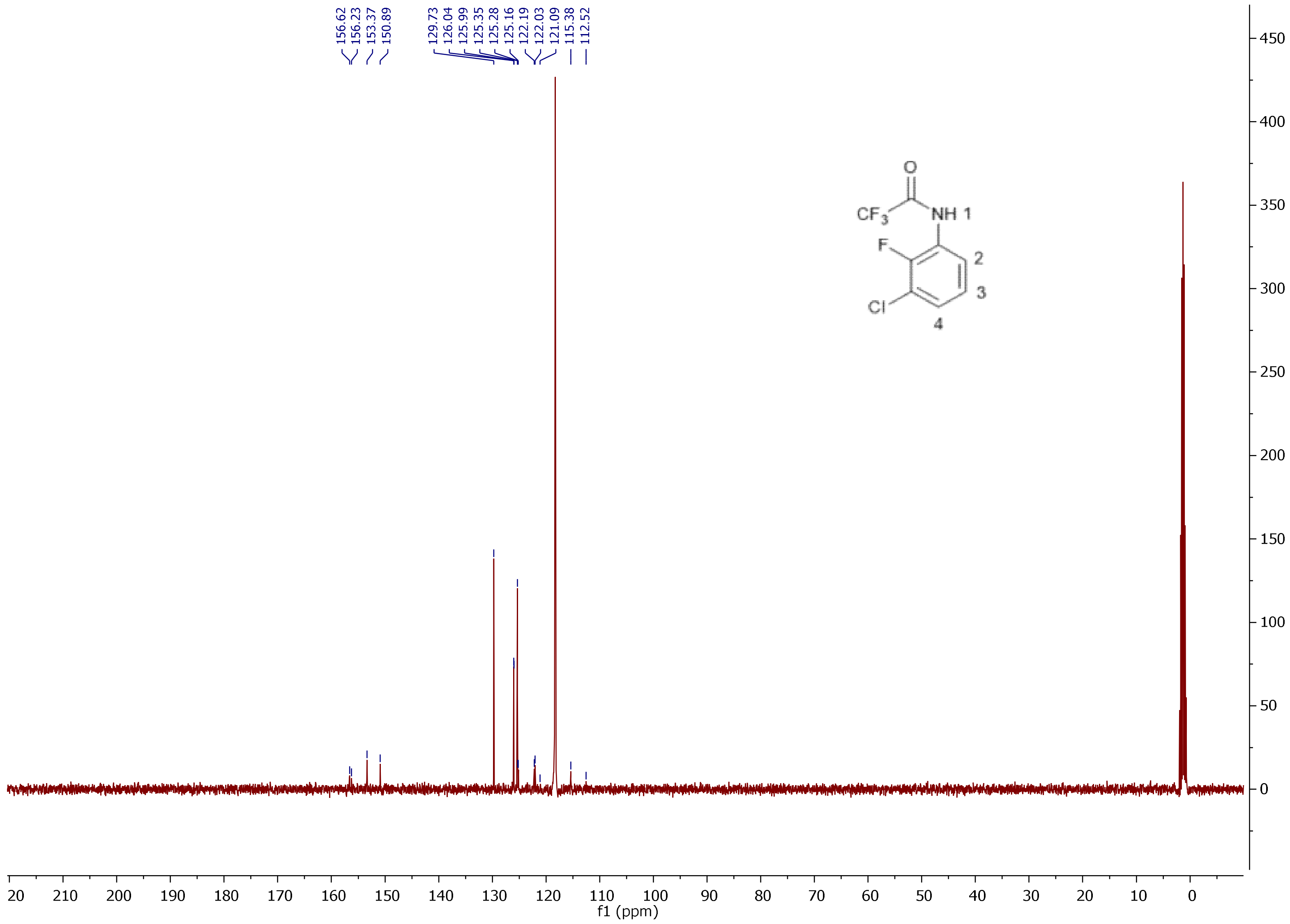

$^{19}F\{^{1}H\}$ NMR (376 MHz, CD_3CN) for 2,2,2-trifluoro-*N*-(3-chloro-2-fluorophenyl)acetamide **(2o)**

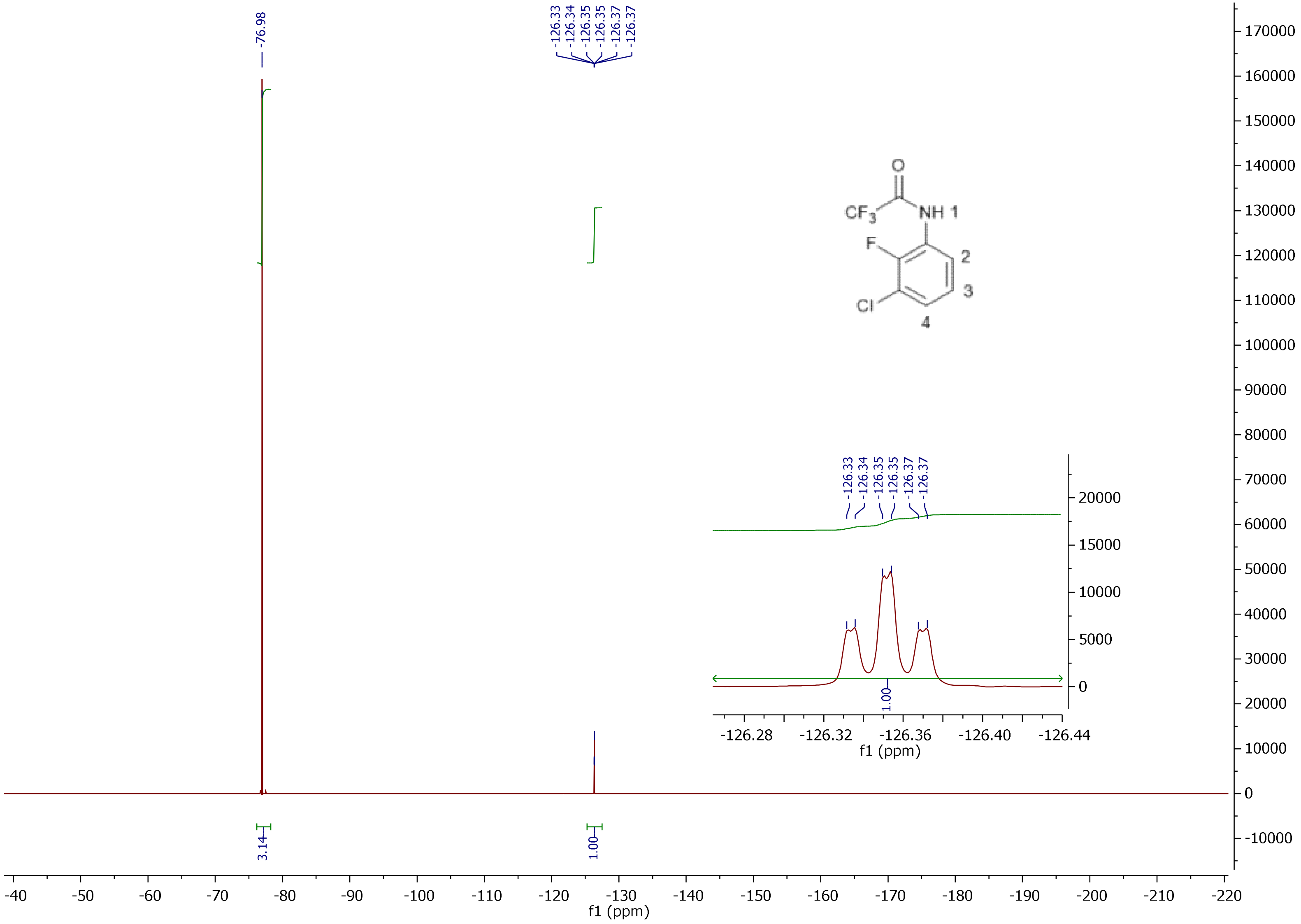

^{1}H NMR (700 MHz, CD_3CN) for 2,2,2-trifluoro-*N*-(5-chloro-2-fluorophenyl)acetamide **(2o')**

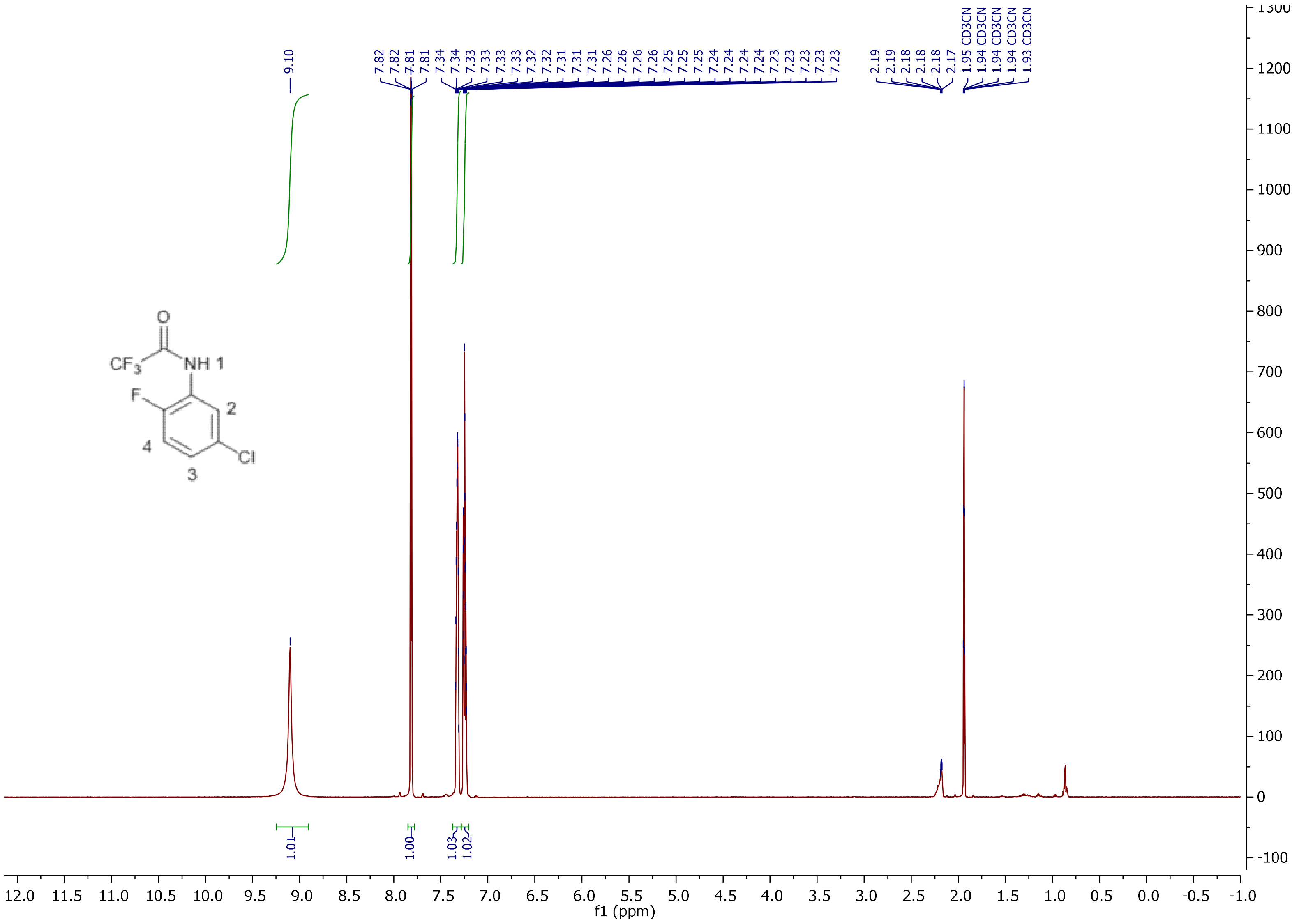

^{13}C NMR (176 MHz, CD_3CN) for 2,2,2-trifluoro-*N*-(5-chloro-2-fluorophenyl)acetamide **(2o')**

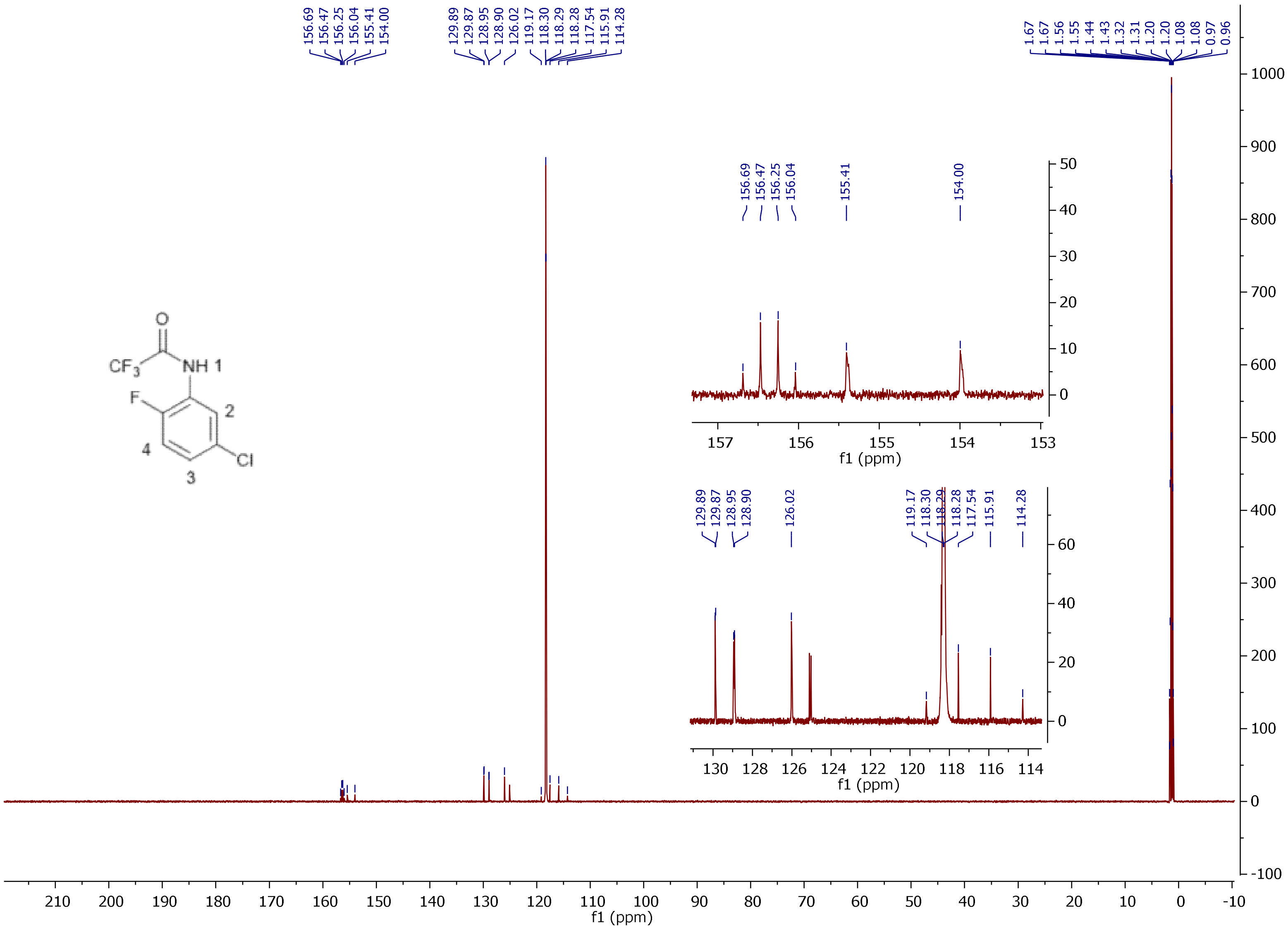

^{19}F{^{1}H} NMR (376 MHz, CD_3CN) for 2,2,2-trifluoro-*N*-(5-chloro-2-fluorophenyl)acetamide **(2o')**

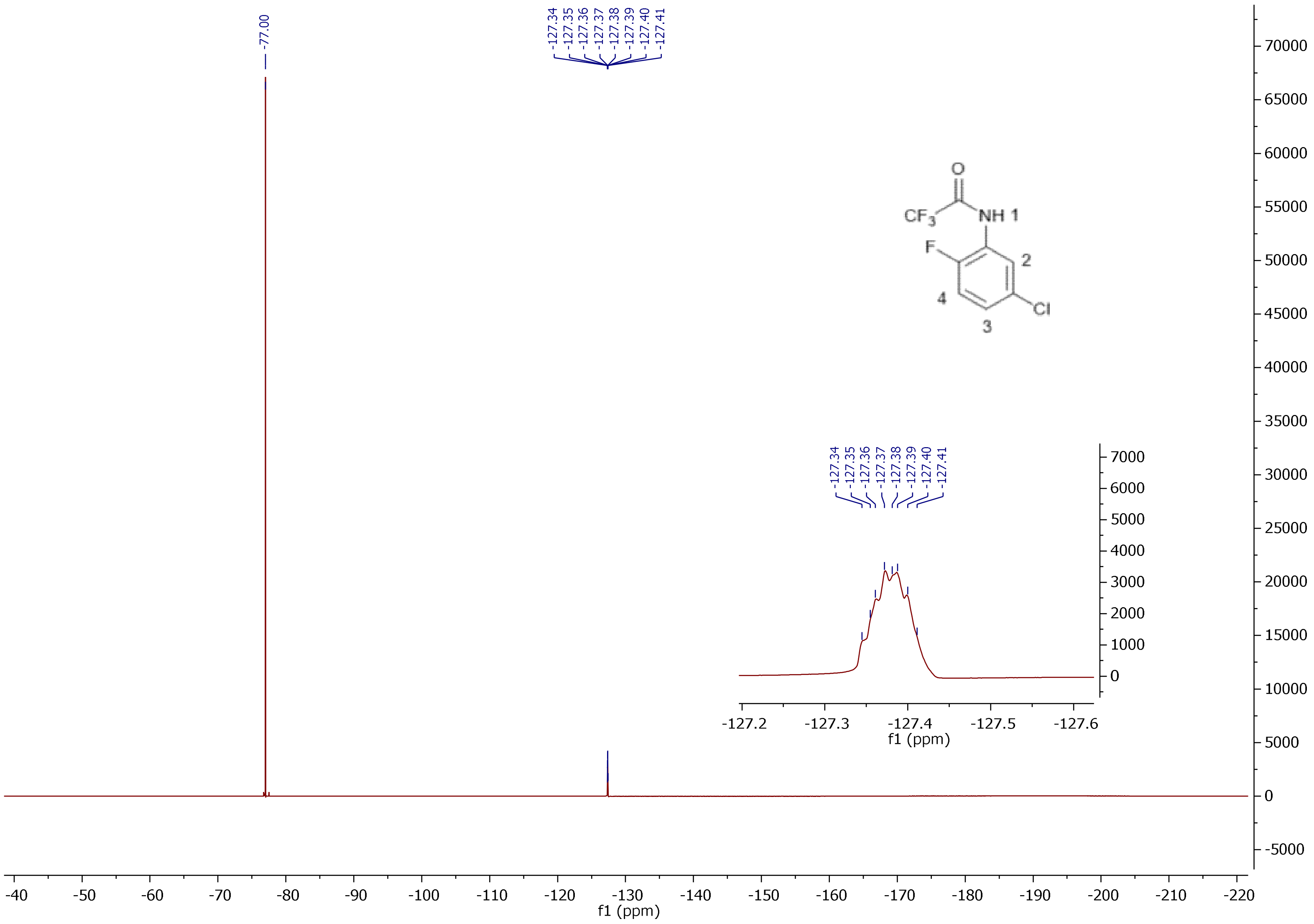

^{1}H NMR (700 MHz, CD_3CN) for fluorination of *N*-(3-bromophenyl)-2,2,2-trifluoroacetamide **(3p)**

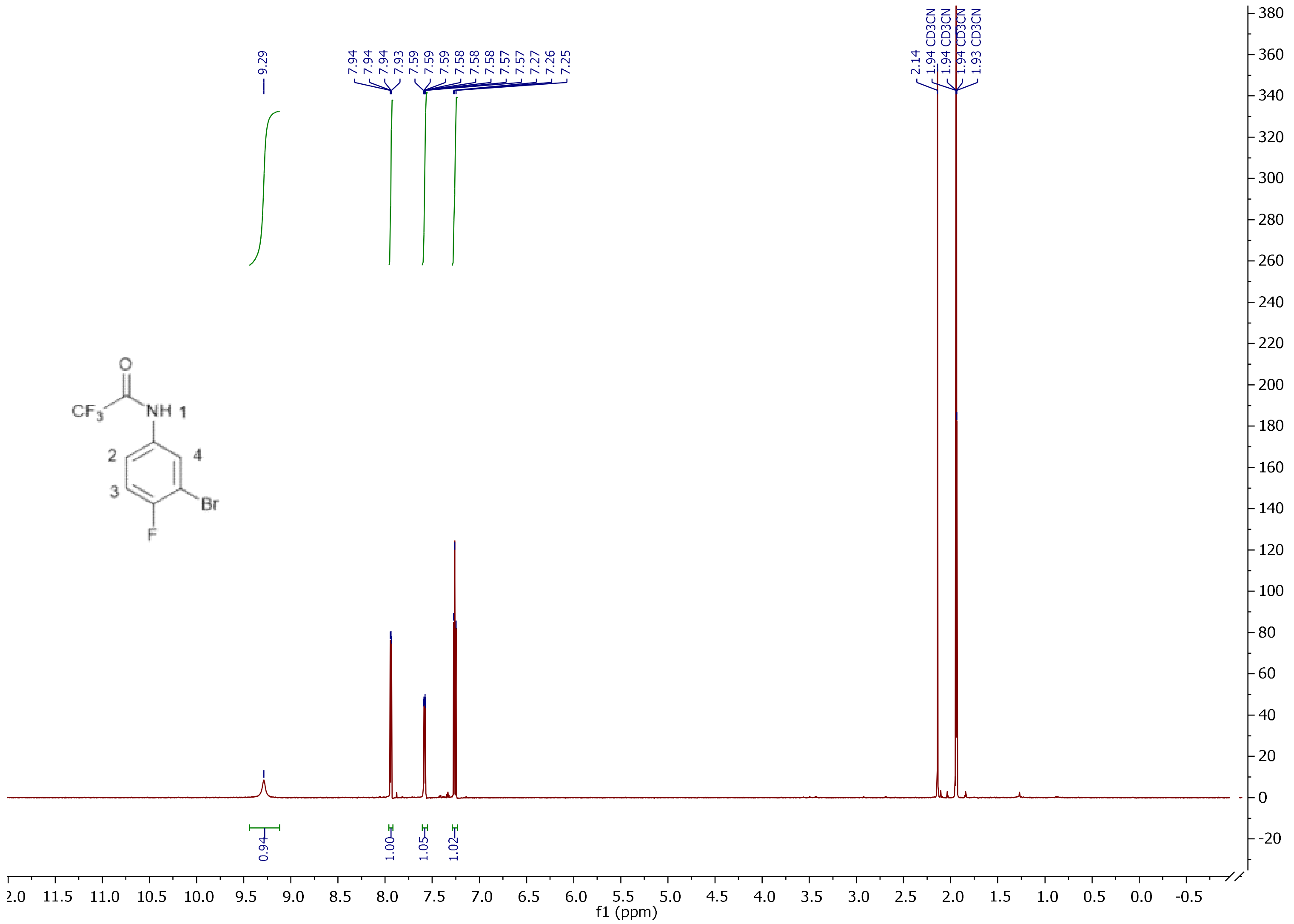

^{13}C NMR (176 MHz, CD_3CN) for fluorination of *N*-(3-bromophenyl)-2,2,2-trifluoroacetamide **(3p)**

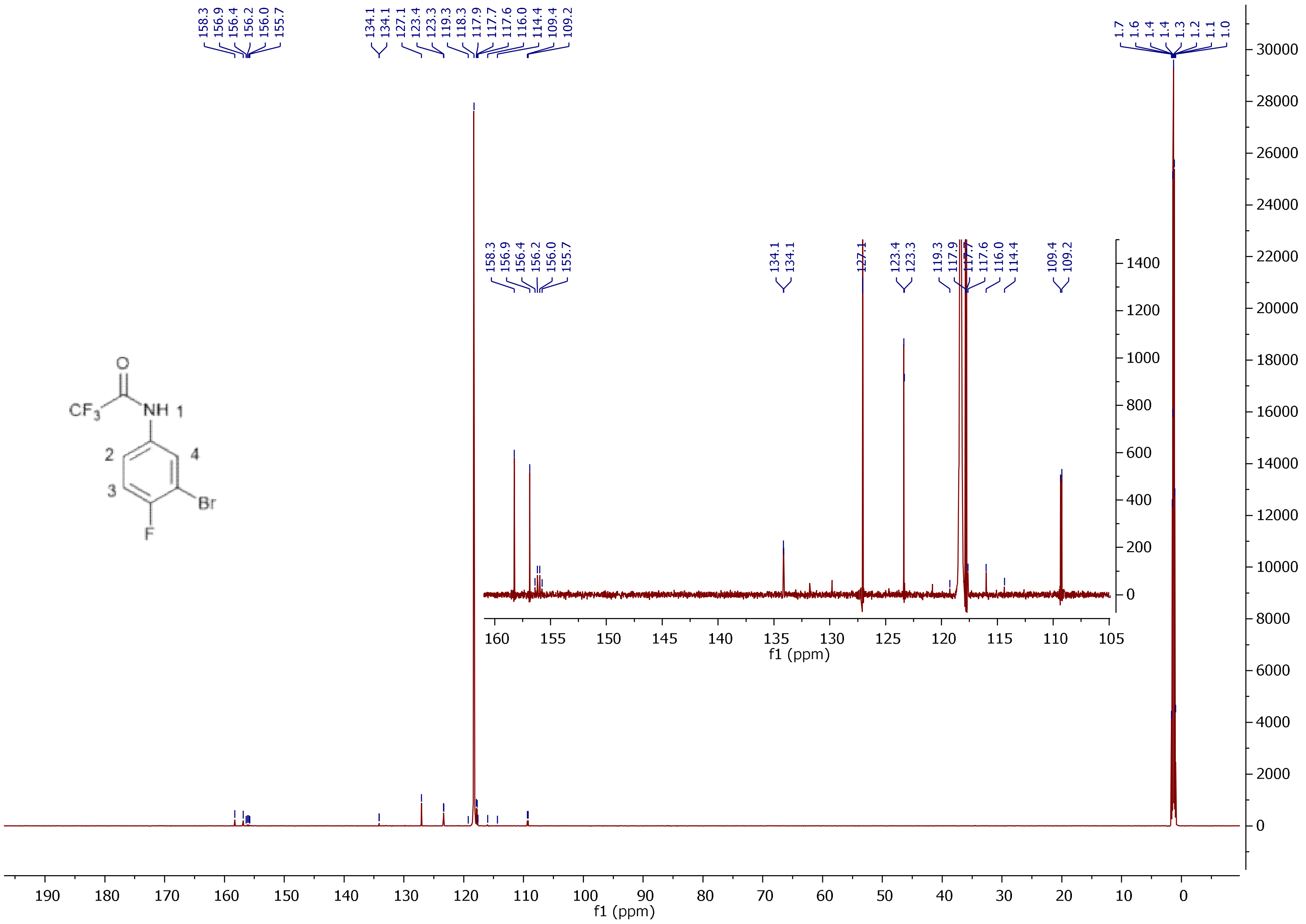

$^{19}F\{^{1}H\}$ NMR (376 MHz, CD_3CN) for fluorination of *N*-(3-bromophenyl)-2,2,2-trifluoroacetamide **(3p)**

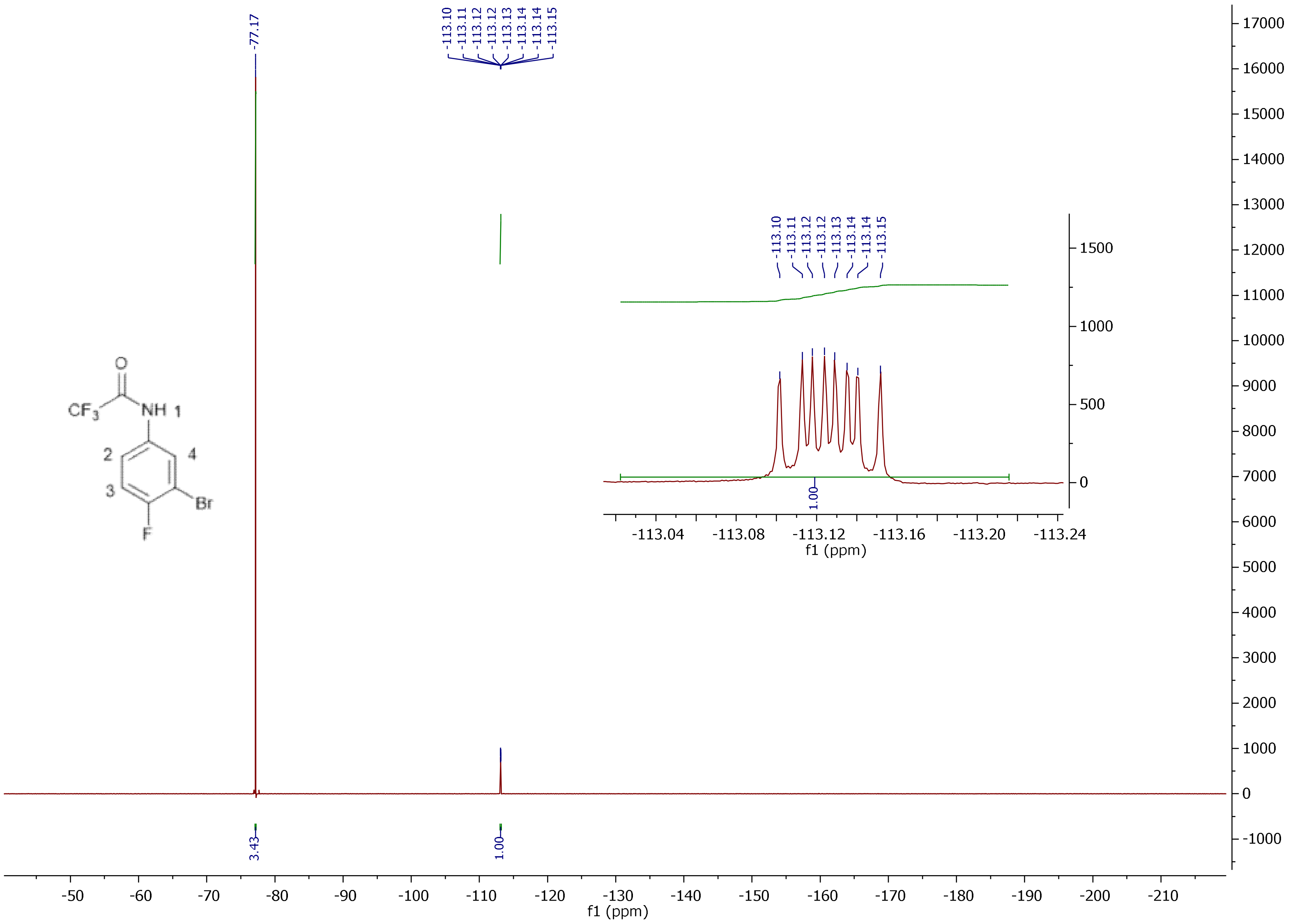

HMBC NMR (CD_3CN) for fluorination of *N*-(3-bromophenyl)-2,2,2-trifluoroacetamide **(3p)**

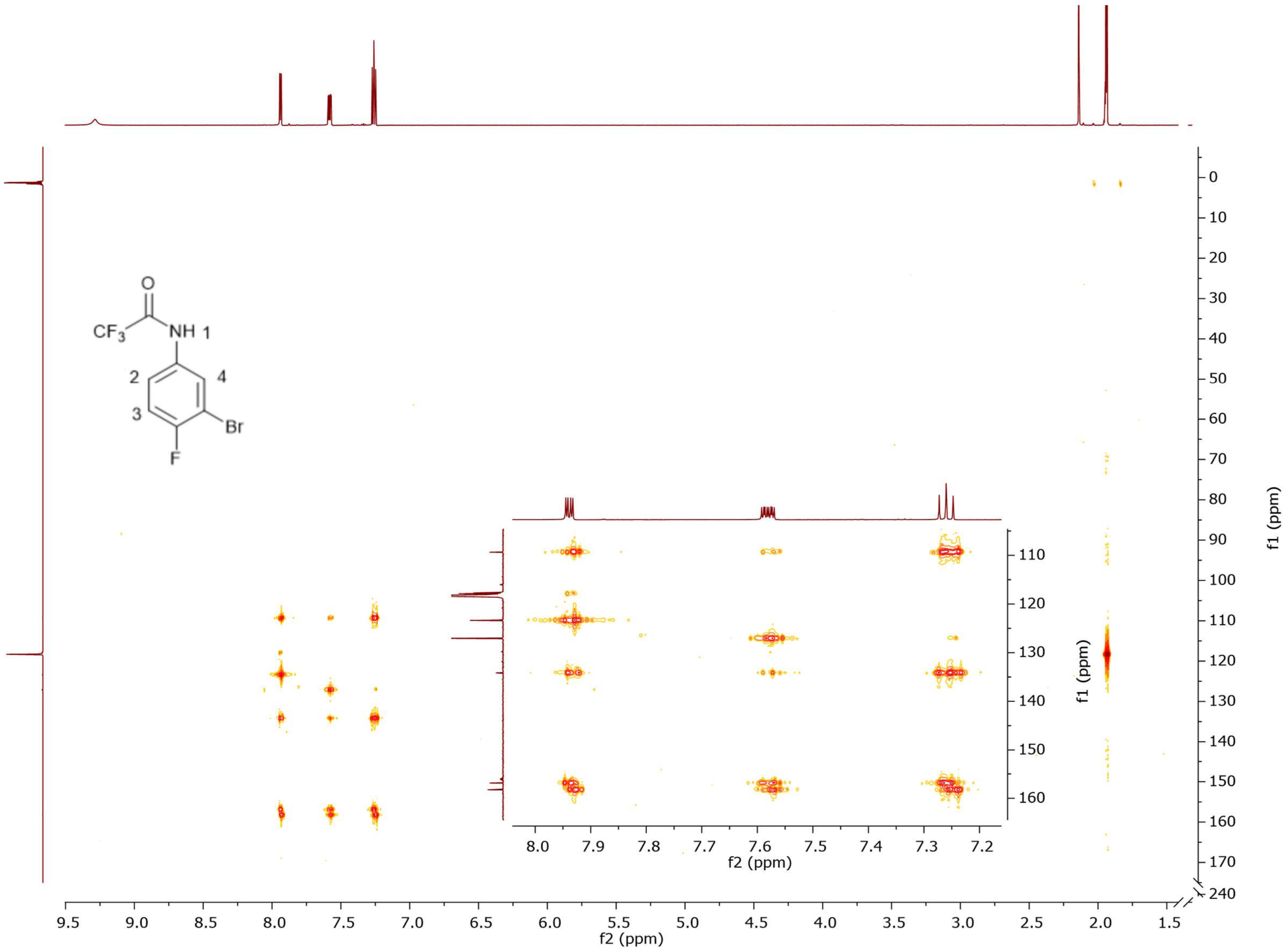

^{1}H NMR (700 MHz, CD_3CN) for fluorination of *N*-(3,5-dichlorophenyl)-2,2,2-trifluoroacetamide **(3q)**

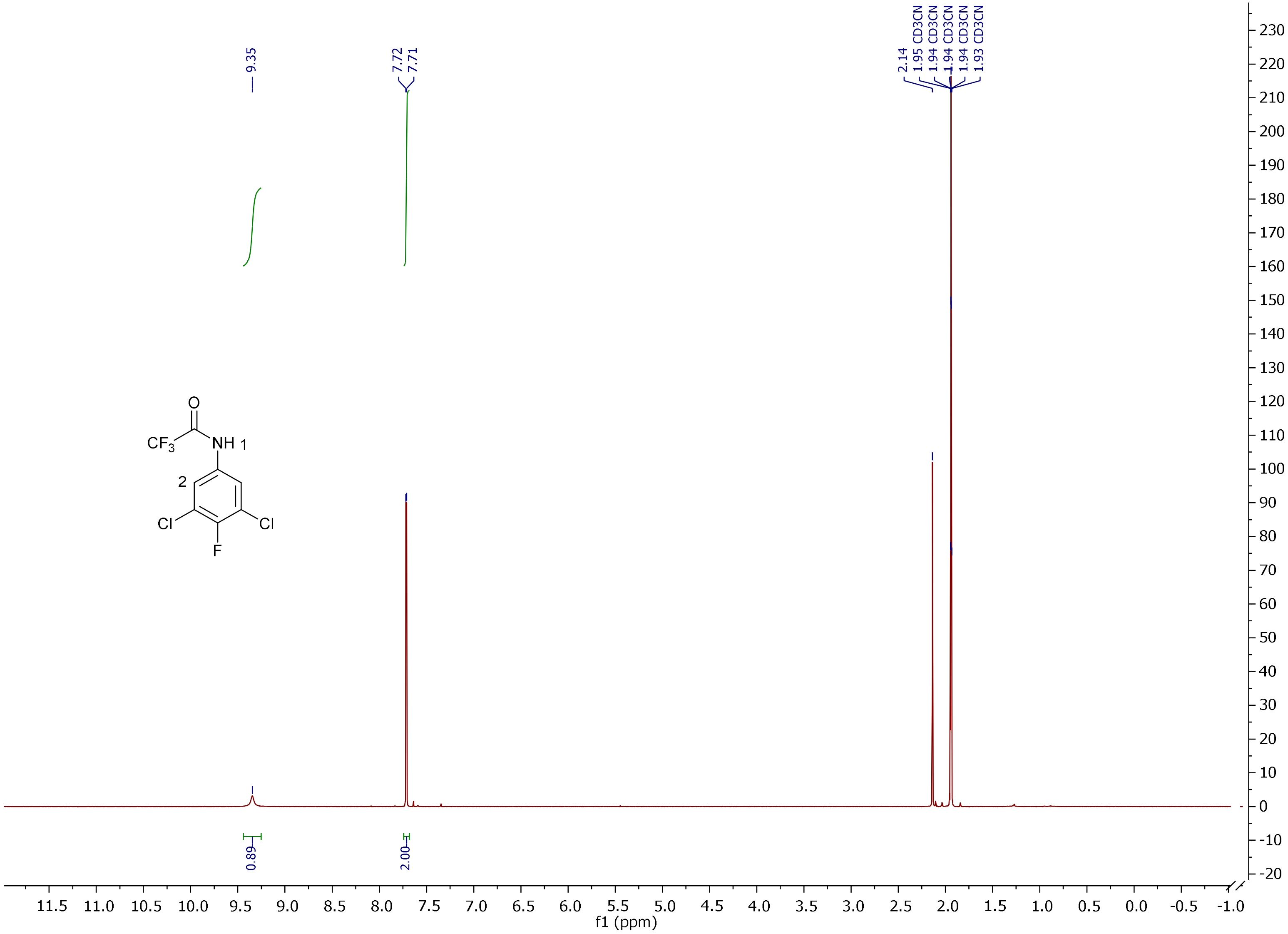

^{13}C NMR (176 MHz, CD_3CN) for fluorination of *N*-(3,5-dichlorophenyl)-2,2,2-trifluoroacetamide **(3q)**

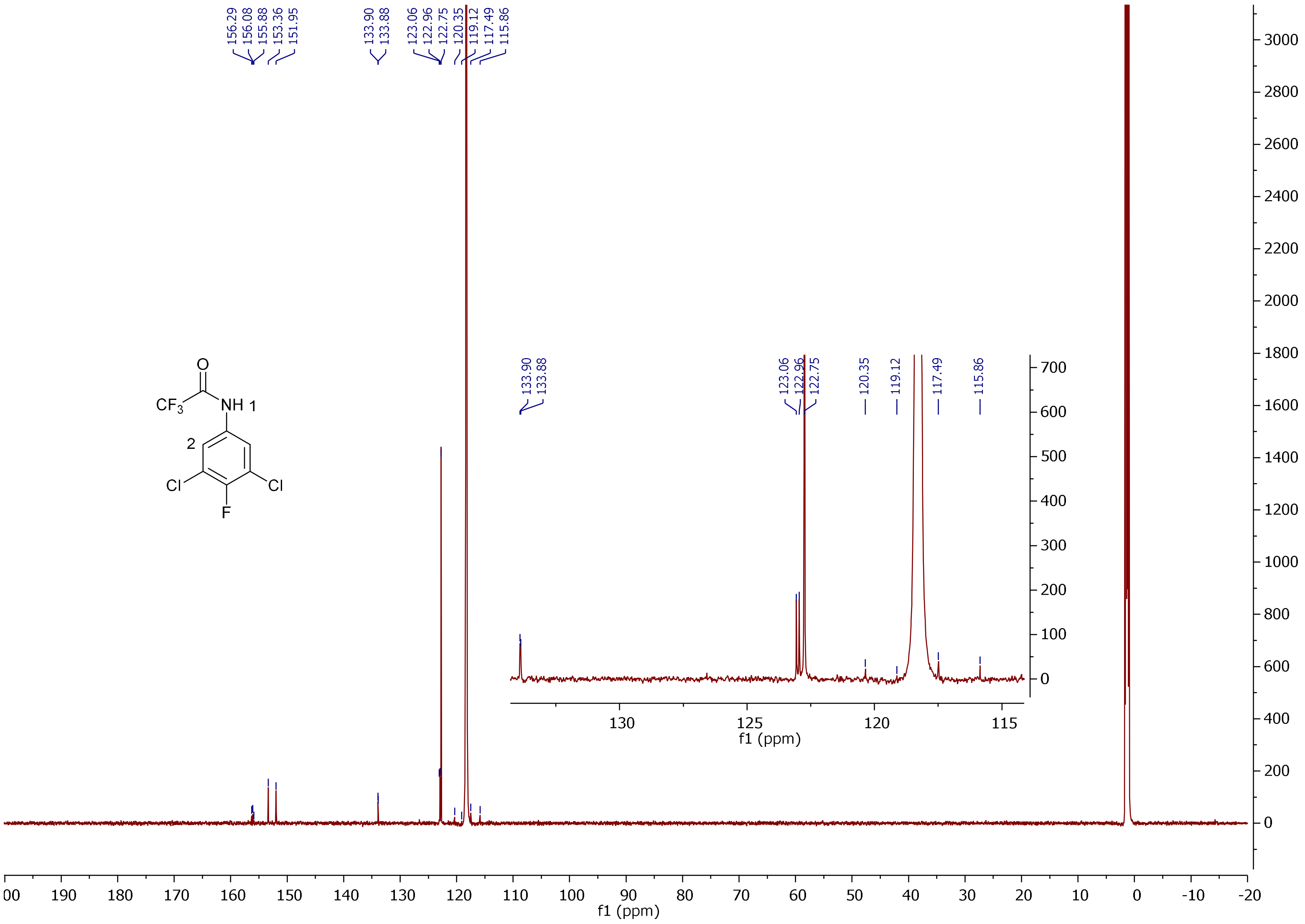

^{19}F{^{1}H} NMR (376 MHz, CD_3CN) for fluorination of *N*-(3,5-dichlorophenyl)-2,2,2-trifluoroacetamide **(3q)**

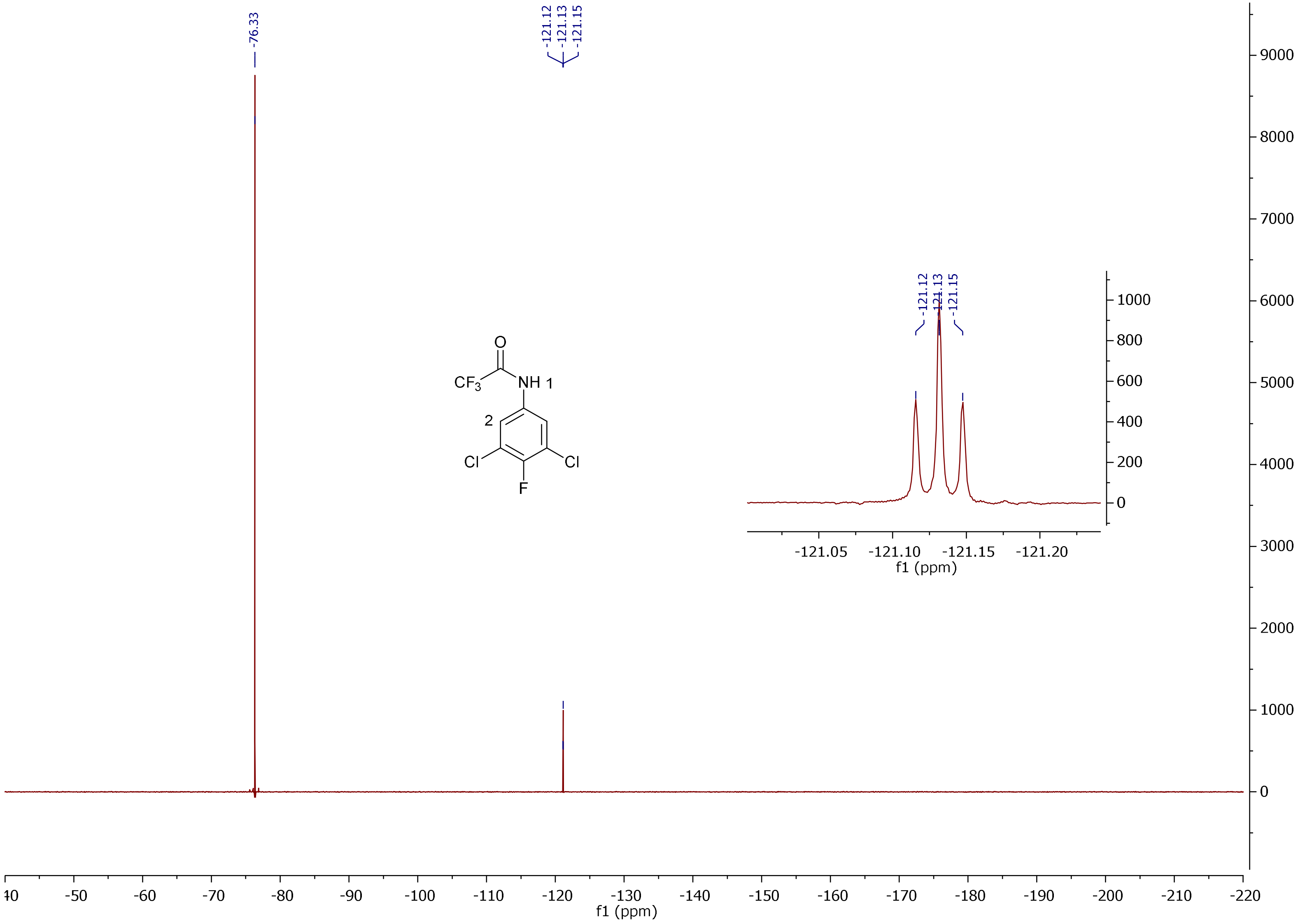

^{1}H NMR (700 MHz, CD_3CN) for fluorination of *N*-(2-bromo-3-methylphenyl)-2,2,2-trifluoroacetamide **(3r)**

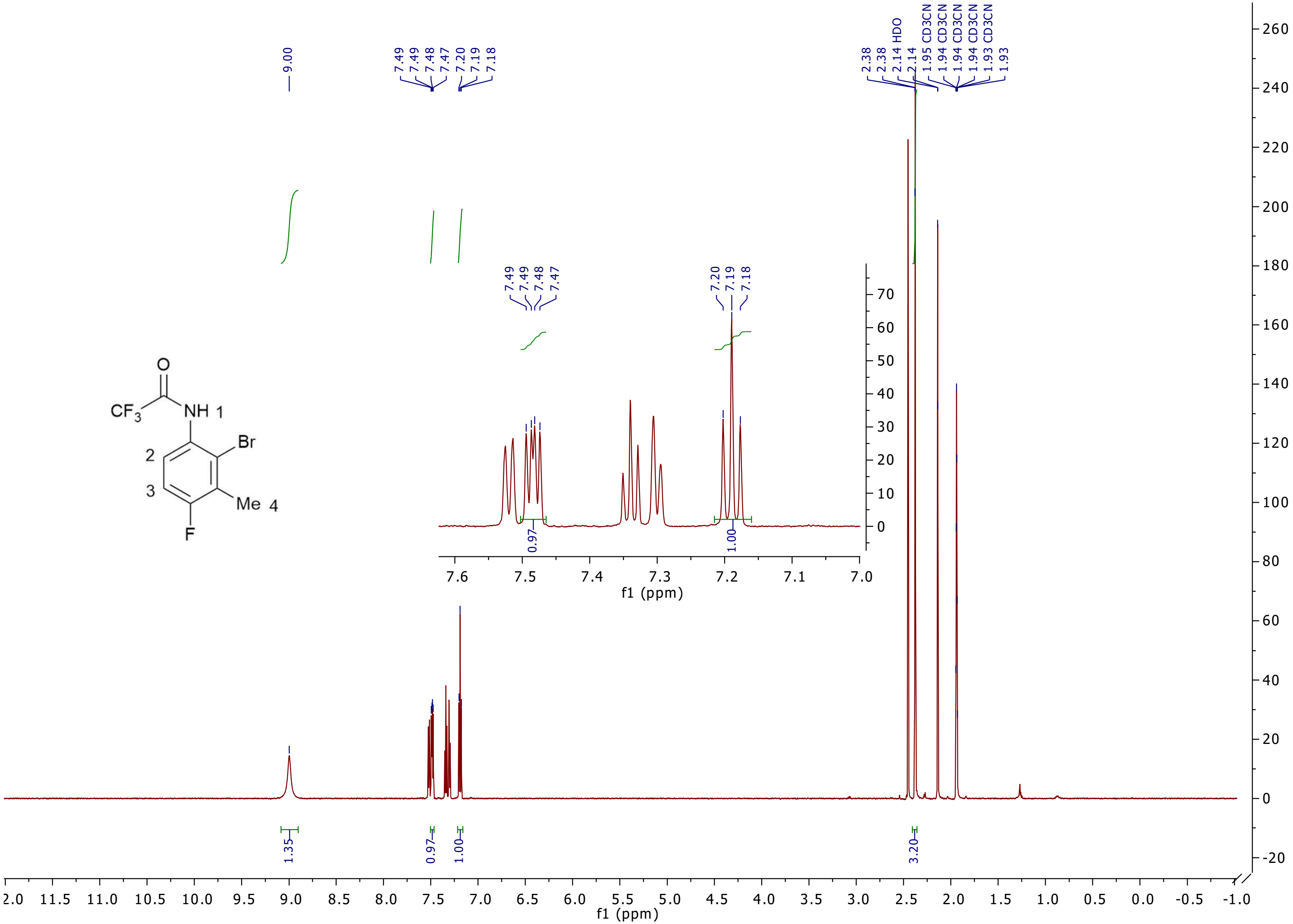

^{13}C NMR (176 MHz, CD_3CN) for fluorination of *N*-(2-bromo-3-methylphenyl)-2,2,2-trifluoroacetamide **(3r)**

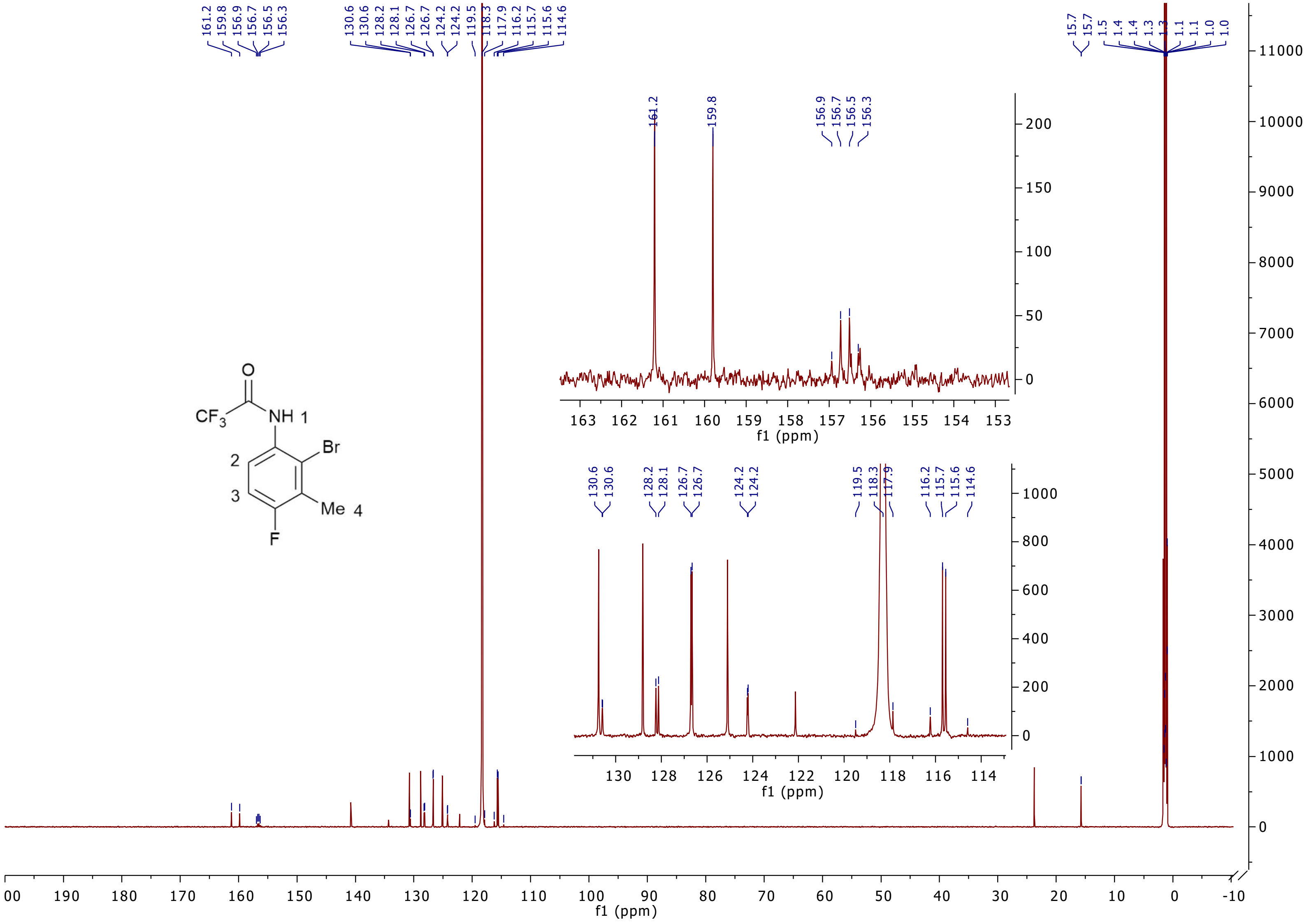

^{19}F{^{1}H} NMR (376 MHz, CD_3CN) for fluorination of *N*-(2-bromo-3-methylphenyl)-2,2,2-trifluoroacetamide **(3r)**

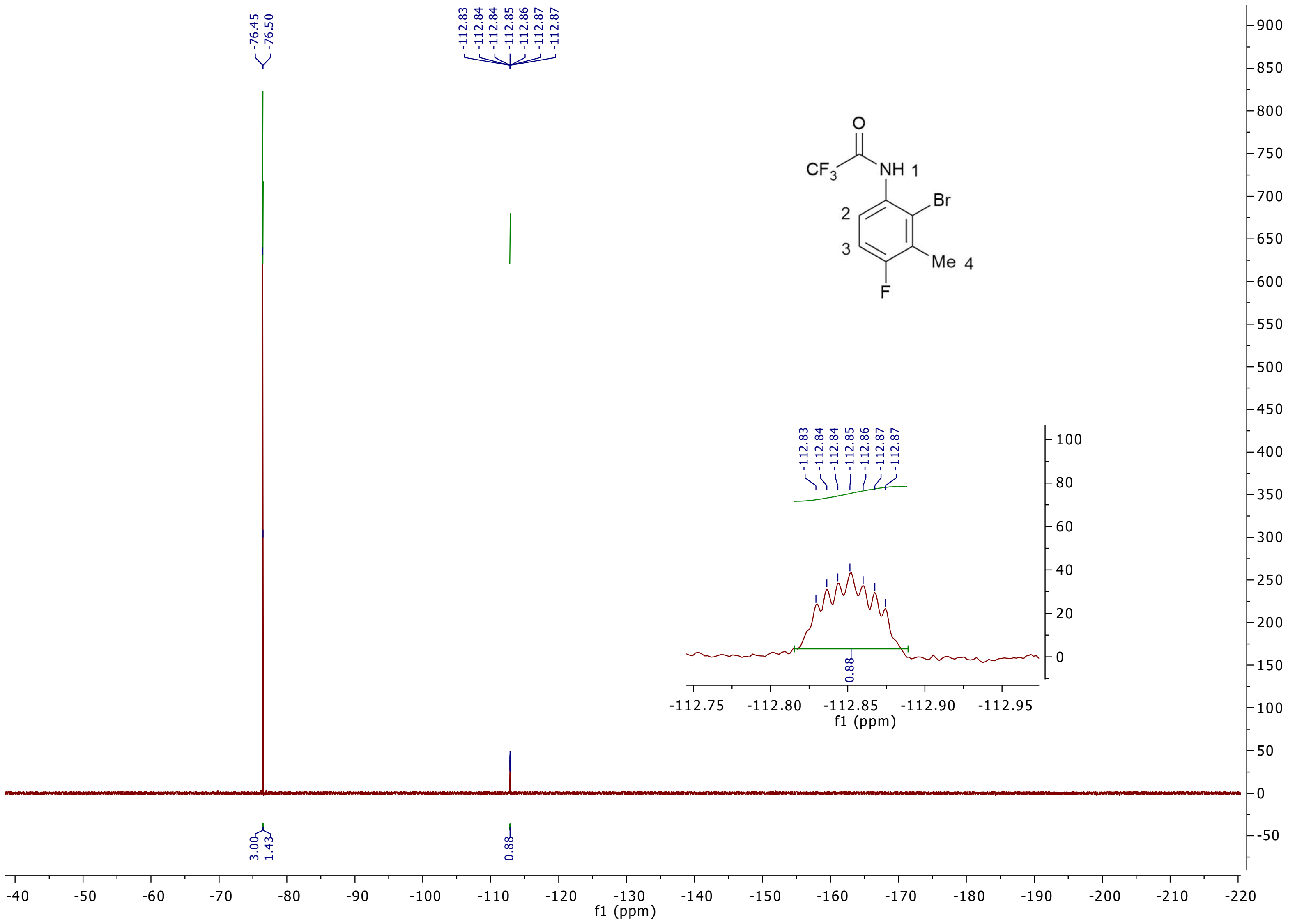

HMBC NMR (CD_3CN) for fluorination of *N*-(2-bromo-3-methylphenyl)-2,2,2-trifluoroacetamide **(3r)**

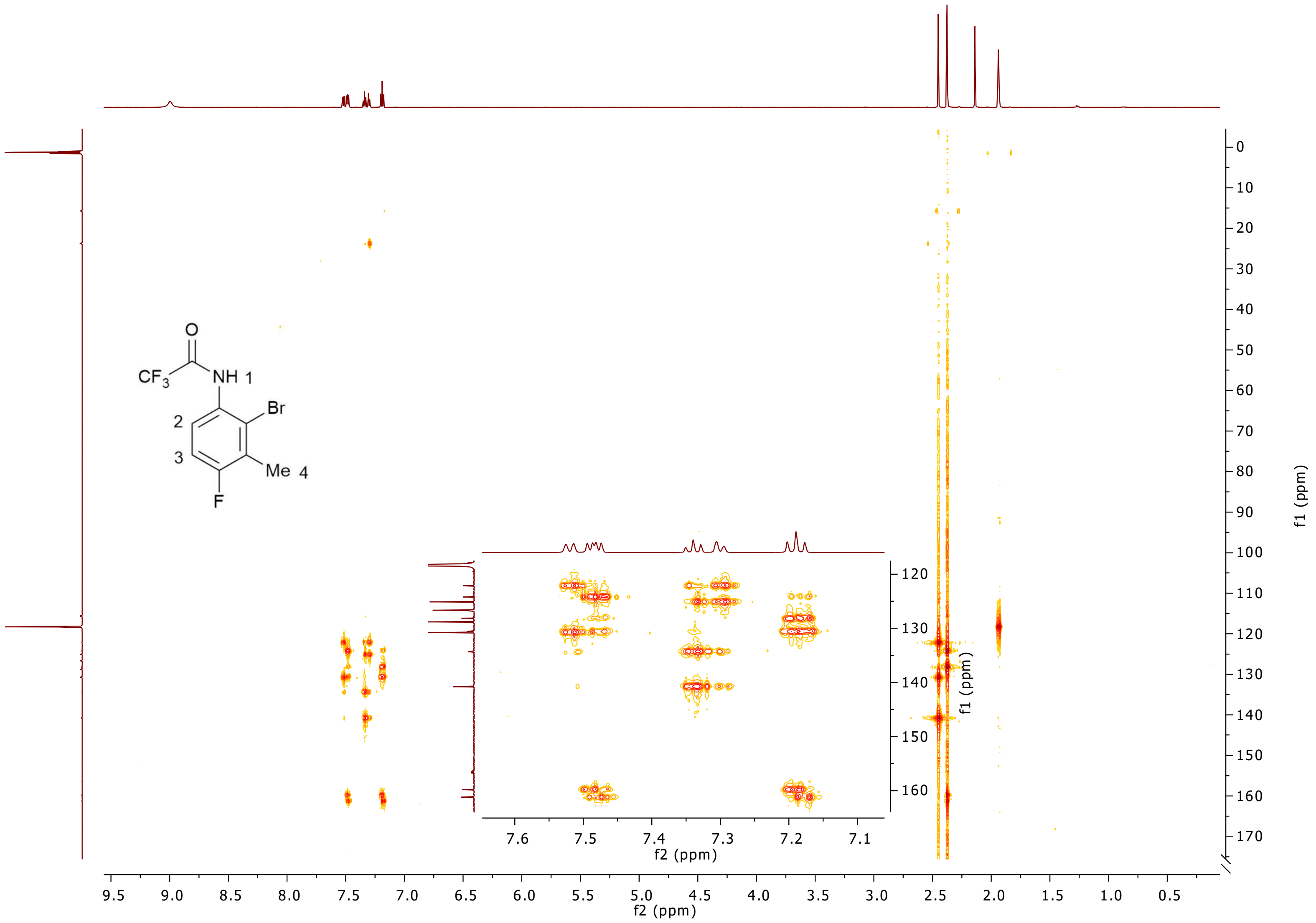

^{1}H NMR (700 MHz, CD_3CN) for fluorination of 2,2,2-trifluoro-*N*-(3-(trifluoromethyl)phenyl)acetamide **(3s)**

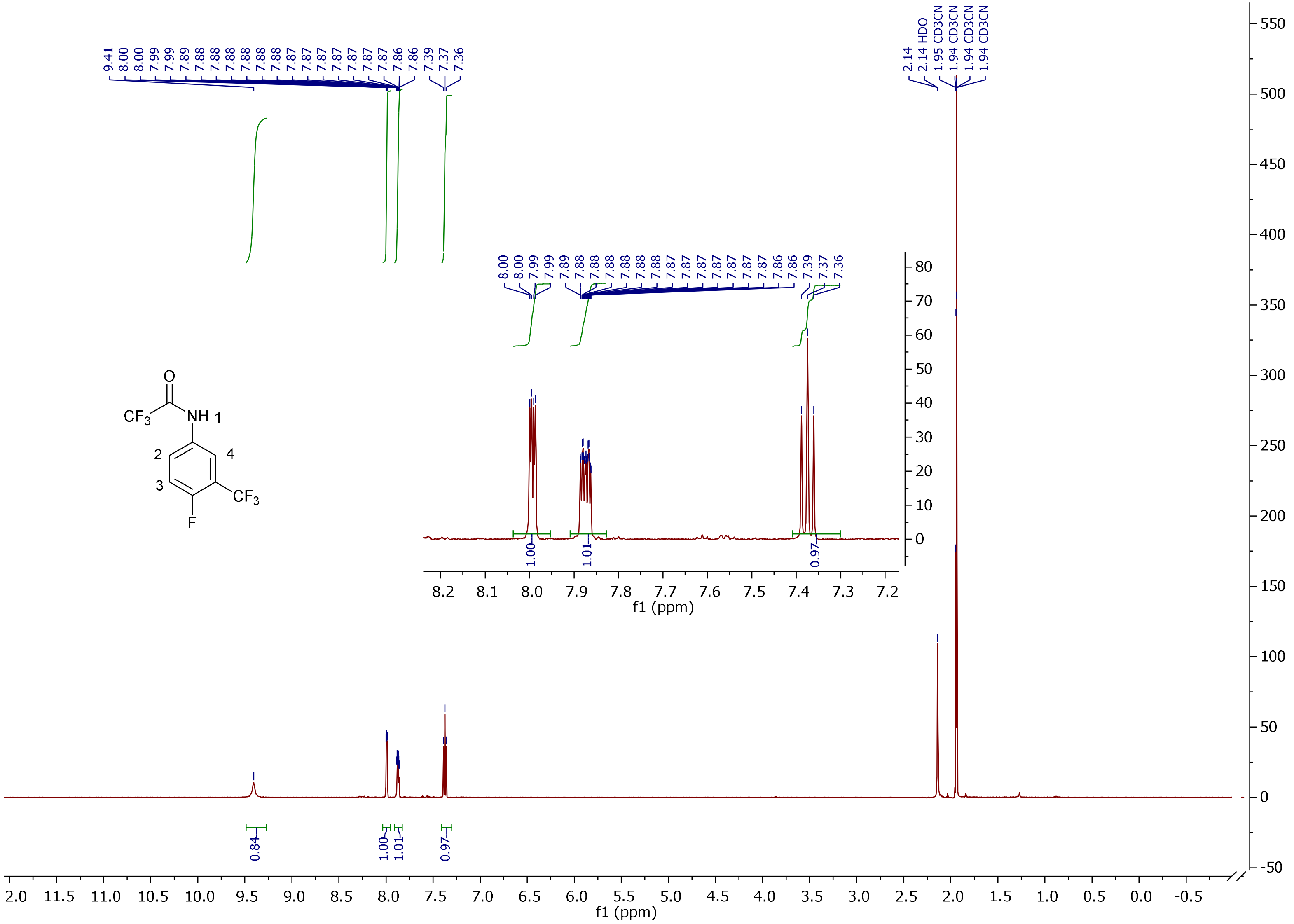

^{13}C NMR (176 MHz, CD_3CN) for fluorination of 2,2,2-trifluoro-*N*-(3-(trifluoromethyl)phenyl)acetamide **(3s)**

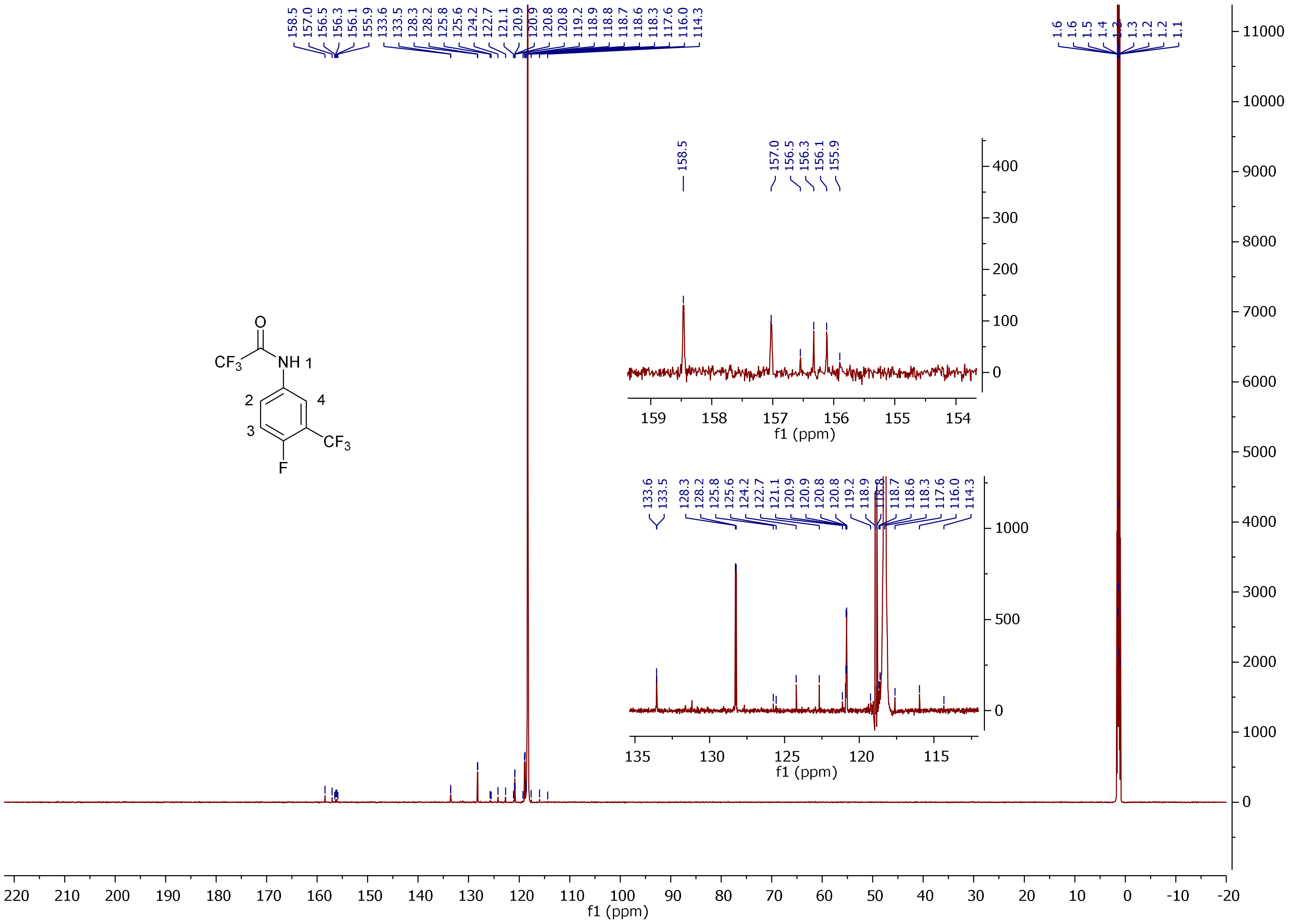

^{19}F{^{1}H} NMR (376 MHz, CD_3CN) for fluorination of 2,2,2-trifluoro-*N*-(3-(trifluoromethyl)phenyl)acetamide **(3s)**

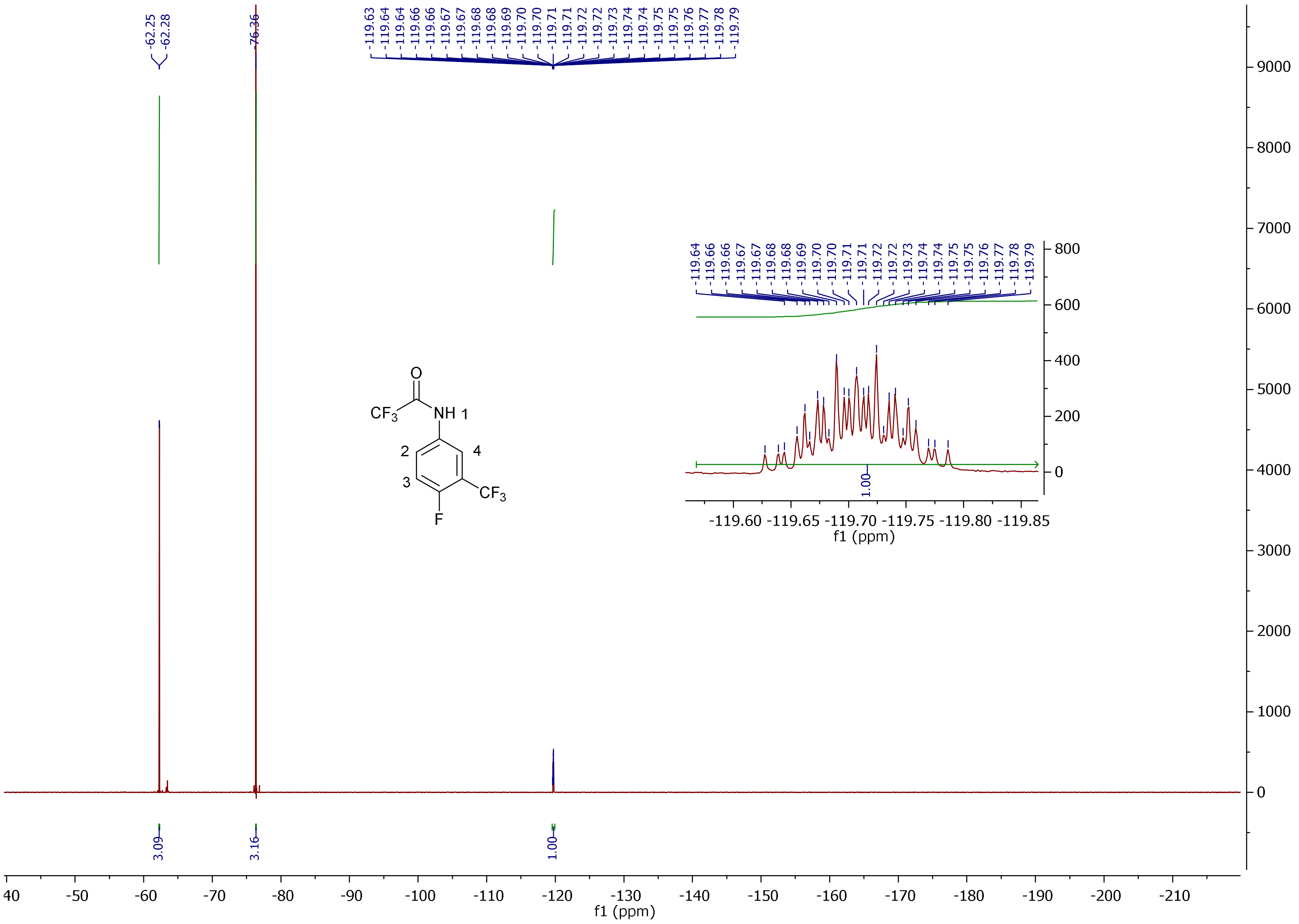

[1]**H NMR** (700 MHz, CD_3CN) for fluorination of *N*-(2-benzoylphenyl)-2,2,2-trifluoroacetamide **(3t)**

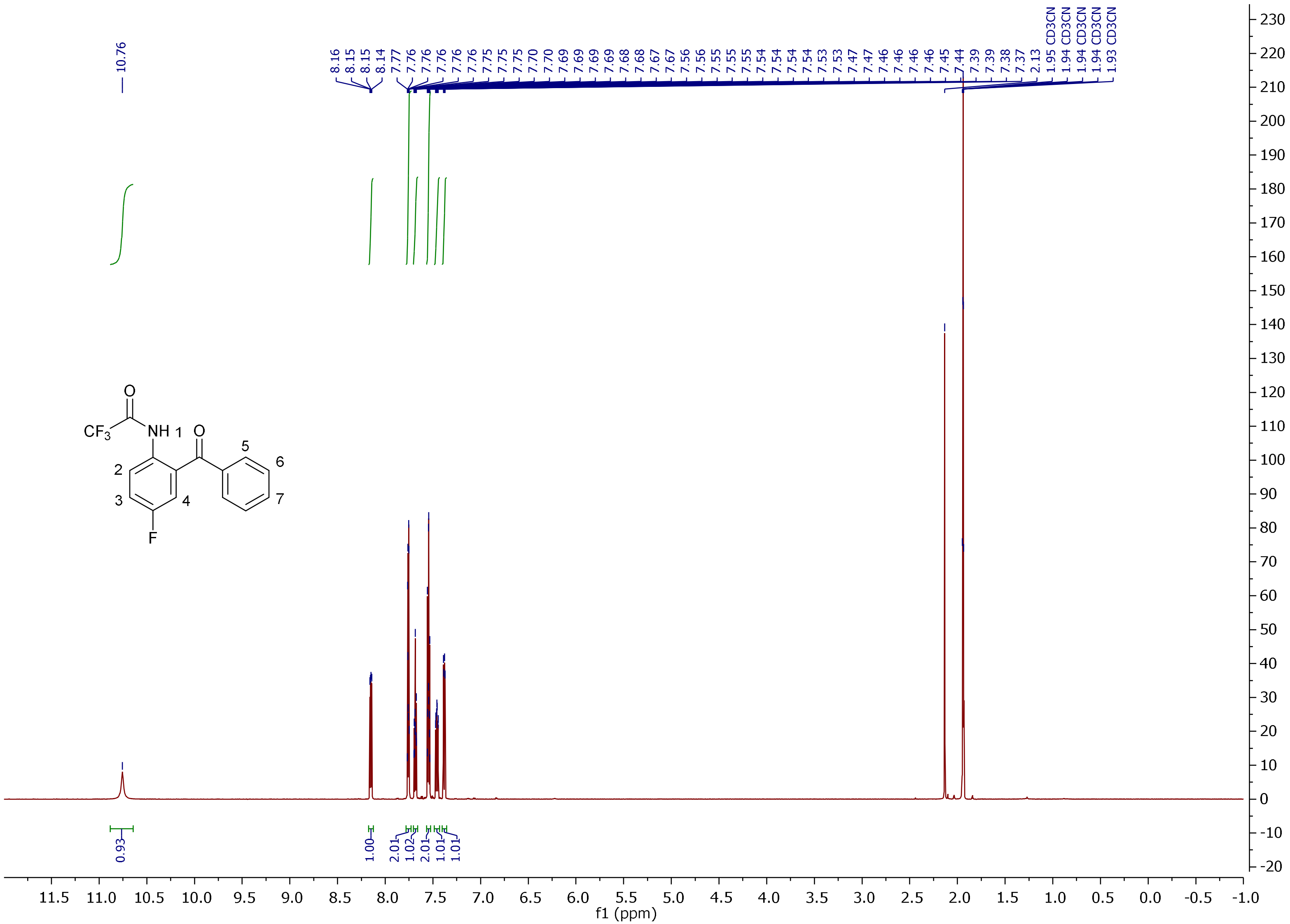

^{13}C NMR (176 MHz, CD_3CN) for fluorination of *N*-(2-benzoylphenyl)-2,2,2-trifluoroacetamide **(3t)**

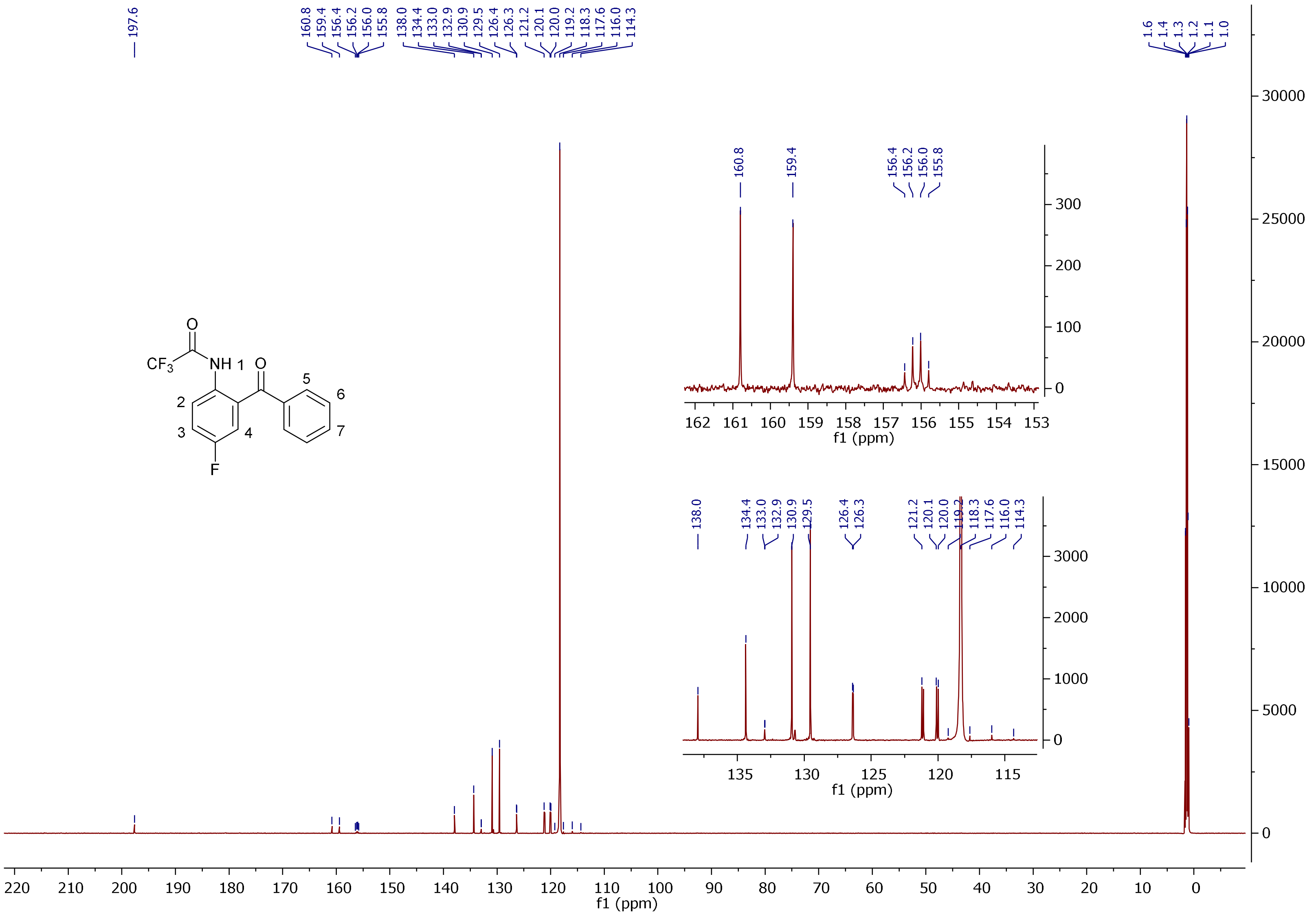

^{19}F{^{1}H} NMR (376 MHz, CD_3CN) for fluorination of *N*-(2-benzoylphenyl)-2,2,2-trifluoroacetamide **(3t)**

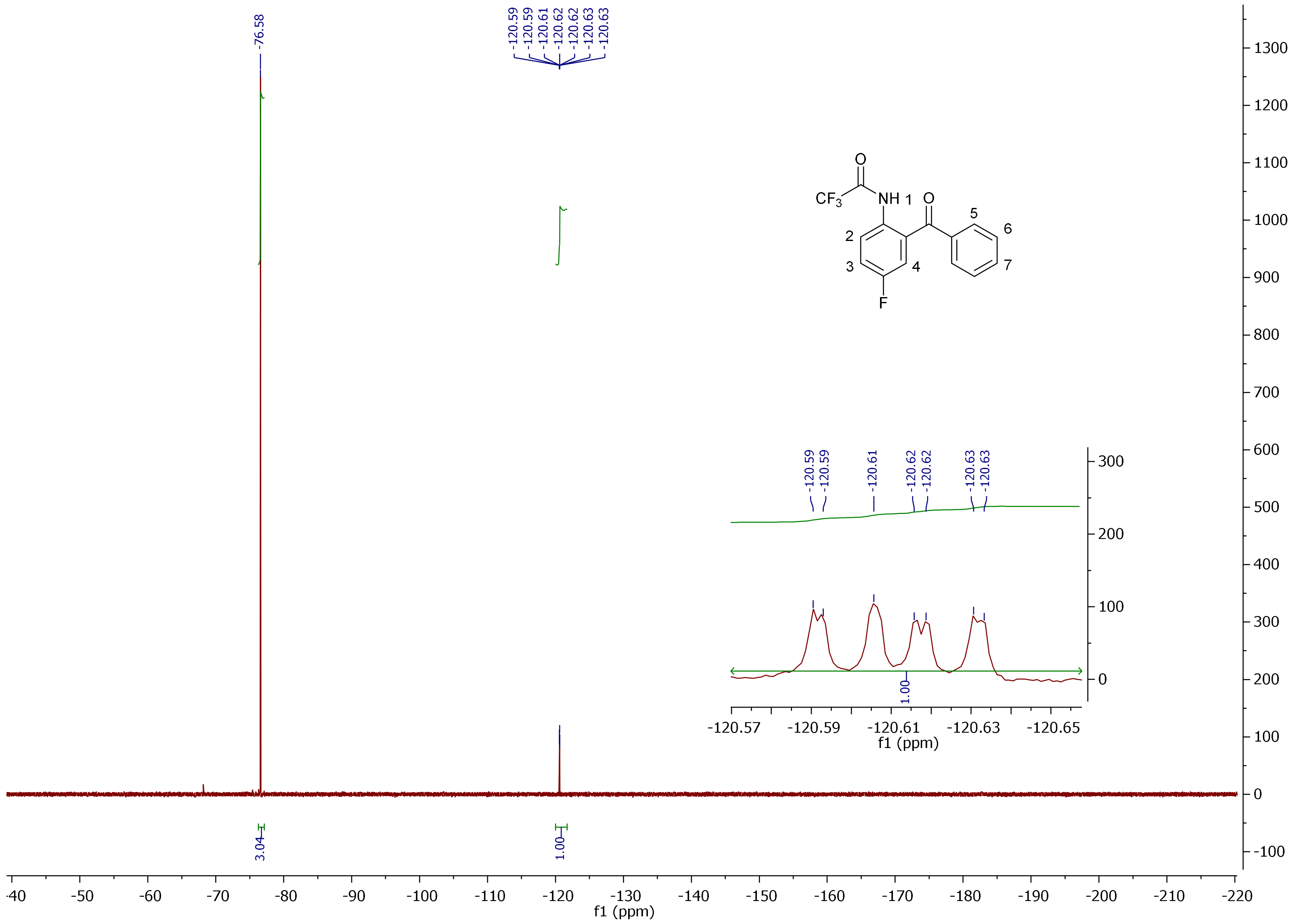

COSY NMR (CD_3CN) for fluorination of *N*-(2-benzoylphenyl)-2,2,2-trifluoroacetamide **(3t)**

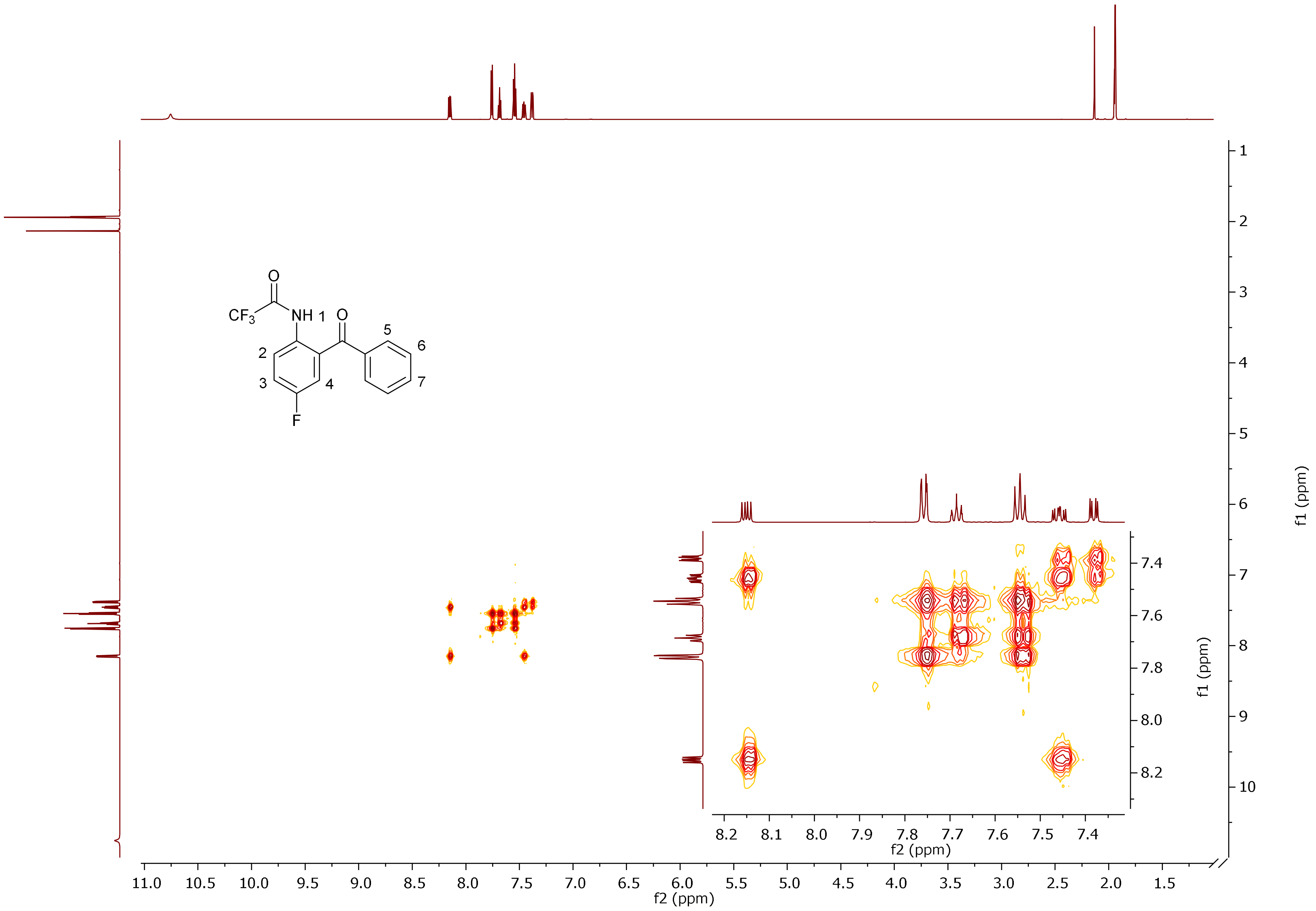

^{1}H NMR (700 MHz, CD_3CN) for fluorination of *N*-(2-benzoylphenyl)-2,2,2-trifluoroacetamide **(4t)**

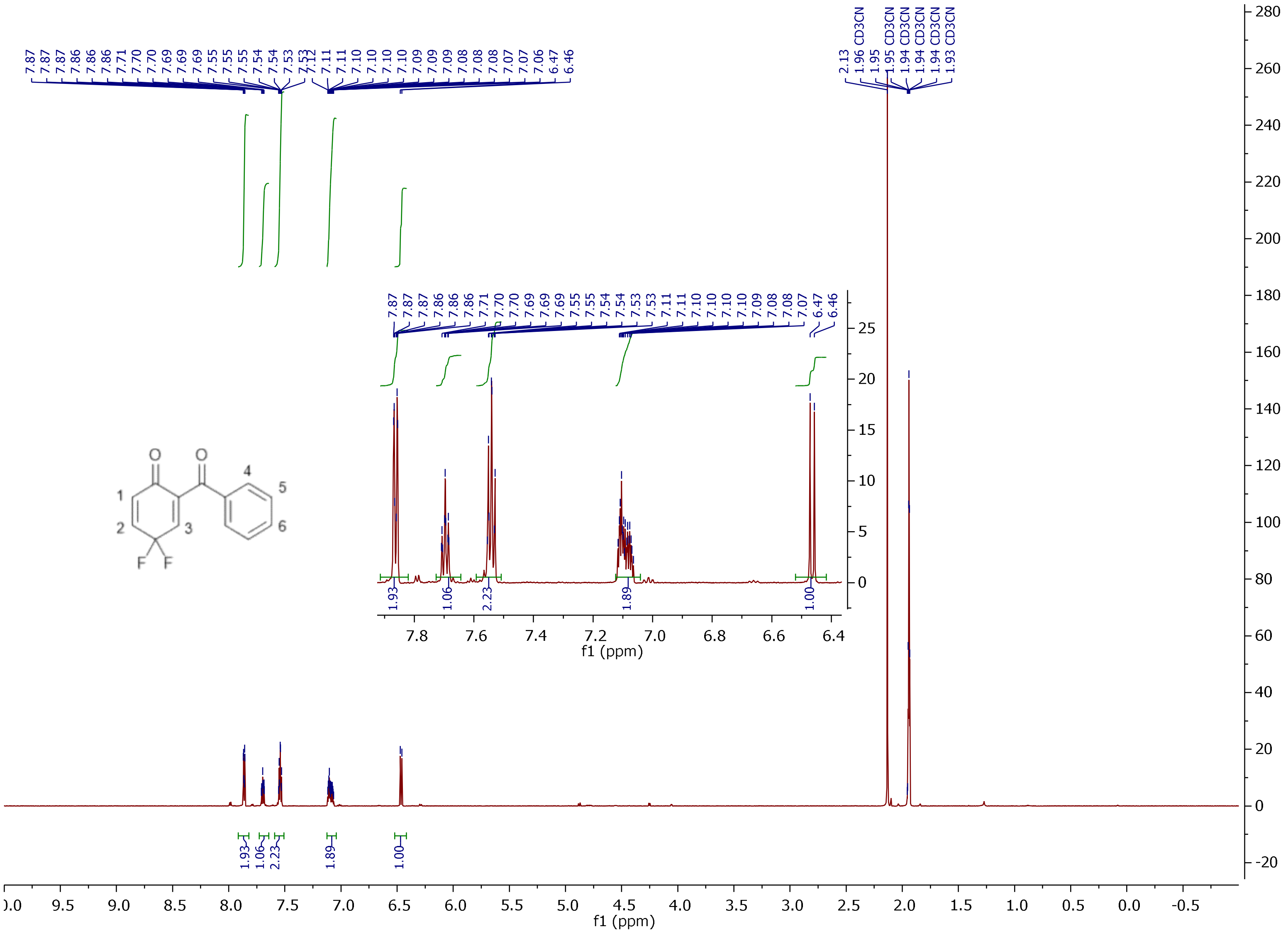

^{13}C NMR (176 MHz, CD_3CN) for fluorination of *N*-(2-benzoylphenyl)-2,2,2-trifluoroacetamide **(4t)**

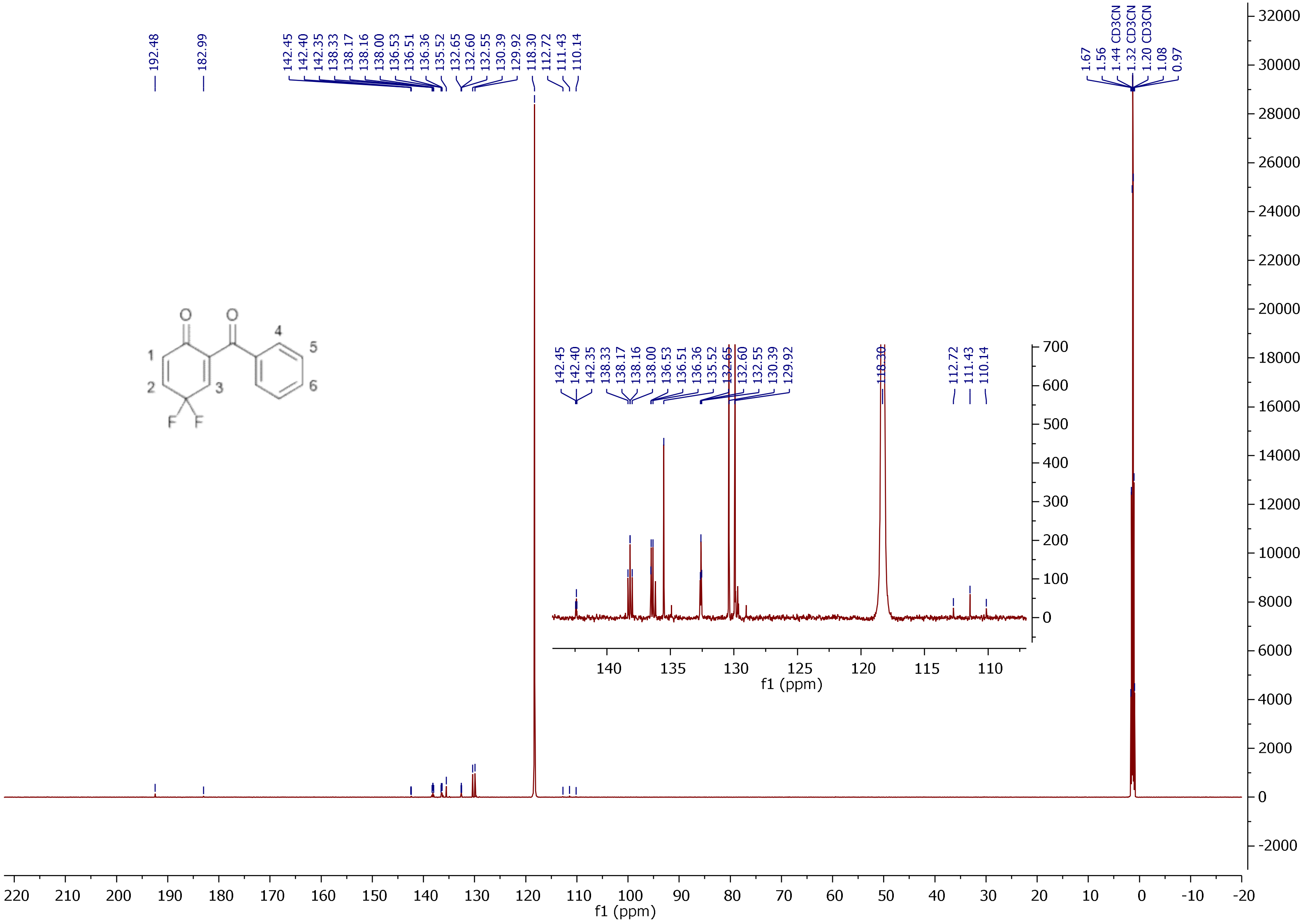

$^{19}F\{^{1}H\}$ NMR (376 MHz, CD_3CN) for fluorination of *N*-(2-benzoylphenyl)-2,2,2-trifluoroacetamide **(4t)**

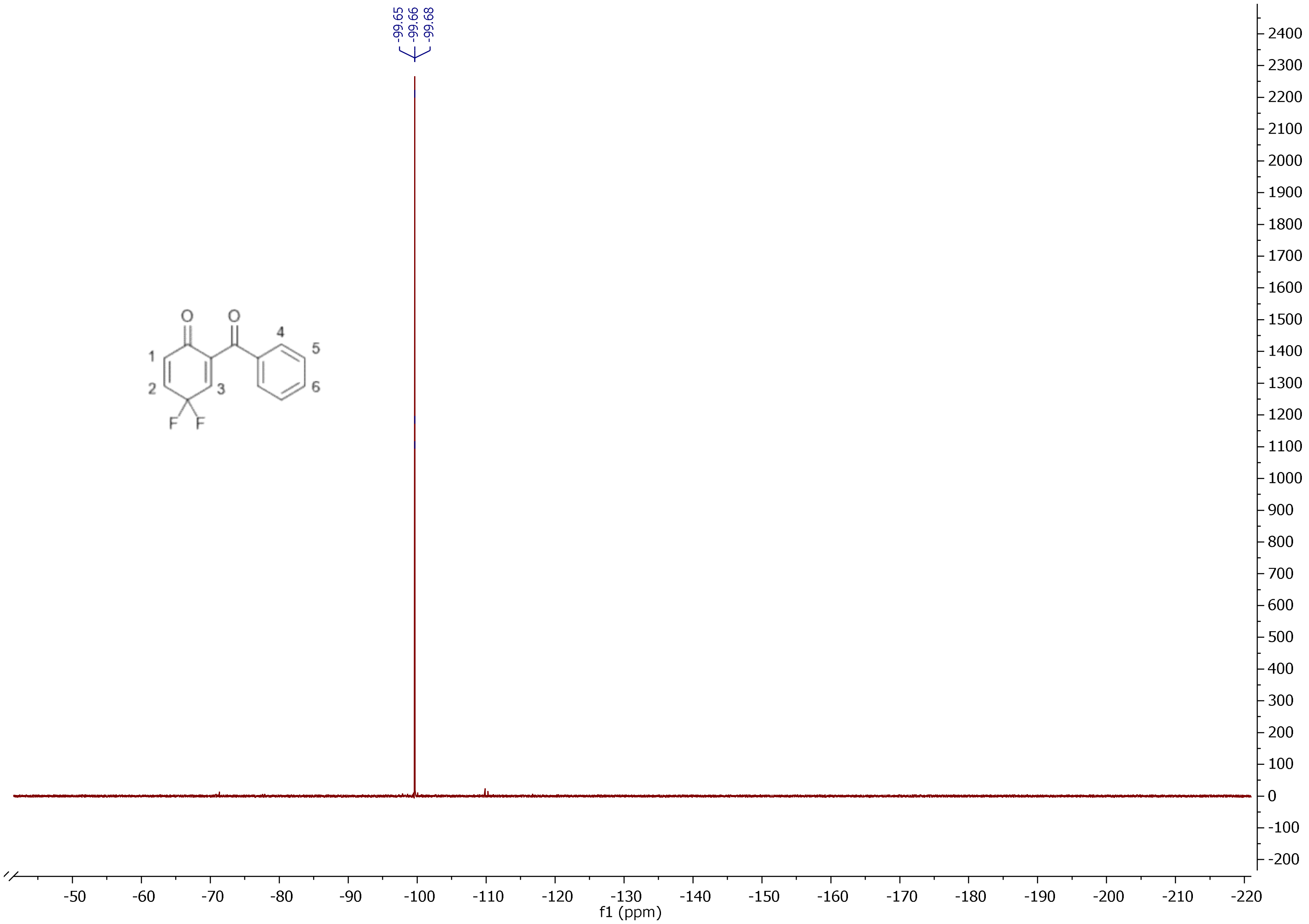

^{1}H NMR (700 MHz, CD_3CN) for fluorination of methyl 3-(2,2,2-trifluoroacetamido)benzoate **(3u)**

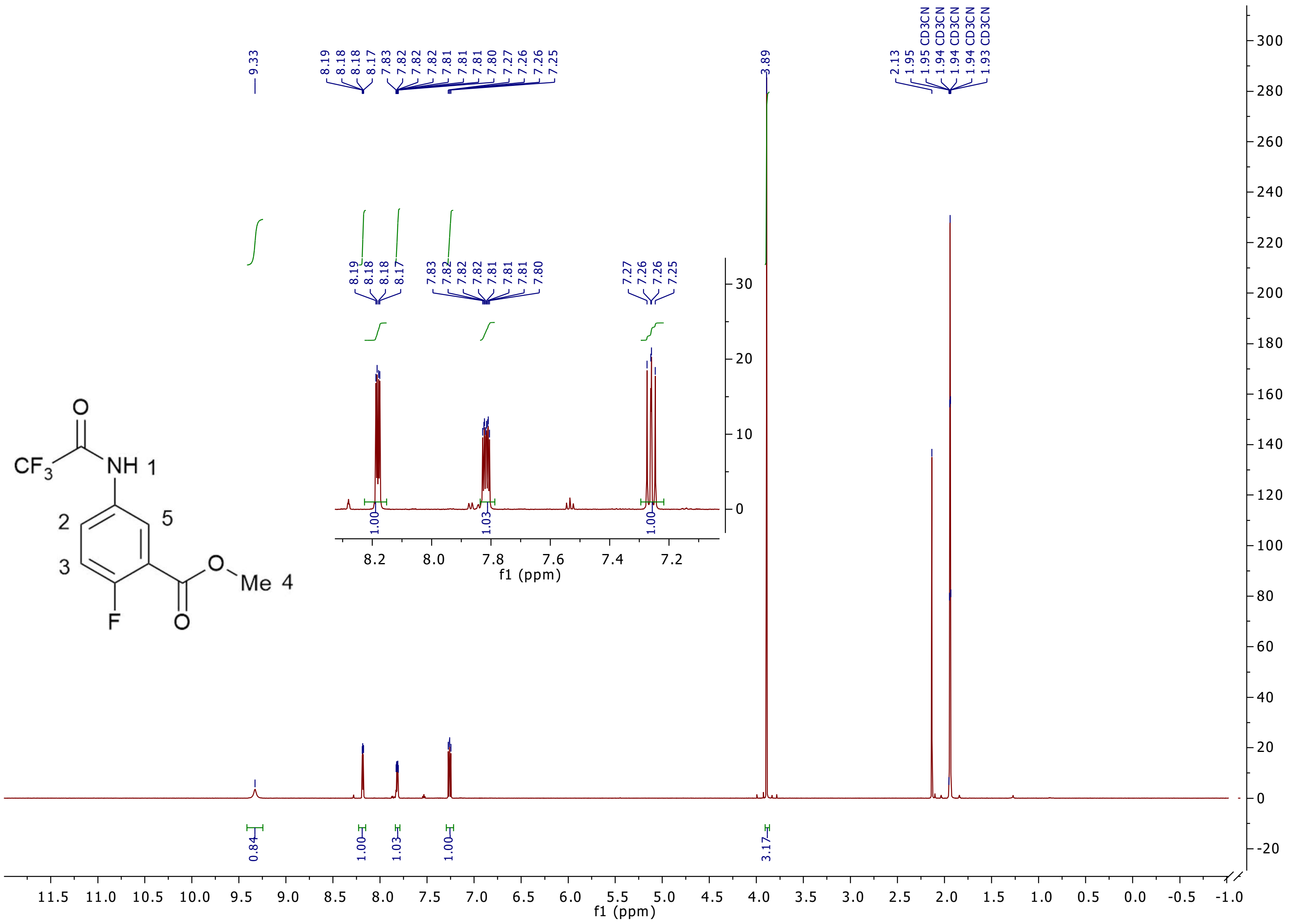

^{13}C NMR (176 MHz, CD_3CN) for fluorination of methyl 3-(2,2,2-trifluoroacetamido)benzoate **(3u)**

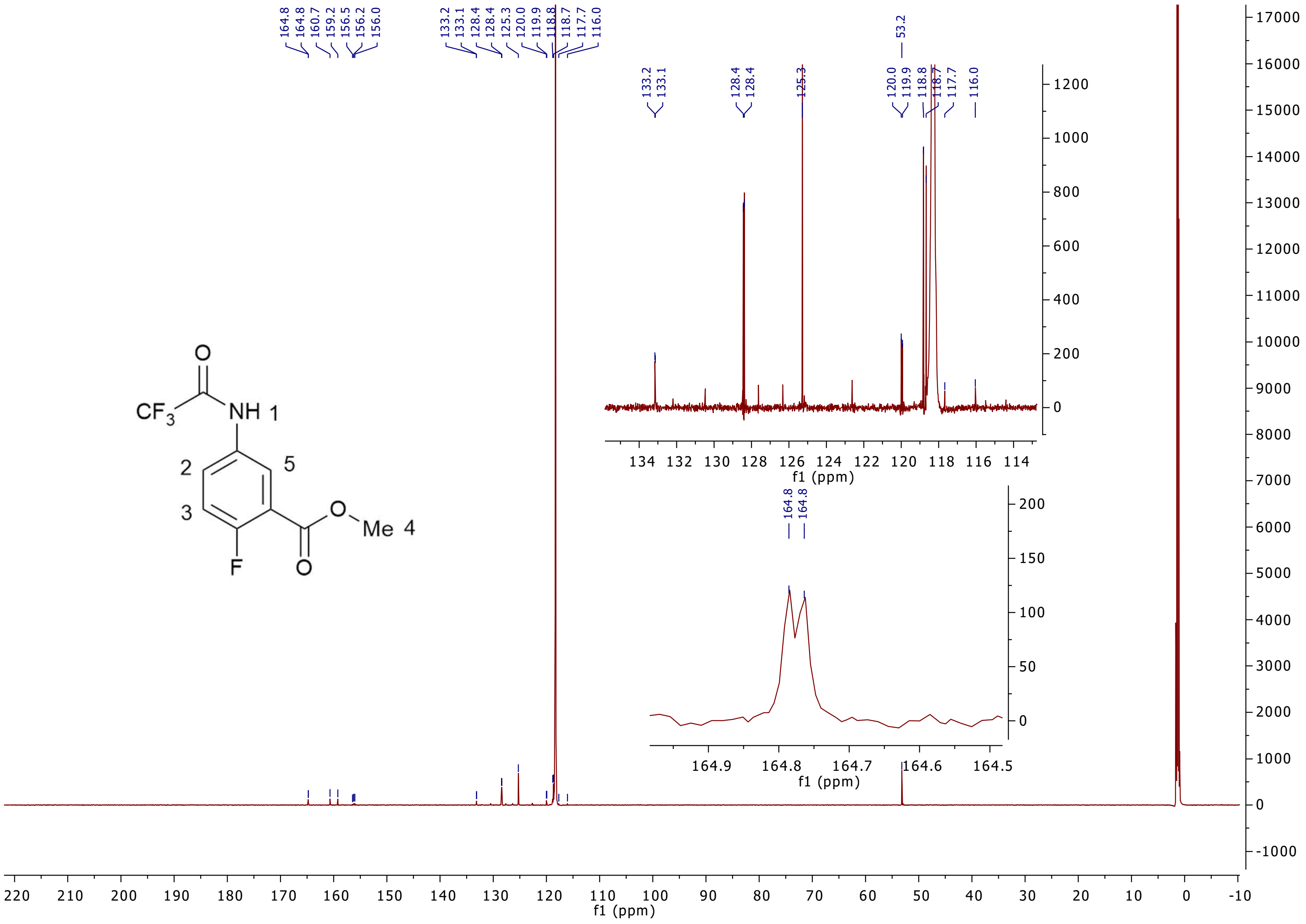

^{19}F{^{1}H} NMR (376 MHz, CD_3CN) for fluorination of methyl 3-(2,2,2-trifluoroacetamido)benzoate **(3u)**

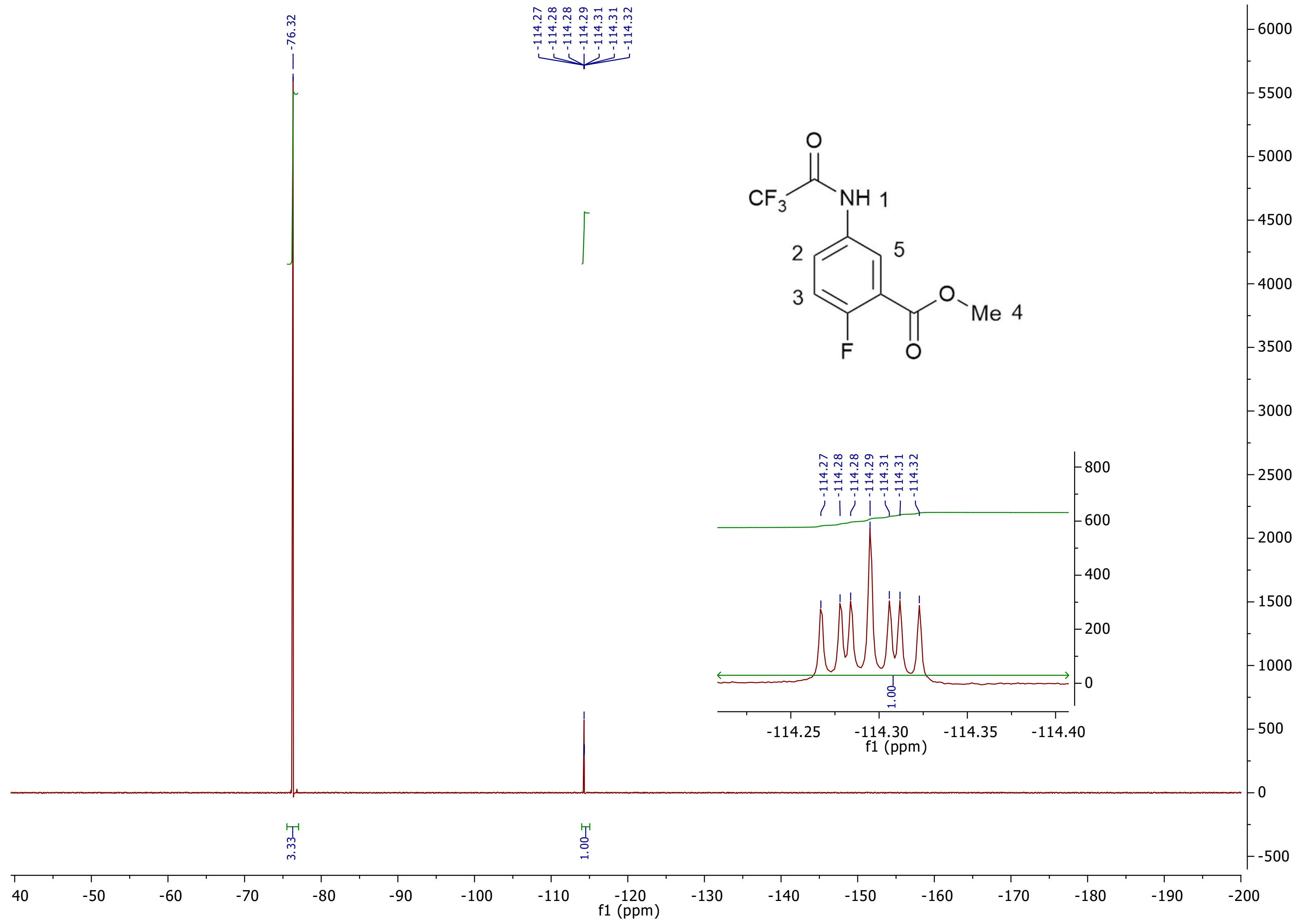

[1]**H NMR** (700 MHz, CD_3CN) for fluorination of *N*-(3-cyanophenyl)-2,2,2-trifluoroacetamide **(3v)**

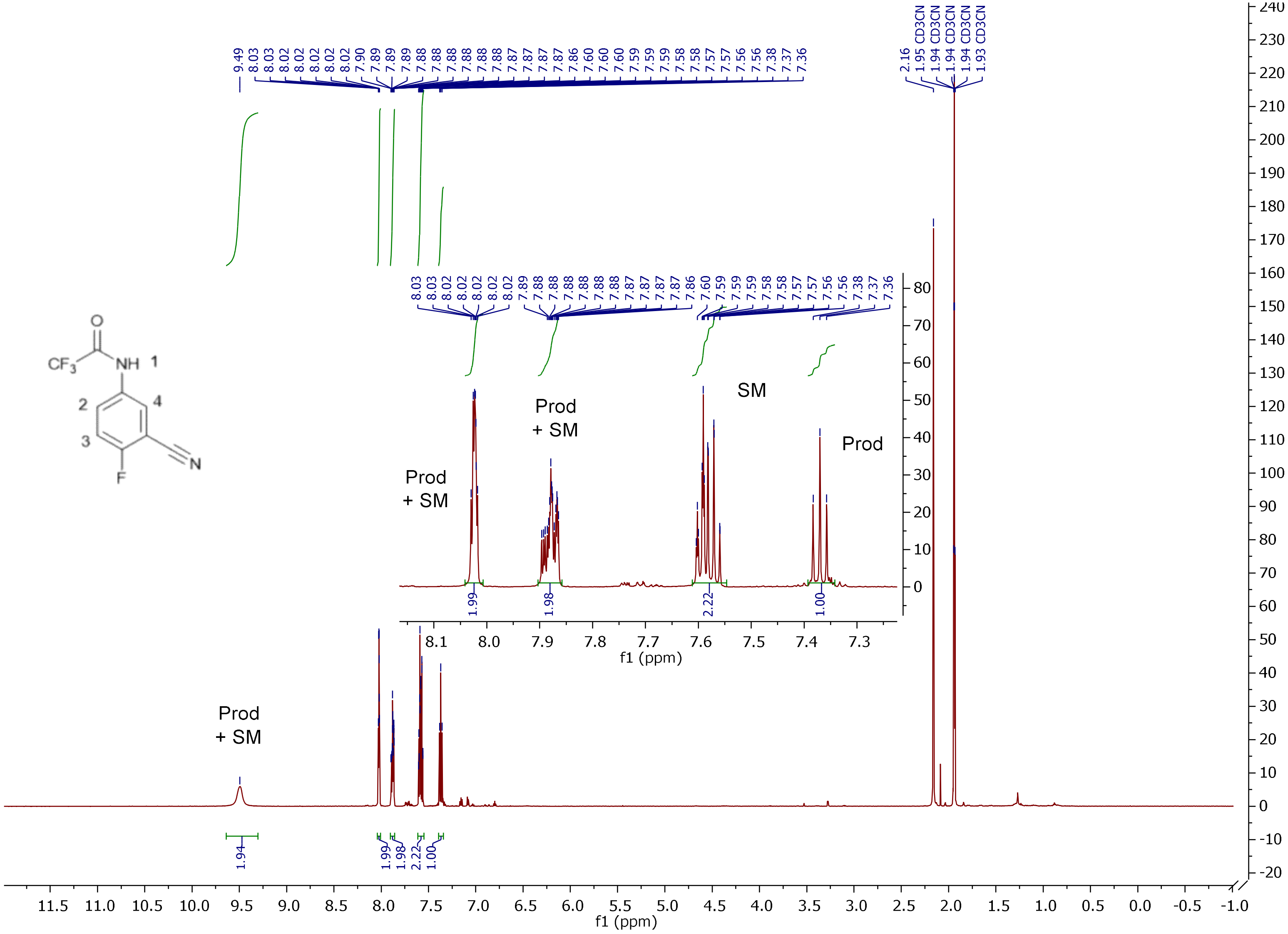

^{13}C NMR (176 MHz, CD_3CN) for fluorination of *N*-(3-cyanophenyl)-2,2,2-trifluoroacetamide **(3v)**

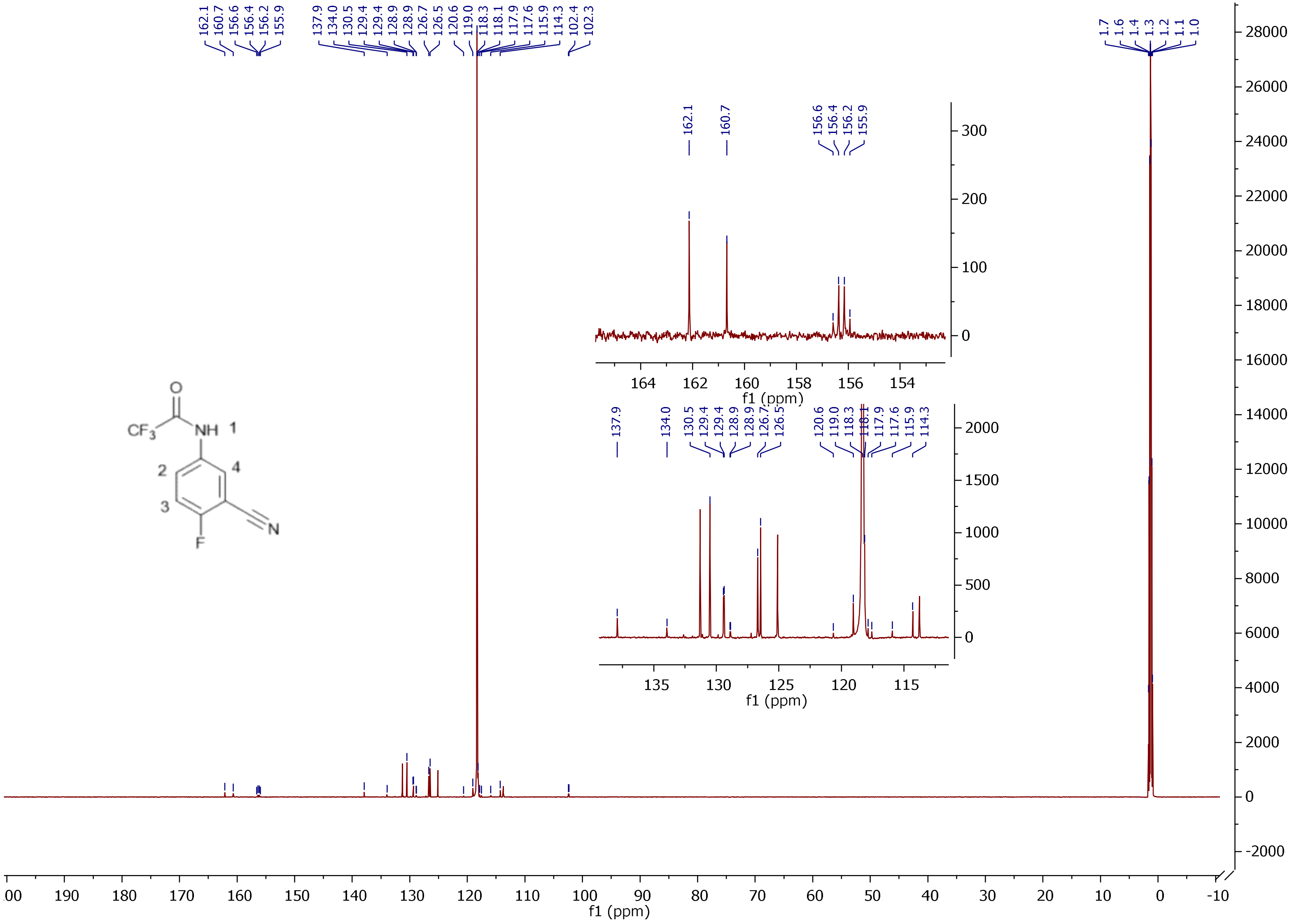

$^{19}F\{^1H\}$ NMR (376 MHz, CD_3CN) for fluorination of *N*-(3-cyanophenyl)-2,2,2-trifluoroacetamide **(3v)**

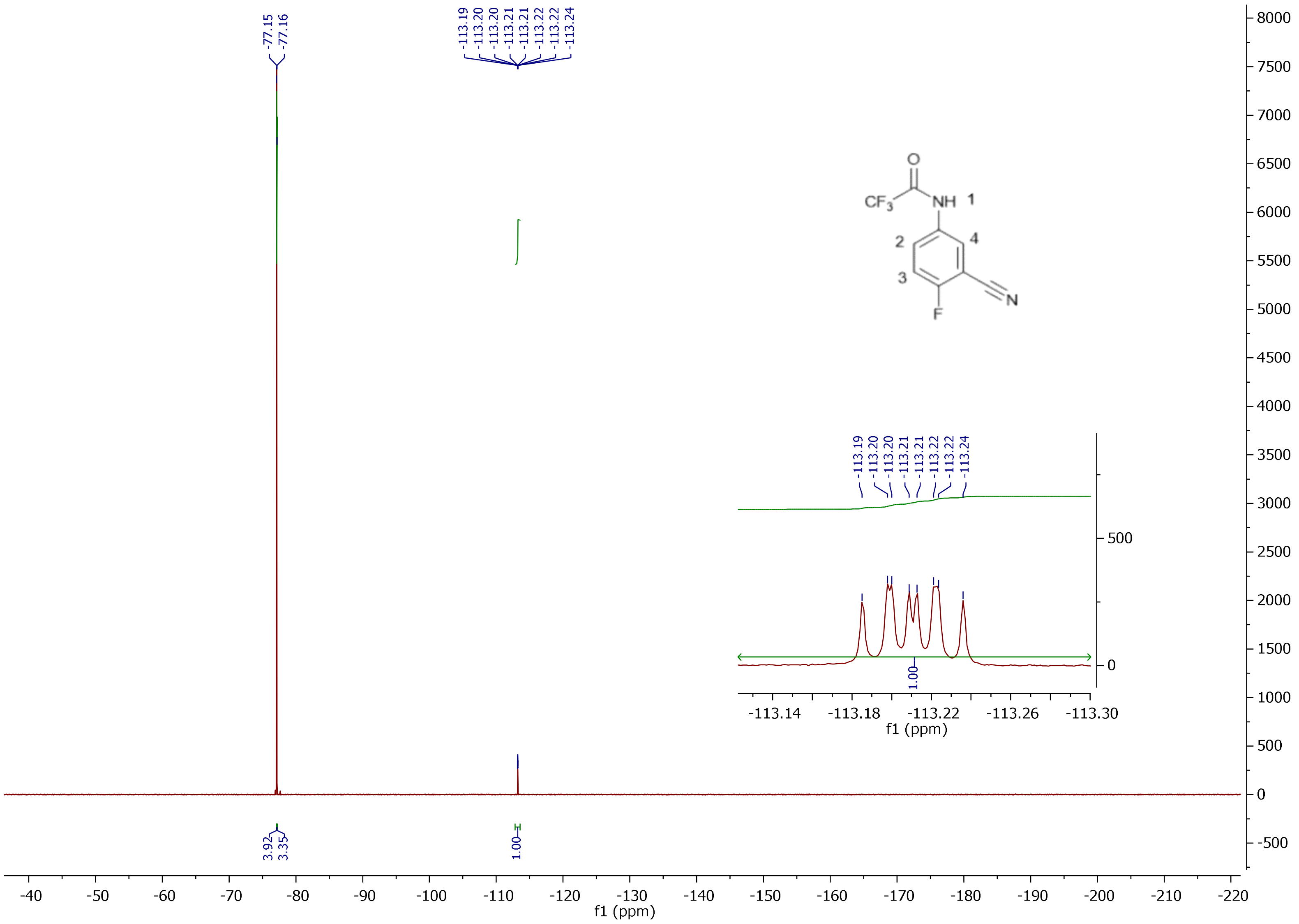

HMBC NMR (CD_3CN) for fluorination of *N*-(3-cyanophenyl)-2,2,2-trifluoroacetamide **(3v)**

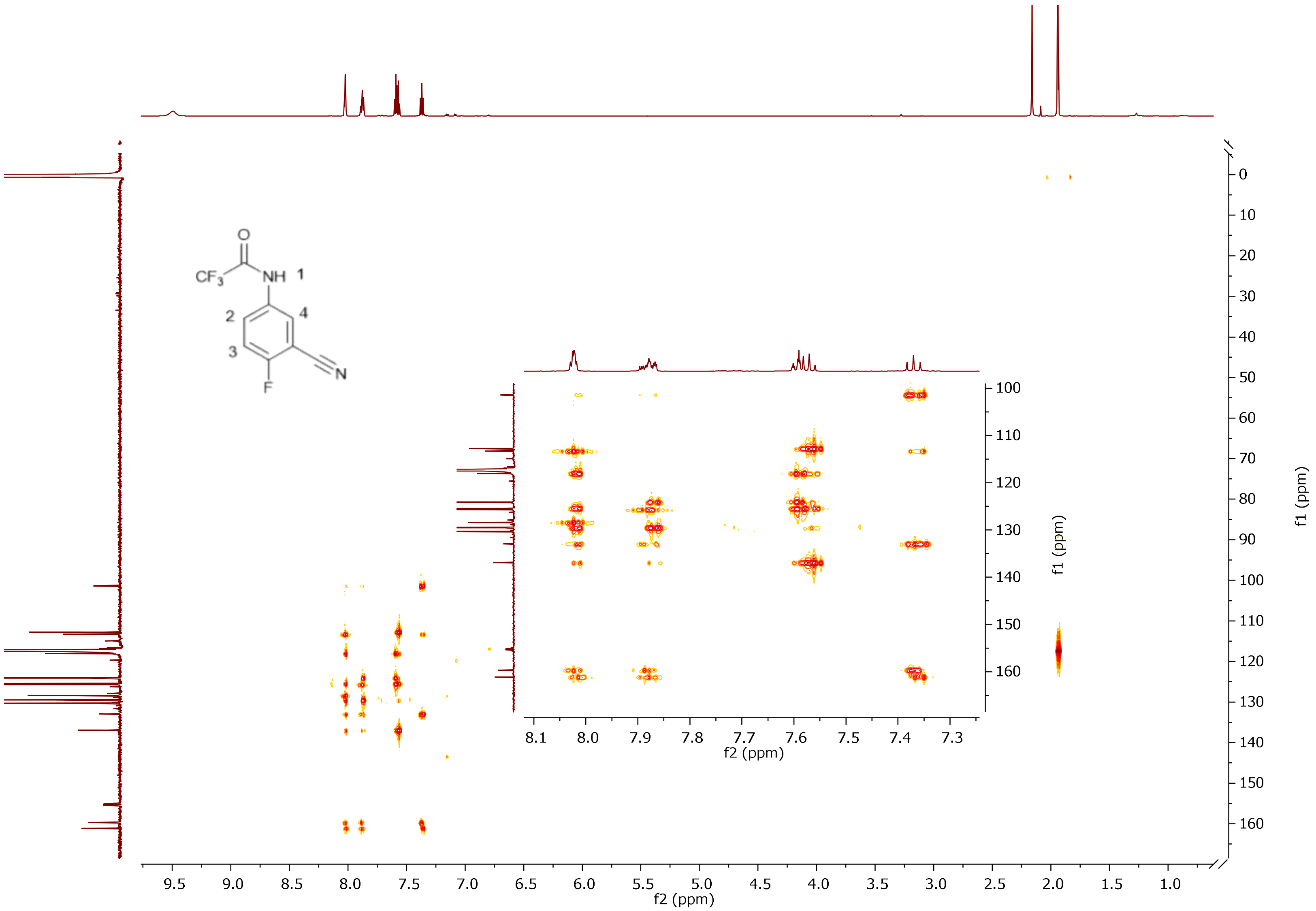

^{1}H NMR (700 MHz, CD_3CN) for fluorination of 2,2,2-trifluoro-*N*-(3-(4,4,5,5-tetramethyl-1,3,2-dioxaborolan-2-yl)phenyl)acetamide **(3w)**

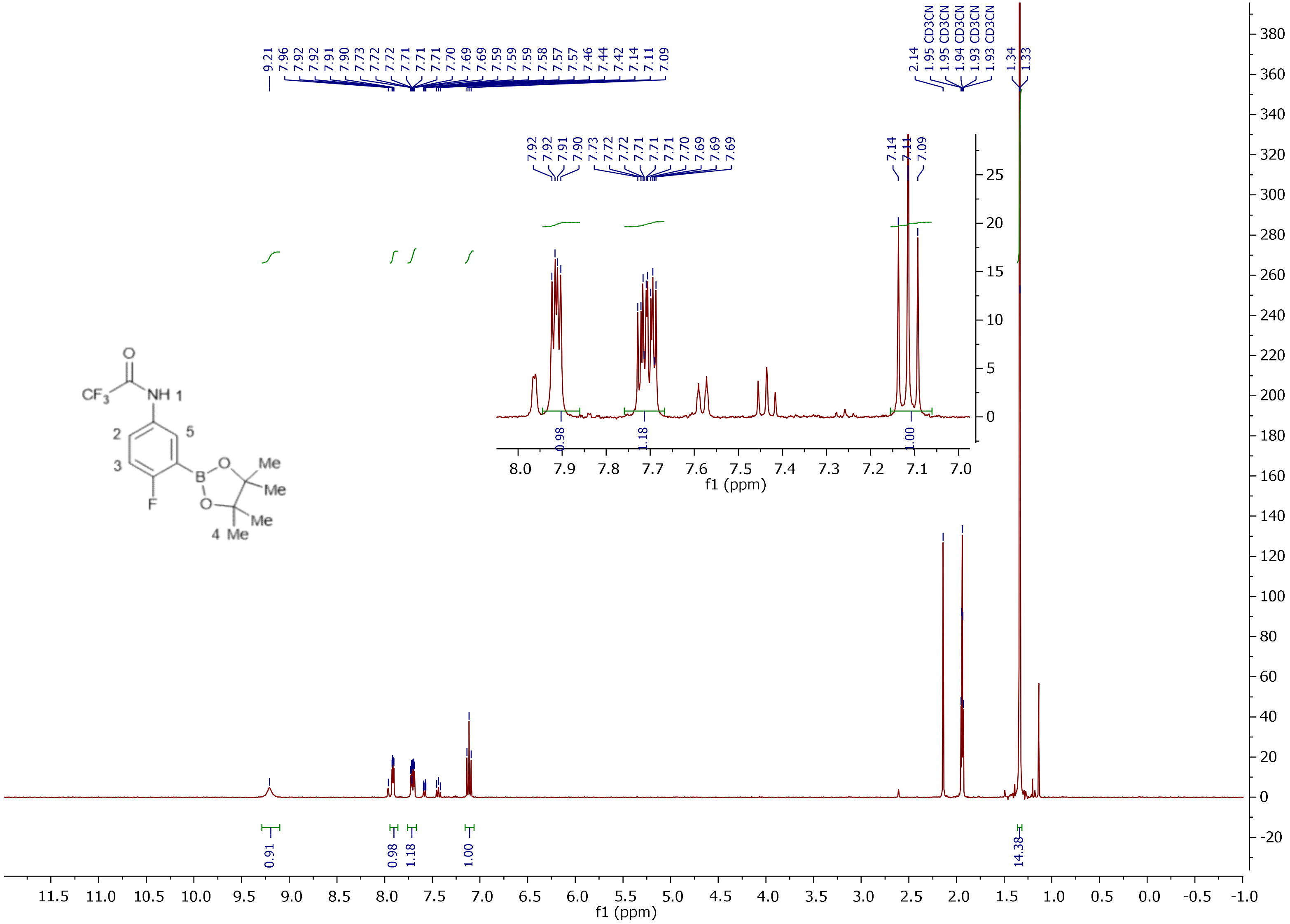

^{13}C NMR (176 MHz, CD_3CN) for fluorination of 2,2,2-trifluoro-*N*-(3-(4,4,5,5-tetramethyl-1,3,2-dioxaborolan-2-yl)phenyl)acetamide **(3w)**

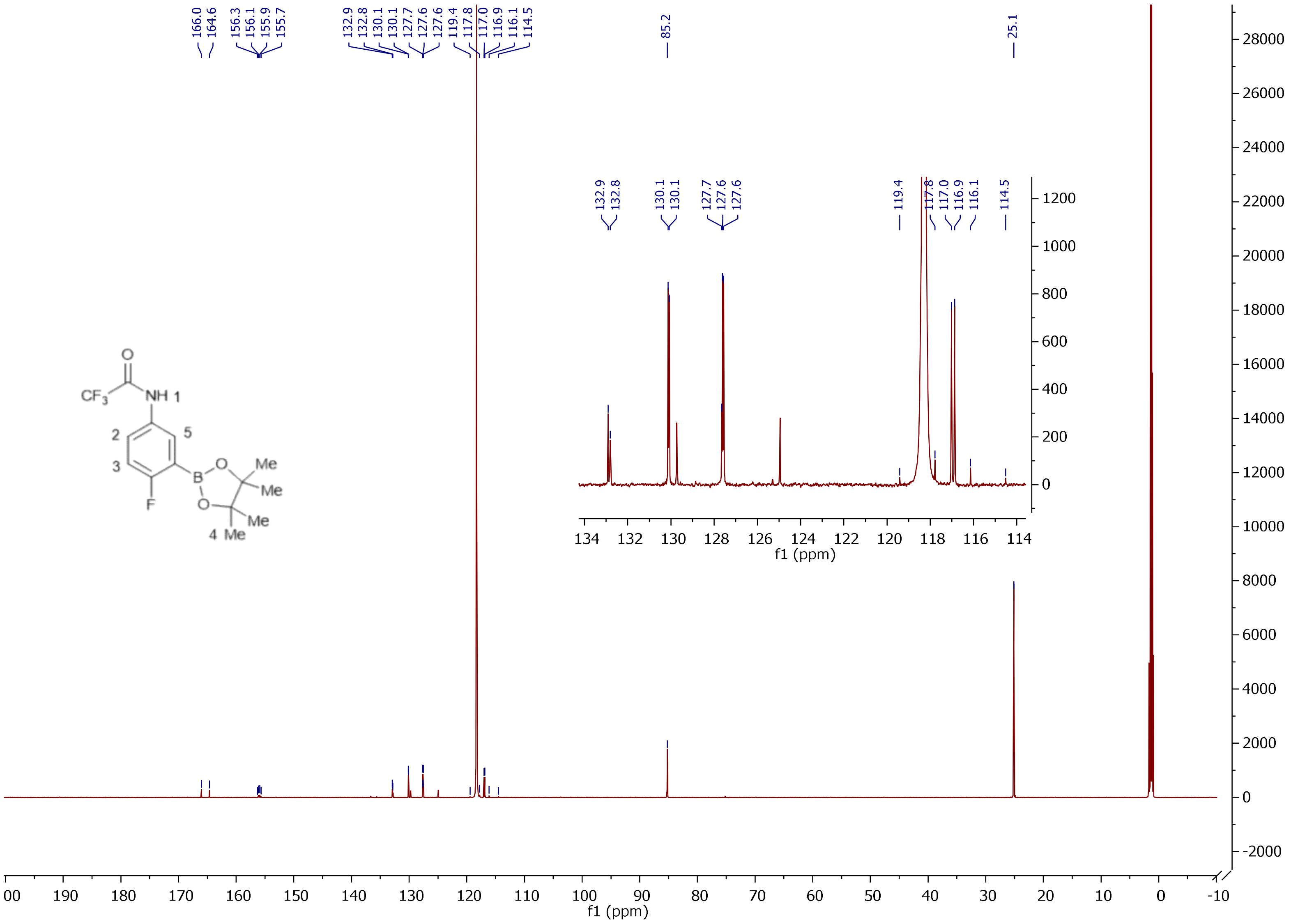

^{19}F{^{1}H} **NMR** (376 MHz, CD_3CN) for fluorination of 2,2,2-trifluoro-*N*-(3-(4,4,5,5-tetramethyl-1,3,2-dioxaborolan-2-yl)phenyl)acetamide **(3w)**

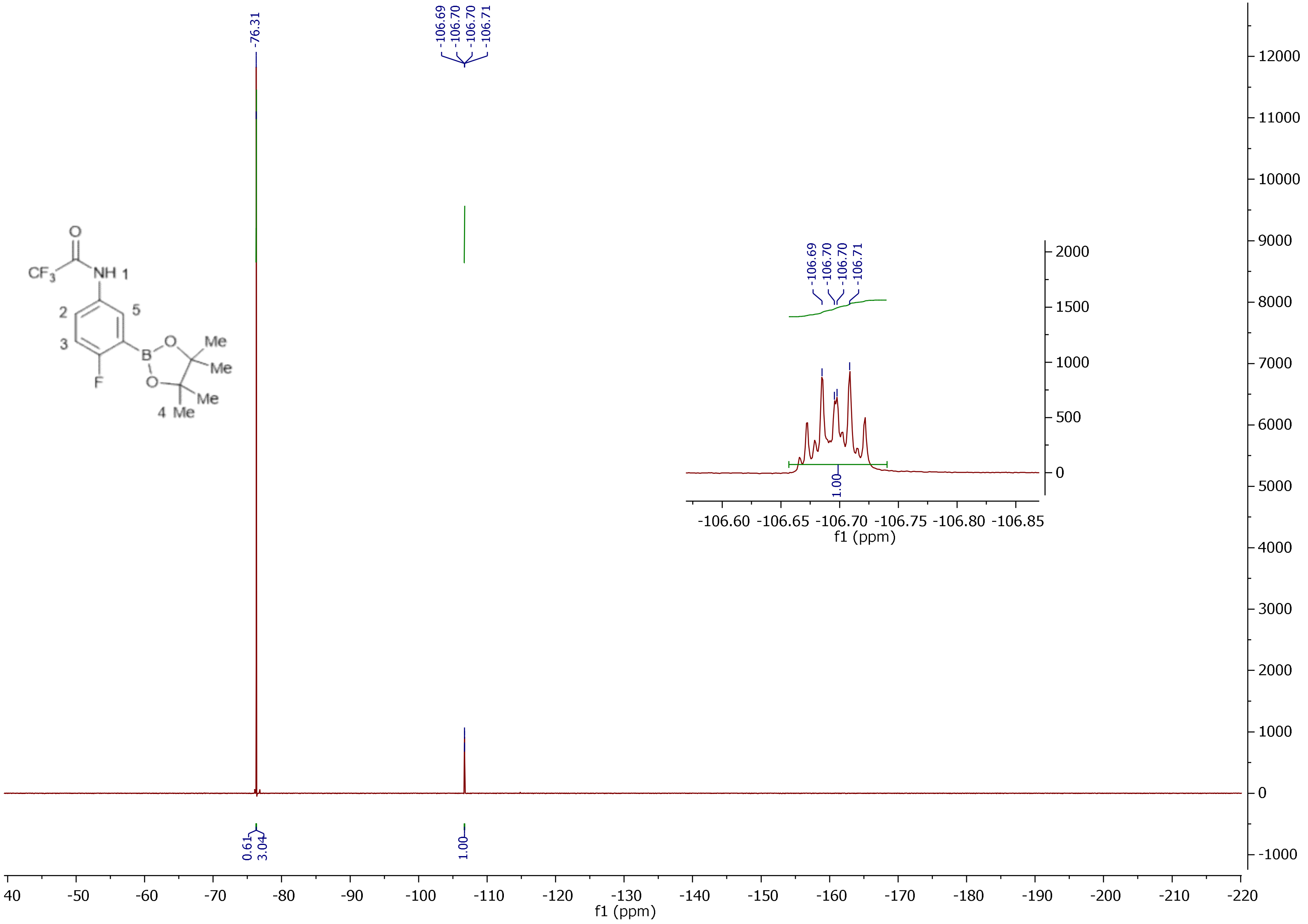

^{11}B NMR (160 MHz, CD_3CN) for fluorination of 2,2,2-trifluoro-*N*-(3-(4,4,5,5-tetramethyl-1,3,2-dioxaborolan-2-yl)phenyl)acetamide **(3w)**

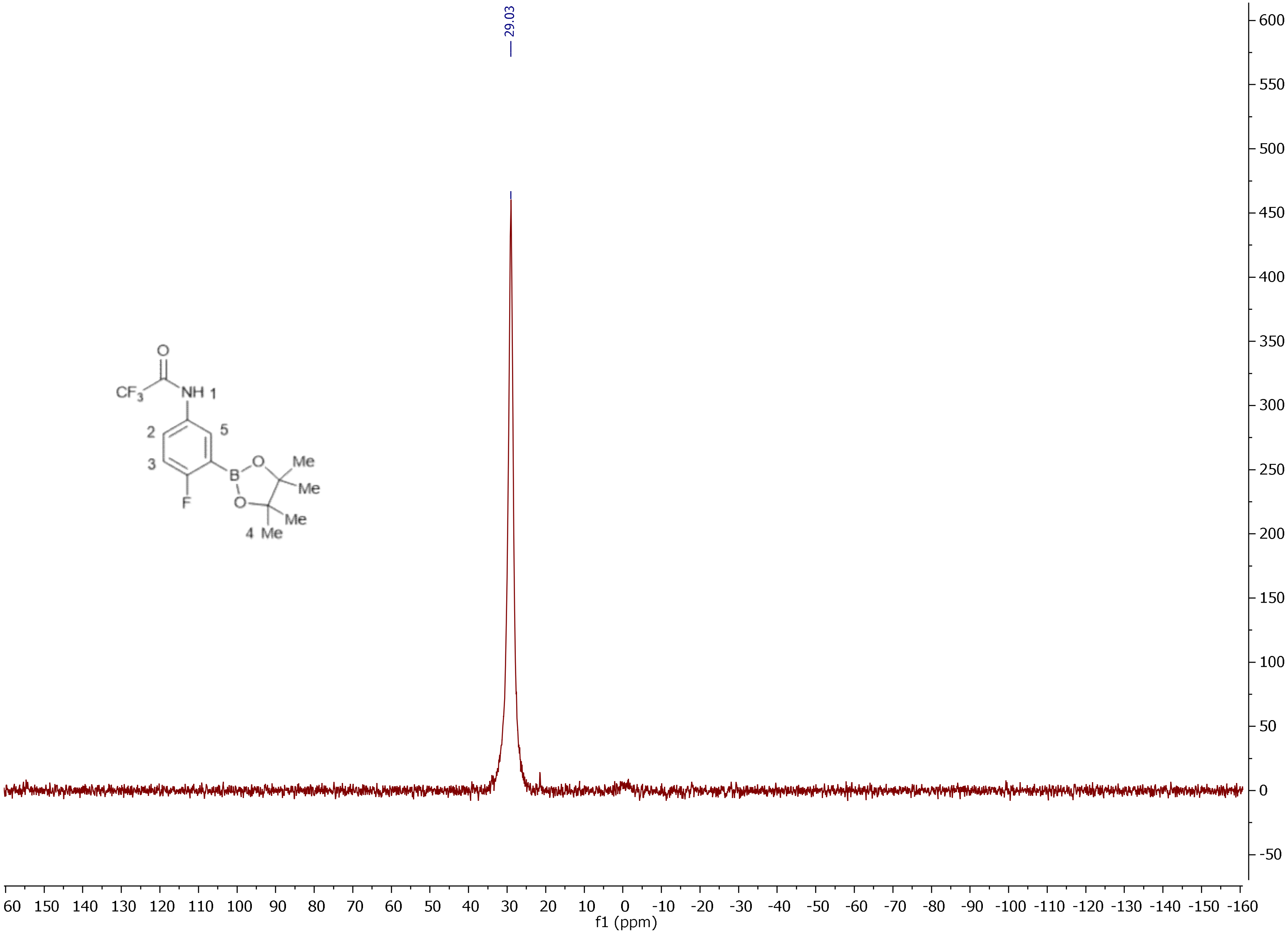

HMBC NMR (CD$_3$CN) for fluorination of 2,2,2-trifluoro-*N*-(3-(4,4,5,5-tetramethyl-1,3,2-dioxaborolan-2-yl)phenyl)acetamide **(3w)**

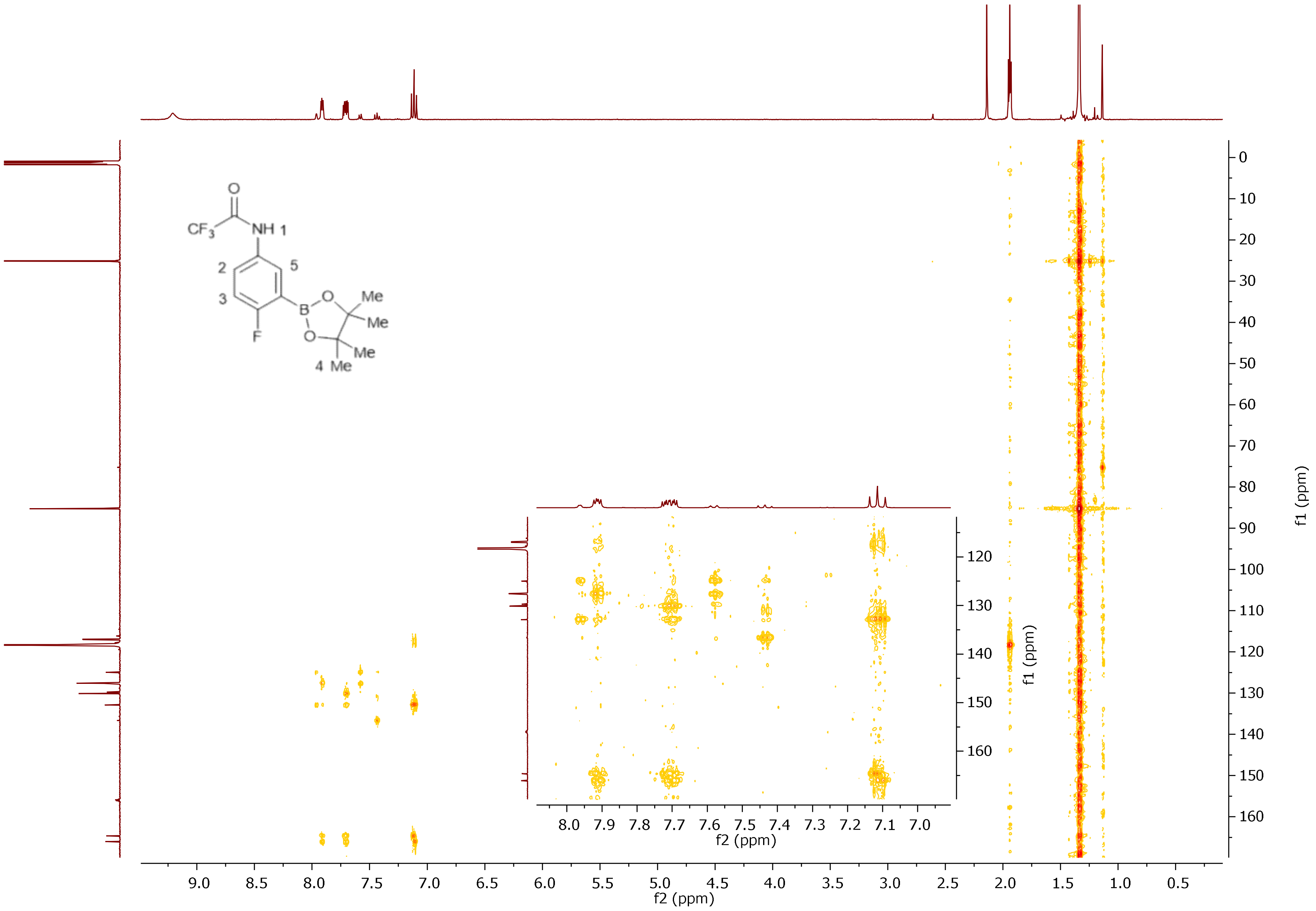

^{1}H NMR (700 MHz, CD_3CN) for fluorination of *N*-(2'-cyano-[1,1'-biphenyl]-3-yl)-2,2,2-trifluoroacetamide **(3x)**

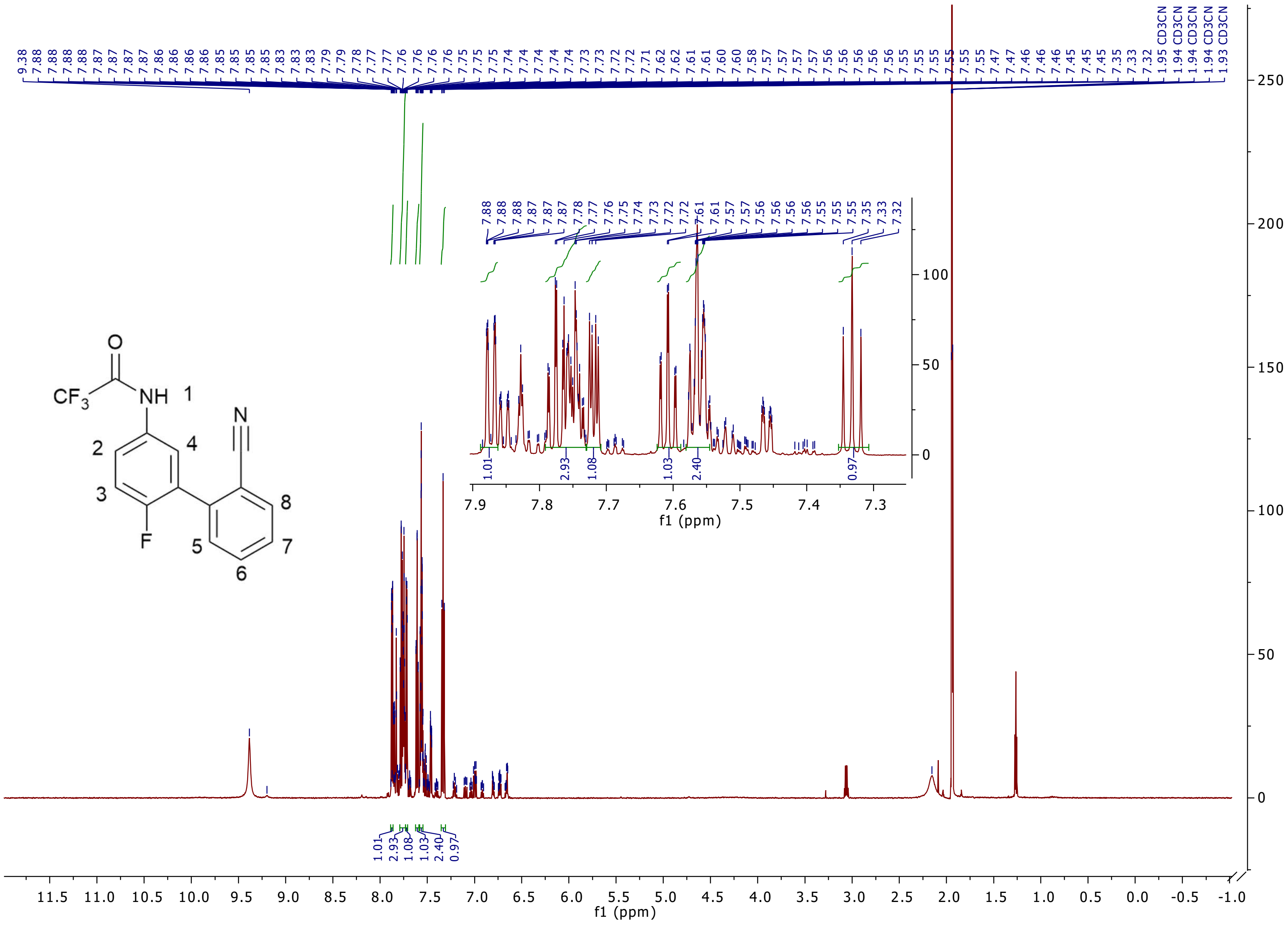

^{13}**C NMR** (176 MHz, CD_3CN) for fluorination of *N*-(2'-cyano-[1,1'-biphenyl]-3-yl)-2,2,2-trifluoroacetamide **(3x)**

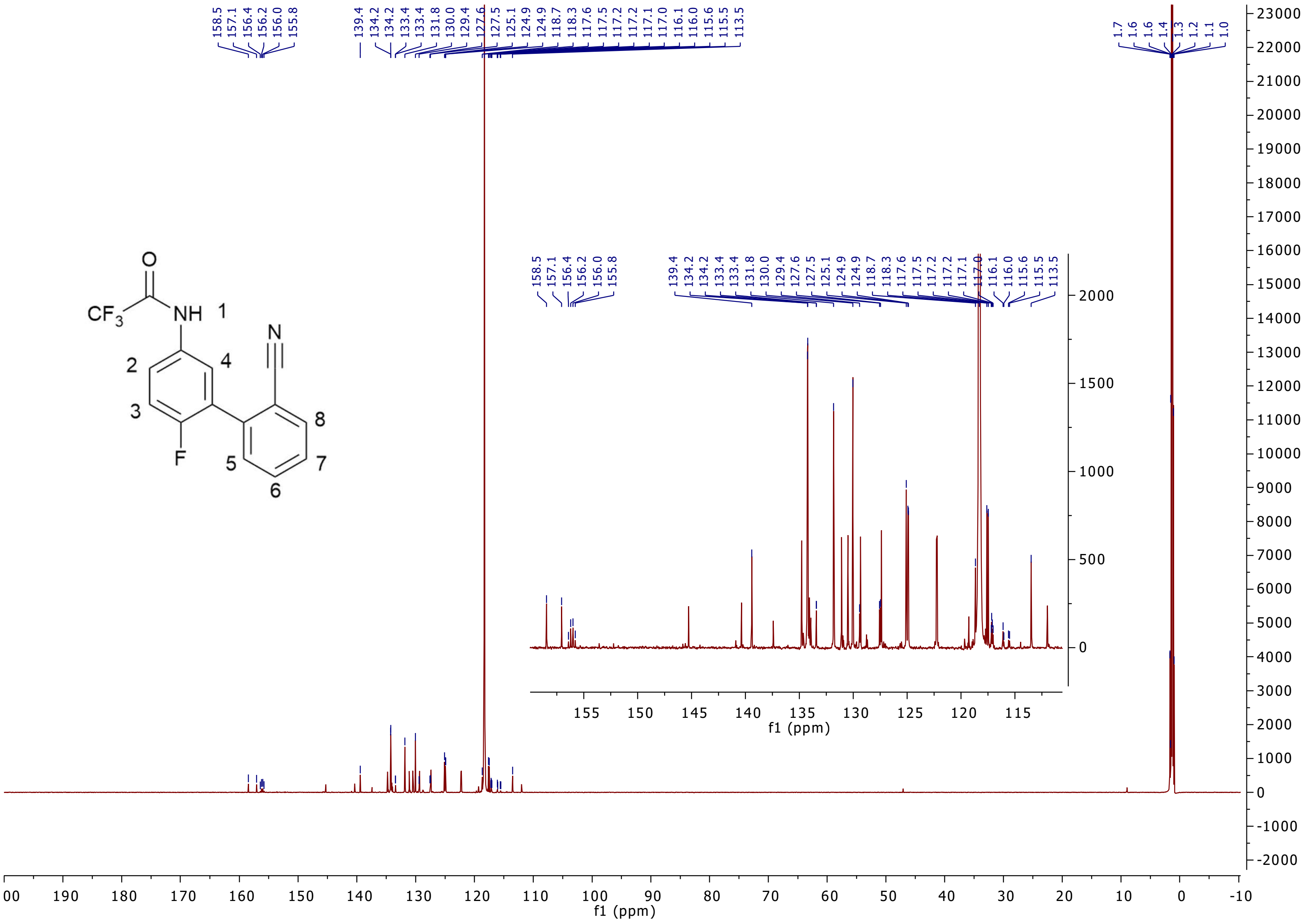

^{19}F{^{1}H} NMR (376 MHz, CD_3CN) for fluorination of *N*-(2'-cyano-[1,1'-biphenyl]-3-yl)-2,2,2-trifluoroacetamide **(3x)**

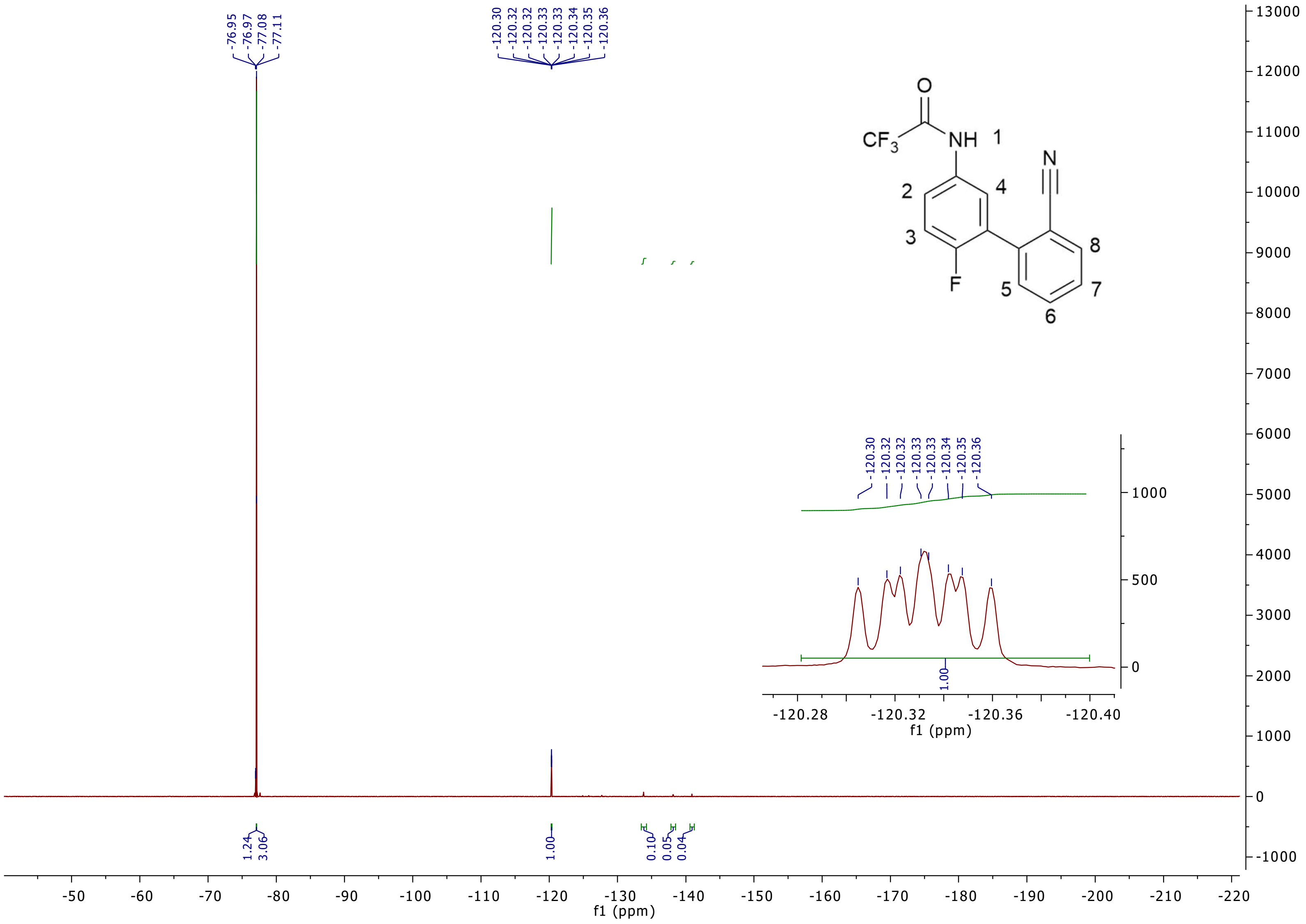

HMBC NMR (CD$_3$CN) for fluorination of *N*-(2'-cyano-[1,1'-biphenyl]-3-yl)-2,2,2-trifluoroacetamide **(3x)**

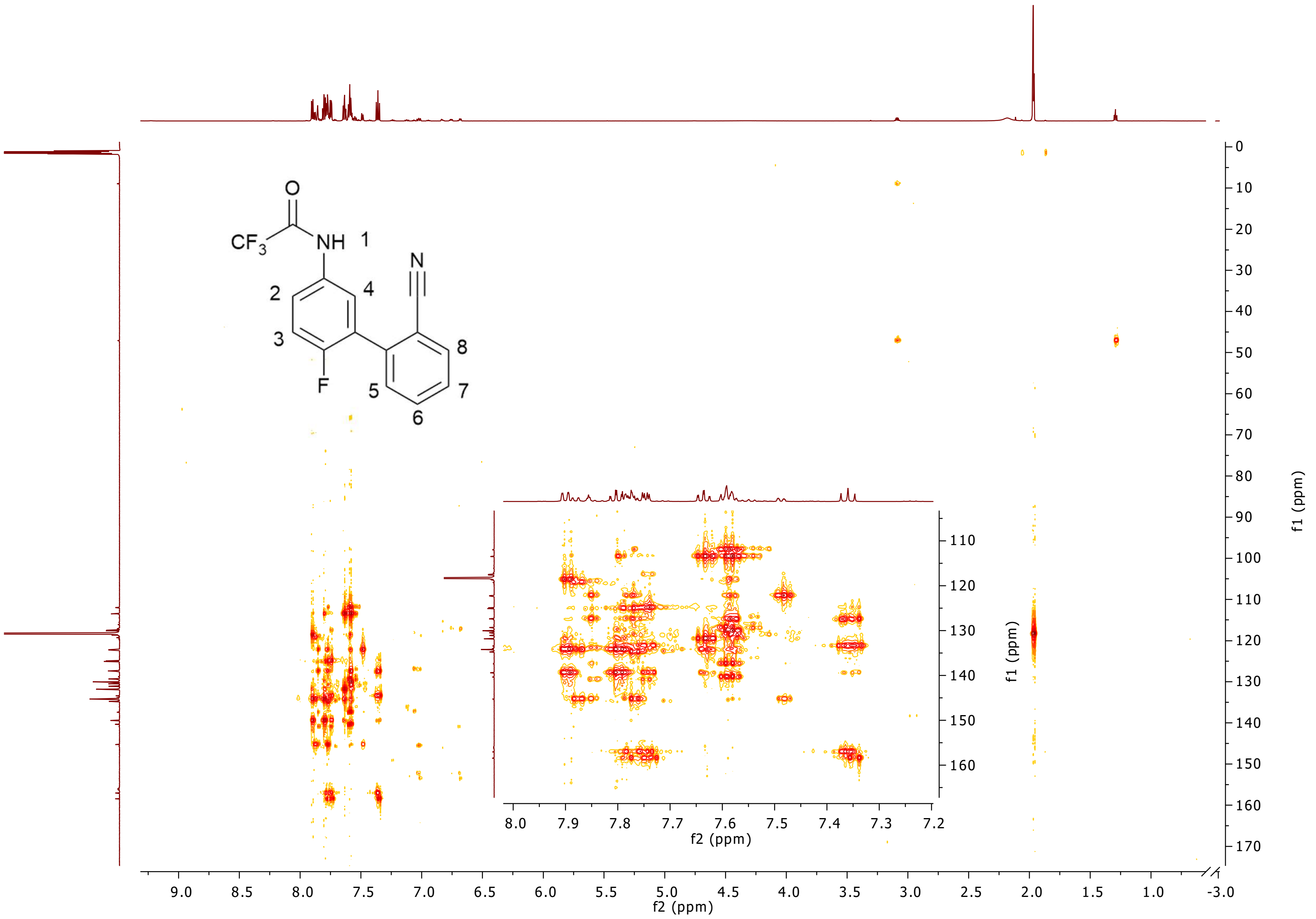

^{1}H NMR (700 MHz, CD_3CN) for fluorination of 2,2,2-trifluoro-*N*-(3-(pyrimidin-5-yl)phenyl)acetamide **(3y)**

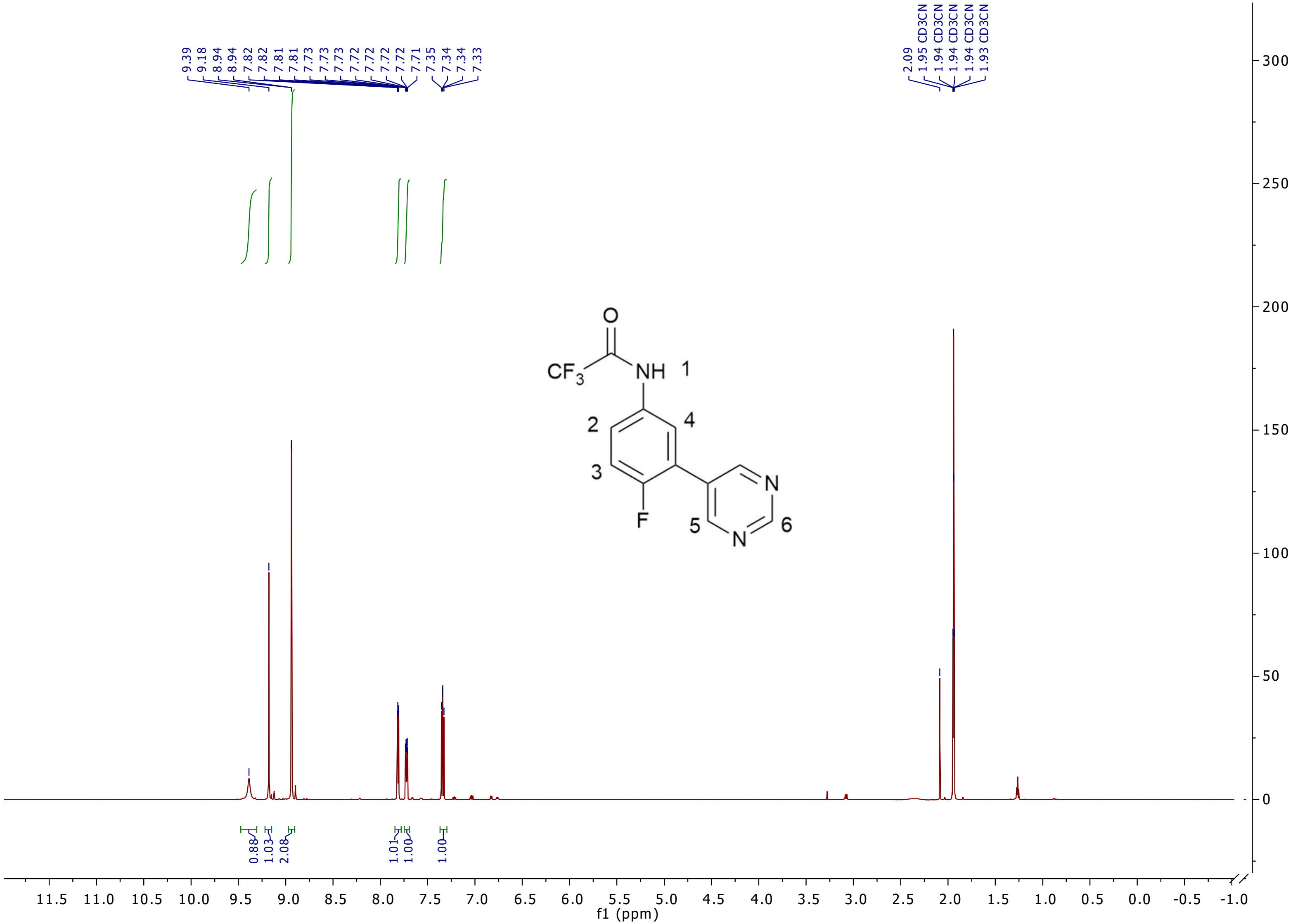

^{13}C NMR (176 MHz, CD_3CN) for fluorination of 2,2,2-trifluoro-*N*-(3-(pyrimidin-5-yl)phenyl)acetamide **(3y)**

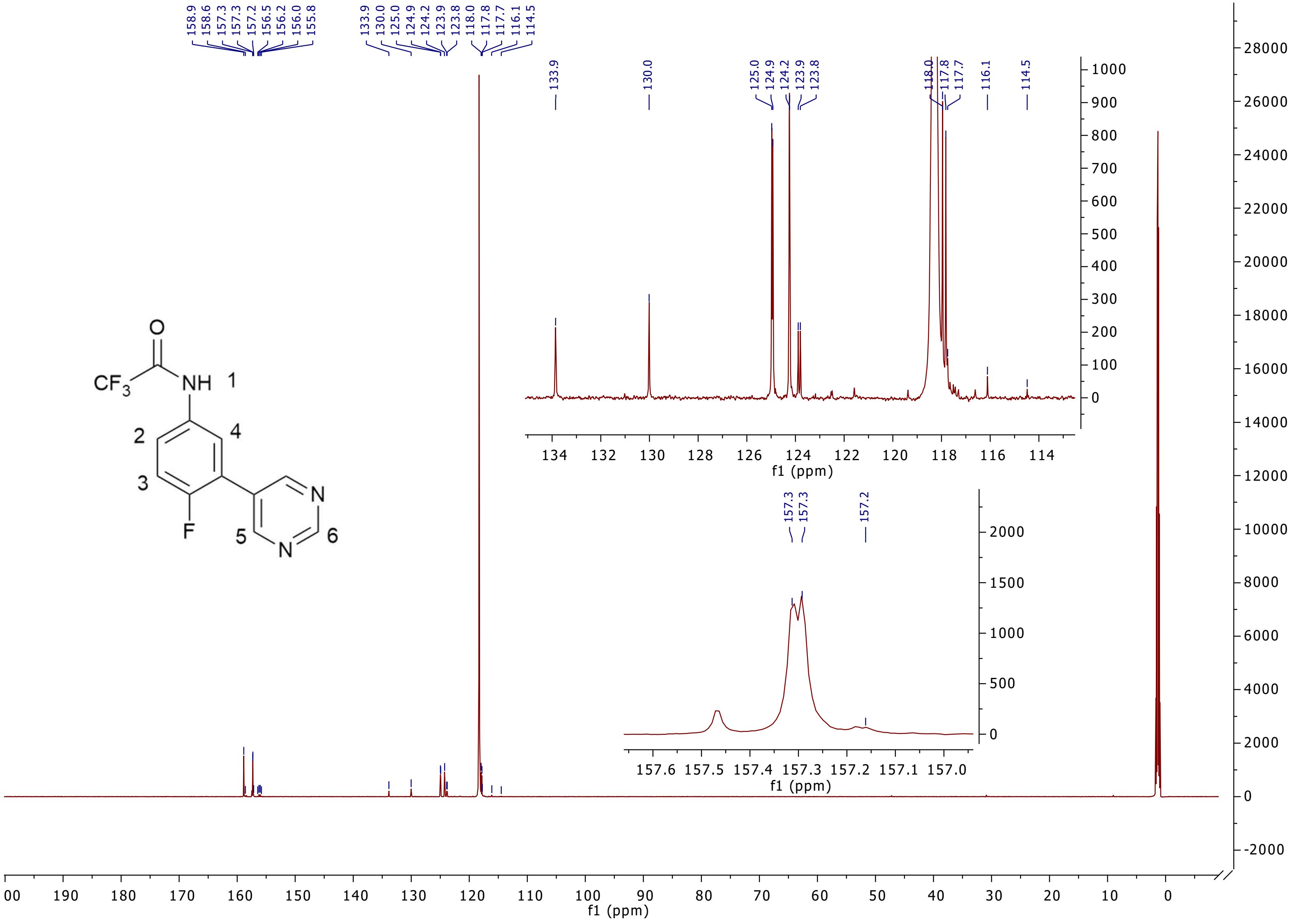

$^{19}F\{^{1}H\}$ NMR (376 MHz, CD_3CN) for fluorination of 2,2,2-trifluoro-*N*-(3-(pyrimidin-5-yl)phenyl)acetamide **(3y)**

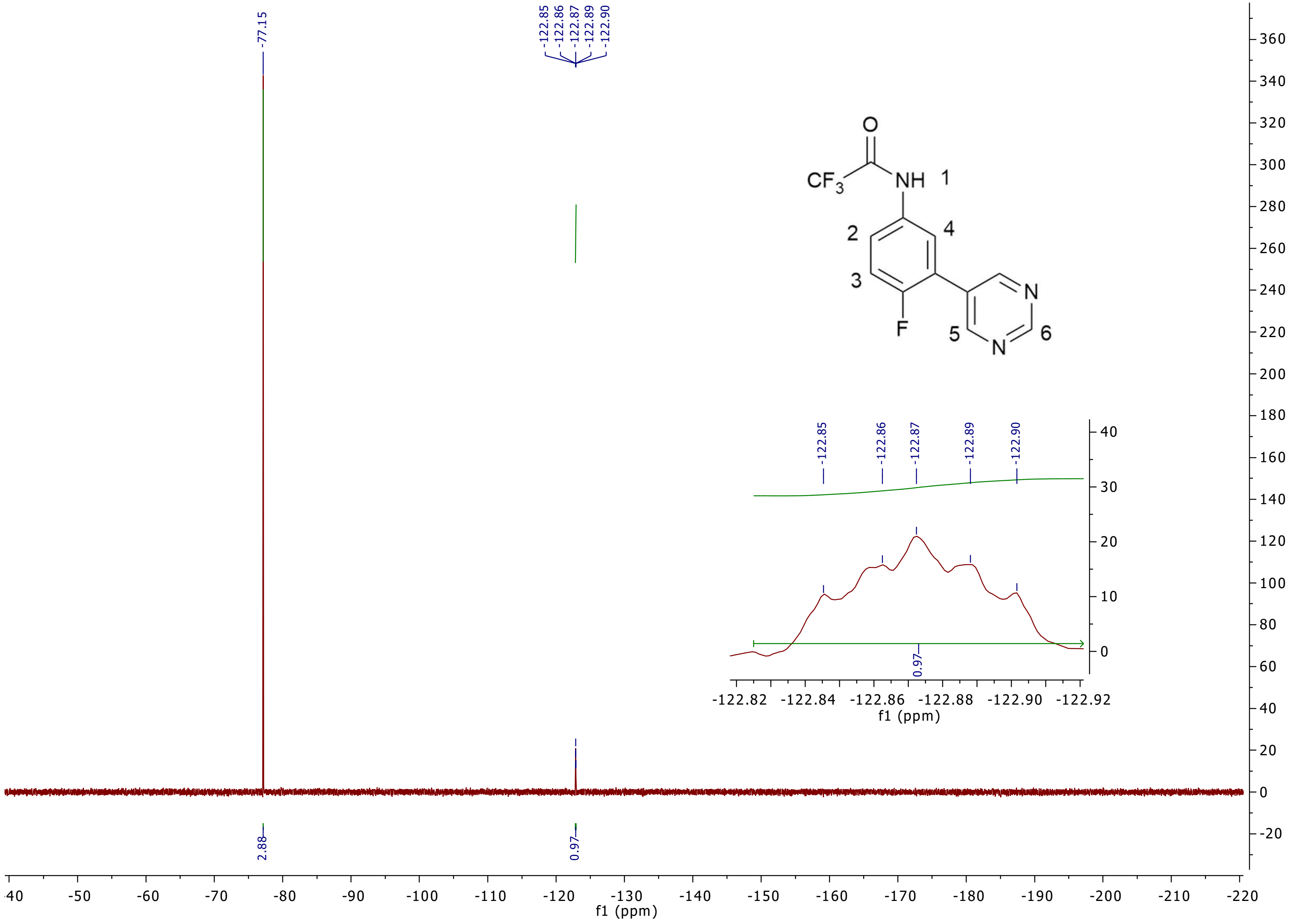

^{1}H NMR (700 MHz, CD_3CN) for fluorination of methyl 2-(4-isobutyl-3-(2,2,2-trifluoroacetamido)phenyl)propanoate **(3z)**

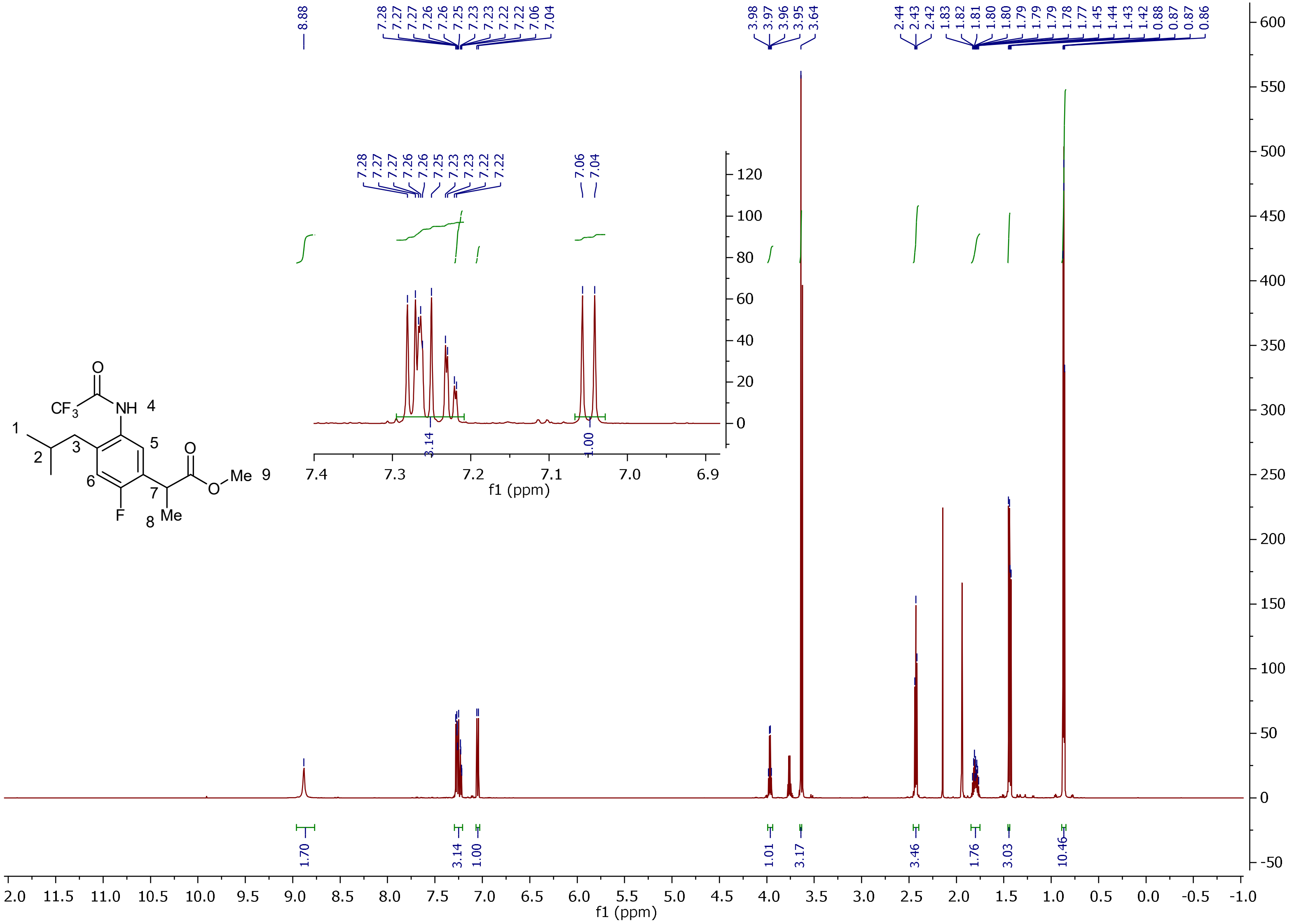

^{13}C NMR (176 MHz, CD_3CN) for fluorination of methyl 2-(4-isobutyl-3-(2,2,2-trifluoroacetamido)phenyl)propanoate **(3z)**

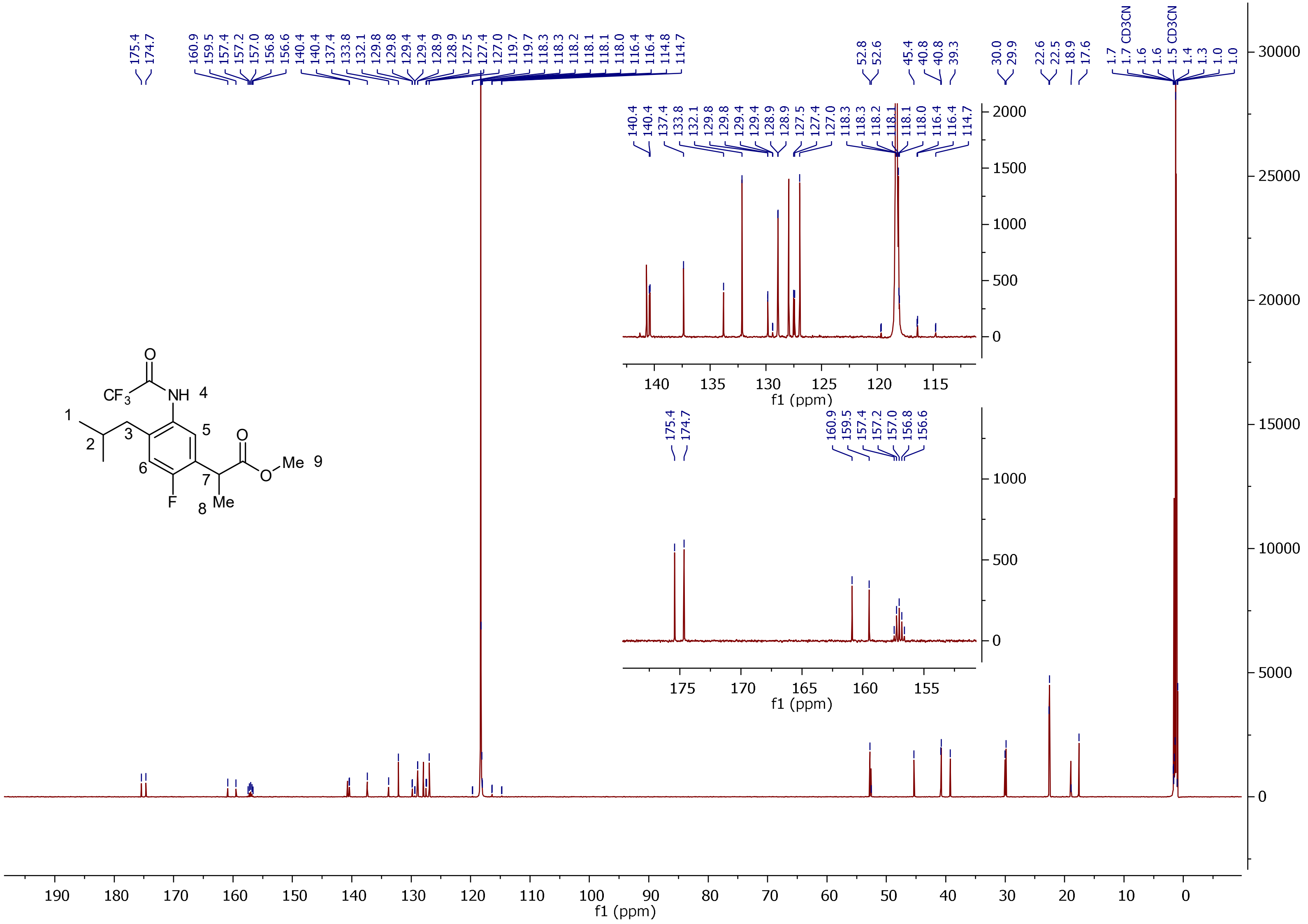

^{19}F{^{1}H} NMR (376 MHz, CD_3CN) for fluorination of methyl 2-(4-isobutyl-3-(2,2,2-trifluoroacetamido)phenyl)propanoate **(3z)**

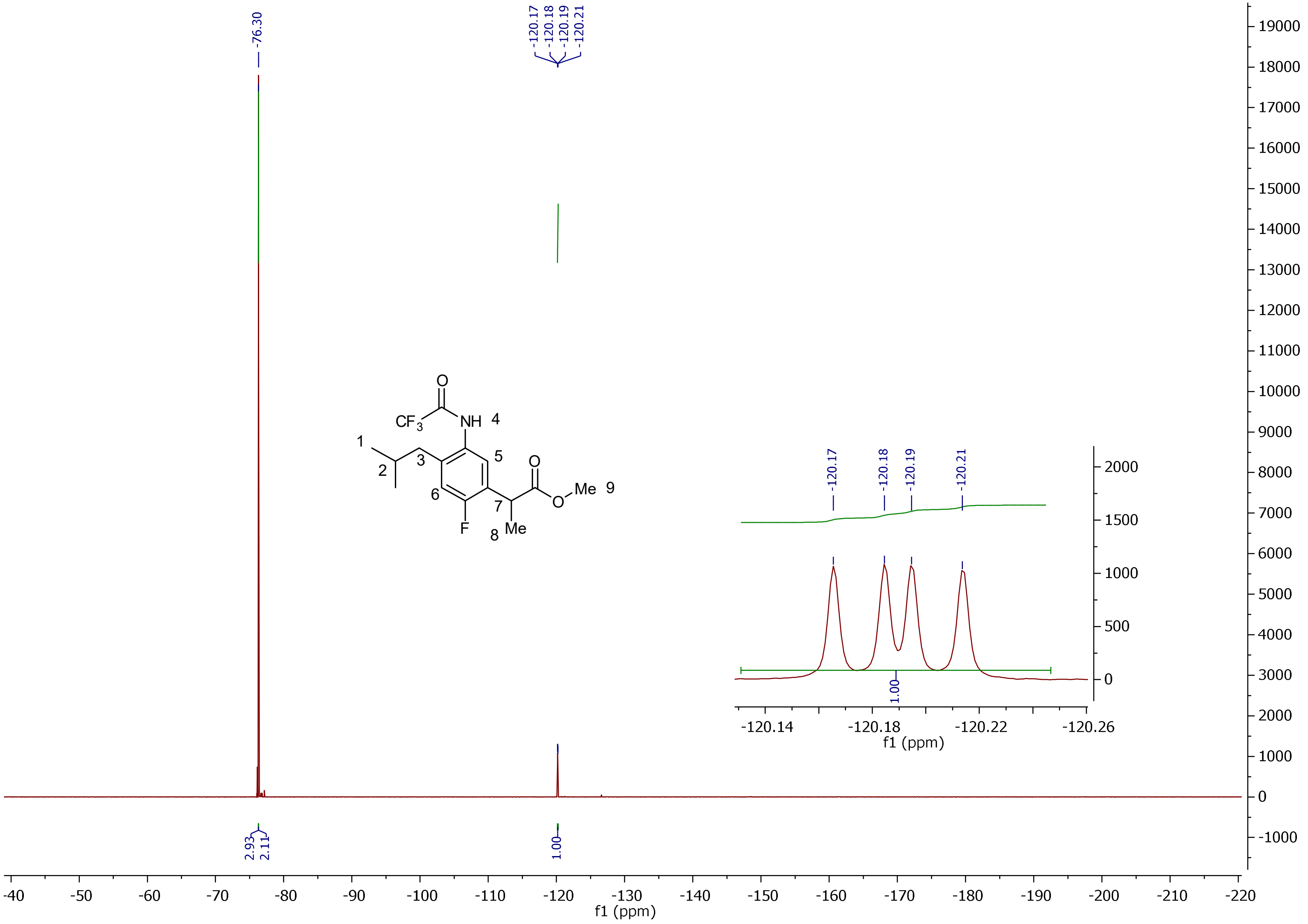

HMBC NMR (CD$_3$CN) for fluorination of methyl 2-(4-isobutyl-3-(2,2,2-trifluoroacetamido)phenyl)propanoate **(3z)**

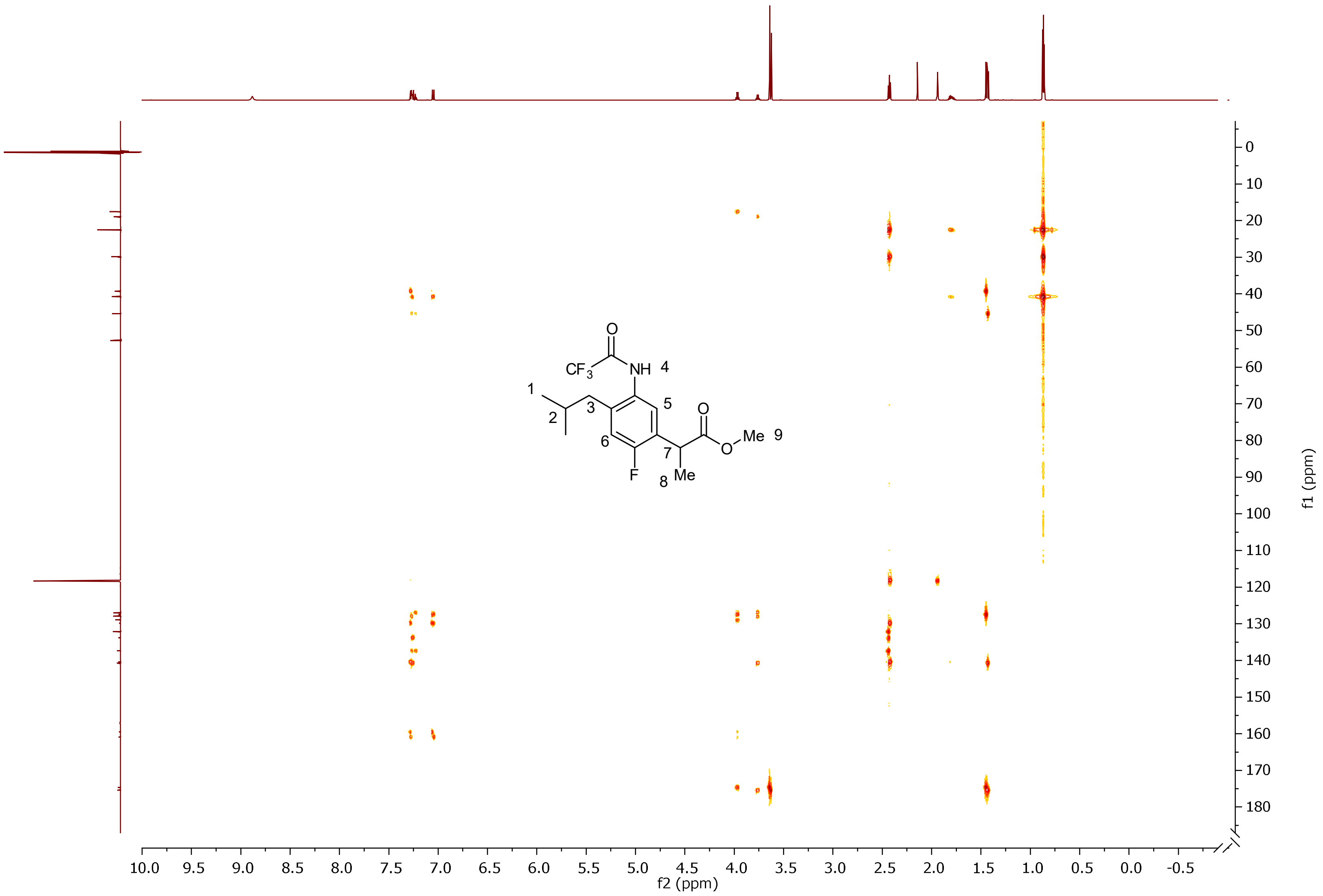

¹H NMR (700 MHz, CD_3CN) for fluorination of *(R)*-3-methoxy-2-methyl-3-oxopropyl 3-(2,2,2-trifluoroacetamido)benzoate **(3aa)**

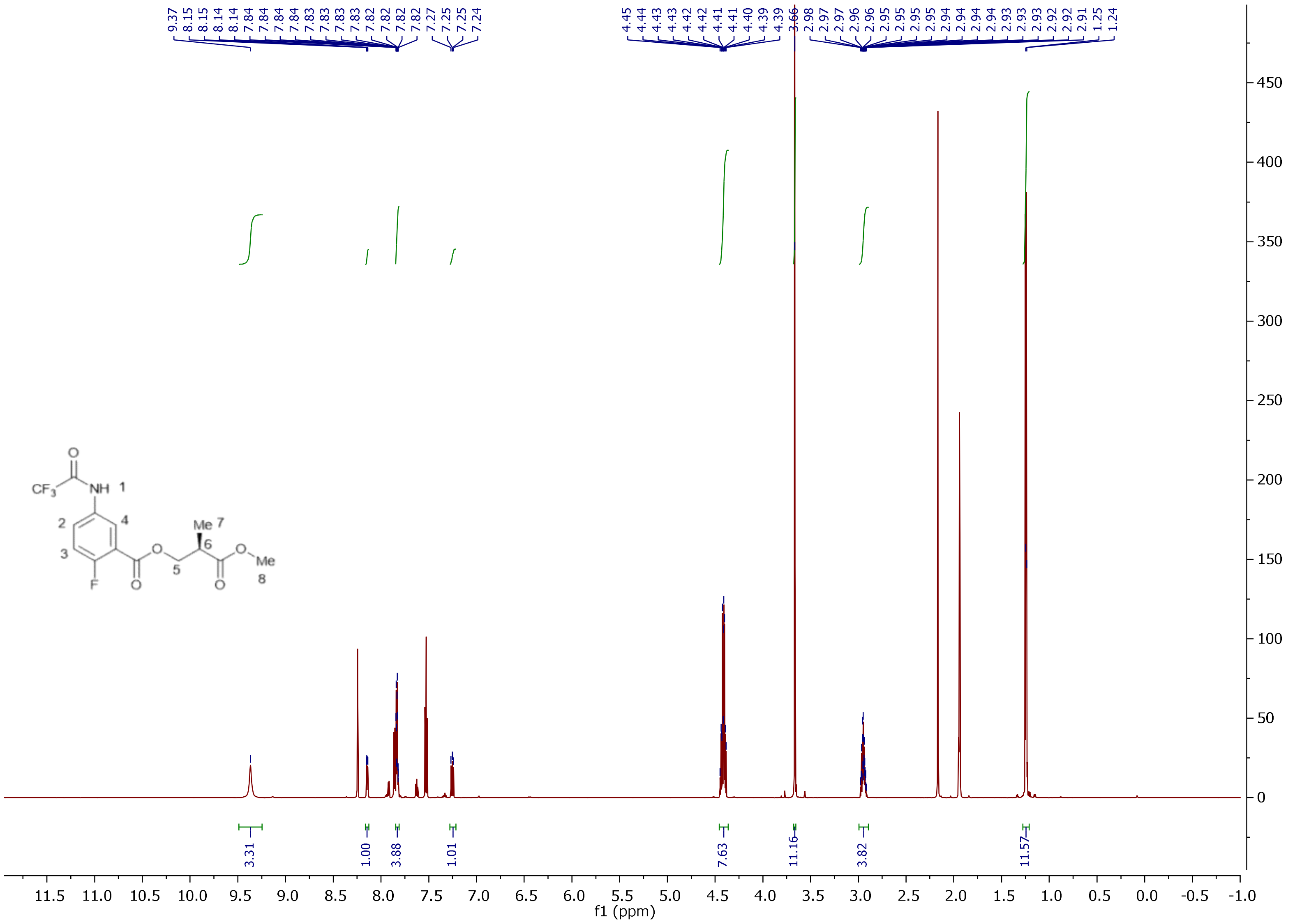

^{13}C NMR (176 MHz, CD_3CN) for fluorination of *(R)*-3-methoxy-2-methyl-3-oxopropyl 3-(2,2,2-trifluoroacetamido)benzoate **(3aa)**

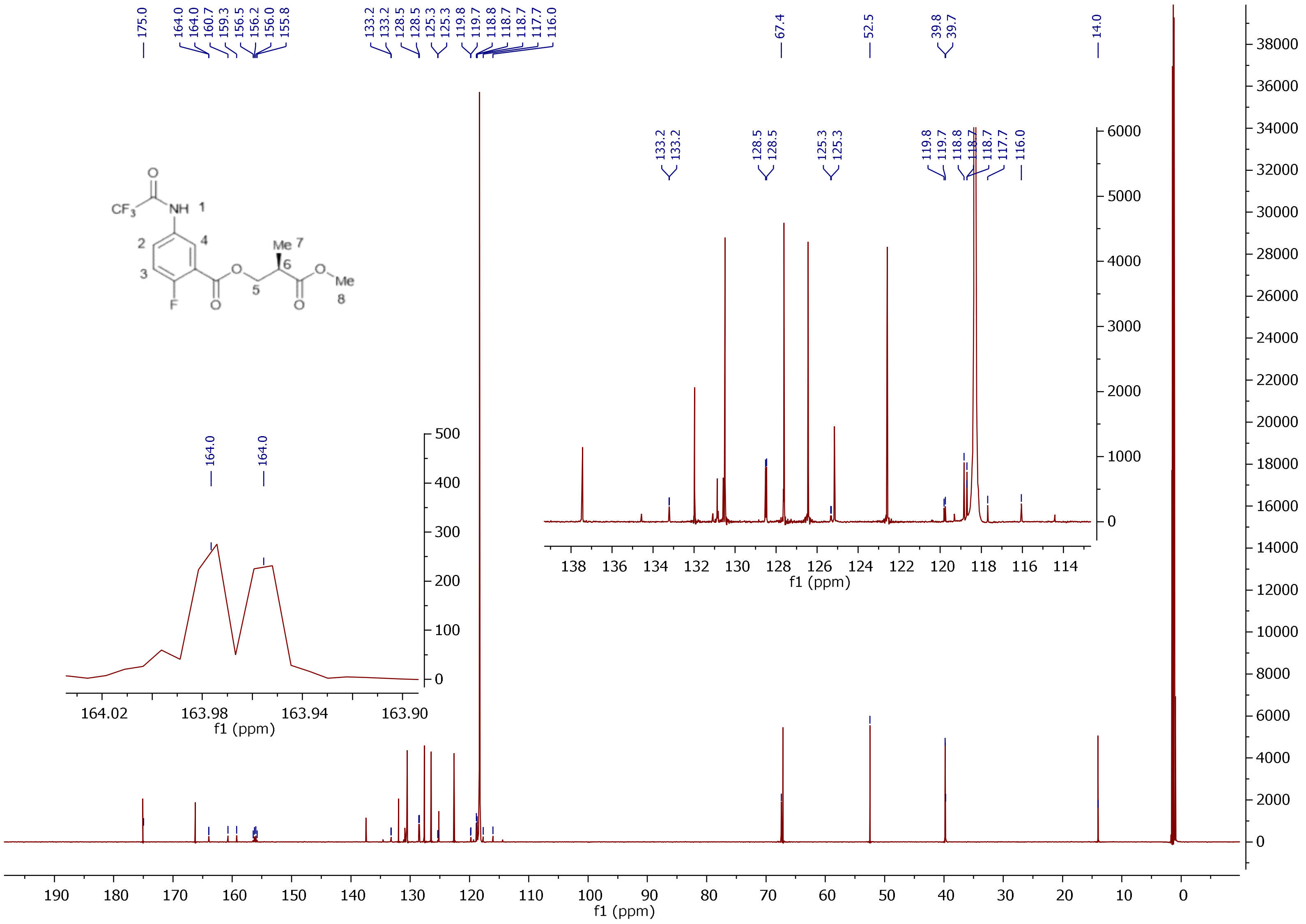

$^{19}F\{^1H\}$ NMR (376 MHz, CD_3CN) for fluorination of *(R)*-3-methoxy-2-methyl-3-oxopropyl 3-(2,2,2-trifluoroacetamido)benzoate **(3aa)**

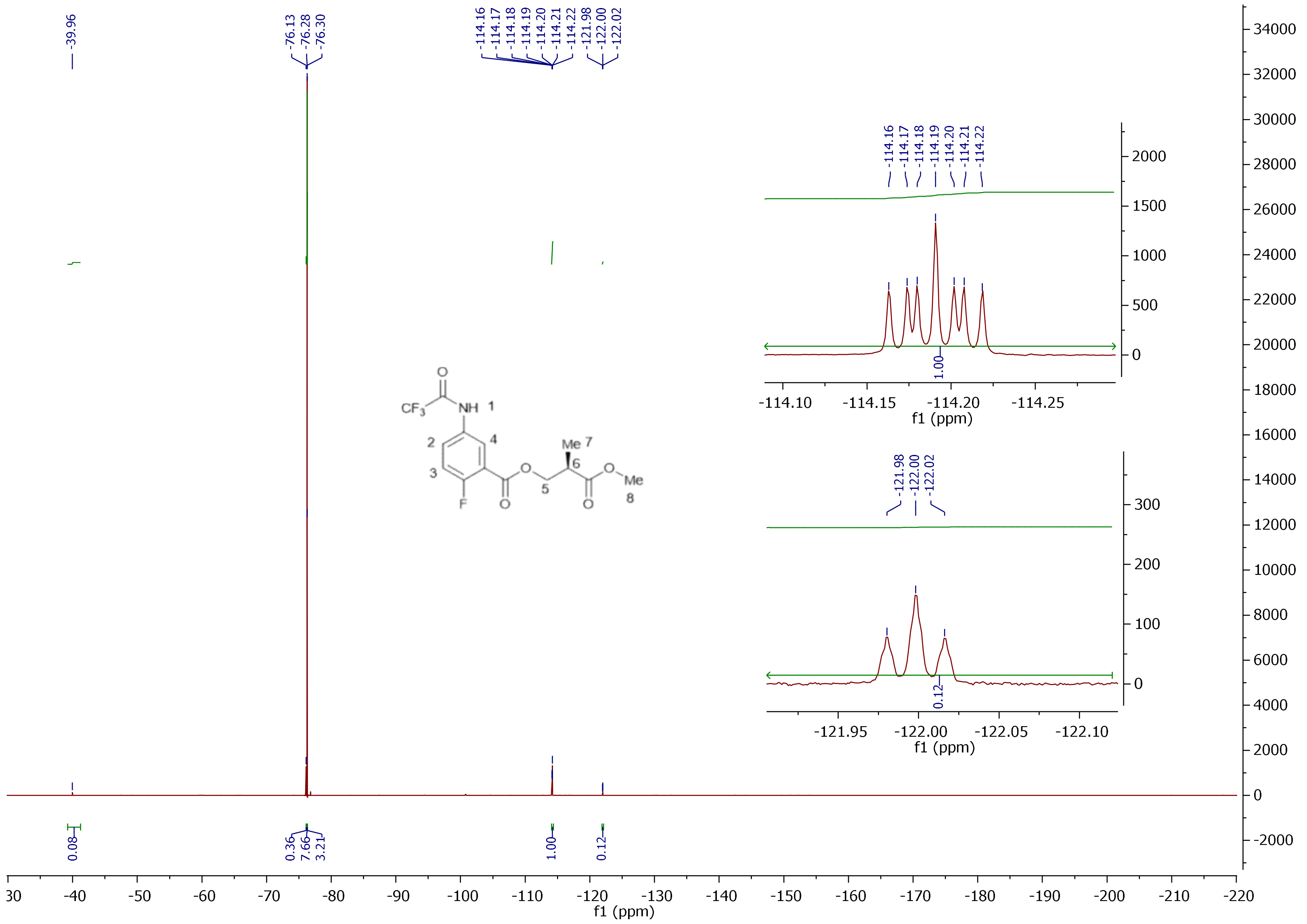

¹H NMR (400 MHz, $CDCl_3$) for 4-methyl-*N*-phenylbenzenesulfonamide

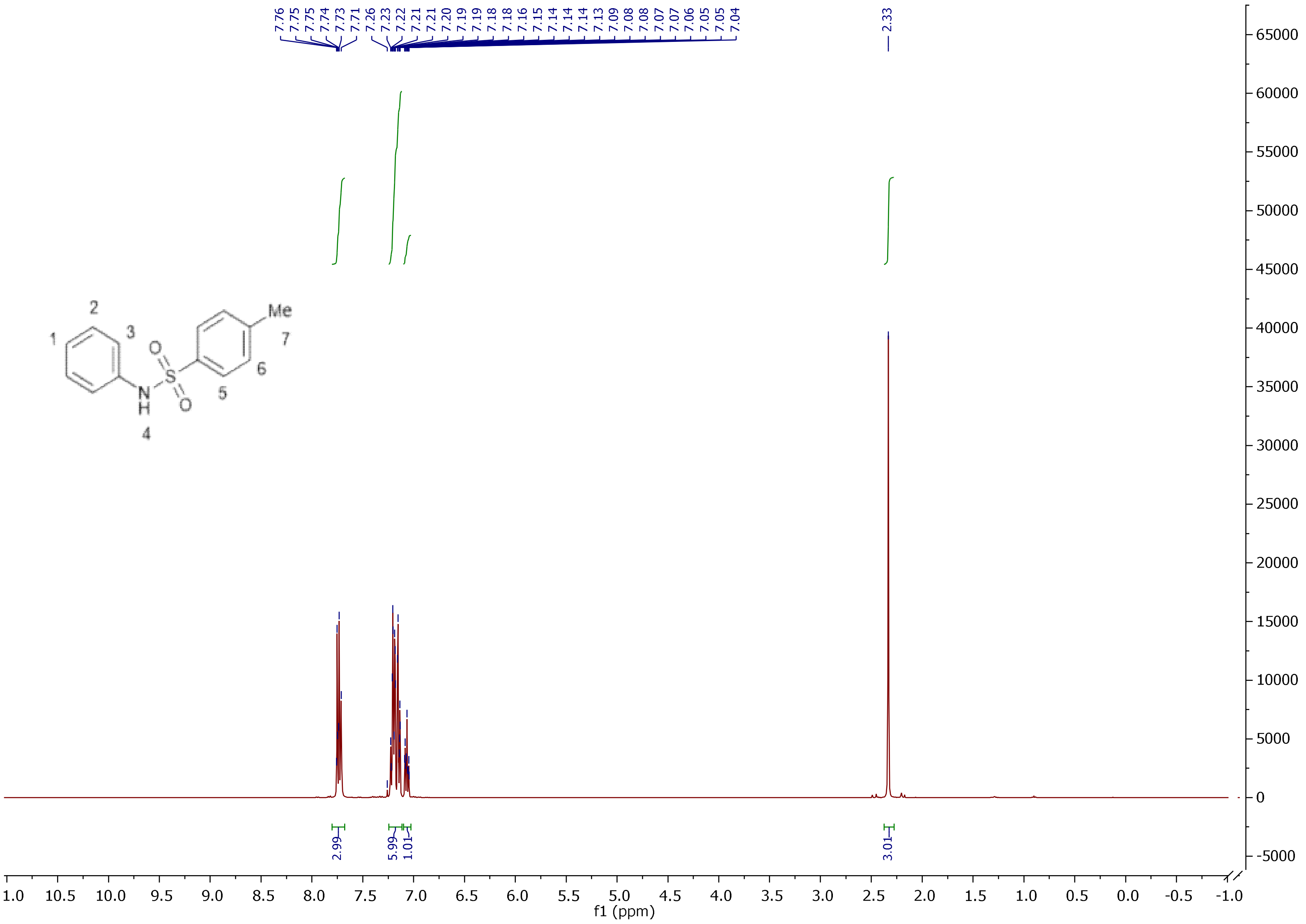

^{13}C NMR (126 MHz, $CDCl_3$) for 4-methyl-*N*-phenylbenzenesulfonamide

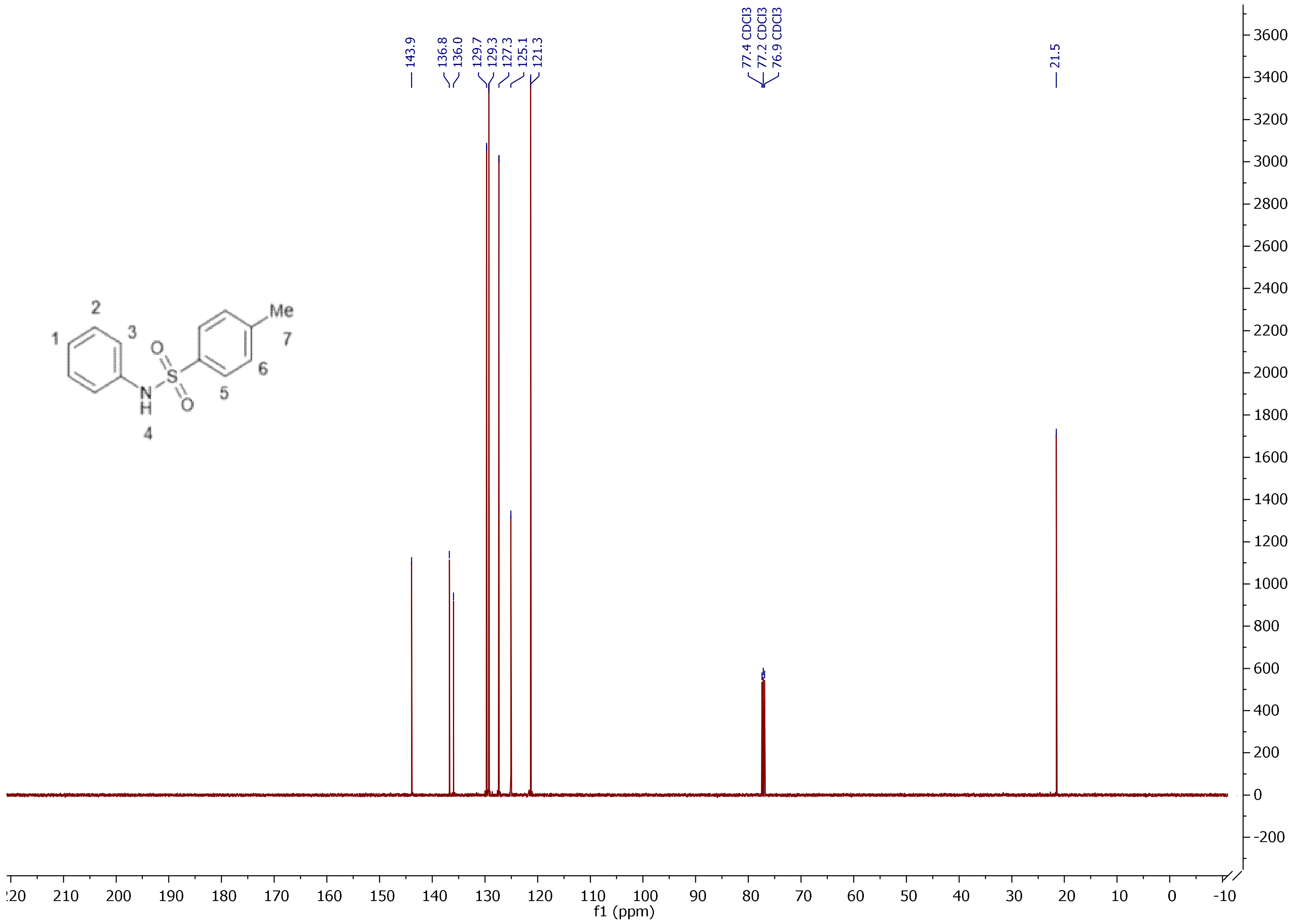

^{1}H NMR (500 MHz, $CDCl_3$) for 1,1,1-trifluoro-*N*-phenylmethanesulfonamide

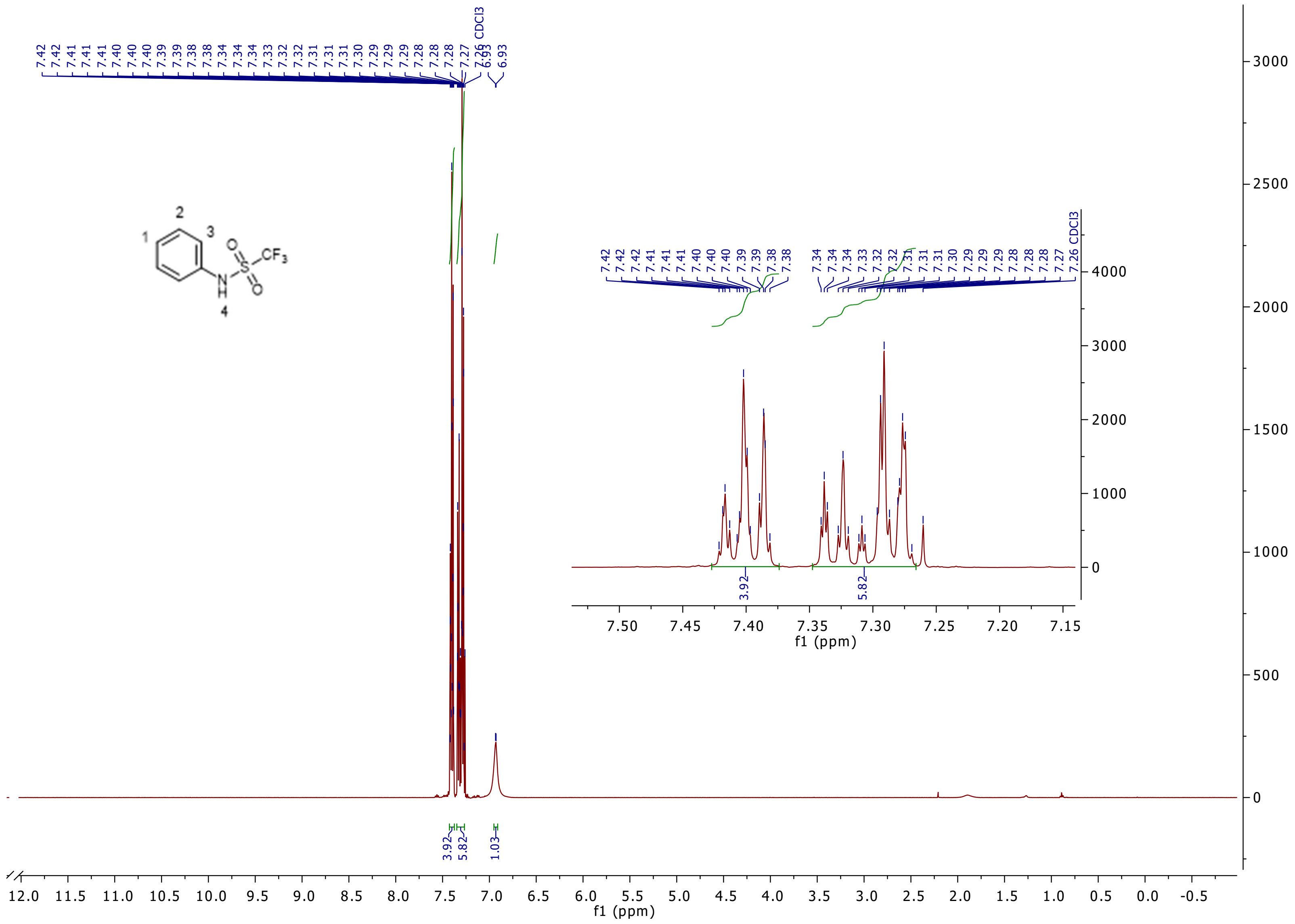

^{13}C NMR (126 MHz, $CDCl_3$) for 1,1,1-trifluoro-*N*-phenylmethanesulfonamide

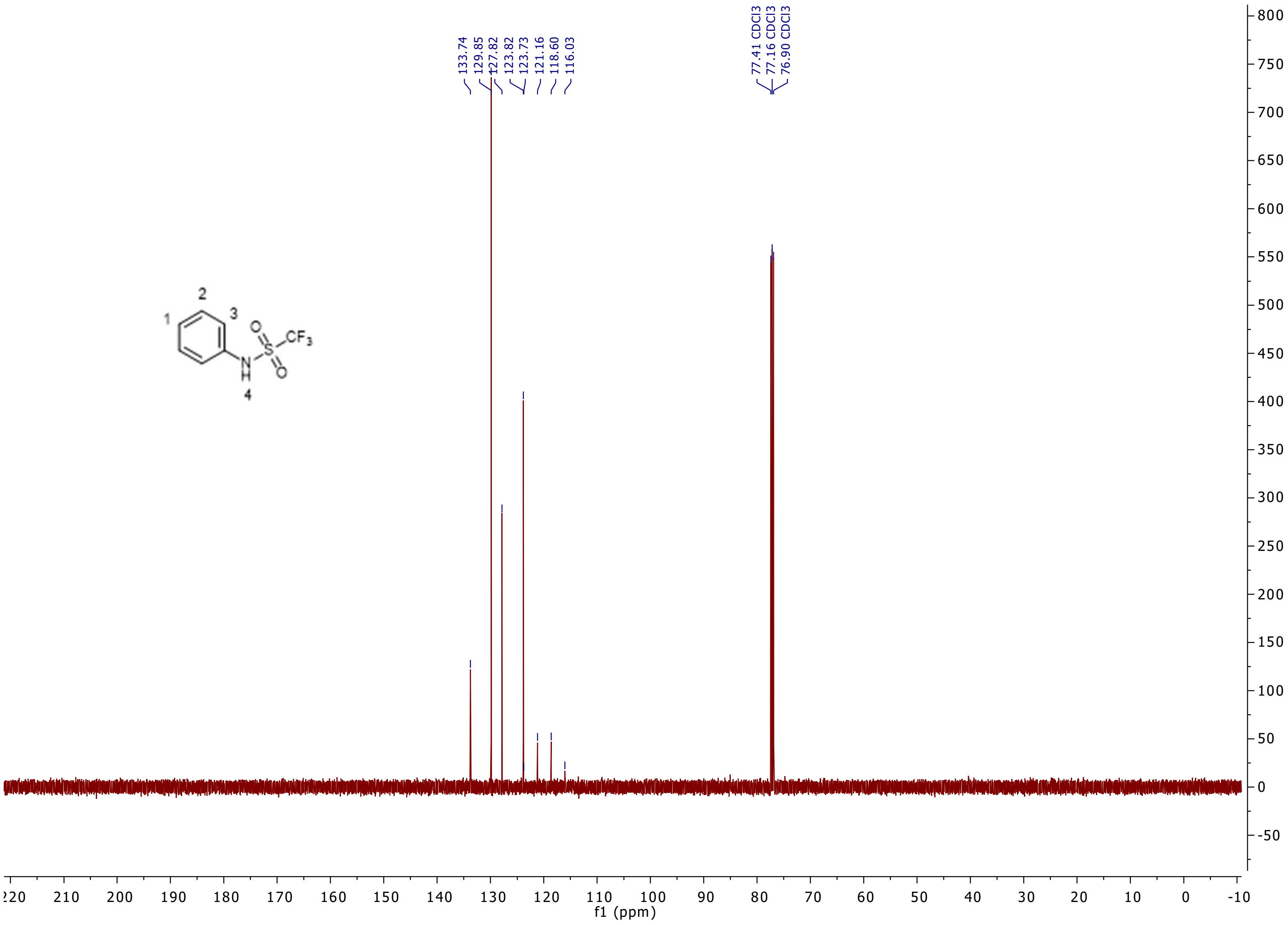

^{19}F NMR (376 MHz, $CDCl_3$) for 1,1,1-trifluoro-*N*-phenylmethanesulfonamide

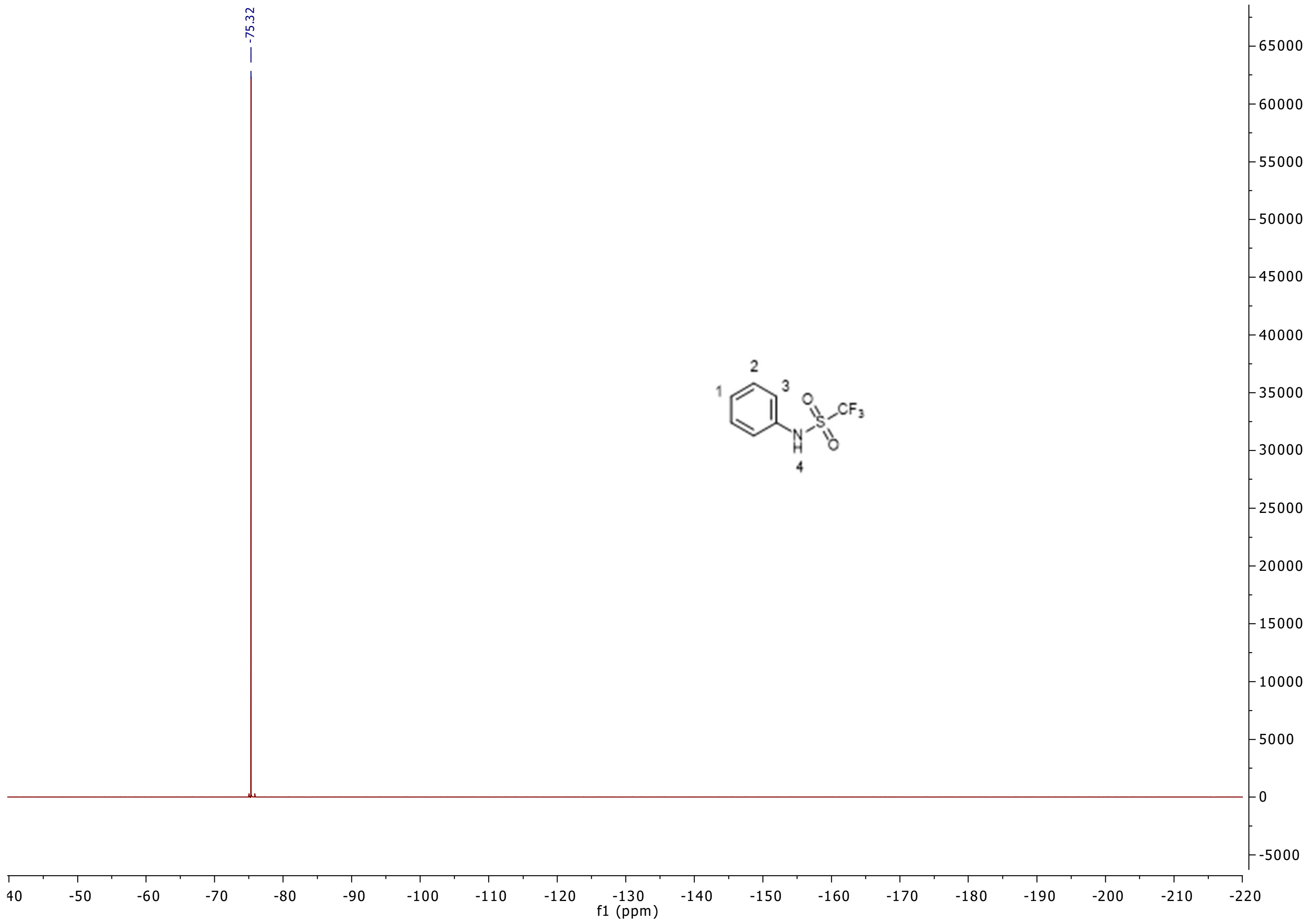

¹H NMR (500 MHz, $CDCl_3$) for *N*-(2-chlorophenyl)-2,2,2-trifluoroacetamide **(1ab)**

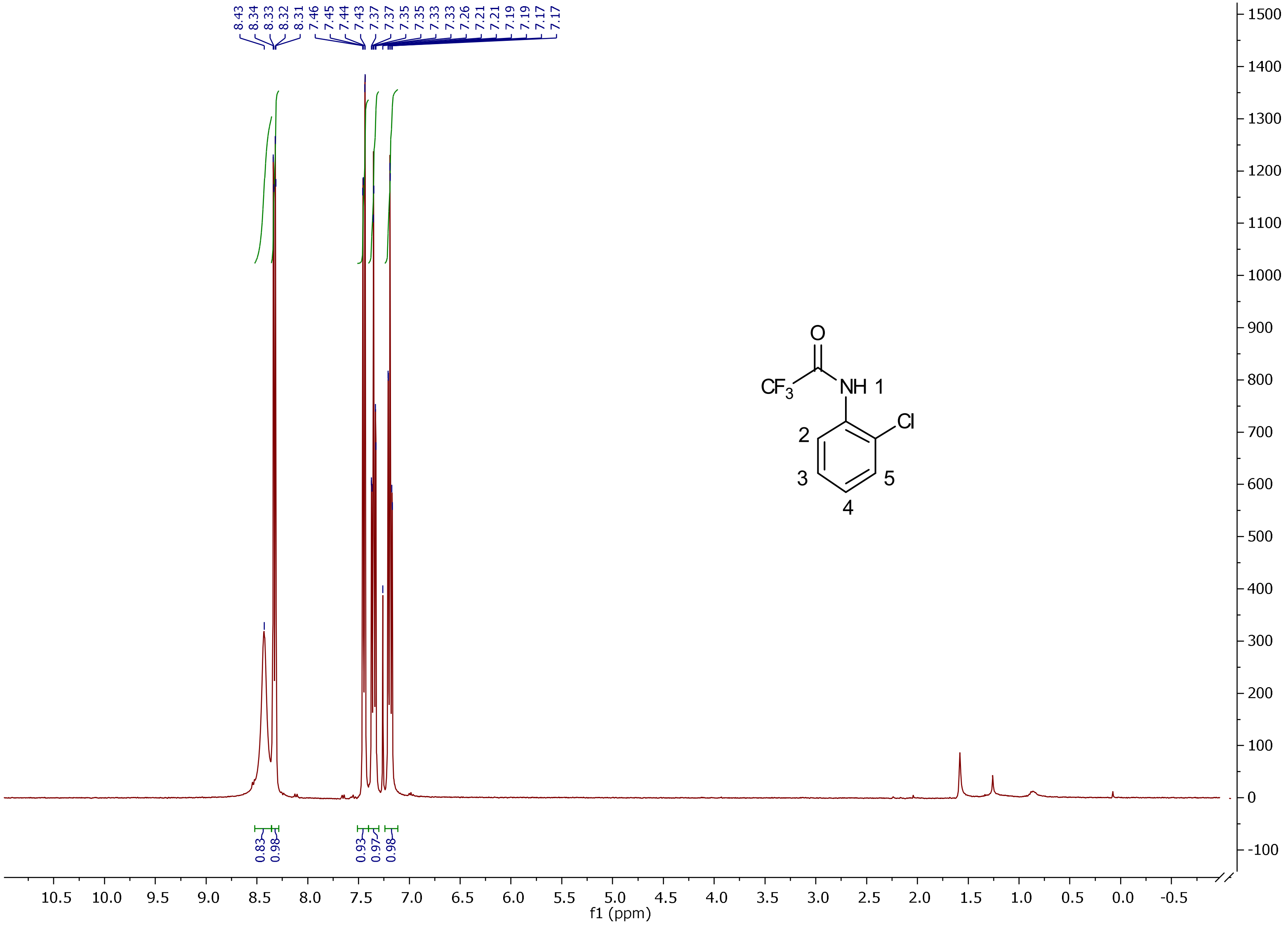

^{13}C NMR (126 MHz, $CDCl_3$) for *N*-(2-chlorophenyl)-2,2,2-trifluoroacetamide **(1ab)**

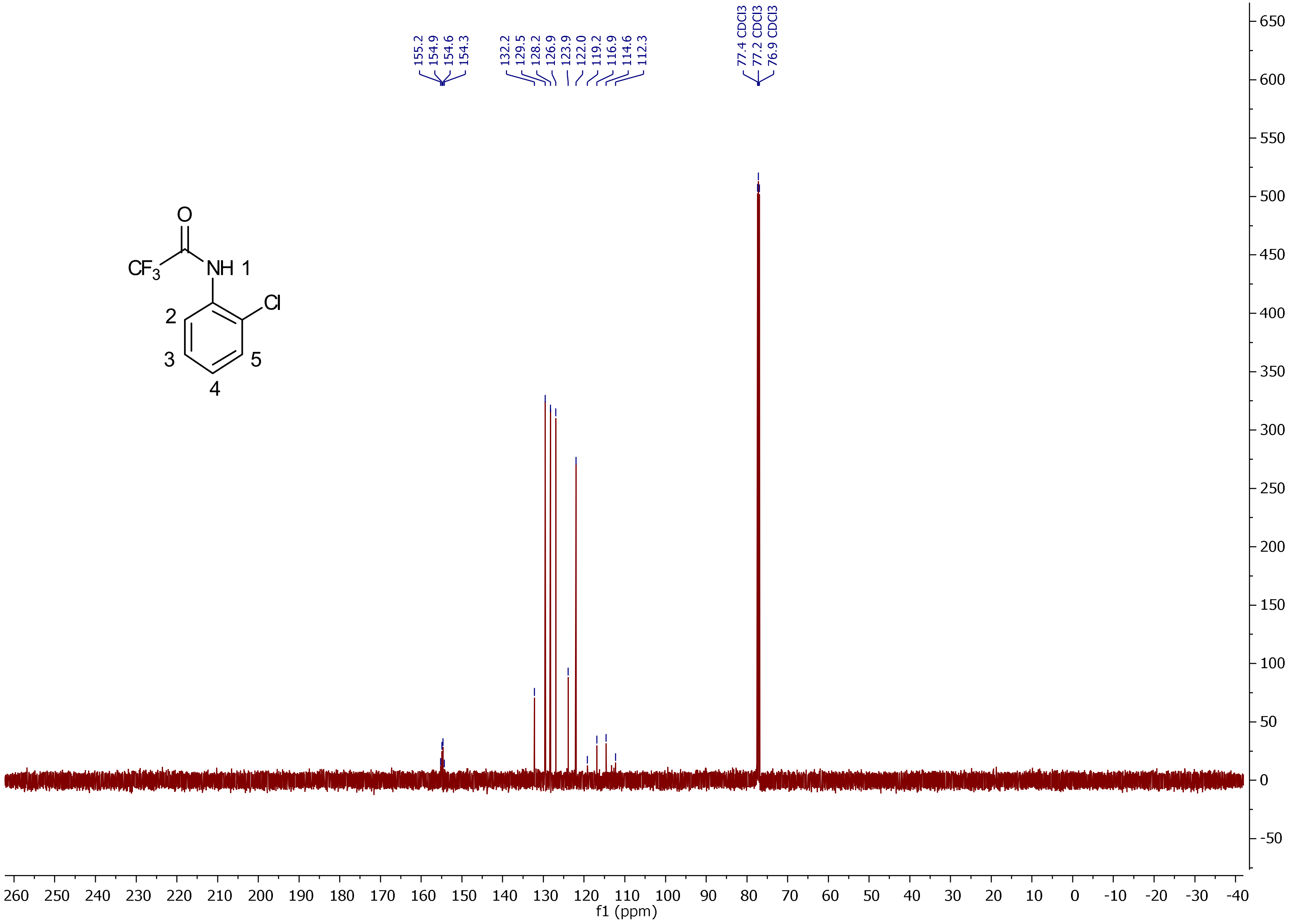

^{19}F NMR (376 MHz, $CDCl_3$) for *N*-(2-chlorophenyl)-2,2,2-trifluoroacetamide **(1ab)**

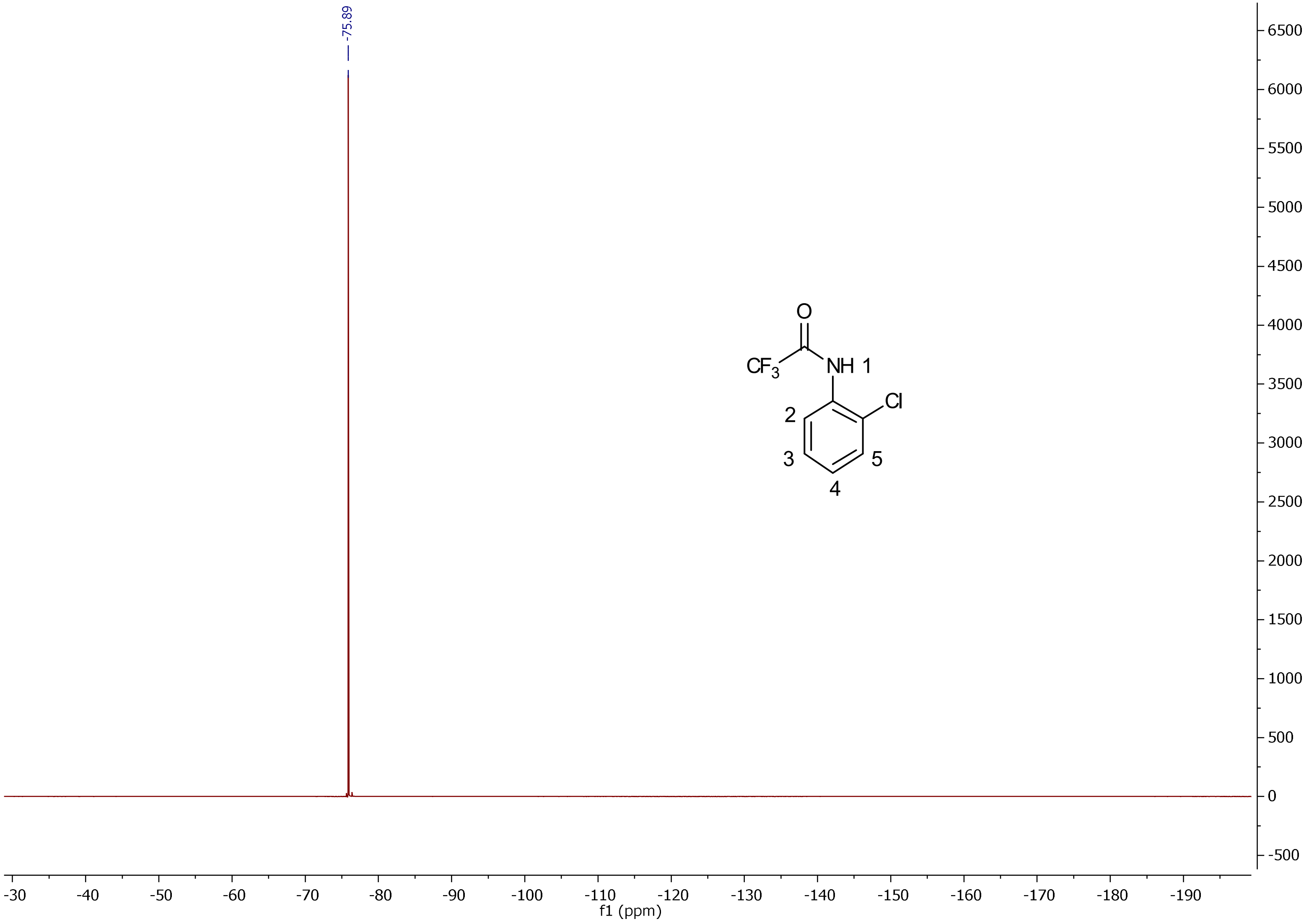

^{1}H NMR (400 MHz, $CDCl_3$) for 2,2,2-trifluoro-*N*-(8-oxo-5,6,7,8-tetrahydronaphthalen-1-yl)acetamide **(1ac)**

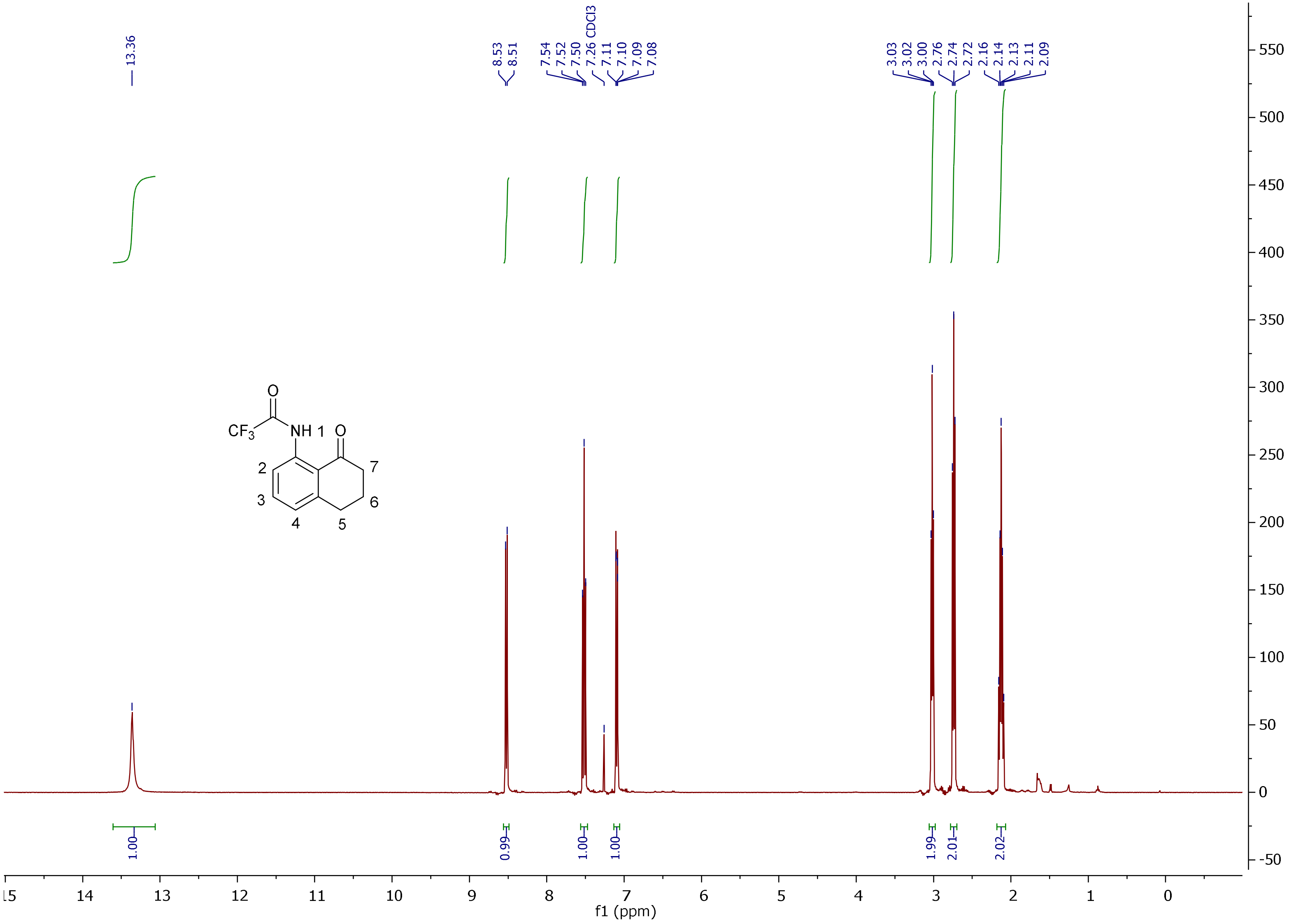

^{13}C NMR (101 MHz, $CDCl_3$) for 2,2,2-trifluoro-*N*-(8-oxo-5,6,7,8-tetrahydronaphthalen-1-yl)acetamide **(1ac)**

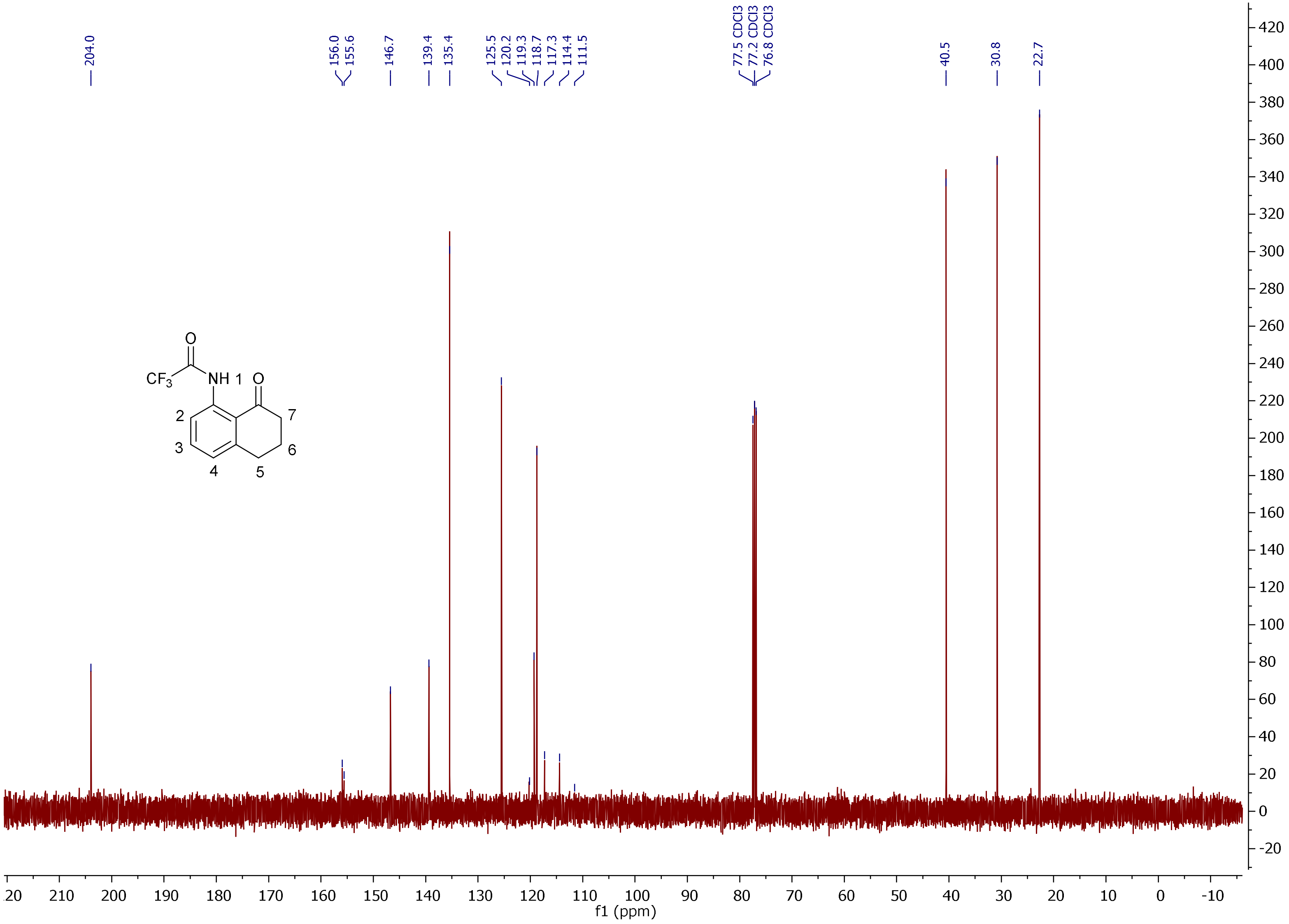

^{19}F NMR (376 MHz, $CDCl_3$) for 2,2,2-trifluoro-*N*-(8-oxo-5,6,7,8-tetrahydronaphthalen-1-yl)acetamide **(1ac)**

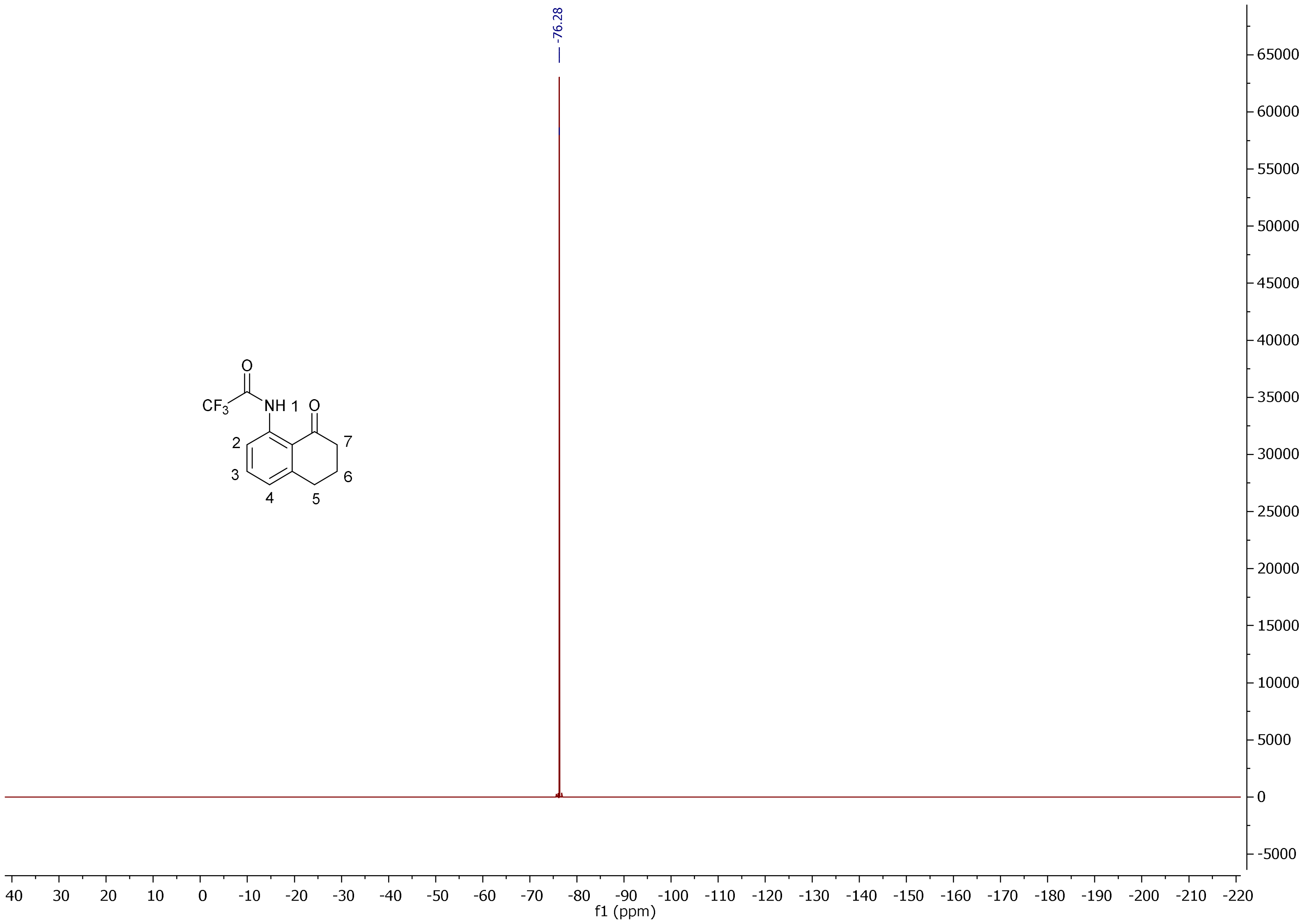

^{1}H NMR (400 MHz, $CDCl_3$) for 2,2,2-trifluoro-*N*-(2-(trifluoromethyl)phenyl)acetamide **(1ad)**

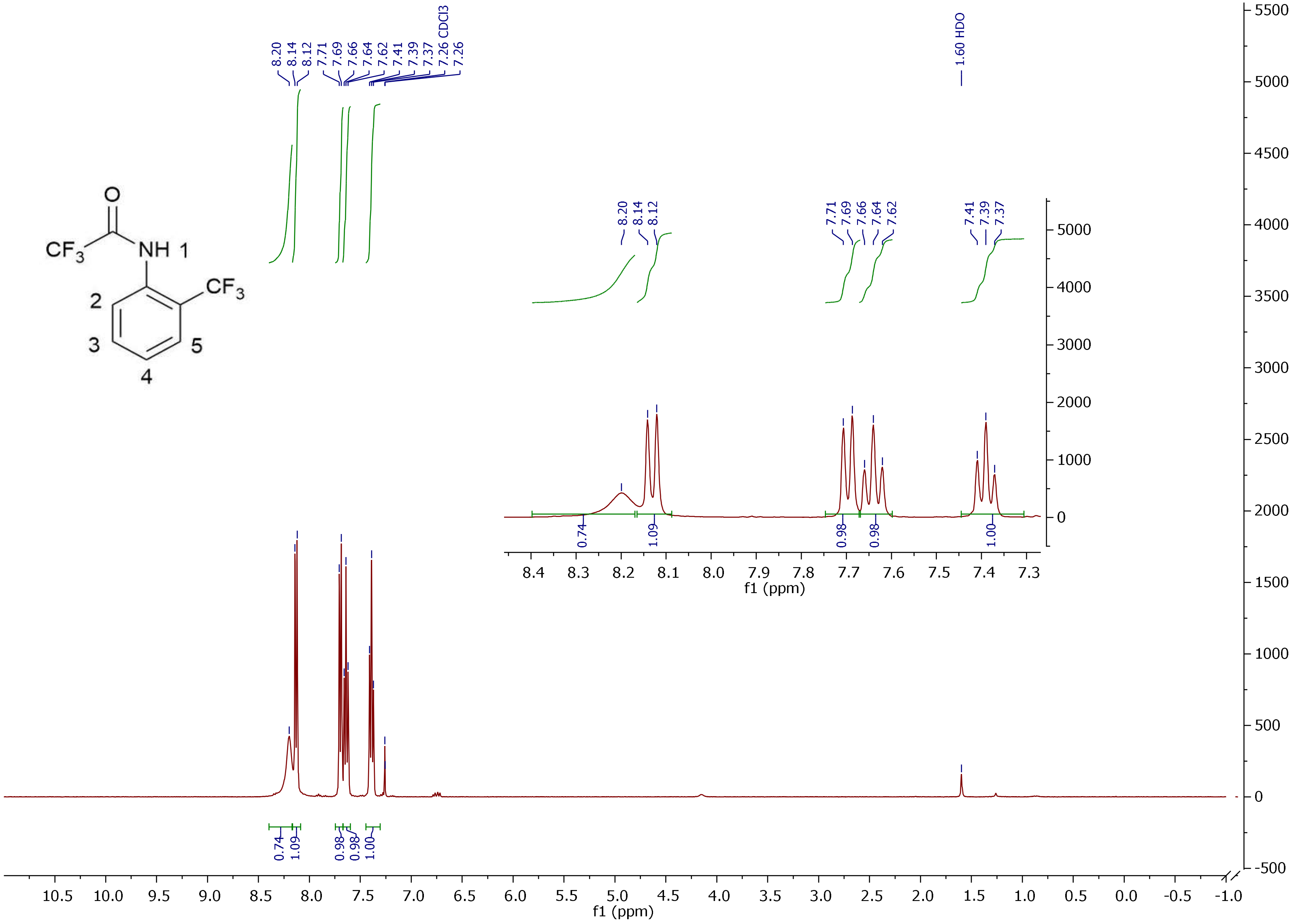

^{13}C NMR (101 MHz, $CDCl_3$) for 2,2,2-trifluoro-*N*-(2-(trifluoromethyl)phenyl)acetamide **(1ad)**

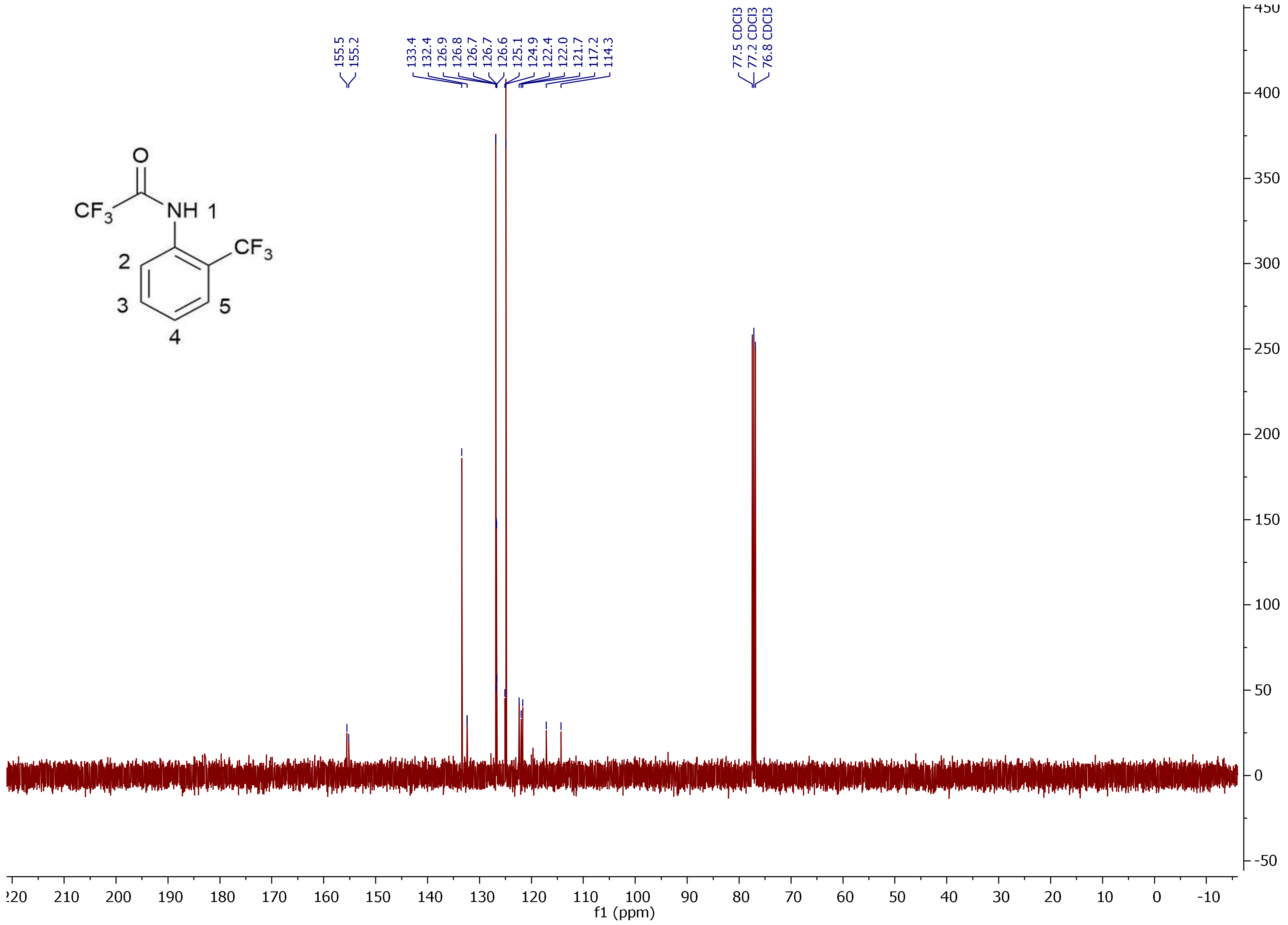

^{19}F NMR (376 MHz, $CDCl_3$) for 2,2,2-trifluoro-*N*-(2-(trifluoromethyl)phenyl)acetamide **(1ad)**

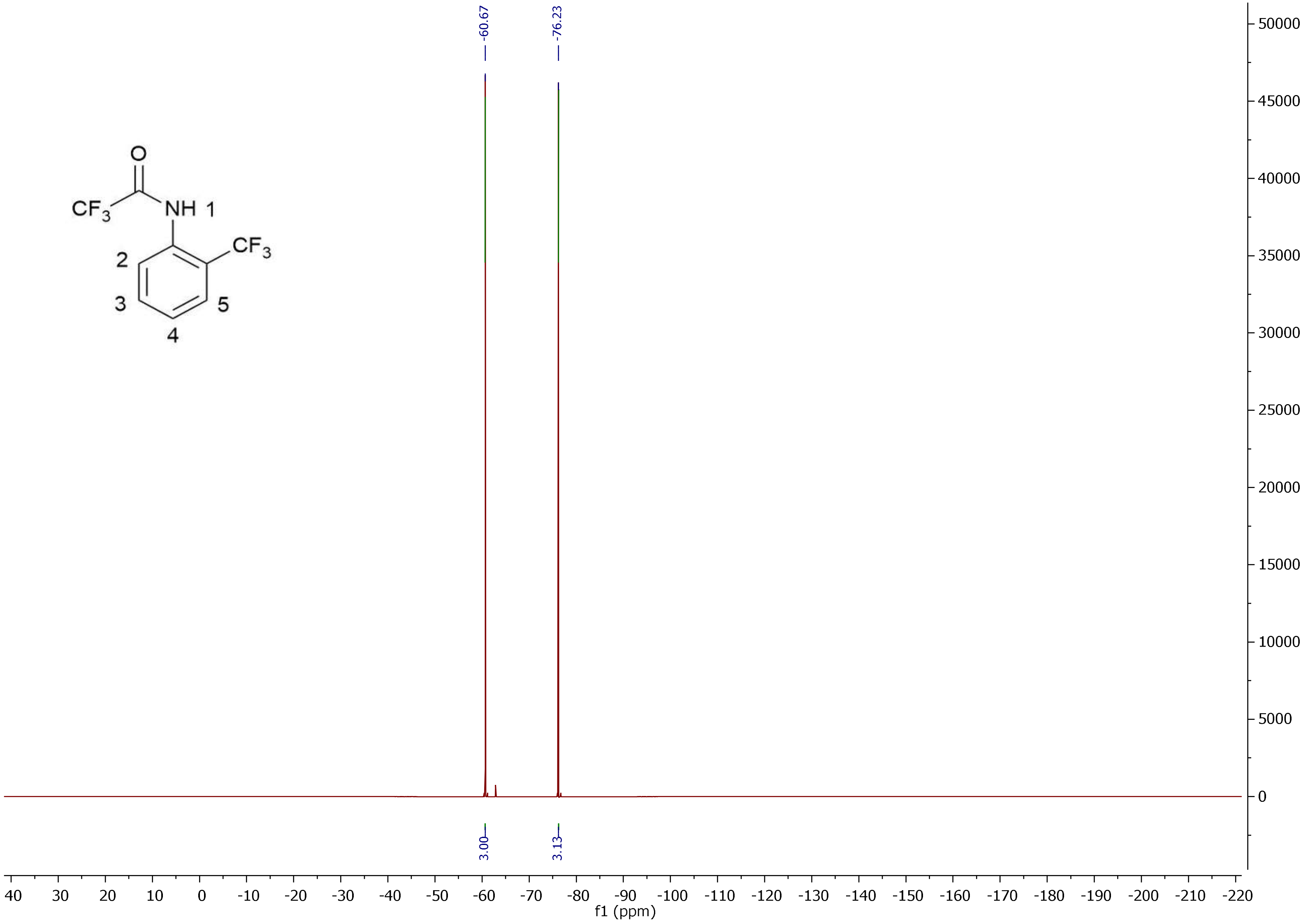

^{1}H NMR (700 MHz, $CDCl_3$) for *N*-([1,1'-biphenyl]-2-yl)-2,2,2-trifluoroacetamide **(1ae)**

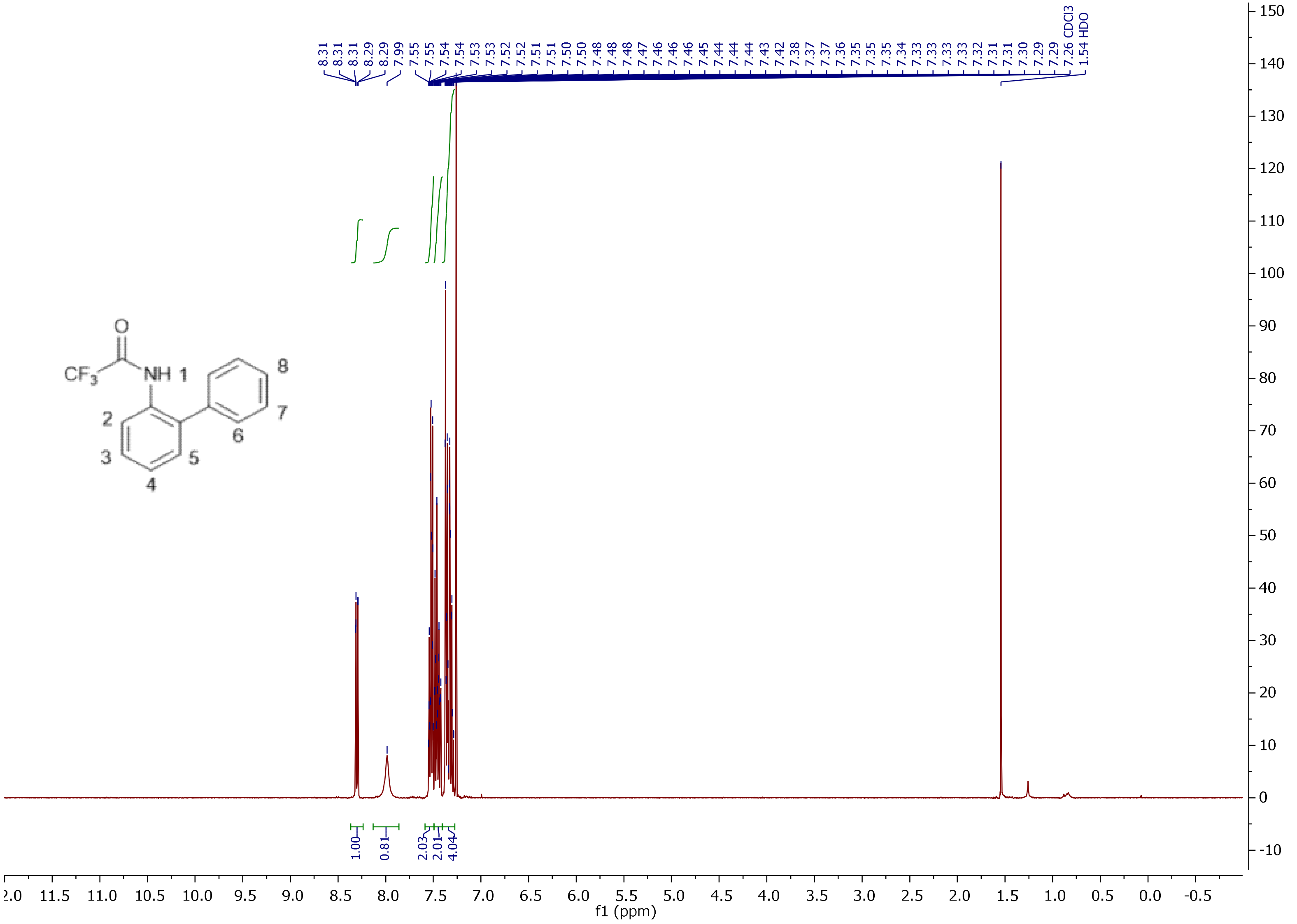

^{13}C NMR (176 MHz, $CDCl_3$) for *N*-([1,1'-biphenyl]-2-yl)-2,2,2-trifluoroacetamide **(1ae)**

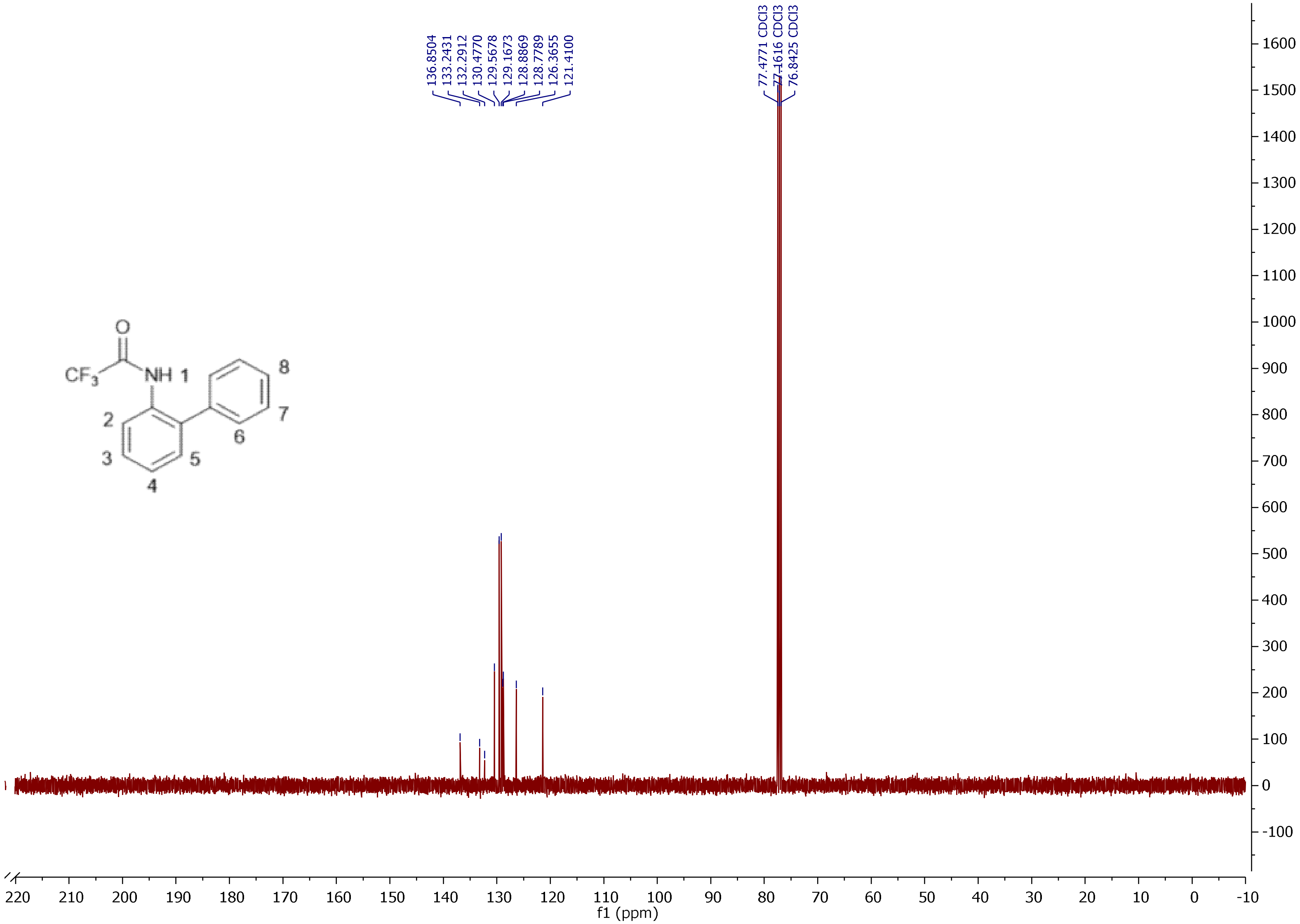

^{19}F NMR (400 MHz, $CDCl_3$) for *N*-([1,1'-biphenyl]-2-yl)-2,2,2-trifluoroacetamide **(1ae)**

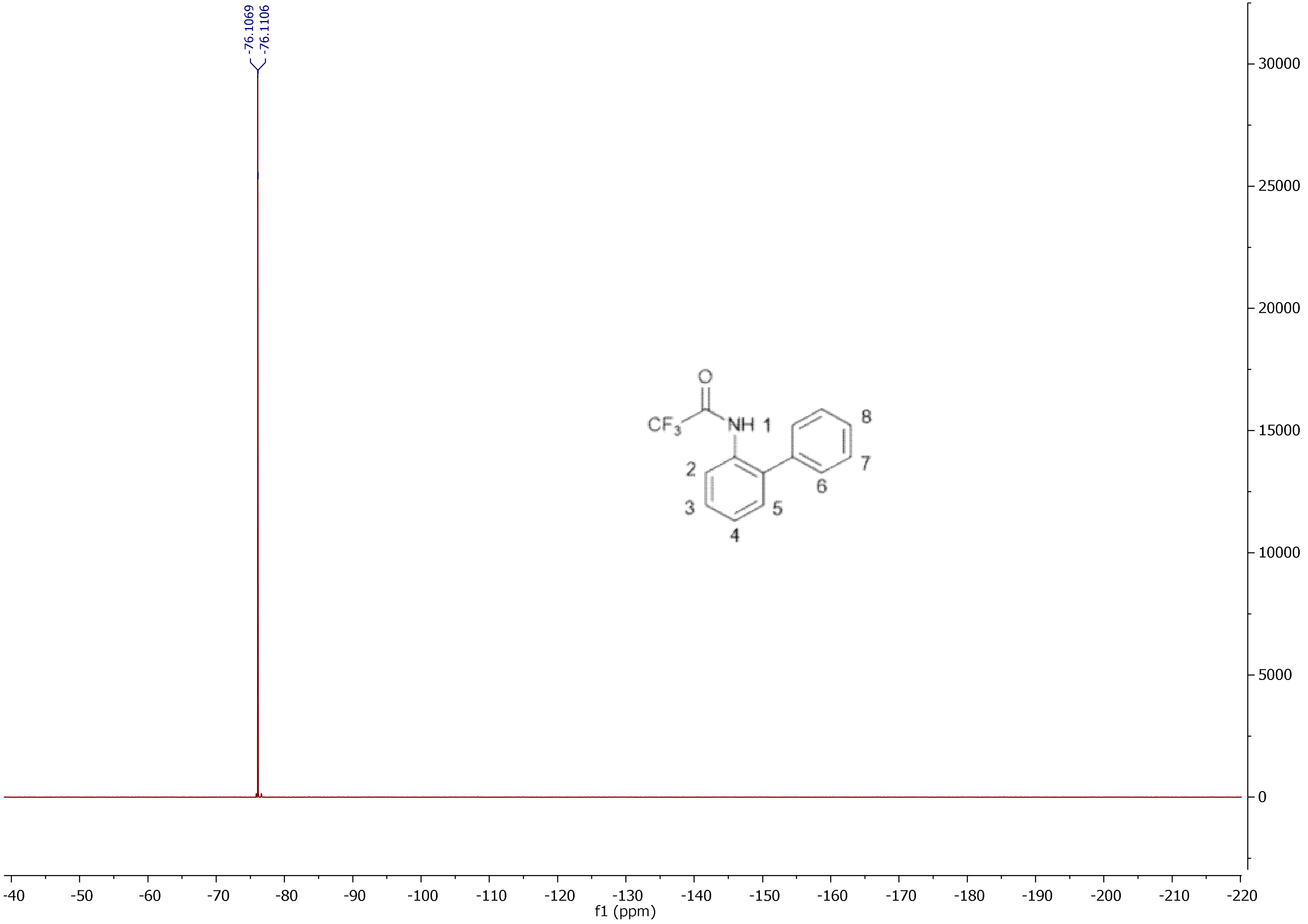

^{1}H NMR (400 MHz, $CDCl_3$) for 2,2,2-trifluoro-*N*-(3-iodophenyl)acetamide **(1af)**

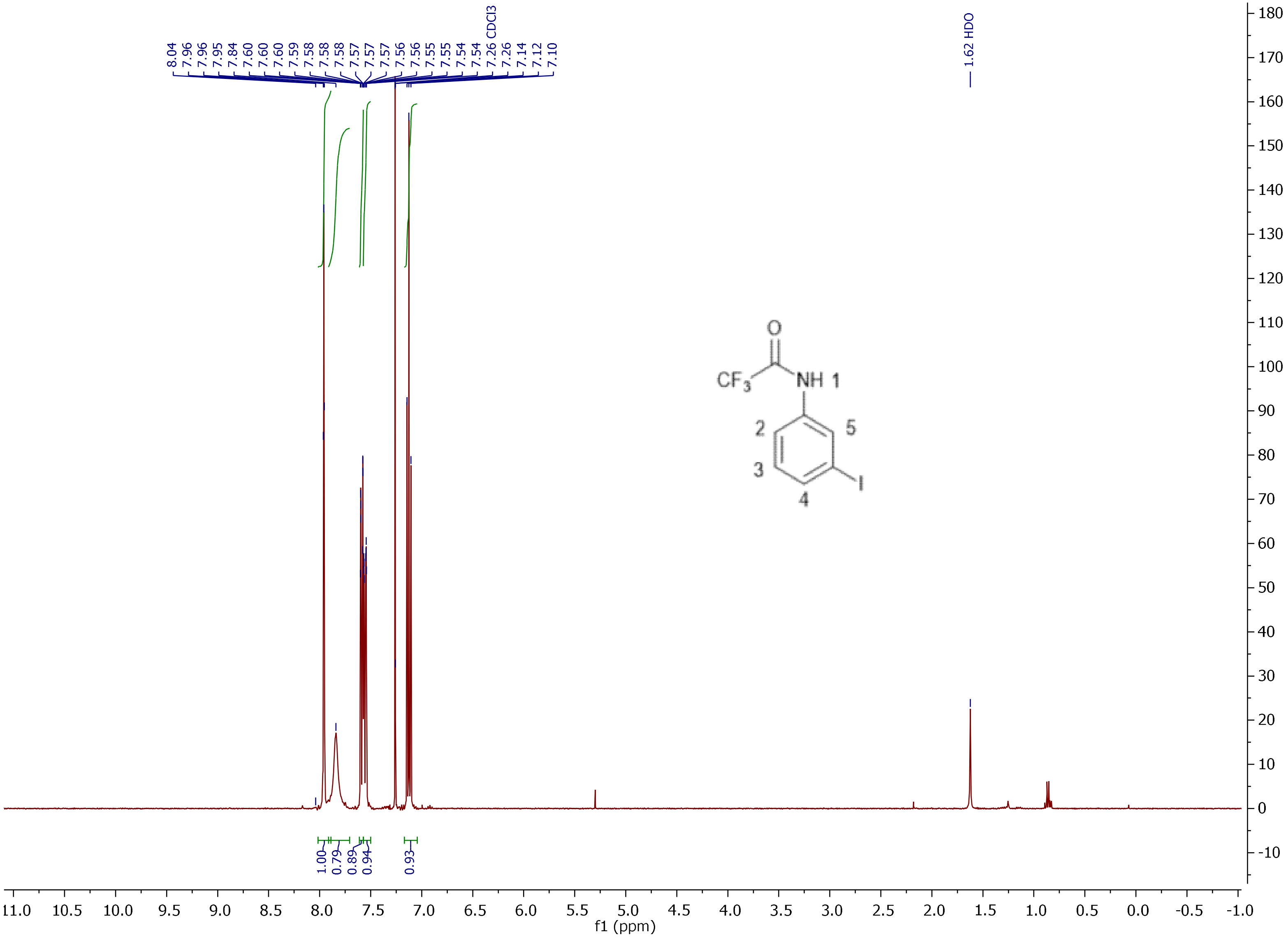

^{13}C NMR (101 MHz, $CDCl_3$) for 2,2,2-trifluoro-*N*-(3-iodophenyl)acetamide **(1af)**

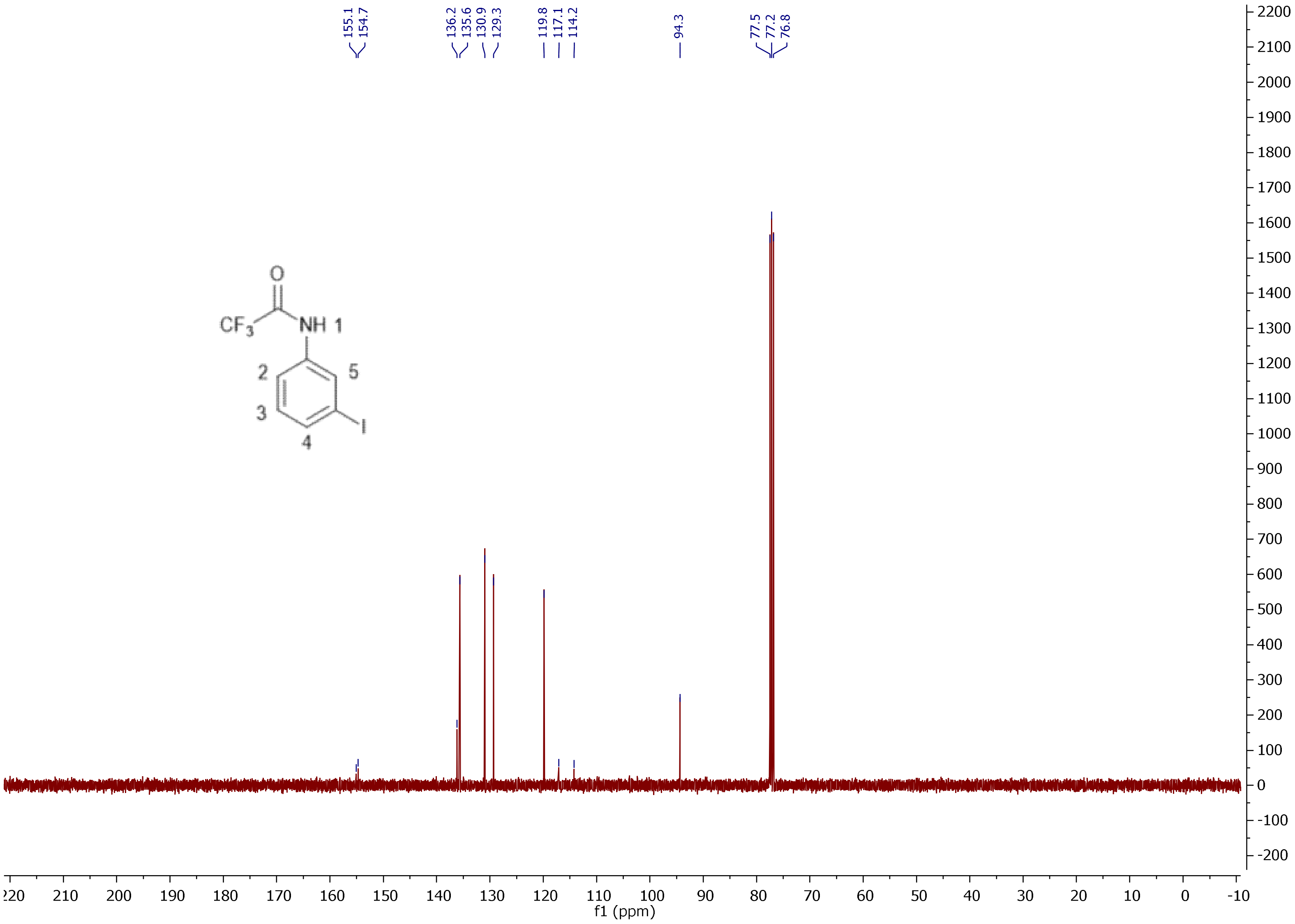

^{19}F NMR (376 MHz, $CDCl_3$) for 2,2,2-trifluoro-*N*-(3-iodophenyl)acetamide **(1af)**

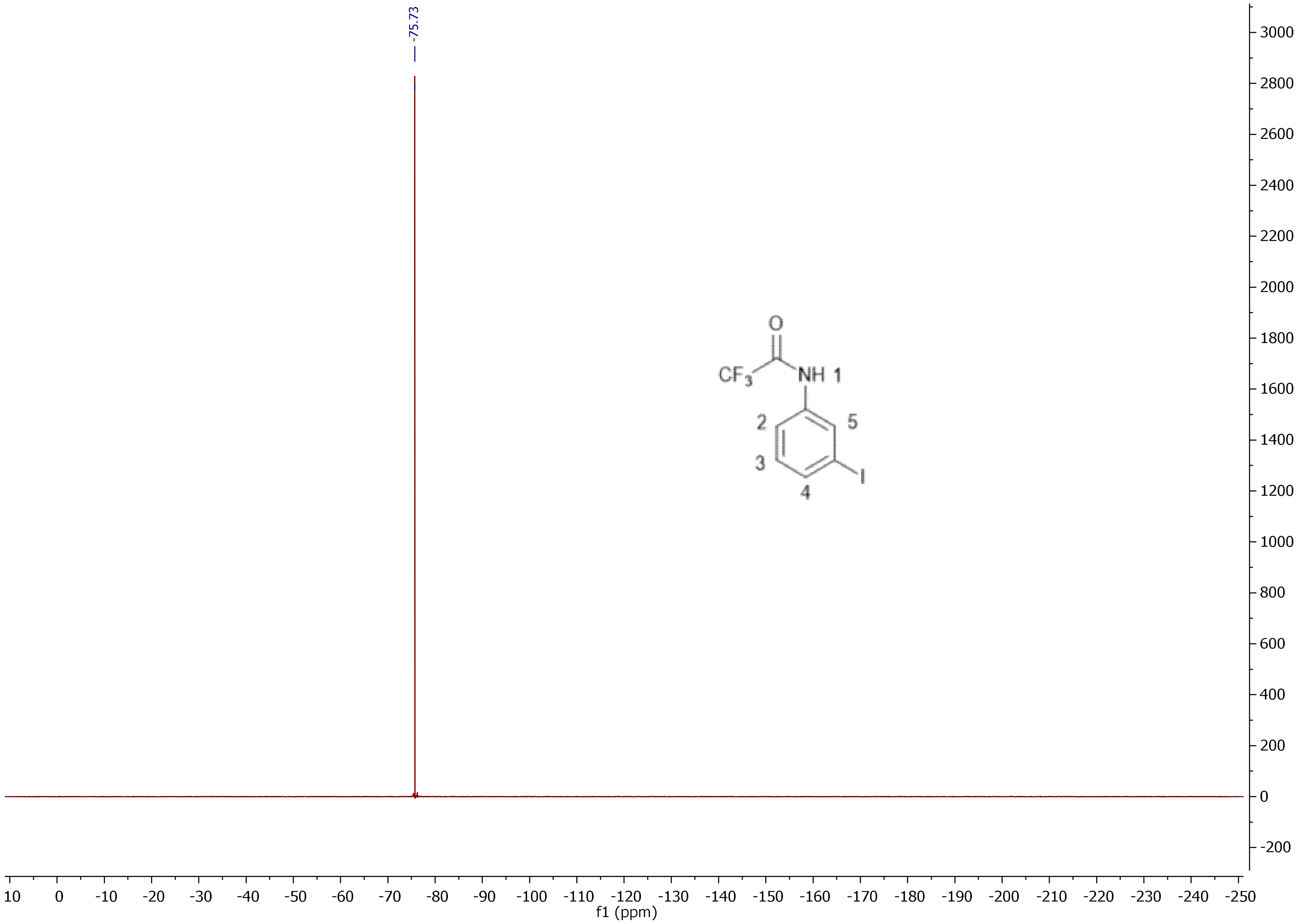

^{1}H NMR (400 MHz, $CDCl_3$) for 2,2,2-trifluoro-*N*-(naphthalen-1-yl)acetamide **(1ag)**

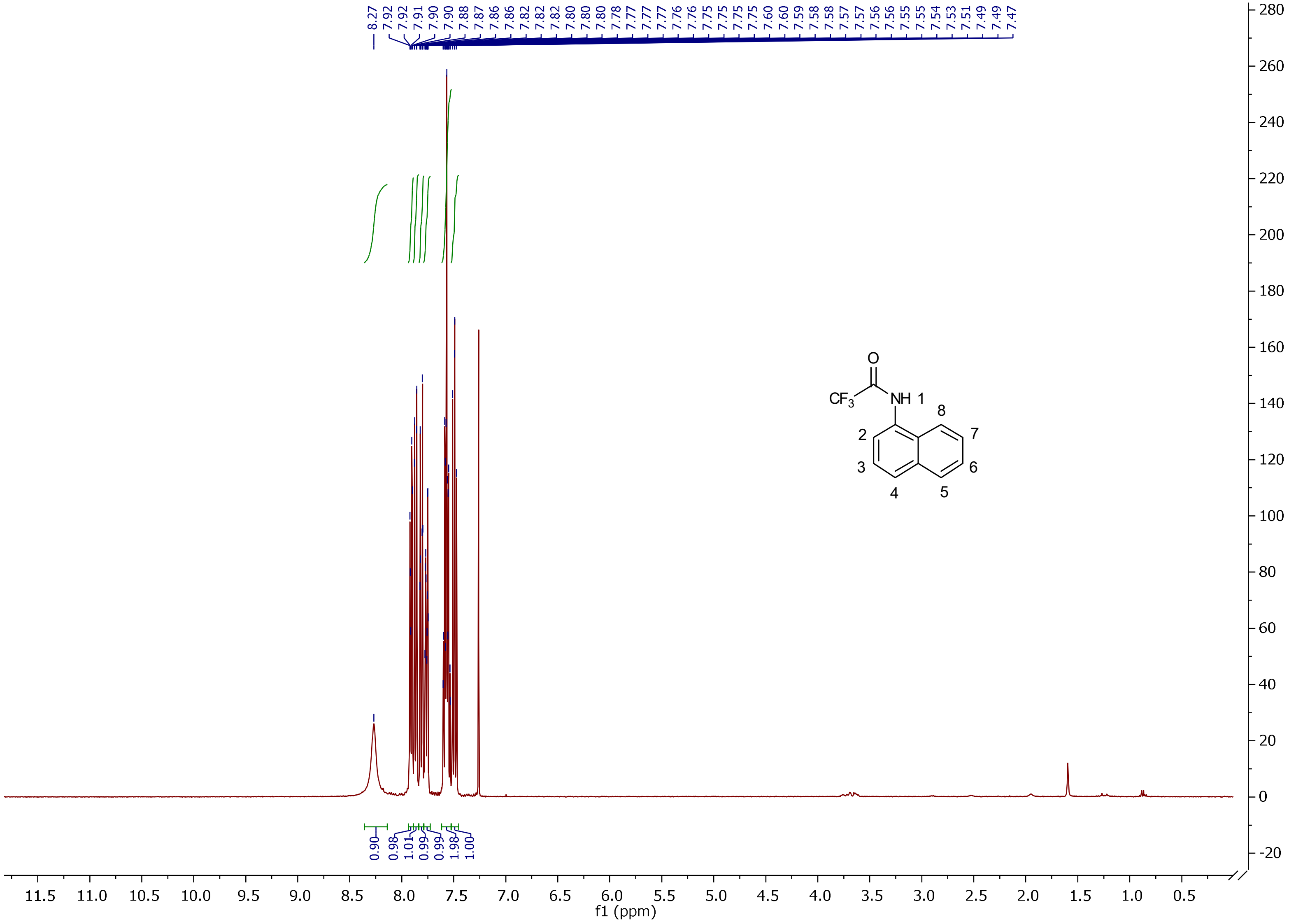

13C NMR (101 MHz, $CDCl_3$) for 2,2,2-trifluoro-*N*-(naphthalen-1-yl)acetamide **(1ag)**

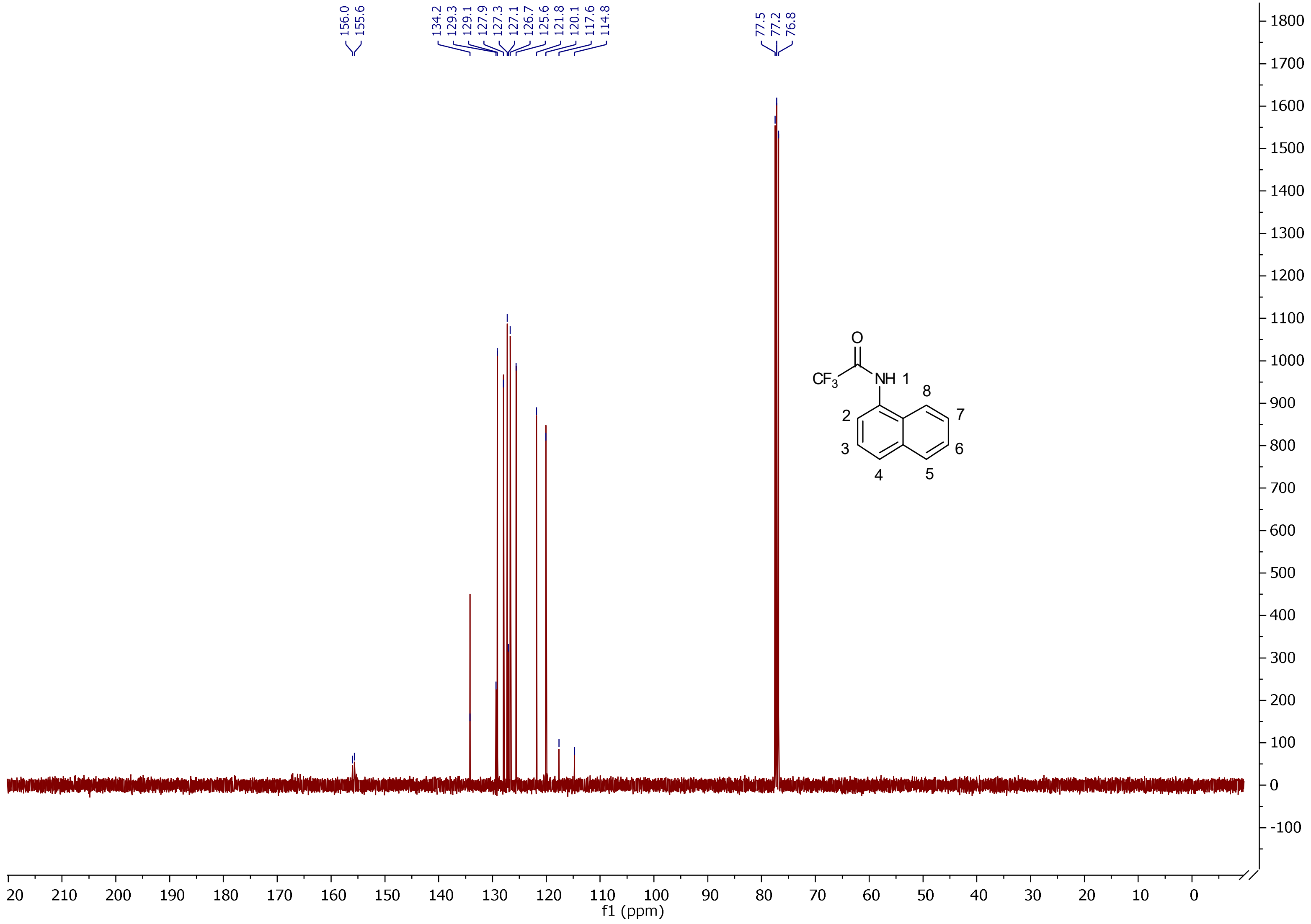

^{19}F NMR (376 MHz, $CDCl_3$) for 2,2,2-trifluoro-*N*-(naphthalen-1-yl)acetamide **(1ag)**

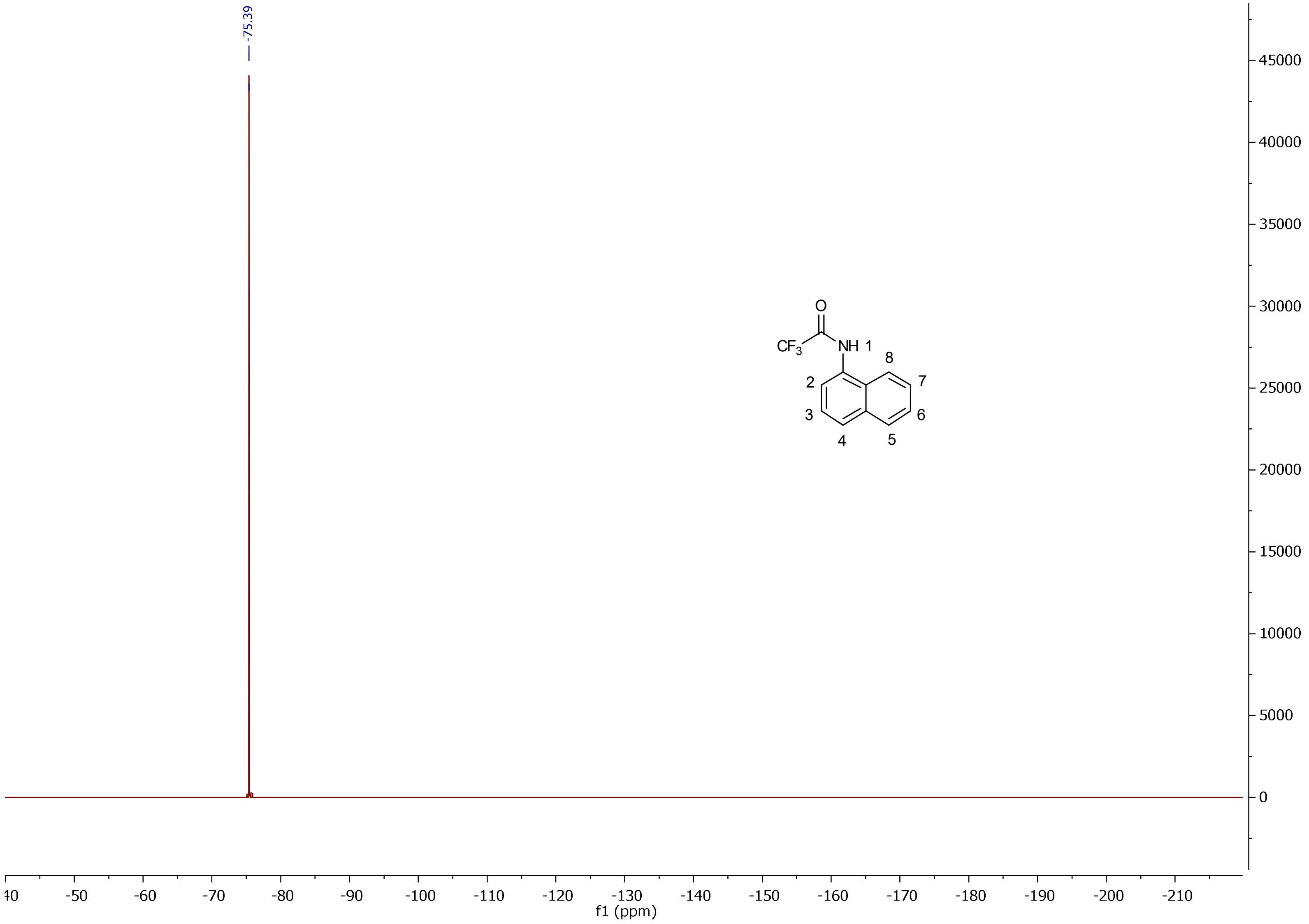

^{1}H NMR (700 MHz, CD_3CN) for fluorination of 4-methyl-*N*-phenylbenzenesulfonamide

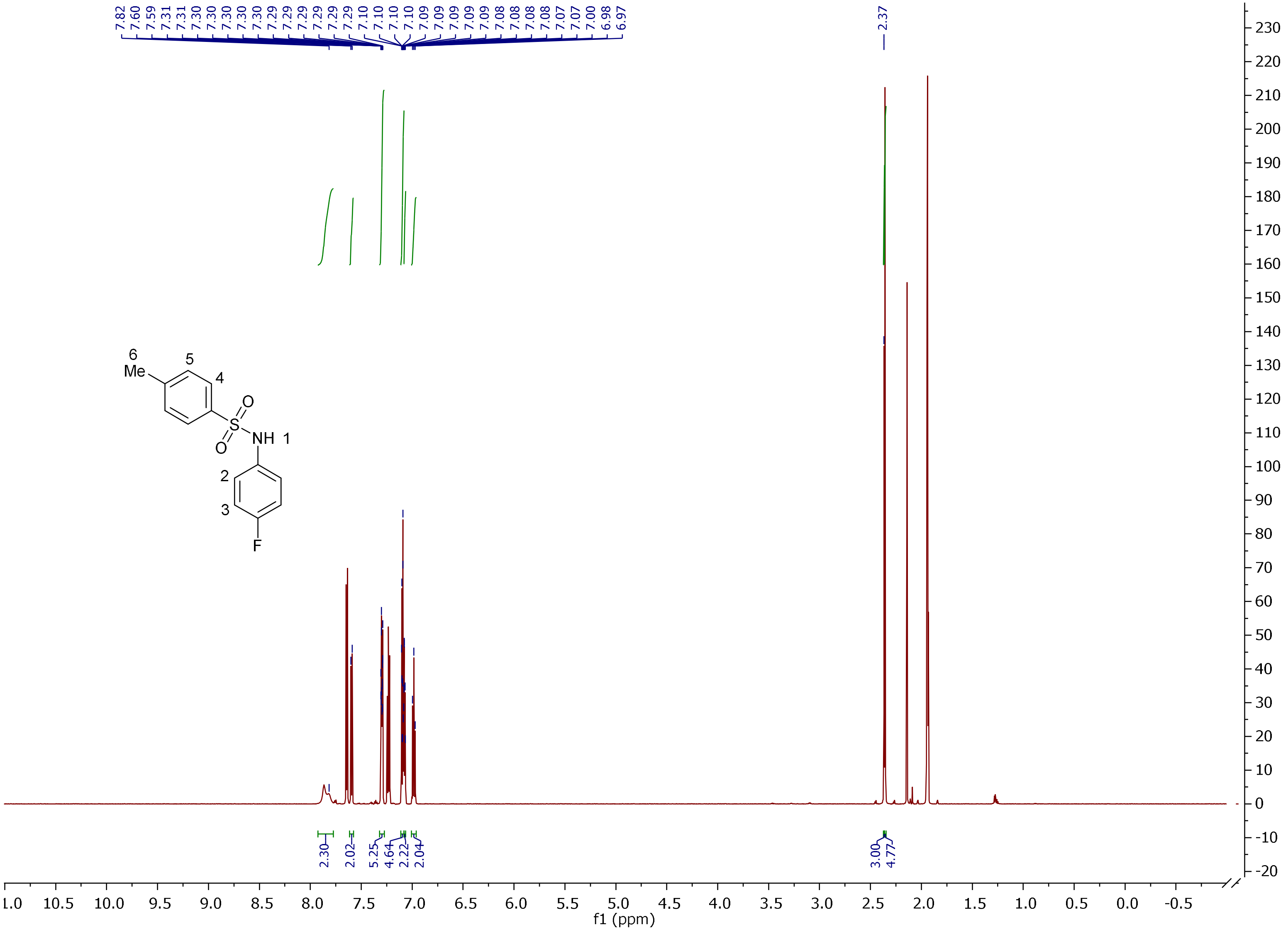

^{13}C NMR (176 MHz, CD_3CN) for fluorination of 4-methyl-*N*-phenylbenzenesulfonamide

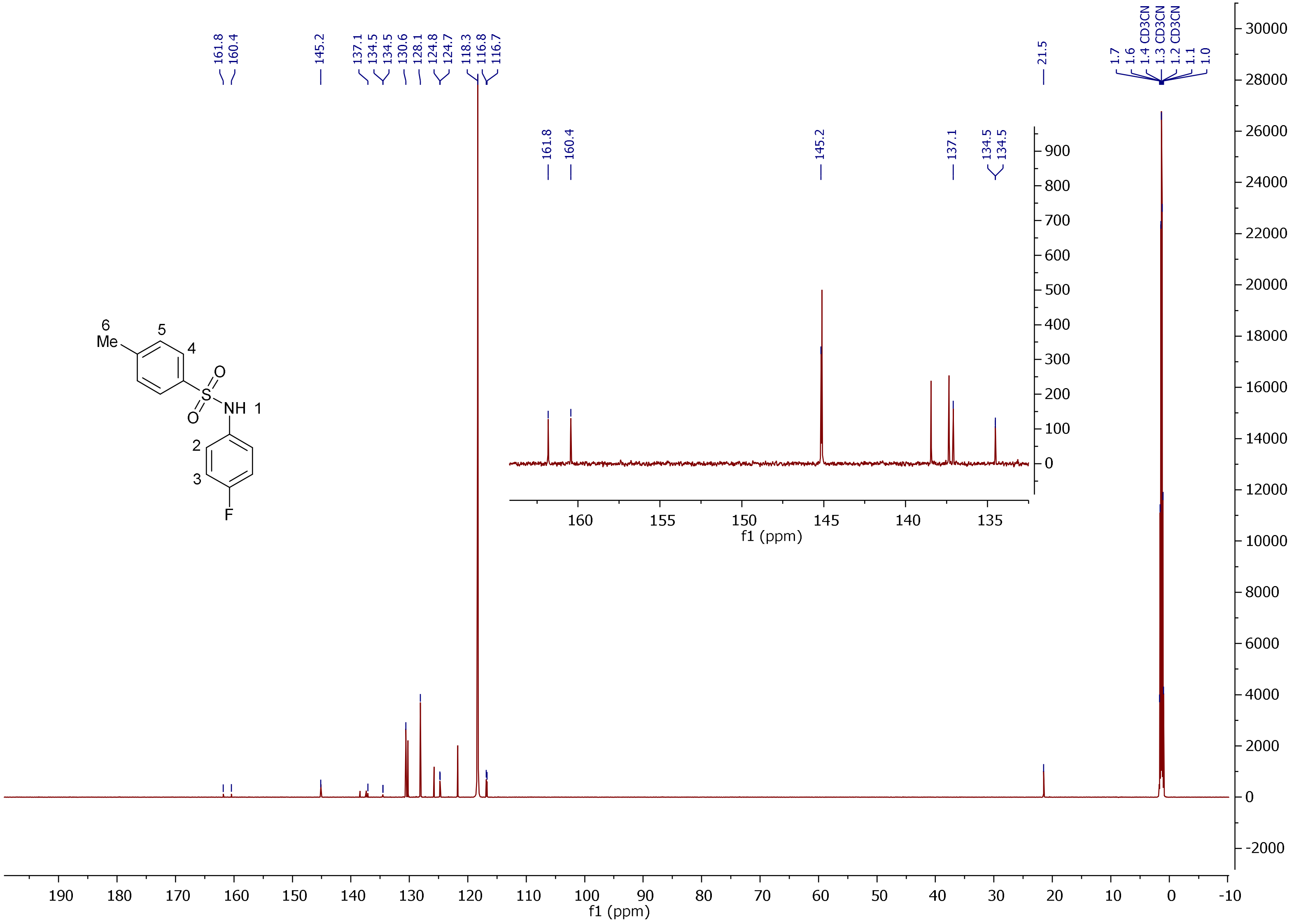

$^{19}F\{^1H\}$ NMR (376 MHz, CD_3CN) for fluorination of 4-methyl-*N*-phenylbenzenesulfonamide

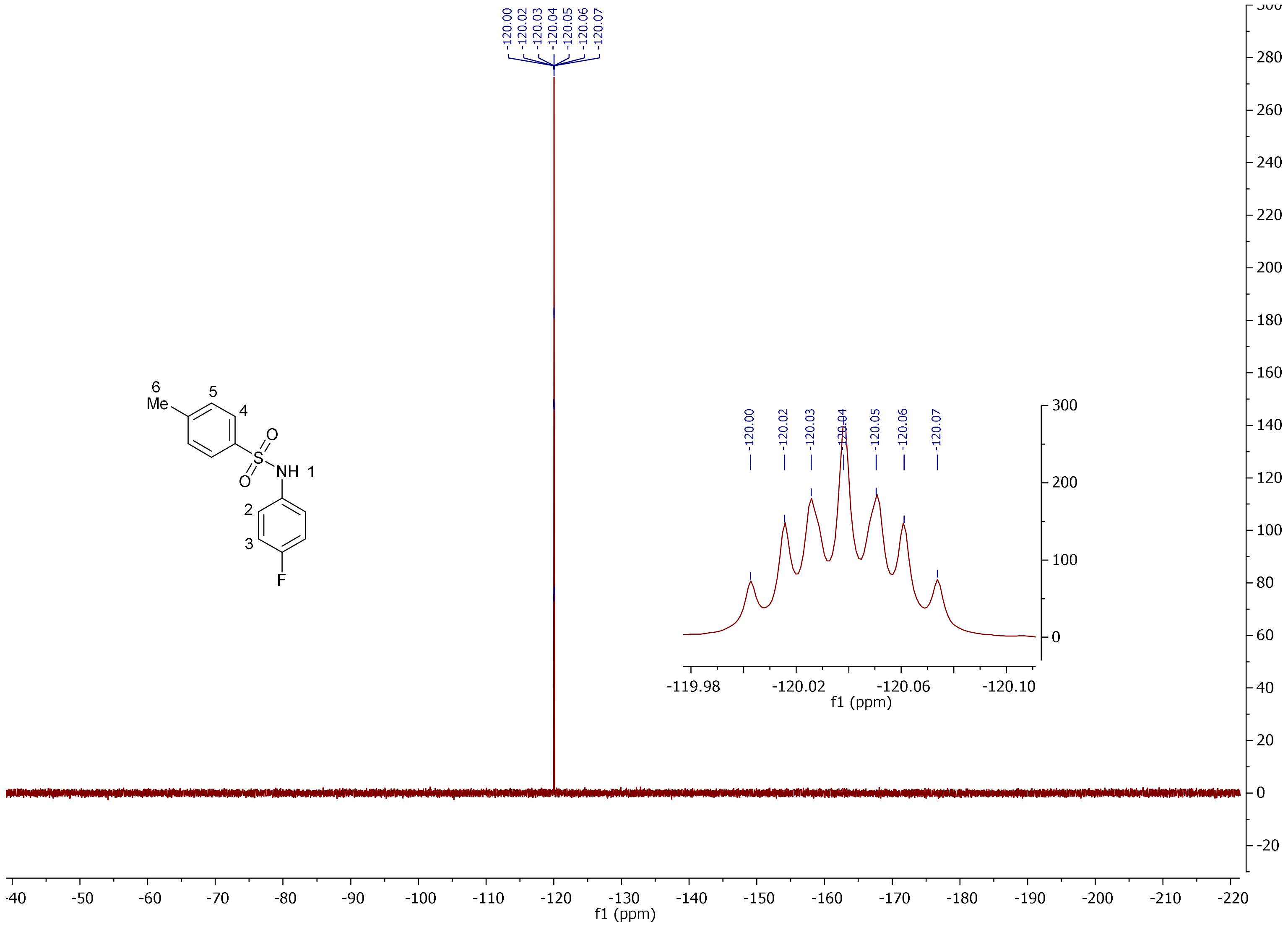

^{1}H NMR (700 MHz, CD_3CN) for fluorination of 2,2,2-trifluoro-*N*-(8-oxo-5,6,7,8-tetrahydronaphthalen-1-yl)acetamide **(3ac)**

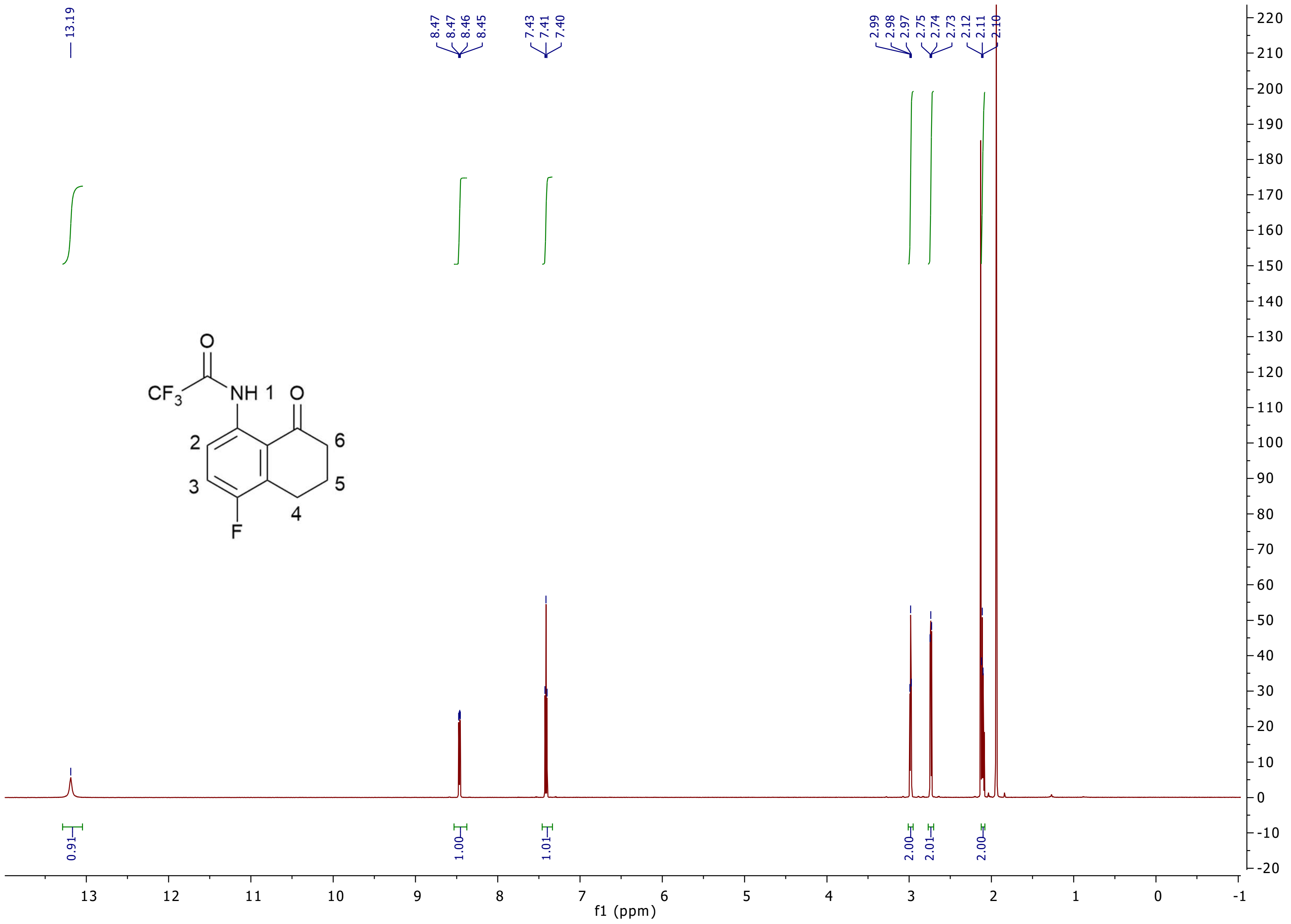

^{13}C NMR (176 MHz, CD_3CN) for fluorination of 2,2,2-trifluoro-*N*-(8-oxo-5,6,7,8-tetrahydronaphthalen-1-yl)acetamide **(3ac)**

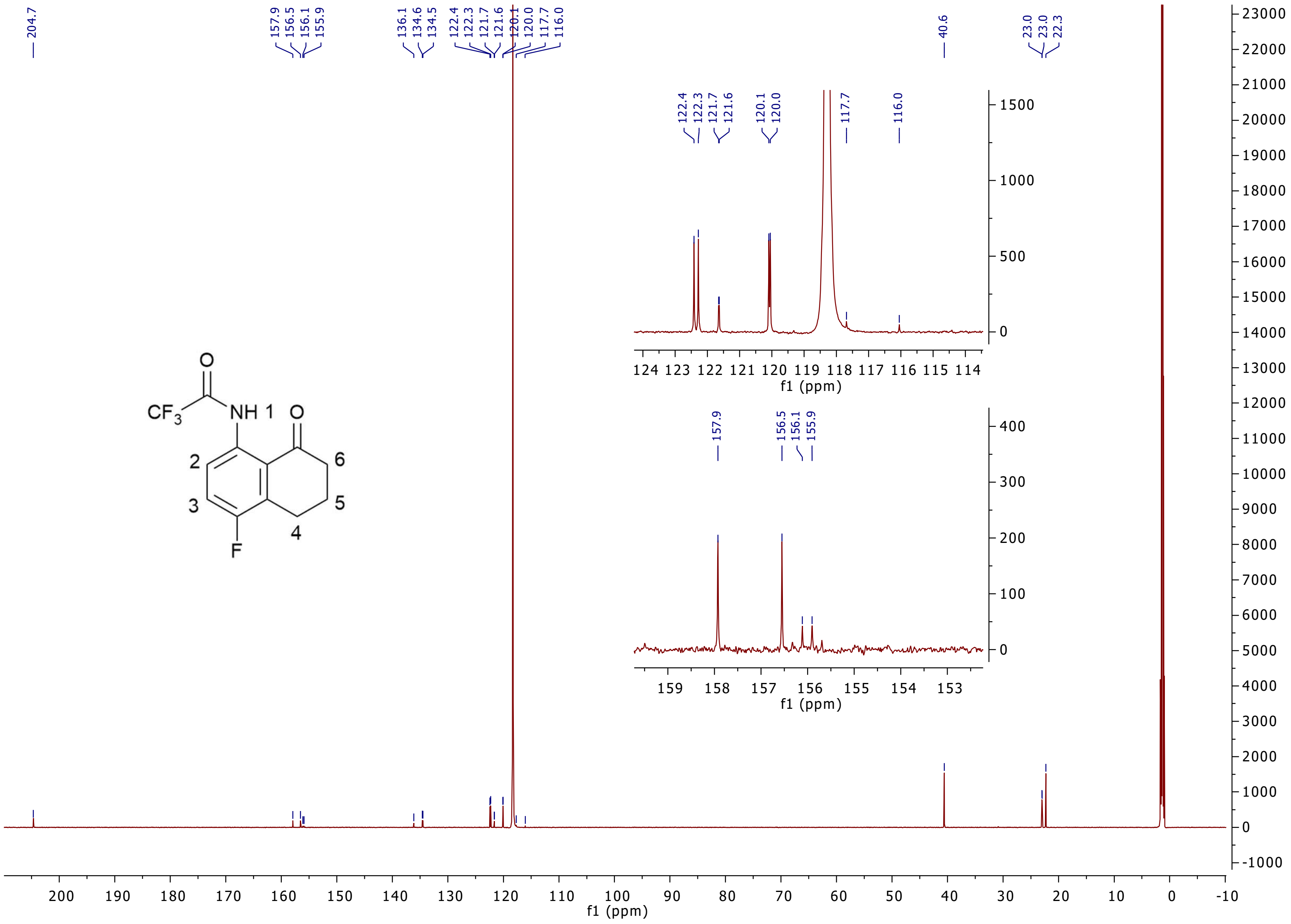

^{19}F{^{1}H} NMR (376 MHz, CD_3CN) for fluorination of 2,2,2-trifluoro-*N*-(8-oxo-5,6,7,8-tetrahydronaphthalen-1-yl)acetamide **(3ac)**

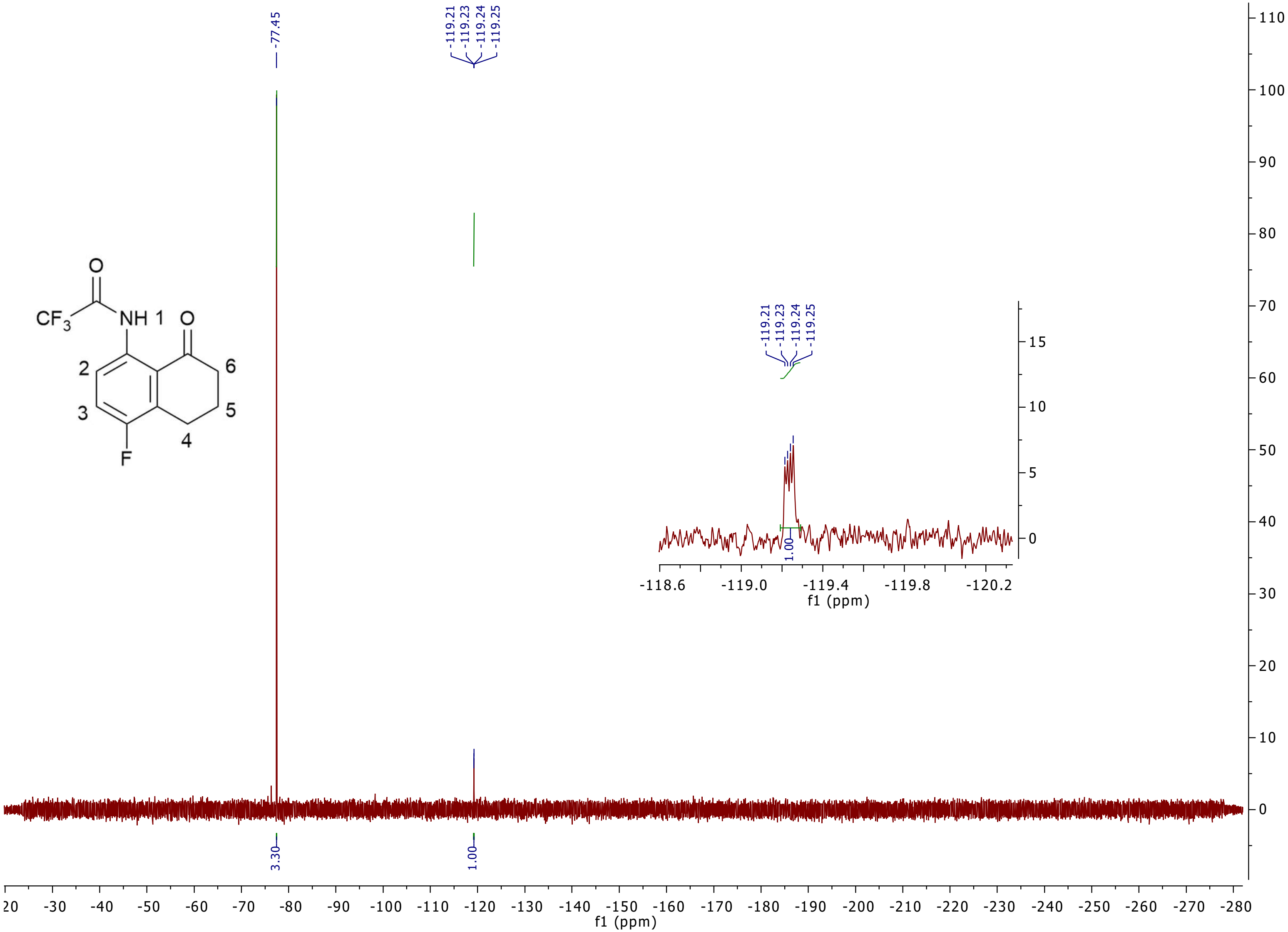

^{1}H NMR (400 MHz, $CDCl_3$) for *N*-(2-benzoylphenyl)-2,2,2-trifluoroacetamide from the 1.0 mmol scale reaction

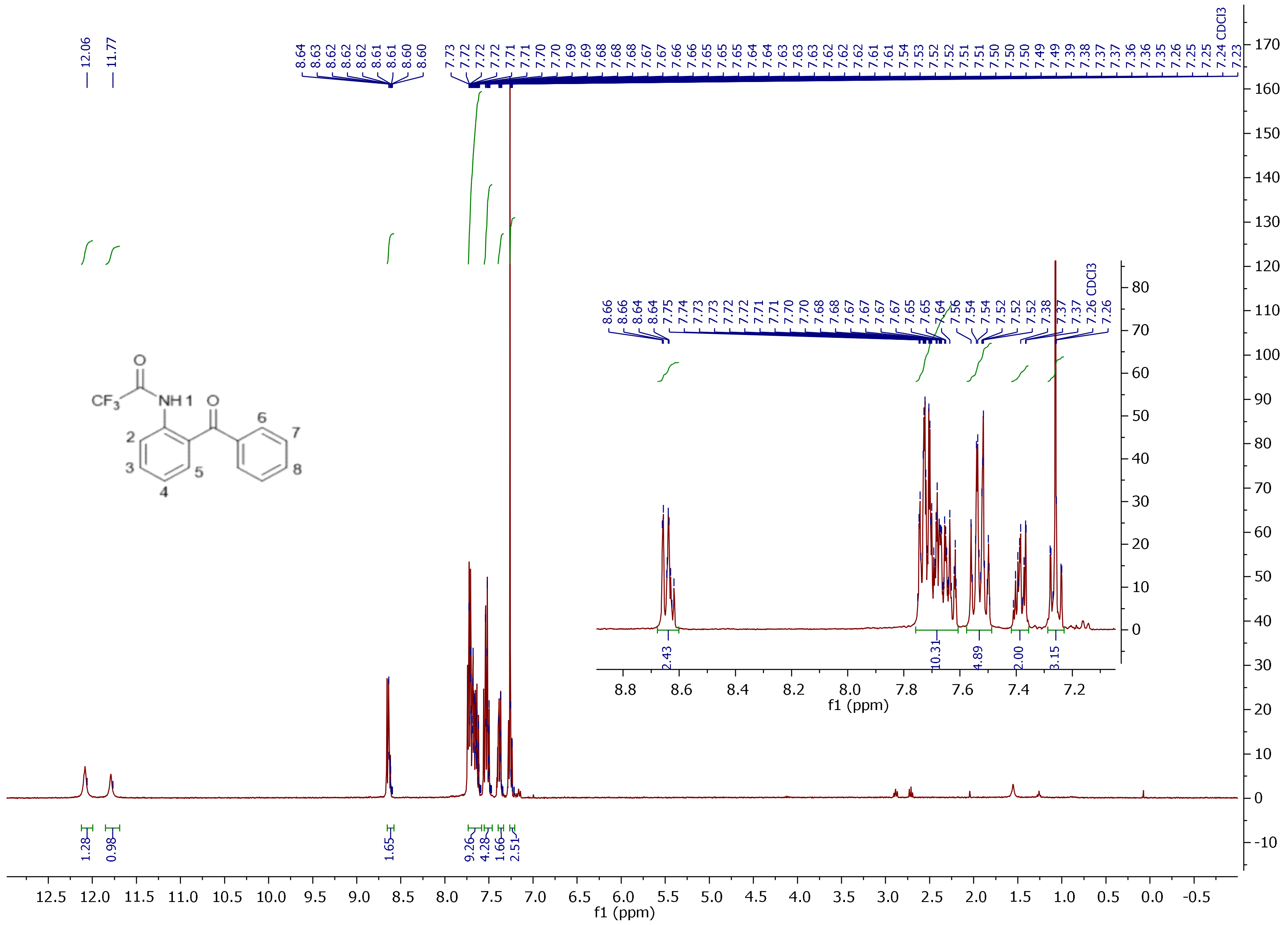

$^{19}F\{^{1}H\}$ NMR (376 MHz, $CDCl_3$) for *N*-(2-benzoylphenyl)-2,2,2-trifluoroacetamide from the 1.0 mmol scale reaction

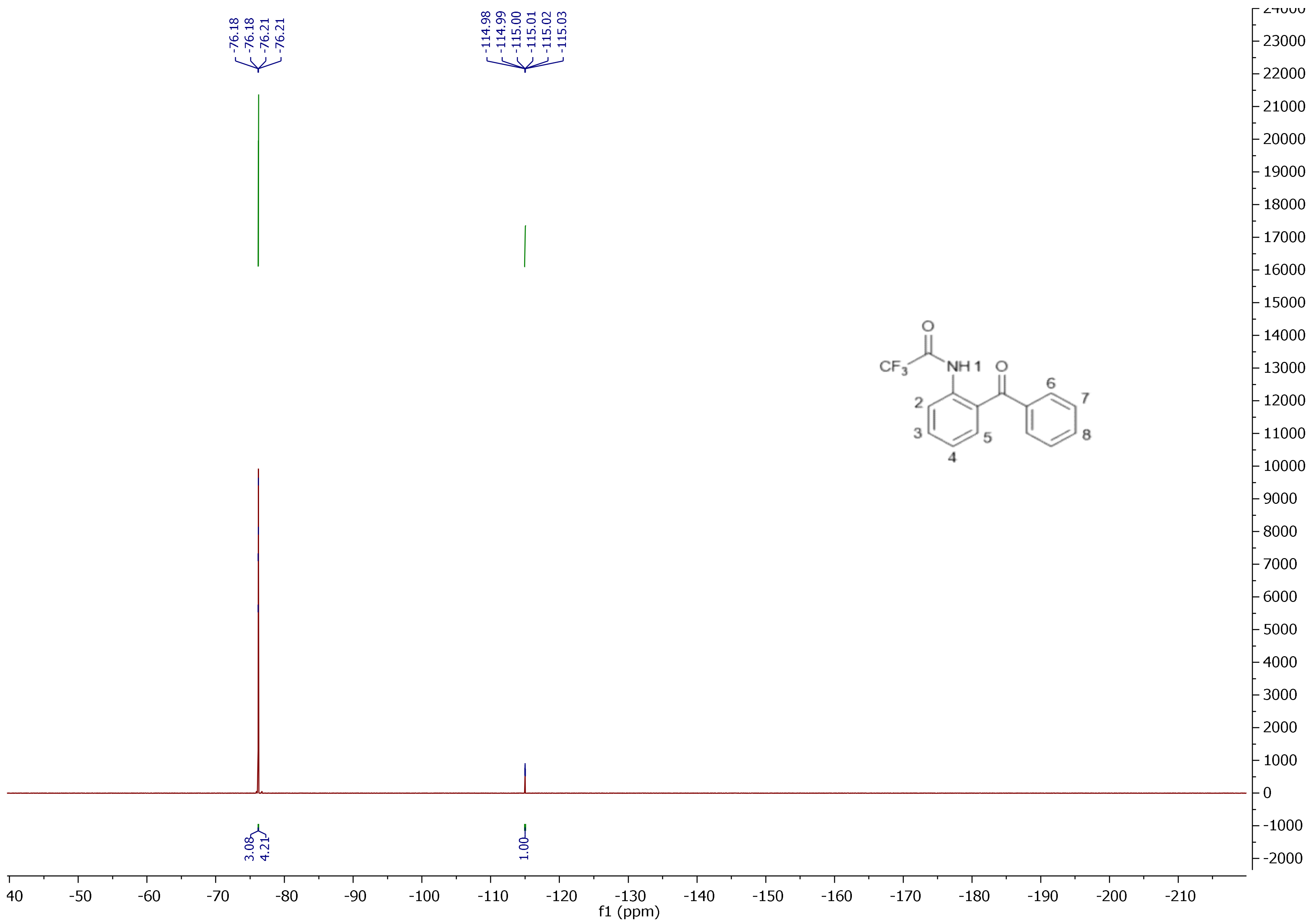

¹H NMR (400 MHz, $CDCl_3$) for 2,2,2-trifluoro-*N*-methyl-*N*-phenylacetamide **(1ah)**

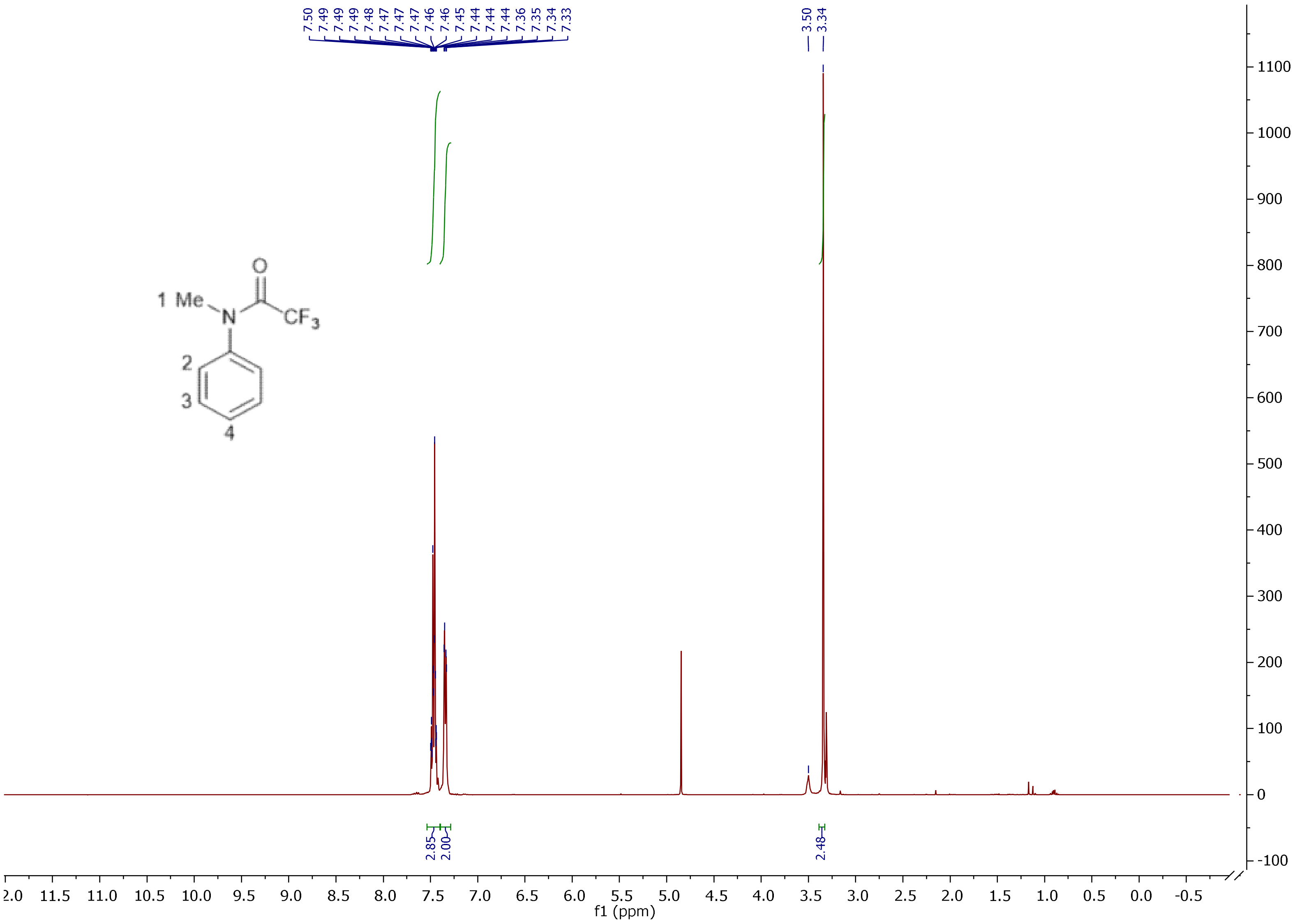

^{13}C NMR (400 MHz, $CDCl_3$) for 2,2,2-trifluoro-*N*-methyl-*N*-phenylacetamide **(1ah)**

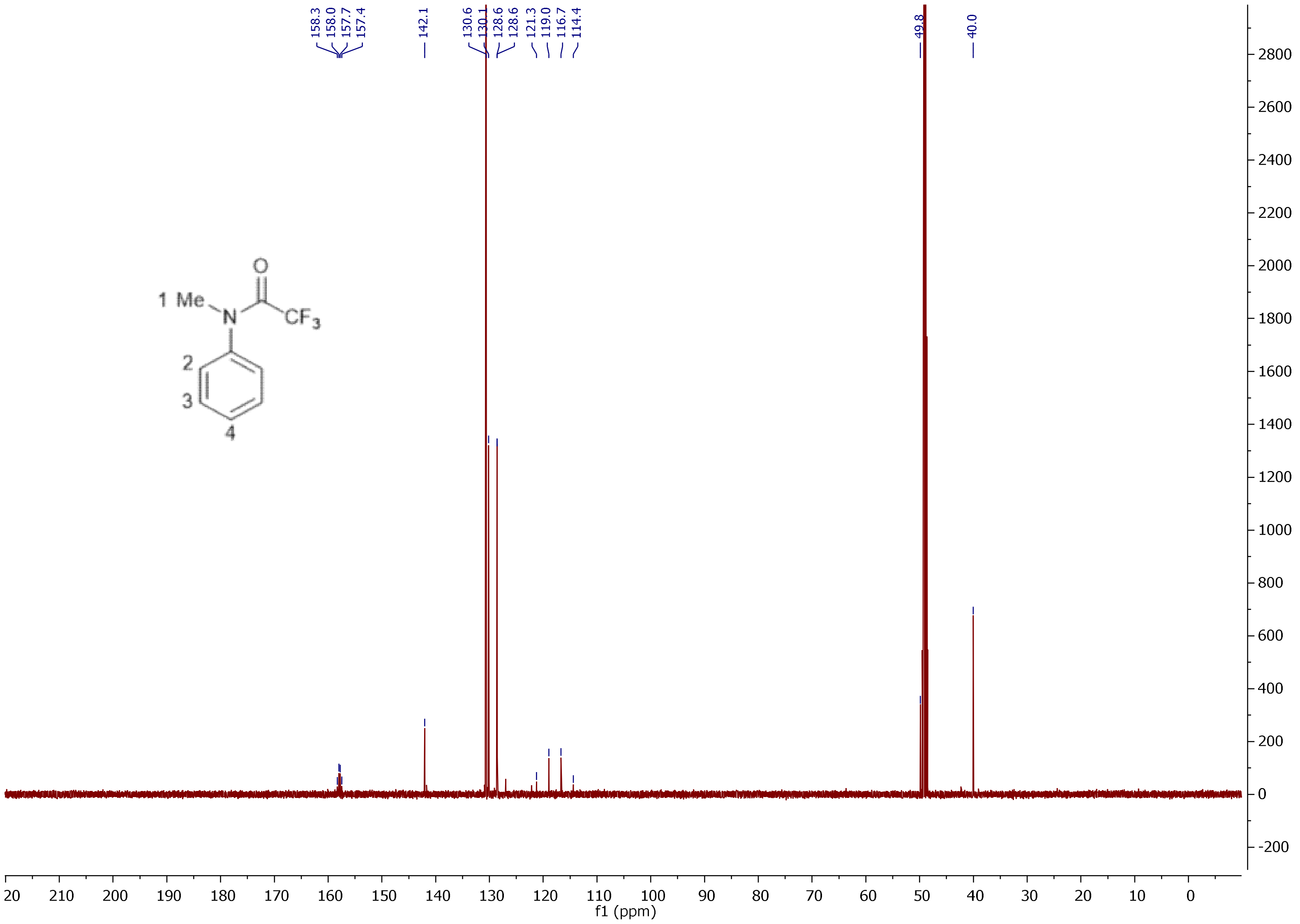

^{19}F NMR (376 MHz, $CDCl_3$) for 2,2,2-trifluoro-*N*-methyl-*N*-phenylacetamide **(1ah)**

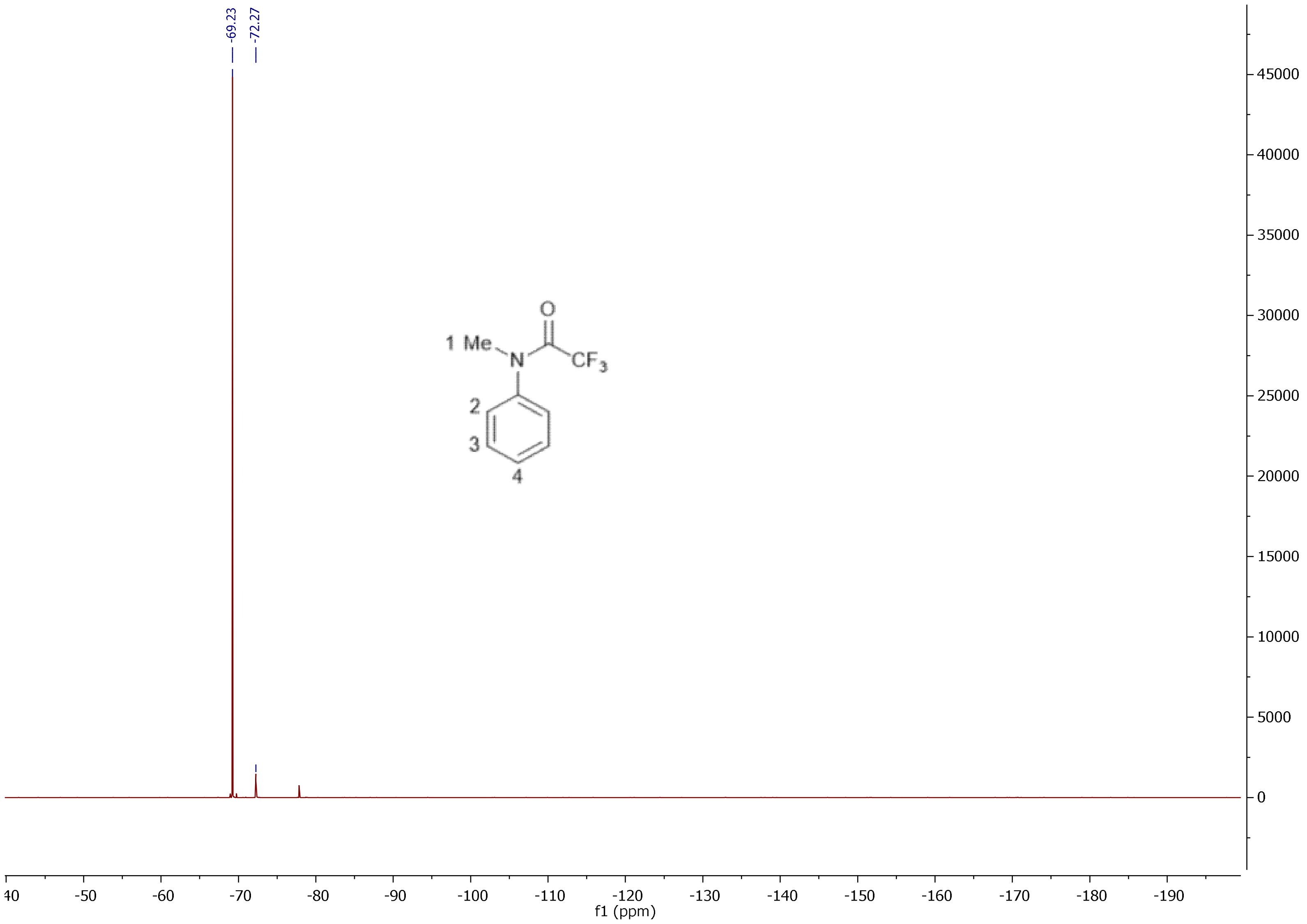

^{1}H NMR (400 MHz, $CDCl_3$) for fluorination of 2,2,2-trifluoro-*N*-methyl-*N*-phenylacetamide **(3ah)**

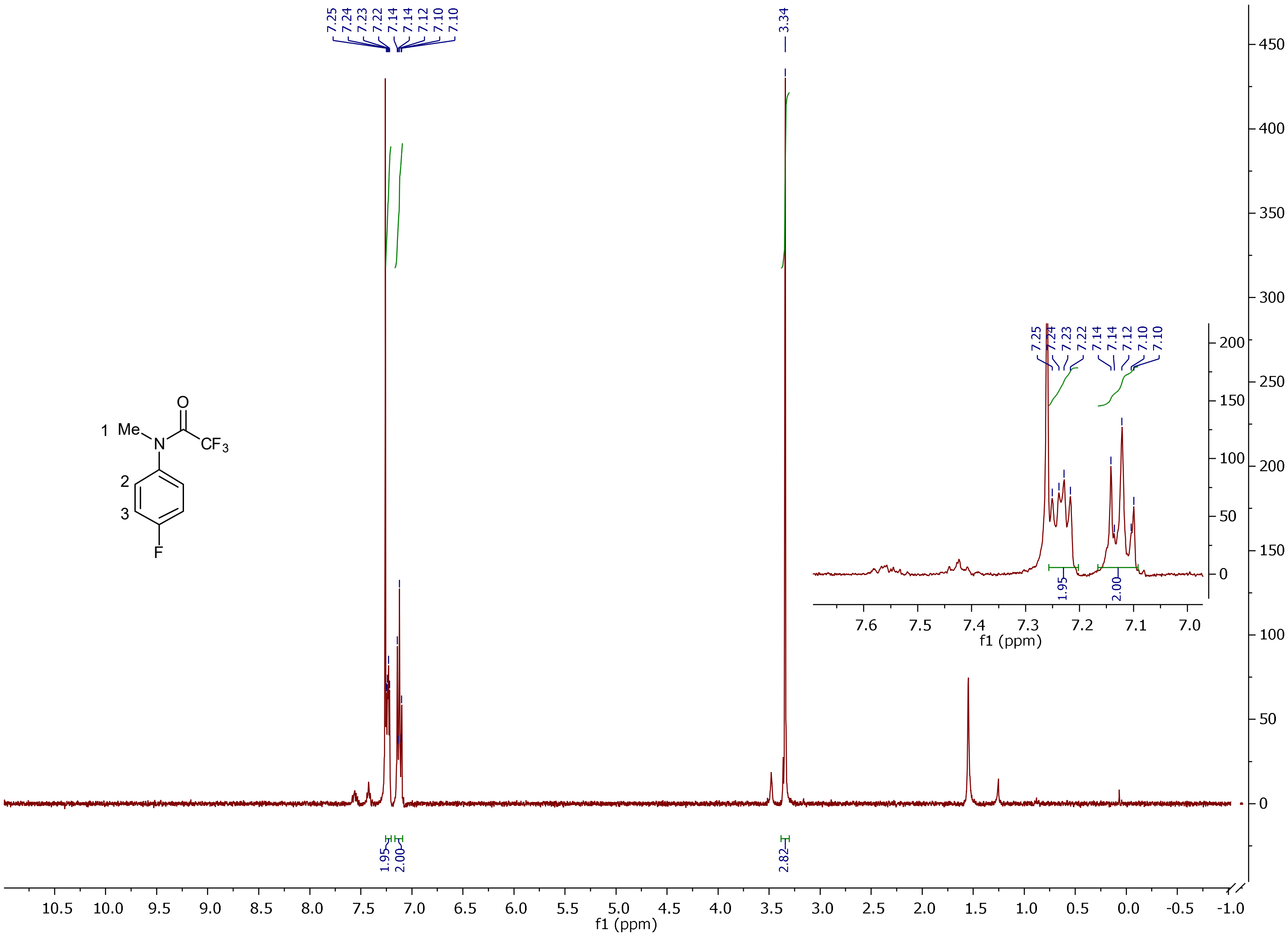

^{19}F NMR (376 MHz, $CDCl_3$) for fluorination of 2,2,2-trifluoro-*N*-methyl-*N*-phenylacetamide **(3ah)**

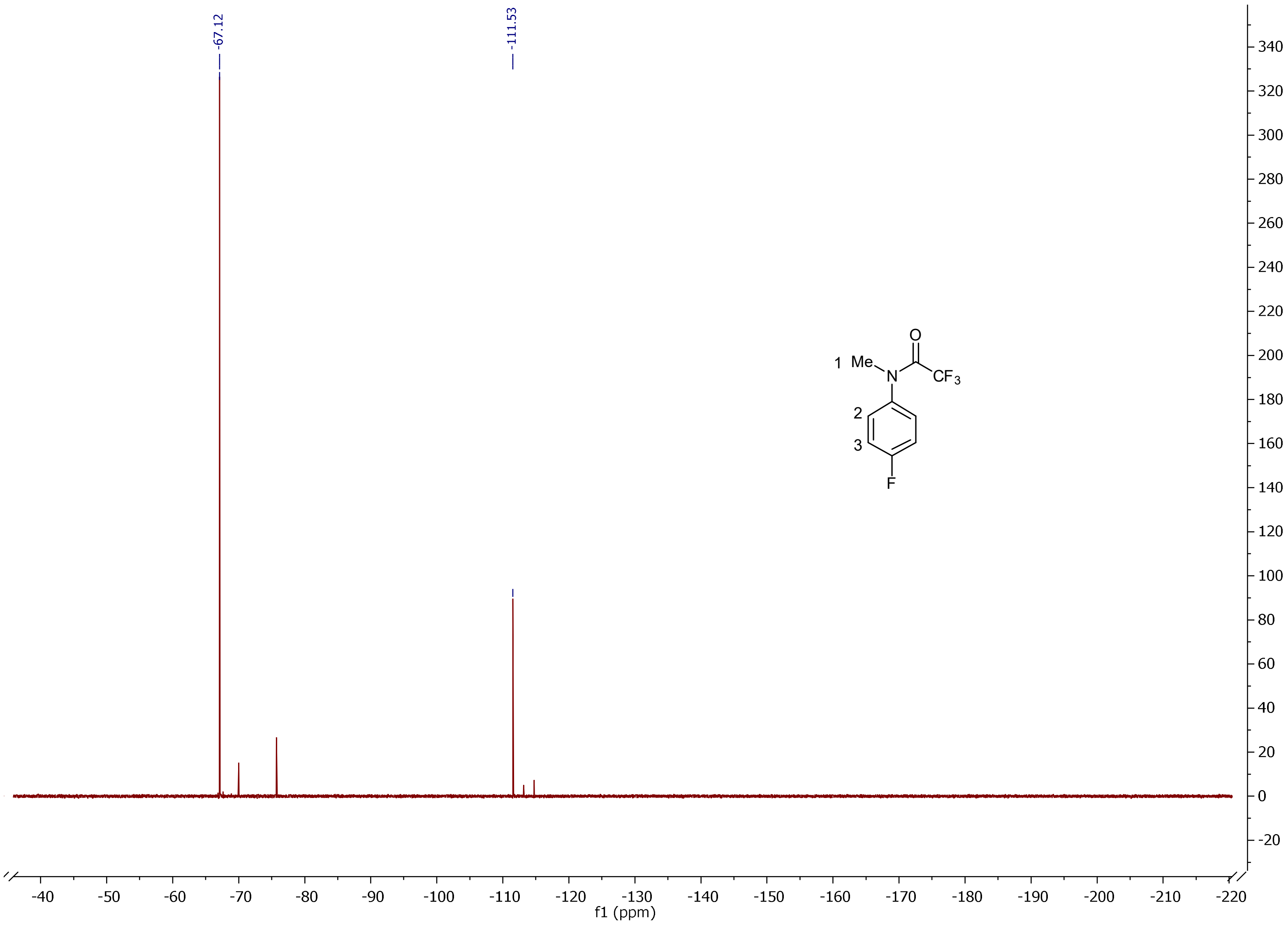

References

1 W. G. Kofron and L. M. Baclawski, *J. Org. Chem.*, 1976, **41**, 10, 1879-1880

2 Y.-Q. Fang and G. S. Hanan, *Synlett*, 2003, **2003**, 0852–0854.

3 Z. Dai, Z. Yu, Y. Bai, J. Li and J. Peng, *Applied Organometallic Chemistry*, 2020, **35**.

4 H. J. Davis, M. T. Mihai and R. J. Phipps, *J. Am. Chem. Soc.*, 2016, **138**, 12759–12762.

5 K. Yamamoto, J. Li, J. A. O. Garber, J. D. Rolfes, G. B. Boursalian, J. C. Borghs, C. Genicot, J. Jacq, M. van Gastel, F. Neese and T. Ritter, *Nature*, 2018, **554**, 511–514.

6 A. V. Iosub and S. S. Stahl, *Org. Lett.* 2015, **17**, 18, 4404–4407

7 H. J. Davis, G. R. Genov and R. J. Phipps, *Angew. Chem. Int. Ed.*, 2017, **56**, 13351–13355.

8 V. D. Duong, A. M. Horan and E. M. McGarrigle, *Org. Lett.* 2020, **22**, 21, 8451–8457

9 K. T. Potts, D. A. Usifer, A. Guadalupe and H. D. Abruna, *J. Am. Chem. Soc.*, 1987, **109**, 3961–3967.

10 J. E. Beves, C. J. Campbell, D. A. Leigh and R. G. Pritchard, *Angew. Chem. Int. Ed.*, 2013, **52**, 6464–6467.

11 N. D. Bogdan, M. Matache, V. M. Meier, C. Dobrotă, I. Dumitru, G. D. Roiban and D. P. Funeriu, *Chem. Eur. J.*, 2010, **16**, 2170–2180.

12 G. U. Priimov, P. Moore, P. K. Maritim, P. K. Butalanyi and N. W. Alcock, *J. Chem. Soc., Dalton Trans.*, 2000, 445–449.

13 J. Uenishi, T. Tanaka, K. Nishiwaki, S. Wakabayashi, S. Oae and H. Tsukube, *J. Org. Chem.*, 1993, **58**, 4382–4388.

14 R. Pollice, M. Bot, I. J. Kobylianskii, I. Shenderovich and P. Chen, *J. Am. Chem. Soc*., 2017, **139**, 37, 13126-13140

15 F. Camerel, B. Donnio, C. Bourgogne, M. Schmutz, D. Guillon, P. Davidson and R. Ziessel, *Chem. Eur. J.*, 2006, **12**, 4261–4274.

16 C. Bai, C.-T. Li, H.-M. Hu, B. Liu, J.-D. Li and G. Xue, *Dalton Trans.*, 2019, **48**, 814-817

17 J. B. Huang, X.-F. Bai, L. Li, Z.-J. Zheng, U. Xu, Y.-M. Cui, J. Cao and L.-W. Xu, *Chem. Eur. J.*, 2017, **23**, 4055-4059

18 Q. Ru, Z. Xue, Y. Wang, Y. Liu, and H. Li, *Eur. J. Inorg. Chem*., 2014, 469-474

19 G. Kaur, M. J. I. Polson and R. M. Hartshorn, *Dalton Trans*., 2015, **44**, 4200

20 A. R. Mazzotti, M. G. Campbell, P. Tang, J. M. Murphy and T. Ritter, *J. Am. Chem. Soc*. 2013, **135**, 38, 14012–14015

21 S. Deolka, M. H. Samha, A. G. Roca, G. C. Haug, J. R. Howard, D. Dalmau, J. Sandres, S. Vasylevskyi, R. T. VanderLinden, R. S. Paton, M. S. Sigman, *J. Am. Chem. Soc.* 2025, **147**, 15, 12878–12889

22 J. Li, J. Matsumoto, T. Otabe, C. Dohno, K. Nakatani, *Bioorg. Med. Chem*. 2015, **23**, 753-758

23 H. Feifei *et. al*., CN Pat., CN107118213, 2017

24 W. Sun, M.-P. Li, L.-J. Li, Q. Huang, M.-Y. Hu and S.-F. Zhu, *Chem. Sci*., 2022, **13**, 2721-2728
25 L. Tao et. al., CN Pat., CN117447467, 2024
26 J. F. J. Engbersen, A. Koudijs, M. H. A. Joosten and H. C. van der Plas, *J. Heterocycl. Chem.*, 1986, **23**, 989–990.
27 W. Verbeet, Y. Husiev and S. Bonnet, *Eur. J. Org. Chem*., 2024, 27, 14, e202400054
28 R. P. King and J. Y. Yang, *Chem. Sci*., 2023, **14**, 13530-13536
29 P.-F. Cao, J. Mangadlao, R. Advincula, *Angew. Chem. Int. Ed*., 2015, **54**, 5127-5131
30 Y. Ouyang, X. Yue, J. Peng, J. Zhu, Q. Shen and W. Li, *Org. Bimol. Chem*., 2022, 20, 6619
31 A. Colombo, G. Di Carlo, C. Dragonetti, M. Magni, A. O. Biroli, M. Pizzotti, D. Roberto, F. Tessore, E. Benazzi, C. A. Bignozzi, L. Casarin and S. Caramori, *Inorg. Chem*., 2017, **56**, 22, 14189-14197
32 R. Losa, C. Lorton, P. Retailleau, J. Bignon and A. Voituriez, *Org. Lett*., 2023, **25**, 27, 5140-5144
33 M. Tordeux, C. Francese and C. Wakselman, *J. Fluor. Chem*., 1989, **43**, 27-34
34 Z. Zheng, A. van der Werf, M. Deliaval, N. Selander, *Org. Lett*., 2020, **22**, 7, 2791-2796
35 R. Yamasaki, M. Harada, R. Nagata, A. Ito, K. Fukuda and I. Okamoto, *J. Org. Chem*., 2022, **87**, 13, 8469-8479
36 V. Kumar, S. Dhawan, P. S. Girase, P. Singh and R. Karpoormath, *Eur. J. Org. Chem*., 2021, 5627-5639
37 Y. Wan, Z. Zhang, N. Ma, J. Ni and G. Zhang, *J. Org. Chem*., 2019, **84**, 780-791
38 N. Cordua, S. R. Steffensen, A. G. Mascherpa, L. G. Christensen and H. H. Jensen, *Org. Lett*. 2025, **27**, 7, 1602–1607
39 S. E. López, Y. Pérez, J. Restrepo, J. Salazar and J. Charris, *J. Fluor. Chem*., 2007, **128**, 566-569
40 M. O. Kitching, O. E. Dixon, M. Baumann and I. R. Baxendale, *Eur. J. Org. Chem*., 2017, 6540-6553
41 R. Sanz, V. Guilarte and N. García, *Org. Biomol. Chem*., 2010, **8**, 3860-3864
42 J. C. Holder, E. D. Goodman, K. Kikushima, M. Gatti, A. X. Marziale and B. M. Stolz, *Tetrahedron*, 2015, **71**, 5781-5792
43 G. Monzón, I. Tirotta and P. Knochel, *Angew. Chem. Int. Ed*., 2012, **51**, 10624-10627
44 Y. M. Mukhtar, Y. Huang, J. Liu, D. Chen and W. Zheng, *Bioorg. Med. Chem. Lett*., 2017, **27**, 11, 2319-2323
45 C.-Z. Tao, J. Li, Y. Fu, L. Liu and Q.-X. Guo, *Tetrahedron Lett.*, 2008, **49**, 70-75
46 C. Liu, K. Li and R. Shang, *ACS Catal*. 2022, **12**, 7, 4103–4109
47 J. Zhang, J. Zhang, G. Hao, W. Xin, F. Yang, M. Zhu and H. Zhou, *J. Med. Chem*., 2019, **62**, 14, 6765-6784
48 M. G. N. Russell, R. W. Carling, L. J. Street, D. J. Hallett, S. Goodacre, E. Mezzogori, M. Reader, S. M. Cook, F. A. Bromidge, R. Newman, A. J. Smith, K. A. Wafford, G. R. Marshall, D. S. Reynolds, R. Dias, P. Ferris, J. Stanley, R. Lincoln, S. J. Tye, W. F. A. Sheppard, B. Sohal, A.

Pike, M. Dominguez, J. R. Atack and J. L. Castro, *Journal of Medicinal Chemistry*, 2006, **49**, 1235–1238.

49 R. Pellicciari, IT Pat., WO2021170658A1, 2021

50 D. S. Baranov, A. S. Smorygina and S. A. Dzuba, *Molecules*, 2022, **27**, 4127.

51 D.-Q. Song, Y. Wang, L.-Z. Wu, P. Yang, Y.-M. Wang, L.-M- Gao, Y. Li, J.-R. Qu, Y.-H. Wang, Y.-H. Li, N.-N. Du, Y.-X. Han, Z.-P. Zhang and J.-D. Jiang, *J. Med. Chem.* 2008, **51**, 11, 3094–3103

52 S. Kur, G. Mohiuddin, N. Yadav and S. K. Pal, *ChemPhysChem*, 2023, **24**, e202300133

53 D. W. Sopher, W. Eilenberg and J. H. H. Meurs, *Angew. Chem*., 1989, **101**, 7, 955-956

54 F. Yang, D. Ding and C. Wang, *Org. Lett*. 2020, **22**, 23, 9203–9209

55 J. Chaturvedi, C. Haldar, R. Bisht, G. Pandey and B. Chattopadhya, *J. Am. Chem. Soc*. 2021, **143**, 20, 7604–7611

56 T. Okawa, Y. Aramaki, M. Yamamoto, T. Kobayashi, S. Fukumoto, Y. Toyoda, T. Henta, A. Hata, S. Ikeda, M. Kaneko, I. D. Hoffman, B.-C. Sang, H. Zou, T. Kawamoto, *J. Med. Chem*., 2017, **60**, 16, 6942–6990

57 G. Jimming, US Pat., WO2017106624A1, 2017

58 T. Namba, M. Hotta, H. Tabata, K. Makino, T. Oshitari, H. Natsugari, H. Takahasni, *J. Org. Chem*. 2021, **86**, 11, 7563–7578

59 C.-Z. Tao, J. Li, Y. Fu, L. Liue and Q.-X. Guo, *Tetrahedron Lett*., 2008, **49**, 1, 70-75

60 X. Chen, Y. Liu, S. Zhang, Y. Li, X.-Y. Zhou, X. Feng, X. Yu, Y.Yamamoto and M. Bao, *Org. Lett*. 2024, **26**, 34, 7233–7238
